# Gallstones as a predictor of elevated cardiovascular disease risk: A meta-analysis and meta-regression of over 7.4 million participants

**DOI:** 10.1371/journal.pone.0314661

**Published:** 2025-03-19

**Authors:** Refli Hasan, Fatemeh Allahbakhshi, Andrey D. Shlyk, Khadija Allahbakhshi

**Affiliations:** 1 Department of Internal Medicine, Faculty of Medicine, Universitas Sumatera Utara, Medan, Indonesia; 2 Department of Cardiology, School of Medicine, Tehran University of Medical Sciences, Tehran, Iran; 3 Department of Prosthetic Dentistry, I.M. Sechenov First Moscow State Medical University (Sechenov University), Moscow, Russia; 4 Department of Internal Medicine, School of Medicine, Isfahan University of Medical Sciences, Isfahan, Iran; Campus Bio-Medico University of Rome, ITALY

## Abstract

**Introduction:**

Gallstone disease (GD) is a prevalent condition frequently encountered in surgical units worldwide. The objective of this comprehensive systematic review and meta-analysis study was to examine the relationship between gallstones and the risk of cardiovascular diseases (CVDs).

**Methods:**

To conduct our study, we performed a systematic review and meta-analysis. We gathered relevant studies from reputable databases, including Web of Science, Scopus, PubMed, Cochrane, Google Scholar, and Embase. The quality of the articles was assessed using the Newcastle-Ottawa Scale checklist. To assess heterogeneity among the studies, we utilized statistical tests such as the Chi-square test, I² statistic, and forest plots. Meta-regression analysis considered variables such as the year of the study, study design, sample size, study quality assessment score, geographical region, average age of subjects, and follow-up duration. Additionally, we evaluated publication bias using Begg’s and Egger’s tests.

**Results:**

Data from 22 studies conducted between 1985 and 2023 were analyzed. The combined number of participants across these studies was 7,496,303. The meta-analysis results revealed that individuals with GD had a higher risk of CVDs (Risk Ratio (RR): 1.29; 95% CI: 1.22–1.36; P <  0.001). Subgroup analysis showed consistent results across good quality studies (RR: 1.20, 95% CI: 11.12–1.28; P <  0.001), moderate quality studies (RR: 1.41, 95% CI: 1.15–1.74; P <  0.001), and low-quality studies (RR: 1.22, 95% CI: 1.15–1.30; P <  0.001). In the meta-regression analysis, none of the variables had a significant relationship with the observed heterogeneity (P-value > 0.10). In a sensitivity analysis, the estimated RR remained consistent, confirming the robustness of the meta-analysis results.

**Conclusion:**

Our findings suggest an association between gallstone disease and an increased risk of CVDs. It seems that one of the important factors of this relationship is having common causes for the formation of gallstones and cardiovascular diseases. However, gallstones can be considered an important sign of increased risk of cardiovascular diseases.

## Introduction

Gallstone disease (GD) is a common condition frequently encountered in surgical units worldwide [1]. Epidemiological research indicates that approximately 10–15% of adults in Western populations have detectable GD via imaging modalities such as ultrasound or CT scan [2]. The prevalence of GD has been steadily increasing in recent decades, paralleling the rise in obesity rates [3].

Individuals with GD consistently exhibit a higher prevalence of established risk factors for cardiovascular diseases (CVDs) compared to those without GD [4]. These risk factors include obesity, elevated blood pressure, dyslipidemia, insulin resistance, and components of the metabolic syndrome, such as central adiposity, which are more commonly observed in GD patients [4–6].

Several primary studies have established a link between GD and subclinical atherosclerosis [7]. Research utilizing ultrasound to measure carotid intima-media thickness (IMT) and the presence of plaque as markers for preclinical CVDs has shown that individuals with GD have higher IMT and plaque scores than those without GD [7,8]. Additionally, observational studies suggest that individuals with GD may face an increased risk of future cardiovascular events requiring hospitalization, such as myocardial infarction or ischemic stroke [9,10]. The chronic pro-inflammatory state associated with GD is thought to contribute to long-term endothelial dysfunction and the progression of atherogenesis [11–13].

While some primary studies have reported an association between GD and increased risk of CVD events [9,14–16], not all have found statistically significant results [10,17]. This inconsistency in findings has led to the need for meta-analyses to synthesize the available evidence.

Several meta-analyses have been conducted to explore the relationship between GD and CVDs risk. For instance, Fairfield et al. conducted a systematic review and meta-analysis of 8 studies, reporting a hazard ratio (HR) of 1.23 (95% CI: 1.16–1.30) for CVDs in people with GD [18]. This association was consistent across subgroups based on gender and diabetes status. Similarly, Upala et al. performed a meta-analysis of 6 studies, finding a odds ratio (OR) of 1.82 (95% CI: 1.47–2.24) for CVDs in individuals with GD [19]. These findings suggest a modest but statistically significant association between GD and CVDs.

Despite these findings, the strength of associations reported varies across studies, and some meta-analyses have noted limitations. For instance, previous meta-analyses have not sufficiently controlled for confounding factors, potentially introducing bias. Additionally, the small number of studies included has limited the ability to conduct detailed subgroup analyses and meta-regression models, restricting a deeper understanding of the relationship between GD and CVDs.

To address these limitations, we conducted a systematic review and meta-analysis of observational studies investigating the association between GD and CVDs risk. Our study aims to provide a more comprehensive assessment by incorporating a larger number of studies and enabling subgroup analyses based on key characteristics. Additionally, we sought to rigorously control for potential confounding variables that may influence the observed associations.

In summary, while previous meta-analyses have demonstrated a modest association between GD and CVDs risk, the evidence remains inconclusive due to study design and analysis limitations. Our systematic review and meta-analysis seek to provide a more robust and detailed understanding of the relationship between GD and CVDs by addressing potential sources of heterogeneity and confounding.

## Materials and methods

### Study design and search strategies

For this systematic review and meta-analysis, we strictly adhered to the guidelines set forth by the Preferred Reporting Items for Systematic Reviews and Meta-Analyses (PRISMA). The review was not registered, and no formal protocol was prepared or published. To ensure comprehensiveness, we conducted an extensive literature search across multiple databases, including Web of Science, PubMed, Cochrane Central Register of Controlled Trials, Embase, Google Scholar, and Scopus. The search included articles from the inception of these databases until June 30th, 2024, ensuring the inclusion of the most recent and relevant studies (See Supplementary files: S1 Tables and S1 Checklist for details).

Our objective was to examine the relationship between GD and the risk of CVDs. To identify relevant keywords and their synonyms pertaining to GD and CVDs, we utilized Medical Subject Headings (MeSH) terms. We included only full-text, original research articles that involved human subjects and provided quantitative analyses of the associations using effect size indices such as odds ratio (OR), hazard ratio (HR), and risk ratio (RR).

To ensure comprehensive search results, we combined keywords using Boolean operators (AND, OR). Additionally, we manually reviewed the reference lists of the included studies to identify any relevant publications that may have been missed during the initial database searches.

### Inclusion and exclusion criteria

To ensure the inclusion of high-quality studies, we applied stringent criteria. Eligible studies were original research articles published in peer-reviewed journals and employed study designs such as case-control, cross-sectional, and retrospective/prospective cohort studies. These designs were deemed suitable for associating the effects of gallstones on cardiovascular outcomes.

To enable an objective evaluation of the associations, studies had to report quantitative effect size estimates along with their 95% confidence intervals. Excluded from the review were letters, editorials, case reports, reviews, and studies that did not involve human subjects or inadequately measured exposure and outcome variables. Studies with incomplete or unclear data reporting and those that lacked sufficient effect estimates or raw data for quantitative meta-analysis were also excluded.

### Article selection process

After conducting comprehensive literature searches, we meticulously checked the records for duplicates using Endnote reference management software [20]. This software automatically identified duplicate records based on matching titles, author names, and publication years. Additionally, the titles of all records were manually cross-checked in an Excel spreadsheet by two independent reviewers.

Two independent reviewers systematically screened the titles, abstracts, and full texts of the studies. The first stage of study selection involved screening the titles to exclude clearly irrelevant articles. Subsequently, the abstracts of the remaining articles were examined to further exclude studies that did not measure the key exposure and outcome variables or utilized ineligible study designs. The full texts of the remaining articles underwent the most rigorous screening stage, during which both independent reviewers assessed each study against the predefined inclusion and exclusion criteria. In cases of disagreements between reviewers at any screening stage, a third independent reviewer was consulted to facilitate a consensus decision.

### Data extraction

To maintain the integrity and consistency of the data, we developed a comprehensive and standardized data extraction form before initiating the extraction process. This form underwent extensive pilot testing by our research team to ensure its effectiveness in capturing all essential study details in an organized and reproducible manner.

Two independent reviewers utilized this standardized form to systematically extract relevant information from each included study. To ensure accuracy, the extracted data were meticulously cross-checked by both reviewers. Any discrepancies were resolved through open discussion and consensus decision-making, involving a third reviewer if necessary.

The comprehensive data extraction form collected critical bibliographic details, study characteristics, population descriptors, reported effect estimates, adjusted covariates, and quality assessment ratings. By capturing this information in a standardized manner, we ensured consistency and completeness across all studies.

### Evaluation of study quality

The methodological quality of the included observational studies was assessed using the widely accepted Newcastle-Ottawa Scale (NOS) [21]. This scale evaluates non-randomized studies based on three key domains: selection of study groups, comparability between groups, and ascertainment of exposure and outcome variables. Each study was assigned a quality rating on a scale ranging from zero to nine points. Scores below 5 indicated low-quality articles, scores between 5 and 7 indicated moderate quality, and scores of 8 or higher indicated good quality [22,23].

### Statistical methods

For studies that presented separate effect estimates for different exposure periods or were stratified by key covariates but did not report an overall estimate, we conducted a meta-analysis to combine the stratified effects. Moreover, for studies that provided raw exposure and outcome data without calculated effect sizes, we utilized Stata software to generate effect estimates along with 95% confidence intervals.

To evaluate heterogeneity between studies, we employed both statistical tests and visual examination of forest plots. The Chi-square test was used to determine if observed differences were statistically significant, with a significance level of P < 0.05. Additionally, we calculated the I² statistic to quantify the proportion of total variation attributable to heterogeneity rather than sampling error. When significant heterogeneity was detected, we employed random-effects models for the meta-analyses. We carefully examined forest plots to visually assess the overlap and distribution of confidence intervals across studies. Any potential outliers were further investigated through meta-regression models, subgroup analyses, and sensitivity analyses to identify potential sources of heterogeneity.

To explore the influence of covariates on heterogeneity, we conducted univariate and multivariate meta-regression analyses using Stata software. Subgroup analyses were also performed, grouping studies based on variables such as gender (male, female), study location (Asian vs. non-Asian countries), study quality (low, medium, high), study design (cohort vs. case-control/cross-sectional), sample size (<10,000 vs. ≥ 10,000), and average age (<50 vs. ≥ 50 years). For continuous variables, such as the year of the study and the average follow-up period, we balanced the subgroup groupings to ensure a similar number of studies in each category. This approach helped to maximize the statistical power of the analysis.

Sensitivity analyses were performed by excluding each study individually to assess the impact of any single study on the overall results. To evaluate potential publication bias, we inspected funnel plots for asymmetry and conducted Egger’s and Begg’s tests. In cases where publication bias was observed, we employed the trim-and-fill method to estimate the effect size of the missing studies and incorporate their corresponding effects into the meta-analysis [24]. In cases of significant heterogeneity, we reported the most conservative results based on the fixed and random-effects models with the highest level of significance [25–27]. Variables with missing data were excluded from analyses that required complete datasets, such as meta-regression and subgroup analyses, to ensure the accuracy and reliability of the statistical results. Meta-regression and subgroup analyses depend on complete datasets for each included variable to produce valid estimates and interpretations. Including variables with incomplete data in these analyses could lead to biased estimates, reduced statistical power, and incorrect conclusions. To avoid these issues, we restricted these analyses to studies or variables with fully available data. All data analyses were conducted using STATA version 14.1 (Stata Corp, College Station, Texas).

## Results

### Characteristics of included studies

**Fig 1:**
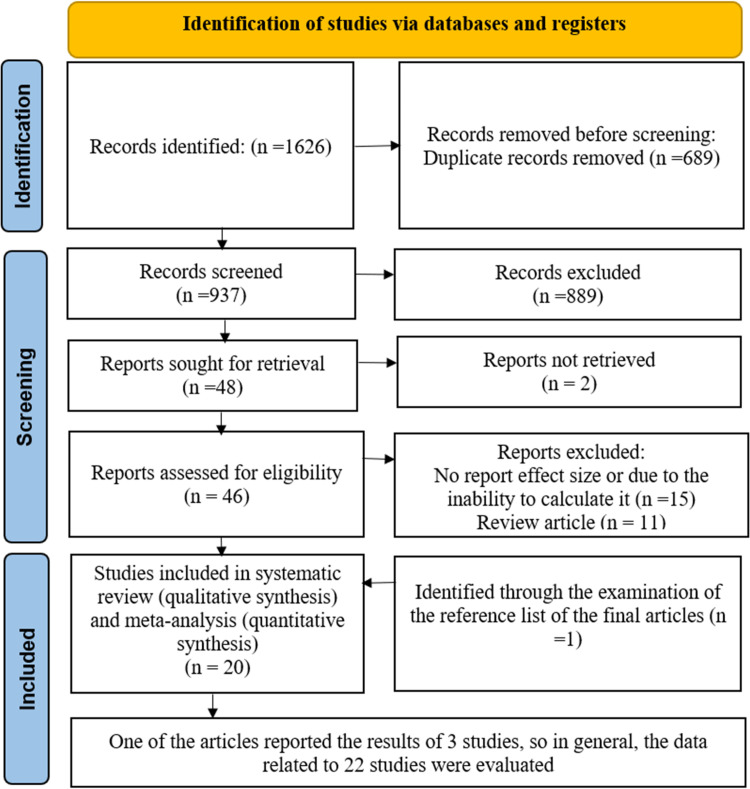
Flowchart depicting the selected studies for meta-analysis.

We conducted a comprehensive electronic search using targeted keywords, which initially yielded 1,626 articles. After removing 689 duplicate entries, we screened the remaining 937 articles against our predefined inclusion and exclusion criteria. This screening process led to the exclusion of 889 articles that did not meet the established standards. Consequently, we identified 48 relevant studies. However, upon further review, 15 studies were excluded because they did not report effect sizes or lacked sufficient information to calculate them, and 11 were excluded as they were review articles. Additionally, 2 articles were unavailable in full text and were therefore removed from the analysis. Ultimately, our systematic review and meta-analysis included 19 studies. One additional study was identified through reference checks, resulting in a total of 20 articles in the final analysis [9,10,12,14–17,28–40]. One of the articles reported the results of 3 studies, so in general, the data related to 22 studies were evaluated [10] (Fig 1).

We analyzed 22 studies to investigate the relationship between GD and CVDs. These studies were conducted between 1985 and 2023 across multiple countries, including Taiwan, South Korea, China, the US, Denmark, the UK, Germany, Mexico, and Spain. The combined number of participants across these studies was 7,496,303 (Tables 1–3).

**Table 1:** Characteristics of the studies included in the meta-analysis of the relationship between GD and CVDs.

First author	Year	Country	Study design	Study duration	Sample size	GD	Without GD	Average age (Year)	NOS score
Chen HY	2023	Taiwan	cohort	2001–2009	5459	407	5052	52.9	6
Chae W	2023	Korean	cohort	2002–2013	64370	13069	51301	55	6
Bai R	2023	China	cohort	2016–2021	11444	1178	10266	36.56	7
Park SM	2022	Korean	cohort	2005–2017	5403937	491267	4912670	53	6
Ho TC	2021	Taiwan	cohort	2001–2011	11796	5992	5804	57	5
Gill ES	2021	Korea	cross-sectional	2008–2010	566	90	476	66	5
Chen CH	2021	Taiwan	cohort	2000–2010	686600	343300	343300	60	8
Shabanzadeh DM	2017	Denmark	cohort	1982–2015	5496	504	4992	41.8	7
Kwon CH	2017	South Korea	cross-sectional	2011–2014	38778	1426	37352	41.1	6
Zheng Y	2016	USA	cohort	1980–2010	112520	8796	103724	46.7	8
Zheng Y	2016	USA	cohort	1989–2011	112919	5227	107692	34.9	8
Zheng Y	2016	USA	cohort	1986–2010	43703	1449	42254	53.8	8
Wirth J	2015	Germany	cohort	1994–2006	46486	4828	41658	50.5	7
Lv J	2015	China	cohort	2004–2013	487373	28345	459028	51	9
Wei CY	2014	Taiwan	cohort	2000–2003	406536	135512	271024	55	8
Olaiya MT	2013	Taiwan	cohort	2004–2005	34905	6981	27924	45	7
Jiang ZY	2013	China	cross-sectional	2007–2011	1270	206	1064	64.5	6
Khan HN	2009	UK	cohort	1988–1998	4420	2210	2210	78	6
Méndez-Sánchez N	2008	Mexico	cross-sectional	2004–2006	191	62	129	47	5
González-Pérez A	2007	Spain	case control	1996	12353	2353	10000	55	5
Méndez-Sánchez N	2005	Mexico	cross-sectional	2003	473	119	354	47	4
Bortnichak EA	1985	USA	cohort	1948–1974	4708	502	4206	–	6

**Table 2:** The effect size of the relationship between GD and CVDs in included studies.

First author	Year	^*^Name of data extractors	Date of data extraction	The study was eligible to be included in the review?	Study cohort	Follow up (Year)	Women %	CVDs
Chen HY	2023	RH, FA, KA	July-2024	Yes	Male aged under 40 years old and over 79 years old who had a history of stroke or ischemic heart disease	5–11	0	2.10 (1.49–2.96)
Chae W	2023	RH, FA, KA	July-2024	Yes	All Korean citizens’ claims data are included in the NHIS database	4.6	52	1.30 (1.15–1.47)
Bai R	2023	RH, FA, KA	July-2024	Yes	Uyghur adults in Xinjiang	4.92	60	2.89 (2.54–3.29)
Park SM	2022	RH, FA, KA	July-2024	Yes	All patients with gallstones from the KNHI data	7.5	48	1.20 (1.12–1.29)
Ho TC	2021	RH, FA, KA	July-2024	Yes	Patients with GD	6	51	1.2 (1.16–1.25)
Gill ES	2021	RH, FA, KA	July-2024	Yes	Patients aged 40–89 years who had undergone abdominal ultrasound or abdominal computed tomography	2	28.4	2.01 (1.15–3.53)
Chen CH	2021	RH, FA, KA	July-2024	Yes	We enrolled the patients aged ≥ 20 years who had a new diagnosis of GD	6.5	50.5	1.11 (1.09–1.13)
Shabanzadeh DM	2017	RH, FA, KA	July-2024	Yes	General population	32	49.5	1.36 [1.17; 1.59]
Kwon CH	2017	RH, FA, KA	July-2024	Yes	Individuals who participated in a comprehensive health-screening program		22	0.86 (0.73–1.02)
Zheng Y	2016	RH, FA, KA	July-2024	Yes	The Nurses’ Health Study -NHS	8	100	1.15 (1.10–1.19)
Zheng Y	2016	RH, FA, KA	July-2024	Yes	The Nurses’ Health Study -NHS II	8	100	1.27 (1.14–1.42)
Zheng Y	2016	RH, FA, KA	July-2024	Yes	Health Professionals Follow-up Study -HPFS	8	0	1.10 (1.04–1.16)
Wirth J	2015	RH, FA, KA	July-2024	Yes	Participants from 10 European countries	8	58	1.24 (1.02–1.50)
Lv J	2015	RH, FA, KA	July-2024	Yes	participants aged 30–79 years in the China Kadoorie Biobank study	7.2	60	1.23 (1.17–1.28)
Wei CY	2014	RH, FA, KA	July-2024	Yes	The GD cohort comprised individuals with GD newly diagnosed in 2000–2003	10	50.7	1.29 (1.26–1.315)
Olaiya MT	2013	RH, FA, KA	July-2024	Yes	Population including 6,981 patients with GD was identified from The Taiwan	6	56.2	1.32 (122–1.43)
Jiang ZY	2013	RH, FA, KA	July-2024	Yes	Patients undergoing coronary angiography for the first time at Ruijin Hospital for suspected coronary artery disease	0	41	1.59 (1.10–2.31)
Khan HN	2009	RH, FA, KA	July-2024	Yes	General population	10	56	1.03 (0.91–1.16)
Méndez-Sánchez N	2008	RH, FA, KA	July-2024	Yes	Consecutive asymptomatic subjects who were referred to the checkup	0	42	2.12 (1.04–4.34)
González-Pérez A	2007	RH, FA, KA	July-2024	Yes	General population	0.9	58	1.21 (1.18–1.24)
Méndez-Sánchez N	2005	RH, FA, KA	July-2024	Yes	Consecutive asymptomatic subjects who were referred to the checkup	0	39	2.84 (1.33–6.07)
Bortnichak EA	1985	RH, FA, KA	July-2024	Yes	FRAMINGHAM cohort population	26	57	1.55 (1.22–1.95)

* RH; Refli Hasan, FA; Fatemeh Allahbakhsi, KA; Khadija Allahbakhshi.

**Table 3:** Adjusted variables in included studies in the meta-analysis of the relationship between GD and CVDs.

First author	year	Adjusted variables
Chen HY [14]	2023	BMI, alcohol consumption, and regular exercise
Chae W [15]	2023	Age, sex, and year
Bai R [16]	2023	Sex, age, hypertension, T2DM, overweight, and HDL levels
Park SM [9]	2022	Sex, age, SBP/DBP, Pulse pressure, Fasting plasma glucose, BMI, Smoking, Alcohol drinking, Physical activity.
Ho TC [28]	2021	Age, sex, cardiovascular, and gastrointestinal comorbidity
Gill ES [29]	2021	Age, sex, HTN, AF, OCAD, Smoking history
Chen CH [38]	2021	Age, sex and comorbidities of hypertension, diabetes mellitus, hyperlipidemia, CHD, heart failure, COPD, PAOD, chronic renal disease, stroke, cirrhosis and alcohol-related illness.
Shabanzadeh DM [30]	2017	Age, sex, cohort number, body mass index (kg/m 2), systolic blood pressure [140 mmHg, diastolic blood pressure [90 mmHg, non-high density lipoprotein cholesterol, high density lipoprotein cholesterol, smoking (never, past, current), alcohol consumption (units/week), diet (western, prudent, healthy), physical activity level (sedentary, light, moderate or vigorous), social group (I–V), time-dependent coefficients for age, time-dependent coefficients for high density lipoprotein cholesterol, non-high density lipoprotein cholesterol, diastolic blood pressure, and/or smoking.
Kwon CH [39]	2017	Age, center, year of screening exam, smoking status, alcohol intake, physical activity, education level, BMI, medication for hypertension, medication for diabetes, and medication for hyperlipidemia; HOMA-IR and fatty liver, metabolic syndrome; hs-CRP
Zheng Y [10]	2016	Age, race, family history of MI, marital status, smoking status, body mass index, physical activity, diabetes, hypertension, hypercholesterolemia, regular use of aspirin, daily intake of alcohol, daily intake of the energy-adjusted dietary cholesterol, Healthy Eating Index, and daily energy intake
Wirth J [31]	2015	Sex, age, study center, educational achievement, physical activity, smoking habits, alcohol intake, body mass index, waist circumference, and prevalent high blood pressure and hyperlipidemia
Lv J [32]	2015	Age, sex (for whole cohort only), level of education, marital status, alcohol consumption, smoking status, physical activity, intake frequencies of red meat, fresh fruits, and vegetables, prevalent hypertension, prevalent diabetes, family history of heart attack, menopausal status (for women only). Model 3 additionally included body mass index (BMI).
Wei CY [33]	2014	Age, sex and history of hypertension, diabetes, coronary heart disease, atrial fibrillation, and hyperlipidemia.
Olaiya MT [34]	2013	Age, gender, peripheral vascular disease, chronic obstructive pulmonary disease, diabetes mellitus, hyperlipidemia, alcoholism, chronic liver disease, and anemia
Jiang ZY [12]	2013	Age, gender, BMI, and waist circumference; serum TC, TG, LDL-C, and HDL-C, and fasting glucose concentrations; and history of hypertension, DM, NAFLD and MetS
Khan HN [40]	2009	–
Méndez-Sánchez N [17]	2008	Adult treatment panel; BMI, carotid artery intima–media thickness; HDL, homeostasis model assessment
González-Pérez A [35]	2007	Age, Sex, Hyperlipidemia, Hypertension, Diabetes, Alcohol, BMI, Smoking, health services utilization
Méndez-Sánchez N [36]	2005	Age, gender, and BMI
Bortnichak EA [37]	1985	Sex, diabetes, left ventricular hypertrophy, serum cholesterol, age, length of follow-up, systolic blood pressure, Framingham Relative Weight, cigarette smoking.

### Association between GD and CVDs

In 20 of the 22 studies examined, a statistically significant relationship between gallstones and cardiovascular disease risk was observed [9,10,12,14–17,28–38]. However, two studies did not find a significant relationship [39,40]. Overall, gallstones appear to be associated with an increased risk of developing cardiovascular diseases. To further analyze the effect sizes presented in each study, a general effect size was used in the meta-analysis.

**Fig 2:**
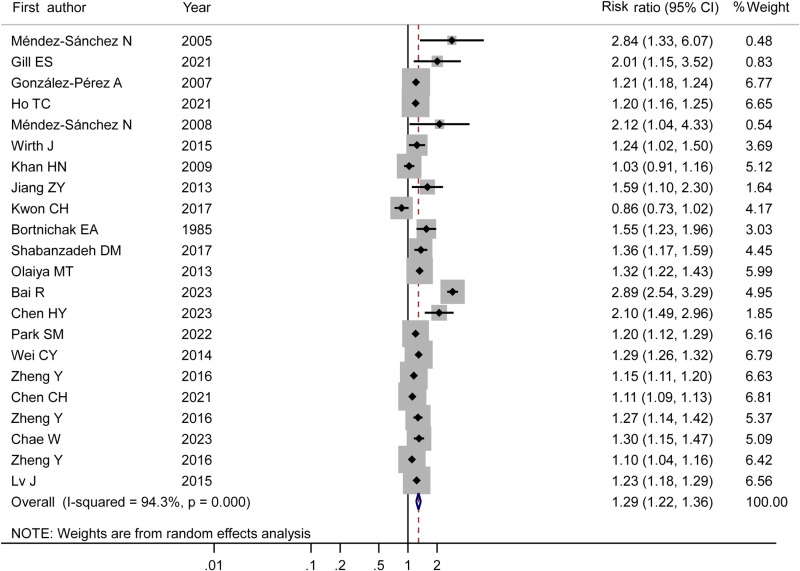
Relationship between GD and CVDs.

The results of meta-analysis demonstrated that individuals with GD had a higher risk of developing CVDs compared to those without GD. The RR for CVDs in the GD population was 1.29 (95% CI: 1.22–1.36; P <  0.001) (Fig 2).

### Assessment of publication bias

**Fig 3:**
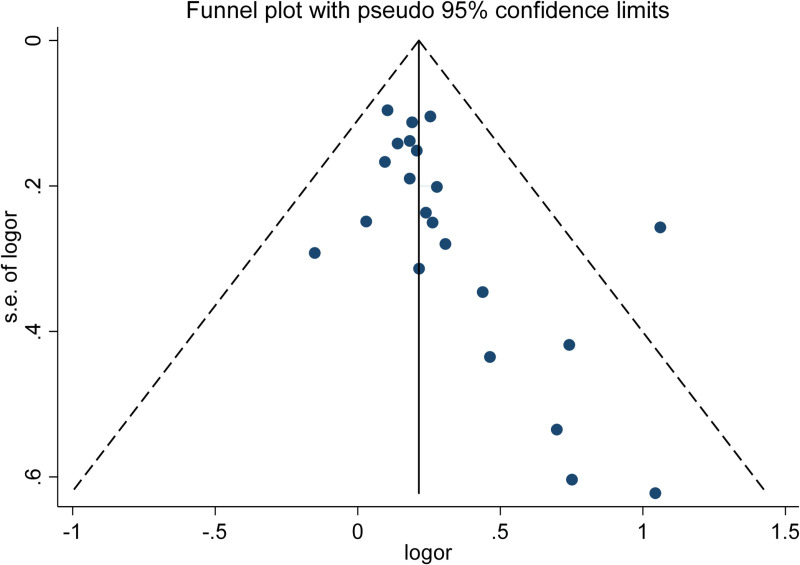
Evaluation of publication bias in meta-analysis studies of the relationship between GD and CVDs.

In assessing publication bias, the Egger’s test (P =  0.103) did not indicate significant bias, whereas the Begg’s test (P =  0.045) suggested the presence of publication bias in the studies (Fig 3).

### Estimation of revised effect size

**Fig 4:**
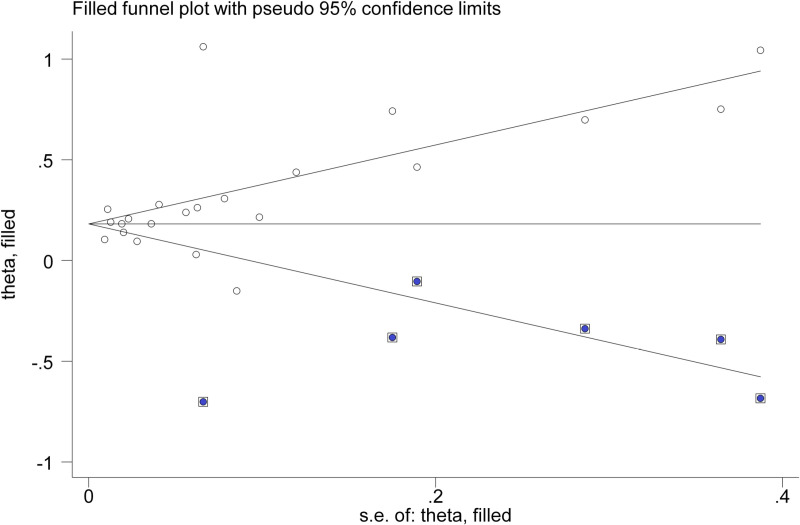
Trim-and-fill method to estimate the effect size of the missing studies of the relationship between GD and CVDs.

Given the Begg’s test results, which indicated potential publication bias, we employed the trim-and-fill method to estimate the effect size of the missing studies and included them in the meta-analysis. This approach estimated the effect size for six missing studies, which were then incorporated into the analysis. After this adjustment, the revised RR for the relationship between GD and CVDs was 1.20 (95% CI: 1.13–1.27; P <  0.001) (Fig 4).

### Meta-regression analysis

In the meta-regression analysis, various variables were assessed, including the year of study, study design, sample size, study quality assessment score, geographical region, average age of subjects, and follow-up duration. None of these variables showed a significant relationship with the observed heterogeneity (P > 0.10) (Table 4).

**Table 4:** Results of meta-regression analysis for the relationship between GD and CVDs.

Meta-regression REML estimate of between-study variance % residual variation due to heterogeneity Proportion of between—study variance explained Joint test for all covariates With Knapp-Hartung modification					Taue2 = 0.07616 I-suuared_res = 93.63% Adj R-squared = -21.82% Model F (7,11) = 0.85 Prob>F = 0.5683	
***Mean***	***Coef.***	***Std. Err.***	***T***	***p>‖t‖***	***[95% Conf. Interval]***	
The year of study	-0.01576	0.1406895	-0.11	0.912	-0.3175091	0.285989
Study design	0.2075697	0.2238072	0.93	0.369	-0.2724489	0.6875884
Sample size	-0.2768924	0.1677387	-1.65	0.121	-0.6366563	0.828714
Quality score	-0.0653552	0.1211389	-0.54	0.598	-0.3251724	0.1944619
Age average	-0.1313607	0.1501278	-0.87	0.396	-0.4533529	0.1906315
Geographical location	0.160672	0.0951486	0.17	0.868	-0.1880063	0.2201407
Follow-up period	-0.174299	0.1997319	-0.87	0.398	-0.6026812	0.2540833
-cons	1.019256	0.487372	2.09	0.055	-0.026053	2.064565

### Sensitivity analysis

**Fig 5:**
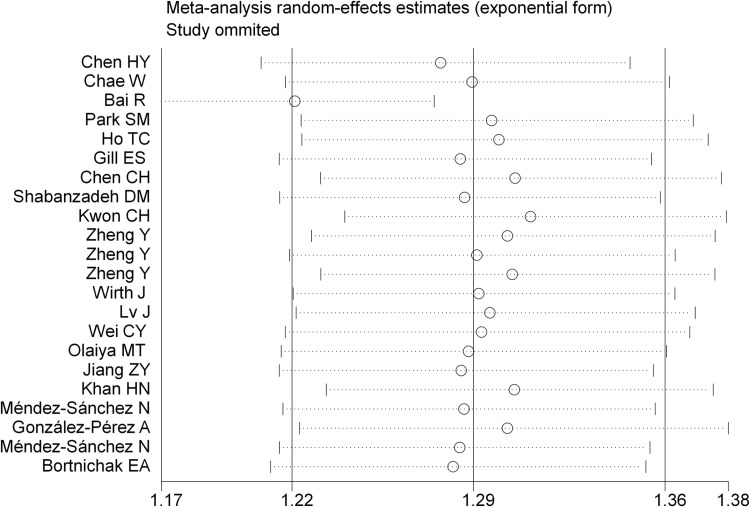
Results of sensitivity analysis for the relationship between GD and CVDs.

A sensitivity analysis, conducted by systematically removing each study, confirmed the robustness of the meta-analysis results, as the estimated RR remained consistent (Fig 5).

### Subgroup analysis

The results of subgroup analysis indicated that the RR of CVDs in individuals with GD was higher in Asian countries, in studies conducted in 2016 or later, in studies with a sample size of less than 10,000 participants, in studies where the average age of participants was under 50 years, in studies with a follow-up period of less than 7 years, in cross-sectional/ case- control studies, in females, and in studies of moderate quality. Nevertheless, the relationship between GD and CVDs remained statistically significant across all subgroups (Table 5).

**Table 5:** Subgroup analysis of the association between GD and CVDs.

*Characteristics*		*Number of studies*	*RR (95% CI)*	*P-value*
Study location	Asian countries	12	1.34 (1.23–1.47)	≤0.001
	Non-Asian countries	10	1.20 (1.13–1.27)	≤0.001
Time period	2015 or earlier	10	1.25 (1.19–1.32)	≤0.001
	2016 or later	12	1.31 (1.19–1.43)	≤0.001
Sample size	<10000	8	1.59 (1.27–1.98)	≤0.001
	≥10000	14	1.25 (1.18–1.33)	≤0.001
Age average	<50	8	1.46 (1.17–1.84)	≤0.001
	≥50	14	1.22 (1.16–1.28)	≤0.001
Follow up time	≤7 years	11	1.37 (1.24–1.51)	≤0.001
	>7 years	11	1.23 (1.16–1.30)	≤0.001
Gender	Female	6	1.35 (1.22–1.48)	≤0.001
	Male	4	1.18 (1.11–1.25)	≤0.001
Study design	Cross-sectional/ Case- control	6	1.40 (1.08–1.811)	0.011
	Cohort	16	1.30 (1.22–1.39)	≤0.001
Quality assessment	Good quality	7	1.20 (1.12–1.28)	≤0.001
	Moderate quality	10	1.41 (1.15–1.74)	0.001
	Low quality	5	1.22 (1.15–1.30)	≤0.001

## Discussion

This study presents a comprehensive analysis of the association between GD and the risk of CVDs. Our findings indicate that GD is significantly correlated with an elevated risk of developing CVDs. Specifically, our analysis demonstrated that individuals with GD have a RR of 1.29 (95% CI: 1.22–1.36; P <  0.001) for developing CVDs compared to those without GD. This result is consistent with previous studies that have identified a similar relationship between GD and cardiovascular risk [9,14–16]. For example, a 2023 study by Chen et al., which included 5,459 individuals aged 40 years and older, found that those with GD exhibited a notably higher RR of 2.10 (95% CI: 1.49–2.96) for CVDs [14]. Similarly, Bai et al. conducted a cohort study involving 11,444 Uyghur adults in Xinjiang, revealing that the risk of CVDs in individuals with GD was nearly threefold higher (RR: 2.89, 95% CI: 2.54–3.29) compared to those without GD [16]. These studies robustly support the notion of an increased cardiovascular risk associated with GD across diverse populations, underscoring the consistency and strength of this association.

Further reinforcing these findings, a meta-analysis by Fairfield et al., which pooled data from eight observational studies, identified a significantly increased risk of both fatal and non-fatal CVD events in individuals with GD (RR: 1.23, 95% CI: 1.16–1.30) [18]. The meta-analysis also conducted subgroup analyses, revealing that the RR of CVDs among women with GD was 1.24 (95% CI: 1.17–1.32), slightly higher than the RR for men (1.18, 95% CI: 1.04–1.33). Additionally, Fairfield et al. noted that individuals with both diabetes and GD exhibited a lower RR (1.13, 95% CI: 1.06–1.20) compared to non-diabetic individuals with GD, whose RR was 1.23 (95% CI: 1.15–1.32). These findings suggest that the GD-CVDs link may be influenced by variables such as gender and comorbid conditions, including diabetes.

Our study also identified variations in CVD risk based on specific study characteristics. For instance, the RR of CVDs in women with GD was 1.35 (95% CI: 1.22–1.48), while in men, the RR was 1.18 (95% CI: 1.11–1.25). These results suggest that although both men and women with GD are at an increased risk of CVDs, the magnitude of this risk may differ slightly between genders.

Further stratified analyses revealed that the RR of CVDs in individuals with GD varied by geographic region, study design, sample size, and follow-up duration. Notably, studies conducted in Asian countries reported a higher RR compared to those carried out in non-Asian countries. Additionally, studies with smaller sample sizes (<10,000 participants) and shorter follow-up periods (<7 years) tended to report higher RRs. Similarly, studies employing cross-sectional or case-control designs, as well as those with lower quality assessment scores, showed a greater RR for the association between GD and CVDs. These findings echo Fan et al.‘s meta-analysis, which included eight primary studies and observed that the RR of CVDs was higher in Asian studies, those with follow-up periods of less than 10 years, and studies with sample sizes below 50,000 participants [41]. Collectively, these observations imply that certain study characteristics—such as geographic location, study design, and sample size—may influence the observed strength of the association between GD and CVDs. However, it is worth noting that in the meta-regression analysis conducted in our study, these variables were not significantly associated with the observed heterogeneity.

GD and CVDs share several important risk factors, both modifiable and non-modifiable. Modifiable risk factors include obesity, diabetes, dyslipidemia, hypertension, chronic inflammation, metabolic syndrome, poor diet, and physical inactivity. These factors contribute to the development of both GD and atherosclerotic CVDs [36,42]. Dietary habits, such as consuming a poor-quality diet high in red meat, refined grains, and added sugars while low in fruits and vegetables, increase the risk of both conditions [42,43]. Physical inactivity, or a sedentary lifestyle, also poses a shared risk for GD and CVDs due to the lack of protective effects of regular exercise [44].

Non-modifiable risk factors include genetic predisposition, familial patterns, and estrogen levels [45,46]. Genetic factors and family history play a role in GD formation and cardiovascular traits [46,47]. Estrogen levels influence the risk of both conditions, with relative protection in pre-menopausal women that decreases after menopause due to declining estrogen levels [45,46]. The significant overlap in the risk factor profiles between GD and CVDs suggests a biological connection. Individuals with GD may be more susceptible to developing atherosclerotic cardiovascular complications if these shared risks are not addressed.

Understanding the common modifiable risk factors for GD and CVDs is paramount for effective prevention and clinical management of both conditions [48–50]. Lifestyle-related factors offer opportunities for reducing risk through behavioral changes at the individual level. Maintaining a healthy body weight, or engaging in modest weight loss if overweight/obese, can significantly lower the risks of GD and cardiovascular events like heart attack and stroke [8,42]. Even a 5–10% reduction in initial body weight through dietary changes and increased physical activity is beneficial [51]. Regular exercise is also crucial, as accumulating at least 150 minutes of moderate activity per week provides cardioprotective effects while also helping to maintain weight loss over the long term [49,51].

Adopting a balanced, nutritious diet is another key strategy. Eating plenty of fruits, vegetables, whole grains, lean proteins, and healthy fats while limiting processed foods, red meat, refined carbohydrates, and excess added sugars can aid weight control and reduce inflammation [44,49,50]. The Mediterranean diet pattern has shown benefits for both GD and CVDs through its emphasis on plant-based foods [52,53]. Addressing modifiable lifestyle-related risks through modest lifestyle changes has been demonstrated in numerous studies to significantly lower disease risk over time [42,53]. Encouraging patients to focus on diet, exercise, and weight management should be an important part of clinical guidance for preventing and managing both GD and cardiovascular conditions.

### Implications of the results for practice, policy, and future research

The findings of our meta-analysis have several important implications. Clinically, they suggest that individuals with GD should be considered at a higher risk for CVDs, emphasizing the need for proactive cardiovascular risk management in this population. For policymakers, these results underline the importance of integrating gallstone screening and management into public health strategies aimed at reducing the burden of CVDs.

In terms of future research, our study highlights the need for more comprehensive and longitudinal studies that can better control for confounding variables and establish causal relationships. Future research should also aim to standardize the definitions and diagnostic criteria for GD and CVDs to minimize heterogeneity. Additionally, there is a need for more studies from diverse geographical regions and populations to ensure the generalizability of the findings. Studies exploring the biological mechanisms linking GD to CVDs would further enhance our understanding and potentially lead to targeted therapeutic interventions.

By addressing these limitations and implications, we can improve the quality and applicability of research in this field, ultimately leading to better health outcomes for individuals at risk of both gallstones and cardiovascular diseases.

## Conclusion

In conclusion, our systematic review and meta-analysis demonstrate that GD is associated with an increased risk of CVDs. It seems that one of the important factors of this relationship is having common causes for the formation of GD and CVDs. However, gallstones can be considered an important sign of increased risk of cardiovascular diseases. Further research is needed to elucidate the mechanisms underlying these associations and to confirm our findings in diverse populations.

## Supporting information

S1 Tables Table 1: Search strategy for included studies. Table 2: The results of the Newcastle-Ottawa Scale (NOS) for assessment of the quality of the observational studies. Table 3: Characteristics of the studies included in the meta-analysis. Table 4: The effect size of the relationship between GD and CVDs in included studies. Table 5: Adjusted variables in included studies in the meta-analysis of the relationship between GD and CVDs. Table 6: Effect size and computational scales (log of effect size and corresponding confidence interval, standard error of effect size, weight assigned to each study (for studies included in the analysis. Table 7: Studies identified in the literature search.
(PDF)

S1 Checklist PRISMA 2020 checklist. (PDF)
