# Supplementary material for: Gallstones as a predictor of elevated cardiovascular disease risk: A meta-analysis and meta-regression of over 7.4 million participants
Source: PLoS One. 2025 Mar 19;20(3):e0314661. doi: 10.1371/journal.pone.0314661 (PMC11922230; doi:10.1371/journal.pone.0314661)
Supplement: S1 Tables — Table 2: The results of the Newcastle-Ottawa Scale (NOS) for assessment of the quality of the observational studies. Table 3: Characteristics of the studies included in the meta-analysis. Table 4: The effect size of the relationship between GD and CVDs in included studies. Table 5: Adjusted variables in included studies in the meta-analysis of the relationship between GD and CVDs. Table 6: Effect size and computational scales (log of effect size and corresponding confidence interval, standard error of effect size, weight assigned to each study (for studies included in the analysis. Table 7: Studies identified in the literature search. (PDF) [file pone.0314661.s001.pdf]

Supporting information

List of tables provided

Tables	*Title*
**Table 1**	**Search strategy for included studies.**
**Table 2**	**The results of the Newcastle-Ottawa Scale (NOS) for assessment of the quality of the observational studies.**
**Table 3**	**Characteristics of the studies included in the meta-analysis.**
**Table 4**	**The effect size of the relationship between GD and CVDs in included studies.**
**Table 5**	**Adjusted variables in included studies in the meta-analysis of the relationship between GD and CVDs.**
**Table 6**	**Effect size and computational scales (log of effect size and corresponding confidence interval, standard error of effect size, weight assigned to each study (for studies included in the analysis.**
**Table 7**	**Studies identified in the literature search.**

Table 1: Search strategy for included studies

MEDLINE via PubMed	("Gallstones"[Title/Abstract] OR "biliary calculi"[Title/Abstract] OR "calculi biliary"[Title/Abstract] OR "gall stone"[Title/Abstract] OR "gall stones"[Title/Abstract] OR "Gallstone"[Title/Abstract] OR "common bile duct calculi"[Title/Abstract] OR (("Gallstones"[MeSH Terms] OR "Gallstones"[All Fields] OR ("Biliary"[All Fields] AND "Calculi"[All Fields]) OR "biliary calculi"[All Fields]) AND "common bile duct"[Title/Abstract]) OR "common bile duct gall stones"[Title/Abstract] OR (("common bile duct"[MeSH Terms] OR ("Common"[All Fields] AND "Bile"[All Fields] AND "Duct"[All Fields]) OR "common bile duct"[All Fields]) AND "gall stone"[Title/Abstract]) OR "common bile duct gallstones"[Title/Abstract] OR "gall stones common bile duct"[Title/Abstract] OR "common bile duct gallstone"[Title/Abstract] OR "gallstones common bile duct"[Title/Abstract]) AND (((((((((((("cardiovascular disease"[Title/Abstract] OR "disease cardiovascular"[Title/Abstract] OR "cardiac events"[Title/Abstract] OR "cardiac event"[Title/Abstract] OR "event cardiac"[Title/Abstract] OR "adverse cardiac event"[Title/Abstract] OR "adverse cardiac events"[Title/Abstract] OR (("cardiacs"[All Fields] OR "Heart"[MeSH Terms] OR "Heart"[All Fields] OR "Cardiac"[All Fields]) AND "event adverse"[Title/Abstract]) OR "cardiac events adverse"[Title/Abstract] OR "major adverse cardiac events"[Title/Abstract] OR "abnormalities cardiovascular"[Title/Abstract] OR "abnormality cardiovascular"[Title/Abstract] OR "cardiovascular abnormality"[Title/Abstract] OR "defect congenital heart"[Title/Abstract] OR "abnormality heart"[Title/Abstract] OR "heart abnormality"[Title/Abstract] OR "congenital heart defect"[Title/Abstract] OR "congenital heart defects"[Title/Abstract] OR "defects congenital heart"[Title/Abstract] OR "heart abnormalities"[Title/Abstract] OR "heart defect congenital"[Title/Abstract] OR "congenital heart disease"[Title/Abstract] OR "congenital heart diseases"[Title/Abstract] OR "disease congenital heart"[Title/Abstract] OR "heart disease congenital"[Title/Abstract] OR "heart malformation of"[Title/Abstract] OR "malformation of heart"[Title/Abstract] OR (("Abnormalities"[MeSH Subheading] OR "Abnormalities"[All Fields] OR "Malformations"[All Fields] OR "congenital abnormalities"[MeSH Terms] OR ("Congenital"[All Fields] AND "Abnormalities"[All Fields]) OR "congenital

	abnormalities"[All Fields] OR "Malformation"[All Fields] OR "malformational"[All Fields] OR "malformative"[All Fields] OR "malformed"[All Fields]) AND "Hearts"[Title/Abstract]) OR "malformations vascular"[Title/Abstract] OR "malformation vascular"[Title/Abstract] OR "vascular malformation"[Title/Abstract] OR "cardiovascular infection"[Title/Abstract] OR "infection cardiovascular"[Title/Abstract] OR "infections cardiovascular"[Title/Abstract] OR "bacterial endocarditides"[Title/Abstract] OR "bacterial endocarditis"[Title/Abstract] OR (("Endocarditis"[MeSH Terms] OR "Endocarditis"[All Fields] OR "Endocarditides"[All Fields]) AND "Bacterial"[Title/Abstract]) OR "cardiovascular syphilis"[Title/Abstract] OR "aortitis syphilitic"[Title/Abstract] OR (("Aortitis"[MeSH Terms] OR "Aortitis"[All Fields] OR "Aortitides"[All Fields]) AND "Syphilitic"[Title/Abstract]) OR (("Syphilitic"[All Fields] OR "syphilitics"[All Fields]) AND "Aortitides"[Title/Abstract]) OR "syphilitic aortitis"[Title/Abstract] OR "heart disease"[Title/Abstract] OR "cardiac disorders"[Title/Abstract] OR "cardiac disorder"[Title/Abstract] OR "heart disorders"[Title/Abstract] OR "heart disorder"[Title/Abstract] OR "cardiac diseases"[Title/Abstract] OR "cardiac disease"[Title/Abstract] OR "arrhythmia cardiac"[Title/Abstract] OR "Arrhythmia"[Title/Abstract] OR "Arrythmia"[Title/Abstract] OR "cardiac arrhythmia"[Title/Abstract] OR "cardiac arrhythmias"[Title/Abstract] OR "cardiac dysrhythmia"[Title/Abstract] OR "dysrhythmia cardiac"[Title/Abstract] OR "carcinoid heart diseases"[Title/Abstract] OR "heart disease carcinoid"[Title/Abstract] OR (("Heart"[MeSH Terms] OR "Heart"[All Fields] OR "Hearts"[All Fields] OR "heart s"[All Fields]) AND "diseases carcinoid"[Title/Abstract]) OR "cardiac conduction system diseases"[Title/Abstract] OR "cardiac conduction defects"[Title/Abstract] OR "conduction defect cardiac"[Title/Abstract] OR (("Abnormalities"[MeSH Subheading] OR "Abnormalities"[All Fields] OR "Defects"[All Fields] OR "Defect"[All Fields] OR "defect s"[All Fields] OR "defected"[All Fields] OR "defective"[All Fields] OR "defectively"[All Fields] OR "defectives"[All Fields]) AND "cardiac conduction"[Title/Abstract]) OR "cardiac conduction defect"[Title/Abstract] OR "post cardiac arrest syndrome"[Title/Abstract] OR "post cardiac arrest syndromes"[Title/Abstract] OR "postcardiac arrest syndrome"[Title/Abstract] OR ("Postcardiac"[All Fields] AND "arrest syndromes"[Title/Abstract]) OR "postresuscitation disease"[Title/Abstract] OR

	(("Postresuscitation"[All Fields] OR "postresuscitative"[All Fields]) AND "Diseases"[Title/Abstract]) OR "postpericardiotomy syndromes"[Title/Abstract] OR "syndrome postpericardiotomy"[Title/Abstract] OR (("syndrom"[All Fields] OR "syndromal"[All Fields] OR "syndromally"[All Fields] OR "Syndrome"[MeSH Terms] OR "Syndrome"[All Fields] OR "Syndromes"[All Fields] OR "syndrome s"[All Fields] OR "syndromic"[All Fields] OR "syndroms"[All Fields]) AND "Postpericardiotomy"[Title/Abstract]) OR "postcommissurotomy syndrome"[Title/Abstract] OR ("Postcommissurotomy"[All Fields] AND "Syndromes"[Title/Abstract]) OR (("syndrom"[All Fields] OR "syndromal"[All Fields] OR "syndromally"[All Fields] OR "Syndrome"[MeSH Terms] OR "Syndrome"[All Fields] OR "Syndromes"[All Fields] OR "syndrome s"[All Fields] OR "syndromic"[All Fields] OR "syndroms"[All Fields]) AND "Postcommissurotomy"[Title/Abstract]) OR (("syndrom"[All Fields] OR "syndromal"[All Fields] OR "syndromally"[All Fields] OR "Syndrome"[MeSH Terms] OR "Syndrome"[All Fields] OR "Syndromes"[All Fields] OR "syndrome s"[All Fields] OR "syndromic"[All Fields] OR "syndroms"[All Fields]) AND "Postcommissurotomy"[Title/Abstract]) OR "cor pulmonale"[Title/Abstract] OR "disease pulmonary heart"[Title/Abstract] OR "diseases pulmonary heart"[Title/Abstract] OR "heart disease pulmonary"[Title/Abstract] OR "heart diseases pulmonary"[Title/Abstract] OR "pulmonary heart diseases"[Title/Abstract] OR "disease rheumatic heart"[Title/Abstract] OR "diseases rheumatic heart"[Title/Abstract] OR "heart disease rheumatic"[Title/Abstract] OR "heart diseases rheumatic"[Title/Abstract] OR "rheumatic heart diseases"[Title/Abstract] OR "bouillaud disease"[Title/Abstract] OR (("Disease"[MeSH Terms] OR "Disease"[All Fields] OR "Diseases"[All Fields] OR "disease s"[All Fields] OR "diseased"[All Fields]) AND "Bouillaud"[Title/Abstract]) OR "bouillaud s disease"[Title/Abstract] OR "bouillauds disease"[Title/Abstract] OR (("Disease"[MeSH Terms] OR "Disease"[All Fields] OR "Diseases"[All Fields] OR "disease s"[All Fields] OR "diseased"[All Fields]) AND "Bouillaud's"[Title/Abstract]) OR (("dysfunctional"[All Fields] OR "dysfunctionals"[All Fields] OR "dysfunctioning"[All Fields] OR "Dysfunctions"[All Fields] OR "physiopathology"[MeSH Subheading] OR "physiopathology"[All Fields] OR "Dysfunction"[All Fields]) AND "Ventricular"[Title/Abstract]) OR "dysfunction ventricular"[Title/Abstract] OR

	"ventricular dysfunctions"[Title/Abstract] OR (("obstruct"[All Fields] OR "obstructed"[All Fields] OR "obstructing"[All Fields] OR "Obstruction"[All Fields] OR "Obstructions"[All Fields] OR "Obstructive"[All Fields] OR "obstructs"[All Fields]) AND "ventricular outflow"[Title/Abstract]) OR "outflow obstruction ventricular"[Title/Abstract] OR "ventricular outflow obstructions"[Title/Abstract] OR "cardiovascular pregnancy complications"[Title/Abstract] OR "cardiovascular pregnancy complication"[Title/Abstract] OR (("complicances"[All Fields] OR "complicate"[All Fields] OR "complicated"[All Fields] OR "complicates"[All Fields] OR "complicating"[All Fields] OR "Complication"[All Fields] OR "complication s"[All Fields] OR "Complications"[MeSH Subheading] OR "Complications"[All Fields]) AND "cardiovascular pregnancy"[Title/Abstract]) OR (("Pregnancy"[MeSH Terms] OR "Pregnancy"[All Fields] OR "pregnancies"[All Fields] OR "pregnancy s"[All Fields]) AND "complication cardiovascular"[Title/Abstract]) OR (("complicances"[All Fields] OR "complicate"[All Fields] OR "complicated"[All Fields] OR "complicates"[All Fields] OR "complicating"[All Fields] OR "Complication"[All Fields] OR "complication s"[All Fields] OR "Complications"[MeSH Subheading] OR "Complications"[All Fields]) AND "cardiovascular pregnancy"[Title/Abstract]) OR "pregnancy cardiovascular complications"[Title/Abstract] OR (("Pregnancy"[MeSH Terms] OR "Pregnancy"[All Fields] OR "pregnancies"[All Fields] OR "pregnancy s"[All Fields]) AND "cardiovascular complications"[Title/Abstract]) OR "amniotic fluid embolisms"[Title/Abstract] OR (("embol"[All Fields] OR "embolics"[All Fields] OR "embolisations"[All Fields] OR "embolise"[All Fields] OR "embolised"[All Fields] OR "embolising"[All Fields] OR "Embolism"[MeSH Terms] OR "Embolism"[All Fields] OR "embolic"[All Fields] OR "Embolisms"[All Fields] OR "embolization, therapeutic"[MeSH Terms] OR ("embolization"[All Fields] AND "therapeutic"[All Fields]) OR "therapeutic embolization"[All Fields] OR "embolisation"[All Fields] OR "embolization"[All Fields] OR "embolizations"[All Fields] OR "embolize"[All Fields] OR "embolized"[All Fields] OR "embolizes"[All Fields] OR "embolizing"[All Fields]) AND "amniotic fluid"[Title/Abstract]) OR "amniotic fluid embolism"[Title/Abstract] OR "diseases vascular"[Title/Abstract] OR "disease vascular"[Title/Abstract] OR "vascular disease"[Title/Abstract] OR "Aneurysms"[Title/Abstract] OR "fusiform

	aneurysm"[Title/Abstract] OR "aneurysm fusiform"[Title/Abstract] OR "aneurysms fusiform"[Title/Abstract] OR "fusiform aneurysms"[Title/Abstract] OR "saccular aneurysm"[Title/Abstract] OR "aneurysms false"[Title/Abstract] OR "false aneurysms"[Title/Abstract] OR "Pseudoaneurysm"[Title/Abstract] OR "Pseudoaneurysms"[Title/Abstract] OR "false aneurysm"[Title/Abstract] OR "Angiodysplasias"[Title/Abstract] OR "Angioedemas"[Title/Abstract] OR (("Urticaria"[MeSH Terms] OR "Urticaria"[All Fields] OR "Urticarias"[All Fields]) AND "Giant"[Title/Abstract]) OR "giant urticaria"[Title/Abstract] OR (("giant s"[All Fields] OR "gigantism"[MeSH Terms] OR "gigantism"[All Fields] OR "Giant"[All Fields] OR "giants"[All Fields]) AND "Urticarias"[Title/Abstract]) OR (("Urticaria"[MeSH Terms] OR "Urticaria"[All Fields] OR "Urticarias"[All Fields]) AND "Giant"[Title/Abstract]) OR "angioneurotic edema"[Title/Abstract] OR "angioneurotic edemas"[Title/Abstract] OR "edema angioneurotic"[Title/Abstract] OR (("Edema"[MeSH Terms] OR "Edema"[All Fields] OR "Edemas"[All Fields] OR "oedemas"[All Fields] OR "oedema"[All Fields]) AND "Angioneurotic"[Title/Abstract]) OR "quincke s edema"[Title/Abstract] OR "edema quincke s"[Title/Abstract] OR "quincke edema"[Title/Abstract] OR "quinckes edema"[Title/Abstract] OR "Angiomatoses"[Title/Abstract] OR "aortic disease"[Title/Abstract] OR "disease aortic"[Title/Abstract] OR "diseases aortic"[Title/Abstract] OR "arterial occlusive disease"[Title/Abstract] OR "disease arterial occlusive"[Title/Abstract] OR "occlusive disease arterial"[Title/Abstract] OR "arterial obstructive diseases"[Title/Abstract] OR "arterial obstructive disease"[Title/Abstract] OR "disease arterial obstructive"[Title/Abstract] OR "obstructive disease arterial"[Title/Abstract] OR "arterial occlusion"[Title/Abstract] OR "arterial occlusions"[Title/Abstract] OR "occlusion arterial"[Title/Abstract] OR "arteriovenous malformation"[Title/Abstract] OR "malformation arteriovenous"[Title/Abstract] OR "malformations arteriovenous"[Title/Abstract] OR "capillary leak syndromes"[Title/Abstract] OR "clarkson disease"[Title/Abstract] OR "systemic capillary leak syndrome"[Title/Abstract] OR "clinical capillary leak syndrome"[Title/Abstract] OR "cerebrovascular disorder"[Title/Abstract] OR "brain vascular disorders"[Title/Abstract] OR "brain vascular disorder"[Title/Abstract] OR (("blood vessels"[MeSH Terms] OR ("Blood"[All Fields] AND "vessels"[All Fields]) OR "blood vessels"[All Fields] OR

	"Vascular"[All Fields] OR "neovascularization, pathologic"[MeSH Terms] OR ("neovascularization"[All Fields] AND "pathologic"[All Fields]) OR "pathologic neovascularization"[All Fields] OR "vascularisation"[All Fields] OR "vascularization"[All Fields] OR "vascularisations"[All Fields] OR "vascularise"[All Fields] OR "vascularised"[All Fields] OR "vascularities"[All Fields] OR "vascularitis"[All Fields] OR "vascularity"[All Fields] OR "vascularizations"[All Fields] OR "vascularize"[All Fields] OR "vascularized"[All Fields] OR "vascularizes"[All Fields] OR "vascularizing"[All Fields] OR "vasculars"[All Fields]) AND "disorder brain"[Title/Abstract]) OR (("blood vessels"[MeSH Terms] OR ("Blood"[All Fields] AND "vessels"[All Fields]) OR "blood vessels"[All Fields] OR "Vascular"[All Fields] OR "neovascularization, pathologic"[MeSH Terms] OR ("neovascularization"[All Fields] AND "pathologic"[All Fields]) OR "pathologic neovascularization"[All Fields] OR "vascularisation"[All Fields] OR "vascularization"[All Fields] OR "vascularisations"[All Fields] OR "vascularise"[All Fields] OR "vascularised"[All Fields] OR "vascularities"[All Fields] OR "vascularitis"[All Fields] OR "vascularity"[All Fields] OR "vascularizations"[All Fields] OR "vascularize"[All Fields] OR "vascularized"[All Fields] OR "vascularizes"[All Fields] OR "vascularizing"[All Fields] OR "vasculars"[All Fields]) AND "disorders brain"[Title/Abstract]) OR "intracranial vascular disorders"[Title/Abstract] OR "intracranial vascular disorder"[Title/Abstract] OR (("vascular diseases"[MeSH Terms] OR ("Vascular"[All Fields] AND "Diseases"[All Fields]) OR "vascular diseases"[All Fields] OR ("Vascular"[All Fields] AND "Disorder"[All Fields]) OR "vascular disorder"[All Fields]) AND "Intracranial"[Title/Abstract]) OR "vascular disorders intracranial"[Title/Abstract] OR "vascular diseases intracranial"[Title/Abstract] OR "intracranial vascular disease"[Title/Abstract] OR "intracranial vascular diseases"[Title/Abstract] OR "vascular disease intracranial"[Title/Abstract] OR "cerebrovascular diseases"[Title/Abstract] OR "cerebrovascular disease"[Title/Abstract] OR "disease cerebrovascular"[Title/Abstract] OR "diseases cerebrovascular"[Title/Abstract] OR "cerebrovascular insufficiency"[Title/Abstract] OR "cerebrovascular insufficiencies"[Title/Abstract] OR (("Insufficiencies"[All Fields] OR "Insufficiency"[All Fields] OR "insufficient"[All Fields] OR "insufficiently"[All Fields]) AND "Cerebrovascular"[Title/Abstract]) OR "insufficiency

	cerebrovascular"[Title/Abstract] OR "cerebrovascular occlusion"[Title/Abstract] OR "cerebrovascular occlusions"[Title/Abstract] OR "occlusion cerebrovascular"[Title/Abstract] OR (("dental occlusion"[MeSH Terms] OR ("dental"[All Fields] AND "Occlusion"[All Fields]) OR "dental occlusion"[All Fields] OR "Occlusion"[All Fields] OR "occlused"[All Fields] OR "Occlusions"[All Fields] OR "Occlusive"[All Fields] OR "occlusives"[All Fields]) AND "Cerebrovascular"[Title/Abstract]) OR "ischemic colitis"[Title/Abstract] OR "compartment syndrome"[Title/Abstract] OR (("Angiopathies"[All Fields] OR "vascular diseases"[MeSH Terms] OR ("Vascular"[All Fields] AND "Diseases"[All Fields]) OR "vascular diseases"[All Fields] OR "Angiopathy"[All Fields]) AND "Diabetic"[Title/Abstract]) OR "angiopathy diabetic"[Title/Abstract] OR "diabetic angiopathy"[Title/Abstract] OR "diabetic vascular complications"[Title/Abstract] OR "diabetic vascular complication"[Title/Abstract] OR "vascular complication diabetic"[Title/Abstract] OR "vascular complications diabetic"[Title/Abstract] OR "diabetic vascular diseases"[Title/Abstract] OR "diabetic vascular disease"[Title/Abstract] OR "vascular disease diabetic"[Title/Abstract] OR "vascular diseases diabetic"[Title/Abstract] OR "microangiopathy diabetic"[Title/Abstract] OR "diabetic microangiopathies"[Title/Abstract] OR "diabetic microangiopathy"[Title/Abstract] OR "microangiopathies diabetic"[Title/Abstract] OR "Thrombosis"[Title/Abstract]) AND "Embolism"[Title/Abstract]) OR "hand arm vibration syndrome"[Title/Abstract] OR "hand arm vibration syndromes"[Title/Abstract] OR "syndrome hand arm vibration"[Title/Abstract] OR (("syndrom"[All Fields] OR "syndromal"[All Fields] OR "syndromally"[All Fields] OR "Syndrome"[MeSH Terms] OR "Syndrome"[All Fields] OR "Syndromes"[All Fields] OR "syndrome s"[All Fields] OR "syndromic"[All Fields] OR "syndroms"[All Fields]) AND "hand arm vibration"[Title/Abstract]) OR (("vibrate"[All Fields] OR "vibrated"[All Fields] OR "vibrates"[All Fields] OR "vibrating"[All Fields] OR "Vibration"[MeSH Terms] OR "Vibration"[All Fields] OR "vibrations"[All Fields] OR "vibrational"[All Fields] OR "vibrator"[All Fields] OR "vibrators"[All Fields]) AND "syndrome hand arm"[Title/Abstract]) OR ((("vibrate"[All Fields] OR "vibrated"[All Fields] OR "vibrates"[All Fields] OR "vibrating"[All Fields] OR "Vibration"[MeSH Terms] OR "Vibration"[All Fields] OR "vibrations"[All Fields] OR "vibrational"[All Fields] OR

	"vibrator"[All Fields] OR "vibrators"[All Fields]) AND ("syndrom"[All Fields] OR "syndromal"[All Fields] OR "syndromally"[All Fields] OR "Syndrome"[MeSH Terms] OR "Syndrome"[All Fields] OR "Syndromes"[All Fields] OR "syndrome s"[All Fields] OR "syndromic"[All Fields] OR "syndroms"[All Fields])) AND "Hand-Arm"[Title/Abstract]) OR "effusion pericardial"[Title/Abstract] OR "effusions pericardial"[Title/Abstract] OR "pericardial effusions"[Title/Abstract] OR "Hemopericardium"[Title/Abstract] OR "Chylopericardium"[Title/Abstract] OR "Chylopericardiums"[Title/Abstract] OR "Pericarditis"[Title/Abstract] OR "Pleuropericarditis"[Title/Abstract] OR "heart ruptures"[Title/Abstract] OR "cardiac rupture"[Title/Abstract] OR "cardiac ruptures"[Title/Abstract] OR (("Free"[All Fields] AND "Wall"[All Fields]) AND "rupture heart"[Title/Abstract]) OR "cardiac free wall rupture"[Title/Abstract] OR "ventricular free wall rupture"[Title/Abstract] OR "heart valve disease"[Title/Abstract] OR "valve disease heart"[Title/Abstract] OR "valvular heart diseases"[Title/Abstract] OR "heart disease valvular"[Title/Abstract] OR "valvular heart disease"[Title/Abstract] OR "heart valvular disease"[Title/Abstract] OR (("Disease"[MeSH Terms] OR "Disease"[All Fields] OR "Diseases"[All Fields] OR "disease s"[All Fields] OR "diseased"[All Fields]) AND "heart valvular"[Title/Abstract]) OR "heart valvular diseases"[Title/Abstract] OR "valvular disease heart"[Title/Abstract] OR "heart disease ischemic"[Title/Abstract] OR "disease ischemic heart"[Title/Abstract] OR "diseases ischemic heart"[Title/Abstract] OR "heart diseases ischemic"[Title/Abstract] OR "ischemic heart diseases"[Title/Abstract] OR "ischemia myocardial"[Title/Abstract] OR "ischemias myocardial"[Title/Abstract] OR "myocardial ischemias"[Title/Abstract] OR (("ischaemic heart disease"[All Fields] OR "myocardial ischemia"[MeSH Terms] OR ("Myocardial"[All Fields] AND "Ischemia"[All Fields]) OR "myocardial ischemia"[All Fields] OR ("Ischemic"[All Fields] AND "Heart"[All Fields] AND "Disease"[All Fields]) OR "ischemic heart disease"[All Fields] OR "coronary artery disease"[MeSH Terms] OR ("coronary"[All Fields] AND "Artery"[All Fields] AND "Disease"[All Fields]) OR "coronary artery disease"[All Fields]) AND "stunning myocardial"[Title/Abstract]) OR "stunned myocardium"[Title/Abstract] OR "myocardium stunned"[Title/Abstract] OR "hibernation myocardial"[Title/Abstract] OR "myocardial hibernation"[Title/Abstract] OR "disease hepatic veno occlusive"[Title/Abstract] OR "hepatic veno occlusive diseases"[Title/Abstract] OR

	"hepatic veno occlusive disease"[Title/Abstract] OR "veno occlusive disease hepatic"[Title/Abstract] OR "veno occlusive disease hepatic"[Title/Abstract] OR "sinusoidal obstruction syndrome"[Title/Abstract] OR "syndrome sinusoidal obstruction"[Title/Abstract] OR "blood pressure high"[Title/Abstract] OR "blood pressures high"[Title/Abstract] OR "high blood pressure"[Title/Abstract] OR "high blood pressures"[Title/Abstract] OR "blood pressure low"[Title/Abstract] OR "hypotension vascular"[Title/Abstract] OR "low blood pressure"[Title/Abstract] OR "vascular hypotension"[Title/Abstract] OR "ischemia mesenteric"[Title/Abstract] OR (("ischaemia"[All Fields] OR "Ischemia"[MeSH Terms] OR "Ischemia"[All Fields] OR "ischaemias"[All Fields] OR "Ischemias"[All Fields]) AND "Mesenteric"[Title/Abstract]) OR "mesenteric ischemias"[Title/Abstract] OR "mesenteric vascular insufficiency"[Title/Abstract] OR (("Insufficiencies"[All Fields] OR "Insufficiency"[All Fields] OR "insufficient"[All Fields] OR "insufficiently"[All Fields]) AND "mesenteric vascular"[Title/Abstract]) OR (("Insufficiencies"[All Fields] OR "Insufficiency"[All Fields] OR "insufficient"[All Fields] OR "insufficiently"[All Fields]) AND "mesenteric vascular"[Title/Abstract]) OR (("mesenterical"[All Fields] OR "mesenteritis"[All Fields] OR "mesentery"[MeSH Terms] OR "mesentery"[All Fields] OR "Mesenteric"[All Fields]) AND "vascular insufficiencies"[Title/Abstract]) OR ((("blood vessels"[MeSH Terms] OR ("Blood"[All Fields] AND "vessels"[All Fields]) OR "blood vessels"[All Fields] OR "Vascular"[All Fields] OR "neovascularization, pathologic"[MeSH Terms] OR ("neovascularization"[All Fields] AND "pathologic"[All Fields]) OR "pathologic neovascularization"[All Fields] OR "vascularisation"[All Fields] OR "vascularization"[All Fields] OR "vascularisations"[All Fields] OR "vascularise"[All Fields] OR "vascularised"[All Fields] OR "vascularities"[All Fields] OR "vascularitis"[All Fields] OR "vascularity"[All Fields] OR "vascularizations"[All Fields] OR "vascularize"[All Fields] OR "vascularized"[All Fields] OR "vascularizes"[All Fields] OR "vascularizing"[All Fields] OR "vasculars"[All Fields]) AND ("Insufficiencies"[All Fields] OR "Insufficiency"[All Fields] OR "insufficient"[All Fields] OR "insufficiently"[All Fields])) AND "Mesenteric"[Title/Abstract]) OR "vascular insufficiency mesenteric"[Title/Abstract] OR "occlusive mesenteric arterial ischemia"[Title/Abstract] OR "acute mesenteric arterial embolus"[Title/Abstract] OR "nonocclusive mesenteric ischemia"[Title/Abstract] OR

	"ischemia nonocclusive mesenteric"[Title/Abstract] OR (("ischaemia"[All Fields] OR "Ischemia"[MeSH Terms] OR "Ischemia"[All Fields] OR "ischaemias"[All Fields] OR "Ischemias"[All Fields]) AND "nonocclusive mesenteric"[Title/Abstract]) OR "mesenteric ischemia nonocclusive"[Title/Abstract] OR (("mesenteric ischemia"[MeSH Terms] OR ("Mesenteric"[All Fields] AND "Ischemia"[All Fields]) OR "mesenteric ischemia"[All Fields] OR ("Mesenteric"[All Fields] AND "Ischemias"[All Fields]) OR "mesenteric ischemias"[All Fields]) AND "Nonocclusive"[Title/Abstract]) OR ("Nonocclusive"[All Fields] AND "mesenteric ischemias"[Title/Abstract]) OR "acute mesenteric arterial thrombosis"[Title/Abstract] OR "mesenteric venous thrombosis"[Title/Abstract] OR "mesenteric venous thromboses"[Title/Abstract] OR (("thrombose"[All Fields] OR "thrombosing"[All Fields] OR "Thrombosis"[MeSH Terms] OR "Thrombosis"[All Fields] OR "thrombosed"[All Fields] OR "Thromboses"[All Fields]) AND "mesenteric venous"[Title/Abstract]) OR "thrombosis mesenteric venous"[Title/Abstract] OR (("venous thrombosis"[MeSH Terms] OR ("Venous"[All Fields] AND "Thrombosis"[All Fields]) OR "venous thrombosis"[All Fields] OR ("Venous"[All Fields] AND "Thromboses"[All Fields]) OR "venous thromboses"[All Fields]) AND "Mesenteric"[Title/Abstract]) OR "venous thrombosis mesenteric"[Title/Abstract] OR "heart disease ischemic"[Title/Abstract] OR "disease ischemic heart"[Title/Abstract] OR "diseases ischemic heart"[Title/Abstract] OR "heart diseases ischemic"[Title/Abstract] OR "ischemic heart diseases"[Title/Abstract] OR "ischemia myocardial"[Title/Abstract] OR "ischemias myocardial"[Title/Abstract] OR "myocardial ischemias"[Title/Abstract] OR "ischemic heart disease"[Title/Abstract] OR "myocardial ischemia"[Title/Abstract] OR "optic ischaemic neuropathy"[Title/Abstract] OR (("ischaemics"[All Fields] OR "Ischemia"[MeSH Terms] OR "Ischemia"[All Fields] OR "Ischaemic"[All Fields] OR "Ischemic"[All Fields] OR "ischemical"[All Fields] OR "ischemically"[All Fields] OR "ischemics"[All Fields] OR "ischemized"[All Fields]) AND "neuropathy optic"[Title/Abstract]) OR (("Neuropathies"[All Fields] OR "Neuropathy"[All Fields]) AND "optic ischaemic"[Title/Abstract]) OR ((("eye"[MeSH Terms] OR "eye"[All Fields] OR "Optic"[All Fields] OR "optic s"[All Fields] OR "optical"[All Fields] OR "optically"[All Fields] OR "optics"[All Fields]) AND ("ischaemics"[All Fields] OR "Ischemia"[MeSH Terms] OR "Ischemia"[All Fields] OR "Ischaemic"[All Fields] OR

	"Ischemic"[All Fields] OR "ischemical"[All Fields] OR "ischemically"[All Fields] OR "ischemics"[All Fields] OR "ischemized"[All Fields])) AND "Neuropathies"[Title/Abstract]) OR "optic ischemic neuropathy"[Title/Abstract] OR "ischemic neuropathy optic"[Title/Abstract] OR (("Neuropathies"[All Fields] OR "Neuropathy"[All Fields]) AND "optic ischemic"[Title/Abstract]) OR (("eye"[MeSH Terms] OR "eye"[All Fields] OR "Optic"[All Fields] OR "optic s"[All Fields] OR "optical"[All Fields] OR "optically"[All Fields] OR "optics"[All Fields]) AND "ischemic neuropathies"[Title/Abstract]) OR "ischemic optic neuropathy"[Title/Abstract] OR "ischemic optic neuropathies"[Title/Abstract] OR "neuropathy ischemic optic"[Title/Abstract] OR "optic nerve ischemia"[Title/Abstract] OR "ischemia optic nerve"[Title/Abstract] OR "nerve ischemia optic"[Title/Abstract] OR "optic nerve ischemias"[Title/Abstract] OR "anterior ischemic optic neuropathy"[Title/Abstract] OR "optic neuropathy anterior ischemic"[Title/Abstract] OR "posterior ischemic optic neuropathy"[Title/Abstract] OR "optic neuropathy posterior ischemic"[Title/Abstract] OR "nonarteritic anterior ischemic optic neuropathy"[Title/Abstract] OR ("Hepatis"[All Fields] AND "Peliosis"[Title/Abstract]) OR "atherosclerotic ulcer penetrating"[Title/Abstract] OR "penetrating atherosclerotic ulcers"[Title/Abstract] OR (("Ulcer"[MeSH Terms] OR "Ulcer"[All Fields] OR "ulcerate"[All Fields] OR "ulcerated"[All Fields] OR "ulcerates"[All Fields] OR "ulcerating"[All Fields] OR "ulceration"[All Fields] OR "ulcerations"[All Fields] OR "ulcerative"[All Fields] OR "Ulcers"[All Fields] OR "ulcer s"[All Fields] OR "ulcerous"[All Fields]) AND "penetrating atherosclerotic"[Title/Abstract]) OR "penetrating aortic ulcer"[Title/Abstract] OR (("Aorta"[MeSH Terms] OR "Aorta"[All Fields] OR "Aortic"[All Fields] OR "aortics"[All Fields]) AND "ulcer penetrating"[Title/Abstract]) OR "penetrating aortic ulcers"[Title/Abstract] OR (("Ulcer"[MeSH Terms] OR "Ulcer"[All Fields] OR "ulcerate"[All Fields] OR "ulcerated"[All Fields] OR "ulcerates"[All Fields] OR "ulcerating"[All Fields] OR "ulceration"[All Fields] OR "ulcerations"[All Fields] OR "ulcerative"[All Fields] OR "Ulcers"[All Fields] OR "ulcer s"[All Fields] OR "ulcerous"[All Fields]) AND "penetrating aortic"[Title/Abstract]) OR (("penetrating atherosclerotic ulcer"[MeSH Terms] OR ("Penetrating"[All Fields] AND "Atherosclerotic"[All Fields] AND "Ulcer"[All Fields]) OR "penetrating atherosclerotic ulcer"[All Fields] OR

	("Penetrating"[All Fields] AND "Ulcer"[All Fields]) OR "penetrating ulcer"[All Fields]) AND "Aorta"[Title/Abstract]) OR (("penetrating atherosclerotic ulcer"[MeSH Terms] OR ("Penetrating"[All Fields] AND "Atherosclerotic"[All Fields] AND "Ulcer"[All Fields]) OR "penetrating atherosclerotic ulcer"[All Fields] OR ("Penetrating"[All Fields] AND "Ulcer"[All Fields]) OR "penetrating ulcer"[All Fields]) AND "Aortas"[Title/Abstract]) OR "aortic penetrating ulcer"[Title/Abstract] OR "aortic penetrating ulcers"[Title/Abstract] OR (("penetrability"[All Fields] OR "penetrable"[All Fields] OR "penetrate"[All Fields] OR "penetrated"[All Fields] OR "penetrates"[All Fields] OR "Penetrating"[All Fields] OR "penetration"[All Fields] OR "penetrations"[All Fields]) AND "ulcer aortic"[Title/Abstract]) OR (("Ulcer"[MeSH Terms] OR "Ulcer"[All Fields] OR "ulcerate"[All Fields] OR "ulcerated"[All Fields] OR "ulcerates"[All Fields] OR "ulcerating"[All Fields] OR "ulceration"[All Fields] OR "ulcerations"[All Fields] OR "ulcerative"[All Fields] OR "Ulcers"[All Fields] OR "ulcer s"[All Fields] OR "ulcerous"[All Fields]) AND "aortic penetrating"[Title/Abstract]) OR "penetrating ulcer"[Title/Abstract] OR "penetrating ulcers"[Title/Abstract] OR "ulcer penetrating"[Title/Abstract] OR "disease peripheral vascular"[Title/Abstract] OR "peripheral vascular disease"[Title/Abstract] OR "vascular disease peripheral"[Title/Abstract] OR "diseases peripheral vascular"[Title/Abstract] OR "peripheral angiopathies"[Title/Abstract] OR (("Angiopathies"[All Fields] OR "vascular diseases"[MeSH Terms] OR ("Vascular"[All Fields] AND "Diseases"[All Fields]) OR "vascular diseases"[All Fields] OR "Angiopathy"[All Fields]) AND "Peripheral"[Title/Abstract]) OR "angiopathy peripheral"[Title/Abstract] OR "peripheral angiopathy"[Title/Abstract] OR "vascular diseases peripheral"[Title/Abstract] OR "Pre-Hypertension"[Title/Abstract] OR "Pre-Hypertension"[Title/Abstract] OR (("Disease"[MeSH Terms] OR "Disease"[All Fields] OR "Diseases"[All Fields] OR "disease s"[All Fields] OR "diseased"[All Fields]) AND "pulmonary veno occlusive"[Title/Abstract]) OR "pulmonary veno occlusive diseases"[Title/Abstract] OR ("Veno-Occlusive"[All Fields] AND "diseases pulmonary"[Title/Abstract]) OR "pulmonary veno occlusive disease"[Title/Abstract] OR "veno occlusive disease pulmonary"[Title/Abstract] OR "veno occlusive disease pulmonary"[Title/Abstract] OR "pulmonary venoocclusive disease"[Title/Abstract] OR "pulmonary venoocclusive diseases"[Title/Abstract] OR "venoocclusive disease

	pulmonary"[Title/Abstract] OR ("Venoocclusive"[All Fields] AND "diseases pulmonary"[Title/Abstract]) OR "reperfusion injuries"[Title/Abstract] OR "injury ischemia reperfusion"[Title/Abstract] OR "injury ischemia reperfusion"[Title/Abstract] OR "ischemia reperfusion injuries"[Title/Abstract] OR "injury reperfusion"[Title/Abstract] OR "ischemia reperfusion injury"[Title/Abstract] OR "ischemia reperfusion injury"[Title/Abstract] OR "reperfusion damage"[Title/Abstract] OR "damage reperfusion"[Title/Abstract] OR "reperfusion damages"[Title/Abstract] OR "occlusion retinal vein"[Title/Abstract] OR "retinal vein occlusions"[Title/Abstract] OR "vein occlusion retinal"[Title/Abstract] OR "retinal vein thrombosis"[Title/Abstract] OR "retinal vein thromboses"[Title/Abstract] OR "vein thrombosis retinal"[Title/Abstract] OR "thrombosis retinal vein"[Title/Abstract] OR "branch vein occlusion"[Title/Abstract] OR "branch vein occlusions"[Title/Abstract] OR "occlusion branch vein"[Title/Abstract] OR "vein occlusion branch"[Title/Abstract] OR "branch retinal vein occlusion"[Title/Abstract] OR "retinal branch vein occlusion"[Title/Abstract] OR "central retinal vein occlusion"[Title/Abstract] OR "scimitar anomaly"[Title/Abstract] OR "anomaly scimitar"[Title/Abstract] OR "total anomalous pulmonary venous return"[Title/Abstract] OR "total anomalous pulmonary venous return"[Title/Abstract] OR "anomalous pulmonary venous return"[Title/Abstract] OR "pulmonary venous return anomaly"[Title/Abstract] OR "vascular diseases spinal cord"[Title/Abstract] OR "Hematomyelia"[Title/Abstract] OR "posterior spinal artery syndrome"[Title/Abstract] OR "infarction splenic"[Title/Abstract] OR "infarctions splenic"[Title/Abstract] OR "splenic infarctions"[Title/Abstract] OR "splenic infarct"[Title/Abstract] OR "infarct splenic"[Title/Abstract] OR "infarcts splenic"[Title/Abstract] OR "splenic infarcts"[Title/Abstract] OR "infarct of the spleen"[Title/Abstract] OR "pulmonary vein stenoses"[Title/Abstract] OR "pulmonary vein stenosis"[Title/Abstract] OR (("constriction, pathologic"[MeSH Terms] OR ("constriction"[All Fields] AND "pathologic"[All Fields]) OR "pathologic constriction"[All Fields] OR "stenose"[All Fields] OR "Stenoses"[All Fields] OR "stenosed"[All Fields]) AND "pulmonary vein"[Title/Abstract]) OR (("Veins"[MeSH Terms] OR "Veins"[All Fields] OR "Vein"[All Fields]) AND "stenoses pulmonary"[Title/Abstract]) OR "vein stenosis pulmonary"[Title/Abstract] OR "svc syndrome"[Title/Abstract] OR "svc syndromes"[Title/Abstract] OR "syndrome svc"[Title/Abstract] OR "obstruction of the

	superior vena cava"[Title/Abstract] OR "svc obstruction"[Title/Abstract] OR "obstruction svc"[Title/Abstract] OR "svc obstructions"[Title/Abstract] OR "superior vena cava obstruction"[Title/Abstract] OR "superior vena cava thrombosis"[Title/Abstract] OR "svc thrombosis"[Title/Abstract] OR ("SVC"[All Fields] AND "Thromboses"[Title/Abstract]) OR "thrombosis svc"[Title/Abstract] OR "Telangiectases"[Title/Abstract] OR "Telangiectasia"[Title/Abstract] OR "Telangiectasias"[Title/Abstract] OR "spider veins"[Title/Abstract] OR "spider vein"[Title/Abstract] OR (("Veins"[MeSH Terms] OR "Veins"[All Fields] OR "Vein"[All Fields]) AND "Spider"[Title/Abstract]) OR "veins spider"[Title/Abstract] OR (("Outlet"[All Fields] OR "outlets"[All Fields]) AND "syndromes thoracic"[Title/Abstract]) OR "outlet syndrome thoracic"[Title/Abstract] OR "syndromes thoracic outlet"[Title/Abstract] OR "syndrome thoracic outlet"[Title/Abstract] OR "thoracic outlet syndromes"[Title/Abstract] OR (("Aperture"[All Fields] OR "aperture s"[All Fields] OR "apertured"[All Fields] OR "apertures"[All Fields] OR "aperturing"[All Fields]) AND "syndrome thoracic outlet"[Title/Abstract]) OR ("Neurovascular"[All Fields] AND "syndrome thoracic outlet"[Title/Abstract]) OR "superior thoracic aperture syndrome"[Title/Abstract] OR ((("thoracal"[All Fields] OR "thoracical"[All Fields] OR "thorax"[MeSH Terms] OR "thorax"[All Fields] OR "Thoracic"[All Fields] OR "thoracics"[All Fields]) AND ("Outlet"[All Fields] OR "outlets"[All Fields])) AND "neurovascular syndrome"[Title/Abstract]) OR "scalenus anticus syndrome"[Title/Abstract] OR "syndrome scalenus anticus"[Title/Abstract] OR "costoclavicular syndrome"[Title/Abstract] OR "costoclavicular syndromes"[Title/Abstract] OR "syndrome costoclavicular"[Title/Abstract] OR (("syndrom"[All Fields] OR "syndromal"[All Fields] OR "syndromally"[All Fields] OR "Syndrome"[MeSH Terms] OR "Syndrome"[All Fields] OR "Syndromes"[All Fields] OR "syndrome s"[All Fields] OR "syndromic"[All Fields] OR "syndroms"[All Fields]) AND "Costoclavicular"[Title/Abstract]) OR ((("thoracal"[All Fields] OR "thoracical"[All Fields] OR "thorax"[MeSH Terms] OR "thorax"[All Fields] OR "Thoracic"[All Fields] OR "thoracics"[All Fields]) AND ("Outlet"[All Fields] OR "outlets"[All Fields])) AND "nerve compression syndrome"[Title/Abstract]) OR ((("Nerve"[All Fields] OR "nerve s"[All Fields] OR "nerved"[All Fields] OR "nerves"[All Fields]) AND ("compress"[All Fields] OR "compressed"[All Fields] OR "compresses"[All Fields] OR "compressibilities"[All Fields]

	OR "compressibility"[All Fields] OR "compressible"[All Fields] OR "compressing"[All Fields] OR "Compression"[All Fields] OR "compression s"[All Fields] OR "compressions"[All Fields] OR "compressive"[All Fields] OR "compressively"[All Fields])) AND "syndrome thoracic outlet"[Title/Abstract]) OR "neurogenic thoracic outlet syndrome"[Title/Abstract] OR "thoracic outlet syndrome neurogenic"[Title/Abstract] OR (("Neurologic"[All Fields] OR "neurological"[All Fields] OR "neurologically"[All Fields]) AND "syndrome thoracic outlet"[Title/Abstract]) OR ((("thoracal"[All Fields] OR "thoracical"[All Fields] OR "thorax"[MeSH Terms] OR "thorax"[All Fields] OR "Thoracic"[All Fields] OR "thoracics"[All Fields]) AND ("Outlet"[All Fields] OR "outlets"[All Fields])) AND "neurologic syndrome"[Title/Abstract]) OR "arterial thoracic outlet syndrome"[Title/Abstract] OR "venous thoracic outlet syndrome"[Title/Abstract] OR "Varicoceles"[Title/Abstract] OR "varicose vein"[Title/Abstract] OR "veins varicose"[Title/Abstract] OR "vein varicose"[Title/Abstract] OR "Varices"[Title/Abstract] OR "Varix"[Title/Abstract] OR "fistulas vascular"[Title/Abstract] OR "fistula vascular"[Title/Abstract] OR "vascular fistulas"[Title/Abstract] OR "neoplasms vascular"[Title/Abstract] OR "neoplasm vascular"[Title/Abstract] OR "vascular neoplasm"[Title/Abstract] OR (("injurie"[All Fields] OR "injuried"[All Fields] OR "Injuries"[MeSH Subheading] OR "Injuries"[All Fields] OR "wounds and injuries"[MeSH Terms] OR ("wounds"[All Fields] AND "Injuries"[All Fields]) OR "wounds and injuries"[All Fields] OR "injurious"[All Fields] OR "injury s"[All Fields] OR "injuryed"[All Fields] OR "injurys"[All Fields] OR "Injury"[All Fields]) AND "vascular system"[Title/Abstract]) OR (("injurie"[All Fields] OR "injuried"[All Fields] OR "Injuries"[MeSH Subheading] OR "Injuries"[All Fields] OR "wounds and injuries"[MeSH Terms] OR ("wounds"[All Fields] AND "Injuries"[All Fields]) OR "wounds and injuries"[All Fields] OR "injurious"[All Fields] OR "injury s"[All Fields] OR "injuryed"[All Fields] OR "injurys"[All Fields] OR "Injury"[All Fields]) AND "vascular system"[Title/Abstract]) OR (("System"[All Fields] OR "system s"[All Fields] OR "systems"[All Fields]) AND "injuries vascular"[Title/Abstract]) OR (("System"[All Fields] OR "system s"[All Fields] OR "systems"[All Fields]) AND "injury vascular"[Title/Abstract]) OR "vascular system injury"[Title/Abstract] OR "vascular injuries"[Title/Abstract] OR "injuries vascular"[Title/Abstract] OR "injury

	vascular"[Title/Abstract] OR "vascular injury"[Title/Abstract] OR "Vasculitides"[Title/Abstract] OR "Angiitis"[Title/Abstract] OR "Angiitides"[Title/Abstract] OR "Vasoplegias"[Title/Abstract] OR "postoperative vasoplegic syndrome"[Title/Abstract] OR "post operative vasoplegic syndrome"[Title/Abstract] OR "post operative vasoplegic syndrome"[Title/Abstract] OR (("postoperative period"[MeSH Terms] OR ("Postoperative"[All Fields] AND "period"[All Fields]) OR "postoperative period"[All Fields] OR ("Post"[All Fields] AND "operative"[All Fields]) OR "Post-operative"[All Fields]) AND "vasoplegic syndromes"[Title/Abstract]) OR (("syndrom"[All Fields] OR "syndromal"[All Fields] OR "syndromally"[All Fields] OR "Syndrome"[MeSH Terms] OR "Syndrome"[All Fields] OR "Syndromes"[All Fields] OR "syndrome s"[All Fields] OR "syndromic"[All Fields] OR "syndroms"[All Fields]) AND "post operative vasoplegic"[Title/Abstract]) OR (("syndrom"[All Fields] OR "syndromal"[All Fields] OR "syndromally"[All Fields] OR "Syndrome"[MeSH Terms] OR "Syndrome"[All Fields] OR "Syndromes"[All Fields] OR "syndrome s"[All Fields] OR "syndromic"[All Fields] OR "syndroms"[All Fields]) AND "post operative vasoplegic"[Title/Abstract]) OR ("Vasoplegic"[All Fields] AND "syndrome post operative"[Title/Abstract]) OR (("Vasoplegic"[All Fields] AND ("syndrom"[All Fields] OR "syndromal"[All Fields] OR "syndromally"[All Fields] OR "Syndrome"[MeSH Terms] OR "Syndrome"[All Fields] OR "Syndromes"[All Fields] OR "syndrome s"[All Fields] OR "syndromic"[All Fields] OR "syndroms"[All Fields])) AND "Post-operative"[Title/Abstract]) OR "vasoplegic syndrome"[Title/Abstract] OR (("Insufficiencies"[All Fields] OR "Insufficiency"[All Fields] OR "insufficient"[All Fields] OR "insufficiently"[All Fields]) AND "Venous"[Title/Abstract]) OR "insufficiency venous"[Title/Abstract] OR "venous insufficiencies"[Title/Abstract] OR "Hyperemias"[Title/Abstract] OR "venous engorgement"[Title/Abstract] OR "engorgement venous"[Title/Abstract] OR "passive hyperemia"[Title/Abstract] OR "hyperemia passive"[Title/Abstract] OR "venous congestion"[Title/Abstract] OR "congestion venous"[Title/Abstract] OR "active hyperemia"[Title/Abstract] OR (("hyperaemia"[All Fields] OR "Hyperemia"[MeSH Terms] OR "Hyperemia"[All Fields] OR "Hyperemias"[All Fields]) AND "Active"[Title/Abstract]) OR "arterial hyperemia"[Title/Abstract] OR "hyperemia arterial"[Title/Abstract] OR "reactive

	hyperemia"[Title/Abstract] OR "hyperemia reactive"[Title/Abstract] OR (("hyperaemia"[All Fields] OR "Hyperemia"[MeSH Terms] OR "Hyperemia"[All Fields] OR "Hyperemias"[All Fields]) AND "Reactive"[Title/Abstract]) OR "reactive hyperemias"[Title/Abstract] OR "hemostatic disorder"[Title/Abstract] OR (("haemostat"[All Fields] OR "haemostatically"[All Fields] OR "haemostatics"[All Fields] OR "hemostatics"[Pharmacological Action] OR "hemostatics"[MeSH Terms] OR "hemostatics"[All Fields] OR "haemostats"[All Fields] OR "hemostasis"[MeSH Terms] OR "hemostasis"[All Fields] OR "haemostatic"[All Fields] OR "hemostat"[All Fields] OR "hemostatically"[All Fields] OR "Hemostatic"[All Fields] OR "hemostats"[All Fields]) AND "disorders vascular"[Title/Abstract]) OR (("Disease"[MeSH Terms] OR "Disease"[All Fields] OR "Disorder"[All Fields] OR "Disorders"[All Fields] OR "disorder s"[All Fields] OR "disordes"[All Fields]) AND "vascular hemostatic"[Title/Abstract]) OR (("Disease"[MeSH Terms] OR "Disease"[All Fields] OR "Disorder"[All Fields] OR "Disorders"[All Fields] OR "disorder s"[All Fields] OR "disordes"[All Fields]) AND "vascular hemostatic"[Title/Abstract]) OR (("haemostat"[All Fields] OR "haemostatically"[All Fields] OR "haemostatics"[All Fields] OR "hemostatics"[Pharmacological Action] OR "hemostatics"[MeSH Terms] OR "hemostatics"[All Fields] OR "haemostats"[All Fields] OR "hemostasis"[MeSH Terms] OR "hemostasis"[All Fields] OR "haemostatic"[All Fields] OR "hemostat"[All Fields] OR "hemostatically"[All Fields] OR "Hemostatic"[All Fields] OR "hemostats"[All Fields]) AND "disorder vascular"[Title/Abstract]) OR (("blood vessels"[MeSH Terms] OR ("Blood"[All Fields] AND "vessels"[All Fields]) OR "blood vessels"[All Fields] OR "Vascular"[All Fields] OR "neovascularization, pathologic"[MeSH Terms] OR ("neovascularization"[All Fields] AND "pathologic"[All Fields]) OR "pathologic neovascularization"[All Fields] OR "vascularisation"[All Fields] OR "vascularization"[All Fields] OR "vascularisations"[All Fields] OR "vascularise"[All Fields] OR "vascularised"[All Fields] OR "vascularities"[All Fields] OR "vascularitis"[All Fields] OR "vascularity"[All Fields] OR "vascularizations"[All Fields] OR "vascularize"[All Fields] OR "vascularized"[All Fields] OR "vascularizes"[All Fields] OR "vascularizing"[All Fields] OR "vasculars"[All Fields]) AND "hemostatic disorder"[Title/Abstract]) OR (("blood vessels"[MeSH Terms] OR ("Blood"[All Fields] AND "vessels"[All Fields]) OR

	"blood vessels"[All Fields] OR "Vascular"[All Fields] OR "neovascularization, pathologic"[MeSH Terms] OR ("neovascularization"[All Fields] AND "pathologic"[All Fields]) OR "pathologic neovascularization"[All Fields] OR "vascularisation"[All Fields] OR "vascularization"[All Fields] OR "vascularisations"[All Fields] OR "vascularise"[All Fields] OR "vascularised"[All Fields] OR "vascularities"[All Fields] OR "vascularitis"[All Fields] OR "vascularity"[All Fields] OR "vascularizations"[All Fields] OR "vascularize"[All Fields] OR "vascularized"[All Fields] OR "vascularizes"[All Fields] OR "vascularizing"[All Fields] OR "vasculars"[All Fields]) AND "hemostatic disorders"[Title/Abstract]) OR "Endocarditides"[Title/Abstract] OR "infective endocarditis"[Title/Abstract] OR (("Endocarditis"[MeSH Terms] OR "Endocarditis"[All Fields] OR "Endocarditides"[All Fields]) AND "Infective"[Title/Abstract]) OR "endocarditis infective"[Title/Abstract] OR (("infect"[All Fields] OR "infectability"[All Fields] OR "infectable"[All Fields] OR "infectant"[All Fields] OR "infectants"[All Fields] OR "infected"[All Fields] OR "infecteds"[All Fields] OR "infectibility"[All Fields] OR "infectible"[All Fields] OR "infecting"[All Fields] OR "infection s"[All Fields] OR "Infections"[MeSH Terms] OR "Infections"[All Fields] OR "Infection"[All Fields] OR "Infective"[All Fields] OR "infectiveness"[All Fields] OR "infectives"[All Fields] OR "infectivities"[All Fields] OR "infects"[All Fields] OR "pathogenicity"[MeSH Subheading] OR "pathogenicity"[All Fields] OR "infectivity"[All Fields]) AND "Endocarditides"[Title/Abstract]) OR "Cardiomyopathy"[Title/Abstract] OR "myocardial disease"[Title/Abstract] OR "disease myocardial"[Title/Abstract] OR "diseases myocardial"[Title/Abstract] OR "myocardial diseases"[Title/Abstract] OR "Myocardiopathies"[Title/Abstract] OR "Myocardiopathy"[Title/Abstract] OR "cardiomyopathies primary"[Title/Abstract] OR "cardiomyopathy primary"[Title/Abstract] OR "primary cardiomyopathies"[Title/Abstract] OR "primary cardiomyopathy"[Title/Abstract] OR "primary myocardial disease"[Title/Abstract] OR "disease primary myocardial"[Title/Abstract] OR "diseases primary myocardial"[Title/Abstract] OR (("myocardially"[All Fields] OR "Myocardium"[MeSH Terms] OR "Myocardium"[All Fields] OR "Myocardial"[All Fields]) AND "disease primary"[Title/Abstract]) OR "myocardial diseases primary"[Title/Abstract] OR "primary myocardial diseases"[Title/Abstract] OR "cardiomyopathies secondary"[Title/Abstract] OR

	"cardiomyopathy secondary"[Title/Abstract] OR "secondary cardiomyopathies"[Title/Abstract] OR "secondary cardiomyopathy"[Title/Abstract] OR (("myocardially"[All Fields] OR "Myocardium"[MeSH Terms] OR "Myocardium"[All Fields] OR "Myocardial"[All Fields]) AND "diseases secondary"[Title/Abstract]) OR (("Disease"[MeSH Terms] OR "Disease"[All Fields] OR "Diseases"[All Fields] OR "disease s"[All Fields] OR "diseased"[All Fields]) AND "secondary myocardial"[Title/Abstract]) OR (("Disease"[MeSH Terms] OR "Disease"[All Fields] OR "Diseases"[All Fields] OR "disease s"[All Fields] OR "diseased"[All Fields]) AND "secondary myocardial"[Title/Abstract]) OR "myocardial disease secondary"[Title/Abstract] OR "secondary myocardial disease"[Title/Abstract] OR "secondary myocardial diseases"[Title/Abstract] OR "Cardiotoxicities"[Title/Abstract] OR "cardiac toxicity"[Title/Abstract] OR "cardiac toxicities"[Title/Abstract] OR "toxicity cardiac"[Title/Abstract] OR "aneurysm heart"[Title/Abstract] OR "aneurysms heart"[Title/Abstract] OR "heart aneurysms"[Title/Abstract] OR "cardiac aneurysm"[Title/Abstract] OR "aneurysm cardiac"[Title/Abstract] OR "aneurysms cardiac"[Title/Abstract] OR "cardiac aneurysms"[Title/Abstract] OR "enlarged heart"[Title/Abstract] OR "heart enlarged"[Title/Abstract] OR "heart enlargement"[Title/Abstract] OR "enlargement heart"[Title/Abstract] OR "cardiac hypertrophy"[Title/Abstract] OR "cardiac hypertrophies"[Title/Abstract] OR (("Hypertrophy"[MeSH Terms] OR "Hypertrophy"[All Fields] OR "hypertrophied"[All Fields] OR "Hypertrophies"[All Fields] OR "hypertrophying"[All Fields]) AND "Cardiac"[Title/Abstract]) OR "hypertrophy cardiac"[Title/Abstract] OR "heart hypertrophy"[Title/Abstract] OR "heart hypertrophies"[Title/Abstract] OR (("Hypertrophy"[MeSH Terms] OR "Hypertrophy"[All Fields] OR "hypertrophied"[All Fields] OR "Hypertrophies"[All Fields] OR "hypertrophying"[All Fields]) AND "Heart"[Title/Abstract]) OR "hypertrophy heart"[Title/Abstract] OR "arrest heart"[Title/Abstract] OR "Asystole"[Title/Abstract] OR "Asystoles"[Title/Abstract] OR "cardiac arrest"[Title/Abstract] OR "arrest cardiac"[Title/Abstract] OR "cardiopulmonary arrest"[Title/Abstract] OR "arrest cardiopulmonary"[Title/Abstract] OR "defect congenital heart"[Title/Abstract] OR "abnormality heart"[Title/Abstract] OR "heart abnormality"[Title/Abstract] OR "congenital heart defect"[Title/Abstract] OR "congenital

	heart defects"[Title/Abstract] OR "defects congenital heart"[Title/Abstract] OR "heart abnormalities"[Title/Abstract] OR "heart defect congenital"[Title/Abstract] OR "congenital heart disease"[Title/Abstract] OR "congenital heart diseases"[Title/Abstract] OR "disease congenital heart"[Title/Abstract] OR "heart disease congenital"[Title/Abstract] OR "heart malformation of"[Title/Abstract] OR "malformation of heart"[Title/Abstract] OR (("Abnormalities"[MeSH Subheading] OR "Abnormalities"[All Fields] OR "Malformations"[All Fields] OR "congenital abnormalities"[MeSH Terms] OR ("Congenital"[All Fields] AND "Abnormalities"[All Fields]) OR "congenital abnormalities"[All Fields] OR "Malformation"[All Fields] OR "malformational"[All Fields] OR "malformative"[All Fields] OR "malformed"[All Fields]) AND "Hearts"[Title/Abstract]) OR "cardiac failure"[Title/Abstract] OR "heart decompensation"[Title/Abstract] OR "decompensation heart"[Title/Abstract] OR "congestive heart failure"[Title/Abstract] OR "heart failure congestive"[Title/Abstract] OR "heart failure right sided"[Title/Abstract] OR "heart failure right sided"[Title/Abstract] OR "right sided heart failure"[Title/Abstract] OR "right sided heart failure"[Title/Abstract] OR "heart failure left sided"[Title/Abstract] OR "heart failure left sided"[Title/Abstract] OR "left sided heart failure"[Title/Abstract] OR "left sided heart failure"[Title/Abstract] OR "myocardial failure"[Title/Abstract] OR "heart neoplasm"[Title/Abstract] OR "neoplasm heart"[Title/Abstract] OR "cardiac neoplasms"[Title/Abstract] OR "cardiac neoplasm"[Title/Abstract] OR "neoplasm cardiac"[Title/Abstract] OR "cardiac tumor"[Title/Abstract] OR "tumor cardiac"[Title/Abstract] OR "tumors cardiac"[Title/Abstract] OR "cardiac tumors"[Title/Abstract] OR "neoplasms cardiac"[Title/Abstract] OR "neoplasms heart"[Title/Abstract] OR "cardiac carcinoma"[Title/Abstract] OR "carcinoma cardiac"[Title/Abstract] OR "carcinomas cardiac"[Title/Abstract] OR "cardiac carcinomas"[Title/Abstract] OR "cardiac cancer"[Title/Abstract] OR "cancer cardiac"[Title/Abstract] OR "cancers cardiac"[Title/Abstract] OR "cardiac cancers"[Title/Abstract] OR "heart cancer"[Title/Abstract] OR "cancer heart"[Title/Abstract] OR "cancers heart"[Title/Abstract] OR "heart cancers"[Title/Abstract] OR "heart tumor"[Title/Abstract] OR "heart tumors"[Title/Abstract] OR "tumor heart"[Title/Abstract] OR "tumors heart"[Title/Abstract] OR "intracavitary tumors of the heart"[Title/Abstract] OR

	((("primaries"[All Fields] OR "Primary"[All Fields]) AND ("cardiacs"[All Fields] OR "Heart"[MeSH Terms] OR "Heart"[All Fields] OR "Cardiac"[All Fields])) AND "tumors childhood"[Title/Abstract]) OR (("myocardially"[All Fields] OR "Myocardium"[MeSH Terms] OR "Myocardium"[All Fields] OR "Myocardial"[All Fields]) AND ("cysts"[MeSH Terms] OR "cysts"[All Fields] OR "cyst"[All Fields] OR "neurofibroma"[MeSH Terms] OR "neurofibroma"[All Fields] OR "neurofibromas"[All Fields] OR "tumor s"[All Fields] OR "tumoral"[All Fields] OR "tumorous"[All Fields] OR "tumour"[All Fields] OR "Neoplasms"[MeSH Terms] OR "Neoplasms"[All Fields] OR "Tumor"[All Fields] OR "tumour s"[All Fields] OR "tumoural"[All Fields] OR "tumourous"[All Fields] OR "tumours"[All Fields] OR "Tumors"[All Fields]))) AND ("Rhabdomyomas"[Title/Abstract] AND "Fibromas"[Title/Abstract])) OR (("myocardially"[All Fields] OR "Myocardium"[MeSH Terms] OR "Myocardium"[All Fields] OR "Myocardial"[All Fields]) AND ("cysts"[MeSH Terms] OR "cysts"[All Fields] OR "cyst"[All Fields] OR "neurofibroma"[MeSH Terms] OR "neurofibroma"[All Fields] OR "neurofibromas"[All Fields] OR "tumor s"[All Fields] OR "tumoral"[All Fields] OR "tumorous"[All Fields] OR "tumour"[All Fields] OR "Neoplasms"[MeSH Terms] OR "Neoplasms"[All Fields] OR "Tumor"[All Fields] OR "tumour s"[All Fields] OR "tumoural"[All Fields] OR "tumourous"[All Fields] OR "tumours"[All Fields] OR "Tumors"[All Fields]))) AND ("Rhabdomyomas"[Title/Abstract] AND "Fibromas"[Title/Abstract])) OR (("cysts"[MeSH Terms] OR "cysts"[All Fields] OR "cyst"[All Fields] OR "neurofibroma"[MeSH Terms] OR "neurofibroma"[All Fields] OR "neurofibromas"[All Fields] OR "tumor s"[All Fields] OR "tumoral"[All Fields] OR "tumorous"[All Fields] OR "tumour"[All Fields] OR "Neoplasms"[MeSH Terms] OR "Neoplasms"[All Fields] OR "Tumor"[All Fields] OR "tumour s"[All Fields] OR "tumoural"[All Fields] OR "tumourous"[All Fields] OR "tumours"[All Fields] OR "Tumors"[All Fields]) AND ("myocardially"[All Fields] OR "Myocardium"[MeSH Terms] OR "Myocardium"[All Fields] OR "Myocardial"[All Fields]))) AND ("Rhabdomyomas"[Title/Abstract] AND "Fibromas"[Title/Abstract])) OR (("cysts"[MeSH Terms] OR "cysts"[All Fields] OR "cyst"[All Fields] OR "neurofibroma"[MeSH Terms] OR "neurofibroma"[All Fields] OR "neurofibromas"[All Fields] OR "tumor s"[All Fields] OR "tumoral"[All Fields] OR "tumorous"[All Fields] OR "tumour"[All Fields] OR "Neoplasms"[MeSH Terms] OR "Neoplasms"[All Fields] OR

	"Tumor"[All Fields] OR "tumour s"[All Fields] OR "tumoural"[All Fields] OR "tumourous"[All Fields] OR "tumours"[All Fields] OR "Tumors"[All Fields]) AND ("myocardially"[All Fields] OR "Myocardium"[MeSH Terms] OR "Myocardium"[All Fields] OR "Myocardial"[All Fields]))) AND ("Rhabdomyomas"[Title/Abstract] AND "Fibromas"[Title/Abstract])) OR "cardiac tamponades"[Title/Abstract] OR "tamponade cardiac"[Title/Abstract] OR (("Tamponade"[All Fields] OR "tamponaded"[All Fields] OR "Tamponades"[All Fields] OR "tamponading"[All Fields]) AND "Cardiac"[Title/Abstract]) OR "pericardial tamponade"[Title/Abstract] OR "pericardial tamponades"[Title/Abstract] OR "tamponade pericardial"[Title/Abstract] OR (("Tamponade"[All Fields] OR "tamponaded"[All Fields] OR "Tamponades"[All Fields] OR "tamponading"[All Fields]) AND "Pericardial"[Title/Abstract]) OR "output low cardiac"[Title/Abstract] OR "low cardiac output"[Title/Abstract] OR "low cardiac output syndrome"[Title/Abstract] OR "cardiovascular diseases"[Title/Abstract] OR "cardiovascular abnormalities"[Title/Abstract] OR "heart defects congenital"[Title/Abstract] OR "vascular malformations"[Title/Abstract] OR "cardiovascular infections"[Title/Abstract] OR "endocarditis bacterial"[Title/Abstract] OR "syphilis cardiovascular"[Title/Abstract] OR "tuberculosis cardiovascular"[Title/Abstract] OR "heart diseases"[Title/Abstract] OR "arrhythmias cardiac"[Title/Abstract] OR "carcinoid heart disease"[Title/Abstract] OR "cardiac conduction system disease"[Title/Abstract] OR "cardiac output high"[Title/Abstract] OR "cardiac output low"[Title/Abstract] OR "cardiac tamponade"[Title/Abstract] OR "Cardiomegaly"[Title/Abstract] OR "Cardiomyopathies"[Title/Abstract] OR "Cardiotoxicity"[Title/Abstract] OR "Endocarditis"[Title/Abstract] OR "heart aneurysm"[Title/Abstract] OR "heart arrest"[Title/Abstract] OR "heart defects congenital"[Title/Abstract] OR "heart failure"[Title/Abstract] OR "heart neoplasms"[Title/Abstract] OR "heart rupture"[Title/Abstract] OR "heart valve diseases"[Title/Abstract] OR "myocardial ischemia"[Title/Abstract] OR "myocardial stunning"[Title/Abstract] OR "pericardial effusion"[Title/Abstract] OR "Pericarditis"[Title/Abstract] OR "Pneumopericardium"[Title/Abstract] OR "post cardiac arrest syndrome"[Title/Abstract] OR "postpericardiotomy syndrome"[Title/Abstract] OR "pulmonary heart disease"[Title/Abstract] OR "rheumatic heart disease"[Title/Abstract] OR "ventricular

	dysfunction"[Title/Abstract] OR "ventricular outflow obstruction"[Title/Abstract] OR "pregnancy complications cardiovascular"[Title/Abstract] OR "embolism amniotic fluid"[Title/Abstract] OR "vascular diseases"[Title/Abstract] OR "Aneurysm"[Title/Abstract] OR "aneurysm false"[Title/Abstract] OR "Angiodysplasia"[Title/Abstract] OR "Angioedema"[Title/Abstract] OR "Angiomatosis"[Title/Abstract] OR "aortic diseases"[Title/Abstract] OR "arterial occlusive diseases"[Title/Abstract] OR "arteriovenous malformations"[Title/Abstract] OR "capillary leak syndrome"[Title/Abstract] OR "cerebrovascular disorders"[Title/Abstract] OR "colitis ischemic"[Title/Abstract] OR "compartment syndromes"[Title/Abstract] OR "diabetic angiopathies"[Title/Abstract] OR "Embolism"[Title/Abstract]) AND "Thrombosis"[Title/Abstract]) OR "hand arm vibration syndrome"[Title/Abstract] OR "Hemorrhoids"[Title/Abstract] OR "hemostatic disorders"[Title/Abstract] OR "hepatic veno occlusive disease"[Title/Abstract] OR "Hyperemia"[Title/Abstract] OR "Hypertension"[Title/Abstract] OR "Hypotension"[Title/Abstract] OR "mesenteric ischemia"[Title/Abstract] OR "myocardial ischemia"[Title/Abstract] OR "optic neuropathy ischemic"[Title/Abstract] OR "peliosis hepatis"[Title/Abstract] OR "penetrating atherosclerotic ulcer"[Title/Abstract] OR "peripheral vascular diseases"[Title/Abstract] OR "Prehypertension"[Title/Abstract] OR "pulmonary veno occlusive disease"[Title/Abstract] OR "reperfusion injury"[Title/Abstract] OR "retinal vein occlusion"[Title/Abstract] OR "scimitar syndrome"[Title/Abstract] OR "spinal cord vascular diseases"[Title/Abstract] OR "splenic infarction"[Title/Abstract] OR "stenosis pulmonary vein"[Title/Abstract] OR "superior vena cava syndrome"[Title/Abstract] OR "Telangiectasis"[Title/Abstract] OR "thoracic outlet syndrome"[Title/Abstract] OR "Varicocele"[Title/Abstract] OR "varicose veins"[Title/Abstract] OR "vascular fistula"[Title/Abstract] OR "vascular neoplasms"[Title/Abstract] OR "vascular system injuries"[Title/Abstract] OR "Vasculitis"[Title/Abstract] OR "Vasoplegia"[Title/Abstract] OR "venous insufficiency"[Title/Abstract])
Scopus	TITLE-ABS-KEY ((Cardiovascular Disease OR Disease, Cardiovascular OR Cardiac Events OR Cardiac Event OR Event, Cardiac OR Adverse Cardiac Event OR Adverse Cardiac Events OR Cardiac Event, Adverse OR Cardiac Events, Adverse OR Major Adverse Cardiac Events OR Abnormalities, Cardiovascular OR Abnormality,

	Cardiovascular OR Cardiovascular Abnormality OR Defect, Congenital Heart OR Abnormality, Heart OR Heart Abnormality OR Congenital Heart Defect OR Congenital Heart Defects OR Defects, Congenital Heart OR Heart Abnormalities OR Heart Defect, Congenital OR Congenital Heart Disease OR Congenital Heart Diseases OR Disease, Congenital Heart OR Heart Disease, Congenital OR Heart, Malformation Of OR Malformation Of Heart OR Malformation Of Hearts OR Malformations, Vascular OR Malformation, Vascular OR Vascular Malformation OR Cardiovascular Infection OR Infection, Cardiovascular OR Infections, Cardiovascular OR Bacterial Endocarditides OR Bacterial Endocarditis OR Endocarditides, Bacterial OR Cardiovascular Syphilis OR Aortitis, Syphilitic OR Aortitides, Syphilitic OR Syphilitic Aortitides OR Syphilitic Aortitis OR Heart Disease OR Cardiac Disorders OR Cardiac Disorder OR Heart Disorders OR Heart Disorder OR Cardiac Diseases OR Cardiac Disease OR Arrhythmia, Cardiac OR Arrhythmia OR Arrythmia OR Cardiac Arrhythmia OR Cardiac Arrhythmias OR Cardiac Dysrhythmia OR Dysrhythmia, Cardiac OR Carcinoid Heart Diseases OR Heart Disease, Carcinoid OR Heart Diseases, Carcinoid OR Cardiac Conduction System Diseases OR Cardiac Conduction Defects OR Conduction Defect, Cardiac OR Defect, Cardiac Conduction OR Cardiac Conduction Defect OR Post Cardiac Arrest Syndrome OR Post-Cardiac Arrest Syndromes OR Postcardiac Arrest Syndrome OR Postcardiac Arrest Syndromes OR Postresuscitation Disease OR Postresuscitation Diseases OR Postpericardiotomy Syndromes OR Syndrome, Postpericardiotomy OR Syndromes, Postpericardiotomy OR Postcommissurotomy Syndrome OR Postcommissurotomy Syndromes OR Syndrome, Postcommissurotomy OR Syndromes, Postcommissurotomy OR Cor Pulmonale OR Disease, Pulmonary Heart OR Diseases, Pulmonary Heart OR Heart Disease, Pulmonary OR Heart Diseases, Pulmonary OR Pulmonary Heart Diseases OR Disease, Rheumatic Heart OR Diseases, Rheumatic Heart OR Heart Disease, Rheumatic OR Heart Diseases, Rheumatic OR Rheumatic Heart Diseases OR Bouillaud Disease OR Disease, Bouillaud OR Bouillaud's Disease OR Bouillauds Disease OR Disease, Bouillaud's OR Dysfunctions, Ventricular OR Dysfunction, Ventricular OR Ventricular Dysfunctions OR Obstruction, Ventricular Outflow OR Outflow Obstruction, Ventricular OR Ventricular Outflow Obstructions OR Cardiovascular Pregnancy Complications OR Cardiovascular Pregnancy Complication OR Complication, Cardiovascular Pregnancy OR Pregnancy

	Complication, Cardiovascular OR Complications, Cardiovascular Pregnancy OR Pregnancy, Cardiovascular Complications OR Pregnancies, Cardiovascular Complications OR Amniotic Fluid Embolisms OR Embolisms, Amniotic Fluid OR Amniotic Fluid Embolism OR Diseases, Vascular OR Disease, Vascular OR Vascular Disease OR Aneurysms OR Fusiform Aneurysm OR Aneurysm, Fusiform OR Aneurysms, Fusiform OR Fusiform Aneurysms OR Saccular Aneurysm OR Aneurysms, False OR False Aneurysms OR Pseudoaneurysm OR Pseudoaneurysms OR False Aneurysm OR Angiodysplasias OR Angioedemas OR Urticaria, Giant OR Giant Urticaria OR Giant Urticarias OR Urticarias, Giant OR Angioneurotic Edema OR Angioneurotic Edemas OR Edema, Angioneurotic OR Edemas, Angioneurotic OR Quincke's Edema OR Edema, Quincke's OR Quincke Edema OR Quinckes Edema OR Angiomatoses OR Aortic Disease OR Disease, Aortic OR Diseases, Aortic OR Arterial Occlusive Disease OR Disease, Arterial Occlusive OR Occlusive Disease, Arterial OR Arterial Obstructive Diseases OR Arterial Obstructive Disease OR Disease, Arterial Obstructive OR Obstructive Disease, Arterial OR Arterial Occlusion OR Arterial Occlusions OR Occlusion, Arterial OR Arteriovenous Malformation OR Malformation, Arteriovenous OR Malformations, Arteriovenous OR Capillary Leak Syndromes OR Clarkson Disease OR Systemic Capillary Leak Syndrome OR Clinical Capillary Leak Syndrome OR Cerebrovascular Disorder OR Brain Vascular Disorders OR Brain Vascular Disorder OR Vascular Disorder, Brain OR Vascular Disorders, Brain OR Intracranial Vascular Disorders OR Intracranial Vascular Disorder OR Vascular Disorder, Intracranial OR Vascular Disorders, Intracranial OR Vascular Diseases, Intracranial OR Intracranial Vascular Disease OR Intracranial Vascular Diseases OR Vascular Disease, Intracranial OR Cerebrovascular Diseases OR Cerebrovascular Disease OR Disease, Cerebrovascular OR Diseases, Cerebrovascular OR Cerebrovascular Insufficiency OR Cerebrovascular Insufficiencies OR Insufficiencies, Cerebrovascular OR Insufficiency, Cerebrovascular OR Cerebrovascular Occlusion OR Cerebrovascular Occlusions OR Occlusion, Cerebrovascular OR Occlusions, Cerebrovascular OR Ischemic Colitis OR Compartment Syndrome OR Angiopathies, Diabetic OR Angiopathy, Diabetic OR Diabetic Angiopathy OR Diabetic Vascular Complications OR Diabetic Vascular Complication OR Vascular Complication, Diabetic OR Vascular Complications, Diabetic OR Diabetic Vascular Diseases OR Diabetic Vascular Disease OR Vascular Disease, Diabetic OR

	Vascular Diseases, Diabetic OR Microangiopathy, Diabetic OR Diabetic Microangiopathies OR Diabetic Microangiopathy OR Microangiopathies, Diabetic OR Thrombosis and Embolism OR Hand Arm Vibration Syndrome OR Hand-Arm Vibration Syndromes OR Syndrome, Hand-Arm Vibration OR Syndromes, Hand-Arm Vibration OR Vibration Syndrome, Hand-Arm OR Vibration Syndromes, Hand-Arm OR Effusion, Pericardial OR Effusions, Pericardial OR Pericardial Effusions OR Hemopericardium OR Chylopericardium OR Chylopericardiums OR Pericarditis OR Pleuropericarditis OR Heart Ruptures OR Cardiac Rupture OR Cardiac Ruptures OR Free Wall Rupture, Heart OR Cardiac Free Wall Rupture OR Ventricular Free Wall Rupture OR Heart Valve Disease OR Valve Disease, Heart OR Valvular Heart Diseases OR Heart Disease, Valvular OR Valvular Heart Disease OR Heart Valvular Disease OR Disease, Heart Valvular OR Heart Valvular Diseases OR Valvular Disease, Heart OR Heart Disease, Ischemic OR Disease, Ischemic Heart OR Diseases, Ischemic Heart OR Heart Diseases, Ischemic OR Ischemic Heart Diseases OR Ischemia, Myocardial OR Ischemias, Myocardial OR Myocardial Ischemias OR Ischemic Heart Disease Stunning, Myocardial OR Stunned Myocardium OR Myocardium, Stunned OR Hibernation, Myocardial OR Myocardial Hibernation OR Disease, Hepatic Veno-Occlusive OR Hepatic Veno-Occlusive Diseases OR Hepatic Veno Occlusive Disease OR Veno-Occlusive Disease, Hepatic OR Veno Occlusive Disease, Hepatic OR Sinusoidal Obstruction Syndrome OR Syndrome, Sinusoidal Obstruction OR Blood Pressure, High OR Blood Pressures, High OR High Blood Pressure OR High Blood Pressures OR Blood Pressure, Low OR Hypotension, Vascular OR Low Blood Pressure OR Vascular Hypotension OR Ischemia, Mesenteric OR Ischemias, Mesenteric OR Mesenteric Ischemias OR Mesenteric Vascular Insufficiency OR Insufficiencies, Mesenteric Vascular OR Insufficiency, Mesenteric Vascular OR Mesenteric Vascular Insufficiencies OR Vascular Insufficiencies, Mesenteric OR Vascular Insufficiency, Mesenteric OR Occlusive Mesenteric Arterial Ischemia OR Acute Mesenteric Arterial Embolus OR Nonocclusive Mesenteric Ischemia OR Ischemia, Nonocclusive Mesenteric OR Ischemias, Nonocclusive Mesenteric OR Mesenteric Ischemia, Nonocclusive OR Mesenteric Ischemias, Nonocclusive OR Nonocclusive Mesenteric Ischemias OR Acute Mesenteric Arterial Thrombosis OR Mesenteric Venous Thrombosis OR Mesenteric Venous Thromboses OR Thromboses, Mesenteric Venous OR Thrombosis, Mesenteric Venous OR Venous

	Thromboses, Mesenteric OR Venous Thrombosis, Mesenteric OR Heart Disease, Ischemic OR Disease, Ischemic Heart OR Diseases, Ischemic Heart OR Heart Diseases, Ischemic OR Ischemic Heart Diseases OR Ischemia, Myocardial OR Ischemias, Myocardial OR Myocardial Ischemias OR Ischemic Heart Disease OR Myocardial Ischemia OR Optic Ischaemic Neuropathy OR Ischaemic Neuropathy, Optic OR Neuropathy, Optic Ischaemic OR Optic Ischaemic Neuropathies OR Optic Ischemic Neuropathy OR Ischemic Neuropathy, Optic OR Neuropathy, Optic Ischemic OR Optic Ischemic Neuropathies OR Ischemic Optic Neuropathy OR Ischemic Optic Neuropathies OR Neuropathy, Ischemic Optic OR Optic Nerve Ischemia OR Ischemia, Optic Nerve OR Nerve Ischemia, Optic OR Optic Nerve Ischemias OR Anterior Ischemic Optic Neuropathy OR Optic Neuropathy, Anterior Ischemic OR Posterior Ischemic Optic Neuropathy OR Optic Neuropathy, Posterior Ischemic OR Nonarteritic Anterior Ischemic Optic Neuropathy OR Hepatis, Peliosis OR Atherosclerotic Ulcer, Penetrating OR Penetrating Atherosclerotic Ulcers OR Ulcer, Penetrating Atherosclerotic OR Penetrating Aortic Ulcer OR Aortic Ulcer, Penetrating OR Penetrating Aortic Ulcers OR Ulcer, Penetrating Aortic OR Penetrating Ulcer Aorta OR Penetrating Ulcer Aortas OR Aortic Penetrating Ulcer OR Aortic Penetrating Ulcers OR Penetrating Ulcer, Aortic OR Ulcer, Aortic Penetrating OR Penetrating Ulcer OR Penetrating Ulcers OR Ulcer, Penetrating OR Disease, Peripheral Vascular OR Peripheral Vascular Disease OR Vascular Disease, Peripheral OR Diseases, Peripheral Vascular OR Peripheral Angiopathies OR Angiopathies, Peripheral OR Angiopathy, Peripheral OR Peripheral Angiopathy OR Vascular Diseases, Peripheral OR Pre-Hypertension OR Pre Hypertension OR Diseases, Pulmonary Veno-Occlusive OR Pulmonary Veno-Occlusive Diseases OR Veno-Occlusive Diseases, Pulmonary OR Pulmonary Veno Occlusive Disease OR Veno-Occlusive Disease, Pulmonary OR Veno Occlusive Disease, Pulmonary OR Pulmonary Venoocclusive Disease OR Pulmonary Venoocclusive Diseases OR Venoocclusive Disease, Pulmonary OR Venoocclusive Diseases, Pulmonary OR Reperfusion Injuries OR Injury, Ischemia-Reperfusion OR Injury, Ischemia Reperfusion OR Ischemia-Reperfusion Injuries OR Injury, Reperfusion OR Ischemia-Reperfusion Injury OR Ischemia Reperfusion Injury OR Reperfusion Damage OR Damage, Reperfusion OR Reperfusion Damages OR Occlusion, Retinal Vein OR Retinal Vein Occlusions OR Vein Occlusion, Retinal OR Retinal Vein Thrombosis OR Retinal Vein

	Thromboses OR Vein Thrombosis, Retinal OR Thrombosis, Retinal Vein OR Branch Vein Occlusion OR Branch Vein Occlusions OR Occlusion, Branch Vein OR Vein Occlusion, Branch OR Branch Retinal Vein Occlusion OR Retinal Branch Vein Occlusion OR Central Retinal Vein Occlusion OR Scimitar Anomaly OR Anomaly, Scimitar OR Total Anomalous Pulmonary Venous Return OR Total Anomalous Pulmonary Venous Return OR Anomalous Pulmonary Venous Return OR Pulmonary Venous Return Anomaly OR Vascular Diseases, Spinal Cord OR Hematomyelia OR Posterior Spinal Artery Syndrome OR Infarction, Splenic OR Infarctions, Splenic OR Splenic Infarctions OR Splenic Infarct OR Infarct, Splenic OR Infarcts, Splenic OR Splenic Infarcts OR Infarct of the Spleen OR Pulmonary Vein Stenoses OR Pulmonary Vein Stenosis OR Stenoses, Pulmonary Vein OR Vein Stenoses, Pulmonary OR Vein Stenosis, Pulmonary OR SVC Syndrome OR SVC Syndromes OR Syndrome, SVC OR Obstruction of the Superior Vena Cava OR SVC Obstruction OR Obstruction, SVC OR SVC Obstructions OR Superior Vena Cava Obstruction OR Superior Vena Cava Thrombosis OR SVC Thrombosis OR SVC Thromboses OR Thrombosis, SVC OR Telangiectases OR Telangiectasia OR Telangiectasias OR Spider Veins OR Spider Vein OR Vein, Spider OR Veins, Spider OR Outlet Syndromes, Thoracic OR Outlet Syndrome, Thoracic OR Syndromes, Thoracic Outlet OR Syndrome, Thoracic Outlet OR Thoracic Outlet Syndromes OR Aperture Syndrome, Thoracic Outlet OR Neurovascular Syndrome, Thoracic Outlet OR Superior Thoracic Aperture Syndrome OR Thoracic Outlet Neurovascular Syndrome OR Scalenus Anticus Syndrome OR Syndrome, Scalenus Anticus OR Costoclavicular Syndrome OR Costoclavicular Syndromes OR Syndrome, Costoclavicular OR Syndromes, Costoclavicular OR Thoracic Outlet Nerve Compression Syndrome OR Nerve Compression Syndrome, Thoracic Outlet OR Neurogenic Thoracic Outlet Syndrome OR Thoracic Outlet Syndrome, Neurogenic OR Neurologic Syndrome, Thoracic Outlet OR Thoracic Outlet Neurologic Syndrome OR Arterial Thoracic Outlet Syndrome OR Venous Thoracic Outlet Syndrome OR Varicoceles OR Varicose Vein OR Veins, Varicose OR Vein, Varicose OR Varices OR Varix OR Fistulas, Vascular OR Fistula, Vascular OR Vascular Fistulas OR Neoplasms, Vascular OR Neoplasm, Vascular OR Vascular Neoplasm OR Injuries, Vascular System OR Injury, Vascular System OR System Injuries, Vascular OR System Injury, Vascular OR Vascular System Injury OR Vascular Injuries OR Injuries, Vascular

	OR Injury, Vascular OR Vascular Injury OR Vasculitides OR Angiitis OR Angiitides OR Vasoplegias OR Postoperative Vasoplegic Syndrome OR Post-operative Vasoplegic Syndrome OR Post operative Vasoplegic Syndrome OR Post-operative Vasoplegic Syndromes OR Syndrome, Post-operative Vasoplegic OR Syndromes, Post-operative Vasoplegic OR Vasoplegic Syndrome, Post-operative OR Vasoplegic Syndromes, Post-operative OR Vasoplegic Syndrome OR Insufficiencies, Venous OR Insufficiency, Venous OR Venous Insufficiencies OR Hyperemias OR Venous Engorgement OR Engorgement, Venous OR Passive Hyperemia OR Hyperemia, Passive OR Venous Congestion OR Congestion, Venous OR Active Hyperemia OR Hyperemia, Active OR Arterial Hyperemia OR Hyperemia, Arterial OR Reactive Hyperemia OR Hyperemia, Reactive OR Hyperemias, Reactive OR Reactive Hyperemias OR Hemostatic Disorder OR Hemostatic Disorders, Vascular OR Disorders, Vascular Hemostatic OR Disorder, Vascular Hemostatic OR Hemostatic Disorder, Vascular OR Vascular Hemostatic Disorder OR Vascular Hemostatic Disorders OR Endocarditides OR Infective Endocarditis OR Endocarditides, Infective OR Endocarditis, Infective OR Infective Endocarditides OR Cardiomyopathy OR Myocardial Disease OR Disease, Myocardial OR Diseases, Myocardial OR Myocardial Diseases OR Myocardiopathies OR Myocardiopathy OR Cardiomyopathies, Primary OR Cardiomyopathy, Primary OR Primary Cardiomyopathies OR Primary Cardiomyopathy OR Primary Myocardial Disease OR Disease, Primary Myocardial OR Diseases, Primary Myocardial OR Myocardial Disease, Primary OR Myocardial Diseases, Primary OR Primary Myocardial Diseases OR Cardiomyopathies, Secondary OR Cardiomyopathy, Secondary OR Secondary Cardiomyopathies OR Secondary Cardiomyopathy OR Myocardial Diseases, Secondary OR Disease, Secondary Myocardial OR Diseases, Secondary Myocardial OR Myocardial Disease, Secondary OR Secondary Myocardial Disease OR Secondary Myocardial Diseases OR Cardiotoxicities OR Cardiac Toxicity OR Cardiac Toxicities OR Toxicity, Cardiac OR Aneurysm, Heart OR Aneurysms, Heart OR Heart Aneurysms OR Cardiac Aneurysm OR Aneurysm, Cardiac OR Aneurysms, Cardiac OR Cardiac Aneurysms OR Enlarged Heart OR Heart, Enlarged OR Heart Enlargement OR Enlargement, Heart OR Cardiac Hypertrophy OR Cardiac Hypertrophies OR Hypertrophies, Cardiac OR Hypertrophy, Cardiac OR Heart Hypertrophy OR Heart Hypertrophies OR Hypertrophies, Heart OR Hypertrophy, Heart OR Arrest, Heart OR

	Asystole OR Asystoles OR Cardiac Arrest OR Arrest, Cardiac OR Cardiopulmonary Arrest OR Arrest, Cardiopulmonary OR Defect, Congenital Heart OR Abnormality, Heart OR Heart Abnormality OR Congenital Heart Defect OR Congenital Heart Defects OR Defects, Congenital Heart OR Heart Abnormalities OR Heart Defect, Congenital OR Congenital Heart Disease OR Congenital Heart Diseases OR Disease, Congenital Heart OR Heart Disease, Congenital OR Heart, Malformation Of OR Malformation Of Heart OR Malformation Of Hearts OR Cardiac Failure OR Heart Decompensation OR Decompensation, Heart OR Congestive Heart Failure OR Heart Failure, Congestive OR Heart Failure, Right-Sided OR Heart Failure, Right Sided OR Right-Sided Heart Failure OR Right Sided Heart Failure OR Heart Failure, Left-Sided OR Heart Failure, Left Sided OR Left-Sided Heart Failure OR Left Sided Heart Failure OR Myocardial Failure OR Heart Neoplasm OR Neoplasm, Heart OR Cardiac Neoplasms OR Cardiac Neoplasm OR Neoplasm, Cardiac OR Cardiac Tumor OR Tumor, Cardiac OR Tumors, Cardiac OR Cardiac Tumors OR Neoplasms, Cardiac OR Neoplasms, Heart OR Cardiac Carcinoma OR Carcinoma, Cardiac OR Carcinomas, Cardiac OR Cardiac Carcinomas OR Cardiac Cancer OR Cancer, Cardiac OR Cancers, Cardiac OR Cardiac Cancers OR Heart Cancer OR Cancer, Heart OR Cancers, Heart OR Heart Cancers OR Heart Tumor OR Heart Tumors OR Tumor, Heart OR Tumors, Heart OR Intracavitary Tumors of the Heart OR Primary Cardiac Tumors, Childhood OR Myocardial Tumors (Rhabdomyomas and Fibromas) OR Myocardial Tumor (Rhabdomyomas and Fibromas) OR Tumor, Myocardial (Rhabdomyomas and Fibromas) OR Tumors, Myocardial (Rhabdomyomas and Fibromas) OR Cardiac Tamponades OR Tamponade, Cardiac OR Tamponades, Cardiac OR Pericardial Tamponade OR Pericardial Tamponades OR Tamponade, Pericardial OR Tamponades, Pericardial OR Output, Low Cardiac OR Low Cardiac Output OR Low Cardiac Output Syndrome OR Cardiovascular Diseases OR Cardiovascular Abnormalities OR Heart Defects, Congenital OR Vascular Malformations OR Cardiovascular Infections OR Endocarditis, Bacterial OR Syphilis, Cardiovascular OR Tuberculosis, Cardiovascular OR Heart Diseases OR Arrhythmias, Cardiac OR Carcinoid Heart Disease OR Cardiac Conduction System Disease OR Cardiac Output, High OR Cardiac Output, Low OR Cardiac Tamponade OR Cardiomegaly OR Cardiomyopathies OR Cardiotoxicity OR Endocarditis OR Heart Aneurysm OR Heart Arrest OR Heart Defects, Congenital OR Heart Failure OR

	Heart Neoplasms OR Heart Rupture OR Heart Valve Diseases OR Myocardial Ischemia OR Myocardial Stunning OR Pericardial Effusion OR Pericarditis OR Pneumopericardium OR Post-Cardiac Arrest Syndrome OR Postpericardiotomy Syndrome OR Pulmonary Heart Disease OR Rheumatic Heart Disease OR Ventricular Dysfunction OR Ventricular Outflow Obstruction OR Pregnancy Complications, Cardiovascular OR Embolism, Amniotic Fluid OR Vascular Diseases OR Aneurysm OR Aneurysm, False OR Angiodysplasia OR Angioedema OR Angiomatosis OR Aortic Diseases OR Arterial Occlusive Diseases OR Arteriovenous Malformations OR Capillary Leak Syndrome OR Cerebrovascular Disorders OR Colitis, Ischemic OR Compartment Syndromes OR Diabetic Angiopathies OR Embolism and Thrombosis OR Hand-Arm Vibration Syndrome OR Hemorrhoids OR Hemostatic Disorders OR Hepatic Veno-Occlusive Disease OR Hyperemia OR Hypertension OR Hypotension OR Mesenteric Ischemia OR Myocardial Ischemia OR Optic Neuropathy, Ischemic OR Peliosis Hepatis OR Penetrating Atherosclerotic Ulcer OR Peripheral Vascular Diseases OR Prehypertension OR Pulmonary Veno-Occlusive Disease OR Reperfusion Injury OR Retinal Vein Occlusion OR Scimitar Syndrome OR Spinal Cord Vascular Diseases OR Splenic Infarction OR "Stenosis, Pulmonary Vein" OR Superior Vena Cava Syndrome OR Telangiectasis OR Thoracic Outlet Syndrome OR Varicocele OR Varicose Veins OR Vascular Fistula OR Vascular Neoplasms OR Vascular System Injuries OR Vasculitis OR Vasoplegia OR Venous Insufficiency) AND TITLE-ABS-KEY ((Gallstones OR Biliary Calculi OR Calculi, Biliary OR Gall Stone OR Gall Stones OR Gallstone OR Common Bile Duct Calculi OR Biliary Calculi, Common Bile Duct OR Common Bile Duct Gall Stones OR Common Bile Duct Gall Stone OR Common Bile Duct Gallstones OR Gall Stones, Common Bile Duct OR Common Bile Duct Gallstone OR Gallstones, Common Bile Duct))
EMBASE	(Cardiovascular Disease OR Disease, Cardiovascular OR Cardiac Events OR Cardiac Event OR Event, Cardiac OR Adverse Cardiac Event OR Adverse Cardiac Events OR Cardiac Event, Adverse OR Cardiac Events, Adverse OR Major Adverse Cardiac Events OR Abnormalities, Cardiovascular OR Abnormality, Cardiovascular OR Cardiovascular Abnormality OR Defect, Congenital Heart OR Abnormality, Heart OR Heart Abnormality OR Congenital Heart Defect OR Congenital Heart Defects OR Defects, Congenital Heart

	OR Heart Abnormalities OR Heart Defect, Congenital OR Congenital Heart Disease OR Congenital Heart Diseases OR Disease, Congenital Heart OR Heart Disease, Congenital OR Heart, Malformation Of OR Malformation Of Heart OR Malformation Of Hearts OR Malformations, Vascular OR Malformation, Vascular OR Vascular Malformation OR Cardiovascular Infection OR Infection, Cardiovascular OR Infections, Cardiovascular OR Bacterial Endocarditides OR Bacterial Endocarditis OR Endocarditides, Bacterial OR Cardiovascular Syphilis OR Aortitis, Syphilitic OR Aortitides, Syphilitic OR Syphilitic Aortitides OR Syphilitic Aortitis OR Heart Disease OR Cardiac Disorders OR Cardiac Disorder OR Heart Disorders OR Heart Disorder OR Cardiac Diseases OR Cardiac Disease OR Arrhythmia, Cardiac OR Arrhythmia OR Arrythmia OR Cardiac Arrhythmia OR Cardiac Arrhythmias OR Cardiac Dysrhythmia OR Dysrhythmia, Cardiac OR Carcinoid Heart Diseases OR Heart Disease, Carcinoid OR Heart Diseases, Carcinoid OR Cardiac Conduction System Diseases OR Cardiac Conduction Defects OR Conduction Defect, Cardiac OR Defect, Cardiac Conduction OR Cardiac Conduction Defect OR Post Cardiac Arrest Syndrome OR Post-Cardiac Arrest Syndromes OR Postcardiac Arrest Syndrome OR Postcardiac Arrest Syndromes OR Postresuscitation Disease OR Postresuscitation Diseases OR Postpericardiotomy Syndromes OR Syndrome, Postpericardiotomy OR Syndromes, Postpericardiotomy OR Postcommissurotomy Syndrome OR Postcommissurotomy Syndromes OR Syndrome, Postcommissurotomy OR Syndromes, Postcommissurotomy OR Cor Pulmonale OR Disease, Pulmonary Heart OR Diseases, Pulmonary Heart OR Heart Disease, Pulmonary OR Heart Diseases, Pulmonary OR Pulmonary Heart Diseases OR Disease, Rheumatic Heart OR Diseases, Rheumatic Heart OR Heart Disease, Rheumatic OR Heart Diseases, Rheumatic OR Rheumatic Heart Diseases OR Bouillaud Disease OR Disease, Bouillaud OR Bouillaud's Disease OR Bouillauds Disease OR Disease, Bouillaud's OR Dysfunctions, Ventricular OR Dysfunction, Ventricular OR Ventricular Dysfunctions OR Obstruction, Ventricular Outflow OR Outflow Obstruction, Ventricular OR Ventricular Outflow Obstructions OR Cardiovascular Pregnancy Complications OR Cardiovascular Pregnancy Complication OR Complication, Cardiovascular Pregnancy OR Pregnancy Complication, Cardiovascular OR Complications, Cardiovascular Pregnancy OR Pregnancy, Cardiovascular Complications OR Pregnancies, Cardiovascular Complications OR Amniotic Fluid Embolisms OR Embolisms, Amniotic Fluid OR Amniotic Fluid

	Embolism OR Diseases, Vascular OR Disease, Vascular OR Vascular Disease OR Aneurysms OR Fusiform Aneurysm OR Aneurysm, Fusiform OR Aneurysms, Fusiform OR Fusiform Aneurysms OR Saccular Aneurysm OR Aneurysms, False OR False Aneurysms OR Pseudoaneurysm OR Pseudoaneurysms OR False Aneurysm OR Angiodysplasias OR Angioedemas OR Urticaria, Giant OR Giant Urticaria OR Giant Urticarias OR Urticarias, Giant OR Angioneurotic Edema OR Angioneurotic Edemas OR Edema, Angioneurotic OR Edemas, Angioneurotic OR Quincke's Edema OR Edema, Quincke's OR Quincke Edema OR Quinckes Edema OR Angiomatoses OR Aortic Disease OR Disease, Aortic OR Diseases, Aortic OR Arterial Occlusive Disease OR Disease, Arterial Occlusive OR Occlusive Disease, Arterial OR Arterial Obstructive Diseases OR Arterial Obstructive Disease OR Disease, Arterial Obstructive OR Obstructive Disease, Arterial OR Arterial Occlusion OR Arterial Occlusions OR Occlusion, Arterial OR Arteriovenous Malformation OR Malformation, Arteriovenous OR Malformations, Arteriovenous OR Capillary Leak Syndromes OR Clarkson Disease OR Systemic Capillary Leak Syndrome OR Clinical Capillary Leak Syndrome OR Cerebrovascular Disorder OR Brain Vascular Disorders OR Brain Vascular Disorder OR Vascular Disorder, Brain OR Vascular Disorders, Brain OR Intracranial Vascular Disorders OR Intracranial Vascular Disorder OR Vascular Disorder, Intracranial OR Vascular Disorders, Intracranial OR Vascular Diseases, Intracranial OR Intracranial Vascular Disease OR Intracranial Vascular Diseases OR Vascular Disease, Intracranial OR Cerebrovascular Diseases OR Cerebrovascular Disease OR Disease, Cerebrovascular OR Diseases, Cerebrovascular OR Cerebrovascular Insufficiency OR Cerebrovascular Insufficiencies OR Insufficiencies, Cerebrovascular OR Insufficiency, Cerebrovascular OR Cerebrovascular Occlusion OR Cerebrovascular Occlusions OR Occlusion, Cerebrovascular OR Occlusions, Cerebrovascular OR Ischemic Colitis OR Compartment Syndrome OR Angiopathies, Diabetic OR Angiopathy, Diabetic OR Diabetic Angiopathy OR Diabetic Vascular Complications OR Diabetic Vascular Complication OR Vascular Complication, Diabetic OR Vascular Complications, Diabetic OR Diabetic Vascular Diseases OR Diabetic Vascular Disease OR Vascular Disease, Diabetic OR Vascular Diseases, Diabetic OR Microangiopathy, Diabetic OR Diabetic Microangiopathies OR Diabetic Microangiopathy OR Microangiopathies, Diabetic OR Thrombosis and Embolism OR Hand Arm Vibration Syndrome OR Hand-Arm Vibration Syndromes OR

	Syndrome, Hand-Arm Vibration OR Syndromes, Hand-Arm Vibration OR Vibration Syndrome, Hand-Arm OR Vibration Syndromes, Hand-Arm OR Effusion, Pericardial OR Effusions, Pericardial OR Pericardial Effusions OR Hemopericardium OR Chylopericardium OR Chylopericardiums OR Pericarditis OR Pleuropericarditis OR Heart Ruptures OR Cardiac Rupture OR Cardiac Ruptures OR Free Wall Rupture, Heart OR Cardiac Free Wall Rupture OR Ventricular Free Wall Rupture OR Heart Valve Disease OR Valve Disease, Heart OR Valvular Heart Diseases OR Heart Disease, Valvular OR Valvular Heart Disease OR Heart Valvular Disease OR Disease, Heart Valvular OR Heart Valvular Diseases OR Valvular Disease, Heart OR Heart Disease, Ischemic OR Disease, Ischemic Heart OR Diseases, Ischemic Heart OR Heart Diseases, Ischemic OR Ischemic Heart Diseases OR Ischemia, Myocardial OR Ischemias, Myocardial OR Myocardial Ischemias OR Ischemic Heart Disease Stunning, Myocardial OR Stunned Myocardium OR Myocardium, Stunned OR Hibernation, Myocardial OR Myocardial Hibernation OR Disease, Hepatic Veno-Occlusive OR Hepatic Veno-Occlusive Diseases OR Hepatic Veno Occlusive Disease OR Veno-Occlusive Disease, Hepatic OR Veno Occlusive Disease, Hepatic OR Sinusoidal Obstruction Syndrome OR Syndrome, Sinusoidal Obstruction OR Blood Pressure, High OR Blood Pressures, High OR High Blood Pressure OR High Blood Pressures OR Blood Pressure, Low OR Hypotension, Vascular OR Low Blood Pressure OR Vascular Hypotension OR Ischemia, Mesenteric OR Ischemias, Mesenteric OR Mesenteric Ischemias OR Mesenteric Vascular Insufficiency OR Insufficiencies, Mesenteric Vascular OR Insufficiency, Mesenteric Vascular OR Mesenteric Vascular Insufficiencies OR Vascular Insufficiencies, Mesenteric OR Vascular Insufficiency, Mesenteric OR Occlusive Mesenteric Arterial Ischemia OR Acute Mesenteric Arterial Embolus OR Nonocclusive Mesenteric Ischemia OR Ischemia, Nonocclusive Mesenteric OR Ischemias, Nonocclusive Mesenteric OR Mesenteric Ischemia, Nonocclusive OR Mesenteric Ischemias, Nonocclusive OR Nonocclusive Mesenteric Ischemias OR Acute Mesenteric Arterial Thrombosis OR Mesenteric Venous Thrombosis OR Mesenteric Venous Thromboses OR Thromboses, Mesenteric Venous OR Thrombosis, Mesenteric Venous OR Venous Thromboses, Mesenteric OR Venous Thrombosis, Mesenteric OR Heart Disease, Ischemic OR Disease, Ischemic Heart OR Diseases, Ischemic Heart OR Heart Diseases, Ischemic OR Ischemic Heart Diseases OR Ischemia, Myocardial OR Ischemias, Myocardial OR

	Myocardial Ischemias OR Ischemic Heart Disease OR Myocardial Ischemia OR Optic Ischaemic Neuropathy OR Ischaemic Neuropathy, Optic OR Neuropathy, Optic Ischaemic OR Optic Ischaemic Neuropathies OR Optic Ischemic Neuropathy OR Ischemic Neuropathy, Optic OR Neuropathy, Optic Ischemic OR Optic Ischemic Neuropathies OR Ischemic Optic Neuropathy OR Ischemic Optic Neuropathies OR Neuropathy, Ischemic Optic OR Optic Nerve Ischemia OR Ischemia, Optic Nerve OR Nerve Ischemia, Optic OR Optic Nerve Ischemias OR Anterior Ischemic Optic Neuropathy OR Optic Neuropathy, Anterior Ischemic OR Posterior Ischemic Optic Neuropathy OR Optic Neuropathy, Posterior Ischemic OR Nonarteritic Anterior Ischemic Optic Neuropathy OR Hepatis, Peliosis OR Atherosclerotic Ulcer, Penetrating OR Penetrating Atherosclerotic Ulcers OR Ulcer, Penetrating Atherosclerotic OR Penetrating Aortic Ulcer OR Aortic Ulcer, Penetrating OR Penetrating Aortic Ulcers OR Ulcer, Penetrating Aortic OR Penetrating Ulcer Aorta OR Penetrating Ulcer Aortas OR Aortic Penetrating Ulcer OR Aortic Penetrating Ulcers OR Penetrating Ulcer, Aortic OR Ulcer, Aortic Penetrating OR Penetrating Ulcer OR Penetrating Ulcers OR Ulcer, Penetrating OR Disease, Peripheral Vascular OR Peripheral Vascular Disease OR Vascular Disease, Peripheral OR Diseases, Peripheral Vascular OR Peripheral Angiopathies OR Angiopathies, Peripheral OR Angiopathy, Peripheral OR Peripheral Angiopathy OR Vascular Diseases, Peripheral OR Pre-Hypertension OR Pre Hypertension OR Diseases, Pulmonary Veno-Occlusive OR Pulmonary Veno-Occlusive Diseases OR Veno-Occlusive Diseases, Pulmonary OR Pulmonary Veno Occlusive Disease OR Veno-Occlusive Disease, Pulmonary OR Veno Occlusive Disease, Pulmonary OR Pulmonary Venoocclusive Disease OR Pulmonary Venoocclusive Diseases OR Venoocclusive Disease, Pulmonary OR Venoocclusive Diseases, Pulmonary OR Reperfusion Injuries OR Injury, Ischemia-Reperfusion OR Injury, Ischemia Reperfusion OR Ischemia-Reperfusion Injuries OR Injury, Reperfusion OR Ischemia-Reperfusion Injury OR Ischemia Reperfusion Injury OR Reperfusion Damage OR Damage, Reperfusion OR Reperfusion Damages OR Occlusion, Retinal Vein OR Retinal Vein Occlusions OR Vein Occlusion, Retinal OR Retinal Vein Thrombosis OR Retinal Vein Thromboses OR Vein Thrombosis, Retinal OR Thrombosis, Retinal Vein OR Branch Vein Occlusion OR Branch Vein Occlusions OR Occlusion, Branch Vein OR Vein Occlusion, Branch OR Branch Retinal Vein Occlusion OR Retinal Branch Vein Occlusion OR Central

	Retinal Vein Occlusion OR Scimitar Anomaly OR Anomaly, Scimitar OR Total Anomalous Pulmonary Venous Return OR Total Anomalous Pulmonary Venous Return OR Anomalous Pulmonary Venous Return OR Pulmonary Venous Return Anomaly OR Vascular Diseases, Spinal Cord OR Hematomyelia OR Posterior Spinal Artery Syndrome OR Infarction, Splenic OR Infarctions, Splenic OR Splenic Infarctions OR Splenic Infarct OR Infarct, Splenic OR Infarcts, Splenic OR Splenic Infarcts OR Infarct of the Spleen OR Pulmonary Vein Stenoses OR Pulmonary Vein Stenosis OR Stenoses, Pulmonary Vein OR Vein Stenoses, Pulmonary OR Vein Stenosis, Pulmonary OR SVC Syndrome OR SVC Syndromes OR Syndrome, SVC OR Obstruction of the Superior Vena Cava OR SVC Obstruction OR Obstruction, SVC OR SVC Obstructions OR Superior Vena Cava Obstruction OR Superior Vena Cava Thrombosis OR SVC Thrombosis OR SVC Thromboses OR Thrombosis, SVC OR Telangiectases OR Telangiectasia OR Telangiectasias OR Spider Veins OR Spider Vein OR Vein, Spider OR Veins, Spider OR Outlet Syndromes, Thoracic OR Outlet Syndrome, Thoracic OR Syndromes, Thoracic Outlet OR Syndrome, Thoracic Outlet OR Thoracic Outlet Syndromes OR Aperture Syndrome, Thoracic Outlet OR Neurovascular Syndrome, Thoracic Outlet OR Superior Thoracic Aperture Syndrome OR Thoracic Outlet Neurovascular Syndrome OR Scalenus Anticus Syndrome OR Syndrome, Scalenus Anticus OR Costoclavicular Syndrome OR Costoclavicular Syndromes OR Syndrome, Costoclavicular OR Syndromes, Costoclavicular OR Thoracic Outlet Nerve Compression Syndrome OR Nerve Compression Syndrome, Thoracic Outlet OR Neurogenic Thoracic Outlet Syndrome OR Thoracic Outlet Syndrome, Neurogenic OR Neurologic Syndrome, Thoracic Outlet OR Thoracic Outlet Neurologic Syndrome OR Arterial Thoracic Outlet Syndrome OR Venous Thoracic Outlet Syndrome OR Varicoceles OR Varicose Vein OR Veins, Varicose OR Vein, Varicose OR Varices OR Varix OR Fistulas, Vascular OR Fistula, Vascular OR Vascular Fistulas OR Neoplasms, Vascular OR Neoplasm, Vascular OR Vascular Neoplasm OR Injuries, Vascular System OR Injury, Vascular System OR System Injuries, Vascular OR System Injury, Vascular OR Vascular System Injury OR Vascular Injuries OR Injuries, Vascular OR Injury, Vascular OR Vascular Injury OR Vasculitides OR Angiitis OR Angiitides OR Vasoplegias OR Postoperative Vasoplegic Syndrome OR Post-operative Vasoplegic Syndrome OR Post operative Vasoplegic Syndrome OR Post-operative Vasoplegic

	Syndromes OR Syndrome, Post-operative Vasoplegic OR Syndromes, Post-operative Vasoplegic OR Vasoplegic Syndrome, Post-operative OR Vasoplegic Syndromes, Post-operative OR Vasoplegic Syndrome OR Insufficiencies, Venous OR Insufficiency, Venous OR Venous Insufficiencies OR Hyperemias OR Venous Engorgement OR Engorgement, Venous OR Passive Hyperemia OR Hyperemia, Passive OR Venous Congestion OR Congestion, Venous OR Active Hyperemia OR Hyperemia, Active OR Arterial Hyperemia OR Hyperemia, Arterial OR Reactive Hyperemia OR Hyperemia, Reactive OR Hyperemias, Reactive OR Reactive Hyperemias OR Hemostatic Disorder OR Hemostatic Disorders, Vascular OR Disorders, Vascular Hemostatic OR Disorder, Vascular Hemostatic OR Hemostatic Disorder, Vascular OR Vascular Hemostatic Disorder OR Vascular Hemostatic Disorders OR Endocarditides OR Infective Endocarditis OR Endocarditides, Infective OR Endocarditis, Infective OR Infective Endocarditides OR Cardiomyopathy OR Myocardial Disease OR Disease, Myocardial OR Diseases, Myocardial OR Myocardial Diseases OR Myocardiopathies OR Myocardiopathy OR Cardiomyopathies, Primary OR Cardiomyopathy, Primary OR Primary Cardiomyopathies OR Primary Cardiomyopathy OR Primary Myocardial Disease OR Disease, Primary Myocardial OR Diseases, Primary Myocardial OR Myocardial Disease, Primary OR Myocardial Diseases, Primary OR Primary Myocardial Diseases OR Cardiomyopathies, Secondary OR Cardiomyopathy, Secondary OR Secondary Cardiomyopathies OR Secondary Cardiomyopathy OR Myocardial Diseases, Secondary OR Disease, Secondary Myocardial OR Diseases, Secondary Myocardial OR Myocardial Disease, Secondary OR Secondary Myocardial Disease OR Secondary Myocardial Diseases OR Cardiotoxicities OR Cardiac Toxicity OR Cardiac Toxicities OR Toxicity, Cardiac OR Aneurysm, Heart OR Aneurysms, Heart OR Heart Aneurysms OR Cardiac Aneurysm OR Aneurysm, Cardiac OR Aneurysms, Cardiac OR Cardiac Aneurysms OR Enlarged Heart OR Heart, Enlarged OR Heart Enlargement OR Enlargement, Heart OR Cardiac Hypertrophy OR Cardiac Hypertrophies OR Hypertrophies, Cardiac OR Hypertrophy, Cardiac OR Heart Hypertrophy OR Heart Hypertrophies OR Hypertrophies, Heart OR Hypertrophy, Heart OR Arrest, Heart OR Asystole OR Asystoles OR Cardiac Arrest OR Arrest, Cardiac OR Cardiopulmonary Arrest OR Arrest, Cardiopulmonary OR Defect, Congenital Heart OR Abnormality, Heart OR Heart Abnormality OR Congenital Heart Defect OR Congenital Heart Defects OR Defects,

	Congenital Heart OR Heart Abnormalities OR Heart Defect, Congenital OR Congenital Heart Disease OR Congenital Heart Diseases OR Disease, Congenital Heart OR Heart Disease, Congenital OR Heart, Malformation Of OR Malformation Of Heart OR Malformation Of Hearts OR Cardiac Failure OR Heart Decompensation OR Decompensation, Heart OR Congestive Heart Failure OR Heart Failure, Congestive OR Heart Failure, Right-Sided OR Heart Failure, Right Sided OR Right-Sided Heart Failure OR Right Sided Heart Failure OR Heart Failure, Left-Sided OR Heart Failure, Left Sided OR Left-Sided Heart Failure OR Left Sided Heart Failure OR Myocardial Failure OR Heart Neoplasm OR Neoplasm, Heart OR Cardiac Neoplasms OR Cardiac Neoplasm OR Neoplasm, Cardiac OR Cardiac Tumor OR Tumor, Cardiac OR Tumors, Cardiac OR Cardiac Tumors OR Neoplasms, Cardiac OR Neoplasms, Heart OR Cardiac Carcinoma OR Carcinoma, Cardiac OR Carcinomas, Cardiac OR Cardiac Carcinomas OR Cardiac Cancer OR Cancer, Cardiac OR Cancers, Cardiac OR Cardiac Cancers OR Heart Cancer OR Cancer, Heart OR Cancers, Heart OR Heart Cancers OR Heart Tumor OR Heart Tumors OR Tumor, Heart OR Tumors, Heart OR Intracavitary Tumors of the Heart OR Primary Cardiac Tumors, Childhood OR Myocardial Tumors (Rhabdomyomas and Fibromas) OR Myocardial Tumor (Rhabdomyomas and Fibromas) OR Tumor, Myocardial (Rhabdomyomas and Fibromas) OR Tumors, Myocardial (Rhabdomyomas and Fibromas) OR Cardiac Tamponades OR Tamponade, Cardiac OR Tamponades, Cardiac OR Pericardial Tamponade OR Pericardial Tamponades OR Tamponade, Pericardial OR Tamponades, Pericardial OR Output, Low Cardiac OR Low Cardiac Output OR Low Cardiac Output Syndrome OR Cardiovascular Diseases OR Cardiovascular Abnormalities OR Heart Defects, Congenital OR Vascular Malformations OR Cardiovascular Infections OR Endocarditis, Bacterial OR Syphilis, Cardiovascular OR Tuberculosis, Cardiovascular OR Heart Diseases OR Arrhythmias, Cardiac OR Carcinoid Heart Disease OR Cardiac Conduction System Disease OR Cardiac Output, High OR Cardiac Output, Low OR Cardiac Tamponade OR Cardiomegaly OR Cardiomyopathies OR Cardiotoxicity OR Endocarditis OR Heart Aneurysm OR Heart Arrest OR Heart Defects, Congenital OR Heart Failure OR Heart Neoplasms OR Heart Rupture OR Heart Valve Diseases OR Myocardial Ischemia OR Myocardial Stunning OR Pericardial Effusion OR Pericarditis OR Pneumopericardium OR Post-Cardiac Arrest Syndrome OR Postpericardiotomy Syndrome OR Pulmonary Heart

	Disease OR Rheumatic Heart Disease OR Ventricular Dysfunction OR Ventricular Outflow Obstruction OR Pregnancy Complications, Cardiovascular OR Embolism, Amniotic Fluid OR Vascular Diseases OR Aneurysm OR Aneurysm, False OR Angiodysplasia OR Angioedema OR Angiomatosis OR Aortic Diseases OR Arterial Occlusive Diseases OR Arteriovenous Malformations OR Capillary Leak Syndrome OR Cerebrovascular Disorders OR Colitis, Ischemic OR Compartment Syndromes OR Diabetic Angiopathies OR Embolism and Thrombosis OR Hand-Arm Vibration Syndrome OR Hemorrhoids OR Hemostatic Disorders OR Hepatic Veno-Occlusive Disease OR Hyperemia OR Hypertension OR Hypotension OR Mesenteric Ischemia OR Myocardial Ischemia OR Optic Neuropathy, Ischemic OR Peliosis Hepatis OR Penetrating Atherosclerotic Ulcer OR Peripheral Vascular Diseases OR Prehypertension OR Pulmonary Veno-Occlusive Disease OR Reperfusion Injury OR Retinal Vein Occlusion OR Scimitar Syndrome OR Spinal Cord Vascular Diseases OR Splenic Infarction OR "Stenosis, Pulmonary Vein" OR Superior Vena Cava Syndrome OR Telangiectasis OR Thoracic Outlet Syndrome OR Varicocele OR Varicose Veins OR Vascular Fistula OR Vascular Neoplasms OR Vascular System Injuries OR Vasculitis OR Vasoplegia OR Venous Insufficiency) AND (Gallstones OR Biliary Calculi OR Calculi, Biliary OR Gall Stone OR Gall Stones OR Gallstone OR Common Bile Duct Calculi OR Biliary Calculi, Common Bile Duct OR Common Bile Duct Gall Stones OR Common Bile Duct Gall Stone OR Common Bile Duct Gallstones OR Gall Stones, Common Bile Duct OR Common Bile Duct Gallstone OR Gallstones, Common Bile Duct))
Cochrane Library	(("Cardiovascular Disease " OR " Disease, Cardiovascular " OR " Cardiac Events " OR " Cardiac Event " OR " Event, Cardiac " OR " Adverse Cardiac Event " OR " Adverse Cardiac Events " OR " Cardiac Event, Adverse " OR " Cardiac Events, Adverse " OR " Maj" OR " Adverse Cardiac Events " OR " Abn" OR "malities, Cardiovascular " OR " Abn" OR "mality, Cardiovascular " OR " Cardiovascular Abn" OR "mality " OR " Defect, Congenital Heart " OR " Abn" OR "mality, Heart " OR " Heart Abn" OR "mality " OR " Congenital Heart Defect " OR " Congenital Heart Defects " OR " Defects, Congenital Heart " OR " Heart Abn" OR "malities " OR " Heart Defect, Congenital " OR " Congenital Heart Disease " OR " Congenital Heart Diseases " OR " Disease, Congenital Heart " OR " Heart Disease, Congenital " OR " Heart, Malf" OR "mation Of " OR " Malf" OR "mation Of Heart " OR " Malf" OR "mation Of Hearts " OR " Malf" OR "mations, Vascular " OR " Malf" OR "mation, Vascular " OR " Vascular Malf" OR "mation " OR " Cardiovascular Infection " OR " Infection, Cardiovascular " OR " Infections, Cardiovascular " OR " Bacterial Endocarditides " OR " Bacterial Endocarditis " OR " Endocarditides, Bacterial " OR " Cardiovascular Syphilis " OR " A" OR "titis, Syphilitic " OR " A" OR "titides, Syphilitic "

	OR “ Syphilitic A” OR “titides “ OR “ Syphilitic A” OR “titis “ OR “ Heart Disease “ OR “ Cardiac Dis” OR “ders “ OR “ Cardiac Dis” OR “der “ OR “ Heart Dis” OR “ders “ OR “ Heart Dis” OR “der “ OR “ Cardiac Diseases “ OR “ Cardiac Disease “ OR “ Arrhythmia, Cardiac “ OR “ Arrhythmia “ OR “ Arrythmia “ OR “ Cardiac Arrhythmia “ OR “ Cardiac Arrhythmias “ OR “ Cardiac Dysrhythmia “ OR “ Dysrhythmia, Cardiac “ OR “ Carcinoid Heart Diseases “ OR “ Heart Disease, Carcinoid “ OR “ Heart Diseases, Carcinoid “ OR “ Cardiac Conduction System Diseases “ OR “ Cardiac Conduction Defects “ OR “ Conduction Defect, Cardiac “ OR “ Defect, Cardiac Conduction “ OR “ Cardiac Conduction Defect “ OR “ Post Cardiac Arrest Syndrome “ OR “ Post-Cardiac Arrest Syndromes “ OR “ Postcardiac Arrest Syndrome “ OR “ Postcardiac Arrest Syndromes “ OR “ Postresuscitation Disease “ OR “ Postresuscitation Diseases “ OR “ Postpericardiotomy Syndromes “ OR “ Syndrome, Postpericardiotomy “ OR “ Syndromes, Postpericardiotomy “ OR “ Postcommissurotomy Syndrome “ OR “ Postcommissurotomy Syndromes “ OR “ Syndrome, Postcommissurotomy “ OR “ Syndromes, Postcommissurotomy “ OR “ C” OR “ Pulmonale “ OR “ Disease, Pulmonary Heart “ OR “ Diseases, Pulmonary Heart “ OR “ Heart Disease, Pulmonary “ OR “ Heart Diseases, Pulmonary “ OR “ Pulmonary Heart Diseases “ OR “ Disease, Rheumatic Heart “ OR “ Diseases, Rheumatic Heart “ OR “ Heart Disease, Rheumatic “ OR “ Heart Diseases, Rheumatic “ OR “ Rheumatic Heart Diseases “ OR “ Bouillaud Disease “ OR “ Disease, Bouillaud “ OR “ Bouillaud's Disease “ OR “ Bouillauds Disease “ OR “ Disease, Bouillaud's “ OR “ Dysfunctions, Ventricular “ OR “ Dysfunction, Ventricular “ OR “ Ventricular Dysfunctions “ OR “ Obstruction, Ventricular Outflow “ OR “ Outflow Obstruction, Ventricular “ OR “ Ventricular Outflow Obstructions “ OR “ Cardiovascular Pregnancy Complications “ OR “ Cardiovascular Pregnancy Complication “ OR “ Complication, Cardiovascular Pregnancy “ OR “ Pregnancy Complication, Cardiovascular “ OR “ Complications, Cardiovascular Pregnancy “ OR “ Pregnancy, Cardiovascular Complications “ OR “ Pregnancies, Cardiovascular Complications “ OR “ Amniotic Fluid Embolisms “ OR “ Embolisms, Amniotic Fluid “ OR “ Amniotic Fluid Embolism “ OR “ Diseases, Vascular “ OR “ Disease, Vascular “ OR “ Vascular Disease “ OR “ Aneurysms “ OR “ Fusif” OR “m Aneurysm “ OR “ Aneurysm, Fusif” OR “m “ OR “ Aneurysms, Fusif” OR “m “ OR “ Fusif” OR “m Aneurysms “ OR “ Saccular Aneurysm “ OR “ Aneurysms, False “ OR “ False Aneurysms “ OR “ Pseudoaneurysm “ OR “ Pseudoaneurysms “ OR “ False Aneurysm “ OR “ Angiodysplasias “ OR “ Angioedemas “ OR “ Urticaria, Giant “ OR “ Giant Urticaria “ OR “ Giant Urticarias “ OR “ Urticarias, Giant “ OR “ Angioneurotic Edema “ OR “ Angioneurotic Edemas “ OR “ Edema, Angioneurotic “ OR “ Edemas, Angioneurotic “ OR “ Quincke's Edema “ OR “ Edema, Quincke's “ OR “ Quincke Edema “ OR “ Quinckes Edema “ OR “ Angiomatoses “ OR “ A” OR “tic Disease “ OR “ Disease, A” OR “tic “ OR “ Diseases, A” OR “tic “ OR “ Arterial Occlusive Disease “ OR “ Disease, Arterial Occlusive “ OR “ Occlusive Disease, Arterial “ OR “ Arterial Obstructive Diseases “ OR “ Arterial Obstructive Disease “ OR “ Disease, Arterial Obstructive “ OR “ Obstructive Disease, Arterial “ OR “ Arterial Occlusion “ OR “ Arterial Occlusions “ OR “ Occlusion, Arterial “ OR “ Arteriovenous Malf” OR “mation “ OR “ Malf” OR “mation, Arteriovenous “ OR “ Malf” OR “mations, Arteriovenous “ OR “ Capillary Leak Syndromes “ OR “ Clarkson Disease “ OR “ Systemic

	Capillary Leak Syndrome " OR " Clinical Capillary Leak Syndrome " OR " Cerebrovascular Dis" OR "der " OR " Brain Vascular Dis" OR "ders " OR " Brain Vascular Dis" OR "der " OR " Vascular Dis" OR "der, Brain " OR " Vascular Dis" OR "ders, Brain " OR " Intracranial Vascular Dis" OR "ders " OR " Intracranial Vascular Dis" OR "der " OR " Vascular Dis" OR "der, Intracranial " OR " Vascular Dis" OR "ders, Intracranial " OR " Vascular Diseases, Intracranial " OR " Intracranial Vascular Disease " OR " Intracranial Vascular Diseases " OR " Vascular Disease, Intracranial " OR " Cerebrovascular Diseases " OR " Cerebrovascular Disease " OR " Disease, Cerebrovascular " OR " Diseases, Cerebrovascular " OR " Cerebrovascular Insufficiency " OR " Cerebrovascular Insufficiencies " OR " Insufficiencies, Cerebrovascular " OR " Insufficiency, Cerebrovascular " OR " Cerebrovascular Occlusion " OR " Cerebrovascular Occlusions " OR " Occlusion, Cerebrovascular " OR " Occlusions, Cerebrovascular " OR " Ischemic Colitis " OR " Compartment Syndrome " OR " Angiopathies, Diabetic " OR " Angiopathy, Diabetic " OR " Diabetic Angiopathy " OR " Diabetic Vascular Complications " OR " Diabetic Vascular Complication " OR " Vascular Complication, Diabetic " OR " Vascular Complications, Diabetic " OR " Diabetic Vascular Diseases " OR " Diabetic Vascular Disease " OR " Vascular Disease, Diabetic " OR " Vascular Diseases, Diabetic " OR " Microangiopathy, Diabetic " OR " Diabetic Microangiopathies " OR " Diabetic Microangiopathy " OR " Microangiopathies, Diabetic " OR " Thrombosis and Embolism " OR " Hand Arm Vibration Syndrome " OR " Hand-Arm Vibration Syndromes " OR " Syndrome, Hand-Arm Vibration " OR " Syndromes, Hand-Arm Vibration " OR " Vibration Syndrome, Hand-Arm " OR " Vibration Syndromes, Hand-Arm " OR " Effusion, Pericardial " OR " Effusions, Pericardial " OR " Pericardial Effusions " OR " Hemopericardium " OR " Chylopericardium " OR " Chylopericardiums " OR " Pericarditis " OR " Pleuropericarditis " OR " Heart Ruptures " OR " Cardiac Rupture " OR " Cardiac Ruptures " OR " Free Wall Rupture, Heart " OR " Cardiac Free Wall Rupture " OR " Ventricular Free Wall Rupture " OR " Heart Valve Disease " OR " Valve Disease, Heart " OR " Valvular Heart Diseases " OR " Heart Disease, Valvular " OR " Valvular Heart Disease " OR " Heart Valvular Disease " OR " Disease, Heart Valvular " OR " Heart Valvular Diseases " OR " Valvular Disease, Heart " OR " Heart Disease, Ischemic " OR " Disease, Ischemic Heart " OR " Diseases, Ischemic Heart " OR " Heart Diseases, Ischemic " OR " Ischemic Heart Diseases " OR " Ischemia, Myocardial " OR " Ischemias, Myocardial " OR " Myocardial Ischemias " OR " Ischemic Heart Disease Stunning, Myocardial " OR " Stunned Myocardium " OR " Myocardium, Stunned " OR " Hibernation, Myocardial " OR " Myocardial Hibernation " OR " Disease, Hepatic Veno-Occlusive " OR " Hepatic Veno-Occlusive Diseases " OR " Hepatic Veno Occlusive Disease " OR " Veno-Occlusive Disease, Hepatic " OR " Veno Occlusive Disease, Hepatic " OR " Sinusoidal Obstruction Syndrome " OR " Syndrome, Sinusoidal Obstruction " OR " Blood Pressure, High " OR " Blood Pressures, High " OR " High Blood Pressure " OR " High Blood Pressures " OR " Blood Pressure, Low " OR " Hypotension, Vascular " OR " Low Blood Pressure " OR " Vascular Hypotension " OR " Ischemia, Mesenteric " OR " Ischemias, Mesenteric " OR " Mesenteric Ischemias " OR " Mesenteric Vascular Insufficiency " OR " Insufficiencies, Mesenteric Vascular " OR " Insufficiency, Mesenteric Vascular " OR " Mesenteric Vascular

	Insufficiencies “ OR “ Vascular Insufficiencies, Mesenteric “ OR “ Vascular Insufficiency, Mesenteric “ OR “ Occlusive Mesenteric Arterial Ischemia “ OR “ Acute Mesenteric Arterial Embolus “ OR “ Nonocclusive Mesenteric Ischemia “ OR “ Ischemia, Nonocclusive Mesenteric “ OR “ Ischemias, Nonocclusive Mesenteric “ OR “ Mesenteric Ischemia, Nonocclusive “ OR “ Mesenteric Ischemias, Nonocclusive “ OR “ Nonocclusive Mesenteric Ischemias “ OR “ Acute Mesenteric Arterial Thrombosis “ OR “ Mesenteric Venous Thrombosis “ OR “ Mesenteric Venous Thromboses “ OR “ Thromboses, Mesenteric Venous “ OR “ Thrombosis, Mesenteric Venous “ OR “ Venous Thromboses, Mesenteric “ OR “ Venous Thrombosis, Mesenteric “ OR “ Heart Disease, Ischemic “ OR “ Disease, Ischemic Heart “ OR “ Diseases, Ischemic Heart “ OR “ Heart Diseases, Ischemic “ OR “ Ischemic Heart Diseases “ OR “ Ischemia, Myocardial “ OR “ Ischemias, Myocardial “ OR “ Myocardial Ischemias “ OR “ Ischemic Heart Disease “ OR “ Myocardial Ischemia “ OR “ Optic Ischaemic Neuropathy “ OR “ Ischaemic Neuropathy, Optic “ OR “ Neuropathy, Optic Ischaemic “ OR “ Optic Ischaemic Neuropathies “ OR “ Optic Ischemic Neuropathy “ OR “ Ischemic Neuropathy, Optic “ OR “ Neuropathy, Optic Ischemic “ OR “ Optic Ischemic Neuropathies “ OR “ Ischemic Optic Neuropathy “ OR “ Ischemic Optic Neuropathies “ OR “ Neuropathy, Ischemic Optic “ OR “ Optic Nerve Ischemia “ OR “ Ischemia, Optic Nerve “ OR “ Nerve Ischemia, Optic “ OR “ Optic Nerve Ischemias “ OR “ Anteri” OR “ Ischemic Optic Neuropathy “ OR “ Optic Neuropathy, Anteri” OR “ Ischemic “ OR “ Posteri” OR “ Ischemic Optic Neuropathy “ OR “ Optic Neuropathy, Posteri” OR “ Ischemic “ OR “ Nonarteritic Anteri” OR “ Ischemic Optic Neuropathy “ OR “ Hepatis, Peliosis “ OR “ Atherosclerotic Ulcer, Penetrating “ OR “ Penetrating Atherosclerotic Ulcers “ OR “ Ulcer, Penetrating Atherosclerotic “ OR “ Penetrating A” OR “tic Ulcer “ OR “ A” OR “tic Ulcer, Penetrating “ OR “ Penetrating A” OR “tic Ulcers “ OR “ Ulcer, Penetrating A” OR “tic “ OR “ Penetrating Ulcer A” OR “ta “ OR “ Penetrating Ulcer A” OR “tas “ OR “ A” OR “tic Penetrating Ulcer “ OR “ A” OR “tic Penetrating Ulcers “ OR “ Penetrating Ulcer, A” OR “tic “ OR “ Ulcer, A” OR “tic Penetrating “ OR “ Penetrating Ulcer “ OR “ Penetrating Ulcers “ OR “ Ulcer, Penetrating “ OR “ Disease, Peripheral Vascular “ OR “ Peripheral Vascular Disease “ OR “ Vascular Disease, Peripheral “ OR “ Diseases, Peripheral Vascular “ OR “ Peripheral Angiopathies “ OR “ Angiopathies, Peripheral “ OR “ Angiopathy, Peripheral “ OR “ Peripheral Angiopathy “ OR “ Vascular Diseases, Peripheral “ OR “ Pre-Hypertension “ OR “ Pre Hypertension “ OR “ Diseases, Pulmonary Veno-Occlusive “ OR “ Pulmonary Veno-Occlusive Diseases “ OR “ Veno-Occlusive Diseases, Pulmonary “ OR “ Pulmonary Veno Occlusive Disease “ OR “ Veno-Occlusive Disease, Pulmonary “ OR “ Veno Occlusive Disease, Pulmonary “ OR “ Pulmonary Venoocclusive Disease “ OR “ Pulmonary Venoocclusive Diseases “ OR “ Venoocclusive Disease, Pulmonary “ OR “ Venoocclusive Diseases, Pulmonary “ OR “ Reperfusion Injuries “ OR “ Injury, Ischemia-Reperfusion “ OR “ Injury, Ischemia Reperfusion “ OR “ Ischemia-Reperfusion Injuries “ OR “ Injury, Reperfusion “ OR “ Ischemia-Reperfusion Injury “ OR “ Ischemia Reperfusion Injury “ OR “ Reperfusion Damage “ OR “ Damage, Reperfusion “ OR “ Reperfusion Damages “ OR “ Occlusion, Retinal Vein “ OR “ Retinal Vein Occlusions “ OR “ Vein Occlusion, Retinal “ OR “ Retinal Vein Thrombosis “ OR “ Retinal Vein Thromboses “ OR “ Vein Thrombosis, Retinal “ OR

	“ Thrombosis, Retinal Vein “ OR “ Branch Vein Occlusion “ OR “ Branch Vein Occlusions “ OR “ Occlusion, Branch Vein “ OR “ Vein Occlusion, Branch “ OR “ Branch Retinal Vein Occlusion “ OR “ Retinal Branch Vein Occlusion “ OR “ Central Retinal Vein Occlusion “ OR “ Scimitar Anomaly “ OR “ Anomaly, Scimitar “ OR “ Total Anomalous Pulmonary Venous Return “ OR “ Total Anomalous Pulmonary Venous Return “ OR “ Anomalous Pulmonary Venous Return “ OR “ Pulmonary Venous Return Anomaly “ OR “ Vascular Diseases, Spinal C” OR “d “ OR “ Hematomyelia “ OR “ Posteri” OR “ Spinal Artery Syndrome “ OR “ Infarction, Splenic “ OR “ Infarctions, Splenic “ OR “ Splenic Infarctions “ OR “ Splenic Infarct “ OR “ Infarct, Splenic “ OR “ Infarcts, Splenic “ OR “ Splenic Infarcts “ OR “ Infarct of the Spleen “ OR “ Pulmonary Vein Stenoses “ OR “ Pulmonary Vein Stenosis “ OR “ Stenoses, Pulmonary Vein “ OR “ Vein Stenoses, Pulmonary “ OR “ Vein Stenosis, Pulmonary “ OR “ SVC Syndrome “ OR “ SVC Syndromes “ OR “ Syndrome, SVC “ OR “ Obstruction of the Superi” OR “ Vena Cava “ OR “ SVC Obstruction “ OR “ Obstruction, SVC “ OR “ SVC Obstructions “ OR “ Superi” OR “ Vena Cava Obstruction “ OR “ Superi” OR “ Vena Cava Thrombosis “ OR “ SVC Thrombosis “ OR “ SVC Thromboses “ OR “ Thrombosis, SVC “ OR “ Telangiectases “ OR “ Telangiectasia “ OR “ Telangiectasias “ OR “ Spider Veins “ OR “ Spider Vein “ OR “ Vein, Spider “ OR “ Veins, Spider “ OR “ Outlet Syndromes, Th” OR “acic “ OR “ Outlet Syndrome, Th” OR “acic “ OR “ Syndromes, Th” OR “acic Outlet “ OR “ Syndrome, Th” OR “acic Outlet “ OR “ Th” OR “acic Outlet Syndromes “ OR “ Aperture Syndrome, Th” OR “acic Outlet “ OR “ Neurovascular Syndrome, Th” OR “acic Outlet “ OR “ Superi” OR “ Th” OR “acic Aperture Syndrome “ OR “ Th” OR “acic Outlet Neurovascular Syndrome “ OR “ Scalenus Anticus Syndrome “ OR “ Syndrome, Scalenus Anticus “ OR “ Costoclavicular Syndrome “ OR “ Costoclavicular Syndromes “ OR “ Syndrome, Costoclavicular “ OR “ Syndromes, Costoclavicular “ OR “ Th” OR “acic Outlet Nerve Compression Syndrome “ OR “ Nerve Compression Syndrome, Th” OR “acic Outlet “ OR “ Neurogenic Th” OR “acic Outlet Syndrome “ OR “ Th” OR “acic Outlet Syndrome, Neurogenic “ OR “ Neurologic Syndrome, Th” OR “acic Outlet “ OR “ Th” OR “acic Outlet Neurologic Syndrome “ OR “ Arterial Th” OR “acic Outlet Syndrome “ OR “ Venous Th” OR “acic Outlet Syndrome “ OR “ Varicoceles “ OR “ Varicose Vein “ OR “ Veins, Varicose “ OR “ Vein, Varicose “ OR “ Varices “ OR “ Varix “ OR “ Fistulas, Vascular “ OR “ Fistula, Vascular “ OR “ Vascular Fistulas “ OR “ Neoplasms, Vascular “ OR “ Neoplasm, Vascular “ OR “ Vascular Neoplasm “ OR “ Injuries, Vascular System “ OR “ Injury, Vascular System “ OR “ System Injuries, Vascular “ OR “ System Injury, Vascular “ OR “ Vascular System Injury “ OR “ Vascular Injuries “ OR “ Injuries, Vascular “ OR “ Injury, Vascular “ OR “ Vascular Injury “ OR “ Vasculitides “ OR “ Angiitis “ OR “ Angiitides “ OR “ Vasoplegias “ OR “ Postoperative Vasoplegic Syndrome “ OR “ Post-operative Vasoplegic Syndrome “ OR “ Post operative Vasoplegic Syndrome “ OR “ Post-operative Vasoplegic Syndromes “ OR “ Syndrome, Post-operative Vasoplegic “ OR “ Syndromes, Post-operative Vasoplegic “ OR “ Vasoplegic Syndrome, Post-operative “ OR “ Vasoplegic Syndromes, Post-operative “ OR “ Vasoplegic Syndrome “ OR “ Insufficiencies, Venous “ OR “ Insufficiency, Venous “ OR “ Venous Insufficiencies “ OR “ Hyperemias “ OR “ Venous Eng” OR “gement “ OR “ Eng” OR “gement, Venous “ OR “ Passive Hyperemia “

	OR “ Hyperemia, Passive “ OR “ Venous Congestion “ OR “ Congestion, Venous “ OR “ Active Hyperemia “ OR “ Hyperemia, Active “ OR “ Arterial Hyperemia “ OR “ Hyperemia, Arterial “ OR “ Reactive Hyperemia “ OR “ Hyperemia, Reactive “ OR “ Hyperemias, Reactive “ OR “ Reactive Hyperemias “ OR “ Hemostatic Dis” OR “der “ OR “ Hemostatic Dis” OR “ders, Vascular “ OR “ Dis” OR “ders, Vascular Hemostatic “ OR “ Dis” OR “der, Vascular Hemostatic “ OR “ Hemostatic Dis” OR “der, Vascular “ OR “ Vascular Hemostatic Dis” OR “der “ OR “ Vascular Hemostatic Dis” OR “ders “ OR “ Endocarditides “ OR “ Infective Endocarditis “ OR “ Endocarditides, Infective “ OR “ Endocarditis, Infective “ OR “ Infective Endocarditides “ OR “ Cardiomyopathy “ OR “ Myocardial Disease “ OR “ Disease, Myocardial “ OR “ Diseases, Myocardial “ OR “ Myocardial Diseases “ OR “ Myocardiopathies “ OR “ Myocardiopathy “ OR “ Cardiomyopathies, Primary “ OR “ Cardiomyopathy, Primary “ OR “ Primary Cardiomyopathies “ OR “ Primary Cardiomyopathy “ OR “ Primary Myocardial Disease “ OR “ Disease, Primary Myocardial “ OR “ Diseases, Primary Myocardial “ OR “ Myocardial Disease, Primary “ OR “ Myocardial Diseases, Primary “ OR “ Primary Myocardial Diseases “ OR “ Cardiomyopathies, Secondary “ OR “ Cardiomyopathy, Secondary “ OR “ Secondary Cardiomyopathies “ OR “ Secondary Cardiomyopathy “ OR “ Myocardial Diseases, Secondary “ OR “ Disease, Secondary Myocardial “ OR “ Diseases, Secondary Myocardial “ OR “ Myocardial Disease, Secondary “ OR “ Secondary Myocardial Disease “ OR “ Secondary Myocardial Diseases “ OR “ Cardiotoxicities “ OR “ Cardiac Toxicity “ OR “ Cardiac Toxicities “ OR “ Toxicity, Cardiac “ OR “ Aneurysm, Heart “ OR “ Aneurysms, Heart “ OR “ Heart Aneurysms “ OR “ Cardiac Aneurysm “ OR “ Aneurysm, Cardiac “ OR “ Aneurysms, Cardiac “ OR “ Cardiac Aneurysms “ OR “ Enlarged Heart “ OR “ Heart, Enlarged “ OR “ Heart Enlargement “ OR “ Enlargement, Heart “ OR “ Cardiac Hypertrophy “ OR “ Cardiac Hypertrophies “ OR “ Hypertrophies, Cardiac “ OR “ Hypertrophy, Cardiac “ OR “ Heart Hypertrophy “ OR “ Heart Hypertrophies “ OR “ Hypertrophies, Heart “ OR “ Hypertrophy, Heart “ OR “ Arrest, Heart “ OR “ Asystole “ OR “ Asystoles “ OR “ Cardiac Arrest “ OR “ Arrest, Cardiac “ OR “ Cardiopulmonary Arrest “ OR “ Arrest, Cardiopulmonary “ OR “ Defect, Congenital Heart “ OR “ Abn” OR “mality, Heart “ OR “ Heart Abn” OR “mality “ OR “ Congenital Heart Defect “ OR “ Congenital Heart Defects “ OR “ Defects, Congenital Heart “ OR “ Heart Abn” OR “malities “ OR “ Heart Defect, Congenital “ OR “ Congenital Heart Disease “ OR “ Congenital Heart Diseases “ OR “ Disease, Congenital Heart “ OR “ Heart Disease, Congenital “ OR “ Heart, Malf” OR “mation Of “ OR “ Malf” OR “mation Of Heart “ OR “ Malf” OR “mation Of Hearts “ OR “ Cardiac Failure “ OR “ Heart Decompensation “ OR “ Decompensation, Heart “ OR “ Congestive Heart Failure “ OR “ Heart Failure, Congestive “ OR “ Heart Failure, Right-Sided “ OR “ Heart Failure, Right Sided “ OR “ Right-Sided Heart Failure “ OR “ Right Sided Heart Failure “ OR “ Heart Failure, Left-Sided “ OR “ Heart Failure, Left Sided “ OR “ Left-Sided Heart Failure “ OR “ Left Sided Heart Failure “ OR “ Myocardial Failure “ OR “ Heart Neoplasm “ OR “ Neoplasm, Heart “ OR “ Cardiac Neoplasms “ OR “ Cardiac Neoplasm “ OR “ Neoplasm, Cardiac “ OR “ Cardiac Tum” OR “ “ OR “ Tum” OR “, Cardiac “ OR “ Tum” OR “s, Cardiac “ OR “ Cardiac Tum” OR “s “ OR “ Neoplasms, Cardiac “ OR “ Neoplasms, Heart “ OR “ Cardiac Carcinoma “ OR “ Carcinoma, Cardiac “

	OR “ Carcinomas, Cardiac “ OR “ Cardiac Carcinomas “ OR “ Cardiac Cancer “ OR “ Cancer, Cardiac “ OR “ Cancers, Cardiac “ OR “ Cardiac Cancers “ OR “ Heart Cancer “ OR “ Cancer, Heart “ OR “ Cancers, Heart “ OR “ Heart Cancers “ OR “ Heart Tum” OR “ “ OR “ Heart Tum” OR “s “ OR “ Tum” OR “, Heart “ OR “ Tum” OR “s, Heart “ OR “ Intracavitary Tum” OR “s of the Heart “ OR “ Primary Cardiac Tum” OR “s, Childhood “ OR “ Myocardial Tum” OR “s (Rhabdomyomas and Fibromas) “ OR “ Myocardial Tum” OR “ (Rhabdomyomas and Fibromas) “ OR “ Tum” OR “, Myocardial (Rhabdomyomas and Fibromas) “ OR “ Tum” OR “s, Myocardial (Rhabdomyomas and Fibromas) “ OR “ Cardiac Tamponades “ OR “ Tamponade, Cardiac “ OR “ Tamponades, Cardiac “ OR “ Pericardial Tamponade “ OR “ Pericardial Tamponades “ OR “ Tamponade, Pericardial “ OR “ Tamponades, Pericardial “ OR “ Output, Low Cardiac “ OR “ Low Cardiac Output “ OR “ Low Cardiac Output Syndrome “ OR “ Cardiovascular Diseases “ OR “ Cardiovascular Abn” OR “malities “ OR “ Heart Defects, Congenital “ OR “ Vascular Malf” OR “mations “ OR “ Cardiovascular Infections “ OR “ Endocarditis, Bacterial “ OR “ Syphilis, Cardiovascular “ OR “ Tuberculosis, Cardiovascular “ OR “ Heart Diseases “ OR “ Arrhythmias, Cardiac “ OR “ Carcinoid Heart Disease “ OR “ Cardiac Conduction System Disease “ OR “ Cardiac Output, High “ OR “ Cardiac Output, Low “ OR “ Cardiac Tamponade “ OR “ Cardiomegaly “ OR “ Cardiomyopathies “ OR “ Cardiotoxicity “ OR “ Endocarditis “ OR “ Heart Aneurysm “ OR “ Heart Arrest “ OR “ Heart Defects, Congenital “ OR “ Heart Failure “ OR “ Heart Neoplasms “ OR “ Heart Rupture “ OR “ Heart Valve Diseases “ OR “ Myocardial Ischemia “ OR “ Myocardial Stunning “ OR “ Pericardial Effusion “ OR “ Pericarditis “ OR “ Pneumopericardium “ OR “ Post-Cardiac Arrest Syndrome “ OR “ Postpericardiotomy Syndrome “ OR “ Pulmonary Heart Disease “ OR “ Rheumatic Heart Disease “ OR “ Ventricular Dysfunction “ OR “ Ventricular Outflow Obstruction “ OR “ Pregnancy Complications, Cardiovascular “ OR “ Embolism, Amniotic Fluid “ OR “ Vascular Diseases “ OR “ Aneurysm “ OR “ Aneurysm, False “ OR “ Angiodysplasia “ OR “ Angioedema “ OR “ Angiomatosis “ OR “ A” OR “tic Diseases “ OR “ Arterial Occlusive Diseases “ OR “ Arteriovenous Malf” OR “mations “ OR “ Capillary Leak Syndrome “ OR “ Cerebrovascular Dis” OR “ders “ OR “ Colitis, Ischemic “ OR “ Compartment Syndromes “ OR “ Diabetic Angiopathies “ OR “ Embolism and Thrombosis “ OR “ Hand-Arm Vibration Syndrome “ OR “ Hem” OR “rhoids “ OR “ Hemostatic Dis” OR “ders “ OR “ Hepatic Veno-Occlusive Disease “ OR “ Hyperemia “ OR “ Hypertension “ OR “ Hypotension “ OR “ Mesenteric Ischemia “ OR “ Myocardial Ischemia “ OR “ Optic Neuropathy, Ischemic “ OR “ Peliosis Hepatis “ OR “ Penetrating Atherosclerotic Ulcer “ OR “ Peripheral Vascular Diseases “ OR “ Prehypertension “ OR “ Pulmonary Veno-Occlusive Disease “ OR “ Reperfusion Injury “ OR “ Retinal Vein Occlusion “ OR “ Scimitar Syndrome “ OR “ Spinal C” OR “d Vascular Diseases “ OR “ Splenic Infarction “ OR “ “Stenosis, Pulmonary Vein” “ OR “ “Superi” OR “ Vena Cava Syndrome “ OR “ Telangiectasis “ OR “ Th” OR “acic Outlet Syndrome “ OR “ Varicocele “ OR “ Varicose Veins “ OR “ Vascular Fistula “ OR “ Vascular Neoplasms “ OR “ Vascular System Injuries “ OR “ Vasculitis “ OR “ Vasoplegia “ OR “ Venous Insufficiency”) in Title Abstract Keyword AND (“Gallstones “ OR “ Biliary Calculi “ OR “ Calculi, Biliary “ OR “ Gall Stone “ OR “ Gall Stones “ OR “ Gallstone “ OR “ Common Bile Duct Calculi “ OR

	" Biliary Calculi, Common Bile Duct " OR " Common Bile Duct Gall Stones " OR " Common Bile Duct Gall Stone " OR " Common Bile Duct Gallstones " OR " Gall Stones, Common Bile Duct " OR " Common Bile Duct Gallstone " OR " Gallstones, Common Bile Duct")) in Title Abstract Keyword
Google Scholar	(Gallstones OR "Biliary Calculi" OR "Gall Stone" OR "Gall Stones" OR Gallstone OR "Common Bile Duct Calculi") AND (Cardiovascular Diseases OR Cardiovascular Infections OR Heart Diseases OR Vascular Diseases)<br>The first 500 articles were considered.
Web Of Science	Cardiovascular Disease OR Disease, Cardiovascular OR Cardiac Events OR Cardiac Event OR Event, Cardiac OR Adverse Cardiac Event OR Adverse Cardiac Events OR Cardiac Event, Adverse OR Cardiac Events, Adverse OR Major Adverse Cardiac Events OR Abnormalities, Cardiovascular OR Abnormality, Cardiovascular OR Cardiovascular Abnormality OR Defect, Congenital Heart OR Abnormality, Heart OR Heart Abnormality OR Congenital Heart Defect OR Congenital Heart Defects OR Defects, Congenital Heart OR Heart Abnormalities OR Heart Defect, Congenital OR Congenital Heart Disease OR Congenital Heart Diseases OR Disease, Congenital Heart OR Heart Disease, Congenital OR Heart, Malformation Of OR Malformation Of Heart OR Malformation Of Hearts OR Malformations, Vascular OR Malformation, Vascular OR Vascular Malformation OR Cardiovascular Infection OR Infection, Cardiovascular OR Infections, Cardiovascular OR Bacterial Endocarditides OR Bacterial Endocarditis OR Endocarditides, Bacterial OR Cardiovascular Syphilis OR Aortitis, Syphilitic OR Aortitides, Syphilitic OR Syphilitic Aortitides OR Syphilitic Aortitis OR Heart Disease OR Cardiac Disorders OR Cardiac Disorder OR Heart Disorders OR Heart Disorder OR Cardiac Diseases OR Cardiac Disease OR Arrhythmia, Cardiac OR Arrhythmia OR Arrythmia OR Cardiac Arrhythmia OR Cardiac Arrhythmias OR Cardiac Dysrhythmia OR Dysrhythmia, Cardiac OR Carcinoid Heart Diseases OR Heart Disease, Carcinoid OR Heart Diseases, Carcinoid OR Cardiac Conduction System Diseases OR Cardiac Conduction Defects OR Conduction Defect, Cardiac OR Defect, Cardiac Conduction OR Cardiac Conduction Defect OR Post Cardiac Arrest Syndrome OR Post-Cardiac Arrest Syndromes OR Postcardiac Arrest Syndrome OR Postcardiac Arrest Syndromes OR Postresuscitation Disease OR Postresuscitation Diseases OR Postpericardiotomy Syndromes OR Syndrome, Postpericardiotomy OR Syndromes,

	Postpericardiotomy OR Postcommissurotomy Syndrome OR Postcommissurotomy Syndromes OR Syndrome, Postcommissurotomy OR Syndromes, Postcommissurotomy OR Cor Pulmonale OR Disease, Pulmonary Heart OR Diseases, Pulmonary Heart OR Heart Disease, Pulmonary OR Heart Diseases, Pulmonary OR Pulmonary Heart Diseases OR Disease, Rheumatic Heart OR Diseases, Rheumatic Heart OR Heart Disease, Rheumatic OR Heart Diseases, Rheumatic OR Rheumatic Heart Diseases OR Bouillaud Disease OR Disease, Bouillaud OR Bouillaud's Disease OR Bouillauds Disease OR Disease, Bouillaud's OR Dysfunctions, Ventricular OR Dysfunction, Ventricular OR Ventricular Dysfunctions OR Obstruction, Ventricular Outflow OR Outflow Obstruction, Ventricular OR Ventricular Outflow Obstructions OR Cardiovascular Pregnancy Complications OR Cardiovascular Pregnancy Complication OR Complication, Cardiovascular Pregnancy OR Pregnancy Complication, Cardiovascular OR Complications, Cardiovascular Pregnancy OR Pregnancy, Cardiovascular Complications OR Pregnancies, Cardiovascular Complications OR Amniotic Fluid Embolisms OR Embolisms, Amniotic Fluid OR Amniotic Fluid Embolism OR Diseases, Vascular OR Disease, Vascular OR Vascular Disease OR Aneurysms OR Fusiform Aneurysm OR Aneurysm, Fusiform OR Aneurysms, Fusiform OR Fusiform Aneurysms OR Saccular Aneurysm OR Aneurysms, False OR False Aneurysms OR Pseudoaneurysm OR Pseudoaneurysms OR False Aneurysm OR Angiodysplasias OR Angioedemas OR Urticaria, Giant OR Giant Urticaria OR Giant Urticarias OR Urticarias, Giant OR Angioneurotic Edema OR Angioneurotic Edemas OR Edema, Angioneurotic OR Edemas, Angioneurotic OR Quincke's Edema OR Edema, Quincke's OR Quincke Edema OR Quinckes Edema OR Angiomatoses OR Aortic Disease OR Disease, Aortic OR Diseases, Aortic OR Arterial Occlusive Disease OR Disease, Arterial Occlusive OR Occlusive Disease, Arterial OR Arterial Obstructive Diseases OR Arterial Obstructive Disease OR Disease, Arterial Obstructive OR Obstructive Disease, Arterial OR Arterial Occlusion OR Arterial Occlusions OR Occlusion, Arterial OR Arteriovenous Malformation OR Malformation, Arteriovenous OR Malformations, Arteriovenous OR Capillary Leak Syndromes OR Clarkson Disease OR Systemic Capillary Leak Syndrome OR Clinical Capillary Leak Syndrome OR Cerebrovascular Disorder OR Brain Vascular Disorders OR Brain Vascular Disorder OR Vascular Disorder, Brain OR Vascular Disorders, Brain OR Intracranial Vascular Disorders OR Intracranial Vascular Disorder OR Vascular Disorder,

	Intracranial OR Vascular Disorders, Intracranial OR Vascular Diseases, Intracranial OR Intracranial Vascular Disease OR Intracranial Vascular Diseases OR Vascular Disease, Intracranial OR Cerebrovascular Diseases OR Cerebrovascular Disease OR Disease, Cerebrovascular OR Diseases, Cerebrovascular OR Cerebrovascular Insufficiency OR Cerebrovascular Insufficiencies OR Insufficiencies, Cerebrovascular OR Insufficiency, Cerebrovascular OR Cerebrovascular Occlusion OR Cerebrovascular Occlusions OR Occlusion, Cerebrovascular OR Occlusions, Cerebrovascular OR Ischemic Colitis OR Compartment Syndrome OR Angiopathies, Diabetic OR Angiopathy, Diabetic OR Diabetic Angiopathy OR Diabetic Vascular Complications OR Diabetic Vascular Complication OR Vascular Complication, Diabetic OR Vascular Complications, Diabetic OR Diabetic Vascular Diseases OR Diabetic Vascular Disease OR Vascular Disease, Diabetic OR Vascular Diseases, Diabetic OR Microangiopathy, Diabetic OR Diabetic Microangiopathies OR Diabetic Microangiopathy OR Microangiopathies, Diabetic OR Thrombosis and Embolism OR Hand Arm Vibration Syndrome OR Hand-Arm Vibration Syndromes OR Syndrome, Hand-Arm Vibration OR Syndromes, Hand-Arm Vibration OR Vibration Syndrome, Hand-Arm OR Vibration Syndromes, Hand-Arm OR Effusion, Pericardial OR Effusions, Pericardial OR Pericardial Effusions OR Hemopericardium OR Chylopericardium OR Chylopericardiums OR Pericarditis OR Pleuropericarditis OR Heart Ruptures OR Cardiac Rupture OR Cardiac Ruptures OR Free Wall Rupture, Heart OR Cardiac Free Wall Rupture OR Ventricular Free Wall Rupture OR Heart Valve Disease OR Valve Disease, Heart OR Valvular Heart Diseases OR Heart Disease, Valvular OR Valvular Heart Disease OR Heart Valvular Disease OR Disease, Heart Valvular OR Heart Valvular Diseases OR Valvular Disease, Heart OR Heart Disease, Ischemic OR Disease, Ischemic Heart OR Diseases, Ischemic Heart OR Heart Diseases, Ischemic OR Ischemic Heart Diseases OR Ischemia, Myocardial OR Ischemias, Myocardial OR Myocardial Ischemias OR Ischemic Heart Disease Stunning, Myocardial OR Stunned Myocardium OR Myocardium, Stunned OR Hibernation, Myocardial OR Myocardial Hibernation OR Disease, Hepatic Veno-Occlusive OR Hepatic Veno-Occlusive Diseases OR Hepatic Veno Occlusive Disease OR Veno-Occlusive Disease, Hepatic OR Veno Occlusive Disease, Hepatic OR Sinusoidal Obstruction Syndrome OR Syndrome, Sinusoidal Obstruction OR Blood Pressure, High OR Blood Pressures, High OR High Blood Pressure OR High Blood

	Pressures OR Blood Pressure, Low OR Hypotension, Vascular OR Low Blood Pressure OR Vascular Hypotension OR Ischemia, Mesenteric OR Ischemias, Mesenteric OR Mesenteric Ischemias OR Mesenteric Vascular Insufficiency OR Insufficiencies, Mesenteric Vascular OR Insufficiency, Mesenteric Vascular OR Mesenteric Vascular Insufficiencies OR Vascular Insufficiencies, Mesenteric OR Vascular Insufficiency, Mesenteric OR Occlusive Mesenteric Arterial Ischemia OR Acute Mesenteric Arterial Embolus OR Nonocclusive Mesenteric Ischemia OR Ischemia, Nonocclusive Mesenteric OR Ischemias, Nonocclusive Mesenteric OR Mesenteric Ischemia, Nonocclusive OR Mesenteric Ischemias, Nonocclusive OR Nonocclusive Mesenteric Ischemias OR Acute Mesenteric Arterial Thrombosis OR Mesenteric Venous Thrombosis OR Mesenteric Venous Thromboses OR Thromboses, Mesenteric Venous OR Thrombosis, Mesenteric Venous OR Venous Thromboses, Mesenteric OR Venous Thrombosis, Mesenteric OR Heart Disease, Ischemic OR Disease, Ischemic Heart OR Diseases, Ischemic Heart OR Heart Diseases, Ischemic OR Ischemic Heart Diseases OR Ischemia, Myocardial OR Ischemias, Myocardial OR Myocardial Ischemias OR Ischemic Heart Disease OR Myocardial Ischemia OR Optic Ischaemic Neuropathy OR Ischaemic Neuropathy, Optic OR Neuropathy, Optic Ischaemic OR Optic Ischaemic Neuropathies OR Optic Ischemic Neuropathy OR Ischemic Neuropathy, Optic OR Neuropathy, Optic Ischemic OR Optic Ischemic Neuropathies OR Ischemic Optic Neuropathy OR Ischemic Optic Neuropathies OR Neuropathy, Ischemic Optic OR Optic Nerve Ischemia OR Ischemia, Optic Nerve OR Nerve Ischemia, Optic OR Optic Nerve Ischemias OR Anterior Ischemic Optic Neuropathy OR Optic Neuropathy, Anterior Ischemic OR Posterior Ischemic Optic Neuropathy OR Optic Neuropathy, Posterior Ischemic OR Nonarteritic Anterior Ischemic Optic Neuropathy OR Hepatis, Peliosis OR Atherosclerotic Ulcer, Penetrating OR Penetrating Atherosclerotic Ulcers OR Ulcer, Penetrating Atherosclerotic OR Penetrating Aortic Ulcer OR Aortic Ulcer, Penetrating OR Penetrating Aortic Ulcers OR Ulcer, Penetrating Aortic OR Penetrating Ulcer Aorta OR Penetrating Ulcer Aortas OR Aortic Penetrating Ulcer OR Aortic Penetrating Ulcers OR Penetrating Ulcer, Aortic OR Ulcer, Aortic Penetrating OR Penetrating Ulcer OR Penetrating Ulcers OR Ulcer, Penetrating OR Disease, Peripheral Vascular OR Peripheral Vascular Disease OR Vascular Disease, Peripheral OR Diseases, Peripheral Vascular OR Peripheral Angiopathies OR Angiopathies, Peripheral OR

	Angiopathy, Peripheral OR Peripheral Angiopathy OR Vascular Diseases, Peripheral OR Pre-Hypertension OR Pre Hypertension OR Diseases, Pulmonary Veno-Occlusive OR Pulmonary Veno-Occlusive Diseases OR Veno-Occlusive Diseases, Pulmonary OR Pulmonary Veno Occlusive Disease OR Veno-Occlusive Disease, Pulmonary OR Veno Occlusive Disease, Pulmonary OR Pulmonary Venoocclusive Disease OR Pulmonary Venoocclusive Diseases OR Venoocclusive Disease, Pulmonary OR Venoocclusive Diseases, Pulmonary OR Reperfusion Injuries OR Injury, Ischemia-Reperfusion OR Injury, Ischemia Reperfusion OR Ischemia-Reperfusion Injuries OR Injury, Reperfusion OR Ischemia-Reperfusion Injury OR Ischemia Reperfusion Injury OR Reperfusion Damage OR Damage, Reperfusion OR Reperfusion Damages OR Occlusion, Retinal Vein OR Retinal Vein Occlusions OR Vein Occlusion, Retinal OR Retinal Vein Thrombosis OR Retinal Vein Thromboses OR Vein Thrombosis, Retinal OR Thrombosis, Retinal Vein OR Branch Vein Occlusion OR Branch Vein Occlusions OR Occlusion, Branch Vein OR Vein Occlusion, Branch OR Branch Retinal Vein Occlusion OR Retinal Branch Vein Occlusion OR Central Retinal Vein Occlusion OR Scimitar Anomaly OR Anomaly, Scimitar OR Total Anomalous Pulmonary Venous Return OR Total Anomalous Pulmonary Venous Return OR Anomalous Pulmonary Venous Return OR Pulmonary Venous Return Anomaly OR Vascular Diseases, Spinal Cord OR Hematomyelia OR Posterior Spinal Artery Syndrome OR Infarction, Splenic OR Infarctions, Splenic OR Splenic Infarctions OR Splenic Infarct OR Infarct, Splenic OR Infarcts, Splenic OR Splenic Infarcts OR Infarct of the Spleen OR Pulmonary Vein Stenoses OR Pulmonary Vein Stenosis OR Stenoses, Pulmonary Vein OR Vein Stenoses, Pulmonary OR Vein Stenosis, Pulmonary OR SVC Syndrome OR SVC Syndromes OR Syndrome, SVC OR Obstruction of the Superior Vena Cava OR SVC Obstruction OR Obstruction, SVC OR SVC Obstructions OR Superior Vena Cava Obstruction OR Superior Vena Cava Thrombosis OR SVC Thrombosis OR SVC Thromboses OR Thrombosis, SVC OR Telangiectases OR Telangiectasia OR Telangiectasias OR Spider Veins OR Spider Vein OR Vein, Spider OR Veins, Spider OR Outlet Syndromes, Thoracic OR Outlet Syndrome, Thoracic OR Syndromes, Thoracic Outlet OR Syndrome, Thoracic Outlet OR Thoracic Outlet Syndromes OR Aperture Syndrome, Thoracic Outlet OR Neurovascular Syndrome, Thoracic Outlet OR Superior Thoracic Aperture Syndrome OR Thoracic Outlet Neurovascular Syndrome OR Scalenus

	Anticus Syndrome OR Syndrome, Scalenus Anticus OR Costoclavicular Syndrome OR Costoclavicular Syndromes OR Syndrome, Costoclavicular OR Syndromes, Costoclavicular OR Thoracic Outlet Nerve Compression Syndrome OR Nerve Compression Syndrome, Thoracic Outlet OR Neurogenic Thoracic Outlet Syndrome OR Thoracic Outlet Syndrome, Neurogenic OR Neurologic Syndrome, Thoracic Outlet OR Thoracic Outlet Neurologic Syndrome OR Arterial Thoracic Outlet Syndrome OR Venous Thoracic Outlet Syndrome OR Varicoceles OR Varicose Vein OR Veins, Varicose OR Vein, Varicose OR Varices OR Varix OR Fistulas, Vascular OR Fistula, Vascular OR Vascular Fistulas OR Neoplasms, Vascular OR Neoplasm, Vascular OR Vascular Neoplasm OR Injuries, Vascular System OR Injury, Vascular System OR System Injuries, Vascular OR System Injury, Vascular OR Vascular System Injury OR Vascular Injuries OR Injuries, Vascular OR Injury, Vascular OR Vascular Injury OR Vasculitides OR Angiitis OR Angiitides OR Vasoplegias OR Postoperative Vasoplegic Syndrome OR Post-operative Vasoplegic Syndrome OR Post operative Vasoplegic Syndrome OR Post-operative Vasoplegic Syndromes OR Syndrome, Post-operative Vasoplegic OR Syndromes, Post-operative Vasoplegic OR Vasoplegic Syndrome, Post-operative OR Vasoplegic Syndromes, Post-operative OR Vasoplegic Syndrome OR Insufficiencies, Venous OR Insufficiency, Venous OR Venous Insufficiencies OR Hyperemias OR Venous Engorgement OR Engorgement, Venous OR Passive Hyperemia OR Hyperemia, Passive OR Venous Congestion OR Congestion, Venous OR Active Hyperemia OR Hyperemia, Active OR Arterial Hyperemia OR Hyperemia, Arterial OR Reactive Hyperemia OR Hyperemia, Reactive OR Hyperemias, Reactive OR Reactive Hyperemias OR Hemostatic Disorder OR Hemostatic Disorders, Vascular OR Disorders, Vascular Hemostatic OR Disorder, Vascular Hemostatic OR Hemostatic Disorder, Vascular OR Vascular Hemostatic Disorder OR Vascular Hemostatic Disorders OR Endocarditides OR Infective Endocarditis OR Endocarditides, Infective OR Endocarditis, Infective OR Infective Endocarditides OR Cardiomyopathy OR Myocardial Disease OR Disease, Myocardial OR Diseases, Myocardial OR Myocardial Diseases OR Myocardiopathies OR Myocardiopathy OR Cardiomyopathies, Primary OR Cardiomyopathy, Primary OR Primary Cardiomyopathies OR Primary Cardiomyopathy OR Primary Myocardial Disease OR Disease, Primary Myocardial OR Diseases, Primary Myocardial OR Myocardial Disease, Primary OR Myocardial Diseases, Primary OR

	Primary Myocardial Diseases OR Cardiomyopathies, Secondary OR Cardiomyopathy, Secondary OR Secondary Cardiomyopathies OR Secondary Cardiomyopathy OR Myocardial Diseases, Secondary OR Disease, Secondary Myocardial OR Diseases, Secondary Myocardial OR Myocardial Disease, Secondary OR Secondary Myocardial Disease OR Secondary Myocardial Diseases OR Cardiotoxicities OR Cardiac Toxicity OR Cardiac Toxicities OR Toxicity, Cardiac OR Aneurysm, Heart OR Aneurysms, Heart OR Heart Aneurysms OR Cardiac Aneurysm OR Aneurysm, Cardiac OR Aneurysms, Cardiac OR Cardiac Aneurysms OR Enlarged Heart OR Heart, Enlarged OR Heart Enlargement OR Enlargement, Heart OR Cardiac Hypertrophy OR Cardiac Hypertrophies OR Hypertrophies, Cardiac OR Hypertrophy, Cardiac OR Heart Hypertrophy OR Heart Hypertrophies OR Hypertrophies, Heart OR Hypertrophy, Heart OR Arrest, Heart OR Asystole OR Asystoles OR Cardiac Arrest OR Arrest, Cardiac OR Cardiopulmonary Arrest OR Arrest, Cardiopulmonary OR Defect, Congenital Heart OR Abnormality, Heart OR Heart Abnormality OR Congenital Heart Defect OR Congenital Heart Defects OR Defects, Congenital Heart OR Heart Abnormalities OR Heart Defect, Congenital OR Congenital Heart Disease OR Congenital Heart Diseases OR Disease, Congenital Heart OR Heart Disease, Congenital OR Heart, Malformation Of OR Malformation Of Heart OR Malformation Of Hearts OR Cardiac Failure OR Heart Decompensation OR Decompensation, Heart OR Congestive Heart Failure OR Heart Failure, Congestive OR Heart Failure, Right-Sided OR Heart Failure, Right Sided OR Right-Sided Heart Failure OR Right Sided Heart Failure OR Heart Failure, Left-Sided OR Heart Failure, Left Sided OR Left-Sided Heart Failure OR Left Sided Heart Failure OR Myocardial Failure OR Heart Neoplasm OR Neoplasm, Heart OR Cardiac Neoplasms OR Cardiac Neoplasm OR Neoplasm, Cardiac OR Cardiac Tumor OR Tumor, Cardiac OR Tumors, Cardiac OR Cardiac Tumors OR Neoplasms, Cardiac OR Neoplasms, Heart OR Cardiac Carcinoma OR Carcinoma, Cardiac OR Carcinomas, Cardiac OR Cardiac Carcinomas OR Cardiac Cancer OR Cancer, Cardiac OR Cancers, Cardiac OR Cardiac Cancers OR Heart Cancer OR Cancer, Heart OR Cancers, Heart OR Heart Cancers OR Heart Tumor OR Heart Tumors OR Tumor, Heart OR Tumors, Heart OR Intracavitary Tumors of the Heart OR Primary Cardiac Tumors, Childhood OR Myocardial Tumors (Rhabdomyomas and Fibromas) OR Myocardial Tumor (Rhabdomyomas and Fibromas) OR Tumor, Myocardial

	(Rhabdomyomas and Fibromas) OR Tumors, Myocardial (Rhabdomyomas and Fibromas) OR Cardiac Tamponades OR Tamponade, Cardiac OR Tamponades, Cardiac OR Pericardial Tamponade OR Pericardial Tamponades OR Tamponade, Pericardial OR Tamponades, Pericardial OR Output, Low Cardiac OR Low Cardiac Output OR Low Cardiac Output Syndrome OR Cardiovascular Diseases OR Cardiovascular Abnormalities OR Heart Defects, Congenital OR Vascular Malformations OR Cardiovascular Infections OR Endocarditis, Bacterial OR Syphilis, Cardiovascular OR Tuberculosis, Cardiovascular OR Heart Diseases OR Arrhythmias, Cardiac OR Carcinoid Heart Disease OR Cardiac Conduction System Disease OR Cardiac Output, High OR Cardiac Output, Low OR Cardiac Tamponade OR Cardiomegaly OR Cardiomyopathies OR Cardiotoxicity OR Endocarditis OR Heart Aneurysm OR Heart Arrest OR Heart Defects, Congenital OR Heart Failure OR Heart Neoplasms OR Heart Rupture OR Heart Valve Diseases OR Myocardial Ischemia OR Myocardial Stunning OR Pericardial Effusion OR Pericarditis OR Pneumopericardium OR Post-Cardiac Arrest Syndrome OR Postpericardiotomy Syndrome OR Pulmonary Heart Disease OR Rheumatic Heart Disease OR Ventricular Dysfunction OR Ventricular Outflow Obstruction OR Pregnancy Complications, Cardiovascular OR Embolism, Amniotic Fluid OR Vascular Diseases OR Aneurysm OR Aneurysm, False OR Angiodysplasia OR Angioedema OR Angiomatosis OR Aortic Diseases OR Arterial Occlusive Diseases OR Arteriovenous Malformations OR Capillary Leak Syndrome OR Cerebrovascular Disorders OR Colitis, Ischemic OR Compartment Syndromes OR Diabetic Angiopathies OR Embolism and Thrombosis OR Hand-Arm Vibration Syndrome OR Hemorrhoids OR Hemostatic Disorders OR Hepatic Veno-Occlusive Disease OR Hyperemia OR Hypertension OR Hypotension OR Mesenteric Ischemia OR Myocardial Ischemia OR Optic Neuropathy, Ischemic OR Peliosis Hepatis OR Penetrating Atherosclerotic Ulcer OR Peripheral Vascular Diseases OR Prehypertension OR Pulmonary Veno-Occlusive Disease OR Reperfusion Injury OR Retinal Vein Occlusion OR Scimitar Syndrome OR Spinal Cord Vascular Diseases OR Splenic Infarction OR “Stenosis, Pulmonary Vein” OR Superior Vena Cava Syndrome OR Telangiectasis OR Thoracic Outlet Syndrome OR Varicocele OR Varicose Veins OR Vascular Fistula OR Vascular Neoplasms OR Vascular System Injuries OR Vasculitis OR Vasoplegia OR Venous Insufficiency (Topic) AND Gallstones OR Biliary Calculi OR Calculi, Biliary OR Gall Stone OR Gall Stones OR Gallstone OR

	Common Bile Duct Calculi OR Biliary Calculi, Common Bile Duct OR Common Bile Duct Gall Stones OR Common Bile Duct Gall Stone OR Common Bile Duct Gallstones OR Gall Stones, Common Bile Duct OR Common Bile Duct Gallstone OR Gallstones, Common Bile Duct (Topic)=1315

Table 2: The results of the Newcastle-Ottawa Scale (NOS) for assessment of the quality of the observational studies

Publication first author	Year	Components of the NOS checklist			Total score
		Selection	Comparability	Exposure/ Outcome	
Chen HY	2023	2	2	2	6
Chae W	2023	3	1	2	6
Bai R	2023	3	2	2	7
Park SM	2022	2	2	2	6
Ho TC	2021	2	1	2	5
Gill ES	2021	2	2	1	5
Chen CH	2021	3	2	3	8
Shabanzadeh DM	2017	3	2	2	7
Kwon CH	2017	3	1	2	6
Zheng Y	2016	4	2	2	8
Zheng Y	2016	3	2	3	8
Zheng Y	2016	3	2	3	8
Wirth J	2015	3	1	3	7
Lv J	2015	4	2	3	9
Wei CY	2014	4	2	2	8
Olaiya MT	2013	3	2	2	7
Jiang ZY	2013	3	1	2	6
Khan HN	2009	2	2	2	6
Méndez-Sánchez N	2008	2	1	2	5
González-Pérez A	2007	3	1	1	5
Méndez-Sánchez N	2005	2	1	1	4
Bortnichak EA	1985	3	1	2	6

Table 3: Characteristics of the studies included in the meta-analysis

First author	Year	Country	Study design	Study duration	Sample size	GD	Without GD	Average age	NOS score
Chen HY	2023	Taiwan	cohort	2001-2009	5,459	407	5,052	52.9	6
Chae W	2023	Korean	cohort	2002-2013	64,370	13,069	51,301	55	6
Bai R	2023	China	cohort	2016-2021	11,444	1,178	10,266	36.56	7
Park SM	2022	Korean	cohort	2005-2017	5,403,937	491,267	4,912,670	53	6
Ho TC	2021	Taiwan	cohort	2001-2011	11,796	5,992	5,804	57	5
Gill ES	2021	Korea	cross-sectional	2008-2010	566	90	476	66	5
Chen CH	2021	Taiwan	cohort	2000-2010	686,600	343,300	343,300	60	8
Shabanzadeh DM	2017	Denmark	cohort	1982-2015	5,496	504	4,992	41.8	7
Kwon CH	2017	South Korea	cross-sectional	2011-2014	38,778	1,426	37,352	41.1	6
Zheng Y	2016	USA	cohort	1980-2010	112,520	8,796	103,724	46.7	8
Zheng Y	2016	USA	cohort	1989-2011	112,919	5,227	107,692	34.9	8
Zheng Y	2016	USA	cohort	1986-2010	43,703	1,449	42,254	53.8	8
Wirth J	2015	Germany	cohort	1994-2006	46,486	4,828	41,658	50.5	7
Lv J	2015	China	cohort	2004-2013	487,373	28,345	459,028	51	9
Wei CY	2014	Taiwan	cohort	2000-2003	406,536	135,512	271,024	55	8
Olaiya MT	2013	Taiwan	cohort	2004-2005	34,905	6,981	27,924	45	7
Jiang ZY	2013	China	cross-sectional	2007-2011	1,270	206	1,064	64.5	6
Khan HN	2009	UK	cohort	1988-1998	4,420	2,210	2,210	78	6
Méndez-Sánchez N	2008	Mexico	cross-sectional	2004-2006	191	62	129	47	5
González-Pérez A	2007	Spain	case control	1996	12,353	2,353	10,000	55	5
Méndez-Sánchez N	2005	Mexico	cross-sectional	2003	473	119	354	47	4
Bortnichak EA	1985	USA	cohort	1948-1974	4,708	502	4,206	-	6

NOS; Newcastle-Ottawa Scale, UK; United Kingdom, US; United States.

Table 4: The effect size of the relationship between GD and CVDs in included studies.

First author	Year	Study cohort	Follow up (Year)	Women %	CVDs
Chen HY	2023	Male aged under 40 years old and over 79 years old who had a history of stroke or ischemic heart disease	5-11	0	2.10(1.49-2.96)
Chae W	2023	All Korean citizens' claims data are included in the NHIS database.	4.6	52	1.30(1.15-1.47)
Bai R	2023	Uyghur adults in Xinjiang	4.92	60	2.89(2.54-3.29)
Park SM	2022	All patients with gallstones from the KNHI data	7.5	48	1.20(1.12-1.29)
Ho TC	2021	Patients with GD.	6	51	1.2(1.16-1.25)
Gill ES	2021	Patients aged 40–89 years who had undergone abdominal ultrasound or abdominal computed tomography	2	28.4	2.01(1.15-3.53)
Chen CH	2021	We enrolled the patients aged ≥20 years who had a new diagnosis of GD	6.5	50.5	1.11(1.09-1.13)
Shabanzadeh DM	2017	General population	32	49.5	1.36[1.17;1.59]
Kwon CH	2017	Individuals who participated in a comprehensive health-screening program		22	0.86 (0.73-1.02)
Zheng Y	2016	The Nurses' Health Study -NHS	8	100	1.15(1.10-1.19)
Zheng Y	2016	The Nurses' Health Study -NHS II	8	100	1.27(1.14-1.42)
Zheng Y	2016	Health Professionals Follow-up Study -HPFS	8	0	1.10(1.04-1.16)
Wirth J	2015	Participants from 10 European countries	8	58	1.24(1.02-1.50)
Lv J	2015	participants aged 30–79 years in the China Kadoorie Biobank study	7.2	60	1.23 (1.17–1.28)
Wei CY	2014	The GD cohort comprised individuals with GD newly diagnosed in 2000-2003	10	50.7	1.29(1.26-1.315)
Olaiya MT	2013	Population including 6,981 patients with GD was identified from The Taiwan	6	56.2	1.32(122-1.43)
Jiang ZY	2013	Patients undergoing coronary angiography for the first time at Ruijin Hospital for suspected coronary artery disease	0	41	1.59(1.10-2.31)
Khan HN	2009	General population	10	56	1.03(0.91-1.16)
Méndez-Sánchez N	2008	Consecutive asymptomatic subjects who were referred to the checkup	0	42	2.12(1.04-4.34)
González-Pérez A	2007	General population	0.9	58	1.21(1.18-1.24)
Méndez-Sánchez N	2005	Consecutive asymptomatic subjects who were referred to the checkup	0	39	2.84 (1.33–6.07)
Bortnichak EA	1985	FRAMINGHAM cohort population	26	57	1.55(1.22-1.95)

Table 5: Adjusted variables in included studies in the meta-analysis of the relationship between GD and CVDs.

First author	year	Adjusted variables
Chen HY	2023	BMI, alcohol consumption, and regular exercise
Chae W	2023	Age, sex, and year
Bai R	2023	Sex, age, hypertension, T2DM, overweight, and HDL levels
Park SM	2022	Sex, age, SBP/DBP, Pulse pressure, Fasting plasma glucose, BMI, Smoking, Alcohol drinking, Physical activity.
Ho TC	2021	Age, sex, cardiovascular, and gastrointestinal comorbidity
Gill ES	2021	Age, sex, HTN, AF, OCAD, Smoking history
Chen CH	2021	Age, sex and comorbidities of hypertension, diabetes mellitus, hyperlipidemia, CHD, heart failure, COPD, PAOD, chronic renal disease, stroke, cirrhosis and alcohol-related illness.
Shabanzadeh DM	2017	Age, sex, cohort number, body mass index (kg/m 2), systolic blood pressure [140 mmHg, diastolic blood pressure[90 mmHg, non-high density lipoprotein cholesterol, high density lipoprotein cholesterol, smoking (never, past, current), alcohol consumption (units/week), diet (western, prudent, healthy), physical activity level (sedentary, light, moderate or vigorous), social group (I–V), time-dependent coefficients for age, time-dependent coefficients for high density lipoprotein cholesterol, non-high density lipoprotein cholesterol, diastolic blood pressure, and/or smoking.
Kwon CH	2017	Age, center, year of screening exam, smoking status, alcohol intake, physical activity, education level, BMI, medication for hypertension, medication for diabetes, and medication for hyperlipidemia; HOMA-IR and fatty liver, metabolic syndrome; hs-CRP
Zheng Y	2016	Age, race, family history of MI, marital status, smoking status, body mass index, physical activity, diabetes, hypertension, hypercholesterolemia, regular use of aspirin, daily intake of alcohol, daily intake of the energy-adjusted dietary cholesterol, Healthy Eating Index, and daily energy intake
Wirth J	2015	Sex, age, study center, educational achievement, physical activity, smoking habits, alcohol intake, body mass index, waist circumference, and prevalent high blood pressure and hyperlipidemia
Lv J	2015	Age, sex (for whole cohort only), level of education, marital status, alcohol consumption, smoking status, physical activity, intake frequencies of red meat, fresh fruits, and vegetables, prevalent hypertension, prevalent diabetes, family history of heart attack, menopausal status (for women only). Model 3 additionally included body mass index (BMI).
Wei CY	2014	Age, sex and history of hypertension, diabetes, coronary heart disease, atrial fibrillation, and hyperlipidemia.
Olaiya MT	2013	Age, gender, peripheral vascular disease, chronic obstructive pulmonary disease, diabetes mellitus, hyperlipidemia, alcoholism, chronic liver disease, and anemia

Jiang ZY	2013	Age, gender, BMI, and waist circumference; serum TC, TG, LDL-C, and HDL-C, and fasting glucose concentrations; and history of hypertension, DM, NAFLD and MetS
Khan HN	2009	-
Méndez-Sánchez N	2008	Adult treatment panel; BMI, carotid artery intima–media thickness; HDL, homeostasis model assessment
González-Pérez A	2007	Age, Sex, Hyperlipidemia, Hypertension, Diabetes, Alcohol, BMI, Smoking, health services utilization
Méndez-Sánchez N	2005	Age, gender, and BMI
Bortnichak EA	1985	Sex, diabetes, left ventricular hypertrophy, serum cholesterol, age, length of follow-up,<br>systolic blood pressure, Framingham Relative Weight, cigarette smoking.

Table 6. Effect size and computational scales (log of effect size and corresponding confidence interval, standard error of effect size, weight assigned to each study) for studies included in the analysis

First authors	Year	*Name of data extractors	Date of data extraction	The study was eligible to be included in the review?	Effect size	Lower limit	Upper limit	log Effect size	log Lower limit	log Upper limit	Weight	selog Effect size
Méndez-Sánchez N	2005	RH, FA, KA	July-2024	Yes	2.84	1.33	6.07	1.043804	0.2851789	1.803359	1.51818	0.3872907
Gill ES	2021	RH, FA, KA	July-2024	Yes	2.01	1.15	3.53	0.6981347	0.1397619	1.261298	1.121536	0.2861061
González-Pérez A	2007	RH, FA, KA	July-2024	Yes	1.21	1.18	1.24	0.1906204	0.1655144	0.2151114	0.0495969	0.0126523
Ho TC	2021	RH, FA, KA	July-2024	Yes	1.2	1.16	1.25	0.1823216	0.14842	0.2231435	0.0747235	0.0190621
Méndez-Sánchez N	2008	RH, FA, KA	July-2024	Yes	2.12	1.04	4.34	0.7514161	0.0392207	1.467874	1.428654	0.3644525
Wirth J	2015	RH, FA, KA	July-2024	Yes	1.24	1.02	1.5	0.2151114	0.0198026	0.4054651	0.3856625	0.0983833
Khan HN	2009	RH, FA, KA	July-2024	Yes	1.03	.91	1.16	0.0295588	-0.0943107	0.14842	0.2427307	0.0619211
Jiang ZY	2013	RH, FA, KA	July-2024	Yes	1.59	1.1	2.31	0.463734	0.0953102	0.8372476	0.7419374	0.1892698
Kwon CH	2017	RH, FA, KA	July-2024	Yes	.86	.73	1.02	-0.1508229	-0.3147107	0.0198026	0.3345134	0.085335
Bortnichak EA	1985	RH, FA, KA	July-2024	Yes	1.55	1.22	1.95	0.4382549	0.1988509	0.6678294	0.4689785	0.1196374
Shabanzadeh DM	2017	RH, FA, KA	July-2024	Yes	1.36	1.17	1.59	0.3074847	0.1570037	0.463734	0.3067303	0.0782475
Olaiya MT	2013	RH, FA, KA	July-2024	Yes	1.32	1.22	1.43	0.2776317	0.1988509	0.3576744	0.1588236	0.0405162
Bai R	2023	RH, FA, KA	July-2024	Yes	2.89	2.54	3.29	1.061257	0.9321641	1.190888	0.2587235	0.0660009
Chen HY	2023	RH, FA, KA	July-2024	Yes	2.1	1.49	2.96	0.7419373	0.3987761	1.085189	0.6864131	0.1751054

Park SM	2022	RH, FA, KA	July-2024	Yes	1.2	1.12	1.29	0.1823216	0.1133287	0.2546422	0.1413135	0.0360494
Wei CY	2014	RH, FA, KA	July-2024	Yes	1.29	1.26	1.315	0.2546422	0.2311117	0.2738367	0.042725	0.0108992
Zheng Y	2016	RH, FA, KA	July-2024	Yes	1.15	1.1	1.19	0.1397619	0.0953102	0.1739533	0.0786431	0.020062
Chen CH	2021	RH, FA, KA	July-2024	Yes	1.11	1.09	1.13	0.10436	0.0861777	0.1222176	0.0360399	0.0091939
Zheng Y	2016	RH, FA, KA	July-2024	Yes	1.27	1.14	1.42	0.2390169	0.1310283	0.3506569	0.2196286	0.0560277
Chae W	2023	RH, FA, KA	July-2024	Yes	1.3	1.15	1.47	0.2623643	0.1397619	0.3852624	0.2455005	0.0626277
Zheng Y	2016	RH, FA, KA	July-2024	Yes	1.1	1.04	1.16	0.0953102	0.0392207	0.14842	0.1091993	0.027857
Lv J	2015	RH, FA, KA	July-2024	Yes	1.23	1.17	1.28	0.2070142	0.1570037	0.2468601	0.0898563	0.0229225

*RH; Refli Hasan, FA; Fatemeh Allahbakhsi, KA; Khadija Allahbakhshi.

All the studies included in the systematic review were also included in the meta-analysis.

Table 7. Studies identified in the literature search

Number	***Article specifications***	***Article status***	***Description***
1	Chen HY, Chang CJ, Yang YC, Lu FH, Sun ZJ, Wu JS. Renal Stones and Gallstones Correlated with the Ten-Year Risk Estimation of Atherosclerotic Cardiovascular Disease Based on the Pooled Cohort Risk Assessment of Males Aged 40-79. J Clin Med. 2023;12(6). Epub 20230316. doi: 10.3390/jcm12062309. PubMed PMID: 36983309; PubMed Central PMCID: PMCPMC10052154.	Included in the final analysis	Article included in systematic review and meta-analysis
2	Chae W, Lee HS, Jo JH, Chung MJ, Bang S, Park SW, et al. Impact of cholecystectomy on acute coronary syndrome according to metabolic condition: a nationwide population-based cohort study. Sci Rep. 2023;13(1):7300. Epub 20230505. doi: 10.1038/s41598-023-33440-4. PubMed PMID: 37147417; PubMed Central PMCID: PMCPMC10163235.	Included in the final analysis	Article included in systematic review and meta-analysis
3	Bai R, Wang J, Yang J, Cheng X, Zhang S, Zhang H, et al.!!!Gallbladder disease is associated with the risk of cardiovascular disease among Uyghurs in Xinjiang: a prospective cohort study. BMC Public Health. 2023;23(1):242. Epub 20230204. doi: 10.1186/s12889-023-15098-9. PubMed PMID: 36737734; PubMed Central PMCID: PMCPMC9898978.	Included in the final analysis	Article included in systematic review and meta-analysis
4	Park SM, Kim HJ, Kang TU, Swan H, Ahn HS. Cholecystectomy reduces the risk of myocardial and cerebral infarction in patients with gallstone-related infection. Sci Rep. 2022;12(1):16749. Epub 20221006. doi: 10.1038/s41598-022-20700-y. PubMed PMID: 36202881; PubMed Central PMCID: PMCPMC9537563.	Included in the final analysis	Article included in systematic review and meta-analysis
5	Ho TC, Chen YC, Lin CC, Tai HC, Wei CY, Yeh YH, Hsu CY. Reduced Risk of Atrial Fibrillation Following Cholecystectomy: A Nationwide Population-Based Study. Front Aging Neurosci. 2021;13:706815. Epub 20210902. doi: 10.3389/fnagi.2021.706815. PubMed PMID: 34539379; PubMed Central PMCID: PMCPMC8445074.	Included in the final analysis	Article included in systematic review and meta-analysis
6	Gill ES, Jeong YJ, Lee J. Association between gallstone disease and ischemic stroke in Korea. Neurology Asia. 2021;26(4).	Included in the final analysis	Article included in systematic review and meta-analysis

7	Chen CH, Lin CL, Kao CH. Risk of aortic dissection or aneurysm in patients with gallstone disease: a retrospective cohort study in Taiwan. BMJ Open. 2021;11(8):e049316. Epub 20210826. doi: 10.1136/bmjopen-2021-049316. PubMed PMID: 34446491; PubMed Central PMCID: PMCPMC8395267.	Included in the final analysis	Article included in systematic review and meta-analysis
8	Shabanzadeh DM, Skaaby T, Sørensen LT, Jørgensen T. Screen-detected gallstone disease and cardiovascular disease. Eur J Epidemiol. 2017;32(6):501-10. Epub 20170527. doi: 10.1007/s10654-017-0263-x. PubMed PMID: 28551778.	Included in the final analysis	Article included in systematic review and meta-analysis
9	Kwon CH, Kang JG, Lee HJ, Kim NH, Sung JW, Cheong E, Sung KC. ??Absence of association between gallstone and coronary artery calcification. Atherosclerosis. 2017;258:51-5. Epub 20170201. doi: 10.1016/j.atherosclerosis.2017.01.035. PubMed PMID: 28192729.	Included in the final analysis	Article included in systematic review and meta-analysis
10	Zheng Y, Xu M, Li Y, Hruby A, Rimm EB, Hu FB, et al. Gallstones and Risk of Coronary Heart Disease: Prospective Analysis of 270 000 Men and Women From 3 US Cohorts and Meta-Analysis. Arterioscler Thromb Vasc Biol. 2016;36(9):1997-2003. Epub 20160818. doi: 10.1161/atvbaha.116.307507. PubMed PMID: 27540264; PubMed Central PMCID: PMCPMC5001914.	Included in the final analysis	Article included in systematic review and meta-analysis
11	Wirth J, di Giuseppe R, Wientzek A, Katzke VA, Kloss M, Kaaks R, et al. Presence of gallstones and the risk of cardiovascular diseases: The EPIC-Germany cohort study. Eur J Prev Cardiol. 2015;22(3):326-34. Epub 20131031. doi: 10.1177/2047487313512218. PubMed PMID: 24177267.	Included in the final analysis	Article included in systematic review and meta-analysis
12	Lv J, Qi L, Yu C, Guo Y, Bian Z, Chen Y, et al. Gallstone Disease and the Risk of Ischemic Heart Disease. Arterioscler Thromb Vasc Biol. 2015;35(10):2232-7. Epub 20150813. doi: 10.1161/atvbaha.115.306043. PubMed PMID: 26272939; PubMed Central PMCID: PMCPMC4587542.	Included in the final analysis	Article included in systematic review and meta-analysis
13	Wei CY, Chung TC, Chen CH, Lin CC, Sung FC, Chung WT, et al. Gallstone disease and the risk of stroke: a nationwide population-based study. J Stroke Cerebrovasc Dis. 2014;23(7):1813-20. Epub 20140621. doi: 10.1016/j.jstrokecerebrovasdis.2014.04.024. PubMed PMID: 24957305.	Included in the final analysis	Article included in systematic review and meta-analysis
14	Olaiya MT, Chiou HY, Jeng JS, Lien LM, Hsieh FI. Significantly increased risk of cardiovascular disease among patients with gallstone disease: a population-based cohort study. PLoS One. 2013;8(10):e76448. Epub 20131003. doi: 10.1371/journal.pone.0076448. PubMed PMID: 24098504; PubMed Central PMCID: PMCPMC3789705.	Included in the final analysis	Article included in systematic review and meta-analysis

15	Jiang ZY, Sheng X, Xu CY, Li WW, Chang XX, Sun LY, et al. Gallbladder gallstone disease is associated with newly diagnosed coronary artery atherosclerotic disease: a cross-sectional study. PLoS One. 2013;8(9):e75400. Epub 20130918. doi: 10.1371/journal.pone.0075400. PubMed PMID: 24058685; PubMed Central PMCID: PMCPMC3776774.	Included in the final analysis	Article included in systematic review and meta-analysis
16	Khan HN, Harrison M, Bassett EE, Bates T. A 10-year follow-up of a longitudinal study of gallstone prevalence at necropsy in South East England. Dig Dis Sci. 2009;54(12):2736-41. doi: 10.1007/s10620-008-0682-3. PubMed PMID: 19160052.	Included in the final analysis	Article included in systematic review and meta-analysis
17	Méndez-Sánchez N, Zamora-Valdés D, Flores-Rangel JA, Pérez-Sosa JA, Vásquez-Fernández F, Lezama-Mora JI, et al. Gallstones are associated with carotid atherosclerosis. Liver Int. 2008;28(3):402-6. Epub 20071206. doi: 10.1111/j.1478-3231.2007.01632.x. PubMed PMID: 18069975.	Included in the final analysis	Article included in systematic review and meta-analysis
18	González-Pérez A, García Rodríguez LA. Gallbladder disease in the general population: association with cardiovascular morbidity and therapy. Pharmacoepidemiol Drug Saf. 2007;16(5):524-31. doi: 10.1002/pds.1346. PubMed PMID: 17103483.	Included in the final analysis	Article included in systematic review and meta-analysis
19	Méndez-Sánchez N, Bahena-Aponte J, Chávez-Tapia NC, Motola-Kuba D, Sánchez-Lara K, Ponciano-Radríguez G, et al. Strong association between gallstones and cardiovascular disease. Am J Gastroenterol. 2005;100(4):827-30. doi: 10.1111/j.1572-0241.2005.41214.x. PubMed PMID: 15784027.	Included in the final analysis	Article included in systematic review and meta-analysis
20	Bortnichak EA, Freeman DH, Jr., Ostfeld AM, Castelli WP, Kannel WB, Feinleib M, McNamara PM. The association between cholesterol cholelithiasis and coronary heart disease in Framingham, Massachusetts. Am J Epidemiol. 1985;121(1):19-30. doi: 10.1093/oxfordjournals.aje.a113978. PubMed PMID: 3155483.	Included in the final analysis	Article included in systematic review and meta-analysis
21	Rahimi R, Masoumi S, Badali A, Jafari N, Heidari-Soureshjani S, Sherwin CMT. Association Between Gallstone Disease and Risk of Mortality of Cardiovascular Disease and Cancer: A Systematic Review and Meta-Analysis. Cardiovasc Hematol Disord Drug Targets. 2024;24(1):47-58. doi: 10.2174/011871529x298791240607041246. PubMed PMID: 38874034.	Excluded from the final analysis	The article was excluded from the analysis because the article is a review.
22	Tana M, Tana C, Cocco G, Iannetti G, Romano M, Schiavone C. Acute acalculous cholecystitis and cardiovascular disease: a land of confusion. J Ultrasound. 2015;18(4):317-20. Epub 20150726. doi: 10.1007/s40477-015-0176-z. PubMed PMID: 26550069; PubMed Central PMCID: PMCPMC4630273.	Excluded from the final analysis	The article was excluded from the analysis because the article is a review.

23	Upala S, Sanguankeo A, Jaruvongvanich V. Gallstone Disease and the Risk of Cardiovascular Disease: A Systematic Review and Meta-Analysis of Observational Studies. Scand J Surg. 2017;106(1):21-7. Epub 20160708. doi: 10.1177/1457496916650998. PubMed PMID: 27255283.	Excluded from the final analysis	The article was excluded from the analysis because the article is a review.
24	Zhao S-F, Wang A-M, Yu X-J, Wang L-L, Xu X-N, Shi G-J. Association between gallstone and cardio-cerebrovascular disease: Systematic review and meta-analysis. Experimental and Therapeutic Medicine. 2019;17(4):3092-100.	Excluded from the final analysis	The article was excluded from the analysis because the article is a review.
25	Fan LL, Chen BH, Dai ZJ. The relation between gallstone disease and cardiovascular disease. Scientific reports. 2017;7(1):15104.	Excluded from the final analysis	The article was excluded from the analysis because the article is a review.
26	Fairfield CJ, Wigmore SJ, Harrison EM. Gallstone disease and the risk of cardiovascular disease. Scientific reports. 2019;9(1):5830.	Excluded from the final analysis	The article was excluded from the analysis because the article is a review.
27	Beckingham I. Gallstone disease. Bmj. 2001;322(7278):91-4.	Excluded from the final analysis	The article was excluded from the analysis because the article is a review.
28	Ahmed MH, Ali A. Nonalcoholic fatty liver disease and cholesterol gallstones: which comes first? Scandinavian Journal of Gastroenterology. 2014;49(5):521-7.	Excluded from the final analysis	The article was excluded from the analysis because the article is a review.
29	FRIEDMAN GD. The relationship between coronary heart disease and gallbladder disease: A critical review. Annals of Internal Medicine. 1968;68(1):222-35.	Excluded from the final analysis	The article was excluded from the analysis because the article is a review.
30	HAMPTON AG, BECKWITH JR, WOOD JR JE. The relationship between heart disease and gall-bladder disease. Annals of internal medicine. 1959;50(5):1135-48.	Excluded from the final analysis	The article was excluded from the analysis because the article is a review.

31	Song Y, Wang H, Xu Y. Cholecystectomy and risk of cardiovascular disease, all-cause and cause-specific mortality: a systematic review and updated meta-analysis. PeerJ. 2024;12:e18174.	Excluded from the final analysis	The article was excluded from the analysis because the article is a review.
32	Thapa L, Katwal S, Thapa BR. Association between gallstone disease and carotid intima-media thickness: a prospective observational cross-sectional study in a tertiary care center. Annals of Medicine and Surgery. 2024:10.1097.	Excluded from the final analysis	The article was excluded from the analysis because the effect size was not reported in this article or it could not be calculated.
33	Unalp-Arida A, Der JS, Ruhl CE. Longitudinal Study of Comorbidities and Clinical Outcomes in Persons with Gallstone Disease Using Electronic Health Records. J Gastrointest Surg. 2023;27(12):2843-56. Epub 20231101. doi: 10.1007/s11605-023-05861-z. PubMed PMID: 37914859.	Excluded from the final analysis	The article was excluded from the analysis because the effect size was not reported in this article or it could not be calculated.
34	Marco-Martínez J, Elola-Somoza FJ, Fernández-Pérez C, Bernal-Sobrino JL, Azaña-Gómez FJ, García-Klepizg JL, et al. Heart Failure Is a Poor Prognosis Risk Factor in Patients Undergoing Cholecystectomy: Results from a Spanish Data-Based Analysis. J Clin Med. 2021;10(8). Epub 20210416. doi: 10.3390/jcm10081731. PubMed PMID: 33923710; PubMed Central PMCID: PMCPMC8072897.	Excluded from the final analysis	The article was excluded from the analysis because the effect size was not reported in this article or it could not be calculated.
35	Seddighi S, Ghidari ME, Sadeghi A, Shahrbaf MA, Mahmanzar MA, Saadati S, Yari Z. Evaluation of the cardiovascular risk in patients with biliary stones: a descriptive cross-sectional study. Gastroenterol Hepatol Bed Bench. 2018;11(Suppl 1):S14-s9. PubMed PMID: 30774802; PubMed Central PMCID: PMCPMC6348000.	Excluded from the final analysis	The article was excluded from the analysis because the effect size was not reported in this article or it could not be calculated.
36	Yu KJ, Zhang JR, Li Y, Huang X, Liu T, Li C, Wang RT. Gallstone disease is associated with arterial stiffness progression. Hypertens Res. 2017;40(1):31-4. Epub 20160825. doi: 10.1038/hr.2016.109. PubMed PMID: 27558931.	Excluded from the final analysis	The article was excluded from the analysis because the effect size was not reported in this

			article or it could not be calculated.
37	Shabanzadeh DM, Sørensen LT, Jørgensen T. Gallstone disease and mortality: a cohort study. Int J Public Health. 2017;62(3):353-60. Epub 20161104. doi: 10.1007/s00038-016-0916-7. PubMed PMID: 27815564.	Excluded from the final analysis	The article was excluded from the analysis because the effect size was not reported in this article or it could not be calculated.
38	Leubner JK, Ortiz Z, Wolfrey J, Drake L. Can gallstones break the heart? Pancreatitis-induced Takotsubo cardiomyopathy mimicking acute myocardial infarction. J Am Geriatr Soc. 2014;62(9):1814-5. doi: 10.1111/jgs.12999. PubMed PMID: 25243697.	Excluded from the final analysis	The article was excluded from the analysis because the effect size was not reported in this article or it could not be calculated.
39	Kim JH, Ryoo JG, Lee JW, Kim JH. Gallstones are Associated with Intima-Media Thickness of Common Carotid Arteries in Men. Korean J Fam Med. 2014;35(3):136-42. Epub 20140522. doi: 10.4082/kjfm.2014.35.3.136. PubMed PMID: 24921032; PubMed Central PMCID: PMCPMC4040431.	Excluded from the final analysis	The article was excluded from the analysis because the effect size was not reported in this article or it could not be calculated.
40	Ruhl CE, Everhart JE. Gallstone disease is associated with increased mortality in the United States. Gastroenterology. 2011;140(2):508-16. Epub 20101111. doi: 10.1053/j.gastro.2010.10.060. PubMed PMID: 21075109; PubMed Central PMCID: PMCPMC3060665.	Excluded from the final analysis	The article was excluded from the analysis because the effect size was not reported in this article or it could not be calculated.
41	Cher DJ. Myocardial infarction and acute cholecystitis: an application of sequence symmetry analysis. Epidemiology. 2000;11(4):446-9. doi: 10.1097/00001648-200007000-00014. PubMed PMID: 10874553.	Excluded from the final analysis	The article was excluded from the analysis because the effect size was not reported in this

			article or it could not be calculated.
42	Grimaldi CH, Nelson RG, Pettitt DJ, Sampliner RE, Bennett PH, Knowler WC. Increased mortality with gallstone disease: results of a 20-year population-based survey in Pima Indians. Ann Intern Med. 1993;118(3):185-90. doi: 10.7326/0003-4819-118-3-199302010-00005. PubMed PMID: 8417635.	Excluded from the final analysis	The article was excluded from the analysis because the effect size was not reported in this article or it could not be calculated.
43	Núñez Moreno FA, Ortiz Higareda V, Hernández Trejo LL, Acosta Gaxiola LE. Acute Cholecystitis Complicating Cardiac Disease: A Cohort Study From a Tertiary Care Center in Mexico City, Mexico. Cureus. 2024;16(2):e53915. Epub 20240209. doi: 10.7759/cureus.53915. PubMed PMID: 38465030; PubMed Central PMCID: PMCPMC10924644.	Excluded from the final analysis	The article was excluded from the analysis because the effect size was not reported in this article or it could not be calculated.
44	Chae W, Lee HS, Jo JH, Chung MJ, Bang S, Park SW, et al. Impact of cholecystectomy on acute coronary syndrome according to metabolic condition: a nationwide population-based cohort study. Sci Rep. 2023;13(1):7300. Epub 20230505. doi: 10.1038/s41598-023-33440-4. PubMed PMID: 37147417; PubMed Central PMCID: PMCPMC10163235.	Excluded from the final analysis	The article was excluded from the analysis because the effect size was not reported in this article or it could not be calculated.
45	Palsson SH, Engstrom C, Enochsson L, Osterlund E, Sandblom G. Risk factors for postoperative myocardial infarct following cholecystectomy: a population-based study. HPB (Oxford). 2020;22(1):34-40. Epub 20190718. doi: 10.1016/j.hpb.2019.06.018. PubMed PMID: 31327561.	Excluded from the final analysis	The article was excluded from the analysis because the effect size was not reported in this article or it could not be calculated.
46	Wang JY, Lu FH, Sun ZJ, Wu JS, Yang YC, Lee CT, Chang CJ. Gallstone disease associated with increased risk of arterial stiffness in a Taiwanese population. J Hum Hypertens. 2017;31(10):616-9. Epub 20170629. doi: 10.1038/jhh.2017.43. PubMed PMID: 28660886.	Excluded from the final analysis	The article was excluded from the analysis because the effect size was not reported in this

			article or it could not be calculated.
47	Ibrahim S, Hovingh GK, Hutten BA, Stroes ESG, Reeskamp LF. Impact of cumulative LDL-C and other risk factors on CAD prevalence in patients with familial hypercholesterolemia. Eur J Prev Cardiol. 2024. Epub 20241030. doi: 10.1093/eurjpc/zwae349. PubMed PMID: 39475097.	Excluded from the final analysis	The article was excluded from the analysis due to the impossibility of accessing the full text of the article.
48	Bloch A, Girod M, Bersier AL, Beurret L, Marc F, Maeder JP, et al. [Risk factors and psycho-social factors in myocardial infarct of the young man]. Schweiz Med Wochenschr. 1979;109(49):1963-6. PubMed PMID: 538437.	Excluded from the final analysis	The article was excluded from the analysis due to the impossibility of accessing the full text of the article.
49	Khan HN, Harrison M, Bassett EE, Bates T. A 10-Year Follow-up of a Longitudinal Study of Gallstone Prevalence at Necropsy in South East England. Digestive Diseases and Sciences. 2009;54(12):2736-41. doi: 10.1007/s10620-008-0682-3. PubMed PMID: WOS:000271923300028.	Excluded from the final analysis	The article was excluded from the analysis due to the lack of relation between the title and the abstract of the article with the subject under study
50	Gorsky RD, Pamuk E, Williamson DF, Shaffer PA, Koplan JP. The 25-year health care costs of women who remain overweight after 40 years of age. Am J Prev Med. 1996;12(5):388-94. PubMed PMID: 8909650.	Excluded from the final analysis	The article was excluded from the analysis due to the lack of relation between the title and the abstract of the article with the subject under study
51	Bian SN, Yang HH, Wang Q, Xu D, Zhao Y. [The 452th case: rash, hypotension, abdominal pain and headache]. Zhonghua Nei Ke Za Zhi. 2016;55(9):741-4. doi: 10.3760/cma.j.issn.0578-1426.2016.09.020. PubMed PMID: 27586989.	Excluded from the final analysis	The article was excluded from the analysis due to the lack of relation between the title and the abstract of the article with the subject under study

52	Sabeva NS, Liu JJ, Graf GA. The ABCG5 ABCG8 sterol transporter and phytosterols: implications for cardiometabolic disease. Current Opinion in Endocrinology Diabetes and Obesity. 2009;16(2):172-7. doi: 10.1097/MED.0b013e3283292312. PubMed PMID: WOS:000285055600012.	Excluded from the final analysis	The article was excluded from the analysis due to the lack of relation between the title and the abstract of the article with the subject under study
53	Stender S, Frikke-Schmidt R, Nordestgaard BG, Tybjærg-Hansen A. The ABCG5/8 Cholesterol Transporter and Myocardial Infarction Versus Gallstone Disease. Journal of the American College of Cardiology. 2014;63(20):2121-8. doi: 10.1016/j.jacc.2013.12.055. PubMed PMID: WOS:000336372700011.	Excluded from the final analysis	The article was excluded from the analysis due to the lack of relation between the title and the abstract of the article with the subject under study
54	Boraschi P, Giugliano L, Mercogliano G, Donati F, Romano S, Neri E. Abdominal and gastrointestinal manifestations in COVID-19 patients: Is imaging useful? World J Gastroenterol. 2021;27(26):4143-59. doi: 10.3748/wjg.v27.i26.4143. PubMed PMID: 34326615; PubMed Central PMCID: PMCPMC8311532.	Excluded from the final analysis	The article was excluded from the analysis due to the lack of relation between the title and the abstract of the article with the subject under study
55	Boraschi P, Giugliano L, Mercogliano G, Donati F, Romano S, Neri E. Abdominal and gastrointestinal manifestations in COVID-19 patients: Is imaging useful? World Journal of Gastroenterology. 2021;27(26):4143-59. doi: 10.3748/wjg.v27.i26.4143. PubMed PMID: WOS:000680398100009.	Excluded from the final analysis	The article was excluded from the analysis due to the lack of relation between the title and the abstract of the article with the subject under study
56	Chigot JP, Bitker M, Chalgadian R, Laroussinie G, Cabrol A, Gandjbakhch I, et al. [Abdominal complications of heart surgery]. Arch Mal Coeur Vaiss. 1981;74(6):665-73. PubMed PMID: 6794488.	Excluded from the final analysis	The article was excluded from the analysis due to the lack of relation between the title and the abstract of the article with the subject under study

57	Gurusamy KS, Koti R, Davidson BR. Abdominal lift for laparoscopic cholecystectomy. Cochrane Database of Systematic Reviews. 2013;(8). doi: 10.1002/14651858.CD006574.pub4. PubMed PMID: WOS:000323928600044.	Excluded from the final analysis	The article was excluded from the analysis due to the lack of relation between the title and the abstract of the article with the subject under study
58	Gurusamy KS, Koti R, Samraj K, Davidson BR. Abdominal lift for laparoscopic cholecystectomy. Cochrane Database of Systematic Reviews. 2012;(5). doi: 10.1002/14651858.CD006574.pub3. PubMed PMID: WOS:000304099300006.	Excluded from the final analysis	The article was excluded from the analysis due to the lack of relation between the title and the abstract of the article with the subject under study
59	Chaudry G, Navarro OM, Levine DS, Oudjhane K. Abdominal manifestations of cystic fibrosis in children. Pediatr Radiol. 2006;36(3):233-40. Epub 20060104. doi: 10.1007/s00247-005-0049-2. PubMed PMID: 16391928.	Excluded from the final analysis	The article was excluded from the analysis due to the lack of relation between the title and the abstract of the article with the subject under study
60	Pata F, Stamati G, Nardo B. Abdominal Pain and Hypotension in a 70-Year-Old Woman. Jama. 2023;329(18):1603-4. doi: 10.1001/jama.2023.4441. PubMed PMID: 37083972.	Excluded from the final analysis	The article was excluded from the analysis due to the lack of relation between the title and the abstract of the article with the subject under study
61	Pata F, Stamati G, Nardo B. Abdominal Pain and Hypotension in a 70-Year-Old Woman. Jama-Journal of the American Medical Association. 2023;329(18):1603-4. doi: 10.1001/jama.2023.4441. PubMed PMID: WOS:000975895900001.	Excluded from the final analysis	The article was excluded from the analysis due to the lack of relation between the title and the abstract of the article with the subject under study

62	Cormier RE, Chase BA, Peterson GS, Pauker SG. Abdominal pain, atherosclerosis, and atrial fibrillation. The case for mesenteric ischemia. Med Decis Making. 1982;2(3):323-39. doi: 10.1177/0272989x8200200309. PubMed PMID: 7169938.	Excluded from the final analysis	The article was excluded from the analysis due to the lack of relation between the title and the abstract of the article with the subject under study
63	Leonardi MJ, Jamil KG, Hiscox B, Ross D, Hiatt JR. Abdominal Surgery after Lung Transplantation. American Surgeon. 2010;76(10):1130-4. PubMed PMID: WOS:000282855600025.	Excluded from the final analysis	The article was excluded from the analysis due to the lack of relation between the title and the abstract of the article with the subject under study
64	Sekine K, Nagata N, Sakamoto K, Arai T, Shimbo T, Shinozaki M, et al. Abdominal visceral fat accumulation measured by computed tomography associated with an increased risk of gallstone disease. J Gastroenterol Hepatol. 2015;30(8):1325-31. doi: 10.1111/jgh.12965. PubMed PMID: 25869941.	Excluded from the final analysis	The article was excluded from the analysis due to the lack of relation between the title and the abstract of the article with the subject under study
65	Meyer FP. About the 'omnipotence' of the chelation therapy. Forschende Komplementarmedizin. 1998;5(6):266-71. doi: 10.1159/000021151. PubMed PMID: WOS:000078310200002.	Excluded from the final analysis	The article was excluded from the analysis due to the lack of relation between the title and the abstract of the article with the subject under study
66	Kwon CH, Kang JG, Lee HJ, Kim NH, Sung JW, Cheong E, Sung KC. Absence of association between gallstone and coronary artery calcification. Atherosclerosis. 2017;258:51-5. doi: 10.1016/j.atherosclerosis.2017.01.035. PubMed PMID: WOS:000397406500007.	Excluded from the final analysis	The article was excluded from the analysis due to the lack of relation between the title and the abstract of the article with the subject under study

67	Oudhoff JP, Timmermans DR, Rietberg M, Knol DL, van der Wal G. The acceptability of waiting times for elective general surgery and the appropriateness of prioritising patients. BMC Health Serv Res. 2007;7:32. Epub 20070228. doi: 10.1186/1472-6963-7-32. PubMed PMID: 17328816; PubMed Central PMCID: PMCPMC1847814.	Excluded from the final analysis	The article was excluded from the analysis due to the lack of relation between the title and the abstract of the article with the subject under study
68	Oudhoff JP, Timmermans DRM, Rietberg M, Knol DL, van der Wal G. The acceptability of waiting times for elective general surgery and the appropriateness of prioritising patients. Bmc Health Services Research. 2007;7. doi: 10.1186/1472-6963-7-32. PubMed PMID: WOS:000245510700001.	Excluded from the final analysis	The article was excluded from the analysis due to the lack of relation between the title and the abstract of the article with the subject under study
69	Parolin M, Dassie F, Vettor R, Maffei P. Acromegaly and ultrasound: how, when and why? Journal of Endocrinological Investigation. 2020;43(3):279-87. doi: 10.1007/s40618-019-01111-9. PubMed PMID: WOS:000512798600003.	Excluded from the final analysis	The article was excluded from the analysis due to the lack of relation between the title and the abstract of the article with the subject under study
70	Barie PS, Eachempati SR. Acute acalculous cholecystitis. Curr Gastroenterol Rep. 2003;5(4):302-9. doi: 10.1007/s11894-003-0067-x. PubMed PMID: 12864960.	Excluded from the final analysis	The article was excluded from the analysis due to the lack of relation between the title and the abstract of the article with the subject under study
71	Barie PS, Eachempati SR. Acute acalculous cholecystitis. Gastroenterol Clin North Am. 2010;39(2):343-57, x. doi: 10.1016/j.gtc.2010.02.012. PubMed PMID: 20478490.	Excluded from the final analysis	The article was excluded from the analysis due to the lack of relation between the title and the abstract of the article with the subject under study

72	Barie PS, Eachempati SR. Acute Acalculous Cholecystitis. Gastroenterology Clinics of North America. 2010;39(2):343-+. doi: 10.1016/j.gtc.2010.02.012. PubMed PMID: WOS:000278867900012.	Excluded from the final analysis	The article was excluded from the analysis due to the lack of relation between the title and the abstract of the article with the subject under study
73	Doran H, Mihalache O, Bobirca F, Buga C, Patrascu T. Acute acalculous cholecystitis - difficulties of the diagnosis and treatment. Chirurgia. 2010;105(4):465-8. PubMed PMID: WOS:000281760700003.	Excluded from the final analysis	The article was excluded from the analysis due to the lack of relation between the title and the abstract of the article with the subject under study
74	Markaki I, Konsoula A, Markaki L, Spernovasilis N, Papadakis M. Acute acalculous cholecystitis due to infectious causes. World J Clin Cases. 2021;9(23):6674-85. doi: 10.12998/wjcc.v9.i23.6674. PubMed PMID: 34447814; PubMed Central PMCID: PMCPMC8362504.	Excluded from the final analysis	The article was excluded from the analysis due to the lack of relation between the title and the abstract of the article with the subject under study
75	Doran H, Mihalache O, Bobircă F, Bugă C, Pătraşcu T. [Acute acalculous cholecystitis--difficulties of diagnosis and treatment]. Chirurgia (Bucur). 2010;105(4):465-8. PubMed PMID: 20941966.	Excluded from the final analysis	The article was excluded from the analysis due to the lack of relation between the title and the abstract of the article with the subject under study
76	Hoyme M, Surber R, Schulze PC, Prochnau D. [Acute chest pain and new ECG changes in pacemaker patients : A clinical challenge]. Herzschrittmacherther Elektrophysiol. 2017;28(1):60-3. Epub 20170215. doi: 10.1007/s00399-017-0491-5. PubMed PMID: 28204915.	Excluded from the final analysis	The article was excluded from the analysis due to the lack of relation between the title and the abstract of the article with the subject under study

77	Boey JH, Way LW. Acute cholangitis. Ann Surg. 1980;191(3):264-70. doi: 10.1097/00000658-198003000-00002. PubMed PMID: 7362292; PubMed Central PMCID: PMCPMC1344694.	Excluded from the final analysis	The article was excluded from the analysis due to the lack of relation between the title and the abstract of the article with the subject under study
78	Masabanda-Celorio VE, Alvares-Sores ED, Lara-Orosco U. [Acute cholangitis secondary to periampullary duodenal diverticulum. Case report]. Rev Med Inst Mex Seguro Soc. 2023;61(2):234-8. Epub 20230301. PubMed PMID: 37201190; PubMed Central PMCID: PMCPMC10395870.	Excluded from the final analysis	The article was excluded from the analysis due to the lack of relation between the title and the abstract of the article with the subject under study
79	Moreno FAN, Higareda VO, Trejo LLH, Gaxiola LEA. Acute Cholecystitis Complicating Cardiac Disease: A Cohort Study From a Tertiary Care Center in Mexico City, Mexico. Cureus Journal of Medical Science. 2024;16(2). doi: 10.7759/cureus.53915. PubMed PMID: WOS:001173119900013.	Excluded from the final analysis	The article was excluded from the analysis due to the lack of relation between the title and the abstract of the article with the subject under study
80	Auda A, Al Abdullah R, Khalid MO, Alrasheed WY, Alsulaiman SA, Almulhem FT, et al. Acute Cholecystitis Presenting With Septic Shock as the First Presentation in an Elderly Patient. Cureus. 2022;14(1):e20981. Epub 20220106. doi: 10.7759/cureus.20981. PubMed PMID: 35004094; PubMed Central PMCID: PMCPMC8733902.	Excluded from the final analysis	The article was excluded from the analysis due to the lack of relation between the title and the abstract of the article with the subject under study
81	Saluja SS, Ray S, Gulati MS, Pal S, Sahni P, Chattopadhyay TK. Acute cholecystitis with massive upper gastrointestinal bleed: A case report and review of the literature. Bmc Gastroenterology. 2007;7. doi: 10.1186/1471-230x-7-12. PubMed PMID: WOS:000245801200001.	Excluded from the final analysis	The article was excluded from the analysis due to the lack of relation between the title and the abstract of the article with the subject under study

82	Patel N, Ariyarathenam A, Davies W, Harris A. Acute Cholecystits Leading to Ischemic ECG Changes in a Patient with No Underlying Cardiac Disease. Jsls-Journal of the Society of Laparoendoscopic Surgeons. 2011;15(1):105-8. doi: 10.4293/108680811x13022985131534. PubMed PMID: WOS:000293197700022.	Excluded from the final analysis	The article was excluded from the analysis due to the lack of relation between the title and the abstract of the article with the subject under study
83	Coles M, Madray V, Uy P. Acute Esophageal Necrosis in a Septic Patient with a History of Cardiovascular Disease. Case Rep Gastrointest Med. 2020;2020:1416743. Epub 20200511. doi: 10.1155/2020/1416743. PubMed PMID: 32455033; PubMed Central PMCID: PMCPMC7238344.	Excluded from the final analysis	The article was excluded from the analysis due to the lack of relation between the title and the abstract of the article with the subject under study
84	de Alencastro MC, Cardoso KT, Mendes CA, Boteon YL, de Carvalho RB, Fraga GP. Acute intestinal obstruction due to gallstone ileus. Rev Col Bras Cir. 2013;40(4):275-80. doi: 10.1590/s0100-69912013000400004. PubMed PMID: 24173476.	Excluded from the final analysis	The article was excluded from the analysis due to the lack of relation between the title and the abstract of the article with the subject under study
85	Yu M, Huang BW, Lin Y, Nie YX, Zhou ZX, Liu SS, Hou BH. Acute obstructive cholangitis due to fishbone in the common bile duct: a case report and review of the literature. Bmc Gastroenterology. 2019;19(1). doi: 10.1186/s12876-019-1088-8. PubMed PMID: WOS:000495586300001.	Excluded from the final analysis	The article was excluded from the analysis due to the lack of relation between the title and the abstract of the article with the subject under study
86	Fiedler F. Acute pancreatitis. Anasthesiologie & Intensivmedizin. 2014;55:577-93. PubMed PMID: WOS:000345112000003.	Excluded from the final analysis	The article was excluded from the analysis due to the lack of relation between the title and the abstract of the article with the subject under study

87	Khan MZ, Yousaf H, Jamil A, Zaib MS, Haddad N. Acute Pancreatitis Associated With Hypothermia: An Uncommon Presentation. Cureus Journal of Medical Science. 2020;12(6). doi: 10.7759/cureus.8859. PubMed PMID: WOS:000543761200005.	Excluded from the final analysis	The article was excluded from the analysis due to the lack of relation between the title and the abstract of the article with the subject under study
88	Suzuki N, Furuya R, Otsuka T, Miyazaki H, Okano H, Komatsu T, et al. Acute pancreatitis caused by duodenal bezoar and treated with endoscopic procedures. Acute Med Surg. 2022;9(1):e797. Epub 20220930. doi: 10.1002/ams2.797. PubMed PMID: 36203854; PubMed Central PMCID: PMCPMC9525622.	Excluded from the final analysis	The article was excluded from the analysis due to the lack of relation between the title and the abstract of the article with the subject under study
89	Chen CP, Chen CY, Lu CL, Chang FY, Lee SD. Acute pancreatitis complicated by splenic infarction: Case report. Advances in Therapy. 1999;16(6):257-62. PubMed PMID: WOS:000085211800002.	Excluded from the final analysis	The article was excluded from the analysis due to the lack of relation between the title and the abstract of the article with the subject under study
90	Sharma V, Sharma A, Aggarwal A, Bhardwaj G, Aggarwal S. Acute pancreatitis in a patient with vivax malaria. Jop. 2012;13(2):215-6. Epub 20120310. PubMed PMID: 22406604.	Excluded from the final analysis	The article was excluded from the analysis due to the lack of relation between the title and the abstract of the article with the subject under study
91	Chang CC, Hsieh YY, Tsai HD, Yang TC, Yeh LS, Hsu TY. Acute pancreatitis in pregnancy. Zhonghua Yi Xue Za Zhi (Taipei). 1998;61(2):85-92. PubMed PMID: 9532870.	Excluded from the final analysis	The article was excluded from the analysis due to the lack of relation between the title and the abstract of the article with the subject under study

92	Alshammari HS, Alabdali NA, Alanazi WS, Alshmmary SN, Alshammari AM, Alshammri ASS, et al. ACUTE PANCREATITIS SURGICAL APPROACHES. Indo American Journal of Pharmaceutical Sciences. 2019;6(6):12815-21. doi: 10.5281/zenodo.3255439. PubMed PMID: WOS:000475619700286.	Excluded from the final analysis	The article was excluded from the analysis due to the lack of relation between the title and the abstract of the article with the subject under study
93	Khiatah B, Huynh T, Frugoli A, Lyche KD. Acute Pancreatitis with an Ongoing Pancreatic Duct Leak Complicated by Refractory Pleural Effusion: A Case Report. American Journal of Case Reports. 2021;22. doi: 10.12659/ajcr.931330. PubMed PMID: WOS:000663129700001.	Excluded from the final analysis	The article was excluded from the analysis due to the lack of relation between the title and the abstract of the article with the subject under study
94	Foster RJ, Cowell GW. Acute paraumbilical vein recanalization: an unusual complication of acute pancreatitis. BJR Case Rep. 2015;1(1):20150021. Epub 20150420. doi: 10.1259/bjrcr.20150021. PubMed PMID: 30363191; PubMed Central PMCID: PMCPMC6159162.	Excluded from the final analysis	The article was excluded from the analysis due to the lack of relation between the title and the abstract of the article with the subject under study
95	Karsou SA, Alshukhairy A, Hashim IA. Acute presentation of an uncommon cause of renal failure. Labmedicine. 2006;37(10):607-10. doi: 10.1309/f5aggl034yrhvcxq. PubMed PMID: WOS:000240815800018.	Excluded from the final analysis	The article was excluded from the analysis due to the lack of relation between the title and the abstract of the article with the subject under study
96	Arnell TD, DeVirgilio C, Chang L, Bongard F, Stabile BE. Admission factors can predict the need for ICU monitoring in gallstone pancreatitis. American Surgeon. 1996;62(10):815-9. PubMed PMID: WOS:A1996VJ94300008.	Excluded from the final analysis	The article was excluded from the analysis due to the lack of relation between the title and the abstract of the article with the subject under study

97	Laws SAM, Cook PR, Rees M. Adrenal insufficiency masquerading as an acute abdomen. Hospital Medicine. 2001;62(2):118-9. PubMed PMID: WOS:000167078200019.	Excluded from the final analysis	The article was excluded from the analysis due to the lack of relation between the title and the abstract of the article with the subject under study
98	Schreinemakers JM, van Dam PS, Seldenrijk CA, Biesma DH, Borel Rinkes IH. [The adrenocortical carcinoma, a tumour of wide clinical diversity]. Ned Tijdschr Geneeskd. 2004;148(43):2109-13. PubMed PMID: 15553352.	Excluded from the final analysis	The article was excluded from the analysis due to the lack of relation between the title and the abstract of the article with the subject under study
99	Janjua TK, Siddique S, Ibrahim MF, Khurshaidi MN. Aeromonas hydrophila induced necrotizing fasciitis following laparoscopic cholecystectomy. J Pak Med Assoc. 2024;74(3):576-9. doi: 10.47391/jpma.9344. PubMed PMID: 38591302.	Excluded from the final analysis	The article was excluded from the analysis due to the lack of relation between the title and the abstract of the article with the subject under study
100	Moschopoulos C, Bailly JM, Bruninx G, Delcour C. [Agenesis of the right lobe of the liver. Apropos of a case]. Ann Radiol (Paris). 1993;36(4):323-7. PubMed PMID: 8239475.	Excluded from the final analysis	The article was excluded from the analysis due to the lack of relation between the title and the abstract of the article with the subject under study
101	Morley JE. The aging gut: physiology. Clin Geriatr Med. 2007;23(4):757-67, v-vi. doi: 10.1016/j.cger.2007.06.002. PubMed PMID: 17923336.	Excluded from the final analysis	The article was excluded from the analysis due to the lack of relation between the title and the abstract of the article with the subject under study

102	Meza V, Arnold J, Díaz LA, Valverde MA, Idalsoaga F, Ayares G, et al. Alcohol Consumption: Medical Implications, the Liver and Beyond. Alcohol and Alcoholism. 2022;57(3):283-91. doi: 10.1093/alcalc/agac013. PubMed PMID: WOS:000773116300001.	Excluded from the final analysis	The article was excluded from the analysis due to the lack of relation between the title and the abstract of the article with the subject under study
103	de Lorimier AA. Alcohol, wine, and health. Am J Surg. 2000;180(5):357-61. doi: 10.1016/s0002-9610(00)00486-4. PubMed PMID: 11137687.	Excluded from the final analysis	The article was excluded from the analysis due to the lack of relation between the title and the abstract of the article with the subject under study
104	de Lorimier AA. Alcohol, wine, and health. American Journal of Surgery. 2000;180(5):357-61. doi: 10.1016/s0002-9610(00)00486-4. PubMed PMID: WOS:000166008200009.	Excluded from the final analysis	The article was excluded from the analysis due to the lack of relation between the title and the abstract of the article with the subject under study
105	Chen JC, Ng CJ, Chiu TF, Chen HM. Altered neutrophil apoptosis activity is reversed by melatonin in liver ischemia-reperfusion. Journal of Pineal Research. 2003;34(4):260-4. doi: 10.1034/j.1600-079X.2003.t01-1-00031.x. PubMed PMID: WOS:000181835000004.	Excluded from the final analysis	The article was excluded from the analysis due to the lack of relation between the title and the abstract of the article with the subject under study
106	Bhaskar BS, Rao G, Joshi SB, Arun S, Ajay S. Anaesthesia for laparoscopic cholecystectomy in Bartter's syndrome. Indian J Anaesth. 2010;54(4):327-30. doi: 10.4103/0019-5049.68377. PubMed PMID: 20882176; PubMed Central PMCID: PMCPMC2943703.	Excluded from the final analysis	The article was excluded from the analysis due to the lack of relation between the title and the abstract of the article with the subject under study

107	Baddam A, Akuma O, Raj R, Akuma CM, Augustine SW, Sheikh Hanafi I, et al. Analysis of Risk Factors for Cholelithiasis: A Single-Center Retrospective Study. Cureus. 2023;15(9):e46155. Epub 20230928. doi: 10.7759/cureus.46155. PubMed PMID: 37900464; PubMed Central PMCID: PMCPMC10613112.	Excluded from the final analysis	The article was excluded from the analysis due to the lack of relation between the title and the abstract of the article with the subject under study
108	Lorenz FJ, Beauchamp-Perez F, Manni A, Chung T, Goldenberg D, Goyal N. Analysis of Time to Diagnosis and Outcomes Among Adults With Primary Hyperparathyroidism. JAMA Netw Open. 2022;5(12):e2248332. Epub 20221201. doi: 10.1001/jamanetworkopen.2022.48332. PubMed PMID: 36574247; PubMed Central PMCID: PMCPMC9857508.	Excluded from the final analysis	The article was excluded from the analysis due to the lack of relation between the title and the abstract of the article with the subject under study
109	Popović M. [Anemia in chronic renal insufficiency--case report]. Med Pregl. 1999;52(6-8):279-81. PubMed PMID: 10518388.	Excluded from the final analysis	The article was excluded from the analysis due to the lack of relation between the title and the abstract of the article with the subject under study
110	Guo Q, Chen YJ, Huang H. Anesthetic management in untreated Bland-White-Garland syndrome: a case report and literature review. Journal of Pain Research. 2019;12:2167-76. doi: 10.2147/jpr.S200534. PubMed PMID: WOS:000476860100002.	Excluded from the final analysis	The article was excluded from the analysis due to the lack of relation between the title and the abstract of the article with the subject under study
111	Morosetti D, Picchi E, Calcagni A, Lamacchia F, Cavallo AU, Bozzi A, et al. Anomalous development of the inferior vena cava: Case reports of agenesis and hypoplasia. Radiol Case Rep. 2018;13(4):895-903. Epub 20180705. doi: 10.1016/j.radcr.2018.04.018. PubMed PMID: 29997719; PubMed Central PMCID: PMCPMC6037009.	Excluded from the final analysis	The article was excluded from the analysis due to the lack of relation between the title and the abstract of the article with the subject under study

112	Bamback P, Baumgardner KC, Bartanuszova M, Nation HL, Occhialini AP. Anomalous gallbladder septum-A case report. International Journal of Surgery Case Reports. 2021;84. doi: 10.1016/j.ijscr.2021.106082. PubMed PMID: WOS:000675881300021.	Excluded from the final analysis	The article was excluded from the analysis due to the lack of relation between the title and the abstract of the article with the subject under study
113	Cerhan JR, Bernstein L, Severson RK, Davis S, Colt JS, Blair A, Hartge P. Anthropometrics, physical activity, related medical conditions, and the risk of non-hodgkin lymphoma. Cancer Causes Control. 2005;16(10):1203-14. doi: 10.1007/s10552-005-0358-7. PubMed PMID: 16215871.	Excluded from the final analysis	The article was excluded from the analysis due to the lack of relation between the title and the abstract of the article with the subject under study
114	Busbaih Z, Busbaih J, Odeh A, Albeladi AM, Almohammed Saleh AA. Appendiceal Adhesion to the Gallbladder Detected During Laparoscopic Cholecystectomy: A Case Report. Cureus. 2021;13(12):e20625. Epub 20211222. doi: 10.7759/cureus.20625. PubMed PMID: 35106195; PubMed Central PMCID: PMCPMC8788890.	Excluded from the final analysis	The article was excluded from the analysis due to the lack of relation between the title and the abstract of the article with the subject under study
115	Larsson SC, Burgess S. Appraising the causal role of smoking in multiple diseases: A systematic review and meta-analysis of Mendelian randomization studies. Ebiomedicine. 2022;82. doi: 10.1016/j.ebiom.2022.104154. PubMed PMID: WOS:000829756300004.	Excluded from the final analysis	The article was excluded from the analysis due to the lack of relation between the title and the abstract of the article with the subject under study
116	Burkitt DP. ARE OUR COMMONEST DISEASES PREVENTABLE. Preventive Medicine. 1977;6(4):556-9. doi: 10.1016/0091-7435(77)90040-8. PubMed PMID: WOS:A1977EE42700007.	Excluded from the final analysis	The article was excluded from the analysis due to the lack of relation between the title and the abstract of the article with the subject under study

117	Sinha AC, Singh PM, Bhat S. Are we operating too late? Mortality Analysis and Stochastic Simulation of Costs Associated with Bariatric Surgery: Reconsidering the BMI Threshold. Obes Surg. 2016;26(1):219-28. doi: 10.1007/s11695-015-1934-x. PubMed PMID: 26487650.	Excluded from the final analysis	The article was excluded from the analysis due to the lack of relation between the title and the abstract of the article with the subject under study
118	Naeem M, Rahimnajjad NA, Rahimnajjad MK, Khurshid M, Ahmed QJ, Shahid SM, et al. Assessment of characteristics of patients with cholelithiasis from economically deprived rural Karachi, Pakistan. BMC Res Notes. 2012;5:334. Epub 20120628. doi: 10.1186/1756-0500-5-334. PubMed PMID: 22741543; PubMed Central PMCID: PMCPMC3438066.	Excluded from the final analysis	The article was excluded from the analysis due to the lack of relation between the title and the abstract of the article with the subject under study
119	Barcenas CG, Gonzalez-Molina M, Hull AR. Association between acute pancreatitis and malignant hypertension with renal failure. Arch Intern Med. 1978;138(8):1254-6. PubMed PMID: 677980.	Excluded from the final analysis	The article was excluded from the analysis due to the lack of relation between the title and the abstract of the article with the subject under study
120	Stewart L, Griffiss JM, Jarvis GA, Way LW. The association between body mass index and severe biliary infections: a multivariate analysis. Am J Surg. 2012;204(5):574-9. Epub 20120811. doi: 10.1016/j.amjsurg.2012.07.002. PubMed PMID: 22892201.	Excluded from the final analysis	The article was excluded from the analysis due to the lack of relation between the title and the abstract of the article with the subject under study
121	Chavez-Tapia NC, Mac Kinney-Novelo I, Sifuentes-Rentería SE, Torres-Zavala M, Castro-Gastelum G, Sánchez-Lara K, et al. Association between cholecystectomy for gallstone disease and risk factors for cardiovascular disease. Annals of Hepatology. 2012;11(1):85-9. doi: 10.1016/s1665-2681(19)31490-5. PubMed PMID: WOS:000300213500010.	Excluded from the final analysis	The article was excluded from the analysis due to the lack of relation between the title and the abstract of the article with the subject under study

122	Latenstein CSS, Alferink LJM, Darwish Murad S, Drenth JPH, van Laarhoven C, de Reuver PR. The Association Between Cholecystectomy, Metabolic Syndrome, and Nonalcoholic Fatty Liver Disease: A Population-Based Study. Clin Transl Gastroenterol. 2020;11(4):e00170. doi: 10.14309/ctg.0000000000000170. PubMed PMID: 32352682; PubMed Central PMCID: PMCPMC7263655.	Excluded from the final analysis	The article was excluded from the analysis due to the lack of relation between the title and the abstract of the article with the subject under study
123	Zhao SF, Wang AM, Yu XJ, Wang LL, Xu XN, Shi GJ. Association between gallstone and cardio-cerebrovascular disease: Systematic review and meta-analysis. Experimental and Therapeutic Medicine. 2019;17(4):3092-100. doi: 10.3892/etm.2019.7291. PubMed PMID: WOS:000463793600092.	Excluded from the final analysis	The article was excluded from the analysis due to the lack of relation between the title and the abstract of the article with the subject under study
124	Thapa L, Katwal S, Thapa BR. Association between gallstone disease and carotid intima-media thickness: a prospective observational cross-sectional study in a tertiary care center. Annals of Medicine and Surgery. 2024;86(8):4410-5. doi: 10.1097/ms9.0000000000002269. PubMed PMID: WOS:001284492000007.	Excluded from the final analysis	The article was excluded from the analysis due to the lack of relation between the title and the abstract of the article with the subject under study
125	Serin H, Yilmaz YK, Turan Y, Arslan E, Erkoç MF, Doğan A, Celikbilek M. The association between gallstone disease and plaque in the abdominopelvic arteries. J Res Med Sci. 2017;22:11. Epub 20170127. doi: 10.4103/1735-1995.199087. PubMed PMID: 28458703; PubMed Central PMCID: PMCPMC5367247.	Excluded from the final analysis	The article was excluded from the analysis due to the lack of relation between the title and the abstract of the article with the subject under study
126	Serin HI, Yilmaz YK, Turan Y, Arslan E, Erkoç MF, Dogan A, Celikbilek M. The association between gallstone disease and plaque in the abdominopelvic arteries. Journal of Research in Medical Sciences. 2017;22. doi: 10.4103/1735-1995.199087. PubMed PMID: WOS:000402828300011.	Excluded from the final analysis	The article was excluded from the analysis due to the lack of relation between the title and the abstract of the article with the subject under study

127	Lin YC, Chen IC, Chen YJ, Lin CT, Chang JC, Wang TJ, et al. Association between HNF4A rs1800961 polymorphisms and gallstones in a Taiwanese population. J Gastroenterol Hepatol. 2024;39(2):305-11. Epub 20231206. doi: 10.1111/jgh.16426. PubMed PMID: 38058101.	Excluded from the final analysis	The article was excluded from the analysis due to the lack of relation between the title and the abstract of the article with the subject under study
128	Zhang Y, Sun L, Wang X, Chen Z. The association between hypertension and the risk of gallstone disease: a cross-sectional study. BMC Gastroenterol. 2022;22(1):138. Epub 20220326. doi: 10.1186/s12876-022-02149-5. PubMed PMID: 35346065; PubMed Central PMCID: PMCPMC8961935.	Excluded from the final analysis	The article was excluded from the analysis due to the lack of relation between the title and the abstract of the article with the subject under study
129	Pan T, Zhang C, Liang J, Wang X, Di X, Zhou Y, et al. Association between life-ever gallstones and depressive symptoms in U.S. adults: a cross-sectional study. Sci Rep. 2024;14(1):18845. Epub 20240814. doi: 10.1038/s41598-024-69777-7. PubMed PMID: 39143232; PubMed Central PMCID: PMCPMC11325026.	Excluded from the final analysis	The article was excluded from the analysis due to the lack of relation between the title and the abstract of the article with the subject under study
130	Liu X, Yan G, Xu B, Sun M. Association between monocyte-to-high-density lipoprotein-cholesterol ratio and gallstones in U.S. adults: findings from the National Health and Nutrition Examination Survey 2017-2020. Lipids Health Dis. 2024;23(1):173. Epub 20240607. doi: 10.1186/s12944-024-02166-1. PubMed PMID: 38849878; PubMed Central PMCID: PMCPMC11157827.	Excluded from the final analysis	The article was excluded from the analysis due to the lack of relation between the title and the abstract of the article with the subject under study
131	Chen CH, Nien CK, Yang CC, Yeh YH. Association Between Nonalcoholic Fatty Liver Disease and Coronary Artery Calcification. Digestive Diseases and Sciences. 2010;55(6):1752-60. doi: 10.1007/s10620-009-0935-9. PubMed PMID: WOS:000278578800037.	Excluded from the final analysis	The article was excluded from the analysis due to the lack of relation between the title and the abstract of the article with the subject under study

132	Morán S, Duque-López MX, Salmerón-Castro J, Rodríguez-Leal G, Martínez-Salgado H, Uribe M. Association between serum concentration of apolipoproteins A-I and B with gallbladder disease. Arch Med Res. 2003;34(3):194-9. doi: 10.1016/s0188-4409(03)00025-0. PubMed PMID: 14567398.	Excluded from the final analysis	The article was excluded from the analysis due to the lack of relation between the title and the abstract of the article with the subject under study
133	Jia G, Shu XO, Liu Y, Li HL, Cai H, Gao J, et al. Association of Adult Weight Gain With Major Health Outcomes Among Middle-aged Chinese Persons With Low Body Weight in Early Adulthood. JAMA Netw Open. 2019;2(12):e1917371. Epub 20191202. doi: 10.1001/jamanetworkopen.2019.17371. PubMed PMID: 31834393; PubMed Central PMCID: PMCPMC6991199.	Excluded from the final analysis	The article was excluded from the analysis due to the lack of relation between the title and the abstract of the article with the subject under study
134	Chen CH, Lin CL, Kao CH. Association of Gallbladder Polyp and Stroke: A Nationwide, Population-Based Study. Medicine (Baltimore). 2015;94(48):e2192. doi: 10.1097/md.0000000000002192. PubMed PMID: 26632906; PubMed Central PMCID: PMCPMC4674209.	Excluded from the final analysis	The article was excluded from the analysis due to the lack of relation between the title and the abstract of the article with the subject under study
135	Dixit M, Choudhuri G, Mittal B. Association of lipoprotein receptor, receptor-associated protein, and metabolizing enzyme gene polymorphisms with gallstone disease: A case-control study. Hepatology Research. 2006;36(1):61-9. doi: 10.1016/j.hepres.2006.05.005. PubMed PMID: WOS:000241198900010.	Excluded from the final analysis	The article was excluded from the analysis due to the lack of relation between the title and the abstract of the article with the subject under study
136	Prinz RA, Aranha GV. The association of primary hyperparathyroidism and pancreatitis. Am Surg. 1985;51(6):325-9. PubMed PMID: 3994175.	Excluded from the final analysis	The article was excluded from the analysis due to the lack of relation between the title and the abstract of the article with the subject under study

137	Lin BB, Huang RH, Lin BL, Hong YK, Lin ME, He XJ. Associations between nephrolithiasis and diabetes mellitus, hypertension and gallstones: A meta-analysis of cohort studies. Nephrology (Carlton). 2020;25(9):691-9. Epub 20200706. doi: 10.1111/nep.13740. PubMed PMID: 32525230.	Excluded from the final analysis	The article was excluded from the analysis due to the lack of relation between the title and the abstract of the article with the subject under study
138	Brown RC, Gray AR, Tey SL, Chisholm A, Burley V, Greenwood DC, Cade J. Associations between Nut Consumption and Health Vary between Omnivores, Vegetarians, and Vegans. Nutrients. 2017;9(11). doi: 10.3390/nu9111219. PubMed PMID: WOS:000416547200057.	Excluded from the final analysis	The article was excluded from the analysis due to the lack of relation between the title and the abstract of the article with the subject under study
139	Basnet S, Merikanto I, Lahti T, Männistö S, Laatikainen T, Vartiainen E, Partonen T. Associations of common chronic non-communicable diseases and medical conditions with sleep-related problems in a population-based health examination study. Sleep Sci. 2016;9(3):249-54. Epub 20161125. doi: 10.1016/j.slsci.2016.11.003. PubMed PMID: 28123670; PubMed Central PMCID: PMCPMC5241609.	Excluded from the final analysis	The article was excluded from the analysis due to the lack of relation between the title and the abstract of the article with the subject under study
140	Rathi P, Nair S. An Audit of Extrahepatic Portal Vein Obstruction: Experience from Tertiary Referral Center. J Assoc Physicians India. 2023;71(7):11-2. doi: 10.59556/japi.71.0281. PubMed PMID: 37449688.	Excluded from the final analysis	The article was excluded from the analysis due to the lack of relation between the title and the abstract of the article with the subject under study
141	Jorgensen LS, Christiansen P, Raundahl U, Ostgaard S, Christensen NJ, Fenger M, Flachs H. AUTONOMIC NERVOUS-SYSTEM FUNCTION IN PATIENTS WITH FUNCTIONAL ABDOMINAL-PAIN - AN EXPERIMENTAL-STUDY. Scandinavian Journal of Gastroenterology. 1993;28(1):63-8. doi: 10.3109/00365529309096046. PubMed PMID: WOS:A1993KG27500010.	Excluded from the final analysis	The article was excluded from the analysis due to the lack of relation between the title and the abstract of the article with the subject under study

142	Mellon M, Schiller A, Nelson AL, Stohl HE. Awareness of Pregnancy-Associated Health Risks Among Pregnant Women and Male Partners Surveyed in a Prenatal Clinic. J Womens Health (Larchmt). 2020;29(3):376-82. Epub 20191024. doi: 10.1089/jwh.2018.7585. PubMed PMID: 31647358.	Excluded from the final analysis	The article was excluded from the analysis due to the lack of relation between the title and the abstract of the article with the subject under study
143	Kullman E, Jonsson KA, Lindstrom E, Dahlin LG, Ansehn S, Borch K. Bacteremia associated with extracorporeal shockwave lithotripsy of gallbladder stones. Hepato-Gastroenterology. 1995;42(6):816-20. PubMed PMID: WOS:A1995TP86600011.	Excluded from the final analysis	The article was excluded from the analysis due to the lack of relation between the title and the abstract of the article with the subject under study
144	Alabdullah H, Aldarsouni FG, Dagestani H, Mashbari H. Balancing Urgency and Strategy in the Surgical Management of a Complex Case of Gallstone Ileus: A Surgical-Video-Based Case Report of a 60-Year-Old Female. Cureus. 2024;16(8):e67304. Epub 20240820. doi: 10.7759/cureus.67304. PubMed PMID: 39310589; PubMed Central PMCID: PMCPMC11415006.	Excluded from the final analysis	The article was excluded from the analysis due to the lack of relation between the title and the abstract of the article with the subject under study
145	Chen JH, Tsai MS, Chen CY, Lee HM, Cheng CF, Chiu YT, et al. Bariatric Surgery Did Not Increase the Risk of Gallstone Disease in Obese Patients: a Comprehensive Cohort Study. Obesity Surgery. 2019;29(2):464-73. doi: 10.1007/s11695-018-3532-1. PubMed PMID: WOS:000458263000016.	Excluded from the final analysis	The article was excluded from the analysis due to the lack of relation between the title and the abstract of the article with the subject under study
146	Abu-Abeid S, Gavert N, Klausner JM, Szold A. Bariatric surgery in adolescence. J Pediatr Surg. 2003;38(9):1379-82. doi: 10.1016/s0022-3468(03)00400-7. PubMed PMID: 14523824.	Excluded from the final analysis	The article was excluded from the analysis due to the lack of relation between the title and the abstract of the article with the subject under study

147	Grigor'eva IN, Romanova TI. [Basic risk factors and quality of a life at gallstone disease patients]. Eksp Klin Gastroenterol. 2011;(4):21-5. PubMed PMID: 21919235.	Excluded from the final analysis	The article was excluded from the analysis due to the lack of relation between the title and the abstract of the article with the subject under study
148	Curry SJ, Krist AH, Owens DK, Barry MJ, Caughey AB, Davidson K, et al. Behavioral Weight Loss Interventions to Prevent Obesity-Related Morbidity and Mortality in Adults US Preventive Services Task Force Recommendation Statement. Jama-Journal of the American Medical Association. 2018;320(11):1163-71. doi: 10.1001/jama.2018.13022. PubMed PMID: WOS:000444878600016.	Excluded from the final analysis	The article was excluded from the analysis due to the lack of relation between the title and the abstract of the article with the subject under study
149	Anderson JW, Hamilton CC, Brinkmankaplan V. BENEFITS AND RISKS OF AN INTENSIVE VERY-LOW-CALORIE DIET PROGRAM FOR SEVERE OBESITY. American Journal of Gastroenterology. 1992;87(1):6-15. PubMed PMID: WOS:A1992GX88700002.	Excluded from the final analysis	The article was excluded from the analysis due to the lack of relation between the title and the abstract of the article with the subject under study
150	Meyer FP. ber die laquo;Omnipotenz>> der Chelattherapie. Forsch Komplementarmed. 1998;5(6):266-71. doi: 10.1159/000021151. PubMed PMID: 9973659.	Excluded from the final analysis	The article was excluded from the analysis due to the lack of relation between the title and the abstract of the article with the subject under study
151	Galanello R, Origa R. Beta-thalassemia. Orphanet Journal of Rare Diseases. 2010;5. doi: 10.1186/1750-1172-5-11. PubMed PMID: WOS:000279951900001.	Excluded from the final analysis	The article was excluded from the analysis due to the lack of relation between the title and the abstract of the article with the subject under study

152	Kim SY, Song CM, Lim H, Lim MS, Bang W, Choi HG. Bidirectional association between gallstones and renal stones: Two longitudinal follow-up studies using a national sample cohort. Sci Rep. 2019;9(1):2620. Epub 20190222. doi: 10.1038/s41598-019-38964-2. PubMed PMID: 30796254; PubMed Central PMCID: PMCPMC6385337.	Excluded from the final analysis	The article was excluded from the analysis due to the lack of relation between the title and the abstract of the article with the subject under study
153	Knobel B, Rosman P, Gewurtz G. Bilateral hydronephrosis due to fecaloma in an elderly woman. J Clin Gastroenterol. 2000;30(3):311-3. doi: 10.1097/00004836-200004000-00022. PubMed PMID: 10777195.	Excluded from the final analysis	The article was excluded from the analysis due to the lack of relation between the title and the abstract of the article with the subject under study
154	Li C, Li YL, Gai ZB. Bile Acids and Farnesoid X Receptor: Novel Target for the Treatment of Diabetic Cardiomyopathy. Current Protein & Peptide Science. 2019;20(10):976-83. doi: 10.2174/1389203720666190726152847. PubMed PMID: WOS:000487248100004.	Excluded from the final analysis	The article was excluded from the analysis due to the lack of relation between the title and the abstract of the article with the subject under study
155	Boatright JH, Nickerson JM, Moring AG, Pardue MT. Bile acids in treatment of ocular disease. J Ocul Biol Dis Infor. 2009;2(3):149-59. Epub 20090827. doi: 10.1007/s12177-009-9030-x. PubMed PMID: 20046852; PubMed Central PMCID: PMCPMC2798994.	Excluded from the final analysis	The article was excluded from the analysis due to the lack of relation between the title and the abstract of the article with the subject under study
156	Luitel P, Paudel S, Thapaliya I, Dhungana S, Thapa N, Devkota S. Bile duct injury following laparoscopic cholecystectomy in a patient with a trifurcated hepatic duct: A case report. Int J Surg Case Rep. 2024;123:110145. Epub 20240810. doi: 10.1016/j.ijscr.2024.110145. PubMed PMID: 39241475; PubMed Central PMCID: PMCPMC11408799.	Excluded from the final analysis	The article was excluded from the analysis due to the lack of relation between the title and the abstract of the article with the subject under study

157	Luitel P, Paudel S, Thapaliya I, Dhungana S, Thapa N, Devkota S. Bile duct injury following laparoscopic cholecystectomy in a patient with a trifurcated hepatic duct: A case report. International Journal of Surgery Case Reports. 2024;123. doi: 10.1016/j.ijscr.2024.110145. PubMed PMID: WOS:001310704200001.	Excluded from the final analysis	The article was excluded from the analysis due to the lack of relation between the title and the abstract of the article with the subject under study
158	Makutonin M, Moghatederi A, Newton S, Ma Y, Meltzer AC. Biliary colic in the emergency department: A state-wide analysis of one-year costs and clinical outcomes. Surg Open Sci. 2023;12:9-13. Epub 20230210. doi: 10.1016/j.sopen.2023.02.002. PubMed PMID: 36866121; PubMed Central PMCID: PMCPMC9971543.	Excluded from the final analysis	The article was excluded from the analysis due to the lack of relation between the title and the abstract of the article with the subject under study
159	Makutonin M, Moghatederi A, Newton S, Ma Y, Meltzer AC. Biliary colic in the emergency department: A state-wide analysis of one-year costs and clinical outcomes. Surgery Open Science. 2023;12:9-13. doi: 10.1016/j.sopen.2023.02.002. PubMed PMID: WOS:000945891700001.	Excluded from the final analysis	The article was excluded from the analysis due to the lack of relation between the title and the abstract of the article with the subject under study
160	Régent D, Laurent V, Meyer-Bisch L, Barbary-Lefèvre C, Corby-Ciprian S, Mathias J. [Biliary colic: imaging diagnosis]. J Radiol. 2006;87(4 Pt 2):413-29. doi: 10.1016/s0221-0363(06)74024-0. PubMed PMID: 16691173.	Excluded from the final analysis	The article was excluded from the analysis due to the lack of relation between the title and the abstract of the article with the subject under study
161	Gonzalez JJ, Sanz L, Grana JL, Bermejo G, Navarrete F, Martinez E. Biliary lithiasis in the elderly patient: Morbidity and mortality due to biliary surgery. Hepato-Gastroenterology. 1997;44(18):1565-8. PubMed PMID: WOS:A1997YL47100007.	Excluded from the final analysis	The article was excluded from the analysis due to the lack of relation between the title and the abstract of the article with the subject under study

162	Kapoor VK, Benjamin IS. Biliary malignancies. Baillieres Clinical Gastroenterology. 1997;11(4):801-36. doi: 10.1016/s0950-3528(97)90022-1. PubMed PMID: WOS:000072017500011.	Excluded from the final analysis	The article was excluded from the analysis due to the lack of relation between the title and the abstract of the article with the subject under study
163	Régent D, Laurent V, Meyer-Bisch L, Barbary-Lefevre C, Corby-Ciprian S, Mathias J. Biliary pain:: how to recognise it?: How to investigate?: Diagnosis lithiasic cholangitis on the calculations of the VBIH of unknown liver G during the initial chirurgical movement. Journal De Radiologie. 2006;87(4):413-29. doi: 10.1016/s0221-0363(06)74024-0. PubMed PMID: WOS:000237318300002.	Excluded from the final analysis	The article was excluded from the analysis due to the lack of relation between the title and the abstract of the article with the subject under study
164	Arendt T, Nizze H, Mönig H, Kloehn S, Stüber E, Fölsch UR. Biliary pancreatic reflux-induced acute pancreatitis--myth or possibility? Eur J Gastroenterol Hepatol. 1999;11(3):329-35. doi: 10.1097/00042737-199903000-00019. PubMed PMID: 10333208.	Excluded from the final analysis	The article was excluded from the analysis due to the lack of relation between the title and the abstract of the article with the subject under study
165	Arendt T, Nizze H, Mönig H, Kloehn S, Stüber E, Fölsch UR. Biliary pancreatic:: reflux-induced acute pancreatitis -: myth or possibility? European Journal of Gastroenterology & Hepatology. 1999;11(3):329-35. doi: 10.1097/00042737-199903000-00019. PubMed PMID: WOS:000079267300019.	Excluded from the final analysis	The article was excluded from the analysis due to the lack of relation between the title and the abstract of the article with the subject under study
166	Teply BA, Wang H, Luber B, Sullivan R, Rifkind I, Bruns A, et al. Bipolar androgen therapy in men with metastatic castration-resistant prostate cancer after progression on enzalutamide: an open-label, phase 2, multicohort study. Lancet Oncol. 2018;19(1):76-86. Epub 20171214. doi: 10.1016/s1470-2045(17)30906-3. PubMed PMID: 29248236; PubMed Central PMCID: PMCPMC5875180.	Excluded from the final analysis	The article was excluded from the analysis due to the lack of relation between the title and the abstract of the article with the subject under study

167	Vellar ID. The blood supply of the biliary ductal system and its relevance to vasculobiliary injuries following cholecystectomy. Australian and New Zealand Journal of Surgery. 1999;69(11):816-20. doi: 10.1046/j.1440-1622.1999.01702.x. PubMed PMID: WOS:000083616100012.	Excluded from the final analysis	The article was excluded from the analysis due to the lack of relation between the title and the abstract of the article with the subject under study
168	Sağlam F, Sivrikoz E, Alemdar A, Kamalı S, Arslan U, Güven H. Bouveret syndrome: A fatal diagnostic dilemma of gastric outlet obstruction. Ulus Travma Acil Cerrahi Derg. 2015;21(2):157-9. doi: 10.5505/tjtes.2015.62558. PubMed PMID: 25904280.	Excluded from the final analysis	The article was excluded from the analysis due to the lack of relation between the title and the abstract of the article with the subject under study
169	Báez-García JJ, Martínez-Hernández-Magro P, Iriarte-Gállego G. [Bouveret's syndrome; a case report]. Rev Gastroenterol Mex. 2009;74(2):118-21. PubMed PMID: 19666294.	Excluded from the final analysis	The article was excluded from the analysis due to the lack of relation between the title and the abstract of the article with the subject under study
170	Mehdi I, Al Bahrani BJ, Al Lawati TM, Al Mandhari Z, Al Lawati FR. Breast cancer in a patient with Kindler's syndrome. Journal of the Pakistan Medical Association. 2017;67(8):1283-6. PubMed PMID: WOS:000405943300029.	Excluded from the final analysis	The article was excluded from the analysis due to the lack of relation between the title and the abstract of the article with the subject under study
171	Chowdhary M, Kabbani AA, Chhabra A. Canagliflozin-induced pancreatitis: a rare side effect of a new drug. Ther Clin Risk Manag. 2015;11:991-4. Epub 20150626. doi: 10.2147/tcrm.S86641. PubMed PMID: 26170677; PubMed Central PMCID: PMCPMC4489815.	Excluded from the final analysis	The article was excluded from the analysis due to the lack of relation between the title and the abstract of the article with the subject under study

172	Li YN, Li JH, Leng AJ, Zhang GX, Qu JL. Cardiac complications caused by biliary diseases: A review of clinical manifestations, pathogenesis and treatment strategies of cholecardia syndrome. Pharmacological Research. 2024;199. doi: 10.1016/j.phrs.2023.107006. PubMed PMID: WOS:001133475200001.	Excluded from the final analysis	The article was excluded from the analysis due to the lack of relation between the title and the abstract of the article with the subject under study
173	Syrén EL, Enochsson L, Eriksson S, Eklund A, Isaksson B, Sandblom G. Cardiovascular complications after common bile duct stone extractions. Surgical Endoscopy and Other Interventional Techniques. 2021;35(7):3296-302. doi: 10.1007/s00464-020-07766-3. PubMed PMID: WOS:000545478600004.	Excluded from the final analysis	The article was excluded from the analysis due to the lack of relation between the title and the abstract of the article with the subject under study
174	Duvnjak M, Supanc V, Virović L, Tomasić V, Dojcinović B. [Caroli's disease]. Acta Med Croatica. 2003;57(3):249-52. PubMed PMID: 14582472.	Excluded from the final analysis	The article was excluded from the analysis due to the lack of relation between the title and the abstract of the article with the subject under study
175	Zhuang X, Li L. [A case - control study of gallstone disease in female population in Taicang]. Zhonghua Liu Xing Bing Xue Za Zhi. 2000;21(1):44-7. PubMed PMID: 11860758.	Excluded from the final analysis	The article was excluded from the analysis due to the lack of relation between the title and the abstract of the article with the subject under study
176	Fukushima R, Ishii N, Harimoto N, Araki K, Watanabe A, Tsukagoshi M, et al. A case of Mirizzi syndrome accompanied by a pseudoaneurysm that ruptured into the gallbladder: successfully treated by embolization of aneurysm and sequential surgery. Surgical Case Reports. 2022;8(1). doi: 10.1186/s40792-022-01467-w. PubMed PMID: WOS:000811199000001.	Excluded from the final analysis	The article was excluded from the analysis due to the lack of relation between the title and the abstract of the article with the subject under study

177	Kakhktsyan T, Aleksanyan M, Acob T, Hakobyan K. A Case of Primary Biliary Cholangitis in a Patient With Multiple Sclerosis. Cureus. 2024;16(7):e63812. Epub 20240704. doi: 10.7759/cureus.63812. PubMed PMID: 39100035; PubMed Central PMCID: PMCPMC11297683.	Excluded from the final analysis	The article was excluded from the analysis due to the lack of relation between the title and the abstract of the article with the subject under study
178	Khan Z. A Case Report of Endoscopic Retrograde Cholangiopancreatography (ERCP) and Acute Pancreatitis Induced Takotsubo Cardiomyopathy (TCM) in a Patient With Gallstones Induced Acute Pancreatitis and Cholangitis. Cureus Journal of Medical Science. 2022;14(5). doi: 10.7759/cureus.24708. PubMed PMID: WOS:000800318600028.	Excluded from the final analysis	The article was excluded from the analysis due to the lack of relation between the title and the abstract of the article with the subject under study
179	Peng J, Zhang Y, Ling Q, Zhu L, Yao H. Case Report of Overlapping Pyloric Obstruction Due to Dichlorvos Poisoning and Cholelithiasis with Choledocholithiasis. Am J Case Rep. 2024;25:e943101. Epub 20240327. doi: 10.12659/ajcr.943101. PubMed PMID: 38532541; PubMed Central PMCID: PMCPMC10985417.	Excluded from the final analysis	The article was excluded from the analysis due to the lack of relation between the title and the abstract of the article with the subject under study
180	Peng J, Zhang YL, Ling Q, Zhu L, Yao HH. Case Report of Overlapping Pyloric Obstruction Due to Dichlorvos Poisoning and Cholelithiasis with Choledocholithiasis. American Journal of Case Reports. 2024;25. doi: 10.12659/ajcr.943101. PubMed PMID: WOS:001235178000001.	Excluded from the final analysis	The article was excluded from the analysis due to the lack of relation between the title and the abstract of the article with the subject under study
181	Sontakke S, Dhole P, Pathade A, Alnewar P. A Case Report On Obstructive Jaundice With Periampullary Adenocarcinoma And Cholangitis. International Journal of Early Childhood Special Education. 2022;14(5):4495-8. doi: 10.9756/intjecse/v14i5.535. PubMed PMID: WOS:000834158000047.	Excluded from the final analysis	The article was excluded from the analysis due to the lack of relation between the title and the abstract of the article with the subject under study

182	Fu QA, Shen TZ, Yu QY, Jiang L, Yang RQ. Causal effect of gallstone disease on the risk of coronary heart disease or acute myocardial infarction: a Mendelian randomization study. Scientific Reports. 2023;13(1). doi: 10.1038/s41598-023-46117-9. PubMed PMID: WOS:001105087400090.	Excluded from the final analysis	The article was excluded from the analysis due to the lack of relation between the title and the abstract of the article with the subject under study
183	Franks PW, Atabaki-Pasdar N. Causal inference in obesity research. J Intern Med. 2017;281(3):222-32. Epub 20161208. doi: 10.1111/joim.12577. PubMed PMID: 27933671.	Excluded from the final analysis	The article was excluded from the analysis due to the lack of relation between the title and the abstract of the article with the subject under study
184	Franks PW, Atabaki-Pasdar N. Causal inference in obesity research. Journal of Internal Medicine. 2017;281(3):222-32. doi: 10.1111/joim.12577. PubMed PMID: WOS:000394893800001.	Excluded from the final analysis	The article was excluded from the analysis due to the lack of relation between the title and the abstract of the article with the subject under study
185	Nordestgaard AT. Causal relationship from coffee consumption to diseases and mortality: a review of observational and Mendelian randomization studies including cardiometabolic diseases, cancer, gallstones and other diseases. European Journal of Nutrition. 2022;61(2):573-87. doi: 10.1007/s00394-021-02650-9. PubMed PMID: WOS:000678435300001.	Excluded from the final analysis	The article was excluded from the analysis due to the lack of relation between the title and the abstract of the article with the subject under study
186	Chaouch MA, Taieb AH, Kawach A, Zenati H, Gafsi B, Noomen F. Challenges in the management of acute lithiasic cholangitis due to a long-retained plastic biliary stent: A case report. Int J Surg Case Rep. 2024;118:109690. Epub 20240423. doi: 10.1016/j.ijscr.2024.109690. PubMed PMID: 38669806; PubMed Central PMCID: PMCPMC11064558.	Excluded from the final analysis	The article was excluded from the analysis due to the lack of relation between the title and the abstract of the article with the subject under study

187	Syvolap DV. Changes in reservoir and motor-evacuatory function of the gallbladder in patients with asymptomatic cholecystolithiasis after organ-sparing surgery - laparoscopic cholecystolithotomy. Pathologia. 2022;19(1):65-9. doi: 10.14739/2310-1237.2022.1.254270. PubMed PMID: WOS:000810874400010.	Excluded from the final analysis	The article was excluded from the analysis due to the lack of relation between the title and the abstract of the article with the subject under study
188	Riordan JP, Stewart RJ, Pearce NE, Isbister WH. CHANGES IN THE PATTERN OF DISEASE MANAGED BY GENERAL SURGEONS IN NEW-ZEALAND, 1940-80. New Zealand Medical Journal. 1984;97(767):762-5. PubMed PMID: WOS:A1984TT32700002.	Excluded from the final analysis	The article was excluded from the analysis due to the lack of relation between the title and the abstract of the article with the subject under study
189	Miltenburg DM, Schaffer R, Breslin T, Brandt ML. Changing indications for pediatric cholecystectomy. Pediatrics. 2000;105(6):1250-3. doi: 10.1542/peds.105.6.1250. PubMed PMID: WOS:000087441400026.	Excluded from the final analysis	The article was excluded from the analysis due to the lack of relation between the title and the abstract of the article with the subject under study
190	Bugajska J, Gotfryd-Bugajska K, Szura M, Berska J, Pasternak A, Sztefko K. Characteristics of amino acid profiles and incretin hormones in patients with gallstone disease: a pilot study. Polish Archives of Internal Medicine-Polskie Archiwum Medycyny Wewnetrznej. 2019;129(12):883-8. doi: 10.20452/pamw.14990. PubMed PMID: WOS:000511161200006.	Excluded from the final analysis	The article was excluded from the analysis due to the lack of relation between the title and the abstract of the article with the subject under study
191	Searle LC, Graham JM, Prasad C, Blake KD. CHARGE syndrome from birth to adulthood: An individual reported on from 0 to 33 years. American Journal of Medical Genetics Part A. 2005;133A(3):344-9. doi: 10.1002/ajmg.a.30565. PubMed PMID: WOS:000227263900020.	Excluded from the final analysis	The article was excluded from the analysis due to the lack of relation between the title and the abstract of the article with the subject under study

192	Gao J, Yuan GY, Xu Z, Lan LY, Xin WK. Chenodeoxycholic and deoxycholic acids induced positive inotropic and negative chronotropic effects on rat heart. Naunyn-Schmiedebergs Archives of Pharmacology. 2021;394(4):765-73. doi: 10.1007/s00210-020-01962-7. PubMed PMID: WOS:000560287300001.	Excluded from the final analysis	The article was excluded from the analysis due to the lack of relation between the title and the abstract of the article with the subject under study
193	Ros E, Armengol X, Grande L, ToledoPimentel V, Lacima G, Sanz G. Chest pain at rest in patients with coronary artery disease - Myocardial ischemia, esophageal dysfunction, or panic disorder? Digestive Diseases and Sciences. 1997;42(7):1344-53. doi: 10.1023/a:1018821417134. PubMed PMID: WOS:A1997XN21600002.	Excluded from the final analysis	The article was excluded from the analysis due to the lack of relation between the title and the abstract of the article with the subject under study
194	Ros E, Armengol X, Grande L, Toledo-Pimentel V, Lacima G, Sanz G. Chest pain at rest in patients with coronary artery disease. Myocardial ischemia, esophageal dysfunction, or panic disorder? Dig Dis Sci. 1997;42(7):1344-53. doi: 10.1023/a:1018821417134. PubMed PMID: 9246027.	Excluded from the final analysis	The article was excluded from the analysis due to the lack of relation between the title and the abstract of the article with the subject under study
195	Koshiol J, Van de Wyngard V, McGee EE, Cook P, Pfeiffer RM, Mardones N, et al. The Chile Biliary Longitudinal Study: A Gallstone Cohort. American Journal of Epidemiology. 2021;190(2):196-206. doi: 10.1093/aje/kwaa199. PubMed PMID: WOS:000636958300003.	Excluded from the final analysis	The article was excluded from the analysis due to the lack of relation between the title and the abstract of the article with the subject under study
196	Malkan GH, Bhatia SJ, Bashir K, Khemani R, Abraham P, Gandhi MS, Radhakrishnan R. Cholangiopathy associated with portal hypertension: diagnostic evaluation and clinical implications. Gastrointest Endosc. 1999;49(3 Pt 1):344-8. doi: 10.1016/s0016-5107(99)70011-8. PubMed PMID: 10049418.	Excluded from the final analysis	The article was excluded from the analysis due to the lack of relation between the title and the abstract of the article with the subject under study

197	Malkan GH, Bhatia SJ, Bashir K, Khemani R, Abraham P, Gandhi MS, Radhakrishnan R. Cholangiopathy associated with portal hypertension: diagnostic evaluation and clinical implications. Gastrointestinal Endoscopy. 1999;49(3):344-8. doi: 10.1016/s0016-5107(99)70011-8. PubMed PMID: WOS:000079015000011.	Excluded from the final analysis	The article was excluded from the analysis due to the lack of relation between the title and the abstract of the article with the subject under study
198	Kasap E, Akyildiz M, Akarca U. CHOLANGITIS OF PANCREATITIS? DOES THE ANGIOTENSIN-CONVERTING ENZYME GENOTYPE FAVOR EITHER? Balkan Journal of Medical Genetics. 2009;12(2):53-7. doi: 10.2478/v10034-010-0006-8. PubMed PMID: WOS:000276575500007.	Excluded from the final analysis	The article was excluded from the analysis due to the lack of relation between the title and the abstract of the article with the subject under study
199	Song Y, Wang HS, Xu YW. Cholecystectomy and risk of cardiovascular disease, all-cause and cause-specific mortality: a systematic review and updated meta-analysis. Peerj. 2024;12. doi: 10.7717/peerj.18174. PubMed PMID: WOS:001327935700001.	Excluded from the final analysis	The article was excluded from the analysis due to the lack of relation between the title and the abstract of the article with the subject under study
200	Lord RVN, Ho S, Coleman MJ, Spratt PM. Cholecystectomy in cardiothoracic organ transplant recipients. Archives of Surgery. 1998;133(1):73-9. doi: 10.1001/archsurg.133.1.73. PubMed PMID: WOS:000071416200015.	Excluded from the final analysis	The article was excluded from the analysis due to the lack of relation between the title and the abstract of the article with the subject under study
201	Dubecz A, Langer M, Stadlhuber RJ, Schweigert M, Solymosi N, Feith M, Stein HJ. Cholecystectomy in the very elderly--is 90 the new 70? J Gastrointest Surg. 2012;16(2):282-5. Epub 20111206. doi: 10.1007/s11605-011-1708-2. PubMed PMID: 22143419.	Excluded from the final analysis	The article was excluded from the analysis due to the lack of relation between the title and the abstract of the article with the subject under study

202	Huh JH, Lee KJ, Cho YK, Moon S, Kim YJ, Han KD, et al. Cholecystectomy increases the risk of metabolic syndrome in the Korean population: a longitudinal cohort study. Hepatobiliary Surg Nutr. 2023;12(4):523-33. Epub 20221206. doi: 10.21037/hbsn-22-201. PubMed PMID: 37600984; PubMed Central PMCID: PMCPMC10432299.	Excluded from the final analysis	The article was excluded from the analysis due to the lack of relation between the title and the abstract of the article with the subject under study
203	Zhao J, Tian L, Xia B, Mi N, He Q, Yang M, et al. Cholecystectomy is associated with a higher risk of irritable bowel syndrome in the UK Biobank: a prospective cohort study. Front Pharmacol. 2023;14:1244563. Epub 20231208. doi: 10.3389/fphar.2023.1244563. PubMed PMID: 38143491; PubMed Central PMCID: PMCPMC10749201.	Excluded from the final analysis	The article was excluded from the analysis due to the lack of relation between the title and the abstract of the article with the subject under study
204	Kwak MS, Kim D, Chung GE, Kim W, Kim YJ, Yoon JH. Cholecystectomy is independently associated with nonalcoholic fatty liver disease in an Asian population. World J Gastroenterol. 2015;21(20):6287-95. doi: 10.3748/wjg.v21.i20.6287. PubMed PMID: 26034364; PubMed Central PMCID: PMCPMC4445106.	Excluded from the final analysis	The article was excluded from the analysis due to the lack of relation between the title and the abstract of the article with the subject under study
205	Park SM, Kim HJ, Kang TU, Swan H, Ahn HS. Cholecystectomy reduces the risk of myocardial and cerebral infarction in patients with gallstone-related infection. Scientific Reports. 2022;12(1). doi: 10.1038/s41598-022-20700-y. PubMed PMID: WOS:000864845400031.	Excluded from the final analysis	The article was excluded from the analysis due to the lack of relation between the title and the abstract of the article with the subject under study
206	Seal ML. Cholecystitis occurring without stones. Postgrad Med. 1986;79(4):151-4, 8. doi: 10.1080/00325481.1986.11699320. PubMed PMID: 3513145.	Excluded from the final analysis	The article was excluded from the analysis due to the lack of relation between the title and the abstract of the article with the subject under study

207	Nagappa M, Taly AB. Cholecysto-cardiac link: The heart of the matter. Neurology India. 2019;67(2):391-2. doi: 10.4103/0028-3886.258015. PubMed PMID: WOS:000468100900010.	Excluded from the final analysis	The article was excluded from the analysis due to the lack of relation between the title and the abstract of the article with the subject under study
208	Fei Y, Li WQ, Zong GQ, Chen J, Wang W. Cholecystolithotomy Combined Armillarisin A versus Cholecystectomy in Cirrhotic Portal Hypertension Patients with Symptomatic Cholelithiasis. Chirurgia (Bucur). 2017;112(2):143-51. doi: 10.21614/chirurgia.112.2.143. PubMed PMID: 28463673.	Excluded from the final analysis	The article was excluded from the analysis due to the lack of relation between the title and the abstract of the article with the subject under study
209	Valadez-Caballero D, González-Santamaría R, Soto-Mendoza H, Blanco-Figueroa JA, Flores-Pantoja JM. Choledocholithiasis and pregnancy. Hybrid laparo-endoscopic treatment in one step. Cirugia Y Cirujanos. 2014;82(2):158-63. PubMed PMID: WOS:000340837100010.	Excluded from the final analysis	The article was excluded from the analysis due to the lack of relation between the title and the abstract of the article with the subject under study
210	Harrison EC, Roschke EJ, Meyers HI, Edmiston WA, Chan LS, Tatter D, Lau FYK. CHOLELITHIASIS - FREQUENT COMPLICATION OF ARTIFICIAL HEART-VALVE REPLACEMENT. American Heart Journal. 1978;95(4):483-8. doi: 10.1016/0002-8703(78)90240-5. PubMed PMID: WOS:A1978ET44600010.	Excluded from the final analysis	The article was excluded from the analysis due to the lack of relation between the title and the abstract of the article with the subject under study
211	Fry RE, Fry WJ. Cholelithiasis and aortic reconstruction: the problem of simultaneous surgical therapy. Conclusions from a personal series. J Vasc Surg. 1986;4(4):345-50. PubMed PMID: 3761476.	Excluded from the final analysis	The article was excluded from the analysis due to the lack of relation between the title and the abstract of the article with the subject under study

212	Koller T, Kollerova J, Hlavaty T, Huorka M, Payer J. Cholelithiasis and markers of nonalcoholic fatty liver disease in patients with metabolic risk factors. Scand J Gastroenterol. 2012;47(2):197-203. Epub 20111219. doi: 10.3109/00365521.2011.643481. PubMed PMID: 22182015.	Excluded from the final analysis	The article was excluded from the analysis due to the lack of relation between the title and the abstract of the article with the subject under study
213	Kargl S, Gitter R, Pumberger W. Cholelithiasis in children with CHD: is it a problem? Cardiology in the Young. 2017;27(4):630-3. doi: 10.1017/s1047951116000846. PubMed PMID: WOS:000402409100003.	Excluded from the final analysis	The article was excluded from the analysis due to the lack of relation between the title and the abstract of the article with the subject under study
214	Butt Z, Hyder Q. Cholelithiasis in hepatic cirrhosis: evaluating the role of risk factors. J Pak Med Assoc. 2010;60(8):641-4. PubMed PMID: 20726194.	Excluded from the final analysis	The article was excluded from the analysis due to the lack of relation between the title and the abstract of the article with the subject under study
215	Wang HH, Garruti G, Liu M, Portincasa P, Wang DQH. Cholesterol and Lipoprotein Metabolism and Atherosclerosis: Recent Advances in Reverse Cholesterol Transport. Annals of Hepatology. 2017;16:S27-S42. doi: 10.5604/01.3001.0010.5495. PubMed PMID: WOS:000460734300005.	Excluded from the final analysis	The article was excluded from the analysis due to the lack of relation between the title and the abstract of the article with the subject under study
216	Tsibranska-Gyoreva S, Petkov V, Katev V, Krastev D, Vinarov Z, Tcholakova S. Cholesterol solubilization: Interplay between phytosterols, saponins and lipid digestion products. Colloids and Surfaces a-Physicochemical and Engineering Aspects. 2023;662. doi: 10.1016/j.colsurfa.2023.131052. PubMed PMID: WOS:000999603900001.	Excluded from the final analysis	The article was excluded from the analysis due to the lack of relation between the title and the abstract of the article with the subject under study

217	Wolff G, Liebscher C, Orban U. [Chronic gastritis and serum lipids, hypertension and cholelithiasis]. Dtsch Z Verdau Stoffwechselkr. 1988;48(3-4):149-52. PubMed PMID: 3234298.	Excluded from the final analysis	The article was excluded from the analysis due to the lack of relation between the title and the abstract of the article with the subject under study
218	Kudva MV, Zawawi MM. Chronic liver disease in Kuala Lumpur, Malaysia: a clinical study. Singapore Med J. 1990;31(4):368-73. PubMed PMID: 2255936.	Excluded from the final analysis	The article was excluded from the analysis due to the lack of relation between the title and the abstract of the article with the subject under study
219	Molero X, Ayuso JR, Balsells J, Boadas J, Busquets J, Casteras A, et al. Chronic pancreatitis for the clinician: complications and special forms of the disease. Interdisciplinary position paper of the Catalan Society of Digestology (SCD) and the Catalan Pancreatic Society (SCPanc). Minerva Gastroenterology. 2024;70(2):208-24. doi: 10.23736/s2724-5985.22.03127-8. PubMed PMID: WOS:001245623600008.	Excluded from the final analysis	The article was excluded from the analysis due to the lack of relation between the title and the abstract of the article with the subject under study
220	Dong YH, Lu YF, Qiu Q, Huang H, Wei H. Clinical analysis of pericardial devascularization by preserving vagus trunks in 42 patients with portal hypertension. Asian J Surg. 2004;27(2):108-13. doi: 10.1016/s1015-9584(09)60322-5. PubMed PMID: 15140661.	Excluded from the final analysis	The article was excluded from the analysis due to the lack of relation between the title and the abstract of the article with the subject under study
221	Baranskaya EK, Ivashkin VT. The clinical features of gallstone disease in heart failure caused by rheumatic heart diseases. Gastroenterology. 2001;120(5):A388-A. PubMed PMID: WOS:000168514701920.	Excluded from the final analysis	The article was excluded from the analysis due to the lack of relation between the title and the abstract of the article with the subject under study

222	Haffner SM, Diehl AK, Valdez R, Mitchell BD, Hazuda HP, Morales P, Stern MP. CLINICAL GALLBLADDER-DISEASE IN NIDDM SUBJECTS - RELATIONSHIP TO DURATION OF DIABETES AND SEVERITY OF GLYCEMIA. Diabetes Care. 1993;16(9):1276-84. doi: 10.2337/diacare.16.9.1276. PubMed PMID: WOS:A1993LU54900009.	Excluded from the final analysis	The article was excluded from the analysis due to the lack of relation between the title and the abstract of the article with the subject under study
223	Conn HO. A clinical hepatologist's predictions about non-absorbed carbohydrates for the early twenty-first century. Scand J Gastroenterol Suppl. 1997;222:88-92. doi: 10.1080/00365521.1997.11720727. PubMed PMID: 9145456.	Excluded from the final analysis	The article was excluded from the analysis due to the lack of relation between the title and the abstract of the article with the subject under study
224	Jeanty C, Derderian SC, Courtier J, Hirose S. Clinical management of infantile cholelithiasis. Journal of Pediatric Surgery. 2015;50(8):1289-92. doi: 10.1016/j.jpedsurg.2014.10.051. PubMed PMID: WOS:000360121000009.	Excluded from the final analysis	The article was excluded from the analysis due to the lack of relation between the title and the abstract of the article with the subject under study
225	Lodha M, Chauhan AS, Puranik A, Meena SP, Badkur M, Chaudhary R, et al. Clinical Profile and Evaluation of Outcomes of Symptomatic Gallstone Disease in the Senior Citizen Population. Cureus. 2022;14(8):e28492. Epub 20220828. doi: 10.7759/cureus.28492. PubMed PMID: 36185904; PubMed Central PMCID: PMCPMC9513743.	Excluded from the final analysis	The article was excluded from the analysis due to the lack of relation between the title and the abstract of the article with the subject under study
226	Finucci G, Tirelli M, Bellon S, Zambon M, Toffolo L, Merkel C, Zuin R. Clinical significance of cholelithiasis in patients with decompensated cirrhosis. J Clin Gastroenterol. 1990;12(5):538-41. doi: 10.1097/00004836-199010000-00010. PubMed PMID: 2229996.	Excluded from the final analysis	The article was excluded from the analysis due to the lack of relation between the title and the abstract of the article with the subject under study

227	Grace RF, Bianchi P, van Beers EJ, Eber SW, Glader B, Yaish HM, et al. Clinical spectrum of pyruvate kinase deficiency: data from the Pyruvate Kinase Deficiency Natural History Study. Blood. 2018;131(20):2183-92. Epub 20180316. doi: 10.1182/blood-2017-10-810796. PubMed PMID: 29549173.	Excluded from the final analysis	The article was excluded from the analysis due to the lack of relation between the title and the abstract of the article with the subject under study
228	Zhou HB, Yin SN, He YJ, Liu A, Chen AP, Ke S. A clinical study of an indwelling nasobiliary duct placed in a trans-abdominal forward direction without cutting the sphincter of Oddi to treat early acute biliary pancreatitis. International Journal of Clinical and Experimental Medicine. 2020;13(1):310-6. PubMed PMID: WOS:000518396500038.	Excluded from the final analysis	The article was excluded from the analysis due to the lack of relation between the title and the abstract of the article with the subject under study
229	Li W, Tao J, Song XG, Hou MR, Qu K, Gu JT, et al. Clinical study of extrahepatic biliary adenoma. World Journal of Clinical Cases. 2023;11(24):5692-9. doi: 10.12998/wjcc.v11.i24.5692. PubMed PMID: WOS:001081844100007.	Excluded from the final analysis	The article was excluded from the analysis due to the lack of relation between the title and the abstract of the article with the subject under study
230	Napolitano L, Waku M, Di Bartolomeo N, Aceto L, Innocenti P. [Clinical study on laparoscopic approach to cholelithiasis in the elderly]. G Chir. 2004;25(8-9):301-3. PubMed PMID: 15560307.	Excluded from the final analysis	The article was excluded from the analysis due to the lack of relation between the title and the abstract of the article with the subject under study
231	Cucchiaro G, Rossitch JC, Bowie J, Branum GD, Niotis MT, Watters CR, Meyers WC. CLINICAL-SIGNIFICANCE OF ULTRASONOGRAPHICALLY DETECTED COINCIDENTAL GALLSTONES. Digestive Diseases and Sciences. 1990;35(4):417-21. doi: 10.1007/bf01536912. PubMed PMID: WOS:A1990CY65400001.	Excluded from the final analysis	The article was excluded from the analysis due to the lack of relation between the title and the abstract of the article with the subject under study

232	Oliver M. The clofibrate saga: a retrospective commentary. British Journal of Clinical Pharmacology. 2012;74(6):907-10. doi: 10.1111/j.1365-2125.2012.04282.x. PubMed PMID: WOS:000311108800002.	Excluded from the final analysis	The article was excluded from the analysis due to the lack of relation between the title and the abstract of the article with the subject under study
233	Bateson MC, Maclean D, Ross PE, Bouchier IAD. CLOFIBRATE THERAPY AND GALLSTONE INDUCTION. American Journal of Digestive Diseases. 1978;23(7):623-8. doi: 10.1007/bf01072597. PubMed PMID: WOS:A1978FG18200008.	Excluded from the final analysis	The article was excluded from the analysis due to the lack of relation between the title and the abstract of the article with the subject under study
234	Pribis P, Shukitt-Hale B. Cognition: the new frontier for nuts and berries. Am J Clin Nutr. 2014;100 Suppl 1:347s-52s. Epub 20140528. doi: 10.3945/ajcn.113.071506. PubMed PMID: 24871475.	Excluded from the final analysis	The article was excluded from the analysis due to the lack of relation between the title and the abstract of the article with the subject under study
235	Pribis P, Shukitt-Hale B. Cognition: the new frontier for nuts and berries. American Journal of Clinical Nutrition. 2014;100(1):347S-52S. doi: 10.3945/ajcn.113.071506. PubMed PMID: WOS:000337862200006.	Excluded from the final analysis	The article was excluded from the analysis due to the lack of relation between the title and the abstract of the article with the subject under study
236	Harooni J, Joukar F, Goujani R, Sikaroudi MK, Hatami A, Zolghadrpour MA, et al. Cohort profile: the PERSIAN Dena Cohort Study (PDCS) of non-communicable diseases in Southwest Iran. BMJ Open. 2024;14(4):e079697. Epub 20240410. doi: 10.1136/bmjopen-2023-079697. PubMed PMID: 38604628; PubMed Central PMCID: PMCPMC11015286.	Excluded from the final analysis	The article was excluded from the analysis due to the lack of relation between the title and the abstract of the article with the subject under study

237	Gavriilidis P, Paily A. Colonic Perforation Secondary to Gallstone Impaction in the Sigmoid Colon. Case Rep Surg. 2023;2023:9986665. Epub 20230720. doi: 10.1155/2023/9986665. PubMed PMID: 37521369; PubMed Central PMCID: PMCPMC10374380.	Excluded from the final analysis	The article was excluded from the analysis due to the lack of relation between the title and the abstract of the article with the subject under study
238	Gaitini D, Kreitenberg AJ, Fischer D, Maza I, Chowers Y. Color-coded duplex sonography compared to multidetector computed tomography for the diagnosis of crohn disease relapse and complications. J Ultrasound Med. 2011;30(12):1691-9. doi: 10.7863/jum.2011.30.12.1691. PubMed PMID: 22124005.	Excluded from the final analysis	The article was excluded from the analysis due to the lack of relation between the title and the abstract of the article with the subject under study
239	Tokur O, Aydin S, Kantarci M. Commentary on "Gallstone associated celiac trunk thromboembolisms complicated with splenic infarction: A case report". World Journal of Clinical Cases. 2022;10(32):12059-61. doi: 10.12998/wjcc.v10.i32.12059. PubMed PMID: WOS:000886696700045.	Excluded from the final analysis	The article was excluded from the analysis due to the lack of relation between the title and the abstract of the article with the subject under study
240	Tokur O, Aydın S, Kantarci M. Commentary on "Gallstone associated celiac trunk thromboembolisms complicated with splenic infarction: A case report". World J Clin Cases. 2022;10(32):12059-61. doi: 10.12998/wjcc.v10.i32.12059. PubMed PMID: 36405298; PubMed Central PMCID: PMCPMC9669843.	Excluded from the final analysis	The article was excluded from the analysis due to the lack of relation between the title and the abstract of the article with the subject under study
241	Leppaniemi A, Wherry D, Pikoulis E, Hufnagel H, Waasdorp C, Fishback N, Rich N. Common bile duct repair with titanium staples - Comparison with suture closure. Surgical Endoscopy-Ultrasound and Interventional Techniques. 1997;11(7):714-7. doi: 10.1007/s004649900434. PubMed PMID: WOS:A1997XH52300004.	Excluded from the final analysis	The article was excluded from the analysis due to the lack of relation between the title and the abstract of the article with the subject under study

242	Stefano GB, Miller J. Communication between animal cells and the plant foods they ingest: phyto-zooidal dependencies and signaling (Review). Int J Mol Med. 2002;10(4):413-21. PubMed PMID: 12239587.	Excluded from the final analysis	The article was excluded from the analysis due to the lack of relation between the title and the abstract of the article with the subject under study
243	Liang J, Jiang Y, Huang Y, Huang Y, Liu F, Zhang Y, et al. Comorbidities and factors influencing frequent gout attacks in patients with gout: a cross-sectional study. Clin Rheumatol. 2021;40(7):2873-80. Epub 20210204. doi: 10.1007/s10067-021-05595-w. PubMed PMID: 33538925.	Excluded from the final analysis	The article was excluded from the analysis due to the lack of relation between the title and the abstract of the article with the subject under study
244	Sommerfleck F, Schneeberger E, Citera G. Comorbidities in Argentine patients with axial spondyloarthritis: Is nephrolithiasis associated with this disease? Eur J Rheumatol. 2018;5(3):169-72. Epub 20180622. doi: 10.5152/eurjrheum.2018.18002. PubMed PMID: 30071942; PubMed Central PMCID: PMCPMC6116842.	Excluded from the final analysis	The article was excluded from the analysis due to the lack of relation between the title and the abstract of the article with the subject under study
245	Marthandam S, Gunjiganvi M, Jasthi S, Atluri R, Reddy YS, Martandam V. A Comparative Study of Laparoscopic Versus Robotic Cholecystectomies Based on the Parkland Grading Scale. Cureus Journal of Medical Science. 2024;16(9). doi: 10.7759/cureus.68523. PubMed PMID: WOS:001309939400004.	Excluded from the final analysis	The article was excluded from the analysis due to the lack of relation between the title and the abstract of the article with the subject under study
246	Mohammadzade AR, Esmaili F. Comparing Hemodynamic Symptoms and the Level of Abdominal Pain in High- Versus Low-Pressure Carbon Dioxide in Patients Undergoing Laparoscopic Cholecystectomy. Indian Journal of Surgery. 2018;80(1):30-5. doi: 10.1007/s12262-016-1552-4. PubMed PMID: WOS:000428377600007.	Excluded from the final analysis	The article was excluded from the analysis due to the lack of relation between the title and the abstract of the article with the subject under study

247	Kaura SH, Haghighi M, Matza BW, Hajdu CH, Rosenkrantz AB. Comparison of CT and MRI findings in the differentiation of acute from chronic cholecystitis. Clin Imaging. 2013;37(4):687-91. Epub 20130328. doi: 10.1016/j.clinimag.2013.02.009. PubMed PMID: 23541278.	Excluded from the final analysis	The article was excluded from the analysis due to the lack of relation between the title and the abstract of the article with the subject under study
248	Kara YB, Ozel Y. Comparison of the Grades of Fatty Liver Disease With Perioperative Risk Factors in Patients With Laparoscopic Sleeve Gastrectomy. Cureus. 2024;16(9):e69717. Epub 20240919. doi: 10.7759/cureus.69717. PubMed PMID: 39429298; PubMed Central PMCID: PMCPMC11490200.	Excluded from the final analysis	The article was excluded from the analysis due to the lack of relation between the title and the abstract of the article with the subject under study
249	Anwar S, Malik AAR, Hamza A, Shahid MS, Subhan M, Bibi R. A Complex Case of Obstructive Jaundice in a Septuagenarian: Diagnostic Challenges and Therapeutic Strategies. Cureus Journal of Medical Science. 2024;16(7). doi: 10.7759/cureus.64598. PubMed PMID: WOS:001271275700030.	Excluded from the final analysis	The article was excluded from the analysis due to the lack of relation between the title and the abstract of the article with the subject under study
250	Anwar S, Rasool Malik AA, Hamza A, Shahid MS, Subhan M, Bibi R. A Complex Case of Obstructive Jaundice in a Septuagenarian: Diagnostic Challenges and Therapeutic Strategies. Cureus. 2024;16(7):e64598. Epub 20240715. doi: 10.7759/cureus.64598. PubMed PMID: 39149640; PubMed Central PMCID: PMCPMC11325256.	Excluded from the final analysis	The article was excluded from the analysis due to the lack of relation between the title and the abstract of the article with the subject under study
251	Kulkarni SS, Hotta M, Sher L, Selby RR, Parekh D, Buxbaum J, Stapfer M. Complicated gallstone disease: diagnosis and management of Mirizzi syndrome. Surgical Endoscopy and Other Interventional Techniques. 2017;31(5):2215-22. doi: 10.1007/s00464-016-5219-9. PubMed PMID: WOS:000400552300021.	Excluded from the final analysis	The article was excluded from the analysis due to the lack of relation between the title and the abstract of the article with the subject under study

252	Cabrerizo L, Rubio MA, Ballesteros MD, Lopera CM. Complications associated with obesity. Revista Espanola De Nutricion Comunitaria-Spanish Journal of Community Nutrition. 2008;14(3):156-62. PubMed PMID: WOS:000263496600004.	Excluded from the final analysis	The article was excluded from the analysis due to the lack of relation between the title and the abstract of the article with the subject under study
253	Thorbjarnarson B, Glenn F. COMPLICATIONS OF BILIARY TRACT SURGERY. Surg Clin North Am. 1964;44:431-7. doi: 10.1016/s0039-6109(16)37240-1. PubMed PMID: 14180331.	Excluded from the final analysis	The article was excluded from the analysis due to the lack of relation between the title and the abstract of the article with the subject under study
254	Feng Y, Jin WS, Xiang DB, Sun GY, Dai HG. Complications of Laparoscopic Cholecystectomy and its Prevention: A Review and Experience of 400 Cases. Hepato-Gastroenterology. 2012;59(113):47-50. doi: 10.5754/hge11232. PubMed PMID: WOS:000301397300011.	Excluded from the final analysis	The article was excluded from the analysis due to the lack of relation between the title and the abstract of the article with the subject under study
255	Triantafyllidis I, Nikoloudis N, Sapidis N, Chrissidou M, Kalaitsidou I, Chrissidis T. Complications of Laparoscopic Cholecystectomy: Our Experience in a District General Hospital. Surgical Laparoscopy Endoscopy & Percutaneous Techniques. 2009;19(6):449-58. doi: 10.1097/SLE.0b013e3181bd8f6d. PubMed PMID: WOS:000273126800008.	Excluded from the final analysis	The article was excluded from the analysis due to the lack of relation between the title and the abstract of the article with the subject under study
256	Keltikangas-Järvinen L. Concept of alexithymia. I. The prevalence of alexithymia in psychosomatic patients. Psychother Psychosom. 1985;44(3):132-8. doi: 10.1159/000287904. PubMed PMID: 3832145.	Excluded from the final analysis	The article was excluded from the analysis due to the lack of relation between the title and the abstract of the article with the subject under study

257	Simon-Vermot I, Keller U. [Consequences and complications of obesity]. Ther Umsch. 2000;57(8):493-7. doi: 10.1024/0040-5930.57.8.493. PubMed PMID: 11026085.	Excluded from the final analysis	The article was excluded from the analysis due to the lack of relation between the title and the abstract of the article with the subject under study
258	Udekwu PO, Sullivan WG. Contemporary experience with cholecystectomy: establishing 'benchmarks' two decades after the introduction of laparoscopic cholecystectomy. Am Surg. 2013;79(12):1253-7. PubMed PMID: 24351351.	Excluded from the final analysis	The article was excluded from the analysis due to the lack of relation between the title and the abstract of the article with the subject under study
259	Udekwu PO, Sullivan WG. Contemporary Experience with Cholecystectomy: Establishing 'Benchmarks' Two Decades after the Introduction of Laparoscopic Cholecystectomy. American Surgeon. 2013;79(12):1253-7. PubMed PMID: WOS:000336964500015.	Excluded from the final analysis	The article was excluded from the analysis due to the lack of relation between the title and the abstract of the article with the subject under study
260	Rappaport WD, Gordon P, Warneke JA, Neal D, Hunter GC. Contraindications and complications of laparoscopic cholecystectomy. Am Fam Physician. 1994;50(8):1707-11, 14. PubMed PMID: 7977000.	Excluded from the final analysis	The article was excluded from the analysis due to the lack of relation between the title and the abstract of the article with the subject under study
261	Rappaport WD, Gordon P, Warneke JA, Neal D, Hunter GC. CONTRAINDICATIONS AND COMPLICATIONS OF LAPAROSCOPIC CHOLECYSTECTOMY. American Family Physician. 1994;50(8):1707-&. PubMed PMID: WOS:A1994PV98400012.	Excluded from the final analysis	The article was excluded from the analysis due to the lack of relation between the title and the abstract of the article with the subject under study

262	López R, Oyarzún M, Naranjo C, Cumsille F, Ortiz M, Baelum V. Coronary heart disease and periodontitis -: a case control study in Chilean adults. Journal of Clinical Periodontology. 2002;29(5):468-73. doi: 10.1034/j.1600-051X.2002.290513.x. PubMed PMID: WOS:000176702100013.	Excluded from the final analysis	The article was excluded from the analysis due to the lack of relation between the title and the abstract of the article with the subject under study
263	Diehl AK, Haffner SM, Hazuda HP, Stern MP. Coronary risk factors and clinical gallbladder disease: an approach to the prevention of gallstones? Am J Public Health. 1987;77(7):841-5. doi: 10.2105/ajph.77.7.841. PubMed PMID: 3496013; PubMed Central PMCID: PMCPMC1647214.	Excluded from the final analysis	The article was excluded from the analysis due to the lack of relation between the title and the abstract of the article with the subject under study
264	Hadi A, Werge M, Kristiansen KT, Pedersen UG, Karstensen JG, Novovic S, Gluud LL. Coronavirus Disease-19 (COVID-19) associated with severe acute pancreatitis: Case report on three family members. Pancreatology. 2020;20(4):665-7. Epub 20200505. doi: 10.1016/j.pan.2020.04.021. PubMed PMID: 32387082; PubMed Central PMCID: PMCPMC7199002.	Excluded from the final analysis	The article was excluded from the analysis due to the lack of relation between the title and the abstract of the article with the subject under study
265	Akoudad S, Szklo M, McAdams MA, Fulop T, Anderson CA, Coresh J, Köttgen A. Correlates of kidney stone disease differ by race in a multi-ethnic middle-aged population: the ARIC study. Prev Med. 2010;51(5):416-20. Epub 20100827. doi: 10.1016/j.ypmed.2010.08.011. PubMed PMID: 20801154; PubMed Central PMCID: PMCPMC2964449.	Excluded from the final analysis	The article was excluded from the analysis due to the lack of relation between the title and the abstract of the article with the subject under study
266	Segal L, Carter R, Zimmet P. The cost of obesity: the Australian perspective. Pharmacoeconomics. 1994;5(Suppl 1):45-52. doi: 10.2165/00019053-199400051-00009. PubMed PMID: 10147249.	Excluded from the final analysis	The article was excluded from the analysis due to the lack of relation between the title and the abstract of the article with the subject under study

267	Kumar M, Kaushik D, Kaur J, Proestos C, Oz F, Oz E, et al. A Critical Review on Obesity: Herbal Approach, Bioactive Compounds, and Their Mechanism. Applied Sciences-Basel. 2022;12(16). doi: 10.3390/app12168342. PubMed PMID: WOS:000846120800001.	Excluded from the final analysis	The article was excluded from the analysis due to the lack of relation between the title and the abstract of the article with the subject under study
268	Sasaki H, Tanaka H, Nio M. Current management of long-term survivors of biliary atresia: over 40 years of experience in a single center and review of the literature. Pediatr Surg Int. 2017;33(12):1327-33. Epub 20170927. doi: 10.1007/s00383-017-4163-7. PubMed PMID: 28956136.	Excluded from the final analysis	The article was excluded from the analysis due to the lack of relation between the title and the abstract of the article with the subject under study
269	Mun EC, Blackburn GL, Matthews JB. Current status of medical and surgical therapy for obesity. Gastroenterology. 2001;120(3):669-81. doi: 10.1053/gast.2001.22430. PubMed PMID: 11179243.	Excluded from the final analysis	The article was excluded from the analysis due to the lack of relation between the title and the abstract of the article with the subject under study
270	Ping WW, Puvan IS. Current status of oral contraceptive. Med J Malaysia. 1976;30(3):173-7. PubMed PMID: 822261.	Excluded from the final analysis	The article was excluded from the analysis due to the lack of relation between the title and the abstract of the article with the subject under study
271	Tiseo D, Borrelli F, Gentile I, Benassai G, Quarto G, Borgia G. [Cystic echinococcosis in humans: our clinic experience]. Parassitologia. 2004;46(1-2):45-51. PubMed PMID: 15305685.	Excluded from the final analysis	The article was excluded from the analysis due to the lack of relation between the title and the abstract of the article with the subject under study

272	Cucchiaro G, Watters CR, Rossitch JC, Meyers WC. DEATHS FROM GALLSTONES - INCIDENCE AND ASSOCIATED CLINICAL FACTORS. Annals of Surgery. 1989;209(2):149-51. doi: 10.1097/00000658-198902000-00002. PubMed PMID: WOS:A1989T253400002.	Excluded from the final analysis	The article was excluded from the analysis due to the lack of relation between the title and the abstract of the article with the subject under study
273	Birkmeyer JD, Birkmeyer NO. Decision analysis in surgery. Surgery. 1996;120(1):7-15. doi: 10.1016/s0039-6060(96)80234-0. PubMed PMID: WOS:A1996VA23900002.	Excluded from the final analysis	The article was excluded from the analysis due to the lack of relation between the title and the abstract of the article with the subject under study
274	Ikeda S, Kagami T, Tani S, Uotani T, Yamade M, Hamaya Y, et al. Decompressive laparotomy for abdominal compartment syndrome resulting from severe acute pancreatitis: a case report. Bmc Gastroenterology. 2019;19(1). doi: 10.1186/s12876-019-1059-0. PubMed PMID: WOS:000480463700001.	Excluded from the final analysis	The article was excluded from the analysis due to the lack of relation between the title and the abstract of the article with the subject under study
275	Patel AM, Yeola M, Mahakalkar C. Demographic and Risk Factor Profile in Patients of Gallstone Disease in Central India. Cureus. 2022;14(5):e24993. Epub 20220514. doi: 10.7759/cureus.24993. PubMed PMID: 35719762; PubMed Central PMCID: PMCPMC9190443.	Excluded from the final analysis	The article was excluded from the analysis due to the lack of relation between the title and the abstract of the article with the subject under study
276	Zheng Z, Zhang C, Yan J, Ruan Y, Zhao X, San X, et al. Diabetes mellitus is associated with hepatocellular carcinoma: a retrospective case-control study in hepatitis endemic area. PLoS One. 2013;8(12):e84776. Epub 20131226. doi: 10.1371/journal.pone.0084776. PubMed PMID: 24386416; PubMed Central PMCID: PMCPMC3873428.	Excluded from the final analysis	The article was excluded from the analysis due to the lack of relation between the title and the abstract of the article with the subject under study

277	Dumortier J, Vaillant E, Boillot O, Poncet G, Henry L, Scoazec JY, et al. Diagnosis and treatment of biliary obstruction caused by portal cavernoma. Endoscopy. 2003;35(5):446-50. doi: 10.1055/s-2003-38779. PubMed PMID: WOS:000182484600016.	Excluded from the final analysis	The article was excluded from the analysis due to the lack of relation between the title and the abstract of the article with the subject under study
278	Lyon C, Clark DC. Diagnosis of acute abdominal pain in older patients. Am Fam Physician. 2006;74(9):1537-44. PubMed PMID: 17111893.	Excluded from the final analysis	The article was excluded from the analysis due to the lack of relation between the title and the abstract of the article with the subject under study
279	Lyon C, Clark DC. Diagnosis of acute abdominal pain in older patients. American Family Physician. 2006;74(9):1537-44. PubMed PMID: WOS:000241786300011.	Excluded from the final analysis	The article was excluded from the analysis due to the lack of relation between the title and the abstract of the article with the subject under study
280	Li D, Li Y, Wang X, Wu Y, Cui XY, Hu JQ, et al. Diagnosis of myocardial infarction with nonobstructive coronary arteries in a young man in the setting of acute myocardial infarction after endoscopic retrograde cholangiopancreatography: A case report. World Journal of Clinical Cases. 2019;7(19):3062-8. doi: 10.12998/wjcc.v7.i19.3062. PubMed PMID: WOS:000488264700016.	Excluded from the final analysis	The article was excluded from the analysis due to the lack of relation between the title and the abstract of the article with the subject under study
281	Anwer M, Asghar MS, Rahman S, Kadir S, Yasmin F, Mohsin D, et al. Diagnostic Accuracy of Endoscopic Ultrasonography Versus the Gold Standard Endoscopic Retrograde Cholangiopancreatography in Detecting Common Bile Duct Stones. Cureus. 2020;12(12):e12162. Epub 20201219. doi: 10.7759/cureus.12162. PubMed PMID: 33489574; PubMed Central PMCID: PMCPMC7813932.	Excluded from the final analysis	The article was excluded from the analysis due to the lack of relation between the title and the abstract of the article with the subject under study

282	Anwer M, Asghar MS, Rahman S, Kadir S, Yasmin F, Mohsin D, et al. Diagnostic Accuracy of Endoscopic Ultrasonography Versus the Gold Standard Endoscopic Retrograde Cholangiopancreatography in Detecting Common Bile Duct Stones. Cureus Journal of Medical Science. 2020;12(12). doi: 10.7759/cureus.12162. PubMed PMID: WOS:000600139900001.	Excluded from the final analysis	The article was excluded from the analysis due to the lack of relation between the title and the abstract of the article with the subject under study
283	Elkerkary MA, Gbr H, Shaban H. Diagnostic accuracy of intraoperative cholangiography for detection of anatomical variations of the biliary system. Egyptian Journal of Surgery. 2021;40(1):131-9. doi: 10.4103/ejs.ejs_266_20. PubMed PMID: WOS:000656200800017.	Excluded from the final analysis	The article was excluded from the analysis due to the lack of relation between the title and the abstract of the article with the subject under study
284	vanSonnenberg E, Wittich GR, Casola G, Princenthal RA, Hofmann AF, Keightley A, Wing VW. Diagnostic and therapeutic percutaneous gallbladder procedures. Radiology. 1986;160(1):23-6. doi: 10.1148/radiology.160.1.3520647. PubMed PMID: 3520647.	Excluded from the final analysis	The article was excluded from the analysis due to the lack of relation between the title and the abstract of the article with the subject under study
285	Gibadulina IO, Gibadulin NV. [Diagnostic aspects of chronic cholangitis after cholecystectomy]. Eksp Klin Gastroenterol. 2011;(6):68-72. PubMed PMID: 22168082.	Excluded from the final analysis	The article was excluded from the analysis due to the lack of relation between the title and the abstract of the article with the subject under study
286	Pasławski M, Gwizdak J, Złomaniec J. The diagnostic value of different imaging modalities in evaluation of bowel obstruction. Ann Univ Mariae Curie Sklodowska Med. 2004;59(2):268-74. PubMed PMID: 16146091.	Excluded from the final analysis	The article was excluded from the analysis due to the lack of relation between the title and the abstract of the article with the subject under study

287	Debry G. [Diet peculiarities. Vegetarianism, veganism, crudivorism, macrobiotism]. Rev Prat. 1991;41(11):967-72. PubMed PMID: 2063109.	Excluded from the final analysis	The article was excluded from the analysis due to the lack of relation between the title and the abstract of the article with the subject under study
288	Wirth J, Song M, Fung TT, Joshi AD, Tabung FK, Chan AT, et al. Diet-quality scores and the risk of symptomatic gallstone disease: a prospective cohort study of male US health professionals. Int J Epidemiol. 2018;47(6):1938-46. doi: 10.1093/ije/dyy210. PubMed PMID: 30312404; PubMed Central PMCID: PMCPMC6280928.	Excluded from the final analysis	The article was excluded from the analysis due to the lack of relation between the title and the abstract of the article with the subject under study
289	Pawlikowska L, Strautnieks S, Jankowska I, Czubkowski P, Emerick K, Antoniou A, et al. Differences in presentation and progression between severe FIC1 and BSEP deficiencies. J Hepatol. 2010;53(1):170-8. Epub 20100413. doi: 10.1016/j.jhep.2010.01.034. PubMed PMID: 20447715; PubMed Central PMCID: PMCPMC3042805.	Excluded from the final analysis	The article was excluded from the analysis due to the lack of relation between the title and the abstract of the article with the subject under study
290	Arya AK, Bhadada SK, Kumari P, Agrawal K, Mukhopadhyay S, Sarma D, Rao SD. Differences in Primary Hyperparathyroidism Between Pre- and Postmenopausal Women in India. Endocr Pract. 2021;27(7):710-5. Epub 20201229. doi: 10.1016/j.eprac.2020.12.012. PubMed PMID: 33685668.	Excluded from the final analysis	The article was excluded from the analysis due to the lack of relation between the title and the abstract of the article with the subject under study
291	Yang T, Zhong J, Zhang R, Xiao F, Wang Y, Tao H, Hong F. Different types and numbers metabolic abnormalities and risk of gallbladder stone disease in adults. Front Nutr. 2024;11:1443575. Epub 20240909. doi: 10.3389/fnut.2024.1443575. PubMed PMID: 39315007; PubMed Central PMCID: PMCPMC11416965.	Excluded from the final analysis	The article was excluded from the analysis due to the lack of relation between the title and the abstract of the article with the subject under study

292	Teng MS, Yeh KH, Hsu LA, Chou HH, Er LK, Wu SM, Ko YL. Differential Effects of <i>ABCG5/G8</i> Gene Region Variants on Lipid Profile, Blood Pressure Status, and Gallstone Disease History in Taiwan. Genes. 2023;14(3). doi: 10.3390/genes14030754. PubMed PMID: WOS:000958206500001.	Excluded from the final analysis	The article was excluded from the analysis due to the lack of relation between the title and the abstract of the article with the subject under study
293	Malik AM. Difficult laparoscopic cholecystectomies. Is conversion a sensible option? J Pak Med Assoc. 2015;65(7):698-700. PubMed PMID: 26160075.	Excluded from the final analysis	The article was excluded from the analysis due to the lack of relation between the title and the abstract of the article with the subject under study
294	Malik AM. Difficult laparoscopic cholecystectomies. Is conversion a sensible option? Journal of the Pakistan Medical Association. 2015;65(7):698-700. PubMed PMID: WOS:000360442600003.	Excluded from the final analysis	The article was excluded from the analysis due to the lack of relation between the title and the abstract of the article with the subject under study
295	Demircioglu MK, Demircioglu ZG, Önal C, Özler S. Difficult Management of Giant Gallstone Ileus of a Post-cardiopulmonary Resuscitation Patient: A Case Report. Cureus Journal of Medical Science. 2022;14(4). doi: 10.7759/cureus.23911. PubMed PMID: WOS:000785819800023.	Excluded from the final analysis	The article was excluded from the analysis due to the lack of relation between the title and the abstract of the article with the subject under study
296	Smith FC, Gwynn BR. Direct access surgery. Ann R Coll Surg Engl. 1995;77(2):94-6. PubMed PMID: 7793823; PubMed Central PMCID: PMCPMC2502149.	Excluded from the final analysis	The article was excluded from the analysis due to the lack of relation between the title and the abstract of the article with the subject under study

297	Martin S, Tyrrell J, Thomas EL, Bown MJ, Wood AR, Beaumont RN, et al. Disease consequences of higher adiposity uncoupled from its adverse metabolic effects using Mendelian randomisation. Elife. 2022;11. doi: 10.7554/eLife.72452; 10.7554/eLife.72452.sa0; 10.7554/eLife.72452.sa1; 10.7554/eLife.72452.sa2. PubMed PMID: WOS:000751133500001.	Excluded from the final analysis	The article was excluded from the analysis due to the lack of relation between the title and the abstract of the article with the subject under study
298	Martin S, Tyrrell J, Thomas EL, Bown MJ, Wood AR, Beaumont RN, et al. Disease consequences of higher adiposity uncoupled from its adverse metabolic effects using Mendelian randomisation. Elife. 2022;11. Epub 20220125. doi: 10.7554/eLife.72452. PubMed PMID: 35074047; PubMed Central PMCID: PMCPMC8789289.	Excluded from the final analysis	The article was excluded from the analysis due to the lack of relation between the title and the abstract of the article with the subject under study
299	Mets TF. THE DISEASE PATTERN OF ELDERLY MEDICAL PATIENTS IN RWANDA, CENTRAL-AFRICA. Journal of Tropical Medicine and Hygiene. 1993;96(5):291-300. PubMed PMID: WOS:A1993MC77100005.	Excluded from the final analysis	The article was excluded from the analysis due to the lack of relation between the title and the abstract of the article with the subject under study
300	Seftel HC. Diseases in urban and rural Black populations. S Afr Med J. 1977;51(5):121-3. PubMed PMID: 850843.	Excluded from the final analysis	The article was excluded from the analysis due to the lack of relation between the title and the abstract of the article with the subject under study
301	Kim BH, Lee SH, Lee SS, Oh DJ, Chung ES, Lee SJ. Diverticular disease of the colon in Korea. Korean J Intern Med. 1987;2(1):79-83. doi: 10.3904/kjim.1987.2.1.79. PubMed PMID: 3154819; PubMed Central PMCID: PMCPMC4534912.	Excluded from the final analysis	The article was excluded from the analysis due to the lack of relation between the title and the abstract of the article with the subject under study

302	Brodribb AJ, Humphreys DM. Diverticular disease: three studies. Part I--Relation to other disorders and fibre intake. Br Med J. 1976;1(6007):424-5. doi: 10.1136/bmj.1.6007.424. PubMed PMID: 1252774; PubMed Central PMCID: PMCPMC1638933.	Excluded from the final analysis	The article was excluded from the analysis due to the lack of relation between the title and the abstract of the article with the subject under study
303	Starkov Iu G, Strekalovskiĭ VP, Vishnevskiĭ VA, Grigor'ian RS. [Diverticuli of duodenal papillar region and their role in development of choledocholithiasis and strictures of bile and pancreatic ducts]. Khirurgiia (Mosk). 2000;(3):10-3. PubMed PMID: 10761374.	Excluded from the final analysis	The article was excluded from the analysis due to the lack of relation between the title and the abstract of the article with the subject under study
304	Maddu K, Polireddy K, Hsu DR, Hoff C. Do not get stumped: multimodality imaging findings of early and late post-cholecystectomy complications. Emergency Radiology. 2023;30(3):351-62. doi: 10.1007/s10140-023-02131-y. PubMed PMID: WOS:000968250000002.	Excluded from the final analysis	The article was excluded from the analysis due to the lack of relation between the title and the abstract of the article with the subject under study
305	Lai HC, Chang SN, Lin CC, Chen CC, Chou JW, Peng CY, et al. Does diabetes mellitus with or without gallstones increase the risk of gallbladder cancer? Results from a population-based cohort study. J Gastroenterol. 2013;48(7):856-65. Epub 20121016. doi: 10.1007/s00535-012-0683-z. PubMed PMID: 23065035.	Excluded from the final analysis	The article was excluded from the analysis due to the lack of relation between the title and the abstract of the article with the subject under study
306	Azemoto R, Tsuchiya Y, Ai T, Murayama H, Nakagawa Y, Saisho H, Ohto M. Does gallstone formation after open cardiac surgery result only from latent hemolysis by replaced valves? American Journal of Gastroenterology. 1996;91(10):2185-9. PubMed PMID: WOS:A1996VL41800025.	Excluded from the final analysis	The article was excluded from the analysis due to the lack of relation between the title and the abstract of the article with the subject under study

307	Pineño-Flores C, Segura-Sampedro JJ, Morales-Soriano R, González Argente FX. Dorsal inflammatory mass secondary to lost stones after laparoscopic cholecystectomy. Rev Esp Enferm Dig. 2017;109(6):455-6. PubMed PMID: 28597676.	Excluded from the final analysis	The article was excluded from the analysis due to the lack of relation between the title and the abstract of the article with the subject under study
308	Dvoretsky LI. Dr. Sergey P. Botkin's case sheet. Terapevticheskii Arkhiv. 2023;95(7):602-6. doi: 10.26442/00403660.2023.07.202310. PubMed PMID: WOS:001104956400003.	Excluded from the final analysis	The article was excluded from the analysis due to the lack of relation between the title and the abstract of the article with the subject under study
309	Sher PP. Drug interference with laboratory tests: oral contraceptives. Drug Ther (NY). 1977;2(10):61-3. PubMed PMID: 12260155.	Excluded from the final analysis	The article was excluded from the analysis due to the lack of relation between the title and the abstract of the article with the subject under study
310	Mead E, Atkinson G, Richter B, Metzendorf MI, Baur L, Finer N, et al. Drug interventions for the treatment of obesity in children and adolescents. Cochrane Database Syst Rev. 2016;11(11):Cd012436. Epub 20161129. doi: 10.1002/14651858.Cd012436. PubMed PMID: 27899001; PubMed Central PMCID: PMCPMC6472619.	Excluded from the final analysis	The article was excluded from the analysis due to the lack of relation between the title and the abstract of the article with the subject under study
311	Huth K, Burkard M, Goebel T. [Dyslipoproteinemia and diabetes mellitus in a metabolic syndrome]. Fortschr Med. 1992;110(11):200-4. PubMed PMID: 1601361.	Excluded from the final analysis	The article was excluded from the analysis due to the lack of relation between the title and the abstract of the article with the subject under study

312	Cuschieri A, Lezoche E, Morino M, Croce E, Lacy A, Toouli J, et al. EAES multicenter prospective randomized trial comparing two-stage vs single-stage management of patients with gallstone disease and ductal calculi. Surgical Endoscopy-Ultrasound and Interventional Techniques. 1999;13(10):952-7. doi: 10.1007/s004649901145. PubMed PMID: WOS:000082887100001.	Excluded from the final analysis	The article was excluded from the analysis due to the lack of relation between the title and the abstract of the article with the subject under study
313	Schepers NJ, Besselink MGH, van Santvoort HC, Bakker OJ, Bruno MJ, Dutch Pancreatitis Study G. Early management of acute pancreatitis. Best Practice & Research Clinical Gastroenterology. 2013;27(5):727-43. doi: 10.1016/j.bpg.2013.08.007. PubMed PMID: WOS:000327673200009.	Excluded from the final analysis	The article was excluded from the analysis due to the lack of relation between the title and the abstract of the article with the subject under study
314	Hirano T, Manabe T. Effect of short-term pancreatico-biliary duct obstruction with intraductal hypertension on subcellular organelle fragility and pancreatic adenylate energy metabolism in rats: protective effect of a new protease inhibitor, E-3123. Acta Chir Belg. 1994;94(2):80-5. PubMed PMID: 7517090.	Excluded from the final analysis	The article was excluded from the analysis due to the lack of relation between the title and the abstract of the article with the subject under study
315	Hirano T, Manabe T. EFFECT OF SHORT-TERM PANCREATICOBILIARY DUCT OBSTRUCTION WITH INTRADUCTAL HYPERTENSION ON SUBCELLULAR ORGANELLE FRAGILITY AND PANCREATIC ADENYLATE ENERGY-METABOLISM IN RATS - PROTECTIVE EFFECT OF A NEW PROTEASE INHIBITOR, E-3123. Acta Chirurgica Belgica. 1994;(2):80-5. PubMed PMID: WOS:A1994NM78600004.	Excluded from the final analysis	The article was excluded from the analysis due to the lack of relation between the title and the abstract of the article with the subject under study
316	Gelabert RC. Effect of ursodeoxycholic acid combined with statins in hypercholesterolemia treatment: a prospective clinical trial. Revista Clinica Espanola. 2004;204(12):632-5. doi: 10.1016/s0014-2565(04)71566-0. PubMed PMID: WOS:000226818900004.	Excluded from the final analysis	The article was excluded from the analysis due to the lack of relation between the title and the abstract of the article with the subject under study

317	Ostrowska L, Stefańska E, Jastrzebska M, Adamska E, Wujek A, Waszczeniuk M. [Effects of dietary habits modifications on selected metabolic parameters during weight loss in obese persons]. Rocz Panstw Zakl Hig. 2012;63(1):83-90. PubMed PMID: 22642074.	Excluded from the final analysis	The article was excluded from the analysis due to the lack of relation between the title and the abstract of the article with the subject under study
318	Skaaby T, Jorgensen T, Linneberg A. Effects of invitation to participate in health surveys on the incidence of cardiovascular disease: a randomized general population study. International Journal of Epidemiology. 2017;46(2):603-11. doi: 10.1093/ije/dyw311. PubMed PMID: WOS:000402996000049.	Excluded from the final analysis	The article was excluded from the analysis due to the lack of relation between the title and the abstract of the article with the subject under study
319	Baigent C, Landray MJ, Reith C, Emberson J, Wheeler DC, Tomson C, et al. The effects of lowering LDL cholesterol with simvastatin plus ezetimibe in patients with chronic kidney disease (Study of Heart and Renal Protection): a randomised placebo-controlled trial. Lancet. 2011;377(9784):2181-92. doi: 10.1016/s0140-6736(11)60739-3. PubMed PMID: WOS:000292340800031.	Excluded from the final analysis	The article was excluded from the analysis due to the lack of relation between the title and the abstract of the article with the subject under study
320	Stewart L, Grifiss JM, Jarvis GA, Way LW. Elderly patients have more severe biliary infections: influence of complement-killing and induction of TNFalpha production. Surgery. 2008;143(1):103-12. Epub 20071203. doi: 10.1016/j.surg.2007.06.035. PubMed PMID: 18154938.	Excluded from the final analysis	The article was excluded from the analysis due to the lack of relation between the title and the abstract of the article with the subject under study
321	Leandros E, Gomatos IP, Mami P, Kastellanos E, Albanopoulos K, Konstadoulakis MM. Elective laparoscopic cholecystectomy for symptomatic gallstone disease in patients receiving anticoagulant therapy. J Laparoendosc Adv Surg Tech A. 2005;15(4):357-60. doi: 10.1089/lap.2005.15.357. PubMed PMID: 16108736.	Excluded from the final analysis	The article was excluded from the analysis due to the lack of relation between the title and the abstract of the article with the subject under study

322	Filippou DK, Triga A, Rizos S, Grigoriadis E, Shipkov CD, Nissiotis AS. Electrocardiographic changes after laparoscopic cholecystectomy. Folia Med (Plovdiv). 2004;46(4):37-41. PubMed PMID: 15962814.	Excluded from the final analysis	The article was excluded from the analysis due to the lack of relation between the title and the abstract of the article with the subject under study
323	Jehle D, Davis E, Evans T, Harchelroad F, Martin M, Zaiser K, Lucid J. EMERGENCY DEPARTMENT SONOGRAPHY BY EMERGENCY PHYSICIANS. American Journal of Emergency Medicine. 1989;7(6):605-11. doi: 10.1016/0735-6757(89)90283-0. PubMed PMID: WOS:A1989AX60800010.	Excluded from the final analysis	The article was excluded from the analysis due to the lack of relation between the title and the abstract of the article with the subject under study
324	Shishido Y, Fujimoto K, Yano Y, Mitsuoka E, Komatsubara T, Shio S, et al. Emergency surgery for hemobilia due to hepatic artery pseudoaneurysm rupture complicated by Mirizzi syndrome type II: a case report. Bmc Surgery. 2021;21(1). doi: 10.1186/s12893-021-01314-z. PubMed PMID: WOS:000681736000001.	Excluded from the final analysis	The article was excluded from the analysis due to the lack of relation between the title and the abstract of the article with the subject under study
325	Papavramidis TS, Michalopoulos A, Papadopoulos VN, Paramythiotis D, Karadimou V, Kokkinakis H, Fahantidis E. Emphysematous cholecystitis: a case report. Cases J. 2008;1(1):73. Epub 20080807. doi: 10.1186/1757-1626-1-73. PubMed PMID: 18687128; PubMed Central PMCID: PMCPMC2518129.	Excluded from the final analysis	The article was excluded from the analysis due to the lack of relation between the title and the abstract of the article with the subject under study
326	Gonçalves M, Rebelo A, Costa R, Ferreira A. Endoscopic ampullectomy of a giant pedunculated ampullary adenoma. Rev Esp Enferm Dig. 2023;115(8):467-8. doi: 10.17235/reed.2022.9316/2022. PubMed PMID: 36426861.	Excluded from the final analysis	The article was excluded from the analysis due to the lack of relation between the title and the abstract of the article with the subject under study

327	Saraswat VA, Rai P, Kumar T, Mohindra S, Dhiman RK. Endoscopic management of portal cavernoma cholangiopathy: practice, principles and strategy. J Clin Exp Hepatol. 2014;4(Suppl 1):S67-76. Epub 20131022. doi: 10.1016/j.jceh.2013.08.011. PubMed PMID: 25755598; PubMed Central PMCID: PMCPMC4244828.	Excluded from the final analysis	The article was excluded from the analysis due to the lack of relation between the title and the abstract of the article with the subject under study
328	Kim KO, Kim TN, Lee SH. Endoscopic papillary large balloon dilation for the treatment of recurrent bile duct stones in patients with prior sphincterotomy. J Gastroenterol. 2010;45(12):1283-8. Epub 20100716. doi: 10.1007/s00535-010-0284-7. PubMed PMID: 20635102.	Excluded from the final analysis	The article was excluded from the analysis due to the lack of relation between the title and the abstract of the article with the subject under study
329	Vitale GC, Zavaleta CM. Endoscopic retrograde cholangiopancreatography for surgeons. Semin Laparosc Surg. 2003;10(1):19-27. doi: 10.1177/107155170301000105. PubMed PMID: 12695806.	Excluded from the final analysis	The article was excluded from the analysis due to the lack of relation between the title and the abstract of the article with the subject under study
330	Rana SS, Sharma R, Ahmed SU, Gupta R. Endoscopic ultrasound-guided transmural drainage of walled-off pancreatic necrosis in patients with portal hypertension and intra-abdominal collaterals. Indian J Gastroenterol. 2017;36(5):400-4. Epub 20171002. doi: 10.1007/s12664-017-0792-y. PubMed PMID: 28971378.	Excluded from the final analysis	The article was excluded from the analysis due to the lack of relation between the title and the abstract of the article with the subject under study
331	Okudaira K, Kawaguchi A, Inoue T, Hashiguchi K, Tsuzuki Y, Nagao S, et al. Endoscopically removed hepatolithiasis associated with cavernous transformation of the portal vein and antiphospholipid antibody syndrome. Digestive Diseases and Sciences. 2006;51(11):1952-5. doi: 10.1007/s10620-005-9059-z. PubMed PMID: WOS:000242323500011.	Excluded from the final analysis	The article was excluded from the analysis due to the lack of relation between the title and the abstract of the article with the subject under study

332	Martín-Pérez J, Delgado-Plasencia L, Bravo-Gutiérrez A, Lorenzo-Rocha N, Burillo-Putze G, Medina-Arana V. Enterolithotomy and early cholecystectomy, an application of damage control surgery for patients with gallstone ileus. Cirugia Y Cirujanos. 2015;83(2):156-60. doi: 10.1016/j.circir.2015.04.013. PubMed PMID: WOS:000362409600013.	Excluded from the final analysis	The article was excluded from the analysis due to the lack of relation between the title and the abstract of the article with the subject under study
333	Cha BH. Epidemiological Characteristics of Gallbladder Cancer in Jeju Island: A Single-Center, Clinically Based, Age-Sex-Matched, Case-Control Study. Asian Pac J Cancer Prev. 2015;16(18):8451-4. doi: 10.7314/apjcp.2015.16.18.8451. PubMed PMID: 26745100.	Excluded from the final analysis	The article was excluded from the analysis due to the lack of relation between the title and the abstract of the article with the subject under study
334	Li HL, Xu B, Zheng W, Xu WH, Gao J, Shu XO, Xiang YB. [Epidemiological characteristics of obesity and its relation to chronic diseases among middle aged and elderly men]. Zhonghua Liu Xing Bing Xue Za Zhi. 2010;31(4):370-4. PubMed PMID: 20513277.	Excluded from the final analysis	The article was excluded from the analysis due to the lack of relation between the title and the abstract of the article with the subject under study
335	Azadbakht S, Parvaee R, Darabian S. An epidemiological investigation of gallstone disease among patients admitted to Shahid Rahimi teaching hospital in Khorramabad in 2016-2020. Afr Health Sci. 2023;23(2):435-41. doi: 10.4314/ahs.v23i2.50. PubMed PMID: 38223601; PubMed Central PMCID: PMCPMC10782301.	Excluded from the final analysis	The article was excluded from the analysis due to the lack of relation between the title and the abstract of the article with the subject under study
336	Fang JG, Zhu J, Li XJ, Li R, Dai F, Song XM, et al. [Epidemiological survey of prevalence of fatty liver and its risk factors in a general adult population of Shanghai]. Zhonghua Gan Zang Bing Za Zhi. 2005;13(2):83-8. PubMed PMID: 15727689.	Excluded from the final analysis	The article was excluded from the analysis due to the lack of relation between the title and the abstract of the article with the subject under study

337	Diehl AK. EPIDEMIOLOGY AND NATURAL-HISTORY OF GALLSTONE DISEASE. Gastroenterology Clinics of North America. 1991;20(1):1-19. PubMed PMID: WOS:A1991FB57200001.	Excluded from the final analysis	The article was excluded from the analysis due to the lack of relation between the title and the abstract of the article with the subject under study
338	Saw SM, Rajan U. The epidemiology of obesity: a review. Ann Acad Med Singap. 1997;26(4):489-93. PubMed PMID: 9395816.	Excluded from the final analysis	The article was excluded from the analysis due to the lack of relation between the title and the abstract of the article with the subject under study
339	Efremova I, Maslennikov R, Poluektova E, Vasilieva E, Zharikov Y, Suslov A, et al. Epidemiology of small intestinal bacterial overgrowth. World J Gastroenterol. 2023;29(22):3400-21. doi: 10.3748/wjg.v29.i22.3400. PubMed PMID: 37389240; PubMed Central PMCID: PMCPMC10303511.	Excluded from the final analysis	The article was excluded from the analysis due to the lack of relation between the title and the abstract of the article with the subject under study
340	Tomassetti P, Migliori M, Lalli S, Campana D, Tomassetti V, Corinaldesi R. Epidemiology, clinical features and diagnosis of gastroenteropancreatic endocrine tumours. Annals of Oncology. 2001;12:S95-S9. doi: 10.1093/annonc/12.suppl_2.S95. PubMed PMID: WOS:000172485900017.	Excluded from the final analysis	The article was excluded from the analysis due to the lack of relation between the title and the abstract of the article with the subject under study
341	Utian WH. Estrogen replacement in the menopause. Obstet Gynecol Annu. 1979;8:369-91. PubMed PMID: 390456.	Excluded from the final analysis	The article was excluded from the analysis due to the lack of relation between the title and the abstract of the article with the subject under study

342	Porterfield LM. Estrogen replacement therapy. Adv Clin Care. 1991;6(4):5. PubMed PMID: 1854417.	Excluded from the final analysis	The article was excluded from the analysis due to the lack of relation between the title and the abstract of the article with the subject under study
343	Eliakim R, Abulafia O, Sherer DM, Rayburn WF. Estrogen, progesterone and the gastrointestinal tract. Journal of Reproductive Medicine. 2000;45(10):781-8. PubMed PMID: WOS:000090105300001.	Excluded from the final analysis	The article was excluded from the analysis due to the lack of relation between the title and the abstract of the article with the subject under study
344	Lans CA. Ethnomedicines used in Trinidad and Tobago for urinary problems and diabetes mellitus. J Ethnobiol Ethnomed. 2006;2:45. Epub 20061013. doi: 10.1186/1746-4269-2-45. PubMed PMID: 17040567; PubMed Central PMCID: PMCPMC1624823.	Excluded from the final analysis	The article was excluded from the analysis due to the lack of relation between the title and the abstract of the article with the subject under study
345	Sakorafas GH, Tsiotou AG. Etiology and pathogenesis of acute pancreatitis - Current concepts. Journal of Clinical Gastroenterology. 2000;30(4):343-56. doi: 10.1097/00004836-200006000-00002. PubMed PMID: WOS:000087387200002.	Excluded from the final analysis	The article was excluded from the analysis due to the lack of relation between the title and the abstract of the article with the subject under study
346	Husain NE, Hassan AT, Elmadhoun WM, Ahmed MH. Evaluating the safety of Liptruzet (ezetimibe and atorvastatin): what are the potential benefits beyond low-density lipoprotein cholesterol-lowering effect? Expert Opin Drug Saf. 2015;14(9):1445-55. Epub 20150703. doi: 10.1517/14740338.2015.1063613. PubMed PMID: 26134926.	Excluded from the final analysis	The article was excluded from the analysis due to the lack of relation between the title and the abstract of the article with the subject under study

347	Thapar VB, Thapar PM, Goel R, Agarwalla R, Salvi PH, Nasta AM, et al. Evaluation of 30-day morbidity and mortality of laparoscopic cholecystectomy: a multicenter prospective observational Indian Association of Gastrointestinal Endoscopic Surgeons (IAGES) Study. Surgical Endoscopy and Other Interventional Techniques. 2023;37(4):2611-25. doi: 10.1007/s00464-022-09659-z. PubMed PMID: WOS:000882821900001.	Excluded from the final analysis	The article was excluded from the analysis due to the lack of relation between the title and the abstract of the article with the subject under study
348	Twaddell WS, Lefkowitch J, Berk PD. Evolution from primary biliary cirrhosis to primary biliary cirrhosis/autoimmune hepatitis overlap syndrome. Seminars in Liver Disease. 2008;28(1):128-34. doi: 10.1055/s-2008-1042650. PubMed PMID: WOS:000255838400011.	Excluded from the final analysis	The article was excluded from the analysis due to the lack of relation between the title and the abstract of the article with the subject under study
349	Utter A, Goss F. Exercise and gall bladder function. Sports Med. 1997;23(4):218-27. doi: 10.2165/00007256-199723040-00002. PubMed PMID: 9160479.	Excluded from the final analysis	The article was excluded from the analysis due to the lack of relation between the title and the abstract of the article with the subject under study
350	Utter A, Goss F. Exercise and gall bladder function. Sports Medicine. 1997;23(4):218-27. doi: 10.2165/00007256-199723040-00002. PubMed PMID: WOS:A1997WZ53500002.	Excluded from the final analysis	The article was excluded from the analysis due to the lack of relation between the title and the abstract of the article with the subject under study
351	Molina-Molina E, Lunardi Baccetto R, Wang DQH, de Bari O, Krawczyk M, Portincasa P. Exercising the hepatobiliary-gut axis. The impact of physical activity performance. European Journal of Clinical Investigation. 2018;48(8). doi: 10.1111/eci.12958. PubMed PMID: WOS:000439927600002.	Excluded from the final analysis	The article was excluded from the analysis due to the lack of relation between the title and the abstract of the article with the subject under study

352	Sninsky BC, Sehgal PD, Hinshaw JL, McDermott JC, Nakada SY. Expanding endourology for biliary stone disease: the efficacy of intracorporeal lithotripsy on refractory biliary calculi. J Endourol. 2014;28(7):877-80. Epub 20140411. doi: 10.1089/end.2014.0083. PubMed PMID: 24617771.	Excluded from the final analysis	The article was excluded from the analysis due to the lack of relation between the title and the abstract of the article with the subject under study
353	Williams LR, Flinn WR, Yao JST, Vogelzang RL, Roth M, McCarthy WJ, Bergan JJ. EXTENDED USE OF COMPUTED-TOMOGRAPHY IN THE MANAGEMENT OF COMPLEX AORTIC PROBLEMS - A LEARNING-EXPERIENCE. Journal of Vascular Surgery. 1986;4(3):264-71. doi: 10.1067/mva.1986.avs0040264. PubMed PMID: WOS:A1986D927500011.	Excluded from the final analysis	The article was excluded from the analysis due to the lack of relation between the title and the abstract of the article with the subject under study
354	Sharma AK. External biliary fistula. Trop Gastroenterol. 2001;22(3):163-8. PubMed PMID: 11681114.	Excluded from the final analysis	The article was excluded from the analysis due to the lack of relation between the title and the abstract of the article with the subject under study
355	Ragheb S, Choong CK, Gowland S, Bagshaw PF, Frizelle FA. Extracorporeal shock wave lithotripsy for difficult common bile duct stones: initial New Zealand experience. New Zealand Medical Journal. 2000;113(1117):377-8. PubMed PMID: WOS:000089371100005.	Excluded from the final analysis	The article was excluded from the analysis due to the lack of relation between the title and the abstract of the article with the subject under study
356	Jakobeit C. EXTRACORPOREAL SHOCK-WAVE LITHOTRIPSY IN GALLBLADDER STONE PERFORATION. Deutsche Medizinische Wochenschrift. 1992;117(14):535-8. doi: 10.1055/s-2008-1062344. PubMed PMID: WOS:A1992HM20900003.	Excluded from the final analysis	The article was excluded from the analysis due to the lack of relation between the title and the abstract of the article with the subject under study

357	Bland KI, Jones RS, Maher JW, Cotton PB, Pennell TC, Amerson JR, et al. EXTRACORPOREAL SHOCK-WAVE LITHOTRIPSY OF BILE-DUCT CALCULI - AN INTERIM-REPORT OF THE DORNIER UNITED-STATES BILE-DUCT LITHOTRIPSY PROSPECTIVE-STUDY. Annals of Surgery. 1989;209(6):743-55. doi: 10.1097/00000658-198906000-00012. PubMed PMID: WOS:A1989AA43300012.	Excluded from the final analysis	The article was excluded from the analysis due to the lack of relation between the title and the abstract of the article with the subject under study
358	Solnick JV, Franceschi F, Roccarina D, Gasbarrini A. Extragastric manifestations of <i>Helicobacter pylori</i> infection -: Other <i>Helicobacter</i> species. Helicobacter. 2006;11:46-51. doi: 10.1111/j.1478-405X.2006.00430.x. PubMed PMID: WOS:000241197100009.	Excluded from the final analysis	The article was excluded from the analysis due to the lack of relation between the title and the abstract of the article with the subject under study
359	Bohr URM, Annibale B, Franceschi F, Roccarina D, Gasbarrini A. Extragastric manifestations of <i>Helicobacter pylori</i> infection -: Other <i>Helicobacters</i>. Helicobacter. 2007;12:45-53. doi: 10.1111/j.1523-5378.2007.00533.x. PubMed PMID: WOS:000249021000009.	Excluded from the final analysis	The article was excluded from the analysis due to the lack of relation between the title and the abstract of the article with the subject under study
360	Cho SW, Marsh JW, Fontes PA, Daily MF, Nalesnik M, Tublin M, et al. Extrahepatic portal vein aneurysm - Report of six patients and review of the literature. Journal of Gastrointestinal Surgery. 2008;12(1):145-52. doi: 10.1007/s11605-007-0313-x. PubMed PMID: WOS:000252297100024.	Excluded from the final analysis	The article was excluded from the analysis due to the lack of relation between the title and the abstract of the article with the subject under study
361	Stender S, Frikke-Schmidt R, Nordestgaard BG, Tybjærg-Hansen A. Extreme Bilirubin Levels as a Causal Risk Factor for Symptomatic Gallstone Disease. Jama Internal Medicine. 2013;173(13):1222-8. doi: 10.1001/jamainternmed.2013.6465. PubMed PMID: WOS:000323602100014.	Excluded from the final analysis	The article was excluded from the analysis due to the lack of relation between the title and the abstract of the article with the subject under study

362	Özdaş S, Bozkurt H. Factors Affecting the Development of Gallstones Following Laparoscopic Sleeve Gastrectomy. Obes Surg. 2019;29(10):3174-8. doi: 10.1007/s11695-019-03946-w. PubMed PMID: 31129884.	Excluded from the final analysis	The article was excluded from the analysis due to the lack of relation between the title and the abstract of the article with the subject under study
363	Sun H, Warren J, Yip J, Ji Y, Hao SL, Han W, Ding YC. Factors Influencing Gallstone Formation: A Review of the Literature. Biomolecules. 2022;12(4). doi: 10.3390/biom12040550. PubMed PMID: WOS:000786881000001.	Excluded from the final analysis	The article was excluded from the analysis due to the lack of relation between the title and the abstract of the article with the subject under study
364	Porte RJ, Coerkamp EG, Koumans RKJ. False aneurysm of a hepatic artery branch and a recurrent subphrenic abscess - Two unusual complications after laparoscopic cholecystectomy. Surgical Endoscopy-Ultrasound and Interventional Techniques. 1996;10(2):161-3. PubMed PMID: WOS:A1996TV34100012.	Excluded from the final analysis	The article was excluded from the analysis due to the lack of relation between the title and the abstract of the article with the subject under study
365	Liu W, Hada T, Fukui K, Imanishi H, Matsuoka N, Iwasaki A, Higashino K. Familial hypocholinesterasemia found in a family and a new confirmed mutation. Intern Med. 1997;36(1):9-13. doi: 10.2169/internalmedicine.36.9. PubMed PMID: 9058093.	Excluded from the final analysis	The article was excluded from the analysis due to the lack of relation between the title and the abstract of the article with the subject under study
366	Kasulke RJ, Schein CJ, Gliedman ML. FATAL COMPLICATIONS IN SURGERY FOR BILIARY CALCULI. American Surgeon. 1981;47(11):470-3. PubMed PMID: WOS:A1981MP08500002.	Excluded from the final analysis	The article was excluded from the analysis due to the lack of relation between the title and the abstract of the article with the subject under study

367	Manglik S, Pal C, Basu U, Kapoor L, Narayan P, Dubey SK. Feasibility and Safety of Concomitant Laparoscopic Cholecystectomy With Open-Heart Surgery: A Systematic Review and Our Early Clinical Experience. Cureus Journal of Medical Science. 2024;16(1). doi: 10.7759/cureus.52844. PubMed PMID: WOS:001155883600023.	Excluded from the final analysis	The article was excluded from the analysis due to the lack of relation between the title and the abstract of the article with the subject under study
368	Cherkashchenko NA, Livzan MA, Krolevets TS. Features of the course of gallstone disease in patients with non-alcoholic fatty liver disease. Terapevticheskii Arkhiv. 2020;92(2):48-54. doi: 10.26442/00403660.2020.02.000550. PubMed PMID: WOS:000514833300008.	Excluded from the final analysis	The article was excluded from the analysis due to the lack of relation between the title and the abstract of the article with the subject under study
369	Di Vita G, Sciumè C, Lauria Lauria G, Stella C, Raimondo D, Leo P. [Fibrinolytic system after laparoscopic cholecystectomy]. Minerva Chir. 2000;55(9):587-92. PubMed PMID: 11155471.	Excluded from the final analysis	The article was excluded from the analysis due to the lack of relation between the title and the abstract of the article with the subject under study
370	Yassin MA, Soliman AT, De Sanctis V, Yassin KS, Abdulla MA. Final Height and Endocrine Complications in Patients with β-Thalassemia Intermedia: Our Experience in Non-Transfused Versus Infrequently Transfused Patients and Correlations with Liver Iron Content. Mediterr J Hematol Infect Dis. 2019;11(1):e2019026. Epub 20190501. doi: 10.4084/mjhid.2019.026. PubMed PMID: 31205630; PubMed Central PMCID: PMCPMC6548215.	Excluded from the final analysis	The article was excluded from the analysis due to the lack of relation between the title and the abstract of the article with the subject under study
371	Yassin MA, Soliman AT, De Sanctis V, Yassin KS, Abdulla MAJ. Final Height and Endocrine Complications in Patients with β-Thalassemia Intermedia: Our Experience in Non-Transfused Versus Infrequently Transfused Patients and Correlations with Liver Iron Content. Mediterranean Journal of Hematology and Infectious Diseases. 2019;11. doi: 10.4084/mjhid.2019.026. PubMed PMID: WOS:000466715800002.	Excluded from the final analysis	The article was excluded from the analysis due to the lack of relation between the title and the abstract of the article with the subject under study

372	Trucco G, Chiusa L, Tandoi F, Bertero L. First report of a gallbladder hemangioma coexisting with gallstones: a case report and literature review of a rare finding. BMC Surg. 2022;22(1):128. Epub 20220406. doi: 10.1186/s12893-022-01554-7. PubMed PMID: 35382806; PubMed Central PMCID: PMCPMC8985283.	Excluded from the final analysis	The article was excluded from the analysis due to the lack of relation between the title and the abstract of the article with the subject under study
373	Wang L, Zhang Z, Wang Z, Jiang T. First study on the outcomes of biliopancreatic diversion with duodenal switch in Chinese patients with obesity. Front Surg. 2022;9:934434. Epub 20230106. doi: 10.3389/fsurg.2022.934434. PubMed PMID: 36684353; PubMed Central PMCID: PMCPMC9852535.	Excluded from the final analysis	The article was excluded from the analysis due to the lack of relation between the title and the abstract of the article with the subject under study
374	Bjorklund G, Semenova Y, Pivina L, Costea DO. Follow-up after bariatric surgery: A review. Nutrition. 2020;78. doi: 10.1016/j.nut.2020.110831. PubMed PMID: WOS:000573295600012.	Excluded from the final analysis	The article was excluded from the analysis due to the lack of relation between the title and the abstract of the article with the subject under study
375	Cavigelli A, Dietz V. Follow-up care of para-/tetraplegics: selected aspects in internal medicine. Schweizerische Medizinische Wochenschrift. 2000;130(22):851-60. PubMed PMID: WOS:000087885200007.	Excluded from the final analysis	The article was excluded from the analysis due to the lack of relation between the title and the abstract of the article with the subject under study
376	Pape J, Swart O, Duvenage R. Four-year review of admissions to a South African regional hospital general surgery department. Samj South African Medical Journal. 2019;109(2):122-6. doi: 10.7196/SAMJ.2019.v109i2.13433. PubMed PMID: WOS:000457578200018.	Excluded from the final analysis	The article was excluded from the analysis due to the lack of relation between the title and the abstract of the article with the subject under study

377	Szilvási A, Andrikovics H, Pongracz E, Kalina A, Komlosi Z, Klein I, Tordai A. Frequencies of Four ATP-Binding Cassette Transporter G8 Polymorphisms in Patients with Ischemic Vascular Diseases. Genetic Testing and Molecular Biomarkers. 2010;14(5):667-72. doi: 10.1089/gtmb.2010.0035. PubMed PMID: WOS:000283125400014.	Excluded from the final analysis	The article was excluded from the analysis due to the lack of relation between the title and the abstract of the article with the subject under study
378	Chawla A, Dewan R, Sarin SK. The frequency and influence of gallbladder varices on gallbladder functions in patients with portal hypertension. Am J Gastroenterol. 1995;90(11):2010-4. PubMed PMID: 7485012.	Excluded from the final analysis	The article was excluded from the analysis due to the lack of relation between the title and the abstract of the article with the subject under study
379	Shiina Y, Toyoda T, Kawazoe Y, Tateno S, Shirai T, Wakisaka Y, et al. The Frequency and Risk Factors for Cholelithiasis and Gallstones in Adult Patients With Cyanotic Congenital Heart Disease. Journal of the American College of Cardiology. 2009;53(10):A364-A. PubMed PMID: WOS:000263864201510.	Excluded from the final analysis	The article was excluded from the analysis due to the lack of relation between the title and the abstract of the article with the subject under study
380	Grigor'eva IN, Iamlikhanova A. [Frequency combination of a gallstone disease and diabetes]. Eksp Klin Gastroenterol. 2011;(4):99-102. PubMed PMID: 21916209.	Excluded from the final analysis	The article was excluded from the analysis due to the lack of relation between the title and the abstract of the article with the subject under study
381	Mehmood Y, Al-Swailmi FK, Al-Enazi SA. Frequency of obesity and comorbidities in medical students. Pak J Med Sci. 2016;32(6):1528-32. doi: 10.12669/pjms.326.10492. PubMed PMID: 28083058; PubMed Central PMCID: PMCPMC5216314.	Excluded from the final analysis	The article was excluded from the analysis due to the lack of relation between the title and the abstract of the article with the subject under study

382	Chaturvedi N, Ben-Shlomo Y. From the surgery to the surgeon: does deprivation influence consultation and operation rates? Br J Gen Pract. 1995;45(392):127-31. PubMed PMID: 7772389; PubMed Central PMCID: PMCPMC1239172.	Excluded from the final analysis	The article was excluded from the analysis due to the lack of relation between the title and the abstract of the article with the subject under study
383	Dumitraşcu DL, Pascu O, Drăghici A, Pop S, Dumitraşcu D, Iacob G. [Functional dyspepsia in liver cirrhosis]. Rom J Intern Med. 1996;34(1-2):91-6. PubMed PMID: 8963253.	Excluded from the final analysis	The article was excluded from the analysis due to the lack of relation between the title and the abstract of the article with the subject under study
384	Billa RF, Biwole MS, Juimo AG, Bejanga BI, Blackett K. GALL STONE DISEASE IN AFRICAN PATIENTS WITH SICKLE-CELL-ANEMIA - A PRELIMINARY-REPORT FROM YAOUNDE, CAMEROON. Gut. 1991;32(5):539-41. doi: 10.1136/gut.32.5.539. PubMed PMID: WOS:A1991FL21500020.	Excluded from the final analysis	The article was excluded from the analysis due to the lack of relation between the title and the abstract of the article with the subject under study
385	Wang TF, Hwang SJ, Lee EY, Tsai YT, Lin HC, Li CP, et al. Gall-bladder wall thickening in patients with liver cirrhosis. J Gastroenterol Hepatol. 1997;12(6):445-9. doi: 10.1111/j.1440-1746.1997.tb00464.x. PubMed PMID: 9195402.	Excluded from the final analysis	The article was excluded from the analysis due to the lack of relation between the title and the abstract of the article with the subject under study
386	Snow RE, Vandewater SL. GALL-STONES AND ANAESTHETICS. Can Anaesth Soc J. 1963;10:555-66. doi: 10.1007/bf03002089. PubMed PMID: 14076155.	Excluded from the final analysis	The article was excluded from the analysis due to the lack of relation between the title and the abstract of the article with the subject under study

387	Barker DJP, Gardner MJ, Power C. GALL-STONES AND ISCHEMIC-HEART-DISEASE. British Medical Journal. 1980;280(6215):717-. doi: 10.1136/bmj.280.6215.717. PubMed PMID: WOS:A1980JH94900038.	Excluded from the final analysis	The article was excluded from the analysis due to the lack of relation between the title and the abstract of the article with the subject under study
388	vanSonnenberg E, Casola G, Zakko SF, Varney RR, Cox J, Wittich GR, Hofmann AF. Gallbladder and bile duct stones: percutaneous therapy with primary MTBE dissolution and mechanical methods. Radiology. 1988;169(2):505-9. doi: 10.1148/radiology.169.2.3174999. PubMed PMID: 3174999.	Excluded from the final analysis	The article was excluded from the analysis due to the lack of relation between the title and the abstract of the article with the subject under study
389	Zhu JF. [Gallbladder contractile function in patients with portal hypertension]. Zhonghua Wai Ke Za Zhi. 1993;31(1):34-6. PubMed PMID: 8404340.	Excluded from the final analysis	The article was excluded from the analysis due to the lack of relation between the title and the abstract of the article with the subject under study
390	Hirose Y, Tajima Y, Sakata H, Uekusa T, Kamada K, Ikehara T, et al. Gallbladder hemorrhage associated with segmental arterial mediolysis: a case report. Surgical Case Reports. 2024;10(1). doi: 10.1186/s40792-023-01799-1. PubMed PMID: WOS:001137312300002.	Excluded from the final analysis	The article was excluded from the analysis due to the lack of relation between the title and the abstract of the article with the subject under study
391	Rajvanshi P, Atac BS, Seno R, Gupta S. Gallbladder vasculitis associated with type-1 cryoglobulinemia. Dig Dis Sci. 2001;46(2):296-300. doi: 10.1023/a:1005648715013. PubMed PMID: 11281178.	Excluded from the final analysis	The article was excluded from the analysis due to the lack of relation between the title and the abstract of the article with the subject under study

392	Ionescu DL. [The gallstone and arterial hypertension]. Rev Med Chir Soc Med Nat Iasi. 2001;105(1):101-4. PubMed PMID: 12092132.	Excluded from the final analysis	The article was excluded from the analysis due to the lack of relation between the title and the abstract of the article with the subject under study
393	Wu CY, Su CC, Huang HH, Wang YT, Wang CC. Gallstone associated celiac trunk thromboembolisms complicated with splenic infarction: A case report. World J Clin Cases. 2022;10(25):8968-73. doi: 10.12998/wjcc.v10.i25.8968. PubMed PMID: 36157657; PubMed Central PMCID: PMCPMC9477054.	Excluded from the final analysis	The article was excluded from the analysis due to the lack of relation between the title and the abstract of the article with the subject under study
394	Wu CY, Su CC, Huang HH, Wang YT, Wang CC. Gallstone associated celiac trunk thromboembolisms complicated with splenic infarction: A case report. World Journal of Clinical Cases. 2022;10(25):8968-73. doi: 10.12998/wjcc.v10.i25.8968. PubMed PMID: WOS:000866214000018.	Excluded from the final analysis	The article was excluded from the analysis due to the lack of relation between the title and the abstract of the article with the subject under study
395	Lakshmi MV, Sridharan GV, Butterworth D. GALLSTONE CIRRHOSIS - ARE WE ONLY SEEING THE TIP OF THE ICEBERG. British Journal of Clinical Practice. 1993;47(3):164-5. PubMed PMID: WOS:A1993LP29000017.	Excluded from the final analysis	The article was excluded from the analysis due to the lack of relation between the title and the abstract of the article with the subject under study
396	Lakshmi MV, Sridharan GV, Butterworth D. Gallstone cirrhosis: are we only seeing the tip of the iceberg? Br J Clin Pract. 1993;47(3):164-5. PubMed PMID: 8347447.	Excluded from the final analysis	The article was excluded from the analysis due to the lack of relation between the title and the abstract of the article with the subject under study

397	Georgescu D, Ionita I, Lascu A, Hut EF, Dragan S, Ancusa OE, et al. Gallstone Disease and Bacterial Metabolic Performance of Gut Microbiota in Middle-Aged and Older Patients. Int J Gen Med. 2022;15:5513-31. Epub 20220608. doi: 10.2147/ijgm.S350104. PubMed PMID: 35702368; PubMed Central PMCID: PMCPMC9188808.	Excluded from the final analysis	The article was excluded from the analysis due to the lack of relation between the title and the abstract of the article with the subject under study
398	Targher G, Byrne CD. Gallstone Disease and Increased Risk of Ischemic Heart Disease Causal Association or Epiphenomenon? Arteriosclerosis Thrombosis and Vascular Biology. 2015;35(10):2073-5. doi: 10.1161/atvbaha.115.306339. PubMed PMID: WOS:000361610700001.	Excluded from the final analysis	The article was excluded from the analysis due to the lack of relation between the title and the abstract of the article with the subject under study
399	Zheng Y, Xu M, Heianza Y, Ma W, Wang T, Sun D, et al. Gallstone disease and increased risk of mortality: Two large prospective studies in US men and women. J Gastroenterol Hepatol. 2018;33(11):1925-31. Epub 20180527. doi: 10.1111/jgh.14264. PubMed PMID: 29671893; PubMed Central PMCID: PMCPMC9015210.	Excluded from the final analysis	The article was excluded from the analysis due to the lack of relation between the title and the abstract of the article with the subject under study
400	Zheng Y, Xu M, Heianza Y, Ma WJ, Wang TG, Sun DJY, et al. Gallstone disease and increased risk of mortality: Two large prospective studies in US men and women. Journal of Gastroenterology and Hepatology. 2018;33(11):1925-31. doi: 10.1111/jgh.14264. PubMed PMID: WOS:000447150900020.	Excluded from the final analysis	The article was excluded from the analysis due to the lack of relation between the title and the abstract of the article with the subject under study
401	Shabanzadeh DM, Sorensen LT, Jorgensen T. Gallstone disease and mortality: a cohort study. International Journal of Public Health. 2017;62(3):353-60. doi: 10.1007/s00038-016-0916-7. PubMed PMID: WOS:000398821500004.	Excluded from the final analysis	The article was excluded from the analysis due to the lack of relation between the title and the abstract of the article with the subject under study

402	Su W, Zhu JG, Li WP, Chen H, Li HW. Gallstone disease and the risk of cardiac mortality in patients with acute coronary syndrome. Frontiers in Cardiovascular Medicine. 2022;9. doi: 10.3389/fcvm.2022.1033959. PubMed PMID: WOS:000894107000001.	Excluded from the final analysis	The article was excluded from the analysis due to the lack of relation between the title and the abstract of the article with the subject under study
403	Upala S, Sanguankeo A, Jaruvongvanich V. GALLSTONE DISEASE AND THE RISK OF CARDIOVASCULAR DISEASE: A SYSTEMATIC REVIEW AND META-ANALYSIS OF OBSERVATIONAL STUDIES. Scandinavian Journal of Surgery. 2017;106(1):21-7. doi: 10.1177/1457496916650998. PubMed PMID: WOS:000398818200002.	Excluded from the final analysis	The article was excluded from the analysis due to the lack of relation between the title and the abstract of the article with the subject under study
404	Lv J, Qi L, Yu CQ, Guo Y, Bian Z, Chen YP, et al. Gallstone Disease and the Risk of Ischemic Heart Disease. Arteriosclerosis Thrombosis and Vascular Biology. 2015;35(10):2232-7. doi: 10.1161/atvbaha.115.306043. PubMed PMID: WOS:000361610700020.	Excluded from the final analysis	The article was excluded from the analysis due to the lack of relation between the title and the abstract of the article with the subject under study
405	Wei CY, Chung TC, Chen CH, Lin CC, Sung FC, Chung WT, et al. Gallstone disease and the risk of stroke: a nationwide population-based study. J Stroke Cerebrovasc Dis. 2014;23(7):1813-20. Epub 20140621. doi: 10.1016/j.jstrokecerebrovasdis.2014.04.024. PubMed PMID: 24957305.	Excluded from the final analysis	The article was excluded from the analysis due to the lack of relation between the title and the abstract of the article with the subject under study
406	Wang JY, Lu FH, Sun ZJ, Wu JS, Yang YC, Lee CT, Chang CJ. Gallstone disease associated with increased risk of arterial stiffness in a Taiwanese population. J Hum Hypertens. 2017;31(10):616-9. Epub 20170629. doi: 10.1038/jhh.2017.43. PubMed PMID: 28660886.	Excluded from the final analysis	The article was excluded from the analysis due to the lack of relation between the title and the abstract of the article with the subject under study

407	Wang JY, Lu FH, Sun ZJ, Wu JS, Yang YC, Lee CT, Chang CJ. Gallstone disease associated with increased risk of arterial stiffness in a Taiwanese population. Journal of Human Hypertension. 2017;31(10):616-9. doi: 10.1038/jhh.2017.43. PubMed PMID: WOS:000412219400003.	Excluded from the final analysis	The article was excluded from the analysis due to the lack of relation between the title and the abstract of the article with the subject under study
408	Froutan Y, Alizadeh A, Mansour-Ghanaei F, Joukar F, Froutan H, Bagheri FB, et al. Gallstone disease founded by ultrasonography in functional dyspepsia: prevalence and associated factors. Int J Clin Exp Med. 2015;8(7):11283-8. Epub 20150715. PubMed PMID: 26379936; PubMed Central PMCID: PMCPMC4565319.	Excluded from the final analysis	The article was excluded from the analysis due to the lack of relation between the title and the abstract of the article with the subject under study
409	Mallick B, Anand AC. Gallstone Disease in Cirrhosis-Pathogenesis and Management. J Clin Exp Hepatol. 2022;12(2):551-9. Epub 20210916. doi: 10.1016/j.jceh.2021.09.011. PubMed PMID: 35535063; PubMed Central PMCID: PMCPMC9077239.	Excluded from the final analysis	The article was excluded from the analysis due to the lack of relation between the title and the abstract of the article with the subject under study
410	Weng XP, Fu FW, Ling Y, Sun J, Chen WL. Gallstone Disease is an Independent Predictor for Poststroke Cognitive Impairment in Young Patients with Acute Ischemic Stroke. European Neurology. 2020;82(1-3):15-22. doi: 10.1159/000504343. PubMed PMID: WOS:000526850100003.	Excluded from the final analysis	The article was excluded from the analysis due to the lack of relation between the title and the abstract of the article with the subject under study
411	Wildi S, Reber PU, Baer HU. Gallstone disease masking malignant bile duct tumors: A rare but important coincidence. Digestive Surgery. 2000;17(2):174-8. doi: 10.1159/000018824. PubMed PMID: WOS:000086783300017.	Excluded from the final analysis	The article was excluded from the analysis due to the lack of relation between the title and the abstract of the article with the subject under study

412	Eto T. GALLSTONE FORMATION IN DOGS AFTER SELECTIVE OCCLUSION OF THE PORTAL-VEIN BRANCHES. Japanese Journal of Surgery. 1988;18(3):268-75. doi: 10.1007/bf02471443. PubMed PMID: WOS:A1988P109900005.	Excluded from the final analysis	The article was excluded from the analysis due to the lack of relation between the title and the abstract of the article with the subject under study
413	Eto T, Tsuchiya R, Harada N, Tsunoda T, Yamamoto K. GALLSTONE FORMATION IN DOGS BY SELECTIVE OCCLUSION OF PORTAL-VEIN BRANCHES. Digestive Diseases and Sciences. 1986;31(10):S373-S. PubMed PMID: WOS:A1986E217001481.	Excluded from the final analysis	The article was excluded from the analysis due to the lack of relation between the title and the abstract of the article with the subject under study
414	Jarbou SM, al-Hammouri FA. Gallstone ileus in the Jordanian Royal Medical Services in a 10-year period. East Mediterr Health J. 2000;6(5-6):1117-21. PubMed PMID: 12197337.	Excluded from the final analysis	The article was excluded from the analysis due to the lack of relation between the title and the abstract of the article with the subject under study
415	González-Robles ME, Menéndez-Goti LL, de Jesús González-Luna A, Cuevas-Calla CV, Torres-Salazar QL. Gallstone ileus presenting in an elderly patient: A case report. Int J Surg Case Rep. 2024;124:110440. Epub 20241012. doi: 10.1016/j.ijscr.2024.110440. PubMed PMID: 39405751.	Excluded from the final analysis	The article was excluded from the analysis due to the lack of relation between the title and the abstract of the article with the subject under study
416	Gasparrini M, Liverani A, Catracchia V, Conte S, Leonardo G, Marino G, et al. Gallstone ileus: a case report and review of the literature. Chir Ital. 2008;60(5):755-9. PubMed PMID: 19062503.	Excluded from the final analysis	The article was excluded from the analysis due to the lack of relation between the title and the abstract of the article with the subject under study

417	Zappia F, Petracca G, Talarico CA. Gallstone ileus. A case treated with minilaparotomy and a review of the literature. Ann Ital Chir. 2017;6. Epub 20170329. PubMed PMID: 28401880.	Excluded from the final analysis	The article was excluded from the analysis due to the lack of relation between the title and the abstract of the article with the subject under study
418	Wang H, So H, Ko SW, Jung SW, Bang SJ, Park EJ. Gallstone Is Associated with Metabolic Factors and Exercise in Korea. Healthcare (Basel). 2022;10(8). Epub 20220724. doi: 10.3390/healthcare10081372. PubMed PMID: 35893194; PubMed Central PMCID: PMCPMC9329956.	Excluded from the final analysis	The article was excluded from the analysis due to the lack of relation between the title and the abstract of the article with the subject under study
419	Wang H, So H, Ko SW, Jung SW, Bang SJ, Park EJ. Gallstone Is Associated with Metabolic Factors and Exercise in Korea. Healthcare. 2022;10(8). doi: 10.3390/healthcare10081372. PubMed PMID: WOS:000846011200001.	Excluded from the final analysis	The article was excluded from the analysis due to the lack of relation between the title and the abstract of the article with the subject under study
420	Chiu CC, Lee KJ, Weng SF, Yang YM, Lin YS. Gallstone is correlated with an increased risk of idiopathic sudden sensorineural hearing loss: a retrospective cohort study. Bmj Open. 2015;5(9). doi: 10.1136/bmjopen-2015-009018. PubMed PMID: WOS:000363484000084.	Excluded from the final analysis	The article was excluded from the analysis due to the lack of relation between the title and the abstract of the article with the subject under study
421	Jeong YH, Kim KO, Lee HC, Sohn SH, Lee JW, Lee SH, et al. Gallstone prevalence and risk factors in patients with ulcerative colitis in Korean population. Medicine (Baltimore). 2017;96(31):e7653. doi: 10.1097/md.0000000000007653. PubMed PMID: 28767582; PubMed Central PMCID: PMCPMC5626136.	Excluded from the final analysis	The article was excluded from the analysis due to the lack of relation between the title and the abstract of the article with the subject under study

422	Zheng Y, Xu M, Li YP, Hruby A, Rimm EB, Hu FB, et al. Gallstones and Risk of Coronary Heart Disease: Prospective Analysis of 270000 Men and Women From 3 US Cohorts and Meta-Analysis. Arteriosclerosis Thrombosis and Vascular Biology. 2016;36(9):1997-2003. doi: 10.1161/atvbaha.116.307507. PubMed PMID: WOS:000383582900036.	Excluded from the final analysis	The article was excluded from the analysis due to the lack of relation between the title and the abstract of the article with the subject under study
423	Méndez-Sánchez N, Zamora-Valdés D, Flores-Rangel JA, Pérez-Sosa JA, Vásquez-Fernández F, Lezama-Mora JI, et al. Gallstones are associated with carotid atherosclerosis. Liver International. 2008;28(3):402-6. doi: 10.1111/j.1478-3231.2007.01632.x. PubMed PMID: WOS:000253258100015.	Excluded from the final analysis	The article was excluded from the analysis due to the lack of relation between the title and the abstract of the article with the subject under study
424	Abraham P, Desai DC, Joshi AG. Gallstones in portal hypertension: is the liver or the portal hypertension responsible? J Assoc Physicians India. 2002;50:515-7. PubMed PMID: 12164400.	Excluded from the final analysis	The article was excluded from the analysis due to the lack of relation between the title and the abstract of the article with the subject under study
425	Hunt DRH, Chu FCK. Gangrenous cholecystitis in the laparoscopic era. Australian and New Zealand Journal of Surgery. 2000;70(6):428-30. doi: 10.1046/j.1440-1622.2000.01851.x. PubMed PMID: WOS:000088312800008.	Excluded from the final analysis	The article was excluded from the analysis due to the lack of relation between the title and the abstract of the article with the subject under study
426	Mason EE, Printen KJ, Blommers TJ, Scott DH. Gastric bypass for obesity after ten years experience. Int J Obes. 1978;2(2):197-206. PubMed PMID: 711364.	Excluded from the final analysis	The article was excluded from the analysis due to the lack of relation between the title and the abstract of the article with the subject under study

427	Wight CO, Seed M, Yeo WW, McCulloch TA. Gastric outflow obstruction caused by gall stones and leading to death by complex metabolic derangement. Journal of Clinical Pathology. 1997;50(11):963-5. doi: 10.1136/jcp.50.11.963. PubMed PMID: WOS:A1997YK17300019.	Excluded from the final analysis	The article was excluded from the analysis due to the lack of relation between the title and the abstract of the article with the subject under study
428	Sequeira C, Santos I, Lopes S, Carvalheiro V, Mangualde J, Oliveira AP. Gastroduodenal artery pseudoaneurysm presenting with hyperamylasemia. Rev Esp Enferm Dig. 2023;115(9):535-6. doi: 10.17235/reed.2022.9407/2022. PubMed PMID: 36537340.	Excluded from the final analysis	The article was excluded from the analysis due to the lack of relation between the title and the abstract of the article with the subject under study
429	Ebert EC, Nagar M, Hagspiel KD. Gastrointestinal and Hepatic Complications of Sickle Cell Disease. Clinical Gastroenterology and Hepatology. 2010;8(6):483-9. doi: 10.1016/j.cgh.2010.02.016. PubMed PMID: WOS:000278747200007.	Excluded from the final analysis	The article was excluded from the analysis due to the lack of relation between the title and the abstract of the article with the subject under study
430	Ebert E. Gastrointestinal involvement in spinal cord injury: a clinical perspective. J Gastrointestin Liver Dis. 2012;21(1):75-82. PubMed PMID: 22457863.	Excluded from the final analysis	The article was excluded from the analysis due to the lack of relation between the title and the abstract of the article with the subject under study
431	Atkinson DS, Fenlon HM, Kuligowska E. Gastrointestinal/genitourinary case of the day - Case 3: Liver infarction in a patient with sickle cell anemia, splenic atrophy, and gallstones. American Journal of Roentgenology. 1999;173(3):792-3. PubMed PMID: WOS:000082125100058.	Excluded from the final analysis	The article was excluded from the analysis due to the lack of relation between the title and the abstract of the article with the subject under study

432	Hughes DA, Pastores GM. Gaucher Disease. In: Adam MP, Feldman J, Mirzaa GM, Pagon RA, Wallace SE, Amemiya A, editors. GeneReviews(®). Seattle (WA): University of Washington, Seattle	Excluded from the final analysis	The article was excluded from the analysis due to the lack of relation between the title and the abstract of the article with the subject under study
433		Excluded from the final analysis	The article was excluded from the analysis due to the lack of relation between the title and the abstract of the article with the subject under study
434	Lauridsen BK, Stender S, Frikke-Schmidt R, Nordestgaard BG, Tybjærg-Hansen A. Genetic variation in the cholesterol transporter NPC1L1, ischaemic vascular disease, and gallstone disease. European Heart Journal. 2015;36(25):1601-8. doi: 10.1093/eurheartj/ehv108. PubMed PMID: WOS:000358177800013.	Excluded from the final analysis	The article was excluded from the analysis due to the lack of relation between the title and the abstract of the article with the subject under study
435	Puppala S, Dodd GD, Fowler S, Arya R, Schneider J, Farook VS, et al. A genomewide search finds major susceptibility loci for gallbladder disease on chromosome 1 in Mexican Americans. Am J Hum Genet. 2006;78(3):377-92. Epub 20060106. doi: 10.1086/500274. PubMed PMID: 16400619; PubMed Central PMCID: PMCPMC1380282.	Excluded from the final analysis	The article was excluded from the analysis due to the lack of relation between the title and the abstract of the article with the subject under study
436	Lello L, Raben TG, Yong SY, Tellier L, Hsu SDH. Genomic Prediction of 16 Complex Disease Risks Including Heart Attack, Diabetes, Breast and Prostate Cancer. Sci Rep. 2019;9(1):15286. Epub 20191025. doi: 10.1038/s41598-019-51258-x. PubMed PMID: 31653892; PubMed Central PMCID: PMCPMC6814833.	Excluded from the final analysis	The article was excluded from the analysis due to the lack of relation between the title and the abstract of the article with the subject under study

437	Zhang ZJ, Du Z, Liu QH, Wu T, Tang Q, Zhang JH, et al. Glucagon-like peptide 1 analogue prevents cholesterol gallstone formation by modulating intestinal farnesoid X receptor activity. Metabolism-Clinical and Experimental. 2021;118. doi: 10.1016/j.metabol.2021.154728. PubMed PMID: WOS:000649271300009.	Excluded from the final analysis	The article was excluded from the analysis due to the lack of relation between the title and the abstract of the article with the subject under study
438	Antunes ML, Cabral G, Tavares R, Noronha C, Araújo J. Going Round in Circles with a Multisystemic Disease: A Unique Case of Parasitic Aortitis. Eur J Case Rep Intern Med. 2017;4(6):000601. Epub 20170517. doi: 10.12890/2017_000601. PubMed PMID: 30755949; PubMed Central PMCID: PMCPMC6346790.	Excluded from the final analysis	The article was excluded from the analysis due to the lack of relation between the title and the abstract of the article with the subject under study
439	Stewart L, Oesterle AL, Griffiss JM, Jarvis GA, Aagaard B, Way LW. Gram-negative bacteria killed by complement are associated with more severe biliary infections and produce more tumor necrosis factor-alpha in sera. Surgery. 2002;132(2):408-14. doi: 10.1067/msy.2002.127423. PubMed PMID: 12219042.	Excluded from the final analysis	The article was excluded from the analysis due to the lack of relation between the title and the abstract of the article with the subject under study
440	Song ST, Cai LY, Zeng X, Xie WF. Gut Microbial Profile in Asymptomatic Gallstones. Front Microbiol. 2022;13:882265. Epub 20220613. doi: 10.3389/fmicb.2022.882265. PubMed PMID: 35770155; PubMed Central PMCID: PMCPMC9234526.	Excluded from the final analysis	The article was excluded from the analysis due to the lack of relation between the title and the abstract of the article with the subject under study
441	Mikou MM, Mouaffak Y, Benyacob A, Mosaddek A, Faroudy M, Ababou A, et al. [Haemocholecyst: a rare complication of anticoagulant treatment]. Ann Fr Anesth Reanim. 2004;23(7):733-6. doi: 10.1016/j.annfar.2004.02.055. PubMed PMID: 15324963.	Excluded from the final analysis	The article was excluded from the analysis due to the lack of relation between the title and the abstract of the article with the subject under study

442	Van De Walle P, Van Outryve L. Hand-assisted laparoscopic vertical banded gastroplasty: technique and analysis of the first 140 cases. Obes Surg. 2002;12(5):628-33. doi: 10.1381/096089202321019585. PubMed PMID: 12448382.	Excluded from the final analysis	The article was excluded from the analysis due to the lack of relation between the title and the abstract of the article with the subject under study
443	Bonku R, Yu JM. Health aspects of peanuts as an outcome of its chemical composition. Food Science and Human Wellness. 2020;9(1):21-30. doi: 10.1016/j.fshw.2019.12.005. PubMed PMID: WOS:000520851800003.	Excluded from the final analysis	The article was excluded from the analysis due to the lack of relation between the title and the abstract of the article with the subject under study
444	Dwyer JT. Health aspects of vegetarian diets. Am J Clin Nutr. 1988;48(3 Suppl):712-38. doi: 10.1093/ajcn/48.3.712. PubMed PMID: 3046302.	Excluded from the final analysis	The article was excluded from the analysis due to the lack of relation between the title and the abstract of the article with the subject under study
445	Chahal-Kummen M, Salte OBK, Hewitt S, Blom-Høgestøl IK, Risstad H, Kristinsson J, Mala T. Health benefits and risks during 10 years after Roux-en-Y gastric bypass. Surg Endosc. 2020;34(12):5368-76. Epub 20200128. doi: 10.1007/s00464-019-07328-2. PubMed PMID: 31993812; PubMed Central PMCID: PMCPMC7644522.	Excluded from the final analysis	The article was excluded from the analysis due to the lack of relation between the title and the abstract of the article with the subject under study
446	Key TJ, Davey GK, Appleby PN. Health benefits of a vegetarian diet. Proceedings of the Nutrition Society. 1999;58(2):271-5. doi: 10.1017/s0029665199000373. PubMed PMID: WOS:000081952800009.	Excluded from the final analysis	The article was excluded from the analysis due to the lack of relation between the title and the abstract of the article with the subject under study

447	Ros E. Health benefits of nut consumption. Nutrients. 2010;2(7):652-82. Epub 20100624. doi: 10.3390/nu2070652. PubMed PMID: 22254047; PubMed Central PMCID: PMCPMC3257681.	Excluded from the final analysis	The article was excluded from the analysis due to the lack of relation between the title and the abstract of the article with the subject under study
448	Swinburn B, Ashton T, Gillespie J, Cox B, Menon A, Simmons D, Birkbeck J. Health care costs of obesity in New Zealand. Int J Obes Relat Metab Disord. 1997;21(10):891-6. doi: 10.1038/sj.ijo.0800486. PubMed PMID: 9347407.	Excluded from the final analysis	The article was excluded from the analysis due to the lack of relation between the title and the abstract of the article with the subject under study
449	Bray GA. Health hazards of obesity. Endocrinol Metab Clin North Am. 1996;25(4):907-19. doi: 10.1016/s0889-8529(05)70361-3. PubMed PMID: 8977052.	Excluded from the final analysis	The article was excluded from the analysis due to the lack of relation between the title and the abstract of the article with the subject under study
450	Bray GA. Health hazards of obesity. Endocrinology and Metabolism Clinics of North America. 1996;25(4):907-+. doi: 10.1016/s0889-8529(05)70361-3. PubMed PMID: WOS:A1996VZ40200009.	Excluded from the final analysis	The article was excluded from the analysis due to the lack of relation between the title and the abstract of the article with the subject under study
451	Welty TK. Health implications of obesity in American Indians and Alaska Natives. Am J Clin Nutr. 1991;53(6 Suppl):1616s-20s. doi: 10.1093/ajcn/53.6.1616S. PubMed PMID: 2031495.	Excluded from the final analysis	The article was excluded from the analysis due to the lack of relation between the title and the abstract of the article with the subject under study

452	Welty TK. HEALTH IMPLICATIONS OF OBESITY IN AMERICAN-INDIANS AND ALASKA NATIVES. American Journal of Clinical Nutrition. 1991;53(6):S1616-S20. doi: 10.1093/ajcn/53.6.1616S. PubMed PMID: WOS:A1991FP05900019.	Excluded from the final analysis	The article was excluded from the analysis due to the lack of relation between the title and the abstract of the article with the subject under study
453	Vasiljević N, Pecelj-Gec M, Marinković J. [Health implications of obesity]. Srp Arh Celok Lek. 1997;125(9-10):299-302. PubMed PMID: 9340803.	Excluded from the final analysis	The article was excluded from the analysis due to the lack of relation between the title and the abstract of the article with the subject under study
454	Hainer V, Kunesová M, Parízková J, Stunkard A. [Health risks and economic costs associated with obesity requiring a comprehensive weight reduction program]. Cas Lek Cesk. 1997;136(12):367-72. PubMed PMID: 9333508.	Excluded from the final analysis	The article was excluded from the analysis due to the lack of relation between the title and the abstract of the article with the subject under study
455	Mikami T, Hirata K, Oikawa I, Kimura M, Kimura H. Hemobilia caused by a giant benign hemangioma of the liver: Report of a case. Surgery Today-the Japanese Journal of Surgery. 1998;28(9):948-52. doi: 10.1007/s005950050259. PubMed PMID: WOS:000075757200015.	Excluded from the final analysis	The article was excluded from the analysis due to the lack of relation between the title and the abstract of the article with the subject under study
456	Joo YE, Kim HS, Choi SK, Rew JS, Kim HJ, Kim SJ. Hemobilia caused by liver abscess due to intrahepatic duct stones. Journal of Gastroenterology. 2003;38(5):507-11. doi: 10.1007/s00535-002-1087-2. PubMed PMID: WOS:000183185500014.	Excluded from the final analysis	The article was excluded from the analysis due to the lack of relation between the title and the abstract of the article with the subject under study

457	Tanaka T, Takakura K, Maruyama Y, Hidaka A, Nakano M, Torisu Y, Saruta M. Hemobilia Derived from Cystic Artery Pseudoaneurysm. Case Reports in Gastroenterology. 2019;13(1):89-94. doi: 10.1159/000497097. PubMed PMID: WOS:000746569200014.	Excluded from the final analysis	The article was excluded from the analysis due to the lack of relation between the title and the abstract of the article with the subject under study
458	Czerniak A, Thompson JN, Hemingway AP, Soreide O, Benjamin IS, Allison DJ, Blumgart LH. Hemobilia. A disease in evolution. Arch Surg. 1988;123(6):718-21. doi: 10.1001/archsurg.1988.01400300064010. PubMed PMID: 3369935.	Excluded from the final analysis	The article was excluded from the analysis due to the lack of relation between the title and the abstract of the article with the subject under study
459	Wang DD, Wu S, Kong BB, Song LL. Hemophagocytic lymphohistiocytosis with jaundice as first manifestation: A case report. World Journal of Clinical Cases. 2023;11(34):8212-8. doi: 10.12998/wjcc.v11.i34.8212. PubMed PMID: WOS:001129520400016.	Excluded from the final analysis	The article was excluded from the analysis due to the lack of relation between the title and the abstract of the article with the subject under study
460	Walton JM, Abraham RJ, Perey BJ, MacGregor JH, Campbell DR. Hepatic artery pseudoaneurysms in acute pancreatitis. Can J Surg. 1991;34(4):377-80. PubMed PMID: 1868396.	Excluded from the final analysis	The article was excluded from the analysis due to the lack of relation between the title and the abstract of the article with the subject under study
461	Cunningham D, Mills PR, Quigley EMM, Patrick RS, Watkinson G, Mackenzie JF, Russell RI. HEPATIC GRANULOMAS - EXPERIENCE OVER A 10-YEAR PERIOD IN THE WEST OF SCOTLAND. Quarterly Journal of Medicine. 1982;51(202):162-70. PubMed PMID: WOS:A1982NR41000005.	Excluded from the final analysis	The article was excluded from the analysis due to the lack of relation between the title and the abstract of the article with the subject under study

462	Cunnigham D, Mills PR, Quigley EM, Patrick RS, Watkinson G, MacKenzie JF, Russell RI. Hepatic granulomas: experience over a 10-year period in the West of Scotland. Q J Med. 1982;51(202):162-70. PubMed PMID: 7111678.	Excluded from the final analysis	The article was excluded from the analysis due to the lack of relation between the title and the abstract of the article with the subject under study
463	Mitchell E, Ranganathan S, McKiernan P, Squires RH, Strauss K, Soltys K, et al. Hepatic Parenchymal Injury in Crigler-Najjar Type I. J Pediatr Gastroenterol Nutr. 2018;66(4):588-94. doi: 10.1097/mpg.0000000000001843. PubMed PMID: 29176474.	Excluded from the final analysis	The article was excluded from the analysis due to the lack of relation between the title and the abstract of the article with the subject under study
464	Lin JN, Yen CM, Liu CS, Tsai MS, Kuo KK. Hepatic Schistosomiasis japonica in a patient with gallstones and bile duct stones--a case report. Kaohsiung J Med Sci. 2001;17(8):437-40. PubMed PMID: 11715844.	Excluded from the final analysis	The article was excluded from the analysis due to the lack of relation between the title and the abstract of the article with the subject under study
465	Xiao JL, Ng CH, Chan KE, Fu C, Tay P, Yong JN, et al. Hepatic, Extra-hepatic Outcomes and Causes of Mortality in NAFLD-An Umbrella Overview of Systematic Review of Meta-Analysis. Journal of Clinical and Experimental Hepatology. 2023;13(4):656-65. doi: 10.1016/j.jceh.2022.11.006. PubMed PMID: WOS:001035247400001.	Excluded from the final analysis	The article was excluded from the analysis due to the lack of relation between the title and the abstract of the article with the subject under study
466	Chang CH, Wang YY, Jiao Y. Hepatitis A virus-associated acute acalculous cholecystitis in an adult-onset Still's disease patient: A case report and review of the literature. World J Clin Cases. 2023;11(6):1410-8. doi: 10.12998/wjcc.v11.i6.1410. PubMed PMID: 36926135; PubMed Central PMCID: PMCPMC10013114.	Excluded from the final analysis	The article was excluded from the analysis due to the lack of relation between the title and the abstract of the article with the subject under study

467	Pitt HA. Hepatobiliary Hands of Hopkins. Ann Surg. 2018;267(2S Suppl 2):S34-s9. doi: 10.1097/sla.0000000000002606. PubMed PMID: 29206676.	Excluded from the final analysis	The article was excluded from the analysis due to the lack of relation between the title and the abstract of the article with the subject under study
468	Grossman SJ, Joyce JM. Hepatobiliary imaging. Emerg Med Clin North Am. 1991;9(4):853-74. PubMed PMID: 1915052.	Excluded from the final analysis	The article was excluded from the analysis due to the lack of relation between the title and the abstract of the article with the subject under study
469	Colletti PM, Barakos JA, Ralls PW, Siegel ME, Halls JM. HEPATOBILIARY SCINTIGRAPHY AND SCINTIANGIOGRAPHY IN ABDOMINAL-TRAUMA. Clinical Nuclear Medicine. 1987;12(11):901-9. doi: 10.1097/00003072-198711000-00024. PubMed PMID: WOS:A1987L064600024.	Excluded from the final analysis	The article was excluded from the analysis due to the lack of relation between the title and the abstract of the article with the subject under study
470	Bismuth H, Majno PE. Hepatobiliary surgery. Journal of Hepatology. 2000;32:208-24. doi: 10.1016/s0168-8278(00)80427-4. PubMed PMID: WOS:000085968400018.	Excluded from the final analysis	The article was excluded from the analysis due to the lack of relation between the title and the abstract of the article with the subject under study
471	Ball NJ, Duggan MA. HEPATOLITHIASIS IN HEREDITARY HEMORRHAGIC TELANGIECTASIA. Archives of Pathology & Laboratory Medicine. 1990;114(4):423-5. PubMed PMID: WOS:A1990CX41300021.	Excluded from the final analysis	The article was excluded from the analysis due to the lack of relation between the title and the abstract of the article with the subject under study

472	Mendoza A, Oliff S, Elias E. Hereditary haemorrhagic telangiectasia and secondary biliary cirrhosis. Eur J Gastroenterol Hepatol. 1995;7(10):999-1002. doi: 10.1097/00042737-199510000-00017. PubMed PMID: 8590149.	Excluded from the final analysis	The article was excluded from the analysis due to the lack of relation between the title and the abstract of the article with the subject under study
473	Mendoza A, Oliff S, Elias E. HEREDITARY HEMORRHAGIC TELANGIECTASIA AND SECONDARY BILIARY-CIRRHOSIS. European Journal of Gastroenterology & Hepatology. 1995;7(10):999-1002. doi: 10.1097/00042737-199510000-00017. PubMed PMID: WOS:A1995TG40500017.	Excluded from the final analysis	The article was excluded from the analysis due to the lack of relation between the title and the abstract of the article with the subject under study
474	Young SB, Arregui M, Singh K. HIDA scan ejection fraction does not predict sphincter of Oddi hypertension or clinical outcome in patients with suspected chronic acalculous cholecystitis. Surg Endosc. 2006;20(12):1872-8. doi: 10.1007/s00464-005-0245-z. PubMed PMID: 17031746.	Excluded from the final analysis	The article was excluded from the analysis due to the lack of relation between the title and the abstract of the article with the subject under study
475	Rajeeth G, Tilakaratne S, Siriwardana RC. The hidden threat of uncontrollable bleeding from the gallbladder bed during laparoscopic cholecystectomy. International Journal of Surgery Case Reports. 2023;112. doi: 10.1016/j.ijscr.2023.108957. PubMed PMID: WOS:001101469200001.	Excluded from the final analysis	The article was excluded from the analysis due to the lack of relation between the title and the abstract of the article with the subject under study
476	Zeng D, Wu H, Huang Q, Zeng A, Yu Z, Zhong Z. High Levels of Serum Triglyceride, Low-density Lipoprotein Cholesterol, Total Bile Acid, and Total Bilirubin are Risk Factors for Gallstones. Clin Lab. 2021;67(8). doi: 10.7754/Clin.Lab.2021.201228. PubMed PMID: 34383399.	Excluded from the final analysis	The article was excluded from the analysis due to the lack of relation between the title and the abstract of the article with the subject under study

477	Zeng DH, Wu HM, Huang QY, Zeng AX, Yu ZK, Zhong ZX. High Levels of Serum Triglyceride, Low-density Lipoprotein Cholesterol, Total Bile Acid, and Total Bilirubin are Risk Factors for Gallstones. Clinical Laboratory. 2021;67(8):1905-13. doi: 10.7754/Clin.Lab.2021.201228. PubMed PMID: WOS:000684711600020.	Excluded from the final analysis	The article was excluded from the analysis due to the lack of relation between the title and the abstract of the article with the subject under study
478	Shaka H, Asotibe JC, Achebe I, Pudasaini G. Higher Inpatient Morbidity and Mortality in Biliary Pancreatitis Compared to Hypertriglyceridemia-Induced Pancreatitis: A Nationwide Retrospective Study. Cureus Journal of Medical Science. 2020;12(9). doi: 10.7759/cureus.10351. PubMed PMID: WOS:000567771300005.	Excluded from the final analysis	The article was excluded from the analysis due to the lack of relation between the title and the abstract of the article with the subject under study
479	Pullingr CR, Eng C, Salen G, Shefer S, Batta AK, Erickson SK, et al. Human cholesterol 7α-hydroxylase (CYP7A1) deficiency has a hypercholesterolemic phenotype. Journal of Clinical Investigation. 2002;110(1):109-17. doi: 10.1172/jci200215387. PubMed PMID: WOS:000176665100015.	Excluded from the final analysis	The article was excluded from the analysis due to the lack of relation between the title and the abstract of the article with the subject under study
480	Manz F. Hydration and disease. J Am Coll Nutr. 2007;26(5 Suppl):535s-41s. doi: 10.1080/07315724.2007.10719655. PubMed PMID: 17921462.	Excluded from the final analysis	The article was excluded from the analysis due to the lack of relation between the title and the abstract of the article with the subject under study
481	Manz F. Hydration and disease. Journal of the American College of Nutrition. 2007;26(5):535S-41S. doi: 10.1080/07315724.2007.10719655. PubMed PMID: WOS:000250506300005.	Excluded from the final analysis	The article was excluded from the analysis due to the lack of relation between the title and the abstract of the article with the subject under study

482	Zheng C, Zhong X, Ma M, Zheng X, Jiang B, Zheng YP. Hyperlipidaemic acute pancreatitis complicated with multiple deep vein thromboses and pulmonary embolism: a case successfully salvaged by radiologic intervention. Curr Med Res Opin. 2021;37(1):53-7. Epub 20201203. doi: 10.1080/03007995.2020.1854702. PubMed PMID: 33222536.	Excluded from the final analysis	The article was excluded from the analysis due to the lack of relation between the title and the abstract of the article with the subject under study
483	Liesemer K, Mullen N. Hypertensive emergency successfully treated with metoprolol: a case report. Pediatr Emerg Care. 2009;25(5):333-5. doi: 10.1097/PEC.0b013e3181a34816. PubMed PMID: 19444030.	Excluded from the final analysis	The article was excluded from the analysis due to the lack of relation between the title and the abstract of the article with the subject under study
484	Soliman S. Hypertriglyceridemia-Induced Pancreatitis With Rapid Response to Insulin Therapy. J Med Cases. 2021;12(1):23-6. Epub 20201118. doi: 10.14740/jmc3595. PubMed PMID: 34434423; PubMed Central PMCID: PMCPMC8383640.	Excluded from the final analysis	The article was excluded from the analysis due to the lack of relation between the title and the abstract of the article with the subject under study
485	Zobel MJ, Stewart L. Hyponatremia is associated with more severe biliary disease. World J Gastrointest Surg. 2020;12(2):45-54. doi: 10.4240/wjgs.v12.i2.45. PubMed PMID: 32128028; PubMed Central PMCID: PMCPMC7044107.	Excluded from the final analysis	The article was excluded from the analysis due to the lack of relation between the title and the abstract of the article with the subject under study
486	Lin K, Ofori E, Lin AN, Lin S, Lin T, Rasheed A, et al. Hypothermia-Related Acute Pancreatitis. Case Rep Gastroenterol. 2018;12(2):217-23. Epub 20180531. doi: 10.1159/000489296. PubMed PMID: 29928186; PubMed Central PMCID: PMCPMC6006605.	Excluded from the final analysis	The article was excluded from the analysis due to the lack of relation between the title and the abstract of the article with the subject under study

487	Krupa L, Kalinowski P, Ligocka J, Dauer M, Jankowski K, Gozdowska J, et al. The <i>ABCG8</i> polymorphism increases the risk of gallbladder cancer in the general population and gallstones in obese patients from Poland. European Journal of Clinical Investigation. 2024;54(8). doi: 10.1111/eci.14213. PubMed PMID: WOS:001202175800001.	Excluded from the final analysis	The article was excluded from the analysis due to the lack of relation between the title and the abstract of the article with the subject under study
488	Verit A, Güner ND. <i>Helicobacter pylori</i> and urinary system stones: Endoluminal damage as sub-hypothesis to support the current stone theory. Medical Hypotheses. 2014;83(6):677-80. doi: 10.1016/j.mehy.2014.09.016. PubMed PMID: WOS:000347595600010.	Excluded from the final analysis	The article was excluded from the analysis due to the lack of relation between the title and the abstract of the article with the subject under study
489	Panwar U, Singh SK. Identification of Novel Pancreatic Lipase Inhibitors Using In Silico Studies. Endocr Metab Immune Disord Drug Targets. 2019;19(4):449-57. doi: 10.2174/1871530319666181128100903. PubMed PMID: 30484411.	Excluded from the final analysis	The article was excluded from the analysis due to the lack of relation between the title and the abstract of the article with the subject under study
490	Akay T, Sari E. Identification of risk factors involved in recurrence after common bile duct stone removal with ERCP A retrospective observational study. Medicine. 2022;101(9). doi: 10.1097/md.0000000000029037. PubMed PMID: WOS:000764242200014.	Excluded from the final analysis	The article was excluded from the analysis due to the lack of relation between the title and the abstract of the article with the subject under study
491	Hanbidge AE, Buckler PM, O'Malley ME, Wilson SR. Imaging evaluation for acute pain in the right upper quadrant. Radiographics. 2004;24(4):1117-35. doi: 10.1148/rg.244035149. PubMed PMID: WOS:000222436100016.	Excluded from the final analysis	The article was excluded from the analysis due to the lack of relation between the title and the abstract of the article with the subject under study

492	Elmunzer BJ, Noureldin M, Morgan KA, Adams DB, Coté GA, Waljee AK. The Impact of Cholecystectomy After Endoscopic Sphincterotomy for Complicated Gallstone Disease. American Journal of Gastroenterology. 2017;112(10):1596-602. doi: 10.1038/ajg.2017.247. PubMed PMID: WOS:000412299200019.	Excluded from the final analysis	The article was excluded from the analysis due to the lack of relation between the title and the abstract of the article with the subject under study
493	Chae W, Lee HS, Jo JH, Chung MJ, Bang S, Park SW, et al. Impact of cholecystectomy on acute coronary syndrome according to metabolic condition: a nationwide population-based cohort study. Sci Rep. 2023;13(1):7300. Epub 20230505. doi: 10.1038/s41598-023-33440-4. PubMed PMID: 37147417; PubMed Central PMCID: PMCPMC10163235.	Excluded from the final analysis	The article was excluded from the analysis due to the lack of relation between the title and the abstract of the article with the subject under study
494	Strömberg J, Sandblom G. Impact of Comorbidity and Prescription Drugs on Haemorrhage in Cholecystectomy. World Journal of Surgery. 2017;41(8):1985-92. doi: 10.1007/s00268-017-3961-3. PubMed PMID: WOS:000405315900008.	Excluded from the final analysis	The article was excluded from the analysis due to the lack of relation between the title and the abstract of the article with the subject under study
495	Balakrishnan G, Iqbal T, Uppinakudru G, Fernandes R, Bangera S, Dutt RA. The impact of lifestyle stressors, menstrual pattern, and cardiometabolic risk factors on young females with cholelithiasis. Journal of Education and Health Promotion. 2022;11(1). doi: 10.4103/jehp.jehp_1767_21. PubMed PMID: WOS:000877898400008.	Excluded from the final analysis	The article was excluded from the analysis due to the lack of relation between the title and the abstract of the article with the subject under study
496	Chen J, Liu ZT, Lyu JT, Jiang GP. Impact of metabolic disorders on gallstone disease and perioperative recovery after laparoscopic cholecystectomy. Hepatobiliary Pancreat Dis Int. 2024. Epub 20240805. doi: 10.1016/j.hbpd.2024.08.001. PubMed PMID: 39129076.	Excluded from the final analysis	The article was excluded from the analysis due to the lack of relation between the title and the abstract of the article with the subject under study

497	Paajanen H, Käkelä P, Suuronen S, Paajanen J, Juvonen P, Pihlajamäki J. Impact of obesity and associated diseases on outcome after laparoscopic cholecystectomy. Surg Laparosc Endosc Percutan Tech. 2012;22(6):509-13. doi: 10.1097/SLE.0b013e318270473b. PubMed PMID: 23238377.	Excluded from the final analysis	The article was excluded from the analysis due to the lack of relation between the title and the abstract of the article with the subject under study
498	Paajanen H, Käkelä P, Suuronen S, Paajanen J, Juvonen P, Pihlajamäki J. Impact of Obesity and Associated Diseases on Outcome After Laparoscopic Cholecystectomy. Surgical Laparoscopy Endoscopy & Percutaneous Techniques. 2012;22(6):509-13. doi: 10.1097/SLE.0b013e318270473b. PubMed PMID: WOS:000312597400005.	Excluded from the final analysis	The article was excluded from the analysis due to the lack of relation between the title and the abstract of the article with the subject under study
499	Field AE, Coakley EH, Must A, Spadano JL, Laird N, Dietz WH, et al. Impact of overweight on the risk of developing common chronic diseases during a 10-year period. Arch Intern Med. 2001;161(13):1581-6. doi: 10.1001/archinte.161.13.1581. PubMed PMID: 11434789.	Excluded from the final analysis	The article was excluded from the analysis due to the lack of relation between the title and the abstract of the article with the subject under study
500	Field AE, Coakley EH, Spadano JL, Laird N, Dietz WH, Rimm E, Colditz GA. Impact of overweight on the risk of developing common chronic diseases during a 10-year period. Archives of Internal Medicine. 2001;161(13):1581-6. doi: 10.1001/archinte.161.13.1581. PubMed PMID: WOS:000169584900001.	Excluded from the final analysis	The article was excluded from the analysis due to the lack of relation between the title and the abstract of the article with the subject under study
501	Orzeł D, Żmijewski M, Bronkowska M. Impact of products from ground buckwheat added to balanced diets on biochemical blood markers in Wistar rats. Rocz Panstw Zakl Hig. 2015;66(3):239-44. PubMed PMID: 26400120.	Excluded from the final analysis	The article was excluded from the analysis due to the lack of relation between the title and the abstract of the article with the subject under study

502	Jolly K, Chambers R. Improving outcomes for patients with obesity. Practitioner. 2014;258(1773):29-31, 3. PubMed PMID: 25211791.	Excluded from the final analysis	The article was excluded from the analysis due to the lack of relation between the title and the abstract of the article with the subject under study
503	Lu F, Wang L, Chen Y, Zhong X, Huang Z. In vitro cultured calculus bovis attenuates cerebral ischaemia-reperfusion injury by inhibiting neuronal apoptosis and protecting mitochondrial function in rats. J Ethnopharmacol. 2020;263:113168. Epub 20200727. doi: 10.1016/j.jep.2020.113168. PubMed PMID: 32730869.	Excluded from the final analysis	The article was excluded from the analysis due to the lack of relation between the title and the abstract of the article with the subject under study
504	Lu FB, Wang LF, Chen YY, Zhong XM, Huang Z. In vitro cultured calculus bovis attenuates cerebral ischaemia-reperfusion injury by inhibiting neuronal apoptosis and protecting mitochondrial function in rats. Journal of Ethnopharmacology. 2020;263. doi: 10.1016/j.jep.2020.113168. PubMed PMID: WOS:000570065000006.	Excluded from the final analysis	The article was excluded from the analysis due to the lack of relation between the title and the abstract of the article with the subject under study
505	McSherry CK, Glenn F. INCIDENCE AND CAUSES OF DEATH FOLLOWING SURGERY FOR NONMALIGNANT BILIARY-TRACT DISEASE. Annals of Surgery. 1980;191(3):271-5. doi: 10.1097/00000658-198003000-00003. PubMed PMID: WOS:A1980JJ87500003.	Excluded from the final analysis	The article was excluded from the analysis due to the lack of relation between the title and the abstract of the article with the subject under study
506	Guzmán HM, Sepúlveda M, Rosso N, San Martin A, Guzmán F, Guzmán HC. Incidence and Risk Factors for Cholelithiasis After Bariatric Surgery. Obes Surg. 2019;29(7):2110-4. doi: 10.1007/s11695-019-03760-4. PubMed PMID: 31001756.	Excluded from the final analysis	The article was excluded from the analysis due to the lack of relation between the title and the abstract of the article with the subject under study

507	Strömberg J, Sadr-Azodi O, Videhult P, Hammarqvist F, Sandblom G. Incidence and risk factors for symptomatic venous thromboembolism following cholecystectomy. Langenbecks Arch Surg. 2015;400(4):463-9. Epub 20150215. doi: 10.1007/s00423-015-1284-0. PubMed PMID: 25682056.	Excluded from the final analysis	The article was excluded from the analysis due to the lack of relation between the title and the abstract of the article with the subject under study
508	Assakran BS, Khalid R, Albadrani H, Alsuhaibani A, Almutairi A, Alhomidan R, et al. Incidence of Asymptomatic Gallstones in Obese Patients Who Underwent Bariatric Surgery in Qassim Region at King Fahad Specialist Hospital. Cureus. 2023;15(8):e44154. Epub 20230826. doi: 10.7759/cureus.44154. PubMed PMID: 37753031; PubMed Central PMCID: PMCPMC10519147.	Excluded from the final analysis	The article was excluded from the analysis due to the lack of relation between the title and the abstract of the article with the subject under study
509	Faichney A, Donaldson LA. INCIDENCE OF GALLSTONES AND ISCHEMIC-HEART-DISEASE IN THE WEST OF SCOTLAND. British Medical Journal. 1980;280(6211):402-3. doi: 10.1136/bmj.280.6211.402-b. PubMed PMID: WOS:A1980JD95000040.	Excluded from the final analysis	The article was excluded from the analysis due to the lack of relation between the title and the abstract of the article with the subject under study
510	Sarin SK, Guptan RC, Malhotra S. Increased frequency of gallstones in cirrhotic and non-cirrhotic portal hypertension. J Assoc Physicians India. 2002;50:518-22. PubMed PMID: 12164401.	Excluded from the final analysis	The article was excluded from the analysis due to the lack of relation between the title and the abstract of the article with the subject under study
511	Sneineh MA, Harel L, Elnasasra A, Razin H, Rotmensh A, Moscovici S, et al. Increased Incidence of Symptomatic Cholelithiasis After Bariatric Roux-En-Y Gastric Bypass and Previous Bariatric Surgery: a Single Center Experience. Obes Surg. 2020;30(3):846-50. doi: 10.1007/s11695-019-04366-6. PubMed PMID: 31901127.	Excluded from the final analysis	The article was excluded from the analysis due to the lack of relation between the title and the abstract of the article with the subject under study

512	Grimaldi CH, Nelson RG, Pettitt DJ, Sampliner RE, Bennett PH, Knowler WC. INCREASED MORTALITY WITH GALLSTONE DISEASE - RESULTS OF A 20-YEAR POPULATION-BASED SURVEY IN PIMA-INDIANS. Annals of Internal Medicine. 1993;118(3):185-90. doi: 10.7326/0003-4819-118-3-199302010-00005. PubMed PMID: WOS:A1993KJ43900005.	Excluded from the final analysis	The article was excluded from the analysis due to the lack of relation between the title and the abstract of the article with the subject under study
513	Mark-Christensen A, Brandsborg S, Laurberg S, Johansen N, Pachler JH, Thorlacius-Ussing O, et al. Increased Risk of Gallstone Disease Following Colectomy for Ulcerative Colitis. American Journal of Gastroenterology. 2017;112(3):473-8. doi: 10.1038/ajg.2016.564. PubMed PMID: WOS:000397313800018.	Excluded from the final analysis	The article was excluded from the analysis due to the lack of relation between the title and the abstract of the article with the subject under study
514	Kim SY, Lim H, Park B, Lim H, Kim M, Kong IG, Choi HG. Increased risk of gallstones after appendectomy: A longitudinal follow-up study using a national sample cohort. Medicine (Baltimore). 2020;99(20):e20269. doi: 10.1097/md.0000000000020269. PubMed PMID: 32443372; PubMed Central PMCID: PMCPMC7253851.	Excluded from the final analysis	The article was excluded from the analysis due to the lack of relation between the title and the abstract of the article with the subject under study
515	Kim SY, Bang WJ, Lim H, Lim MS, Kim M, Choi HG. Increased risk of gallstones after gastrectomy: A longitudinal follow-up study using a national sample cohort in korea. Medicine (Baltimore). 2019;98(22):e15932. doi: 10.1097/md.0000000000015932. PubMed PMID: 31145363; PubMed Central PMCID: PMCPMC6709130.	Excluded from the final analysis	The article was excluded from the analysis due to the lack of relation between the title and the abstract of the article with the subject under study
516	Al-Salem AH. Indications and complications of splenectomy for children with sickle cell disease. J Pediatr Surg. 2006;41(11):1909-15. doi: 10.1016/j.jpedsurg.2006.06.020. PubMed PMID: 17101369.	Excluded from the final analysis	The article was excluded from the analysis due to the lack of relation between the title and the abstract of the article with the subject under study

517	Al-Salem AH. Indications and complications of splenectomy for children with sickle cell disease. Journal of Pediatric Surgery. 2006;41(11):1909-15. doi: 10.1016/j.jpedsurg.2006.06.020. PubMed PMID: WOS:000242395200024.	Excluded from the final analysis	The article was excluded from the analysis due to the lack of relation between the title and the abstract of the article with the subject under study
518	Shea JA, Berlin JA, Bachwich DR, Staroscik RN, Malet PF, McGuckin M, et al. Indications for and outcomes of cholecystectomy - A comparison of the pre and postlaparoscopic eras. Annals of Surgery. 1998;227(3):343-50. doi: 10.1097/00000658-199803000-00005. PubMed PMID: WOS:000072608200005.	Excluded from the final analysis	The article was excluded from the analysis due to the lack of relation between the title and the abstract of the article with the subject under study
519	Tang K, Ford B, Grasso SL, Swisher J. Infectious aortitis and managing it at a community military hospital. BMJ Case Rep. 2024;17(3). Epub 20240327. doi: 10.1136/bcr-2023-257509. PubMed PMID: 38538095; PubMed Central PMCID: PMCPMC10982747.	Excluded from the final analysis	The article was excluded from the analysis due to the lack of relation between the title and the abstract of the article with the subject under study
520	Tang KLY, Ford B, Grasso SL, Swisher J. Infectious aortitis and managing it at a community military hospital. Bmj Case Reports. 2024;17(3). doi: 10.1136/bcr-2023-257509. PubMed PMID: WOS:001194571700015.	Excluded from the final analysis	The article was excluded from the analysis due to the lack of relation between the title and the abstract of the article with the subject under study
521	Song JH, Liu MY, Ma YX, Wan QQ, Li J, Diao XO, Niu LN. Inflammation-associated ectopic mineralization. Fundamental Research. 2023;3(6):1025-38. doi: 10.1016/j.fmre.2022.04.020. PubMed PMID: WOS:001124637200001.	Excluded from the final analysis	The article was excluded from the analysis due to the lack of relation between the title and the abstract of the article with the subject under study

522	Kasana V, Rajesh S, Chauhan U, Bihari C, Choudhury A, Sarin SK. Inflammatory Myofibroblastic Tumor of Liver Masquerading as Focal Nodular Hyperplasia in a Patient with Non-Cirrhotic Portal Hypertension and Biliary Pancreatitis. Indian J Surg Oncol. 2016;7(1):110-4. Epub 20150220. doi: 10.1007/s13193-015-0381-4. PubMed PMID: 27065695; PubMed Central PMCID: PMCPMC4811821.	Excluded from the final analysis	The article was excluded from the analysis due to the lack of relation between the title and the abstract of the article with the subject under study
523	Qi Q, Han Y, Xue C. [Influence of age on severe pancreatitis]. Zhonghua Wai Ke Za Zhi. 1995;33(9):542-4. PubMed PMID: 8731873.	Excluded from the final analysis	The article was excluded from the analysis due to the lack of relation between the title and the abstract of the article with the subject under study
524	Murphy MA, Joyce WP. Information for surgical patients: implications of the World Wide Web. Eur J Surg. 2001;167(10):728-33. doi: 10.1080/11024150152707699. PubMed PMID: 11775723.	Excluded from the final analysis	The article was excluded from the analysis due to the lack of relation between the title and the abstract of the article with the subject under study
525	Cappellini MD, Musallam KM, Taher AT. Insight onto the pathophysiology and clinical complications of thalassemia intermedia. Hemoglobin. 2009;33 Suppl 1:S145-59. doi: 10.3109/03630260903351528. PubMed PMID: 20001620.	Excluded from the final analysis	The article was excluded from the analysis due to the lack of relation between the title and the abstract of the article with the subject under study
526	Misciagna G, Guerra V, Di Leo A, Correale M, Trevisan M. Insulin and gall stones: a population case control study in southern Italy. Gut. 2000;47(1):144-7. doi: 10.1136/gut.47.1.144. PubMed PMID: WOS:000087935500027.	Excluded from the final analysis	The article was excluded from the analysis due to the lack of relation between the title and the abstract of the article with the subject under study

527	Kim SS, Lee JG, Kim DW, Kim BH, Jeon YK, Kim MR, et al. Insulin resistance as a risk factor for gallbladder stone formation in Korean postmenopausal women. Korean J Intern Med. 2011;26(3):285-93. Epub 20110913. doi: 10.3904/kjim.2011.26.3.285. PubMed PMID: 22016589; PubMed Central PMCID: PMCPMC3192201.	Excluded from the final analysis	The article was excluded from the analysis due to the lack of relation between the title and the abstract of the article with the subject under study
528	Mall-Haefeli M. [Internal medicine problems regarding contraception. Part I]. Schweiz Med Wochenschr. 1980;110(36):1314-9. PubMed PMID: 7444416.	Excluded from the final analysis	The article was excluded from the analysis due to the lack of relation between the title and the abstract of the article with the subject under study
529	vanSonnenberg E, Casola G, Varney RR, Zakko S, Wittich GR, Cox J, Hofmann AF. Interventional radiology in the gallbladder. Radiographics. 1989;9(1):39-49. doi: 10.1148/radiographics.9.1.2643821. PubMed PMID: 2643821.	Excluded from the final analysis	The article was excluded from the analysis due to the lack of relation between the title and the abstract of the article with the subject under study
530	Samsonova NG, Zvenigorodskaia LA, Cherkashova EA, Lazebnik LB. [Intestinal dysbiosis and atherogenic dyslipidemia]. Eksp Klin Gastroenterol. 2010;(3):88-94. PubMed PMID: 20499450.	Excluded from the final analysis	The article was excluded from the analysis due to the lack of relation between the title and the abstract of the article with the subject under study
531	Kelly DA. Intestinal failure-associated liver disease: what do we know today? Gastroenterology. 2006;130(2 Suppl 1):S70-7. doi: 10.1053/j.gastro.2005.10.066. PubMed PMID: 16473076.	Excluded from the final analysis	The article was excluded from the analysis due to the lack of relation between the title and the abstract of the article with the subject under study

532	Arian R, Farwati R, Toutounji Z, Farho MA, Assi BE. Intestinal obstruction induced by gallstone migration through unusual location of a cholecystogastric fistula: A rare case report. Int J Surg Case Rep. 2024;122:110149. Epub 20240810. doi: 10.1016/j.ijscr.2024.110149. PubMed PMID: 39137645; PubMed Central PMCID: PMCPMC11357798.	Excluded from the final analysis	The article was excluded from the analysis due to the lack of relation between the title and the abstract of the article with the subject under study
533	Ohtani T, Kawai C, Shirai Y, Kawakami K, Yoshida K, Hatakeyama K. Intraoperative ultrasonography versus cholangiography during laparoscopic cholecystectomy: A prospective comparative study. Journal of the American College of Surgeons. 1997;185(3):274-82. doi: 10.1016/s1072-7515(01)00928-0. PubMed PMID: WOS:A1997XU13000012.	Excluded from the final analysis	The article was excluded from the analysis due to the lack of relation between the title and the abstract of the article with the subject under study
534	Sheng BW, Zhao QB, Ma M, Zhang JQ. An inverse association of weight and the occurrence of asymptomatic gallbladder stone disease in hypercholesterolemia patients: a case-control study. Lipids in Health and Disease. 2020;19(1). doi: 10.1186/s12944-020-01402-8. PubMed PMID: WOS:000582646600001.	Excluded from the final analysis	The article was excluded from the analysis due to the lack of relation between the title and the abstract of the article with the subject under study
535	Li Y, Han H, You K, Ma C, Fan X. Investigating the association between blood cobalt and gallstones: a cross-sectional study utilizing NHANES data. Front Public Health. 2024;12:1363815. Epub 20240207. doi: 10.3389/fpubh.2024.1363815. PubMed PMID: 38384872; PubMed Central PMCID: PMCPMC10879586.	Excluded from the final analysis	The article was excluded from the analysis due to the lack of relation between the title and the abstract of the article with the subject under study
536	Jiang BQ, Zhong PH, Cheng XB, Yang XL, Yang J, Cao YF. Investigation of health and nutrition status of middle-aged and old residents in the urban district of Chongqing. Asia Pac J Clin Nutr. 2007;16 Suppl 1:17-21. PubMed PMID: 17392070.	Excluded from the final analysis	The article was excluded from the analysis due to the lack of relation between the title and the abstract of the article with the subject under study

537	Jiang BQ, Zhong PH, Cheng XB, Yang XL, Yang J, Cao YF. Investigation of health and nutrition status of middle-aged and old residents in the urban district of Chongqing. Asia Pacific Journal of Clinical Nutrition. 2007;16:17-21. PubMed PMID: WOS:000245926500004.	Excluded from the final analysis	The article was excluded from the analysis due to the lack of relation between the title and the abstract of the article with the subject under study
538	Sharma M, Ponnusamy RP. Is balloon sweeping detrimental in portal biliopathy? A report of 3 cases. Gastrointest Endosc. 2009;70(1):171-3. Epub 20090505. doi: 10.1016/j.gie.2008.11.002. PubMed PMID: 19409559.	Excluded from the final analysis	The article was excluded from the analysis due to the lack of relation between the title and the abstract of the article with the subject under study
539	Sharma M, Ponnusamy RP. Is balloon sweeping detrimental in portal biliopathy? A report of 3 cases. Gastrointestinal Endoscopy. 2009;70(1):171-3. doi: 10.1016/j.gie.2008.11.002. PubMed PMID: WOS:000267523200029.	Excluded from the final analysis	The article was excluded from the analysis due to the lack of relation between the title and the abstract of the article with the subject under study
540	Simi M, Schietroma M, Carlei F, Iannucci D, Cianca G, Leardi S. Is laparoscopic cholecystectomy a safe alternative to open cholecystectomy for pediatric patients with cholelithiasis? Endoscopy. 1996;28(3):312-5. doi: 10.1055/s-2007-1005462. PubMed PMID: WOS:A1996UM79300011.	Excluded from the final analysis	The article was excluded from the analysis due to the lack of relation between the title and the abstract of the article with the subject under study
541	Hussain A, Lafaurie G, Hafeez R, El-Hasani S. Is Specialisation Needed in Laparoscopic Cholecystectomy? A Retrospective Cohort Study of 5122 Patients. Chirurgia. 2020;115(6):756-66. doi: 10.21614/chirurgia.115.6.756. PubMed PMID: WOS:000604905000006.	Excluded from the final analysis	The article was excluded from the analysis due to the lack of relation between the title and the abstract of the article with the subject under study

542	Lonardo A, Grisendi A, Bonilauri S, Rambaldi M, Selmi I, Tondelli E. Ischaemic necrotizing pancreatitis after cardiac surgery. A case report and review of the literature. Italian Journal of Gastroenterology and Hepatology. 1999;31(9):872-5. PubMed PMID: WOS:000084809600009.	Excluded from the final analysis	The article was excluded from the analysis due to the lack of relation between the title and the abstract of the article with the subject under study
543	Hackert T, Hartwig W, Fritz S, Schneider L, Strobel O, Werner J. Ischemic acute pancreatitis: clinical features of 11 patients and review of the literature. Am J Surg. 2009;197(4):450-4. Epub 20080907. doi: 10.1016/j.amjsurg.2008.04.011. PubMed PMID: 18778810.	Excluded from the final analysis	The article was excluded from the analysis due to the lack of relation between the title and the abstract of the article with the subject under study
544	Paraf F, Fabiani JN, Laurian C, Bruneval P. [Ischemic cholecystitis from cholesterol crystal embolism]. Gastroenterol Clin Biol. 1999;23(5):577-80. PubMed PMID: 10429867.	Excluded from the final analysis	The article was excluded from the analysis due to the lack of relation between the title and the abstract of the article with the subject under study
545	Prasaad PR, Shekhar S, Priyadharshini SA. Isolated non-necrotising granulomatous vasculitis of the gall bladder- a rare entity. J Clin Diagn Res. 2014;8(10):Fd01-2. Epub 20141020. doi: 10.7860/jcdr/2014/8599.4932. PubMed PMID: 25478351; PubMed Central PMCID: PMCPMC4253169.	Excluded from the final analysis	The article was excluded from the analysis due to the lack of relation between the title and the abstract of the article with the subject under study
546	Sejeeni NF, Alfhmi S, Aljahdali S, Alzahrani S, Jaha R. Kawasaki Disease With Acute Acalculous Cholecystitis: A Case Report. Cureus. 2023;15(12):e49789. Epub 20231201. doi: 10.7759/cureus.49789. PubMed PMID: 38161523; PubMed Central PMCID: PMCPMC10757816.	Excluded from the final analysis	The article was excluded from the analysis due to the lack of relation between the title and the abstract of the article with the subject under study

547	Mostofsky E, Mukamal KJ, Giovannucci EL, Stampfer MJ, Rimm EB. Key Findings on Alcohol Consumption and a Variety of Health Outcomes From the Nurses' Health Study. Am J Public Health. 2016;106(9):1586-91. Epub 20160726. doi: 10.2105/ajph.2016.303336. PubMed PMID: 27459455; PubMed Central PMCID: PMCPMC4981808.	Excluded from the final analysis	The article was excluded from the analysis due to the lack of relation between the title and the abstract of the article with the subject under study
548	Mostofsky E, Mukamal KJ, Giovannucci EL, Stampfer MJ, Rimm EB. Key Findings on Alcohol Consumption and a Variety of Health Outcomes From the Nurses' Health Study. American Journal of Public Health. 2016;106(9):1586-91. doi: 10.2105/ajph.2016.303336. PubMed PMID: WOS:000388072300022.	Excluded from the final analysis	The article was excluded from the analysis due to the lack of relation between the title and the abstract of the article with the subject under study
549	Hekmat M, Taghipoor HR, Nobahar MR, Monfared MB, Tehrani MMM, Arabnia MK, et al. Laparascopic cholecystectomy and open-heart surgery at the same time. Journal of Cardiac Surgery. 2005;20(6):557-9. doi: 10.1111/j.1540-8191.2005.00096.x. PubMed PMID: WOS:000233541500013.	Excluded from the final analysis	The article was excluded from the analysis due to the lack of relation between the title and the abstract of the article with the subject under study
550	Gadacz TR, Talamini MA, Lillemoe KD, Yeo CJ. Laparoscopic cholecystectomy. Surg Clin North Am. 1990;70(6):1249-62. doi: 10.1016/s0039-6109(16)45282-5. PubMed PMID: 2147301.	Excluded from the final analysis	The article was excluded from the analysis due to the lack of relation between the title and the abstract of the article with the subject under study
551	Hugh TB, Chen FC, Hugh TJ, Li B. LAPAROSCOPIC CHOLECYSTECTOMY - A PROSPECTIVE-STUDY OF OUTCOME IN 100 UNSELECTED PATIENTS. Medical Journal of Australia. 1992;156(5):318-20. doi: 10.5694/j.1326-5377.1992.tb139786.x. PubMed PMID: WOS:A1992HG31100009.	Excluded from the final analysis	The article was excluded from the analysis due to the lack of relation between the title and the abstract of the article with the subject under study

552	Bitner M, Jaszewski R, Jander S, Maciejewski M. Laparoscopic cholecystectomy delayed by complicated myocardial infarction with papillary muscle rupture, and performed after unique complex mitral repair. Videosurgery and Other Miniinvasive Techniques. 2013;8(2):170-3. doi: 10.5114/wiitm.2011.32823. PubMed PMID: WOS:000321600400013.	Excluded from the final analysis	The article was excluded from the analysis due to the lack of relation between the title and the abstract of the article with the subject under study
553	Rumba R, Vanags A, Strumfa I, Pupkevics A, Pavars M. Laparoscopic Cholecystectomy for a Patient with a Lumboperitoneal Shunt: A Rare Case. Chirurgia (Bucur). 2016;111(3):263-5. PubMed PMID: 27452939.	Excluded from the final analysis	The article was excluded from the analysis due to the lack of relation between the title and the abstract of the article with the subject under study
554	Liu YY, Yeh CN, Lee HL, Chu PH, Jan YY, Chen MF. Laparoscopic Cholecystectomy for Gallbladder Disease in Patients with Severe Cardiovascular Disease. World Journal of Surgery. 2009;33(8):1720-6. doi: 10.1007/s00268-009-0072-9. PubMed PMID: WOS:000268779000027.	Excluded from the final analysis	The article was excluded from the analysis due to the lack of relation between the title and the abstract of the article with the subject under study
555	Collet D. Laparoscopic cholecystectomy in 1994 - Results of a prospective survey conducted by SFCERO on 4,624 cases. Surgical Endoscopy-Ultrasound and Interventional Techniques. 1997;11(1):56-63. doi: 10.1007/s004649900295. PubMed PMID: WOS:A1997WB61400014.	Excluded from the final analysis	The article was excluded from the analysis due to the lack of relation between the title and the abstract of the article with the subject under study
556	Leandros E, Albanopoulos K, Tsigris C, Archontovasilis F, Panoussopoulos SG, Skalistira M, et al. Laparoscopic cholecystectomy in cirrhotic patients with symptomatic gallstone disease. ANZ J Surg. 2008;78(5):363-5. doi: 10.1111/j.1445-2197.2008.04478.x. PubMed PMID: 18380734.	Excluded from the final analysis	The article was excluded from the analysis due to the lack of relation between the title and the abstract of the article with the subject under study

557	Alhamid MA, Ilie VC, Aioanei S, Stanciulea O, Minciuna CE, Lacatus M. Laparoscopic Cholecystectomy in Cirrhotic Patients: A Retrospective Study. Chirurgia (Bucur). 2021;116(1):34-41. doi: 10.21614/chirurgia.116.1.34. PubMed PMID: 33638324.	Excluded from the final analysis	The article was excluded from the analysis due to the lack of relation between the title and the abstract of the article with the subject under study
558	Hazzan D, Geron N, Golijanin D, Reissman P, Shiloni E. Laparoscopic cholecystectomy in octogenarians. Surgical Endoscopy and Other Interventional Techniques. 2003;17(5):773-6. doi: 10.1007/s00464-002-8529-z. PubMed PMID: WOS:000183421900023.	Excluded from the final analysis	The article was excluded from the analysis due to the lack of relation between the title and the abstract of the article with the subject under study
559	Dalvi AN, Deshpande AA, Doctor NH, Maydeo A, Bapat RD. Laparoscopic cholecystectomy in patient with portal cavernoma and portal hypertension. Indian J Gastroenterol. 2001;20(1):32-3. PubMed PMID: 11206876.	Excluded from the final analysis	The article was excluded from the analysis due to the lack of relation between the title and the abstract of the article with the subject under study
560	Isam SM, Ismail AA, Mohamed I, Suliman FS. Laparoscopic cholecystectomy in patients with bilharzial portal hypertension. Jsls. 2000;4(2):155-7. PubMed PMID: 10917123; PubMed Central PMCID: PMCPMC3015381.	Excluded from the final analysis	The article was excluded from the analysis due to the lack of relation between the title and the abstract of the article with the subject under study
561	Sasturkar SV, Agrawal N, Arora A, Kumar MPS, Kilambi R, Thapar S, Chattopadhyay TK. Laparoscopic cholecystectomy in patients with portal cavernoma without portal vein decompression. J Minim Access Surg. 2021;17(3):351-5. doi: 10.4103/jmas.JMAS_106_20. PubMed PMID: 32964890; PubMed Central PMCID: PMCPMC8270052.	Excluded from the final analysis	The article was excluded from the analysis due to the lack of relation between the title and the abstract of the article with the subject under study

562	Nan XY, Chan E, Wong KS, Ng J, Izwan S, Cooper M, Damodaran R. Laparoscopic Cholecystectomy in Pregnancy: A Seven-Year Retrospective Study From an Australian Tertiary Center. Cureus Journal of Medical Science. 2023;15(12). doi: 10.7759/cureus.50034. PubMed PMID: WOS:001123983700027.	Excluded from the final analysis	The article was excluded from the analysis due to the lack of relation between the title and the abstract of the article with the subject under study
563	Malik AM, Laghari AA, Talpur KA, Memon A, Pathan R, Memon JM. Laparoscopic cholecystectomy in the elderly patients. An experience at Liaquat University Hospital Jamshoro. J Ayub Med Coll Abbottabad. 2007;19(4):45-8. PubMed PMID: 18693597.	Excluded from the final analysis	The article was excluded from the analysis due to the lack of relation between the title and the abstract of the article with the subject under study
564	Yuksek YN, Akat AZ, Gozalan U, Daglar G, Pala Y, Canturk M, et al. Laparoscopic cholecystectomy under spinal anesthesia. Am J Surg. 2008;195(4):533-6. doi: 10.1016/j.amjsurg.2007.05.043. PubMed PMID: 18304510.	Excluded from the final analysis	The article was excluded from the analysis due to the lack of relation between the title and the abstract of the article with the subject under study
565	Imbelloni LE, Sant'anna R, Fornasari M, Fialho JC. Laparoscopic cholecystectomy under spinal anesthesia: comparative study between conventional-dose and low-dose hyperbaric bupivacaine. Local Reg Anesth. 2011;4:41-6. Epub 20111003. doi: 10.2147/lra.S19979. PubMed PMID: 22915892; PubMed Central PMCID: PMCPMC3417972.	Excluded from the final analysis	The article was excluded from the analysis due to the lack of relation between the title and the abstract of the article with the subject under study
566	Kano N, Yamakawa T, Ishikawa Y, Sakai S, Honda H, Kasugai H, Tachibana A. Laparoscopic cholecystectomy: a report of 409 consecutive cases and its future outlook. Surg Today. 1994;24(5):399-402. doi: 10.1007/bf01427031. PubMed PMID: 8054809.	Excluded from the final analysis	The article was excluded from the analysis due to the lack of relation between the title and the abstract of the article with the subject under study

567	Shamiyeh A, Wayand W. Laparoscopic cholecystectomy: early and late complications and their treatment. Langenbecks Archives of Surgery. 2004;389(3):164-71. doi: 10.1007/s00423-004-0470-2. PubMed PMID: WOS:000221885200002.	Excluded from the final analysis	The article was excluded from the analysis due to the lack of relation between the title and the abstract of the article with the subject under study
568	Haider G, Hussain D, Waheed S, Shah R, Khan AA, Ibrahim M, Shah SM. LAPAROSCOPIC CHOLECYSTECTOMY: OUTCOME OF FIRST 202 CASES IN A DISTRICT HOSPITAL IN GILGIT. J Ayub Med Coll Abbottabad. 2015;27(3):689-91. PubMed PMID: 26721041.	Excluded from the final analysis	The article was excluded from the analysis due to the lack of relation between the title and the abstract of the article with the subject under study
569	Jensen SL, Jensen PF, Wara P, Rokkjaer M. [Laparoscopic cholecystectomy. The first 45 operations]. Ugeskr Laeger. 1991;153(46):3225-8. PubMed PMID: 1835552.	Excluded from the final analysis	The article was excluded from the analysis due to the lack of relation between the title and the abstract of the article with the subject under study
570	Waninger J, Salm R, van Hüllen C, Farthmann EH. [Laparoscopic cholecystectomy]. Fortschr Med. 1991;109(19):392-6. PubMed PMID: 1833296.	Excluded from the final analysis	The article was excluded from the analysis due to the lack of relation between the title and the abstract of the article with the subject under study
571	Li YX, Liu LL, Jiang ZJ, Sun JJ. Laparoscopic Common Bile Duct Exploration is a Safe and Effective Strategy for Elderly Patients. Indian Journal of Surgery. 2024. doi: 10.1007/s12262-024-04021-0. PubMed PMID: WOS:001140294200002.	Excluded from the final analysis	The article was excluded from the analysis due to the lack of relation between the title and the abstract of the article with the subject under study

572	Nomura Y, Sakai H, Akiba J, Hisaka T, Sato T, Goto Y, et al. Laparoscopic left hepatectomy for a patient with intrahepatic cholangiocarcinoma metastasis in the falciform ligament: a case report. BMC Surg. 2021;21(1):122. Epub 20210308. doi: 10.1186/s12893-021-01115-4. PubMed PMID: 33685435; PubMed Central PMCID: PMCPMC7941886.	Excluded from the final analysis	The article was excluded from the analysis due to the lack of relation between the title and the abstract of the article with the subject under study
573	Drouard F, PassoneSzerzyna N, Berthou JC. Laparoscopic treatment of common bile duct stones. Hepato-Gastroenterology. 1997;44(13):16-21. PubMed PMID: WOS:A1997WK98800005.	Excluded from the final analysis	The article was excluded from the analysis due to the lack of relation between the title and the abstract of the article with the subject under study
574	Stewart L. latrogenic Biliary Injuries Identification, Classification, and Management. Surgical Clinics of North America. 2014;94(2):297-+. doi: 10.1016/j.suc.2014.01.008. PubMed PMID: WOS:000335421100008.	Excluded from the final analysis	The article was excluded from the analysis due to the lack of relation between the title and the abstract of the article with the subject under study
575	Quek E, Monkman B, Madani Y. Lessons of the month 1: Mesenteric ischaemia secondary to infective endocarditis. Clinical Medicine. 2022;22(3):282-4. doi: 10.7861/clinmed.2022-0044. PubMed PMID: WOS:000828115000022.	Excluded from the final analysis	The article was excluded from the analysis due to the lack of relation between the title and the abstract of the article with the subject under study
576	Sachdeva S, Khan Z, Ansari MA, Khalique N, Anees A. Lifestyle and gallstone disease: scope for primary prevention. Indian J Community Med. 2011;36(4):263-7. doi: 10.4103/0970-0218.91327. PubMed PMID: 22279255; PubMed Central PMCID: PMCPMC3263145.	Excluded from the final analysis	The article was excluded from the analysis due to the lack of relation between the title and the abstract of the article with the subject under study

577	Hak AE, Choi HK. Lifestyle and gout. Current Opinion in Rheumatology. 2008;20(2):179-86. doi: 10.1097/BOR.0b013e3282f524a2. PubMed PMID: WOS:000253475200010.	Excluded from the final analysis	The article was excluded from the analysis due to the lack of relation between the title and the abstract of the article with the subject under study
578	Jeng KS, Chiang HJ, Shih SC. LIMITATIONS OF PERCUTANEOUS TRANS-HEPATIC CHOLANGIOSCOPY IN THE REMOVAL OF COMPLICATED BILIARY CALCULI. World Journal of Surgery. 1989;13(5):603-10. doi: 10.1007/bf01658880. PubMed PMID: WOS:A1989AT92100018.	Excluded from the final analysis	The article was excluded from the analysis due to the lack of relation between the title and the abstract of the article with the subject under study
579	Preiss D, Tikkanen MJ, Welsh P, Ford I, Lovato LC, Elam MB, et al. Lipid-Modifying Therapies and Risk of Pancreatitis A Meta-analysis. Jama-Journal of the American Medical Association. 2012;308(8):804-11. doi: 10.1001/jama.2012.8439. PubMed PMID: WOS:000307705300024.	Excluded from the final analysis	The article was excluded from the analysis due to the lack of relation between the title and the abstract of the article with the subject under study
580	Nahon S, Cadranel JF, Chazouilleres O, Biour M, Jouannaud V, Marteau P. Liver and inflammatory bowel disease. Gastroenterologie Clinique Et Biologique. 2009;33(5):370-81. doi: 10.1016/j.gcb.2009.02.037. PubMed PMID: WOS:000266576900002.	Excluded from the final analysis	The article was excluded from the analysis due to the lack of relation between the title and the abstract of the article with the subject under study
581	de Miranda Henriques MS, de Morais Villar EJ. The Liver and Polycystic Kidney Disease. In: Li X, editor. Polycystic Kidney Disease. Brisbane (AU): Codon Publications	Excluded from the final analysis	The article was excluded from the analysis due to the lack of relation between the title and the abstract of the article with the subject under study

582		Excluded from the final analysis	The article was excluded from the analysis due to the lack of relation between the title and the abstract of the article with the subject under study
583	Almashhrawi AA, Ahmed KT, Rahman RN, Hammoud GM, Ibdah JA. Liver diseases in pregnancy: diseases not unique to pregnancy. World J Gastroenterol. 2013;19(43):7630-8. doi: 10.3748/wjg.v19.i43.7630. PubMed PMID: 24282352; PubMed Central PMCID: PMCPMC3837261.	Excluded from the final analysis	The article was excluded from the analysis due to the lack of relation between the title and the abstract of the article with the subject under study
584	Patrono D, Mazza E, Paraluppi G, Strignano P, David E, Romagnoli R, Salizzoni M. Liver transplantation for "mass-forming" sclerosing cholangitis after laparoscopic cholecystectomy. Int J Surg Case Rep. 2013;4(10):907-10. Epub 20130803. doi: 10.1016/j.ijscr.2013.07.021. PubMed PMID: 23995476; PubMed Central PMCID: PMCPMC3785926.	Excluded from the final analysis	The article was excluded from the analysis due to the lack of relation between the title and the abstract of the article with the subject under study
585	Meyenberger C, Meierhofer U, MichelHarder C, Knuchel J, Wirth HP, Buhler H, et al. Long-term follow-up after treatment of common bile duct stones by extracorporeal shock-wave lithotripsy. Endoscopy. 1996;28(5):411-7. doi: 10.1055/s-2007-1005502. PubMed PMID: WOS:A1996UZ41600001.	Excluded from the final analysis	The article was excluded from the analysis due to the lack of relation between the title and the abstract of the article with the subject under study
586	Nio M, Ohi R, Shimaoka S, Iwami D, Sano N. [Long-term outcome of surgery for biliary atresia]. Nihon Geka Gakkai Zasshi. 1996;97(8):637-41. PubMed PMID: 8905814.	Excluded from the final analysis	The article was excluded from the analysis due to the lack of relation between the title and the abstract of the article with the subject under study

587	Dakour Aridi H, Alami R, Tamim H, Shamseddine G, Fouani T, Safadi B. Long-term outcomes of laparoscopic sleeve gastrectomy: a Lebanese center experience. Surg Obes Relat Dis. 2016;12(9):1689-96. Epub 20151127. doi: 10.1016/j.soard.2015.11.025. PubMed PMID: 26948944.	Excluded from the final analysis	The article was excluded from the analysis due to the lack of relation between the title and the abstract of the article with the subject under study
588	Bates T, Harrison M, Lowe D, Lawson C, Padley N. LONGITUDINAL-STUDY OF GALL STONE PREVALENCE AT NECROPSY. Gut. 1992;33(1):103-7. doi: 10.1136/gut.33.1.103. PubMed PMID: WOS:A1992HA21200020.	Excluded from the final analysis	The article was excluded from the analysis due to the lack of relation between the title and the abstract of the article with the subject under study
589	Nagamine Y, Godai K, Oki H, Kanmura Y. Management of a centenarian who underwent emergency laparoscopic cholecystectomy under general anesthesia with subcostal transversus abdominis plane block. JA Clin Rep. 2016;2(1):24. Epub 20160923. doi: 10.1186/s40981-016-0050-9. PubMed PMID: 29497679; PubMed Central PMCID: PMCPMC5818852.	Excluded from the final analysis	The article was excluded from the analysis due to the lack of relation between the title and the abstract of the article with the subject under study
590	Bougard M, Barbier L, Godart B, Le Bayon-Bréard AG, Marques F, Salamé E. Management of biliary acute pancreatitis. Journal of Visceral Surgery. 2019;156(2):113-25. doi: 10.1016/j.jviscsurg.2018.08.002. PubMed PMID: WOS:000464972200006.	Excluded from the final analysis	The article was excluded from the analysis due to the lack of relation between the title and the abstract of the article with the subject under study
591	Jeffrey GP, Brind AM, Ormonde DG, Frazer CK, Ferguson J, Bell R, et al. Management of biliary tract complications following liver transplantation. Aust N Z J Surg. 1999;69(10):717-22. doi: 10.1046/j.1440-1622.1999.01671.x. PubMed PMID: 10527348.	Excluded from the final analysis	The article was excluded from the analysis due to the lack of relation between the title and the abstract of the article with the subject under study

592	Jeffrey GP, Brind AM, Ormonde DG, Frazer CK, Ferguson J, Bell R, et al. Management of biliary tract complications following liver transplantation. Australian and New Zealand Journal of Surgery. 1999;69(10):717-22. doi: 10.1046/j.1440-1622.1999.01671.x. PubMed PMID: WOS:000082845800009.	Excluded from the final analysis	The article was excluded from the analysis due to the lack of relation between the title and the abstract of the article with the subject under study
593	Yachha SK, Chetri K, Saraswat VA, Baijal SS, Sikora SS, Lal R, Srivastava A. Management of childhood pancreatic disorders: A multidisciplinary approach. Journal of Pediatric Gastroenterology and Nutrition. 2003;36(2):206-12. doi: 10.1097/00005176-200302000-00009. PubMed PMID: WOS:000180783800009.	Excluded from the final analysis	The article was excluded from the analysis due to the lack of relation between the title and the abstract of the article with the subject under study
594	Dakour Aridi H, Sultanem S, Abtar H, Safadi BY, Fawal H, Alami RS. Management of gallbladder disease after sleeve gastrectomy in a selected Lebanese population. Surg Obes Relat Dis. 2016;12(7):1300-4. Epub 20160201. doi: 10.1016/j.soard.2016.01.029. PubMed PMID: 27178610.	Excluded from the final analysis	The article was excluded from the analysis due to the lack of relation between the title and the abstract of the article with the subject under study
595	van As AB, Millar AJW. Management of paediatric liver trauma. Pediatric Surgery International. 2017;33(4):445-53. doi: 10.1007/s00383-016-4046-3. PubMed PMID: WOS:000398560900007.	Excluded from the final analysis	The article was excluded from the analysis due to the lack of relation between the title and the abstract of the article with the subject under study
596	Kaushik N, Dasari V, Jain D. Management of Pancreatic Calculi in Chronic Pancreatitis: A Review Article. Cureus. 2023;15(3):e35788. Epub 20230305. doi: 10.7759/cureus.35788. PubMed PMID: 37025704; PubMed Central PMCID: PMCPMC10072785.	Excluded from the final analysis	The article was excluded from the analysis due to the lack of relation between the title and the abstract of the article with the subject under study

597	Gupta V, Gupta V, Joshi P, Kumar S, Kulkarni R, Chopra N, et al. Management of post cholecystectomy vascular injuries. Surgeon-Journal of the Royal Colleges of Surgeons of Edinburgh and Ireland. 2019;17(6):326-33. doi: 10.1016/j.surge.2018.10.002. PubMed PMID: WOS:000501646500002.	Excluded from the final analysis	The article was excluded from the analysis due to the lack of relation between the title and the abstract of the article with the subject under study
598	Jin S, Fu Q, Wuyun G, Tu WY. Management of post-hepatectomy complications. World Journal of Gastroenterology. 2013;19(44):7983-91. doi: 10.3748/wjg.v19.i44.7983. PubMed PMID: WOS:000327519300017.	Excluded from the final analysis	The article was excluded from the analysis due to the lack of relation between the title and the abstract of the article with the subject under study
599	Grace RF, Barcellini W. Management of pyruvate kinase deficiency in children and adults. Blood. 2020;136(11):1241-9. doi: 10.1182/blood.2019000945. PubMed PMID: 32702739.	Excluded from the final analysis	The article was excluded from the analysis due to the lack of relation between the title and the abstract of the article with the subject under study
600	Terada T, Terai T, Yamawaki T. Marked diffuse dilations of the biliary tree associated with intrahepatic calculi, biliary sludges and a mucinous cyst of the pancreatic head in a 99-year-old woman. Pathology International. 2003;53(8):563-8. doi: 10.1046/j.1440-1827.2003.01505.x. PubMed PMID: WOS:000184518900011.	Excluded from the final analysis	The article was excluded from the analysis due to the lack of relation between the title and the abstract of the article with the subject under study
601	Chan AW, Sabaratnam RM, Pillay Y. Massive gallstone in an asymptomatic Indigenous Canadian male: Case report and literature review. International Journal of Surgery Case Reports. 2020;72:429-32. doi: 10.1016/j.ijscr.2020.06.028. PubMed PMID: WOS:000548926300020.	Excluded from the final analysis	The article was excluded from the analysis due to the lack of relation between the title and the abstract of the article with the subject under study

602	Joo YE, Kim HS, Choi SK, Rew JS, Kim HR, Kim SJ. Massive gastrointestinal bleeding from jejunal varices. Journal of Gastroenterology. 2000;35(10):775-8. doi: 10.1007/s005350070037. PubMed PMID: WOS:000089858500008.	Excluded from the final analysis	The article was excluded from the analysis due to the lack of relation between the title and the abstract of the article with the subject under study
603	Syme RG, Thomas EJ. Massive hemoperitoneum from transhepatic perforation of the gallbladder: a rare complication of cholelithiasis. Surgery. 1989;105(4):556-9. PubMed PMID: 2928958.	Excluded from the final analysis	The article was excluded from the analysis due to the lack of relation between the title and the abstract of the article with the subject under study
604	Kelly SB, Gauhar T, Pollard R. Massive intraperitoneal hemorrhage from a pancreatic pseudocyst. Am J Gastroenterol. 1999;94(12):3638-41. doi: 10.1111/j.1572-0241.1999.01459.x. PubMed PMID: 10606335.	Excluded from the final analysis	The article was excluded from the analysis due to the lack of relation between the title and the abstract of the article with the subject under study
605	Himi ST, Monalisa NT, Whaiduzzaman MD, Barros A, Uddin MS. MedAi: A Smartwatch-Based Application Framework for the Prediction of Common Diseases Using Machine Learning. Ieee Access. 2023;11:12342-59. doi: 10.1109/access.2023.3236002. PubMed PMID: WOS:000935635300001.	Excluded from the final analysis	The article was excluded from the analysis due to the lack of relation between the title and the abstract of the article with the subject under study
606	Rosato V, Bosetti C, Dal Maso L, Montella M, Serraino D, Negri E, La Vecchia C. Medical conditions, family history of cancer, and the risk of biliary tract cancers. Tumori. 2016;2016(3):252-7. Epub 20150804. doi: 10.5301/tj.5000397. PubMed PMID: 26350180.	Excluded from the final analysis	The article was excluded from the analysis due to the lack of relation between the title and the abstract of the article with the subject under study

607	Barnard ND, Nicholson A, Howard JL. The medical costs attributable to meat consumption. Prev Med. 1995;24(6):646-55. doi: 10.1006/pmed.1995.1100. PubMed PMID: 8610089.	Excluded from the final analysis	The article was excluded from the analysis due to the lack of relation between the title and the abstract of the article with the subject under study
608	Barnard ND, Nicholson A, Howard JL. THE MEDICAL COSTS ATTRIBUTABLE TO MEAT CONSUMPTION. Preventive Medicine. 1995;24(6):646-55. doi: 10.1006/pmed.1995.1100. PubMed PMID: WOS:A1995TJ31900015.	Excluded from the final analysis	The article was excluded from the analysis due to the lack of relation between the title and the abstract of the article with the subject under study
609	Pi-Sunyer FX. The medical risks of obesity. Obes Surg. 2002;12 Suppl 1:6s-11s. doi: 10.1007/bf03342140. PubMed PMID: 11969107.	Excluded from the final analysis	The article was excluded from the analysis due to the lack of relation between the title and the abstract of the article with the subject under study
610	Pi-Sunyer FX. The medical risks of obesity. Obesity Surgery. 2002;12:6S-11S. doi: 10.1007/bf03342140. PubMed PMID: WOS:000175053000003.	Excluded from the final analysis	The article was excluded from the analysis due to the lack of relation between the title and the abstract of the article with the subject under study
611	Lee YM, Kaplan MM. Medical treatment of primary sclerosing cholangitis. J Hepatobiliary Pancreat Surg. 1999;6(4):361-5. doi: 10.1007/s005340050131. PubMed PMID: 10664282.	Excluded from the final analysis	The article was excluded from the analysis due to the lack of relation between the title and the abstract of the article with the subject under study

612	Turunen K, Helander K, Mattila KJ, Sumanen M. Menopause after a history of intrahepatic cholestasis of pregnancy. Menopause-the Journal of the North American Menopause Society. 2013;20(11):1200-3. doi: 10.1097/gme.0000000000000101. PubMed PMID: WOS:000330465000015.	Excluded from the final analysis	The article was excluded from the analysis due to the lack of relation between the title and the abstract of the article with the subject under study
613	Grundy SM. Metabolic complications of obesity. Endocrine. 2000;13(2):155-65. doi: 10.1385/endo:13:2:155. PubMed PMID: WOS:000165136100004.	Excluded from the final analysis	The article was excluded from the analysis due to the lack of relation between the title and the abstract of the article with the subject under study
614	Chen LY, Qiao QH, Zhang SC, Chen YH, Chao GQ, Fang LZ. Metabolic syndrome and gallstone disease. World J Gastroenterol. 2012;18(31):4215-20. doi: 10.3748/wjg.v18.i31.4215. PubMed PMID: 22919256; PubMed Central PMCID: PMCPMC3422804.	Excluded from the final analysis	The article was excluded from the analysis due to the lack of relation between the title and the abstract of the article with the subject under study
615	Chen LY, Qiao QH, Zhang SC, Chen YH, Chao GQ, Fang LZ. Metabolic syndrome and gallstone disease. World Journal of Gastroenterology. 2012;18(31):4215-20. doi: 10.3748/wjg.v18.i31.4215. PubMed PMID: WOS:000307986800020.	Excluded from the final analysis	The article was excluded from the analysis due to the lack of relation between the title and the abstract of the article with the subject under study
616	Ata N, Kucukazman M, Yavuz B, Bulus H, Dal K, Ertugrul DT, et al. The metabolic syndrome is associated with complicated gallstone disease. Canadian Journal of Gastroenterology and Hepatology. 2011;25(5):274-6. doi: 10.1155/2011/356761. PubMed PMID: WOS:000290448100009.	Excluded from the final analysis	The article was excluded from the analysis due to the lack of relation between the title and the abstract of the article with the subject under study

617	Sare M, Gurer S, Taskin V, Aladag M, Hilmioglu F, Gurel M. Mirizzi syndrome: Choice of surgical procedure in the laparoscopic era. Surgical Laparoscopy & Endoscopy. 1998;8(1):63-7. doi: 10.1097/00019509-199802000-00015. PubMed PMID: WOS:000071917300015.	Excluded from the final analysis	The article was excluded from the analysis due to the lack of relation between the title and the abstract of the article with the subject under study
618	Goldberg DM, Soleas GJ, Levesque M. Moderate alcohol consumption: The gentle face of Janus. Clinical Biochemistry. 1999;32(7):505-18. doi: 10.1016/s0009-9120(99)00051-x. PubMed PMID: WOS:000084249900003.	Excluded from the final analysis	The article was excluded from the analysis due to the lack of relation between the title and the abstract of the article with the subject under study
619	Lee YC, Wu JS, Yang YC, Chang CS, Lu FH, Chang CJ. Moderate to severe, but not mild, nonalcoholic fatty liver disease associated with increased risk of gallstone disease. Scand J Gastroenterol. 2014;49(8):1001-6. Epub 20140703. doi: 10.3109/00365521.2014.920912. PubMed PMID: 24989169.	Excluded from the final analysis	The article was excluded from the analysis due to the lack of relation between the title and the abstract of the article with the subject under study
620	Liu B, Cao PK, Wang YZ, Wang WJ, Tian SL, Hertzanu Y, Li YL. Modified percutaneous transhepatic papillary balloon dilation for patients with refractory hepatolithiasis. World Journal of Gastroenterology. 2020;26(27):3929-37. doi: 10.3748/wjg.v26.i27.3929. PubMed PMID: WOS:000557249100006.	Excluded from the final analysis	The article was excluded from the analysis due to the lack of relation between the title and the abstract of the article with the subject under study
621	Bianchi P, Fermo E. Molecular heterogeneity of pyruvate kinase deficiency. Haematologica. 2020;105(9):2218-28. Epub 20200901. doi: 10.3324/haematol.2019.241141. PubMed PMID: 33054047; PubMed Central PMCID: PMCPMC7556514.	Excluded from the final analysis	The article was excluded from the analysis due to the lack of relation between the title and the abstract of the article with the subject under study

622	Kreft I, Vollmannová A, Lidiková J, Musilová J, Germ M, Golob A, et al. Molecular Shield for Protection of Buckwheat Plants from UV-B Radiation. Molecules. 2022;27(17). Epub 20220830. doi: 10.3390/molecules27175577. PubMed PMID: 36080352; PubMed Central PMCID: PMCPMC9457819.	Excluded from the final analysis	The article was excluded from the analysis due to the lack of relation between the title and the abstract of the article with the subject under study
623	Elmfeldt D, Vedin A, Wilhelmsson C, Tibblin G, Wilhelmsen L. MORBIDITY IN REPRESENTATIVE MALE SURVIVORS OF MYOCARDIAL-INFARCTION COMPARED TO REPRESENTATIVE POPULATION SAMPLES. Journal of Chronic Diseases. 1976;29(4):221-31. doi: 10.1016/0021-9681(76)90076-x. PubMed PMID: WOS:A1976BR95400002.	Excluded from the final analysis	The article was excluded from the analysis due to the lack of relation between the title and the abstract of the article with the subject under study
624	Huang X, Chen M, Tan H, Xiao S, Deng J. The morbidity rate of chronic disease among Chinese rural residents: results from Liuyang cohort. Med Princ Pract. 2013;22(4):362-7. Epub 20130125. doi: 10.1159/000346437. PubMed PMID: 23364269; PubMed Central PMCID: PMCPMC5586756.	Excluded from the final analysis	The article was excluded from the analysis due to the lack of relation between the title and the abstract of the article with the subject under study
625	Huang X, Chen MS, Tan HZ, Xiao SY, Deng J. The Morbidity Rate of Chronic Disease among Chinese Rural Residents: Results from Liuyang Cohort. Medical Principles and Practice. 2013;22(4):362-7. doi: 10.1159/000346437. PubMed PMID: WOS:000322526200009.	Excluded from the final analysis	The article was excluded from the analysis due to the lack of relation between the title and the abstract of the article with the subject under study
626	Soustek Z. MORPHOLOGY OF CHOLECYSTITIS AND GALLSTONE FORMATION. Acta Morphologica Academiae Scientiarum Hungaricae. 1981;29(2-3):127-40. PubMed PMID: WOS:A1981MP17100002.	Excluded from the final analysis	The article was excluded from the analysis due to the lack of relation between the title and the abstract of the article with the subject under study

627	Neri E, Boraschi P, Braccini G, Caramella D, Perri G, Bartolozzi C. MR virtual endoscopy of the pancreaticobiliary tract. Magnetic Resonance Imaging. 1999;17(1):59-67. doi: 10.1016/s0730-725x(98)00127-1. PubMed PMID: WOS:000077953600007.	Excluded from the final analysis	The article was excluded from the analysis due to the lack of relation between the title and the abstract of the article with the subject under study
628	Reddy S, Vendrami CL, Mittal P, Borhani AA, Moreno CC, Miller FH. MRI evaluation of bile duct injuries and other post-cholecystectomy complications. Abdominal Radiology. 2021;46(7):3086-104. doi: 10.1007/s00261-020-02947-z. PubMed PMID: WOS:000617425400001.	Excluded from the final analysis	The article was excluded from the analysis due to the lack of relation between the title and the abstract of the article with the subject under study
629	Pu Y, Luo Y. Multi-modal imaging for the diagnosis of spontaneous visceral artery dissection: A case report. World J Gastrointest Surg. 2024;16(5):1430-5. doi: 10.4240/wjgs.v16.i5.1430. PubMed PMID: 38817294; PubMed Central PMCID: PMCPMC11135292.	Excluded from the final analysis	The article was excluded from the analysis due to the lack of relation between the title and the abstract of the article with the subject under study
630	Fan J, Sun Z, Yu C, Guo Y, Pei P, Yang L, et al. Multimorbidity patterns and association with mortality in 0.5 million Chinese adults. Chin Med J (Engl). 2022;135(6):648-57. Epub 20220320. doi: 10.1097/cm9.0000000000001985. PubMed PMID: 35191418; PubMed Central PMCID: PMCPMC9276333.	Excluded from the final analysis	The article was excluded from the analysis due to the lack of relation between the title and the abstract of the article with the subject under study
631	Fan JN, Sun ZJ, Yu CQ, Guo Y, Pei P, Yang L, et al. Multimorbidity patterns and association with mortality in 0.5 million Chinese adults. Chinese Medical Journal. 2022;135(6):648-57. doi: 10.1097/cm9.0000000000001985. PubMed PMID: WOS:000787350600004.	Excluded from the final analysis	The article was excluded from the analysis due to the lack of relation between the title and the abstract of the article with the subject under study

632	Sundaram SS, Sokol RJ. The Multiple Facets of ABCB4 (MDR3) Deficiency. Curr Treat Options Gastroenterol. 2007;10(6):495-503. doi: 10.1007/s11938-007-0049-4. PubMed PMID: 18221610; PubMed Central PMCID: PMCPMC3888315.	Excluded from the final analysis	The article was excluded from the analysis due to the lack of relation between the title and the abstract of the article with the subject under study
633	Cher DJ. Myocardial infarction and acute cholecystitis: Application of sequence symmetry analysis. Epidemiology. 2000;11(4):446-9. doi: 10.1097/00001648-200007000-00014. PubMed PMID: WOS:000087713600014.	Excluded from the final analysis	The article was excluded from the analysis due to the lack of relation between the title and the abstract of the article with the subject under study
634	J F, C S, A RF, M LG, M G. Myotonic Dystrophy-2: Unusual Phenotype Due to a Small CCTG-expansion. Balkan J Med Genet. 2018;21(2):39-43. Epub 20181231. doi: 10.2478/bjmg-2018-0024. PubMed PMID: 30984523; PubMed Central PMCID: PMCPMC6454246.	Excluded from the final analysis	The article was excluded from the analysis due to the lack of relation between the title and the abstract of the article with the subject under study
635	Kumar M, Saraswat VA. Natural history of portal cavernoma cholangiopathy. J Clin Exp Hepatol. 2014;4(Suppl 1):S62-6. Epub 20130827. doi: 10.1016/j.jceh.2013.08.003. PubMed PMID: 25755597; PubMed Central PMCID: PMCPMC4244826.	Excluded from the final analysis	The article was excluded from the analysis due to the lack of relation between the title and the abstract of the article with the subject under study
636	Borgna-Pignatti C, Marsella M, Zanforlin N. The natural history of thalassemia intermedia. Ann N Y Acad Sci. 2010;1202:214-20. doi: 10.1111/j.1749-6632.2010.05550.x. PubMed PMID: 20712795.	Excluded from the final analysis	The article was excluded from the analysis due to the lack of relation between the title and the abstract of the article with the subject under study

637	Hirano T, Manabe T. A new experimental model for gallstone pancreatitis: short-termed pancreatico-biliary duct obstruction and exocrine stimulation with systemic hypotension in rats. Nihon Geka Hokan. 1993;62(1):3-15. PubMed PMID: 8352638.	Excluded from the final analysis	The article was excluded from the analysis due to the lack of relation between the title and the abstract of the article with the subject under study
638	Klar E, Werner J. New pathophysiological findings on acute pancreatitis. Chirurg. 2000;71(3):253-64. PubMed PMID: WOS:000086175800002.	Excluded from the final analysis	The article was excluded from the analysis due to the lack of relation between the title and the abstract of the article with the subject under study
639	Hall CM, Jupiter DC, Regner JL. Newly diagnosed and decompensated congestive heart failure is associated with increased rates of pneumonia, reintubation, and death following laparoscopic cholecystectomy: A NSQIP database review of 143,761 patients. International Journal of Surgery. 2016;35:209-13. doi: 10.1016/j.ijsu.2016.10.005. PubMed PMID: WOS:000386903200033.	Excluded from the final analysis	The article was excluded from the analysis due to the lack of relation between the title and the abstract of the article with the subject under study
640	Reddy SK, Zhan M, Alexander HR, El-Kamary SS. Nonalcoholic fatty liver disease is associated with benign gastrointestinal disorders. World J Gastroenterol. 2013;19(45):8301-11. doi: 10.3748/wjg.v19.i45.8301. PubMed PMID: 24363521; PubMed Central PMCID: PMCPMC3857453.	Excluded from the final analysis	The article was excluded from the analysis due to the lack of relation between the title and the abstract of the article with the subject under study
641	Pedersen SB, Langsted A, Nordestgaard BG. Nonfasting Mild-to-Moderate Hypertriglyceridemia and Risk of Acute Pancreatitis. JAMA Intern Med. 2016;176(12):1834-42. doi: 10.1001/jamainternmed.2016.6875. PubMed PMID: 27820614.	Excluded from the final analysis	The article was excluded from the analysis due to the lack of relation between the title and the abstract of the article with the subject under study

642	Pedersen SB, Langsted A, Nordestgaard BG. Nonfasting Mild-to-Moderate Hypertriglyceridemia and Risk of Acute Pancreatitis. Jama Internal Medicine. 2016;176(12):1834-42. doi: 10.1001/jamainternmed.2016.6875. PubMed PMID: WOS:000390255700021.	Excluded from the final analysis	The article was excluded from the analysis due to the lack of relation between the title and the abstract of the article with the subject under study
643	Oldenkamp CL, Kitamura K. Nonobstetric Surgical Emergencies in Pregnancy. Emerg Med Clin North Am. 2023;41(2):259-67. Epub 20230209. doi: 10.1016/j.emc.2023.01.001. PubMed PMID: 37024162.	Excluded from the final analysis	The article was excluded from the analysis due to the lack of relation between the title and the abstract of the article with the subject under study
644	Wood OB, Popovich NG. Nonpharmacologic treatment of obesity. J Am Pharm Assoc (Wash). 1996;Ns36(11):636-50. doi: 10.1016/s1086-5802(16)30148-6. PubMed PMID: 8952251.	Excluded from the final analysis	The article was excluded from the analysis due to the lack of relation between the title and the abstract of the article with the subject under study
645	Lenchur PD, Frishman WH. A Novel Approach to Calcium Destruction in Coronary and Peripheral Blood Vessels: Intravascular Lithotripsy. Cardiology in Review. 2024;32(6):566-71. doi: 10.1097/crd.0000000000000514. PubMed PMID: WOS:001336412100005.	Excluded from the final analysis	The article was excluded from the analysis due to the lack of relation between the title and the abstract of the article with the subject under study
646	Hernandez-Anzaldo S, Brglez V, Hemmeryckx B, Leung D, Filep JG, Vance JE, et al. Novel Role for Matrix Metalloproteinase 9 in Modulation of Cholesterol Metabolism. Journal of the American Heart Association. 2016;5(10). doi: 10.1161/jaha.116.004228. PubMed PMID: WOS:000386748500049.	Excluded from the final analysis	The article was excluded from the analysis due to the lack of relation between the title and the abstract of the article with the subject under study

647	Tsai CH, Wu JS, Chang YF, Lu FH, Yang YC, Chang CJ. The Number of Metabolic Abnormalities Associated with the Risk of Gallstones in a Non-diabetic Population. Plos One. 2014;9(3). doi: 10.1371/journal.pone.0090310. PubMed PMID: WOS:000332479400062.	Excluded from the final analysis	The article was excluded from the analysis due to the lack of relation between the title and the abstract of the article with the subject under study
648	Zommara MA, Swelam S, Raya-Alvarez E, Imaizumi K, Elmahdy A, Alkhudhayri DA, et al. Nutritional and potential health benefits of chufa oil, olive oil, and anhydrous milk fat against gallstone disease in a C57BL/6N mouse model. Frontiers in Nutrition. 2024;11. doi: 10.3389/fnut.2024.1445484. PubMed PMID: WOS:001328995900001.	Excluded from the final analysis	The article was excluded from the analysis due to the lack of relation between the title and the abstract of the article with the subject under study
649	Sabaté J, Ang Y. Nuts and health outcomes: new epidemiologic evidence. American Journal of Clinical Nutrition. 2009;89(5):S1643-S8. doi: 10.3945/ajcn.2009.26736Q. PubMed PMID: WOS:000265394300056.	Excluded from the final analysis	The article was excluded from the analysis due to the lack of relation between the title and the abstract of the article with the subject under study
650	Dietz WH. Obesity. J Am Coll Nutr. 1989;8 Suppl:13s-21s. doi: 10.1080/07315724.1989.10737966. PubMed PMID: 2681326.	Excluded from the final analysis	The article was excluded from the analysis due to the lack of relation between the title and the abstract of the article with the subject under study
651	Neilson A, Schneider H. Obesity and its comorbidities: present and future importance on health status in Switzerland. Soz Praventivmed. 2005;50(2):78-86. doi: 10.1007/s00038-004-4066-y. PubMed PMID: 15900960.	Excluded from the final analysis	The article was excluded from the analysis due to the lack of relation between the title and the abstract of the article with the subject under study

652	Neilson A, Schneider H. Obesity and its comorbidities: present and future importance on health status in Switzerland. Sozial-Und Praventivmedizin. 2005;50(2):78-86. doi: 10.1007/s00038-004-4066-y. PubMed PMID: WOS:000234046000004.	Excluded from the final analysis	The article was excluded from the analysis due to the lack of relation between the title and the abstract of the article with the subject under study
653	Frankenburg FR, Zanarini MC. Obesity and obesity-related illnesses in borderline patients. J Pers Disord. 2006;20(1):71-80. doi: 10.1521/pedi.2006.20.1.71. PubMed PMID: 16563080.	Excluded from the final analysis	The article was excluded from the analysis due to the lack of relation between the title and the abstract of the article with the subject under study
654	Ashwell M. Obesity in men and women. Int J Obes Relat Metab Disord. 1994;18 Suppl 1:S1-7. PubMed PMID: 8087159.	Excluded from the final analysis	The article was excluded from the analysis due to the lack of relation between the title and the abstract of the article with the subject under study
655	Drew BS, Dixon AF, Dixon JB. Obesity management: update on orlistat. Vasc Health Risk Manag. 2007;3(6):817-21. PubMed PMID: 18200802; PubMed Central PMCID: PMCPMC2350121.	Excluded from the final analysis	The article was excluded from the analysis due to the lack of relation between the title and the abstract of the article with the subject under study
656	Khurram M, Paracha SJ, Khar HT, Hasan Z. Obesity related complications in 100 obese subjects and their age matched controls. J Pak Med Assoc. 2006;56(2):50-3. PubMed PMID: 16555633.	Excluded from the final analysis	The article was excluded from the analysis due to the lack of relation between the title and the abstract of the article with the subject under study

657	Nelly Manrique M, Frisancho O, Zumaeta E, Palomino A, Rodriguez C. [Obscure digestive bleeding by ileal carcinoid tumor]. Rev Gastroenterol Peru. 2011;31(1):81-6. PubMed PMID: 21544161.	Excluded from the final analysis	The article was excluded from the analysis due to the lack of relation between the title and the abstract of the article with the subject under study
658	Singh RD, Mudang U, Abhinav K. Observation on Uncommon Complications of Acute Pancreatitis. Journal of Evolution of Medical and Dental Sciences-Jemds. 2020;9(8):488-91. doi: 10.14260/jemds/2020/110. PubMed PMID: WOS:000520042900005.	Excluded from the final analysis	The article was excluded from the analysis due to the lack of relation between the title and the abstract of the article with the subject under study
659	Riederer J. Obstructive jaundice due to sludge in the common bile duct. Deutsche Medizinische Wochenschrift. 2000;125(1-2):11-4. doi: 10.1055/s-2007-1023877. PubMed PMID: WOS:000084897400003.	Excluded from the final analysis	The article was excluded from the analysis due to the lack of relation between the title and the abstract of the article with the subject under study
660	Wang L, Jiang T, Zhao YH. [One-year outcomes of laparoscopic single-anastomosis duodenal-ileal bypass with sleeve gastrectomy versus laparoscopic sleeve gastrectomy for the treatment of obesity and obesity-related metabolic diseases]. Zhonghua Wei Chang Wai Ke Za Zhi. 2021;24(12):1058-64. doi: 10.3760/cma.j.cn441530-20210126-00044. PubMed PMID: 34923788.	Excluded from the final analysis	The article was excluded from the analysis due to the lack of relation between the title and the abstract of the article with the subject under study
661	Khan AR. Open laparoscopic access for primary trocar using modified Hasson's technique. Saudi Med J. 2003;24 Suppl:S21-4. PubMed PMID: 12778236.	Excluded from the final analysis	The article was excluded from the analysis due to the lack of relation between the title and the abstract of the article with the subject under study

662	Michek J, Wendsche P. [Operations in acute blockade of the common bile duct and the papilla]. Zentralbl Chir. 1983;108(16):1023-5. PubMed PMID: 6637211.	Excluded from the final analysis	The article was excluded from the analysis due to the lack of relation between the title and the abstract of the article with the subject under study
663	Hancock-Cerutti W, Rader DJ. Opposing Effects of ABCG5/8 Function on Myocardial Infarction and Gallstone Disease. Journal of the American College of Cardiology. 2014;63(20):2129-30. doi: 10.1016/j.jacc.2014.02.553. PubMed PMID: WOS:000336372700012.	Excluded from the final analysis	The article was excluded from the analysis due to the lack of relation between the title and the abstract of the article with the subject under study
664	Begos DG, Franco KL, Baldwin JC, Lee FA, Revkin JH, Modlin IM. OPTIMAL TIMING AND INDICATIONS FOR CHOLECYSTECTOMY IN CARDIAC TRANSPLANT PATIENTS. World Journal of Surgery. 1995;19(4):661-7. doi: 10.1007/bf00294752. PubMed PMID: WOS:A1995RK66500031.	Excluded from the final analysis	The article was excluded from the analysis due to the lack of relation between the title and the abstract of the article with the subject under study
665	Ugurlu ET. Our experiences in 1000 case single-centre endoscopic retrograde cholangiopancreatography. Journal of Minimal Access Surgery. 2023;19(1):85-94. doi: 10.4103/jmas.jmas_389_21. PubMed PMID: WOS:000976549300012.	Excluded from the final analysis	The article was excluded from the analysis due to the lack of relation between the title and the abstract of the article with the subject under study
666	Pålsson S, Saliba G, Sandblom G. Outcome after cholecystectomy in the elderly: a population-based register study. Scandinavian Journal of Gastroenterology. 2016;51(8):974-8. doi: 10.3109/00365521.2016.1166517. PubMed PMID: WOS:000377451700013.	Excluded from the final analysis	The article was excluded from the analysis due to the lack of relation between the title and the abstract of the article with the subject under study

667	Lykavieris P, Chardot C, Sokhn M, Gauthier F, Valayer J, Bernard O. Outcome in adulthood of biliary atresia: a study of 63 patients who survived for over 20 years with their native liver. Hepatology. 2005;41(2):366-71. doi: 10.1002/hep.20547. PubMed PMID: 15660386.	Excluded from the final analysis	The article was excluded from the analysis due to the lack of relation between the title and the abstract of the article with the subject under study
668	Bismar HA, Al-Salamah SM. Outcome of laparoscopic cholecystectomy in acute biliary pancreatitis. Saudi Medical Journal. 2003;24(6):660-4. PubMed PMID: WOS:000183769100017.	Excluded from the final analysis	The article was excluded from the analysis due to the lack of relation between the title and the abstract of the article with the subject under study
669	Nio M, Ohi R, Shimaoka S, Iwami D, Sano N. The outcome of surgery for biliary atresia and the current status of long-term survivors. Tohoku J Exp Med. 1997;181(1):235-44. doi: 10.1620/tjem.181.235. PubMed PMID: 9149360.	Excluded from the final analysis	The article was excluded from the analysis due to the lack of relation between the title and the abstract of the article with the subject under study
670	Nikfarjam M, Yeo D, Perini M, Fink MA, Muralidharan V, Starkey G, et al. Outcomes of cholecystectomy for treatment of acute cholecystitis in octogenarians. Anz Journal of Surgery. 2014;84(12):943-8. doi: 10.1111/ans.12313. PubMed PMID: WOS:000345850500014.	Excluded from the final analysis	The article was excluded from the analysis due to the lack of relation between the title and the abstract of the article with the subject under study
671	DiBianco JM, Lange J, Heidenberg D, Mufarrij P. Oxygen Venous Embolism After Hydrogen Peroxide Use During Percutaneous Nephrolithotomy. J Endourol Case Rep. 2019;5(1):25-7. Epub 20190318. doi: 10.1089/cren.2018.0111. PubMed PMID: 30989125; PubMed Central PMCID: PMCPMC6461058.	Excluded from the final analysis	The article was excluded from the analysis due to the lack of relation between the title and the abstract of the article with the subject under study

672	Sbeit W, Greener T, Kadah A, Mari A, Goldin E, Mahamid M, Khoury T. Pancreatic and hepatobiliary manifestations of nonalcoholic fatty pancreatic disease: a referral multi-center experience. Eur J Gastroenterol Hepatol. 2021;33(1S Suppl 1):e297-e301. doi: 10.1097/meg.0000000000002041. PubMed PMID: 33600093.	Excluded from the final analysis	The article was excluded from the analysis due to the lack of relation between the title and the abstract of the article with the subject under study
673	Harvey MH, Wedgwood KR, Austin JA, Reber HA. Pancreatic duct pressure, duct permeability and acute pancreatitis. Br J Surg. 1989;76(8):859-62. doi: 10.1002/bjs.1800760832. PubMed PMID: 2475200.	Excluded from the final analysis	The article was excluded from the analysis due to the lack of relation between the title and the abstract of the article with the subject under study
674	Challand C, Titcomb D, Armstrong CP. Pancreatic pseudocyst causing celiac artery trunk thrombosis. Jop. 2008;9(4):512-4. Epub 20080710. PubMed PMID: 18648144.	Excluded from the final analysis	The article was excluded from the analysis due to the lack of relation between the title and the abstract of the article with the subject under study
675	D LTH, Papo T, Laraki R, Wechsler B, Blétry O, Chapelon C, et al. [Pancreatitis in systemic lupus erythematosus. Review of the literature apropos of 5 cases]. Rev Med Interne. 1994;15(2):89-94. doi: 10.1016/s0248-8663(05)81180-4. PubMed PMID: 8059127.	Excluded from the final analysis	The article was excluded from the analysis due to the lack of relation between the title and the abstract of the article with the subject under study
676	Hasan B, Asif T, Braun C, Bahaj W, Dosokey E, Pauly RR. Pancreatitis in the Setting of Vaso-occlusive Sickle Cell Crisis: A Rare Encounter. Cureus. 2017;9(4):e1193. Epub 20170425. doi: 10.7759/cureus.1193. PubMed PMID: 28553571; PubMed Central PMCID: PMCPMC5444912.	Excluded from the final analysis	The article was excluded from the analysis due to the lack of relation between the title and the abstract of the article with the subject under study

677	Marques VL, Gormezano NW, Bonfá E, Aikawa NE, Terreri MT, Pereira RM, et al. Pancreatitis Subtypes Survey in 852 Childhood-Onset Systemic Lupus Erythematosus Patients. J Pediatr Gastroenterol Nutr. 2016;62(2):328-34. doi: 10.1097/mpg.0000000000000990. PubMed PMID: 26418213.	Excluded from the final analysis	The article was excluded from the analysis due to the lack of relation between the title and the abstract of the article with the subject under study
678	Culp WC, McCowan TC, DeValdenebro M, Wright LB, Workman JL, Culp WC, Jr. Paravertebral block: an improved method of pain control in percutaneous transhepatic biliary drainage. Cardiovasc Intervent Radiol. 2006;29(6):1015-21. doi: 10.1007/s00270-005-0273-z. PubMed PMID: 16988878.	Excluded from the final analysis	The article was excluded from the analysis due to the lack of relation between the title and the abstract of the article with the subject under study
679	Çakmak A, Genç V, Orozakunov E, Kepenekçi I, Çetinkaya Ö, Hazinedaroglu MS. Partial cholecystectomy is a safe and efficient method. Chirurgia. 2009;104(6):701-4. PubMed PMID: WOS:000272627900006.	Excluded from the final analysis	The article was excluded from the analysis due to the lack of relation between the title and the abstract of the article with the subject under study
680	Shibata A, Ogimoto I, Kurozawa Y, Nose T, Yoshimura T, Suzuki H, et al. Past medical history and risk of death due to hepatocellular carcinoma, univariate analysis of JACC study data. Kurume Med J. 2003;50(3-4):109-19. doi: 10.2739/kurumemedj.50.109. PubMed PMID: 14768473.	Excluded from the final analysis	The article was excluded from the analysis due to the lack of relation between the title and the abstract of the article with the subject under study
681	Banish LD, Gilmartin WG. PATHOLOGICAL FINDINGS IN THE HAWAIIAN MONK SEAL. Journal of Wildlife Diseases. 1992;28(3):428-34. doi: 10.7589/0090-3558-28.3.428. PubMed PMID: WOS:A1992JF48500013.	Excluded from the final analysis	The article was excluded from the analysis due to the lack of relation between the title and the abstract of the article with the subject under study

682	Lightner AM, Kirkwood KS. Pathophysiology of gallstone pancreatitis. Front Biosci. 2001;6:E66-76. Epub 20011001. doi: 10.2741/lightner. PubMed PMID: 11578966.	Excluded from the final analysis	The article was excluded from the analysis due to the lack of relation between the title and the abstract of the article with the subject under study
683	Lightner AM, Kirkwood KS. Pathophysiology of gallstone pancreatitis. Frontiers in Bioscience-Landmark. 2001;6:E66-E76. doi: 10.2741/Lightner. PubMed PMID: WOS:000171372600022.	Excluded from the final analysis	The article was excluded from the analysis due to the lack of relation between the title and the abstract of the article with the subject under study
684	Bawazir AO, Bawazir OA, Bawazir RO. A pediatric case of double gallbladder with gallstone. Saudi Medical Journal. 2021;42(4):441-4. doi: 10.15537/smj.2021.42.4.20200534. PubMed PMID: WOS:000637762100013.	Excluded from the final analysis	The article was excluded from the analysis due to the lack of relation between the title and the abstract of the article with the subject under study
685	Ko JS, Yi NJ, Suh KS, Seo JK. Pediatric liver transplantation for fibropolycystic liver disease. Pediatr Transplant. 2012;16(2):195-200. doi: 10.1111/j.1399-3046.2012.01661.x. PubMed PMID: 22360404.	Excluded from the final analysis	The article was excluded from the analysis due to the lack of relation between the title and the abstract of the article with the subject under study
686	Rosenberg JB, Hutcheson KA. Pediatric sickle cell retinopathy: correlation with clinical factors. J aapos. 2011;15(1):49-53. doi: 10.1016/j.jaapos.2010.11.014. PubMed PMID: 21397806.	Excluded from the final analysis	The article was excluded from the analysis due to the lack of relation between the title and the abstract of the article with the subject under study

687	Grønlykke L, Tarp B, Dutoit SH, Wilkens R. Peliosis hepatis: a complicating finding in a case of biliary colic. BMJ Case Rep. 2013;2013. Epub 20130926. doi: 10.1136/bcr-2013-200539. PubMed PMID: 24072833; PubMed Central PMCID: PMCPMC3794215.	Excluded from the final analysis	The article was excluded from the analysis due to the lack of relation between the title and the abstract of the article with the subject under study
688	Devkota HR, Bhandari B, Adhikary P. Perceived mental health, wellbeing and associated factors among Nepali male migrant and non-migrant workers: A qualitative study. J Migr Health. 2021;3:100013. Epub 20201204. doi: 10.1016/j.jmh.2020.100013. PubMed PMID: 34405181; PubMed Central PMCID: PMCPMC8352157.	Excluded from the final analysis	The article was excluded from the analysis due to the lack of relation between the title and the abstract of the article with the subject under study
689	Nikfarjam M, Shen L, Fink MA, Muralidharan V, Starkey G, Jones RM, Christophi C. Percutaneous cholecystostomy for treatment of acute cholecystitis in the era of early laparoscopic cholecystectomy. Surg Laparosc Endosc Percutan Tech. 2013;23(5):474-80. doi: 10.1097/SLE.0b013e318290142d. PubMed PMID: 24105289.	Excluded from the final analysis	The article was excluded from the analysis due to the lack of relation between the title and the abstract of the article with the subject under study
690	Nikfarjam M, Shen L, Fink MA, Muralidharan V, Starkey G, Jones RM, Christophi C. Percutaneous Cholecystostomy for Treatment of Acute Cholecystitis in the Era of Early Laparoscopic Cholecystectomy. Surgical Laparoscopy Endoscopy & Percutaneous Techniques. 2013;23(5):474-80. doi: 10.1097/SLE.0b013e318290142d. PubMed PMID: WOS:000330374600019.	Excluded from the final analysis	The article was excluded from the analysis due to the lack of relation between the title and the abstract of the article with the subject under study
691	vanSonnenberg E, D'Agostino HB, Goodacre BW, Sanchez RB, Casola G. Percutaneous gallbladder puncture and cholecystostomy: results, complications, and caveats for safety. Radiology. 1992;183(1):167-70. doi: 10.1148/radiology.183.1.1549666. PubMed PMID: 1549666.	Excluded from the final analysis	The article was excluded from the analysis due to the lack of relation between the title and the abstract of the article with the subject under study

692	Kim YW, Lee SM, Choi HC, Won JH, Na JB, Cho JM, et al. Percutaneous removal of common bile duct stones using a modified balloon technique. Medicine. 2021;100(14). doi: 10.1097/md.0000000000024486. PubMed PMID: WOS:000659026900005.	Excluded from the final analysis	The article was excluded from the analysis due to the lack of relation between the title and the abstract of the article with the subject under study
693	Sharpe JC, Marxer WL. Physical examination of well persons. what is "adequate" for detection of unsuspected disease? Calif Med. 1962;96(1):35-40. PubMed PMID: 13911411; PubMed Central PMCID: PMCPMC1574759.	Excluded from the final analysis	The article was excluded from the analysis due to the lack of relation between the title and the abstract of the article with the subject under study
694	Potty VH. Physio-chemical aspects, physiological functions, nutritional importance and technological significance of dietary fibres - A critical appraisal. Journal of Food Science and Technology-Mysore. 1996;33(1):1-18. PubMed PMID: WOS:A1996UV93800001.	Excluded from the final analysis	The article was excluded from the analysis due to the lack of relation between the title and the abstract of the article with the subject under study
695	Barragan C, Alshehri H, Marom G, Glazer Y, Swanstrom L, Shlomovitz E. A Pilot Study of Percutaneous Cholecystoenteric Anastomosis: A New Option for High-Risk Patients with Symptomatic Gallstones. Journal of Vascular and Interventional Radiology. 2024;35(1):74-9. doi: 10.1016/j.jvir.2023.09.025. PubMed PMID: WOS:001165897400001.	Excluded from the final analysis	The article was excluded from the analysis due to the lack of relation between the title and the abstract of the article with the subject under study
696	Castellani C, Quinzii C, Altieri S, Mastella G, Assael BM. A pilot survey of cystic fibrosis clinical manifestations in CFTR mutation heterozygotes. Genet Test. 2001;5(3):249-54. doi: 10.1089/10906570152742317. PubMed PMID: 11788092.	Excluded from the final analysis	The article was excluded from the analysis due to the lack of relation between the title and the abstract of the article with the subject under study

697	Essell JH, Thompson JM, Harman GS, Halvorson RD, Snyder MJ, Callander NS, Clement DJ. PILOT TRIAL OF PROPHYLACTIC URSODIOL TO DECREASE THE INCIDENCE OF VENOOCCLUSIVE DISEASE OF THE LIVER IN ALLOGENEIC BONE-MARROW TRANSPLANT PATIENTS. Bone Marrow Transplantation. 1992;10(4):367-72. PubMed PMID: WOS:A1992JT18100009.	Excluded from the final analysis	The article was excluded from the analysis due to the lack of relation between the title and the abstract of the article with the subject under study
698	Laakso M, Suhonen M, Julkunen R, Pyorala K. PLASMA-INSULIN, SERUM-LIPIDS AND LIPOPROTEINS IN GALL STONE DISEASE IN NON-INSULIN-DEPENDENT DIABETIC SUBJECTS - A CASE CONTROL STUDY. Gut. 1990;31(3):344-7. doi: 10.1136/gut.31.3.344. PubMed PMID: WOS:A1990CT53200024.	Excluded from the final analysis	The article was excluded from the analysis due to the lack of relation between the title and the abstract of the article with the subject under study
699	Canada R, Chaudry S, Gaber L, Waters B, Martinez A, Wall B. Polyarteritis nodosa and cryoglobulinemic glomerulonephritis related to chronic hepatitis C. Am J Med Sci. 2006;331(6):329-33. doi: 10.1097/00000441-200606000-00008. PubMed PMID: 16775442.	Excluded from the final analysis	The article was excluded from the analysis due to the lack of relation between the title and the abstract of the article with the subject under study
700	Hernández-Perera JC, Piñeiro-Pérez D, Martínez-Muñiz JO, Correa-Padilla JM, de Armas-Fernández MC, Jordán-González JA, et al. Polyserositis as a Post Covid-19 Complication. Medicc Review. 2022;24(3-4):57-60. doi: 10.37757/mr2022.V24.N3-4.9. PubMed PMID: WOS:000888814900012.	Excluded from the final analysis	The article was excluded from the analysis due to the lack of relation between the title and the abstract of the article with the subject under study
701	Wang SB, Li B, Ungvari GS, Ng CH, Chiu HFK, Kou CG, et al. Poor mental health status and its associations with demographic characteristics and chronic diseases in Chinese elderly. Social Psychiatry and Psychiatric Epidemiology. 2016;51(10):1449-55. doi: 10.1007/s00127-016-1271-y. PubMed PMID: WOS:000385164700010.	Excluded from the final analysis	The article was excluded from the analysis due to the lack of relation between the title and the abstract of the article with the subject under study

702	Choi SY, Lee HK, Yi BH, Lee MH, Lee JE, Min JH, et al. Pope's hat sign: another valuable CT finding of early acute cholecystitis. Abdom Radiol (NY). 2018;43(7):1693-702. doi: 10.1007/s00261-017-1421-z. PubMed PMID: 29198010.	Excluded from the final analysis	The article was excluded from the analysis due to the lack of relation between the title and the abstract of the article with the subject under study
703	Park JM, Park N, Lee SH, Han KD, Kang CD, Lee JM, et al. A population-based cohort study on risk factors for acute pancreatitis: A comparison by age group. Pancreatology. 2023;23(3):321-9. Epub 20230317. doi: 10.1016/j.pan.2023.03.004. PubMed PMID: 36964006.	Excluded from the final analysis	The article was excluded from the analysis due to the lack of relation between the title and the abstract of the article with the subject under study
704	Duseja A. Portal cavernoma cholangiopathy-clinical characteristics. J Clin Exp Hepatol. 2014;4(Suppl 1):S34-6. Epub 20131022. doi: 10.1016/j.jceh.2013.05.014. PubMed PMID: 25755593; PubMed Central PMCID: PMCPMC4244822.	Excluded from the final analysis	The article was excluded from the analysis due to the lack of relation between the title and the abstract of the article with the subject under study
705	Itare VB, Imanirad D, Almaghraby A. Portal Cholangiopathy: An Uncommon Cause of Right Upper Quadrant Pain. Cureus Journal of Medical Science. 2020;12(9). doi: 10.7759/cureus.10281. PubMed PMID: WOS:000566658800014.	Excluded from the final analysis	The article was excluded from the analysis due to the lack of relation between the title and the abstract of the article with the subject under study
706	Poo S, Pencavel TD, Jackson J, Jiao LR. Portal hypertension and chylous ascites complicating acute pancreatitis: the therapeutic value of portal vein stenting. Ann R Coll Surg Engl. 2018;100(1):e1-e3. Epub 20171019. doi: 10.1308/rcsann.2017.0078. PubMed PMID: 29046075; PubMed Central PMCID: PMCPMC5838657.	Excluded from the final analysis	The article was excluded from the analysis due to the lack of relation between the title and the abstract of the article with the subject under study

707	Suárez V, Puerta A, Santos LF, Pérez JM, Varón A, Botero RC. Portal hypertensive biliopathy: A single center experience and literature review. World J Hepatol. 2013;5(3):137-44. doi: 10.4254/wjh.v5.i3.137. PubMed PMID: 23556047; PubMed Central PMCID: PMCPMC3612573.	Excluded from the final analysis	The article was excluded from the analysis due to the lack of relation between the title and the abstract of the article with the subject under study
708	Cardoso R, Casela A, Lopes S, Agostinho C, Souto P, Camacho E, et al. Portal Hypertensive Biliopathy: An Infrequent Cause of Biliary Obstruction. GE Port J Gastroenterol. 2015;22(2):65-9. Epub 20150318. doi: 10.1016/j.jpge.2015.01.003. PubMed PMID: 28868376; PubMed Central PMCID: PMCPMC5579995.	Excluded from the final analysis	The article was excluded from the analysis due to the lack of relation between the title and the abstract of the article with the subject under study
709	Tajima T, Yoshimitsu K, Irie H, Aibe H, Shinozaki K, Nishie A, et al. Portal vein occlusion or stenosis in patients with hepatolithiasis: observation by multiphasic contrast-enhanced CT. Clinical Radiology. 2005;60(4):469-78. doi: 10.1016/j.crad.2004.09.008. PubMed PMID: WOS:000228259900007.	Excluded from the final analysis	The article was excluded from the analysis due to the lack of relation between the title and the abstract of the article with the subject under study
710	Amini A, Vaezi Z, Koury E, Zafar S, Chahla E. Portal Vein Thrombosis and Intra-Abdominal Hypertension Presenting as Complications of Hypertriglyceridemia-Induced Severe Acute Pancreatitis. Cureus. 2020;12(8):e9889. Epub 20200820. doi: 10.7759/cureus.9889. PubMed PMID: 32968555; PubMed Central PMCID: PMCPMC7502419.	Excluded from the final analysis	The article was excluded from the analysis due to the lack of relation between the title and the abstract of the article with the subject under study
711	Theodor E. PORTAL-HYPERTENSION COMPLICATING LIVER INVOLVEMENT IN METASTATIC CARCINOMA - CASE-REPORT. Israel Journal of Medical Sciences. 1979;15(3):285-7. PubMed PMID: WOS:A1979GN78300015.	Excluded from the final analysis	The article was excluded from the analysis due to the lack of relation between the title and the abstract of the article with the subject under study

712	Osborne DR. Porto-biliary fistula and portal hypertension due to gallstones. A case report. Acta Chir Scand. 1980;146(5):363-5. PubMed PMID: 7468067.	Excluded from the final analysis	The article was excluded from the analysis due to the lack of relation between the title and the abstract of the article with the subject under study
713	Sbeit W, Sbeit M, Kalisky I, Katz L, Mari A, Khoury T. The Possible Association of Non-Alcoholic Fatty Liver Disease with Acute Cholangitis: A Retrospective Multicenter Cohort Study. Life (Basel). 2021;12(1). Epub 20211227. doi: 10.3390/life12010035. PubMed PMID: 35054428; PubMed Central PMCID: PMCPMC8779558.	Excluded from the final analysis	The article was excluded from the analysis due to the lack of relation between the title and the abstract of the article with the subject under study
714	Sbeit W, Abukaes H, Ahmad HS, Sbeit M, Kalisky I, Katz L, et al. The possible association of proton pump inhibitor use with acute cholangitis in patients with choledocholithiasis: a multi-center study. Scandinavian Journal of Gastroenterology. 2023;58(1):83-7. doi: 10.1080/00365521.2022.2106150. PubMed PMID: WOS:000836574700001.	Excluded from the final analysis	The article was excluded from the analysis due to the lack of relation between the title and the abstract of the article with the subject under study
715	Sbeit W, Abukaes H, Said Ahmad H, Sbeit M, Kalisky I, Katz L, et al. The possible association of proton pump inhibitor use with acute cholangitis in patients with choledocholithiasis: a multi-center study. Scand J Gastroenterol. 2023;58(1):83-7. Epub 20220805. doi: 10.1080/00365521.2022.2106150. PubMed PMID: 35930433.	Excluded from the final analysis	The article was excluded from the analysis due to the lack of relation between the title and the abstract of the article with the subject under study
716	Hirano T, Manabe T. A possible mechanism for gallstone pancreatitis: repeated short-term pancreaticobiliary duct obstruction with exocrine stimulation in rats. Proc Soc Exp Biol Med. 1993;202(2):246-52. doi: 10.3181/00379727-202-43534. PubMed PMID: 7678705.	Excluded from the final analysis	The article was excluded from the analysis due to the lack of relation between the title and the abstract of the article with the subject under study

717	Shanti I, Samardali M, Bambhroliya Z, Alhusari L. Post-endoscopic Retrograde Cholangiopancreatography Hemorrhagic Pancreatitis in a Young Female: A Case Report. Cureus. 2024;16(5):e60929. Epub 20240523. doi: 10.7759/cureus.60929. PubMed PMID: 38910698; PubMed Central PMCID: PMCPMC11193661.	Excluded from the final analysis	The article was excluded from the analysis due to the lack of relation between the title and the abstract of the article with the subject under study
718	Kazi IA, Siddiqui MA, Thimmappa ND, Abdelaziz A, Gaballah AH, Davis R, et al. Post-operative complications of cholecystectomy: what the radiologist needs to know. Abdominal Radiology. 2024. doi: 10.1007/s00261-024-04387-5. PubMed PMID: WOS:001258630100004.	Excluded from the final analysis	The article was excluded from the analysis due to the lack of relation between the title and the abstract of the article with the subject under study
719	Garcea D, Martuzzi F, Santelmo N, Savoia M, Casertano MG, Furno A, Ruggeri V. Post-surgical deep vein thrombosis prevention: evaluation of the risk/benefit ratio of fractionated and unfractionated heparin. Curr Med Res Opin. 1992;12(9):572-83. doi: 10.1185/03007999209111524. PubMed PMID: 1316258.	Excluded from the final analysis	The article was excluded from the analysis due to the lack of relation between the title and the abstract of the article with the subject under study
720	Ravn SH, Rosenberg J, Bostofte E. POSTMENOPAUSAL HORMONE REPLACEMENT THERAPY - CLINICAL IMPLICATIONS. European Journal of Obstetrics & Gynecology and Reproductive Biology. 1994;53(2):81-93. doi: 10.1016/0028-2243(94)90213-5. PubMed PMID: WOS:A1994NB71600002.	Excluded from the final analysis	The article was excluded from the analysis due to the lack of relation between the title and the abstract of the article with the subject under study
721	Manson JE. POSTMENOPAUSAL HORMONE-THERAPY AND ATHEROSCLEROTIC DISEASE. American Heart Journal. 1994;128(6):1337-43. doi: 10.1016/0002-8703(94)90257-7. PubMed PMID: WOS:A1994PW31200008.	Excluded from the final analysis	The article was excluded from the analysis due to the lack of relation between the title and the abstract of the article with the subject under study

722	Amato A, Mauro M, Trimarchi A, Secondo P, Battaglia C, Griffanti Bartoli F. [Postoperative course after laparoscopic surgery of the upper abdomen]. Minerva Chir. 1994;49(7-8):619-27. PubMed PMID: 7991166.	Excluded from the final analysis	The article was excluded from the analysis due to the lack of relation between the title and the abstract of the article with the subject under study
723	Chetty R, Serra S. A pragmatic approach to vasculitis in the gastrointestinal tract. J Clin Pathol. 2017;70(6):470-5. Epub 20170124. doi: 10.1136/jclinpath-2016-204308. PubMed PMID: 28119348.	Excluded from the final analysis	The article was excluded from the analysis due to the lack of relation between the title and the abstract of the article with the subject under study
724	Shabanzadeh DM, Sorensen LT, Jorgensen T. A Prediction Rule for Risk Stratification of Incidentally Discovered Gallstones: Results From a Large Cohort Study. Gastroenterology. 2016;150(1):156-+. doi: 10.1053/j.gastro.2015.09.002. PubMed PMID: WOS:000366832800033.	Excluded from the final analysis	The article was excluded from the analysis due to the lack of relation between the title and the abstract of the article with the subject under study
725	Vaynshtein J, Sabbag G, Pinsk I, Rahmani I, Reshef A. Predictors for choledocholitiasis in patients undergoing endoscopic ultrasound. Scandinavian Journal of Gastroenterology. 2018;53(3):335-9. doi: 10.1080/00365521.2018.1435716. PubMed PMID: WOS:000426948600015.	Excluded from the final analysis	The article was excluded from the analysis due to the lack of relation between the title and the abstract of the article with the subject under study
726	Nogoy DM, Padmanaban V, Balazero LL, Rosado J, Sifri ZC. Predictors of Difficult Laparoscopic Cholecystectomy on Humanitarian Missions to Peru Difficult Laparoscopic Cholecystectomy in Surgical Missions. J Surg Res. 2021;267:102-8. Epub 20210620. doi: 10.1016/j.jss.2021.04.020. PubMed PMID: 34157489.	Excluded from the final analysis	The article was excluded from the analysis due to the lack of relation between the title and the abstract of the article with the subject under study

727	Qureshi I, Awad ZT. Predictors of failure of the laparoscopic approach for the management of small bowel obstruction. Am Surg. 2010;76(9):947-50. PubMed PMID: 20836340.	Excluded from the final analysis	The article was excluded from the analysis due to the lack of relation between the title and the abstract of the article with the subject under study
728	Liu JK, Braschi C, de Virgilio C, Ozao-Choy J, Kim DY, Moazzez A. Predictors of poor outcomes after cholecystectomy in gallstone pancreatitis: NSQIP analysis of 30-day morbidity and mortality. Langenbecks Arch Surg. 2022;408(1):5. Epub 20221231. doi: 10.1007/s00423-022-02731-1. PubMed PMID: 36585495.	Excluded from the final analysis	The article was excluded from the analysis due to the lack of relation between the title and the abstract of the article with the subject under study
729	Liu JK, Braschi C, de Virgilio C, Ozao-Choy J, Kim DY, Moazzez A. Predictors of poor outcomes after cholecystectomy in gallstone pancreatitis: NSQIP analysis of 30-day morbidity and mortality. Langenbecks Archives of Surgery. 2022;408(1). doi: 10.1007/s00423-022-02731-1. PubMed PMID: WOS:000915687800001.	Excluded from the final analysis	The article was excluded from the analysis due to the lack of relation between the title and the abstract of the article with the subject under study
730	Huang JL, Chen WK, Lin CL, Kao CH, Shih HM. Preeclampsia and the Risk of Pancreatitis: A Nationwide, Population-Based Cohort Study. Gastroenterol Res Pract. 2020;2020:3261542. Epub 20201230. doi: 10.1155/2020/3261542. PubMed PMID: 33456459; PubMed Central PMCID: PMCPMC7787823.	Excluded from the final analysis	The article was excluded from the analysis due to the lack of relation between the title and the abstract of the article with the subject under study
731	Mishra N. Pregnancy With Diabetic Ketoacidosis and Hypertriglyceridemia-Induced Acute Pancreatitis: The Enigmatic Triad. Cureus. 2023;15(12):e50862. Epub 20231220. doi: 10.7759/cureus.50862. PubMed PMID: 38249199; PubMed Central PMCID: PMCPMC10798904.	Excluded from the final analysis	The article was excluded from the analysis due to the lack of relation between the title and the abstract of the article with the subject under study

732	Bahloul M, Ayedi M, Dammak H, Trabelsi K, Bouaziz M. [Pregnancy-induced hypertension complicated by acute pancreatitis]. Ann Fr Anesth Reanim. 2004;23(2):157-9. doi: 10.1016/j.annfar.2003.11.017. PubMed PMID: 15030867.	Excluded from the final analysis	The article was excluded from the analysis due to the lack of relation between the title and the abstract of the article with the subject under study
733	Marcovici I, Marzano D. Pregnancy-induced hypertension complicated by postpartum renal failure and pancreatitis: a case report. Am J Perinatol. 2002;19(4):177-9. doi: 10.1055/s-2002-28500. PubMed PMID: 12012278.	Excluded from the final analysis	The article was excluded from the analysis due to the lack of relation between the title and the abstract of the article with the subject under study
734	Mazza GR, Youssefzadeh AC, Aberle LS, Anderson ZS, Mandelbaum RS, Ouzounian JG, et al. Pregnant patients undergoing cholecystectomy: nationwide assessment of clinical characteristics and outcomes. AJOG Glob Rep. 2024;4(1):100310. Epub 20240111. doi: 10.1016/j.xagr.2024.100310. PubMed PMID: 38304305; PubMed Central PMCID: PMCPMC10830852.	Excluded from the final analysis	The article was excluded from the analysis due to the lack of relation between the title and the abstract of the article with the subject under study
735	Magnano San Lio R, Barchitta M, Maugeri A, Quartarone S, Basile G, Agodi A. Preoperative Risk Factors for Conversion from Laparoscopic to Open Cholecystectomy: A Systematic Review and Meta-Analysis. Int J Environ Res Public Health. 2022;20(1). Epub 20221227. doi: 10.3390/ijerph20010408. PubMed PMID: 36612732; PubMed Central PMCID: PMCPMC9819914.	Excluded from the final analysis	The article was excluded from the analysis due to the lack of relation between the title and the abstract of the article with the subject under study
736	Wirth J, di Giuseppe R, Wientzek A, Katzke VA, Kloss M, Kaaks R, et al. Presence of gallstones and the risk of cardiovascular diseases: the EPIC-Germany cohort study. European Journal of Preventive Cardiology. 2015;22(3):326-34. doi: 10.1177/2047487313512218. PubMed PMID: WOS:000349291000008.	Excluded from the final analysis	The article was excluded from the analysis due to the lack of relation between the title and the abstract of the article with the subject under study

737	Keltikangas-Järvinen L. The prevalence and construct validity of type A behaviour in patients with duodenal ulcers. Br J Med Psychol. 1987;60 (Pt 2):163-7. doi: 10.1111/j.2044-8341.1987.tb02727.x. PubMed PMID: 3620393.	Excluded from the final analysis	The article was excluded from the analysis due to the lack of relation between the title and the abstract of the article with the subject under study
738	Mahfouz MEM, Altowairqi ADM, Alghamdi HY, Alzahrani MSZ, Alqurashi AK, Alhuraity TH, Alqurashi AS. Prevalence and Factors Associated With Post-Cholecystectomy Syndrome in Saudi Arabia. Cureus. 2022;14(12):e32827. Epub 20221222. doi: 10.7759/cureus.32827. PubMed PMID: 36694499; PubMed Central PMCID: PMCPMC9864481.	Excluded from the final analysis	The article was excluded from the analysis due to the lack of relation between the title and the abstract of the article with the subject under study
739	Liao XH, Cao X, Liu J, Xie XH, Sun YH, Zhong BH. Prevalence and features of fatty liver detected by physical examination in Guangzhou. World J Gastroenterol. 2013;19(32):5334-9. doi: 10.3748/wjg.v19.i32.5334. PubMed PMID: 23983438; PubMed Central PMCID: PMCPMC3752569.	Excluded from the final analysis	The article was excluded from the analysis due to the lack of relation between the title and the abstract of the article with the subject under study
740	Shiina Y, Toyoda T, Kawasoe Y, Tateno S, Shirai T, Matsuo K, et al. The prevalence and risk factors for cholelithiasis and asymptomatic gallstones in adults with congenital heart disease. International Journal of Cardiology. 2011;152(2):171-6. doi: 10.1016/j.ijcard.2010.07.011. PubMed PMID: WOS:000296540800011.	Excluded from the final analysis	The article was excluded from the analysis due to the lack of relation between the title and the abstract of the article with the subject under study
741	Song ST, Shi J, Wang XH, Guo YB, Hu PF, Zhu F, et al. Prevalence and risk factors for gallstone disease: A population-based cross-sectional study. J Dig Dis. 2020;21(4):237-45. Epub 20200415. doi: 10.1111/1751-2980.12857. PubMed PMID: 32166900.	Excluded from the final analysis	The article was excluded from the analysis due to the lack of relation between the title and the abstract of the article with the subject under study

742	Zamani F, Sohrabi M, Alipour A, Motamed N, Saeedian FS, Pirzad R, et al. Prevalence and risk factors of cholelithiasis in Amol city, northern Iran: a population based study. Arch Iran Med. 2014;17(11):750-4. PubMed PMID: 25365614.	Excluded from the final analysis	The article was excluded from the analysis due to the lack of relation between the title and the abstract of the article with the subject under study
743	Zhu L, Aili A, Zhang C, Saiding A, Abudureyimu K. Prevalence of and risk factors for gallstones in Uighur and Han Chinese. World J Gastroenterol. 2014;20(40):14942-9. doi: 10.3748/wjg.v20.i40.14942. PubMed PMID: 25356055; PubMed Central PMCID: PMCPMC4209558.	Excluded from the final analysis	The article was excluded from the analysis due to the lack of relation between the title and the abstract of the article with the subject under study
744	Hopper KD, Landis JR, Meilstrup JW, McCauslin MA, Sechtin AG. THE PREVALENCE OF ASYMPTOMATIC GALLSTONES IN THE GENERAL-POPULATION. Investigative Radiology. 1991;26(11):939-45. doi: 10.1097/00004424-199111000-00004. PubMed PMID: WOS:A1991GP56100004.	Excluded from the final analysis	The article was excluded from the analysis due to the lack of relation between the title and the abstract of the article with the subject under study
745	Millán T, Freitte A, Masalleras C, Porte L, Vargas C. [Prevalence of chronic diseases in young postpartum women]. Rev Med Chil. 1992;120(2):210-4. PubMed PMID: 1340563.	Excluded from the final analysis	The article was excluded from the analysis due to the lack of relation between the title and the abstract of the article with the subject under study
746	Diehl AK, Stern MP, Ostrower VS, Friedman PC. Prevalence of clinical gallbladder disease in Mexican-American, Anglo, and black women. South Med J. 1980;73(4):438-41, 43. doi: 10.1097/00007611-198004000-00012. PubMed PMID: 7367932.	Excluded from the final analysis	The article was excluded from the analysis due to the lack of relation between the title and the abstract of the article with the subject under study

747	Fujita T, Hayashi K, Katanoda K, Matsumura Y, Lee JS, Takagi H, et al. Prevalence of diseases and statistical power of the Japan Nurses' Health Study. Ind Health. 2007;45(5):687-94. doi: 10.2486/indhealth.45.687. PubMed PMID: 18057812.	Excluded from the final analysis	The article was excluded from the analysis due to the lack of relation between the title and the abstract of the article with the subject under study
748	Khomusi MM, Parveen S, Iqbal M, Ahmed T, Husain R, Seth US, et al. Prevalence of Empyema or Mucocele or Other Histological Diagnoses in Patients Undergoing Cholecystectomy With Diagnosis of Chronic Cholecystitis. Cureus Journal of Medical Science. 2022;14(4). doi: 10.7759/cureus.23773. PubMed PMID: WOS:000782267200008.	Excluded from the final analysis	The article was excluded from the analysis due to the lack of relation between the title and the abstract of the article with the subject under study
749	Sinha SR, Prakash P. Prevalence of Thyroid Disorder in Gallstone Disease Patients: A Cross-Sectional Study. Cureus. 2024;16(1):e52422. Epub 20240117. doi: 10.7759/cureus.52422. PubMed PMID: 38371154; PubMed Central PMCID: PMCPMC10870244.	Excluded from the final analysis	The article was excluded from the analysis due to the lack of relation between the title and the abstract of the article with the subject under study
750	Kelly DA. Preventing parenteral nutrition liver disease. Early Hum Dev. 2010;86(11):683-7. doi: 10.1016/j.earlhumdev.2010.08.012. PubMed PMID: 20923719.	Excluded from the final analysis	The article was excluded from the analysis due to the lack of relation between the title and the abstract of the article with the subject under study
751	Ai T, Azemoto R, Saisho H. Prevention of gallstones by ursodeoxycholic acid after cardiac surgery. Journal of Gastroenterology. 2003;38(11):1071-6. doi: 10.1007/s00535-003-1198-4. PubMed PMID: WOS:000187004800008.	Excluded from the final analysis	The article was excluded from the analysis due to the lack of relation between the title and the abstract of the article with the subject under study

752	Sasmal PK, Tantia O, Jain M, Khanna S, Sen B. Primary access-related complications in laparoscopic cholecystectomy via the closed technique: experience of a single surgical team over more than 15 years. Surgical Endoscopy and Other Interventional Techniques. 2009;23(11):2407-15. doi: 10.1007/s00464-009-0437-z. PubMed PMID: WOS:000271430100002.	Excluded from the final analysis	The article was excluded from the analysis due to the lack of relation between the title and the abstract of the article with the subject under study
753	Wheeler WE, Hanks J, Raman VK. PRIMARY AORTOENTERIC FISTULAS. American Surgeon. 1992;58(1):53-4. PubMed PMID: WOS:A1992GY74800009.	Excluded from the final analysis	The article was excluded from the analysis due to the lack of relation between the title and the abstract of the article with the subject under study
754	Bhatia P, John S, Kalhan S, Khetan M. Primary laparoscopic cholecystectomy in patients with portal cavernoma and non-obstructive portal biliopathy: Two case reports. J Minim Access Surg. 2014;10(3):161-2. doi: 10.4103/0972-9941.134885. PubMed PMID: 25013336; PubMed Central PMCID: PMCPMC4083552.	Excluded from the final analysis	The article was excluded from the analysis due to the lack of relation between the title and the abstract of the article with the subject under study
755	Oudhoff JP, Timmermans DR, Knol DL, Bijnen AB, Van der Wal G. Prioritising patients on surgical waiting lists: a conjoint analysis study on the priority judgements of patients, surgeons, occupational physicians, and general practitioners. Soc Sci Med. 2007;64(9):1863-75. Epub 20070226. doi: 10.1016/j.socscimed.2007.01.002. PubMed PMID: 17324491.	Excluded from the final analysis	The article was excluded from the analysis due to the lack of relation between the title and the abstract of the article with the subject under study
756	Lv TR, Wang JK, Li FY, Hu HJ. Prognostic factors for resected cases with gallbladder carcinoma: a systematic review and meta-analysis. International Journal of Surgery. 2024;110(7):4342-55. doi: 10.1097/js9.0000000000001403. PubMed PMID: WOS:001270594800005.	Excluded from the final analysis	The article was excluded from the analysis due to the lack of relation between the title and the abstract of the article with the subject under study

757	Ekwattanakit S, Siritanaratkul N, Viprakasit V. A prospective analysis for prevalence of complications in Thai nontransfusion-dependent Hb E/β-thalassemia and α-thalassemia (Hb H disease). Am J Hematol. 2018;93(5):623-9. Epub 20180214. doi: 10.1002/ajh.25046. PubMed PMID: 29359464.	Excluded from the final analysis	The article was excluded from the analysis due to the lack of relation between the title and the abstract of the article with the subject under study
758	Joshipura VP, Haribhakti SP, Patel NR, Naik RP, Soni HN, Patel B, et al. A Prospective Randomized, Controlled Study Comparing Low Pressure Versus High Pressure Pneumoperitoneum During Laparoscopic Cholecystectomy. Surgical Laparoscopy Endoscopy & Percutaneous Techniques. 2009;19(3):234-40. doi: 10.1097/SLE.0b013e3181a97012. PubMed PMID: WOS:000267382100014.	Excluded from the final analysis	The article was excluded from the analysis due to the lack of relation between the title and the abstract of the article with the subject under study
759	Ahmed O, Asghar MS, Khurshaidi MN, Yasmin F, Kanwal N, Khokher AJ, et al. Provision of Surgical Services to COVID-19-Infected Patients at a Tertiary Care Center in Pakistan: A One-Year Clinical Review of the Year 2020 in General Surgery Department. Cureus. 2021;13(1):e12705. Epub 20210114. doi: 10.7759/cureus.12705. PubMed PMID: 33614312; PubMed Central PMCID: PMCPMC7883566.	Excluded from the final analysis	The article was excluded from the analysis due to the lack of relation between the title and the abstract of the article with the subject under study
760	Keltikangas-Järvinen L. 'Psychosomatic personality'--a personality constellation or an illness-related reaction? Br J Med Psychol. 1989;62 (Pt 4):325-31. doi: 10.1111/j.2044-8341.1989.tb02842.x. PubMed PMID: 2597648.	Excluded from the final analysis	The article was excluded from the analysis due to the lack of relation between the title and the abstract of the article with the subject under study
761	Smedema JP, Louw VJ. Pulmonary arterial hypertension after splenectomy for hereditary spherocytosis. Cardiovasc J Afr. 2007;18(2):84-9. PubMed PMID: 17497044.	Excluded from the final analysis	The article was excluded from the analysis due to the lack of relation between the title and the abstract of the article with the subject under study

762	Shelat VG, Chia CLK, Yeo CSW, Qiao W, Woon W, Junnarkar SP. Pyogenic Liver Abscess: Does <i>Escherichia Coli</i> Cause more Adverse Outcomes than <i>Klebsiella Pneumoniae</i>? World Journal of Surgery. 2015;39(10):2535-42. doi: 10.1007/s00268-015-3126-1. PubMed PMID: WOS:000362324100021.	Excluded from the final analysis	The article was excluded from the analysis due to the lack of relation between the title and the abstract of the article with the subject under study
763	Schlienger JL. Radical complications of obesity. Presse Medicale. 2010;39(9):913-20. doi: 10.1016/j.lpm.2010.04.018. PubMed PMID: WOS:000283107800011.	Excluded from the final analysis	The article was excluded from the analysis due to the lack of relation between the title and the abstract of the article with the subject under study
764	Launay D, Hatron PY, Queyrel V, Leteurtre E, Beaulieu S, Lemann M, et al. A rare association: peliosis hepatis and Crohn's disease. Revue De Medecine Interne. 2002;23(2):198-202. doi: 10.1016/s0248-8663(01)00537-9. PubMed PMID: WOS:000174096500013.	Excluded from the final analysis	The article was excluded from the analysis due to the lack of relation between the title and the abstract of the article with the subject under study
765	Wong V, Ali H, Amer K, Ahlawat S. A Rare Case of a Sickle Cell Patient With Post Endoscopic Retrograde Cholangiopancreatography (ERCP) Pancreatitis and Pseudoaneurysm Formation: An Association Worth Exploring. Cureus Journal of Medical Science. 2022;14(1). doi: 10.7759/cureus.21780. PubMed PMID: WOS:000762700500012.	Excluded from the final analysis	The article was excluded from the analysis due to the lack of relation between the title and the abstract of the article with the subject under study
766	Park S, Balasooriya J, Ncube T. A Rare Case of Gallstone Ileus: Bouveret Syndrome Presenting with Concurrent Gallstone Coleus. Case Rep Surg. 2020;2020:8844199. Epub 20201107. doi: 10.1155/2020/8844199. PubMed PMID: 33204566; PubMed Central PMCID: PMCPMC7666622.	Excluded from the final analysis	The article was excluded from the analysis due to the lack of relation between the title and the abstract of the article with the subject under study

767	Tsomidis I, Leonidou K, Papachristodoulou A, Rafailidis P, Prassopoulos P. A rare cause of acute pancreatitis: ischemia caused by free-floating intraluminal aortic thrombus. Hippokratia. 2021;25(3):138-40. PubMed PMID: WOS:000921586900007.	Excluded from the final analysis	The article was excluded from the analysis due to the lack of relation between the title and the abstract of the article with the subject under study
768	Tsomidis I, Leonidou K, Papachristodoulou A, Rafailidis V, Prassopoulos P. A rare cause of acute pancreatitis: ischemia caused by free-floating intraluminal aortic thrombus. Hippokratia. 2021;25(3):138-40. PubMed PMID: 36683902; PubMed Central PMCID: PMCPMC9851138.	Excluded from the final analysis	The article was excluded from the analysis due to the lack of relation between the title and the abstract of the article with the subject under study
769	Hailu S, Gebremariam M, Girma T, Regasa T. Re-operation for symptomatic cholelithiasis with a double gallbladder: A rare case report in a limited imaging setup. International Journal of Surgery Open. 2021;36. doi: 10.1016/j.ijso.2021.100416. PubMed PMID: WOS:000704861900001.	Excluded from the final analysis	The article was excluded from the analysis due to the lack of relation between the title and the abstract of the article with the subject under study
770	Leppäniemi A, Kemppainen E. Recent advances in the surgical management of necrotizing pancreatitis. Curr Opin Crit Care. 2005;11(4):349-52. doi: 10.1097/01.ccx.0000166398.50517.fb. PubMed PMID: 16015114.	Excluded from the final analysis	The article was excluded from the analysis due to the lack of relation between the title and the abstract of the article with the subject under study
771	Grundy SM. RECENT NUTRITION RESEARCH - IMPLICATIONS FOR FOODS OF THE FUTURE. Annals of Medicine. 1991;23(2):187-93. doi: 10.3109/07853899109148046. PubMed PMID: WOS:A1991FR60500018.	Excluded from the final analysis	The article was excluded from the analysis due to the lack of relation between the title and the abstract of the article with the subject under study

772	Grundy SM. Recent nutrition research: implications for foods of the future. Ann Med. 1991;23(2):187-93. doi: 10.3109/07853899109148046. PubMed PMID: 2069795.	Excluded from the final analysis	The article was excluded from the analysis due to the lack of relation between the title and the abstract of the article with the subject under study
773	Fan Z, Pan JY, Zhang YW. Recovery from a biliary stricture of a common bile duct ligature injury: A case report. World J Clin Cases. 2020;8(16):3567-72. doi: 10.12998/wjcc.v8.i16.3567. PubMed PMID: 32913865; PubMed Central PMCID: PMCPMC7457115.	Excluded from the final analysis	The article was excluded from the analysis due to the lack of relation between the title and the abstract of the article with the subject under study
774	Fan Z, Pan JY, Zhang YW. Recovery from a biliary stricture of a common bile duct ligature injury: A case report. World Journal of Clinical Cases. 2020;8(16):3567-72. doi: 10.12998/wjcc.v8.i16.3567. PubMed PMID: WOS:000568923200021.	Excluded from the final analysis	The article was excluded from the analysis due to the lack of relation between the title and the abstract of the article with the subject under study
775	Somogyi L, Martin SP, Ulrich CD. Recurrent Acute Pancreatitis. Curr Treat Options Gastroenterol. 2001;4(5):361-8. doi: 10.1007/s11938-001-0001-y. PubMed PMID: 11560783.	Excluded from the final analysis	The article was excluded from the analysis due to the lack of relation between the title and the abstract of the article with the subject under study
776	Jain PA, Gandhi VV, Desai P, Doctor NH. Recurrent cholangitis in the tropics: Worm or cast? Journal of Postgraduate Medicine. 2010;56(4):287-9. doi: 10.4103/0022-3859.70942. PubMed PMID: WOS:000288713200011.	Excluded from the final analysis	The article was excluded from the analysis due to the lack of relation between the title and the abstract of the article with the subject under study

777	Tian W, Yao QR, Duan ZY, Li CL, Huang DY. Recurrent holangitis caused by common bile duct compression due to hepatic artery variation. International Journal of Clinical and Experimental Medicine. 2016;9(2):4855-7. PubMed PMID: WOS:000374655200554.	Excluded from the final analysis	The article was excluded from the analysis due to the lack of relation between the title and the abstract of the article with the subject under study
778	El Kininy W, Kearney L, Hosam N, Broe P, Keeling A. Recurrent variceal haemorrhage managed with splenic vein stenting. Ir J Med Sci. 2017;186(2):323-7. Epub 20160225. doi: 10.1007/s11845-016-1420-z. PubMed PMID: 26911860.	Excluded from the final analysis	The article was excluded from the analysis due to the lack of relation between the title and the abstract of the article with the subject under study
779	Hanefeld M, Hora C, Schulze J, Rothe G, Barthel U, Haller H. REDUCED INCIDENCE OF CARDIOVASCULAR COMPLICATIONS AND MORTALITY IN HYPERLIPOPROTEINEMIA (HLP) WITH EFFECTIVE LIPID CORRECTION - THE DRESDEN HLP STUDY. Atherosclerosis. 1984;53(1):47-58. doi: 10.1016/0021-9150(84)90104-7. PubMed PMID: WOS:A1984TM58600006.	Excluded from the final analysis	The article was excluded from the analysis due to the lack of relation between the title and the abstract of the article with the subject under study
780	Hanefeld M, Hora C, Schulze J, Rothe G, Barthel U, Haller H. Reduced incidence of cardiovascular complications and mortality in hyperlipoproteinemia (HLP) with effective lipid correction. The Dresden HLP study. Atherosclerosis. 1984;53(1):47-58. doi: 10.1016/0021-9150(84)90104-7. PubMed PMID: 6497944.	Excluded from the final analysis	The article was excluded from the analysis due to the lack of relation between the title and the abstract of the article with the subject under study
781	Wei CY, Chuang SH, Lin CL, Kung WM, Tai HC, Tsai KWK, et al. Reduced risk of stroke following cholecystectomy: A nationwide population-based study. Journal of Gastroenterology and Hepatology. 2019;34(11):1992-8. doi: 10.1111/jgh.14678. PubMed PMID: WOS:000499030500019.	Excluded from the final analysis	The article was excluded from the analysis due to the lack of relation between the title and the abstract of the article with the subject under study

782	Targonski P, Jacobsen SJ, Weston SA, Leibson CL, Pfeifer E, Nemetz P, Roger VL. Referral to autopsy: Effect of antemortem cardiovascular disease - A population-based study in Olmsted County, Minnesota. Annals of Epidemiology. 2001;11(4):264-70. doi: 10.1016/s1047-2797(00)00220-9. PubMed PMID: WOS:000168107100007.	Excluded from the final analysis	The article was excluded from the analysis due to the lack of relation between the title and the abstract of the article with the subject under study
783	Hung MC, Chen CF, Tsou MT, Lin HH, Hwang LC, Hsu CP. Relationship Between Gallstone Disease and Cardiometabolic Risk Factors in Elderly People with Non-Alcoholic Fatty Liver Disease. Diabetes Metab Syndr Obes. 2020;13:3579-85. Epub 20201009. doi: 10.2147/dmso.S266947. PubMed PMID: 33116709; PubMed Central PMCID: PMCPMC7553650.	Excluded from the final analysis	The article was excluded from the analysis due to the lack of relation between the title and the abstract of the article with the subject under study
784	Liao Q, Chen Y, Peng Q, Li C. Relationship between triglyceride-glucose index and gallstones risk: a population-based study. Front Endocrinol (Lausanne). 2024;15:1420999. Epub 20240711. doi: 10.3389/fendo.2024.1420999. PubMed PMID: 39055055; PubMed Central PMCID: PMCPMC11269096.	Excluded from the final analysis	The article was excluded from the analysis due to the lack of relation between the title and the abstract of the article with the subject under study
785	Wang JJ, Li H, Hu JC, Shi RZ, Qin C, Chen X, et al. Relationship of triglyceride-glucose index to gallstone prevalence and age at first gallstone surgery in American adults. Scientific Reports. 2024;14(1). doi: 10.1038/s41598-024-67883-0. PubMed PMID: WOS:001274055500001.	Excluded from the final analysis	The article was excluded from the analysis due to the lack of relation between the title and the abstract of the article with the subject under study
786	Çelik A, Kut A, İlhan B. A report of a case: unusual portosystemic shunt in a hernia sac who has portal hypertension due to cirrhosis. Surg Radiol Anat. 2021;43(3):385-8. Epub 20200923. doi: 10.1007/s00276-020-02568-7. PubMed PMID: 32965518.	Excluded from the final analysis	The article was excluded from the analysis due to the lack of relation between the title and the abstract of the article with the subject under study

787	Sayenko VF, Lavryk AS, Stetsenko OP. Report on bariatric surgery in the Ukraine. Obes Surg. 2000;10(1):54-7. doi: 10.1381/09608920060674120. PubMed PMID: 10715646.	Excluded from the final analysis	The article was excluded from the analysis due to the lack of relation between the title and the abstract of the article with the subject under study
788	Ni HY, Zhang ZH, Fu HZ. [Research and development of Fructus Gardeniae]. Zhongguo Zhong Yao Za Zhi. 2006;31(7):538-41. PubMed PMID: 16780151.	Excluded from the final analysis	The article was excluded from the analysis due to the lack of relation between the title and the abstract of the article with the subject under study
789	Trovato GM, Catalano D, Sciacchitano G, Zuccalà G, Iannetti E. Resistive index of renal artery and blood pressure in postmenopausal women. Maturitas. 2002;41(3):223-30. doi: 10.1016/s0378-5122(01)00290-0. PubMed PMID: 11886768.	Excluded from the final analysis	The article was excluded from the analysis due to the lack of relation between the title and the abstract of the article with the subject under study
790	Trovato GM, Catalano D, Sciacchitano G, Zuccalà G, Iannetti E. Resistive index of renal artery and blood pressure in postmenopausal women. Maturitas. 2002;41(3):223-30. doi: 10.1016/s0378-5122(01)00290-0. PubMed PMID: WOS:000176347000008.	Excluded from the final analysis	The article was excluded from the analysis due to the lack of relation between the title and the abstract of the article with the subject under study
791	Li P, He S, Wu Y, Pang Y, Yang L, Shi J, et al. Retroperitoneal infantile hemangioma: a case report and literature review. Discov Oncol. 2024;15(1):373. Epub 20240827. doi: 10.1007/s12672-024-01260-1. PubMed PMID: 39190162; PubMed Central PMCID: PMCPMC11349721.	Excluded from the final analysis	The article was excluded from the analysis due to the lack of relation between the title and the abstract of the article with the subject under study

792	Mutiri B, Etonyeaku AC, Aloufi M, Alsaud JS. A Review of the Management of Cholelithiasis at Buraydah Central Hospital in the Qassim Region, Saudi Arabia: A Cross-Sectional Study. Cureus. 2023;15(12):e50846. Epub 20231220. doi: 10.7759/cureus.50846. PubMed PMID: 38249213; PubMed Central PMCID: PMCPMC10798649.	Excluded from the final analysis	The article was excluded from the analysis due to the lack of relation between the title and the abstract of the article with the subject under study
793	Alicioglu B. Right Liver Lobe Hypoplasia and Related Abnormalities. Pol J Radiol. 2015;80:503-5. Epub 20151113. doi: 10.12659/pjr.894658. PubMed PMID: 26634012; PubMed Central PMCID: PMCPMC4648108.	Excluded from the final analysis	The article was excluded from the analysis due to the lack of relation between the title and the abstract of the article with the subject under study
794	Vasiliadis K, Engelmann G, Sauer P, Weitz J, Schmidt J. Right porto-ovarian H-shunt for the surgical treatment of symptomatic portal biliopathy: a case report and literature review. HPB Surg. 2009;2009:152195. Epub 20090625. doi: 10.1155/2009/152195. PubMed PMID: 19584934; PubMed Central PMCID: PMCPMC2703746.	Excluded from the final analysis	The article was excluded from the analysis due to the lack of relation between the title and the abstract of the article with the subject under study
795	Cho JY, Han HS, Yoon YS, Ahn KS. Risk Factors for Acute Cholecystitis and a Complicated Clinical Course in Patients With Symptomatic Cholelithiasis. Archives of Surgery. 2010;145(4):329-33. doi: 10.1001/archsurg.2010.35. PubMed PMID: WOS:000295585200004.	Excluded from the final analysis	The article was excluded from the analysis due to the lack of relation between the title and the abstract of the article with the subject under study
796	Mishra K, Behari A, Shukla P, Tsuchiya Y, Endoh K, Asai T, et al. Risk factors for gallbladder cancer development in northern India: A gallstones-matched, case-control study. Indian J Med Res. 2021;154(5):699-706. doi: 10.4103/ijmr.IJMR_201_19. PubMed PMID: 35532588; PubMed Central PMCID: PMCPMC9210525.	Excluded from the final analysis	The article was excluded from the analysis due to the lack of relation between the title and the abstract of the article with the subject under study

797	Higashizono K, Nakatani E, Hawke P, Fujimoto S, Oba N. Risk factors for gallstone disease onset in Japan: Findings from the Shizuoka Study, a population-based cohort study. PLoS One. 2022;17(12):e0274659. Epub 20221230. doi: 10.1371/journal.pone.0274659. PubMed PMID: 36584097; PubMed Central PMCID: PMCPMC9803237.	Excluded from the final analysis	The article was excluded from the analysis due to the lack of relation between the title and the abstract of the article with the subject under study
798	Cui Y, Li Z, Zhao E, Cui N. Risk factors in patients with hereditary gallstones in Chinese pedigrees. Med Princ Pract. 2012;21(5):467-71. Epub 20120331. doi: 10.1159/000337437. PubMed PMID: 22473058.	Excluded from the final analysis	The article was excluded from the analysis due to the lack of relation between the title and the abstract of the article with the subject under study
799	Qin J, Han TQ, Fei J, Jiang ZY, Zhang Y, Yang SY, et al. [Risk factors of familial gallstone disease: study of 135 pedigrees]. Zhonghua Yi Xue Za Zhi. 2005;85(28):1966-9. PubMed PMID: 16313772.	Excluded from the final analysis	The article was excluded from the analysis due to the lack of relation between the title and the abstract of the article with the subject under study
800	Cao Z, Wei J, Zhang N, Liu W, Hong T, He X, Qu Q. Risk factors of systematic biliary complications in patients with gallbladder stones. Ir J Med Sci. 2020;189(3):943-7. Epub 20191219. doi: 10.1007/s11845-019-02161-x. PubMed PMID: 31858450.	Excluded from the final analysis	The article was excluded from the analysis due to the lack of relation between the title and the abstract of the article with the subject under study
801	Chen CH, Lin CL, Kao CH. Risk of aortic dissection or aneurysm in patients with gallstone disease: a retrospective cohort study in Taiwan. Bmj Open. 2021;11(8). doi: 10.1136/bmjopen-2021-049316. PubMed PMID: WOS:000700920300011.	Excluded from the final analysis	The article was excluded from the analysis due to the lack of relation between the title and the abstract of the article with the subject under study

802	Chen CH, Lin CL, Kao CH. The risk of coronary heart disease after diagnosis of gallbladder polyp: a retrospective nationwide population-based cohort study. Ann Transl Med. 2019;7(23):753. doi: 10.21037/atm.2019.11.114. PubMed PMID: 32042769; PubMed Central PMCID: PMCPMC6990032.	Excluded from the final analysis	The article was excluded from the analysis due to the lack of relation between the title and the abstract of the article with the subject under study
803	Chen CH, Lin CL, Kao CH. The risk of coronary heart disease after diagnosis of gallbladder polyp: a retrospective nationwide population-based cohort study. Annals of Translational Medicine. 2019;7(23). doi: 10.21037/atm.2019.11.114. PubMed PMID: WOS:000503257700042.	Excluded from the final analysis	The article was excluded from the analysis due to the lack of relation between the title and the abstract of the article with the subject under study
804	Chen CH, Lin CL, Hsu CY, Kao CH. Risk of gallstones in patients with obstructive sleep apnea: a nationwide observational cohort study. Sleep Breath. 2019;23(1):355-62. Epub 20180707. doi: 10.1007/s11325-018-1696-5. PubMed PMID: 29980983.	Excluded from the final analysis	The article was excluded from the analysis due to the lack of relation between the title and the abstract of the article with the subject under study
805	Kim YJ, Park YS, Shin CM, Han K, Park SH, Yoon H, et al. Risk of Heart Disease after Cholecystectomy: A Nationwide Population-Based Cohort Study in South Korea. Journal of Clinical Medicine. 2021;10(15). doi: 10.3390/jcm10153253. PubMed PMID: WOS:000682074800001.	Excluded from the final analysis	The article was excluded from the analysis due to the lack of relation between the title and the abstract of the article with the subject under study
806	Chen J, Sun YH, Fu T, Lu SY, Shi WM, Zhao JH, et al. Risk of incident cardiovascular disease among patients with gastrointestinal disorder: a prospective cohort study of 330751 individuals. European Heart Journal-Quality of Care and Clinical Outcomes. 2023. doi: 10.1093/ehjqcco/qcad059. PubMed PMID: WOS:001186209200001.	Excluded from the final analysis	The article was excluded from the analysis due to the lack of relation between the title and the abstract of the article with the subject under study

807	Chen CH, Lin CL, Kao CH. The Risk of Venous Thromboembolism in Patients with Gallstones. Int J Environ Res Public Health. 2020;17(8). Epub 20200423. doi: 10.3390/ijerph17082930. PubMed PMID: 32340378; PubMed Central PMCID: PMCPMC7215658.	Excluded from the final analysis	The article was excluded from the analysis due to the lack of relation between the title and the abstract of the article with the subject under study
808	Fletcher A, Amery A, Birkenhager W, Bulpitt C, Clement D, Deleeuw P, et al. RISKS AND BENEFITS IN THE TRIAL OF THE EUROPEAN WORKING PARTY ON HIGH BLOOD-PRESSURE IN THE ELDERLY. Journal of Hypertension. 1991;9(3):225-30. doi: 10.1097/00004872-199103000-00005. PubMed PMID: WOS:A1991FE35100005.	Excluded from the final analysis	The article was excluded from the analysis due to the lack of relation between the title and the abstract of the article with the subject under study
809	Gijsen AF, Vaassen HGM, Vahrmeijer AL, Geelkerken RH, Liem MSL, Bockhorn M, et al. Robot-assisted and fluorescence-guided remnant-cholecystectomy: a prospective dual-center cohort study. Hpb. 2023;25(7):820-5. doi: 10.1016/j.hpb.2023.03.015. PubMed PMID: WOS:001027452700001.	Excluded from the final analysis	The article was excluded from the analysis due to the lack of relation between the title and the abstract of the article with the subject under study
810	Massarrat S, Paidlik A, Pittner P, Schmitz-Moormann P, Wurbs M. The role of certain habits and various diseases in the occurrence of gastritis. Hepatogastroenterology. 1983;30(6):249-53. PubMed PMID: 6676145.	Excluded from the final analysis	The article was excluded from the analysis due to the lack of relation between the title and the abstract of the article with the subject under study
811	Klurfeld DM. The role of dietary fiber in gastrointestinal disease. J Am Diet Assoc. 1987;87(9):1172-7. PubMed PMID: 3040840.	Excluded from the final analysis	The article was excluded from the analysis due to the lack of relation between the title and the abstract of the article with the subject under study

812	Arora S, Anubhuti. Role of neuropeptides in appetite regulation and obesity--a review. Neuropeptides. 2006;40(6):375-401. Epub 20060828. doi: 10.1016/j.npep.2006.07.001. PubMed PMID: 16935329.	Excluded from the final analysis	The article was excluded from the analysis due to the lack of relation between the title and the abstract of the article with the subject under study
813	Czupryniak L, Drzewoski J. [The role of pharmacotherapy for treatment of obesity in adults]. Pol Merkur Lekarski. 2000;9(53):741-5. PubMed PMID: 11204319.	Excluded from the final analysis	The article was excluded from the analysis due to the lack of relation between the title and the abstract of the article with the subject under study
814	Thompson JS. The role of prophylactic cholecystectomy in the short-bowel syndrome. Archives of Surgery. 1996;131(5):556-9. PubMed PMID: WOS:A1996UK35400033.	Excluded from the final analysis	The article was excluded from the analysis due to the lack of relation between the title and the abstract of the article with the subject under study
815	Fuso L, Cisternino L, Di Napoli A, Di Cosmo V, Tramaglino LM, Basso S, et al. Role of spirometric and arterial gas data in predicting pulmonary complications after abdominal surgery. Respiratory Medicine. 2000;94(12):1171-6. doi: 10.1053/rmed.2000.0946. PubMed PMID: WOS:000171944500005.	Excluded from the final analysis	The article was excluded from the analysis due to the lack of relation between the title and the abstract of the article with the subject under study
816	Min SK, Kim SY, Park YJ, Lee W, Jung IM, Lee T, et al. Role of three-dimensional computed tomography venography as a powerful navigator for varicose vein surgery. J Vasc Surg. 2010;51(4):893-9. doi: 10.1016/j.jvs.2009.10.117. PubMed PMID: 20347685.	Excluded from the final analysis	The article was excluded from the analysis due to the lack of relation between the title and the abstract of the article with the subject under study

817	Min SK, Kim SY, Park YJ, Lee W, Jung IM, Lee T, et al. Role of three-dimensional computed tomography venography as a powerful navigator for varicose vein surgery. Journal of Vascular Surgery. 2010;51(4):893-9. doi: 10.1016/j.jvs.2009.10.117. PubMed PMID: WOS:000276220000013.	Excluded from the final analysis	The article was excluded from the analysis due to the lack of relation between the title and the abstract of the article with the subject under study
818	Suleimanov V, Naser H, Al-Taweel A. Rupture of a Huge Pancreatic Pseudocyst in a Superobese Patient: A Condition Mimicking Pulmonary Embolism. Cureus Journal of Medical Science. 2023;15(11). doi: 10.7759/cureus.49643. PubMed PMID: WOS:001122708500008.	Excluded from the final analysis	The article was excluded from the analysis due to the lack of relation between the title and the abstract of the article with the subject under study
819	Sunkara P, Shah PK, Rakshit K, Choudhary SR, Bohidar NP, Dubey SK. Rupture of Cystic Artery Pseudoaneurysm: a Rare Complication of Acute Cholecystitis. Indian Journal of Surgery. 2018;80(1):87-9. doi: 10.1007/s12262-017-1667-2. PubMed PMID: WOS:000428377600017.	Excluded from the final analysis	The article was excluded from the analysis due to the lack of relation between the title and the abstract of the article with the subject under study
820	Wang S, Kou C, Liu Y, Li B, Tao Y, D'Arcy C, et al. Rural-urban differences in the prevalence of chronic disease in northeast China. Asia Pac J Public Health. 2015;27(4):394-406. Epub 20140922. doi: 10.1177/1010539514551200. PubMed PMID: 25246500.	Excluded from the final analysis	The article was excluded from the analysis due to the lack of relation between the title and the abstract of the article with the subject under study
821	Wang SB, Kou CG, Liu YW, Li B, Tao YC, D'Arcy C, et al. Rural-Urban Differences in the Prevalence of Chronic Disease in Northeast China. Asia-Pacific Journal of Public Health. 2015;27(4):394-406. doi: 10.1177/1010539514551200. PubMed PMID: WOS:000354485600004.	Excluded from the final analysis	The article was excluded from the analysis due to the lack of relation between the title and the abstract of the article with the subject under study

822	Leinonen KS, Poutanen KS, Mykkänen HM. Rye bread decreases serum total and LDL cholesterol in men with moderately elevated serum cholesterol. Journal of Nutrition. 2000;130(2):164-70. doi: 10.1093/jn/130.2.164. PubMed PMID: WOS:000085167600007.	Excluded from the final analysis	The article was excluded from the analysis due to the lack of relation between the title and the abstract of the article with the subject under study
823	Leahy AL, Darzi AW, Murchan PM, Ogorman S, Hamilton S, Tanner WA, Keane FBV. A SAFE NEW PROCEDURE FOR HIGH-RISK PATIENTS WITH SYMPTOMATIC GALLSTONES. British Journal of Surgery. 1991;78(11):1319-20. doi: 10.1002/bjs.1800781115. PubMed PMID: WOS:A1991GV01700013.	Excluded from the final analysis	The article was excluded from the analysis due to the lack of relation between the title and the abstract of the article with the subject under study
824	Rana SS, Kumar A, Lal A, Sharma R, Kang M, Gorsi U, Gupta R. Safety and efficacy of angioembolisation followed by endoscopic ultrasound guided transmural drainage for pancreatic fluid collections associated with arterial pseudoaneurysm. Pancreatology. 2017;17(5):658-62. Epub 20170819. doi: 10.1016/j.pan.2017.08.002. PubMed PMID: 28843715.	Excluded from the final analysis	The article was excluded from the analysis due to the lack of relation between the title and the abstract of the article with the subject under study
825	Nojkov B, Cappell MS. Safety and efficacy of ERCP after recent myocardial infarction or unstable angina. Gastrointest Endosc. 2010;72(4):870-80. doi: 10.1016/j.gie.2010.06.024. PubMed PMID: 20883868.	Excluded from the final analysis	The article was excluded from the analysis due to the lack of relation between the title and the abstract of the article with the subject under study
826	Shabanzadeh DM, Skaaby T, Sorensen LT, Jorgensen T. Screen-detected gallstone disease and cardiovascular disease. European Journal of Epidemiology. 2017;32(6):501-10. doi: 10.1007/s10654-017-0263-x. PubMed PMID: WOS:000407244800006.	Excluded from the final analysis	The article was excluded from the analysis due to the lack of relation between the title and the abstract of the article with the subject under study

827	Sethi H, Peddu P, Prachalias A, Kane P, Karani J, Rela M, Heaton N. Selective embolization for bleeding visceral artery pseudoaneurysms in patients with pancreatitis. Hepatobiliary Pancreat Dis Int. 2010;9(6):634-8. PubMed PMID: 21134834.	Excluded from the final analysis	The article was excluded from the analysis due to the lack of relation between the title and the abstract of the article with the subject under study
828	Ljung R, Rück C, Mattsson F, Bexelius TS, Lagergren J, Lindblad M. Selective Serotonin Reuptake Inhibitors and the Risk of Acute Pancreatitis <i>A Swedish Population</i>-<i>Based Case</i>-<i>Control Study</i>. Journal of Clinical Psychopharmacology. 2012;32(3):336-40. doi: 10.1097/JCP.0b013e318253d71a. PubMed PMID: WOS:000304129500006.	Excluded from the final analysis	The article was excluded from the analysis due to the lack of relation between the title and the abstract of the article with the subject under study
829	van Boxel EJ, Rahman S, Lai K, Boulos N, Davis N. Semaglutide treatment for children with obesity: an observational study. Arch Dis Child. 2024;109(10):822-5. Epub 20240925. doi: 10.1136/archdischild-2023-326687. PubMed PMID: 38471743.	Excluded from the final analysis	The article was excluded from the analysis due to the lack of relation between the title and the abstract of the article with the subject under study
830	Yang QY, Ouyang J, Yang JD. Sepsis as an important risk factor for gastrointestinal bleeding in acute coronary syndrome patients: Two case reports. Medicine (Baltimore). 2018;97(36):e12273. doi: 10.1097/md.0000000000012273. PubMed PMID: 30200168; PubMed Central PMCID: PMCPMC6133616.	Excluded from the final analysis	The article was excluded from the analysis due to the lack of relation between the title and the abstract of the article with the subject under study
831	Czepiel J, Merc A, Jedrychowski M, Cholewczuk A, Biesiada G, Garlicki A. Septic shock caused by <i>Raoultella ornithinolytica</i> in a patient with Caroli syndrome. Reviews and Research in Medical Microbiology. 2024;35(4):227-9. doi: 10.1097/mrm.0000000000000388. PubMed PMID: WOS:001321268900002.	Excluded from the final analysis	The article was excluded from the analysis due to the lack of relation between the title and the abstract of the article with the subject under study

832	Fernandes V, Alfaro TM, Baptista JP, Regateiro FS, Fradinho F, Cordeiro CR. Severe ketorolac-induced asthma diagnosed by chest computed tomography. J Thorac Dis. 2017;9(Suppl 16):S1567-s9. doi: 10.21037/jtd.2017.11.36. PubMed PMID: 29255644; PubMed Central PMCID: PMCPMC5717355.	Excluded from the final analysis	The article was excluded from the analysis due to the lack of relation between the title and the abstract of the article with the subject under study
833	Okumura K, Shoji F, Yoshida M, Mizuta A, Makino I, Higashi H. Severe sepsis caused by Aeromonas hydrophila in a patient using tocilizumab: a case report. J Med Case Rep. 2011;5:499. Epub 20111005. doi: 10.1186/1752-1947-5-499. PubMed PMID: 21970314; PubMed Central PMCID: PMCPMC3214171.	Excluded from the final analysis	The article was excluded from the analysis due to the lack of relation between the title and the abstract of the article with the subject under study
834	Nikfarjam M, Harnaen E, Tufail F, Muralidharan V, Fink MA, Starkey G, et al. Sex Differences and Outcomes of Management of Acute Cholecystitis. Surgical Laparoscopy Endoscopy & Percutaneous Techniques. 2013;23(1):61-5. doi: 10.1097/SLE.0b013e3182773e52. PubMed PMID: WOS:000314813300025.	Excluded from the final analysis	The article was excluded from the analysis due to the lack of relation between the title and the abstract of the article with the subject under study
835	Dourakis SP, Tolis G. Sex hormonal preparations and the liver. Eur J Contracept Reprod Health Care. 1998;3(1):7-16. doi: 10.3109/13625189809167479. PubMed PMID: 9678067.	Excluded from the final analysis	The article was excluded from the analysis due to the lack of relation between the title and the abstract of the article with the subject under study
836	Pi-Sunyer FX. Short-term medical benefits and adverse effects of weight loss. Ann Intern Med. 1993;119(7 Pt 2):722-6. doi: 10.7326/0003-4819-119-7_part_2-199310011-00019. PubMed PMID: 8363205.	Excluded from the final analysis	The article was excluded from the analysis due to the lack of relation between the title and the abstract of the article with the subject under study

837	Pisunyer FX. SHORT-TERM MEDICAL BENEFITS AND ADVERSE-EFFECTS OF WEIGHT-LOSS. Annals of Internal Medicine. 1993;119(7):722-6. doi: 10.7326/0003-4819-119-7_Part_2-199310011-00019. PubMed PMID: WOS:A1993MA33000019.	Excluded from the final analysis	The article was excluded from the analysis due to the lack of relation between the title and the abstract of the article with the subject under study
838	Al-Salem AH. Should cholecystectomy be performed concomitantly with splenectomy in children with sickle-cell disease? Pediatr Surg Int. 2003;19(1-2):71-4. Epub 20030116. doi: 10.1007/s00383-002-0804-5. PubMed PMID: 12721729.	Excluded from the final analysis	The article was excluded from the analysis due to the lack of relation between the title and the abstract of the article with the subject under study
839	Karayalcin G, Rosner F, Kim KY, Chandra P, Aballi AJ. Sickle cell anemia- clinical manifestations in 100 patients and review of the literature. Am J Med Sci. 1975;269(1):51-68. doi: 10.1097/00000441-197501000-00007. PubMed PMID: 1130436.	Excluded from the final analysis	The article was excluded from the analysis due to the lack of relation between the title and the abstract of the article with the subject under study
840	Chacko S, Jadhav U, Ghewade B, Wagh P, Prasad R, Wanjari MB. Sickle Cell Disease (SCD) Leading to Pulmonary Arterial Hypertension (PAH) and Cholelithiasis (CL). Cureus. 2023;15(4):e37113. Epub 20230404. doi: 10.7759/cureus.37113. PubMed PMID: 37153282; PubMed Central PMCID: PMCPMC10159007.	Excluded from the final analysis	The article was excluded from the analysis due to the lack of relation between the title and the abstract of the article with the subject under study
841	Tarway NK, Sharma SP, Gupta T, Kanyal VS, Kumar B, Nair RK. Single Anesthesia ERCP and Laparoscopic Cholecystectomy for Management of Common Bile Duct Stones. Journal of Marine Medical Society. 2024;26(2):225-9. doi: 10.4103/jmms.jmms_124_23. PubMed PMID: WOS:001316507300017.	Excluded from the final analysis	The article was excluded from the analysis due to the lack of relation between the title and the abstract of the article with the subject under study

842	Hernández-Rodríguez J, Tan CD, Rodríguez ER, Hoffman GS. Single-organ gallbladder vasculitis: characterization and distinction from systemic vasculitis involving the gallbladder. An analysis of 61 patients. Medicine (Baltimore). 2014;93(24):405-13. doi: 10.1097/md.0000000000000205. PubMed PMID: 25500710; PubMed Central PMCID: PMCPMC4602437.	Excluded from the final analysis	The article was excluded from the analysis due to the lack of relation between the title and the abstract of the article with the subject under study
843	Murakami S, Terakado M, Misumi M, Tsuji Y, Okubo K, Hirayama R, et al. Situs inversus totalis with malignant lymphoma of the stomach: Report of a case. Surgery Today. 2003;33(7):533-6. doi: 10.1007/s10595-002-2530-z. PubMed PMID: WOS:000183776600011.	Excluded from the final analysis	The article was excluded from the analysis due to the lack of relation between the title and the abstract of the article with the subject under study
844	Onizuka Y, Mizuta Y, Isomoto H, Takeshima F, Murase K, Miyazaki M, et al. Sludge and stone formation in the gallbladder in bedridden elderly patients with cerebrovascular disease: influence of feeding method. Journal of Gastroenterology. 2001;36(5):330-7. doi: 10.1007/s005350170099. PubMed PMID: WOS:000168840100006.	Excluded from the final analysis	The article was excluded from the analysis due to the lack of relation between the title and the abstract of the article with the subject under study
845	Hodge AM, English DR, Giles GG, Flicker L. Social connectedness and predictors of successful ageing. Maturitas. 2013;75(4):361-6. Epub 20130606. doi: 10.1016/j.maturitas.2013.05.002. PubMed PMID: 23746413.	Excluded from the final analysis	The article was excluded from the analysis due to the lack of relation between the title and the abstract of the article with the subject under study
846	Backman L, Hallberg D. Some somatic complications after small intestinal bypass operations for obesity. Possible factors of significance in the incidence. Acta Chir Scand. 1975;141(8):790-800. PubMed PMID: 1217445.	Excluded from the final analysis	The article was excluded from the analysis due to the lack of relation between the title and the abstract of the article with the subject under study

847	Navarro SM, Chen S, Situ X, Corwin MT, Loehfelm T, Fananapazir G. Sonographic Assessment of Acute Versus Chronic Cholecystitis: An Ultrasound Probability Stratification Model. J Ultrasound Med. 2023;42(6):1257-65. Epub 20221201. doi: 10.1002/jum.16138. PubMed PMID: 36457230; PubMed Central PMCID: PMCPMC10191874.	Excluded from the final analysis	The article was excluded from the analysis due to the lack of relation between the title and the abstract of the article with the subject under study
848	Gica N, Radoi A, Gica C, Panaitescu AM, Peltecu G, Hulua I. Sonographic Detection of Fetal Cholelithiasis. Diagnostics. 2023;13(18). doi: 10.3390/diagnostics13182900. PubMed PMID: WOS:001076992200001.	Excluded from the final analysis	The article was excluded from the analysis due to the lack of relation between the title and the abstract of the article with the subject under study
849	Patriquin HB, DiPietro M, Barber FE, Teele RL. Sonography of thickened gallbladder wall: causes in children. AJR Am J Roentgenol. 1983;141(1):57-60. doi: 10.2214/ajr.141.1.57. PubMed PMID: 6602530.	Excluded from the final analysis	The article was excluded from the analysis due to the lack of relation between the title and the abstract of the article with the subject under study
850	Sriram K, Ramasubramanian V, Meguid MM. Special postoperative diet orders: Irrational, obsolete, and imprudent. Nutrition. 2016;32(4):498-502. doi: 10.1016/j.nut.2015.10.017. PubMed PMID: WOS:000371851000014.	Excluded from the final analysis	The article was excluded from the analysis due to the lack of relation between the title and the abstract of the article with the subject under study
851	Stewart L, Griffiss JM, Way LW. Spectrum of gallstone disease in the veterans population. Am J Surg. 2005;190(5):746-51. doi: 10.1016/j.amjsurg.2005.07.014. PubMed PMID: 16226952.	Excluded from the final analysis	The article was excluded from the analysis due to the lack of relation between the title and the abstract of the article with the subject under study

852	Stewart L, Griffiss JM, Way LW. Spectrum of gallstone disease in the veterans population. American Journal of Surgery. 2005;190(5):746-51. doi: 10.1016/j.amjsurg.2005.07.014. PubMed PMID: WOS:000232935200017.	Excluded from the final analysis	The article was excluded from the analysis due to the lack of relation between the title and the abstract of the article with the subject under study
853	Bessa SS, Katri KM, Abdel-Salam WN, El-Kayal el SA, Tawfik TA. Spinal versus general anesthesia for day-case laparoscopic cholecystectomy: a prospective randomized study. J Laparoendosc Adv Surg Tech A. 2012;22(6):550-5. Epub 20120611. doi: 10.1089/lap.2012.0110. PubMed PMID: 22686181.	Excluded from the final analysis	The article was excluded from the analysis due to the lack of relation between the title and the abstract of the article with the subject under study
854	Kumar S, Chauhan S. Splenectomy in Thalassemia: The Role of Surgery as an Adjunct to Medical Management. Cureus. 2024;16(6):e62834. Epub 20240621. doi: 10.7759/cureus.62834. PubMed PMID: 39036111; PubMed Central PMCID: PMCPMC11260393.	Excluded from the final analysis	The article was excluded from the analysis due to the lack of relation between the title and the abstract of the article with the subject under study
855	Al-Salem AH, Naserullah Z, Qaisaruddin S, Al-Abkari H, Al-Faraj A, Yassin YM. Splenic complications of the sickling syndromes and the role of splenectomy. J Pediatr Hematol Oncol. 1999;21(5):401-6. doi: 10.1097/00043426-199909000-00012. PubMed PMID: 10524454.	Excluded from the final analysis	The article was excluded from the analysis due to the lack of relation between the title and the abstract of the article with the subject under study
856	Al-Salem AH, Naserullah Z, Qaisaruddin S, Al-Abkari H, Al-Faraj A, Yassin YM. Splenic complications of the sickling syndromes and the role of splenectomy. Journal of Pediatric Hematology Oncology. 1999;21(5):401-6. doi: 10.1097/00043426-199909000-00012. PubMed PMID: WOS:000082935400013.	Excluded from the final analysis	The article was excluded from the analysis due to the lack of relation between the title and the abstract of the article with the subject under study

857	Covello B, Miller J, Fourzali R. Splenic vein stenting for recurrent chylous ascites in sinistral portal hypertension: a case report. CVIR Endovasc. 2021;4(1):26. Epub 20210303. doi: 10.1186/s42155-021-00213-x. PubMed PMID: 33656619; PubMed Central PMCID: PMCPMC7930171.	Excluded from the final analysis	The article was excluded from the analysis due to the lack of relation between the title and the abstract of the article with the subject under study
858	Covello B, Miller J, Fourzali R. Splenic vein stenting for recurrent chylous ascites in sinistral portal hypertension: a case report. Cvir Endovascular. 2021;4(1). doi: 10.1186/s42155-021-00213-x. PubMed PMID: WOS:000703917000001.	Excluded from the final analysis	The article was excluded from the analysis due to the lack of relation between the title and the abstract of the article with the subject under study
859	Bhattacharjee PK, Choudhury D, Rai H, Ram N, Chattopadhyay D, Roy RP. Spontaneous perforation of common bile duct: a rare complication of choledocholithiasis. Indian J Surg. 2009;71(2):92-4. Epub 20090502. doi: 10.1007/s12262-009-0024-5. PubMed PMID: 23133123; PubMed Central PMCID: PMCPMC3452614.	Excluded from the final analysis	The article was excluded from the analysis due to the lack of relation between the title and the abstract of the article with the subject under study
860	Bhattacharjee PK, Choudhury D, Rai H, Ram N, Chattopadhyay D, Roy RP. Spontaneous perforation of common bile duct: a rare complication of choledocholithiasis. Indian Journal of Surgery. 2009;71(2):92-4. doi: 10.1007/s12262-009-0024-5. PubMed PMID: WOS:000266440700008.	Excluded from the final analysis	The article was excluded from the analysis due to the lack of relation between the title and the abstract of the article with the subject under study
861	Jang H, Park CH, Park Y, Jeong E, Lee N, Kim J, Jo Y. Spontaneous resolution of gallbladder hematoma in blunt traumatic injury: A case report. World J Clin Cases. 2021;9(28):8518-23. doi: 10.12998/wjcc.v9.i28.8518. PubMed PMID: 34754862; PubMed Central PMCID: PMCPMC8554416.	Excluded from the final analysis	The article was excluded from the analysis due to the lack of relation between the title and the abstract of the article with the subject under study

862	Coulier B, Maldague P, Pierard F. Spontaneous transhepatic rupture of the gallbladder with massive hemoperitoneum. Jbr-btr. 2012;95(2):92-4. doi: 10.5334/jbr-btr.149. PubMed PMID: 22764665.	Excluded from the final analysis	The article was excluded from the analysis due to the lack of relation between the title and the abstract of the article with the subject under study
863	Ronni-Sivula H. The state of health of patients previously operated on for primary hyperparathyroidism compared with randomized controls. Ann Chir Gynaecol. 1985;74(2):60-5. PubMed PMID: 4026176.	Excluded from the final analysis	The article was excluded from the analysis due to the lack of relation between the title and the abstract of the article with the subject under study
864	Biétry FA, Reich O, Schwenkglenks M, Meier CR. Statin use and risk of cholecystectomy - A case-control analysis using Swiss claims data. Expert Opinion On Drug Safety. 2016;15(12):1577-82. doi: 10.1080/14740338.2016.1240782. PubMed PMID: WOS:000389028600002.	Excluded from the final analysis	The article was excluded from the analysis due to the lack of relation between the title and the abstract of the article with the subject under study
865	Bodmer M, Brauchli YB, Krähenbühl S, Jick SS, Meier CR. Statin Use and Risk of Gallstone Disease Followed by Cholecystectomy. Jama-Journal of the American Medical Association. 2009;302(18):2001-7. doi: 10.1001/jama.2009.1601. PubMed PMID: WOS:000271619700027.	Excluded from the final analysis	The article was excluded from the analysis due to the lack of relation between the title and the abstract of the article with the subject under study
866	Lowyck I, Fevery J. Statins in hepatobiliary diseases: effects, indications and risks. Acta Gastro-Enterologica Belgica. 2007;70(4):381-8. PubMed PMID: WOS:000253817700012.	Excluded from the final analysis	The article was excluded from the analysis due to the lack of relation between the title and the abstract of the article with the subject under study

867	Stender S, Frikke-Schmidt R, Nordestgaard BG, Tybjærg-Hansen A. Sterol Transporter Adenosine Triphosphate-Binding Cassette Transporter G8, Gallstones, and Biliary Cancer in 62,000 Individuals from the General Population. Hepatology. 2011;53(2):640-8. doi: 10.1002/hep.24046. PubMed PMID: WOS:000286855600030.	Excluded from the final analysis	The article was excluded from the analysis due to the lack of relation between the title and the abstract of the article with the subject under study
868	Méndez-Sánchez N, Bahena-Aponte J, Chávez-Tapia NC, Motola-Kuba D, Sánchez-Lara K, Ponciano-Radríguez G, et al. Strong association between gallstones and cardiovascular disease. American Journal of Gastroenterology. 2005;100(4):827-30. doi: 10.1111/j.1572-0241.2005.41214.x. PubMed PMID: WOS:000227720400018.	Excluded from the final analysis	The article was excluded from the analysis due to the lack of relation between the title and the abstract of the article with the subject under study
869	Qin J, Han TQ, Cai XX, Jiang ZH, Yang XM, Zhang Y, et al. [Study on the characteristics of inheritance and epidemiology in one pedigree with gallstone disease]. Zhonghua Liu Xing Bing Xue Za Zhi. 2005;26(6):448-50. PubMed PMID: 16185464.	Excluded from the final analysis	The article was excluded from the analysis due to the lack of relation between the title and the abstract of the article with the subject under study
870	Ando H, Ootake Y, Asaka S. Subacute pulmonary hypertension due to pulmonary tumor microembolism as a clinical manifestation of occult gallbladder adenocarcinoma. Jpn Circ J. 1997;61(1):82-6. doi: 10.1253/jcj.61.82. PubMed PMID: 9070964.	Excluded from the final analysis	The article was excluded from the analysis due to the lack of relation between the title and the abstract of the article with the subject under study
871	Bornman PC, Terblanche J. Subtotal cholecystectomy: for the difficult gallbladder in portal hypertension and cholecystitis. Surgery. 1985;98(1):1-6. PubMed PMID: 3892743.	Excluded from the final analysis	The article was excluded from the analysis due to the lack of relation between the title and the abstract of the article with the subject under study

872	Lata I, Mandelia A, Mandal K. Successful Antenatal Diagnosis and Management of a Rare Case of Congenital Fetal Choledochal Cyst. Maternal-Fetal Medicine. 2021;3(3):225-8. doi: 10.1097/fm9.0000000000000075. PubMed PMID: WOS:000706794500012.	Excluded from the final analysis	The article was excluded from the analysis due to the lack of relation between the title and the abstract of the article with the subject under study
873	Bramucci A, Miceli F, Fontana A, Tusini N, Sereni G, Sassatelli R. Successful Endovascular and Endoscopic Treatment of a Symptomatic Celiac Artery Aneurysm for Obstructive Jaundice: A Clinical Case Report. Annals of Vascular Surgery. 2022;80. doi: 10.1016/j.avsg.2021.10.057. PubMed PMID: WOS:000819841600056.	Excluded from the final analysis	The article was excluded from the analysis due to the lack of relation between the title and the abstract of the article with the subject under study
874	Lee SI, Lim BH, Heo WG, Kim YJ, Kim TH. Successful Removal of a Large Common Bile Duct Stone by Using Direct Peroral Cholangioscopy and Laser Lithotripsy in a Patient with Severe Kyphosis. Clin Endosc. 2016;49(4):395-8. Epub 20160322. doi: 10.5946/ce.2015.109. PubMed PMID: 27000425; PubMed Central PMCID: PMCPMC4977743.	Excluded from the final analysis	The article was excluded from the analysis due to the lack of relation between the title and the abstract of the article with the subject under study
875	Hofmann AF, Amelsberg A, Esch O, Schteingart CD, Lyche K, Jinich H, et al. Successful topical dissolution of cholesterol gallbladder stones using ethyl propionate. Dig Dis Sci. 1997;42(6):1274-82. doi: 10.1023/a:1018818527187. PubMed PMID: 9201095.	Excluded from the final analysis	The article was excluded from the analysis due to the lack of relation between the title and the abstract of the article with the subject under study
876	Gutkin E, Hussain SA, Kim SH. The Successful Treatment of Chronic Cholecystitis with SpyGlass Cholangioscopy-Assisted Gallbladder Drainage and Irrigation through Self-Expandable Metal Stents. Gut and Liver. 2012;6(1):136-8. doi: 10.5009/gnl.2012.6.1.136. PubMed PMID: WOS:000299367400022.	Excluded from the final analysis	The article was excluded from the analysis due to the lack of relation between the title and the abstract of the article with the subject under study

877	Rai R, Nagral S, Nagral A. Surgery in a patient with liver disease. J Clin Exp Hepatol. 2012;2(3):238-46. Epub 20120921. doi: 10.1016/j.jceh.2012.05.003. PubMed PMID: 25755440; PubMed Central PMCID: PMCPMC3940091.	Excluded from the final analysis	The article was excluded from the analysis due to the lack of relation between the title and the abstract of the article with the subject under study
878	Kahng KU, Roslyn JJ. SURGICAL ISSUES FOR THE ELDERLY PATIENT WITH HEPATOBILIARY DISEASE. Surgical Clinics of North America. 1994;74(2):345-73. doi: 10.1016/s0039-6109(16)46286-9. PubMed PMID: WOS:A1994NH33700007.	Excluded from the final analysis	The article was excluded from the analysis due to the lack of relation between the title and the abstract of the article with the subject under study
879	Sebai A, Elaifia R, Atri S, Hammami Y, Haddad A, Kacem JM. Surgical management of a proximal splenic artery aneurysm: A case report. International Journal of Surgery Case Reports. 2024;117. doi: 10.1016/j.ijscr.2024.109499. PubMed PMID: WOS:001208599700001.	Excluded from the final analysis	The article was excluded from the analysis due to the lack of relation between the title and the abstract of the article with the subject under study
880	Al-Abbasi G, Alhilfy AA, Al-Jasim A. Surgical Management of Gallstone Ileus in Low-Settings Hospital during COVID-19 Outbreak: A Case Report. Surg J (N Y). 2021;7(2):e54-e8. Epub 20210525. doi: 10.1055/s-0041-1725160. PubMed PMID: 34056103; PubMed Central PMCID: PMCPMC8149159.	Excluded from the final analysis	The article was excluded from the analysis due to the lack of relation between the title and the abstract of the article with the subject under study
881	Drinnon K, Puckett Y. Surgical Management of Gallstone Ileus: A Clinical Case Report. Cureus. 2021;13(12):e20141. Epub 20211203. doi: 10.7759/cureus.20141. PubMed PMID: 35003974; PubMed Central PMCID: PMCPMC8723775.	Excluded from the final analysis	The article was excluded from the analysis due to the lack of relation between the title and the abstract of the article with the subject under study

882	Parker MA, Kragh N, Sandoval J, Erabti S, Soliman B. Surgical Management of Gallstone Ileus: Approach, Outcome, Case Report, and Literature Review. Cureus. 2024;16(9):e69930. Epub 20240922. doi: 10.7759/cureus.69930. PubMed PMID: 39439607; PubMed Central PMCID: PMCPMC11495830.	Excluded from the final analysis	The article was excluded from the analysis due to the lack of relation between the title and the abstract of the article with the subject under study
883	Varma V, Behera A, Kaman L, Chattopadhyay S, Nundy S. Surgical management of portal cavernoma cholangiopathy. J Clin Exp Hepatol. 2014;4(Suppl 1):S77-84. Epub 20130917. doi: 10.1016/j.jceh.2013.07.005. PubMed PMID: 25755599; PubMed Central PMCID: PMCPMC4244827.	Excluded from the final analysis	The article was excluded from the analysis due to the lack of relation between the title and the abstract of the article with the subject under study
884	Alshamali DM, Horan M, Alhmaidy O, Mozaffar WK, Mozaffar JA, Al Ahmad Y. Surgical removal of giant adrenal lipoma with gallstones in limited resources areas: A case report. Int J Surg Case Rep. 2024;121:109953. Epub 20240715. doi: 10.1016/j.ijscr.2024.109953. PubMed PMID: 39029216; PubMed Central PMCID: PMCPMC11295541.	Excluded from the final analysis	The article was excluded from the analysis due to the lack of relation between the title and the abstract of the article with the subject under study
885	Wetter LA, Way LW. SURGICAL THERAPY FOR GALLSTONE DISEASE. Gastroenterology Clinics of North America. 1991;20(1):157-69. PubMed PMID: WOS:A1991FB57200008.	Excluded from the final analysis	The article was excluded from the analysis due to the lack of relation between the title and the abstract of the article with the subject under study
886	Tracey JY, Moossa AR. [Surgical treatment of benign lesions and strictures of the bile ducts]. Chirurg. 2006;77(4):315-24. doi: 10.1007/s00104-006-1168-5. PubMed PMID: 16557406.	Excluded from the final analysis	The article was excluded from the analysis due to the lack of relation between the title and the abstract of the article with the subject under study

887	Lipsett PA, Pitt HA. Surgical treatment of choledochal cysts. J Hepatobiliary Pancreat Surg. 2003;10(5):352-9. doi: 10.1007/s00534-002-0797-4. PubMed PMID: 14598135.	Excluded from the final analysis	The article was excluded from the analysis due to the lack of relation between the title and the abstract of the article with the subject under study
888	Birgani NB, Motamedi P, Kanaani L. A survey on the today's world problem of increasing obesity. Progress in Nutrition. 2019;21:22-32. doi: 10.23751/pn.v21i2-S.6415. PubMed PMID: WOS:000509707300003.	Excluded from the final analysis	The article was excluded from the analysis due to the lack of relation between the title and the abstract of the article with the subject under study
889	Hoshi Y, Takeshima K, Matsuoka S, Hoshikawa T, Senuma K, Nakamura T, et al. Survival in a Case of Emphysematous Cholecystitis With Sepsis Caused by Clostridium perfringens. Cureus. 2023;15(11):e49705. Epub 20231130. doi: 10.7759/cureus.49705. PubMed PMID: 38033444; PubMed Central PMCID: PMCPMC10688239.	Excluded from the final analysis	The article was excluded from the analysis due to the lack of relation between the title and the abstract of the article with the subject under study
890	Erdogan MB, Kaplan M, Kazaz H, Salman B. Synchronous Open Heart Surgery and Laparoscopic Cholecystectomy: An Observational Case Study with 28 Patients. American Surgeon. 2017;83(3):314-21. PubMed PMID: WOS:000397998900029.	Excluded from the final analysis	The article was excluded from the analysis due to the lack of relation between the title and the abstract of the article with the subject under study
891	Jia LN, Jiang DW, Hu PC, Li X, Shi HC, Cheng DF, Zhang L. Synthesis and evaluation of ¹⁸ F-labeled bile acid compound: A potential PET imaging agent for FXR-related diseases. Nuclear Medicine and Biology. 2014;41(6):495-500. doi: 10.1016/j.nucmedbio.2014.03.016. PubMed PMID: WOS:000336946400009.	Excluded from the final analysis	The article was excluded from the analysis due to the lack of relation between the title and the abstract of the article with the subject under study

892	Yuan Z, Meyerholz DK, Twait EC, Kempuraj D, Williard DE, Samuel I. Systemic inflammation with multiorgan dysfunction is the cause of death in murine ligation-induced acute pancreatitis. J Gastrointest Surg. 2011;15(10):1670-8. Epub 20110729. doi: 10.1007/s11605-011-1643-2. PubMed PMID: 21800226.	Excluded from the final analysis	The article was excluded from the analysis due to the lack of relation between the title and the abstract of the article with the subject under study
893	Luthar Z, Golob A, Germ M, Vombergar B, Kreft I. Tartary Buckwheat in Human Nutrition. Plants (Basel). 2021;10(4). Epub 20210405. doi: 10.3390/plants10040700. PubMed PMID: 33916396; PubMed Central PMCID: PMCPMC8066602.	Excluded from the final analysis	The article was excluded from the analysis due to the lack of relation between the title and the abstract of the article with the subject under study
894	Akbar N, Yaseen T, Muhammad A, Danish M, Adeel M, Khan SA, et al. A Tertiary Care Center's Experience with Clinicopathological Characteristics of Gallbladder Carcinoma in Our Population. Euroasian J Hepatogastroenterol. 2022;12(1):35-9. doi: 10.5005/jp-journals-10018-1375. PubMed PMID: 35990861; PubMed Central PMCID: PMCPMC9357526.	Excluded from the final analysis	The article was excluded from the analysis due to the lack of relation between the title and the abstract of the article with the subject under study
895	Taher AT, Musallam KM, Cappellini MD. Thalassaemia intermedia: an update. Mediterr J Hematol Infect Dis. 2009;1(1):e2009004. Epub 20090829. doi: 10.4084/mjhid.2009.004. PubMed PMID: 21415986; PubMed Central PMCID: PMCPMC3033165.	Excluded from the final analysis	The article was excluded from the analysis due to the lack of relation between the title and the abstract of the article with the subject under study
896	Pantsyrev Iu M, Budzinskiĭ AA, Nozdrachev VI, Lagunchik BP, Konovalov A. [Therapeutic tactics in acute obstruction of the terminal part of the common bile duct]. Khirurgiia (Mosk). 1990;(10):3-8. PubMed PMID: 2283748.	Excluded from the final analysis	The article was excluded from the analysis due to the lack of relation between the title and the abstract of the article with the subject under study

897	Ma X, Gao J, Li Y, Xie J, Feng Z, Jia X, Chen W. Transplantation of splenic tissue after splenectomy: A case report. Exp Ther Med. 2022;24(4):612. Epub 20220803. doi: 10.3892/etm.2022.11549. PubMed PMID: 36160907; PubMed Central PMCID: PMCPMC9468849.	Excluded from the final analysis	The article was excluded from the analysis due to the lack of relation between the title and the abstract of the article with the subject under study
898	Patil SS, Pawar SC, Divekar V, Bakhshi RG. Transversus abdominis plane block for an emergency laparotomy in a high-risk, elderly patient. Indian J Anaesth. 2010;54(3):249-54. doi: 10.4103/0019-5049.65377. PubMed PMID: 20885876; PubMed Central PMCID: PMCPMC2933488.	Excluded from the final analysis	The article was excluded from the analysis due to the lack of relation between the title and the abstract of the article with the subject under study
899	Ladurner R, Kotsianos D, Mutschler W, Mussack T. Traumatic pneumobilia after cardiopulmonary resuscitation. European Journal of Medical Research. 2005;10(11):495-7. PubMed PMID: WOS:000233748500007.	Excluded from the final analysis	The article was excluded from the analysis due to the lack of relation between the title and the abstract of the article with the subject under study
900	Alexander DJ, Fowler R, McMahon MJ. TREATMENT OF ACUTE-PANCREATITIS CAUSED BY CALCULOUS OBSTRUCTION OF THE PANCREATIC DUCT BY ULTRASOUND-GUIDED PERCUTANEOUS DRAINAGE. Pancreas. 1992;7(1):105-8. doi: 10.1097/00006676-199201000-00014. PubMed PMID: WOS:A1992GV82700014.	Excluded from the final analysis	The article was excluded from the analysis due to the lack of relation between the title and the abstract of the article with the subject under study
901	Das AK, Chiura A, Conlin MJ, Eschelman D, Bagley DH. Treatment of biliary calculi using holmium:yttrium aluminum garnet laser. Gastrointestinal Endoscopy. 1998;48(2):207-9. doi: 10.1016/s0016-5107(98)70167-1. PubMed PMID: WOS:000075342400016.	Excluded from the final analysis	The article was excluded from the analysis due to the lack of relation between the title and the abstract of the article with the subject under study

902	Scarpioni R, Ricardi M, Albertazzi V, Melfa L. Treatment of dyslipidemia in chronic kidney disease: Effectiveness and safety of statins. World J Nephrol. 2012;1(6):184-94. doi: 10.5527/wjn.v1.i6.184. PubMed PMID: 24175258; PubMed Central PMCID: PMCPMC3782216.	Excluded from the final analysis	The article was excluded from the analysis due to the lack of relation between the title and the abstract of the article with the subject under study
903	Shearman DJ. The treatment of dyspepsia. Aust Fam Physician. 1977;6(8):1028-32. PubMed PMID: 921613.	Excluded from the final analysis	The article was excluded from the analysis due to the lack of relation between the title and the abstract of the article with the subject under study
904	Dotsenko YV, Naumov VG, Lyakishev AA, Bolotina MG, Bratchikova TV. Treatment of ischemic heart disease in postmenopausal women. Kardiologiya. 2001;41(3):64-70. PubMed PMID: WOS:000170939600017.	Excluded from the final analysis	The article was excluded from the analysis due to the lack of relation between the title and the abstract of the article with the subject under study
905	Sand J, Pakkala S, Nordback I. Twenty to thirty year follow-up after cholecystectomy. Hepato-Gastroenterology. 1996;43(9):534-7. PubMed PMID: WOS:A1996UT46000014.	Excluded from the final analysis	The article was excluded from the analysis due to the lack of relation between the title and the abstract of the article with the subject under study
906	Loreno M, Travali S, Bucceri AM, Scalisi G, Virgilio C, Brogna A. Ultrasonographic study of gallbladder wall thickness and emptying in cirrhotic patients without gallstones. Gastroenterol Res Pract. 2009;2009:683040. Epub 20090810. doi: 10.1155/2009/683040. PubMed PMID: 19680454; PubMed Central PMCID: PMCPMC2723919.	Excluded from the final analysis	The article was excluded from the analysis due to the lack of relation between the title and the abstract of the article with the subject under study

907	Sommerville AJ. ULTRASOUND IN INVESTIGATION OF BILIARY-TRACT DISEASE. Australasian Radiology. 1978;22(1):48-59. doi: 10.1111/j.1440-1673.1978.tb02048.x. PubMed PMID: WOS:A1978FU15900007.	Excluded from the final analysis	The article was excluded from the analysis due to the lack of relation between the title and the abstract of the article with the subject under study
908	Hanis CL, Hewett-Emmett D, Kubrusly LF, Maklad MN, Douglas TC, Mueller WH, et al. An ultrasound survey of gallbladder disease among Mexican Americans in Starr County, Texas: frequencies and risk factors. Ethn Dis. 1993;3(1):32-43. PubMed PMID: 8508103.	Excluded from the final analysis	The article was excluded from the analysis due to the lack of relation between the title and the abstract of the article with the subject under study
909	Launay D, Hatron PY, Queyrel V, Leteurtre E, Beaulieu S, Lemann M, et al. [Unusual association: hepatic peliosis and Crohn's disease]. Rev Med Interne. 2002;23(2):198-202. doi: 10.1016/s0248-8663(01)00537-9. PubMed PMID: 11876065.	Excluded from the final analysis	The article was excluded from the analysis due to the lack of relation between the title and the abstract of the article with the subject under study
910	Luu MB, Deziel DJ. Unusual Complications of Gallstones. Surgical Clinics of North America. 2014;94(2):377-+. doi: 10.1016/j.suc.2014.01.002. PubMed PMID: WOS:000335421100013.	Excluded from the final analysis	The article was excluded from the analysis due to the lack of relation between the title and the abstract of the article with the subject under study
911	Jiao XY, Mo YX, Wu Y, He JY, Zhang P, Hu RL, et al. Upregulated plasma and urinary levels of nucleosides as biological markers in the diagnosis of primary gallbladder cancer. Journal of Separation Science. 2014;37(21):3033-44. doi: 10.1002/jssc.201400638. PubMed PMID: WOS:000344250300003.	Excluded from the final analysis	The article was excluded from the analysis due to the lack of relation between the title and the abstract of the article with the subject under study

912	Colli A, Prati D, Fraquelli M, Segato S, Vescovi PP, Colombo F, et al. The Use of a Pocket-Sized Ultrasound Device Improves Physical Examination: Results of an In- and Outpatient Cohort Study. Plos One. 2015;10(3). doi: 10.1371/journal.pone.0122181. PubMed PMID: WOS:000352083900174.	Excluded from the final analysis	The article was excluded from the analysis due to the lack of relation between the title and the abstract of the article with the subject under study
913	Leuci D, Quinto V. [Usefulness of the follow-up in outpatients affected by liver cirrhosis]. Recenti Prog Med. 2008;99(1):32. PubMed PMID: 18389869.	Excluded from the final analysis	The article was excluded from the analysis due to the lack of relation between the title and the abstract of the article with the subject under study
914	Stender S, Tybjærg-Hansen A. Using human genetics to predict the effects and side-effects of drugs. Current Opinion in Lipidology. 2016;27(2):105-11. doi: 10.1097/mol.0000000000000280. PubMed PMID: WOS:000371635100002.	Excluded from the final analysis	The article was excluded from the analysis due to the lack of relation between the title and the abstract of the article with the subject under study
915	Loehfelm TW, Tse JR, Jeffrey RB, Kamaya A. The utility of hepatic artery velocity in diagnosing patients with acute cholecystitis. Abdom Radiol (NY). 2018;43(5):1159-67. doi: 10.1007/s00261-017-1288-z. PubMed PMID: 28840272.	Excluded from the final analysis	The article was excluded from the analysis due to the lack of relation between the title and the abstract of the article with the subject under study
916	Ambartsumyan L, Flores A, Nurko S, Rodriguez L. Utility of Octreotide in Advancing Enteral Feeds in Children with Chronic Intestinal Pseudo-Obstruction. Paediatr Drugs. 2016;18(5):387-92. doi: 10.1007/s40272-016-0189-x. PubMed PMID: 27520652.	Excluded from the final analysis	The article was excluded from the analysis due to the lack of relation between the title and the abstract of the article with the subject under study

917	Parodi HC, Gutiérrez S, Lattanzi M, Martínez R, Colombato LO. [Value of laboratory tests and echography in the diagnosis of biliary disease in the initial phase of acute pancreatitis]. Acta Gastroenterol Latinoam. 1990;20(3):137-44. PubMed PMID: 2095097.	Excluded from the final analysis	The article was excluded from the analysis due to the lack of relation between the title and the abstract of the article with the subject under study
918	Srinivasaiah N, Bhojak M, Jackson R, Woodcock S. Vascular emergencies in cholelithiasis and cholecystectomy: our experience with two cases and literature review. Hepatobiliary & Pancreatic Diseases International. 2008;7(2):217-20. PubMed PMID: WOS:000255014700019.	Excluded from the final analysis	The article was excluded from the analysis due to the lack of relation between the title and the abstract of the article with the subject under study
919	Sandhu SK, Choy G. Vasculitis of the gallbladder in early rheumatoid arthritis. BMJ Case Rep. 2013;2013. Epub 20130823. doi: 10.1136/bcr-2012-008228. PubMed PMID: 23975907; PubMed Central PMCID: PMCPMC3762444.	Excluded from the final analysis	The article was excluded from the analysis due to the lack of relation between the title and the abstract of the article with the subject under study
920	La Vecchia C, Decarli A, Pagano R. Vegetable consumption and risk of chronic disease. Epidemiology. 1998;9(2):208-10. PubMed PMID: 9504293.	Excluded from the final analysis	The article was excluded from the analysis due to the lack of relation between the title and the abstract of the article with the subject under study
921	La Vecchia C, Decarli A, Pagano R. Vegetable consumption and risk of chronic disease. Epidemiology. 1998;9(2):208-10. PubMed PMID: WOS:000072096000019.	Excluded from the final analysis	The article was excluded from the analysis due to the lack of relation between the title and the abstract of the article with the subject under study

922	Segasothy M, Phillips PA. Vegetarian diet: panacea for modern lifestyle diseases? Qjm-an International Journal of Medicine. 1999;92(9):531-44. doi: 10.1093/qjmed/92.9.531. PubMed PMID: WOS:000082687900009.	Excluded from the final analysis	The article was excluded from the analysis due to the lack of relation between the title and the abstract of the article with the subject under study
923	Leitzmann C. Vegetarian diets: what are the advantages? Forum Nutr. 2005;(57):147-56. doi: 10.1159/000083787. PubMed PMID: 15702597.	Excluded from the final analysis	The article was excluded from the analysis due to the lack of relation between the title and the abstract of the article with the subject under study
924	Itagaki H, Hagino T. Vitamin K deficiency-induced hemorrhagic shock after thoracentesis: a case report. BMC Gastroenterol. 2019;19(1):58. Epub 20190418. doi: 10.1186/s12876-019-0978-0. PubMed PMID: 30999880; PubMed Central PMCID: PMCPMC6472080.	Excluded from the final analysis	The article was excluded from the analysis due to the lack of relation between the title and the abstract of the article with the subject under study
925	Oudhoff JP, Timmermans DR, Knol DL, Bijnen AB, van der Wal G. Waiting for elective general surgery: impact on health related quality of life and psychosocial consequences. BMC Public Health. 2007;7:164. Epub 20070719. doi: 10.1186/1471-2458-7-164. PubMed PMID: 17640382; PubMed Central PMCID: PMCPMC1959190.	Excluded from the final analysis	The article was excluded from the analysis due to the lack of relation between the title and the abstract of the article with the subject under study
926	Oudhoff JP, Timmermans DR, Bijnen AB, van der Wal G. Waiting for elective general surgery: physical, psychological and social consequences. ANZ J Surg. 2004;74(5):361-7. doi: 10.1111/j.1445-1433.2004.02998.x. PubMed PMID: 15144258.	Excluded from the final analysis	The article was excluded from the analysis due to the lack of relation between the title and the abstract of the article with the subject under study

927	Oudhoff JP, Timmermans DRM, Bijnen AB, Van der Wal G. Waiting for elective general surgery: Physical, psychological and social consequences. Anz Journal of Surgery. 2004;74(5):361-7. doi: 10.1111/j.1445-1433.2004.02998.x. PubMed PMID: WOS:000221298300017.	Excluded from the final analysis	The article was excluded from the analysis due to the lack of relation between the title and the abstract of the article with the subject under study
928	Oudhoff JP, Timmermans DR, Knol DL, Bijnen AB, Van der Wal G. Waiting for elective surgery: effect on physical problems and postoperative recovery. ANZ J Surg. 2007;77(10):892-8. doi: 10.1111/j.1445-2197.2007.04268.x. PubMed PMID: 17803558.	Excluded from the final analysis	The article was excluded from the analysis due to the lack of relation between the title and the abstract of the article with the subject under study
929	Dubnov G, Brzezinski A, Berry EM. Weight control and the management of obesity after menopause: the role of physical activity. Maturitas. 2003;44(2):89-101. doi: 10.1016/s0378-5122(02)00328-6. PubMed PMID: WOS:000181384600001.	Excluded from the final analysis	The article was excluded from the analysis due to the lack of relation between the title and the abstract of the article with the subject under study
930	Bickler SW, DeMaio A. Western diseases: current concepts and implications for pediatric surgery research and practice. Pediatr Surg Int. 2008;24(3):251-5. Epub 20071218. doi: 10.1007/s00383-007-2095-3. PubMed PMID: 18087704.	Excluded from the final analysis	The article was excluded from the analysis due to the lack of relation between the title and the abstract of the article with the subject under study
931	Patel K, Devireddy N, Long C, Daya A, Cherneskie J, Krill K. When Blood Is Thicker Than Water: A Case of Acute Pancreatitis Secondary to Familial Hypertriglyceridemia. Cureus. 2024;16(1):e51511. Epub 20240102. doi: 10.7759/cureus.51511. PubMed PMID: 38304641; PubMed Central PMCID: PMCPMC10832547.	Excluded from the final analysis	The article was excluded from the analysis due to the lack of relation between the title and the abstract of the article with the subject under study

932	Hess W. [WHEN TO OPERATE IN DISEASES OF THE BILIARY TRACT AND PANCREAS]. Dia Med. 1963;35:1737-46. PubMed PMID: 14095886.	Excluded from the final analysis	The article was excluded from the analysis due to the lack of relation between the title and the abstract of the article with the subject under study
933	Bove A, Di Renzo RM, Palone G, D'Addetta V, Caldararo F, Antonopulos C, et al. Which differences do elderly patients present in single-stage treatment for cholecysto-choledocholithiasis? International Journal of Surgery. 2014;12:S160-S3. doi: 10.1016/j.ijsu.2014.08.358. PubMed PMID: WOS:000360991700036.	Excluded from the final analysis	The article was excluded from the analysis due to the lack of relation between the title and the abstract of the article with the subject under study
934	Ghassemian AJ, MacDonald KG, Cunningham PG, Swanson M, Brown BM, Morris PG, Pories WJ. The workup for bariatric surgery does not require a routine upper gastrointestinal series. Obes Surg. 1997;7(1):16-8. doi: 10.1381/096089297765556169. PubMed PMID: 9730531.	Excluded from the final analysis	The article was excluded from the analysis due to the lack of relation between the title and the abstract of the article with the subject under study
935	Alammari RK, Alhessan AA, Alturki AA, Aburowais SA, Alsharif MH, Alshehri FH, et al. Xanthogranulomatous Cholecystitis: A Rare Variant of Chronic Cholecystitis. Cureus. 2022;14(1):e21400. Epub 20220119. doi: 10.7759/cureus.21400. PubMed PMID: 35198307; PubMed Central PMCID: PMCPMC8856638.	Excluded from the final analysis	The article was excluded from the analysis due to the lack of relation between the title and the abstract of the article with the subject under study
936	Gadour E, Hassan Z, Hassan A. Y-Shaped Vesica Fellea Duplex Gallbladder Causing Acute Biliary Pancreatitis. Cureus Journal of Medical Science. 2021;13(4). doi: 10.7759/cureus.14676. PubMed PMID: WOS:000645290700006.	Excluded from the final analysis	The article was excluded from the analysis due to the lack of relation between the title and the abstract of the article with the subject under study

937	Mohr-Sasson A, Schiff E, Suday RR, Hayman Z, Kleinbaum Y, Kalter-Farber A, et al. The Yield of Abdominal Ultrasound in the Evaluation of Elevated Liver Enzymes during the Second and the Third Trimester of Pregnancy. Gynecol Obstet Invest. 2017;82(5):517-20. Epub 20170519. doi: 10.1159/000453612. PubMed PMID: 28521326.	Excluded from the final analysis	The article was excluded from the analysis due to the lack of relation between the title and the abstract of the article with the subject under study
938	Chen HY, Chang CJ, Yang YC, Lu FH, Sun ZJ, Wu JS. Renal Stones and Gallstones Correlated with the Ten-Year Risk Estimation of Atherosclerotic Cardiovascular Disease Based on the Pooled Cohort Risk Assessment of Males Aged 40-79. J Clin Med. 2023;12(6). Epub 20230316. doi: 10.3390/jcm12062309. PubMed PMID: 36983309; PubMed Central PMCID: PMCPMC10052154.	Excluded from the final analysis	The article was excluded from the analysis because it was repetitive (duplicate article)
939	Chae W, Lee HS, Jo JH, Chung MJ, Bang S, Park SW, et al. Impact of cholecystectomy on acute coronary syndrome according to metabolic condition: a nationwide population-based cohort study. Sci Rep. 2023;13(1):7300. Epub 20230505. doi: 10.1038/s41598-023-33440-4. PubMed PMID: 37147417; PubMed Central PMCID: PMCPMC10163235.	Excluded from the final analysis	The article was excluded from the analysis because it was repetitive (duplicate article)
940	Bai R, Wang J, Yang J, Cheng X, Zhang S, Zhang H, et al.!!!Gallbladder disease is associated with the risk of cardiovascular disease among Uyghurs in Xinjiang: a prospective cohort study. BMC Public Health. 2023;23(1):242. Epub 20230204. doi: 10.1186/s12889-023-15098-9. PubMed PMID: 36737734; PubMed Central PMCID: PMCPMC9898978.	Excluded from the final analysis	The article was excluded from the analysis because it was repetitive (duplicate article)
941	Park SM, Kim HJ, Kang TU, Swan H, Ahn HS. Cholecystectomy reduces the risk of myocardial and cerebral infarction in patients with gallstone-related infection. Sci Rep. 2022;12(1):16749. Epub 20221006. doi: 10.1038/s41598-022-20700-y. PubMed PMID: 36202881; PubMed Central PMCID: PMCPMC9537563.	Excluded from the final analysis	The article was excluded from the analysis because it was repetitive (duplicate article)
942	Ho TC, Chen YC, Lin CC, Tai HC, Wei CY, Yeh YH, Hsu CY. Reduced Risk of Atrial Fibrillation Following Cholecystectomy: A Nationwide Population-Based Study. Front Aging Neurosci. 2021;13:706815. Epub 20210902. doi: 10.3389/fnagi.2021.706815. PubMed PMID: 34539379; PubMed Central PMCID: PMCPMC8445074.	Excluded from the final analysis	The article was excluded from the analysis because it was repetitive (duplicate article)

943	Gill ES, Jeong YJ, Lee J. Association between gallstone disease and ischemic stroke in Korea. Neurology Asia. 2021;26(4).	Excluded from the final analysis	The article was excluded from the analysis because it was repetitive (duplicate article)
944	Chen CH, Lin CL, Kao CH. Risk of aortic dissection or aneurysm in patients with gallstone disease: a retrospective cohort study in Taiwan. BMJ Open. 2021;11(8):e049316. Epub 20210826. doi: 10.1136/bmjopen-2021-049316. PubMed PMID: 34446491; PubMed Central PMCID: PMCPMC8395267.	Excluded from the final analysis	The article was excluded from the analysis because it was repetitive (duplicate article)
945	Shabanzadeh DM, Skaaby T, Sørensen LT, Jørgensen T. Screen-detected gallstone disease and cardiovascular disease. Eur J Epidemiol. 2017;32(6):501-10. Epub 20170527. doi: 10.1007/s10654-017-0263-x. PubMed PMID: 28551778.	Excluded from the final analysis	The article was excluded from the analysis because it was repetitive (duplicate article)
946	Kwon CH, Kang JG, Lee HJ, Kim NH, Sung JW, Cheong E, Sung KC. ??Absence of association between gallstone and coronary artery calcification. Atherosclerosis. 2017;258:51-5. Epub 20170201. doi: 10.1016/j.atherosclerosis.2017.01.035. PubMed PMID: 28192729.	Excluded from the final analysis	The article was excluded from the analysis because it was repetitive (duplicate article)
947	Zheng Y, Xu M, Li Y, Hruby A, Rimm EB, Hu FB, et al. Gallstones and Risk of Coronary Heart Disease: Prospective Analysis of 270 000 Men and Women From 3 US Cohorts and Meta-Analysis. Arterioscler Thromb Vasc Biol. 2016;36(9):1997-2003. Epub 20160818. doi: 10.1161/atvbaha.116.307507. PubMed PMID: 27540264; PubMed Central PMCID: PMCPMC5001914.	Excluded from the final analysis	The article was excluded from the analysis because it was repetitive (duplicate article)
948	Wirth J, di Giuseppe R, Wientzek A, Katzke VA, Kloss M, Kaaks R, et al. Presence of gallstones and the risk of cardiovascular diseases: The EPIC-Germany cohort study. Eur J Prev Cardiol. 2015;22(3):326-34. Epub 20131031. doi: 10.1177/2047487313512218. PubMed PMID: 24177267.	Excluded from the final analysis	The article was excluded from the analysis because it was repetitive (duplicate article)
949	Lv J, Qi L, Yu C, Guo Y, Bian Z, Chen Y, et al. Gallstone Disease and the Risk of Ischemic Heart Disease. Arterioscler Thromb Vasc Biol. 2015;35(10):2232-7. Epub 20150813. doi: 10.1161/atvbaha.115.306043. PubMed PMID: 26272939; PubMed Central PMCID: PMCPMC4587542.	Excluded from the final analysis	The article was excluded from the analysis because it was repetitive (duplicate article)
950	Wei CY, Chung TC, Chen CH, Lin CC, Sung FC, Chung WT, et al. Gallstone disease and the risk of stroke: a nationwide population-based study. J Stroke Cerebrovasc Dis. 2014;23(7):1813-20. Epub 20140621. doi: 10.1016/j.jstrokecerebrovasdis.2014.04.024. PubMed PMID: 24957305.	Excluded from the final analysis	The article was excluded from the analysis because it was repetitive (duplicate article)

951	Olaiya MT, Chiou HY, Jeng JS, Lien LM, Hsieh FI. Significantly increased risk of cardiovascular disease among patients with gallstone disease: a population-based cohort study. PLoS One. 2013;8(10):e76448. Epub 20131003. doi: 10.1371/journal.pone.0076448. PubMed PMID: 24098504; PubMed Central PMCID: PMCPMC3789705.	Excluded from the final analysis	The article was excluded from the analysis because it was repetitive (duplicate article)
952	Jiang ZY, Sheng X, Xu CY, Li WW, Chang XX, Sun LY, et al. Gallbladder gallstone disease is associated with newly diagnosed coronary artery atherosclerotic disease: a cross-sectional study. PLoS One. 2013;8(9):e75400. Epub 20130918. doi: 10.1371/journal.pone.0075400. PubMed PMID: 24058685; PubMed Central PMCID: PMCPMC3776774.	Excluded from the final analysis	The article was excluded from the analysis because it was repetitive (duplicate article)
953	Khan HN, Harrison M, Bassett EE, Bates T. A 10-year follow-up of a longitudinal study of gallstone prevalence at necropsy in South East England. Dig Dis Sci. 2009;54(12):2736-41. doi: 10.1007/s10620-008-0682-3. PubMed PMID: 19160052.	Excluded from the final analysis	The article was excluded from the analysis because it was repetitive (duplicate article)
954	Méndez-Sánchez N, Zamora-Valdés D, Flores-Rangel JA, Pérez-Sosa JA, Vásquez-Fernández F, Lezama-Mora JI, et al. Gallstones are associated with carotid atherosclerosis. Liver Int. 2008;28(3):402-6. Epub 20071206. doi: 10.1111/j.1478-3231.2007.01632.x. PubMed PMID: 18069975.	Excluded from the final analysis	The article was excluded from the analysis because it was repetitive (duplicate article)
955	González-Pérez A, García Rodríguez LA. Gallbladder disease in the general population: association with cardiovascular morbidity and therapy. Pharmacoepidemiol Drug Saf. 2007;16(5):524-31. doi: 10.1002/pds.1346. PubMed PMID: 17103483.	Excluded from the final analysis	The article was excluded from the analysis because it was repetitive (duplicate article)
956	Méndez-Sánchez N, Bahena-Aponte J, Chávez-Tapia NC, Motola-Kuba D, Sánchez-Lara K, Ponciano-Radríguez G, et al. Strong association between gallstones and cardiovascular disease. Am J Gastroenterol. 2005;100(4):827-30. doi: 10.1111/j.1572-0241.2005.41214.x. PubMed PMID: 15784027.	Excluded from the final analysis	The article was excluded from the analysis because it was repetitive (duplicate article)
957	Bortnichak EA, Freeman DH, Jr., Ostfeld AM, Castelli WP, Kannel WB, Feinleib M, McNamara PM. The association between cholesterol cholelithiasis and coronary heart disease in Framingham, Massachusetts. Am J Epidemiol. 1985;121(1):19-30. doi: 10.1093/oxfordjournals.aje.a113978. PubMed PMID: 3155483.	Excluded from the final analysis	The article was excluded from the analysis because it was repetitive (duplicate article)
958	Chen HY, Chang CJ, Yang YC, Lu FH, Sun ZJ, Wu JS. Renal Stones and Gallstones Correlated with the Ten-Year Risk Estimation of Atherosclerotic Cardiovascular Disease Based on the Pooled Cohort Risk Assessment	Excluded from the final analysis	The article was excluded from the analysis because it was repetitive (duplicate article)

	of Males Aged 40-79. J Clin Med. 2023;12(6). Epub 20230316. doi: 10.3390/jcm12062309. PubMed PMID: 36983309; PubMed Central PMCID: PMCPMC10052154.		
959	Riordan JP, Stewart RJ, Pearce NE, Isbister WH. CHANGES IN THE PATTERN OF DISEASE MANAGED BY GENERAL SURGEONS IN NEW-ZEALAND, 1940-80. New Zealand Medical Journal. 1984;97(767):762-5. PubMed PMID: WOS:A1984TT32700002.	Excluded from the final analysis	The article was excluded from the analysis because it was repetitive (duplicate article)
960	Miltenburg DM, Schaffer R, Breslin T, Brandt ML. Changing indications for pediatric cholecystectomy. Pediatrics. 2000;105(6):1250-3. doi: 10.1542/peds.105.6.1250. PubMed PMID: WOS:000087441400026.	Excluded from the final analysis	The article was excluded from the analysis because it was repetitive (duplicate article)
961	Ros E, Armengol X, Grande L, ToledoPimentel V, Lacima G, Sanz G. Chest pain at rest in patients with coronary artery disease - Myocardial ischemia, esophageal dysfunction, or panic disorder? Digestive Diseases and Sciences. 1997;42(7):1344-53. doi: 10.1023/a:1018821417134. PubMed PMID: WOS:A1997XN21600002.	Excluded from the final analysis	The article was excluded from the analysis because it was repetitive (duplicate article)
962	Malkan GH, Bhatia SJ, Bashir K, Khemani R, Abraham P, Gandhi MS, Radhakrishnan R. Cholangiopathy associated with portal hypertension: diagnostic evaluation and clinical implications. Gastrointest Endosc. 1999;49(3 Pt 1):344-8. doi: 10.1016/s0016-5107(99)70011-8. PubMed PMID: 10049418.	Excluded from the final analysis	The article was excluded from the analysis because it was repetitive (duplicate article)
963	Malkan GH, Bhatia SJ, Bashir K, Khemani R, Abraham P, Gandhi MS, Radhakrishnan R. Cholangiopathy associated with portal hypertension: diagnostic evaluation and clinical implications. Gastrointestinal Endoscopy. 1999;49(3):344-8. doi: 10.1016/s0016-5107(99)70011-8. PubMed PMID: WOS:000079015000011.	Excluded from the final analysis	The article was excluded from the analysis because it was repetitive (duplicate article)
964	Park SM, Kim HJ, Kang TU, Swan H, Ahn HS. Cholecystectomy reduces the risk of myocardial and cerebral infarction in patients with gallstone-related infection. Scientific Reports. 2022;12(1). doi: 10.1038/s41598-022-20700-y. PubMed PMID: WOS:000864845400031.	Excluded from the final analysis	The article was excluded from the analysis because it was repetitive (duplicate article)
965	Nagappa M, Taly AB. Cholecysto-cardiac link: The heart of the matter. Neurology India. 2019;67(2):391-2. doi: 10.4103/0028-3886.258015. PubMed PMID: WOS:000468100900010.	Excluded from the final analysis	The article was excluded from the analysis because it was repetitive (duplicate article)

966	Harrison EC, Roschke EJ, Meyers HI, Edmiston WA, Chan LS, Tatter D, Lau FYK. CHOLELITHIASIS - FREQUENT COMPLICATION OF ARTIFICIAL HEART-VALVE REPLACEMENT. American Heart Journal. 1978;95(4):483-8. doi: 10.1016/0002-8703(78)90240-5. PubMed PMID: WOS:A1978ET44600010.	Excluded from the final analysis	The article was excluded from the analysis because it was repetitive (duplicate article)
967	Koller T, Kollerova J, Hlavaty T, Huorka M, Payer J. Cholelithiasis and markers of nonalcoholic fatty liver disease in patients with metabolic risk factors. Scand J Gastroenterol. 2012;47(2):197-203. Epub 20111219. doi: 10.3109/00365521.2011.643481. PubMed PMID: 22182015.	Excluded from the final analysis	The article was excluded from the analysis because it was repetitive (duplicate article)
968	Wang HH, Garruti G, Liu M, Portincasa P, Wang DQH. Cholesterol and Lipoprotein Metabolism and Atherosclerosis: Recent Advances in Reverse Cholesterol Transport. Annals of Hepatology. 2017;16:S27-S42. doi: 10.5604/01.3001.0010.5495. PubMed PMID: WOS:000460734300005.	Excluded from the final analysis	The article was excluded from the analysis because it was repetitive (duplicate article)
969	Wolff G, Liebscher C, Orban U. [Chronic gastritis and serum lipids, hypertension and cholelithiasis]. Dtsch Z Verdau Stoffwechselkr. 1988;48(3-4):149-52. PubMed PMID: 3234298.	Excluded from the final analysis	The article was excluded from the analysis because it was repetitive (duplicate article)
970	Finucci G, Tirelli M, Bellon S, Zambon M, Toffolo L, Merkel C, Zuin R. Clinical significance of cholelithiasis in patients with decompensated cirrhosis. J Clin Gastroenterol. 1990;12(5):538-41. doi: 10.1097/00004836-199010000-00010. PubMed PMID: 2229996.	Excluded from the final analysis	The article was excluded from the analysis because it was repetitive (duplicate article)
971	Li W, Tao J, Song XG, Hou MR, Qu K, Gu JT, et al. Clinical study of extrahepatic biliary adenoma. World Journal of Clinical Cases. 2023;11(24):5692-9. doi: 10.12998/wjcc.v11.i24.5692. PubMed PMID: WOS:001081844100007.	Excluded from the final analysis	The article was excluded from the analysis because it was repetitive (duplicate article)
972	Bateson MC, Maclean D, Ross PE, Bouchier IAD. CLOFIBRATE THERAPY AND GALLSTONE INDUCTION. American Journal of Digestive Diseases. 1978;23(7):623-8. doi: 10.1007/bf01072597. PubMed PMID: WOS:A1978FG18200008.	Excluded from the final analysis	The article was excluded from the analysis because it was repetitive (duplicate article)
973	Harooni J, Joukar F, Goujani R, Sikaroudi MK, Hatami A, Zolghadrpour MA, et al. Cohort profile: the PERSIAN Dena Cohort Study (PDCS) of non-communicable diseases in Southwest Iran. BMJ Open. 2024;14(4):e079697. Epub 20240410. doi: 10.1136/bmjopen-2023-079697. PubMed PMID: 38604628; PubMed Central PMCID: PMCPMC11015286.	Excluded from the final analysis	The article was excluded from the analysis because it was repetitive (duplicate article)

974	Gaitini D, Kreitenberg AJ, Fischer D, Maza I, Chowers Y. Color-coded duplex sonography compared to multidetector computed tomography for the diagnosis of crohn disease relapse and complications. J Ultrasound Med. 2011;30(12):1691-9. doi: 10.7863/jum.2011.30.12.1691. PubMed PMID: 22124005.	Excluded from the final analysis	The article was excluded from the analysis because it was repetitive (duplicate article)
975	Leppaniemi A, Wherry D, Pikoulis E, Hufnagel H, Waasdorp C, Fishback N, Rich N. Common bile duct repair with titanium staples - Comparison with suture closure. Surgical Endoscopy-Ultrasound and Interventional Techniques. 1997;11(7):714-7. doi: 10.1007/s004649900434. PubMed PMID: WOS:A1997XH52300004.	Excluded from the final analysis	The article was excluded from the analysis because it was repetitive (duplicate article)
976	Liang J, Jiang Y, Huang Y, Huang Y, Liu F, Zhang Y, et al. Comorbidities and factors influencing frequent gout attacks in patients with gout: a cross-sectional study. Clin Rheumatol. 2021;40(7):2873-80. Epub 20210204. doi: 10.1007/s10067-021-05595-w. PubMed PMID: 33538925.	Excluded from the final analysis	The article was excluded from the analysis because it was repetitive (duplicate article)
977	Sommerfleck F, Schneeberger E, Citera G. Comorbidities in Argentine patients with axial spondyloarthritis: Is nephrolithiasis associated with this disease? Eur J Rheumatol. 2018;5(3):169-72. Epub 20180622. doi: 10.5152/eurjrheum.2018.18002. PubMed PMID: 30071942; PubMed Central PMCID: PMCPMC6116842.	Excluded from the final analysis	The article was excluded from the analysis because it was repetitive (duplicate article)
978	Kaura SH, Haghighi M, Matza BW, Hajdu CH, Rosenkrantz AB. Comparison of CT and MRI findings in the differentiation of acute from chronic cholecystitis. Clin Imaging. 2013;37(4):687-91. Epub 20130328. doi: 10.1016/j.clinimag.2013.02.009. PubMed PMID: 23541278.	Excluded from the final analysis	The article was excluded from the analysis because it was repetitive (duplicate article)
979	Kulkarni SS, Hotta M, Sher L, Selby RR, Parekh D, Buxbaum J, Stapfer M. Complicated gallstone disease: diagnosis and management of Mirizzi syndrome. Surgical Endoscopy and Other Interventional Techniques. 2017;31(5):2215-22. doi: 10.1007/s00464-016-5219-9. PubMed PMID: WOS:000400552300021.	Excluded from the final analysis	The article was excluded from the analysis because it was repetitive (duplicate article)
980	Triantafyllidis I, Nikoloudis N, Sapidis N, Chrissidou M, Kalaitsidou I, Chrissidis T. Complications of Laparoscopic Cholecystectomy: Our Experience in a District General Hospital. Surgical Laparoscopy Endoscopy & Percutaneous Techniques. 2009;19(6):449-58. doi: 10.1097/SLE.0b013e3181bd8f6d. PubMed PMID: WOS:000273126800008.	Excluded from the final analysis	The article was excluded from the analysis because it was repetitive (duplicate article)
981	López R, Oyarzún M, Naranjo C, Cumsille F, Ortiz M, Baelum V. Coronary heart disease and periodontitis -: a case control study in Chilean adults. Journal of Clinical Periodontology. 2002;29(5):468-73. doi: 10.1034/j.1600-051X.2002.290513.x. PubMed PMID: WOS:000176702100013.	Excluded from the final analysis	The article was excluded from the analysis because it was repetitive (duplicate article)

982	Akoudad S, Szklo M, McAdams MA, Fulop T, Anderson CA, Coresh J, Köttgen A. Correlates of kidney stone disease differ by race in a multi-ethnic middle-aged population: the ARIC study. Prev Med. 2010;51(5):416-20. Epub 20100827. doi: 10.1016/j.ypmed.2010.08.011. PubMed PMID: 20801154; PubMed Central PMCID: PMCPMC2964449.	Excluded from the final analysis	The article was excluded from the analysis because it was repetitive (duplicate article)
983	Mun EC, Blackburn GL, Matthews JB. Current status of medical and surgical therapy for obesity. Gastroenterology. 2001;120(3):669-81. doi: 10.1053/gast.2001.22430. PubMed PMID: 11179243.	Excluded from the final analysis	The article was excluded from the analysis because it was repetitive (duplicate article)
984	Tiseo D, Borrelli F, Gentile I, Benassai G, Quarto G, Borgia G. [Cystic echinococcosis in humans: our clinic experience]. Parassitologia. 2004;46(1-2):45-51. PubMed PMID: 15305685.	Excluded from the final analysis	The article was excluded from the analysis because it was repetitive (duplicate article)
985	Li D, Li Y, Wang X, Wu Y, Cui XY, Hu JQ, et al. Diagnosis of myocardial infarction with nonobstructive coronary arteries in a young man in the setting of acute myocardial infarction after endoscopic retrograde cholangiopancreatography: A case report. World Journal of Clinical Cases. 2019;7(19):3062-8. doi: 10.12998/wjcc.v7.i19.3062. PubMed PMID: WOS:000488264700016.	Excluded from the final analysis	The article was excluded from the analysis because it was repetitive (duplicate article)
986	Elkerkary MA, Gbr H, Shaban H. Diagnostic accuracy of intraoperative cholangiography for detection of anatomical variations of the biliary system. Egyptian Journal of Surgery. 2021;40(1):131-9. doi: 10.4103/ejs.ejs_266_20. PubMed PMID: WOS:000656200800017.	Excluded from the final analysis	The article was excluded from the analysis because it was repetitive (duplicate article)
987	Debry G. [Diet peculiarities. Vegetarianism, veganism, crudivorism, macrobiotism]. Rev Prat. 1991;41(11):967-72. PubMed PMID: 2063109.	Excluded from the final analysis	The article was excluded from the analysis because it was repetitive (duplicate article)
988	Pawlikowska L, Strautnieks S, Jankowska I, Czubkowski P, Emerick K, Antoniou A, et al. Differences in presentation and progression between severe FIC1 and BSEP deficiencies. J Hepatol. 2010;53(1):170-8. Epub 20100413. doi: 10.1016/j.jhep.2010.01.034. PubMed PMID: 20447715; PubMed Central PMCID: PMCPMC3042805.	Excluded from the final analysis	The article was excluded from the analysis because it was repetitive (duplicate article)

989	Malik AM. Difficult laparoscopic cholecystectomies. Is conversion a sensible option? J Pak Med Assoc. 2015;65(7):698-700. PubMed PMID: 26160075.	Excluded from the final analysis	The article was excluded from the analysis because it was repetitive (duplicate article)
990	Smith FC, Gwynn BR. Direct access surgery. Ann R Coll Surg Engl. 1995;77(2):94-6. PubMed PMID: 7793823; PubMed Central PMCID: PMCPMC2502149.	Excluded from the final analysis	The article was excluded from the analysis because it was repetitive (duplicate article)
991	Martin S, Tyrrell J, Thomas EL, Bown MJ, Wood AR, Beaumont RN, et al. Disease consequences of higher adiposity uncoupled from its adverse metabolic effects using Mendelian randomisation. Elife. 2022;11. Epub 20220125. doi: 10.7554/eLife.72452. PubMed PMID: 35074047; PubMed Central PMCID: PMCPMC8789289.	Excluded from the final analysis	The article was excluded from the analysis because it was repetitive (duplicate article)
992	Kim BH, Lee SH, Lee SS, Oh DJ, Chung ES, Lee SJ. Diverticular disease of the colon in Korea. Korean J Intern Med. 1987;2(1):79-83. doi: 10.3904/kjim.1987.2.1.79. PubMed PMID: 3154819; PubMed Central PMCID: PMCPMC4534912.	Excluded from the final analysis	The article was excluded from the analysis because it was repetitive (duplicate article)
993	Starkov Iu G, Strekalovskiĭ VP, Vishnevskiĭ VA, Grigor'ian RS. [Diverticuli of duodenal papillar region and their role in development of choledocholithiasis and strictures of bile and pancreatic ducts]. Khirurgiia (Mosk). 2000;(3):10-3. PubMed PMID: 10761374.	Excluded from the final analysis	The article was excluded from the analysis because it was repetitive (duplicate article)
994	Maddu K, Polireddy K, Hsu DR, Hoff C. Do not get stumped: multimodality imaging findings of early and late post-cholecystectomy complications. Emergency Radiology. 2023;30(3):351-62. doi: 10.1007/s10140-023-02131-y. PubMed PMID: WOS:000968250000002.	Excluded from the final analysis	The article was excluded from the analysis because it was repetitive (duplicate article)
995	Sher PP. Drug interference with laboratory tests: oral contraceptives. Drug Ther (NY). 1977;2(10):61-3. PubMed PMID: 12260155.	Excluded from the final analysis	The article was excluded from the analysis because it was repetitive (duplicate article)
996	Cuschieri A, Lezoche E, Morino M, Croce E, Lacy A, Toouli J, et al. EAES multicenter prospective randomized trial comparing two-stage vs single-stage management of patients with gallstone disease and ductal calculi. Surgical Endoscopy-Ultrasound and Interventional Techniques. 1999;13(10):952-7. doi: 10.1007/s004649901145. PubMed PMID: WOS:000082887100001.	Excluded from the final analysis	The article was excluded from the analysis because it was repetitive (duplicate article)

997	Hirano T, Manabe T. EFFECT OF SHORT-TERM PANCREATICOBILIARY DUCT OBSTRUCTION WITH INTRADUCTAL HYPERTENSION ON SUBCELLULAR ORGANELLE FRAGILITY AND PANCREATIC ADENYLATE ENERGY-METABOLISM IN RATS - PROTECTIVE EFFECT OF A NEW PROTEASE INHIBITOR, E-3123. Acta Chirurgica Belgica. 1994;(2):80-5. PubMed PMID: WOS:A1994NM78600004.	Excluded from the final analysis	The article was excluded from the analysis because it was repetitive (duplicate article)
998	Skaaby T, Jorgensen T, Linneberg A. Effects of invitation to participate in health surveys on the incidence of cardiovascular disease: a randomized general population study. International Journal of Epidemiology. 2017;46(2):603-11. doi: 10.1093/ije/dyw311. PubMed PMID: WOS:000402996000049.	Excluded from the final analysis	The article was excluded from the analysis because it was repetitive (duplicate article)
999	Stewart L, Grifiss JM, Jarvis GA, Way LW. Elderly patients have more severe biliary infections: influence of complement-killing and induction of TNFalpha production. Surgery. 2008;143(1):103-12. Epub 20071203. doi: 10.1016/j.surg.2007.06.035. PubMed PMID: 18154938.	Excluded from the final analysis	The article was excluded from the analysis because it was repetitive (duplicate article)
1000	Martín-Pérez J, Delgado-Plasencia L, Bravo-Gutiérrez A, Lorenzo-Rocha N, Burillo-Putze G, Medina-Arana V. Enterolithotomy and early cholecystectomy, an application of damage control surgery for patients with gallstone ileus. Cirugia Y Cirujanos. 2015;83(2):156-60. doi: 10.1016/j.circir.2015.04.013. PubMed PMID: WOS:000362409600013.	Excluded from the final analysis	The article was excluded from the analysis because it was repetitive (duplicate article)
1001	Fang JG, Zhu J, Li XJ, Li R, Dai F, Song XM, et al. [Epidemiological survey of prevalence of fatty liver and its risk factors in a general adult population of Shanghai]. Zhonghua Gan Zang Bing Za Zhi. 2005;13(2):83-8. PubMed PMID: 15727689.	Excluded from the final analysis	The article was excluded from the analysis because it was repetitive (duplicate article)
1002	Efremova I, Maslennikov R, Poluektova E, Vasilieva E, Zharikov Y, Suslov A, et al. Epidemiology of small intestinal bacterial overgrowth. World J Gastroenterol. 2023;29(22):3400-21. doi: 10.3748/wjg.v29.i22.3400. PubMed PMID: 37389240; PubMed Central PMCID: PMCPMC10303511.	Excluded from the final analysis	The article was excluded from the analysis because it was repetitive (duplicate article)
1003	Tomassetti P, Migliori M, Lalli S, Campana D, Tomassetti V, Corinaldesi R. Epidemiology, clinical features and diagnosis of gastroenteropancreatic endocrine tumours. Annals of Oncology. 2001;12:S95-S9. doi: 10.1093/annonc/12.suppl_2.S95. PubMed PMID: WOS:000172485900017.	Excluded from the final analysis	The article was excluded from the analysis because it was repetitive (duplicate article)
1004	Eliakim R, Abulafia O, Sherer DM, Rayburn WF. Estrogen, progesterone and the gastrointestinal tract. Journal of Reproductive Medicine. 2000;45(10):781-8. PubMed PMID: WOS:000090105300001.	Excluded from the final analysis	The article was excluded from the analysis because it was repetitive (duplicate article)

1005	Lans CA. Ethnomedicines used in Trinidad and Tobago for urinary problems and diabetes mellitus. J Ethnobiol Ethnomed. 2006;2:45. Epub 20061013. doi: 10.1186/1746-4269-2-45. PubMed PMID: 17040567; PubMed Central PMCID: PMCPMC1624823.	Excluded from the final analysis	The article was excluded from the analysis because it was repetitive (duplicate article)
1006	Sakorafas GH, Tsiotou AG. Etiology and pathogenesis of acute pancreatitis - Current concepts. Journal of Clinical Gastroenterology. 2000;30(4):343-56. doi: 10.1097/00004836-200006000-00002. PubMed PMID: WOS:000087387200002.	Excluded from the final analysis	The article was excluded from the analysis because it was repetitive (duplicate article)
1007	Thapar VB, Thapar PM, Goel R, Agarwalla R, Salvi PH, Nasta AM, et al. Evaluation of 30-day morbidity and mortality of laparoscopic cholecystectomy: a multicenter prospective observational Indian Association of Gastrointestinal Endoscopic Surgeons (IAGES) Study. Surgical Endoscopy and Other Interventional Techniques. 2023;37(4):2611-25. doi: 10.1007/s00464-022-09659-z. PubMed PMID: WOS:000882821900001.	Excluded from the final analysis	The article was excluded from the analysis because it was repetitive (duplicate article)
1008	Utter A, Goss F. Exercise and gall bladder function. Sports Medicine. 1997;23(4):218-27. doi: 10.2165/00007256-199723040-00002. PubMed PMID: WOS:A1997WZ53500002.	Excluded from the final analysis	The article was excluded from the analysis because it was repetitive (duplicate article)
1009	Williams LR, Flinn WR, Yao JST, Vogelzang RL, Roth M, McCarthy WJ, Bergan JJ. EXTENDED USE OF COMPUTED-TOMOGRAPHY IN THE MANAGEMENT OF COMPLEX AORTIC PROBLEMS - A LEARNING-EXPERIENCE. Journal of Vascular Surgery. 1986;4(3):264-71. doi: 10.1067/mva.1986.avs0040264. PubMed PMID: WOS:A1986D927500011.	Excluded from the final analysis	The article was excluded from the analysis because it was repetitive (duplicate article)
1010	Ragheb S, Choong CK, Gowland S, Bagshaw PF, Frizelle FA. Extracorporeal shock wave lithotripsy for difficult common bile duct stones: initial New Zealand experience. New Zealand Medical Journal. 2000;113(1117):377-8. PubMed PMID: WOS:000089371100005.	Excluded from the final analysis	The article was excluded from the analysis because it was repetitive (duplicate article)
1011	Yassin MA, Soliman AT, De Sanctis V, Yassin KS, Abdulla MAJ. Final Height and Endocrine Complications in Patients with β-Thalassemia Intermedia: Our Experience in Non-Transfused Versus Infrequently Transfused Patients and Correlations with Liver Iron Content. Mediterranean Journal of Hematology and Infectious Diseases. 2019;11. doi: 10.4084/mjhid.2019.026. PubMed PMID: WOS:000466715800002.	Excluded from the final analysis	The article was excluded from the analysis because it was repetitive (duplicate article)

1012	Trucco G, Chiusa L, Tandoi F, Bertero L. First report of a gallbladder hemangioma coexisting with gallstones: a case report and literature review of a rare finding. BMC Surg. 2022;22(1):128. Epub 20220406. doi: 10.1186/s12893-022-01554-7. PubMed PMID: 35382806; PubMed Central PMCID: PMCPMC8985283.	Excluded from the final analysis	The article was excluded from the analysis because it was repetitive (duplicate article)
1013	Wang L, Zhang Z, Wang Z, Jiang T. First study on the outcomes of biliopancreatic diversion with duodenal switch in Chinese patients with obesity. Front Surg. 2022;9:934434. Epub 20230106. doi: 10.3389/fsurg.2022.934434. PubMed PMID: 36684353; PubMed Central PMCID: PMCPMC9852535.	Excluded from the final analysis	The article was excluded from the analysis because it was repetitive (duplicate article)
1014	Cavigelli A, Dietz V. Follow-up care of para-/tetraplegics: selected aspects in internal medicine. Schweizerische Medizinische Wochenschrift. 2000;130(22):851-60. PubMed PMID: WOS:000087885200007.	Excluded from the final analysis	The article was excluded from the analysis because it was repetitive (duplicate article)
1015	Pape J, Swart O, Duvenage R. Four-year review of admissions to a South African regional hospital general surgery department. Samj South African Medical Journal. 2019;109(2):122-6. doi: 10.7196/SAMJ.2019.v109i2.13433. PubMed PMID: WOS:000457578200018.	Excluded from the final analysis	The article was excluded from the analysis because it was repetitive (duplicate article)
1016	Szilvási A, Andrikovics H, Pongracz E, Kalina A, Komlosi Z, Klein I, Tordai A. Frequencies of Four ATP-Binding Cassette Transporter G8 Polymorphisms in Patients with Ischemic Vascular Diseases. Genetic Testing and Molecular Biomarkers. 2010;14(5):667-72. doi: 10.1089/gtmb.2010.0035. PubMed PMID: WOS:000283125400014.	Excluded from the final analysis	The article was excluded from the analysis because it was repetitive (duplicate article)
1017	Chawla A, Dewan R, Sarin SK. The frequency and influence of gallbladder varices on gallbladder functions in patients with portal hypertension. Am J Gastroenterol. 1995;90(11):2010-4. PubMed PMID: 7485012.	Excluded from the final analysis	The article was excluded from the analysis because it was repetitive (duplicate article)
1018	Barker DJP, Gardner MJ, Power C. GALL-STONES AND ISCHEMIC-HEART-DISEASE. British Medical Journal. 1980;280(6215):717-. doi: 10.1136/bmj.280.6215.717. PubMed PMID: WOS:A1980JH94900038.	Excluded from the final analysis	The article was excluded from the analysis because it was repetitive (duplicate article)
1019	Zhu JF. [Gallbladder contractile function in patients with portal hypertension]. Zhonghua Wai Ke Za Zhi. 1993;31(1):34-6. PubMed PMID: 8404340.	Excluded from the final analysis	The article was excluded from the analysis because it was repetitive (duplicate article)

1020	Rajvanshi P, Atac BS, Seno R, Gupta S. Gallbladder vasculitis associated with type-1 cryoglobulinemia. Dig Dis Sci. 2001;46(2):296-300. doi: 10.1023/a:1005648715013. PubMed PMID: 11281178.	Excluded from the final analysis	The article was excluded from the analysis because it was repetitive (duplicate article)
1021	Wu CY, Su CC, Huang HH, Wang YT, Wang CC. Gallstone associated celiac trunk thromboembolisms complicated with splenic infarction: A case report. World Journal of Clinical Cases. 2022;10(25):8968-73. doi: 10.12998/wjcc.v10.i25.8968. PubMed PMID: WOS:000866214000018.	Excluded from the final analysis	The article was excluded from the analysis because it was repetitive (duplicate article)
1022	Georgescu D, Ionita I, Lascu A, Hut EF, Dragan S, Ancusa OE, et al. Gallstone Disease and Bacterial Metabolic Performance of Gut Microbiota in Middle-Aged and Older Patients. Int J Gen Med. 2022;15:5513-31. Epub 20220608. doi: 10.2147/ijgm.S350104. PubMed PMID: 35702368; PubMed Central PMCID: PMCPMC9188808.	Excluded from the final analysis	The article was excluded from the analysis because it was repetitive (duplicate article)
1023	Zheng Y, Xu M, Heianza Y, Ma WJ, Wang TG, Sun DJY, et al. Gallstone disease and increased risk of mortality: Two large prospective studies in US men and women. Journal of Gastroenterology and Hepatology. 2018;33(11):1925-31. doi: 10.1111/jgh.14264. PubMed PMID: WOS:000447150900020.	Excluded from the final analysis	The article was excluded from the analysis because it was repetitive (duplicate article)
1024	Upala S, Sanguankeo A, Jaruvongvanich V. GALLSTONE DISEASE AND THE RISK OF CARDIOVASCULAR DISEASE: A SYSTEMATIC REVIEW AND META-ANALYSIS OF OBSERVATIONAL STUDIES. Scandinavian Journal of Surgery. 2017;106(1):21-7. doi: 10.1177/1457496916650998. PubMed PMID: WOS:000398818200002.	Excluded from the final analysis	The article was excluded from the analysis because it was repetitive (duplicate article)
1025	Wang JY, Lu FH, Sun ZJ, Wu JS, Yang YC, Lee CT, Chang CJ. Gallstone disease associated with increased risk of arterial stiffness in a Taiwanese population. J Hum Hypertens. 2017;31(10):616-9. Epub 20170629. doi: 10.1038/jhh.2017.43. PubMed PMID: 28660886.	Excluded from the final analysis	The article was excluded from the analysis because it was repetitive (duplicate article)
1026	Mallick B, Anand AC. Gallstone Disease in Cirrhosis-Pathogenesis and Management. J Clin Exp Hepatol. 2022;12(2):551-9. Epub 20210916. doi: 10.1016/j.jceh.2021.09.011. PubMed PMID: 35535063; PubMed Central PMCID: PMCPMC9077239.	Excluded from the final analysis	The article was excluded from the analysis because it was repetitive (duplicate article)
1027	Wildi S, Reber PU, Baer HU. Gallstone disease masking malignant bile duct tumors: A rare but important coincidence. Digestive Surgery. 2000;17(2):174-8. doi: 10.1159/000018824. PubMed PMID: WOS:000086783300017.	Excluded from the final analysis	The article was excluded from the analysis because it was repetitive (duplicate article)

1028	Eto T, Tsuchiya R, Harada N, Tsunoda T, Yamamoto K. GALLSTONE FORMATION IN DOGS BY SELECTIVE OCCLUSION OF PORTAL-VEIN BRANCHES. Digestive Diseases and Sciences. 1986;31(10):S373-S. PubMed PMID: WOS:A1986E217001481.	Excluded from the final analysis	The article was excluded from the analysis because it was repetitive (duplicate article)
1029	Gasparrini M, Liverani A, Catracchia V, Conte S, Leonardo G, Marino G, et al. Gallstone ileus: a case report and review of the literature. Chir Ital. 2008;60(5):755-9. PubMed PMID: 19062503.	Excluded from the final analysis	The article was excluded from the analysis because it was repetitive (duplicate article)
1030	Wang H, So H, Ko SW, Jung SW, Bang SJ, Park EJ. Gallstone Is Associated with Metabolic Factors and Exercise in Korea. Healthcare (Basel). 2022;10(8). Epub 20220724. doi: 10.3390/healthcare10081372. PubMed PMID: 35893194; PubMed Central PMCID: PMCPMC9329956.	Excluded from the final analysis	The article was excluded from the analysis because it was repetitive (duplicate article)
1031	Jeong YH, Kim KO, Lee HC, Sohn SH, Lee JW, Lee SH, et al. Gallstone prevalence and risk factors in patients with ulcerative colitis in Korean population. Medicine (Baltimore). 2017;96(31):e7653. doi: 10.1097/md.0000000000007653. PubMed PMID: 28767582; PubMed Central PMCID: PMCPMC5626136.	Excluded from the final analysis	The article was excluded from the analysis because it was repetitive (duplicate article)
1032	Zheng Y, Xu M, Li YP, Hruby A, Rimm EB, Hu FB, et al. Gallstones and Risk of Coronary Heart Disease: Prospective Analysis of 270000 Men and Women From 3 US Cohorts and Meta-Analysis. Arteriosclerosis Thrombosis and Vascular Biology. 2016;36(9):1997-2003. doi: 10.1161/atvbaha.116.307507. PubMed PMID: WOS:000383582900036.	Excluded from the final analysis	The article was excluded from the analysis because it was repetitive (duplicate article)
1033	Hunt DRH, Chu FCK. Gangrenous cholecystitis in the laparoscopic era. Australian and New Zealand Journal of Surgery. 2000;70(6):428-30. doi: 10.1046/j.1440-1622.2000.01851.x. PubMed PMID: WOS:000088312800008.	Excluded from the final analysis	The article was excluded from the analysis because it was repetitive (duplicate article)
1034	Wight CO, Seed M, Yeo WW, McCulloch TA. Gastric outflow obstruction caused by gall stones and leading to death by complex metabolic derangement. Journal of Clinical Pathology. 1997;50(11):963-5. doi: 10.1136/jcp.50.11.963. PubMed PMID: WOS:A1997YK17300019.	Excluded from the final analysis	The article was excluded from the analysis because it was repetitive (duplicate article)
1035	Ebert E. Gastrointestinal involvement in spinal cord injury: a clinical perspective. J Gastrointestin Liver Dis. 2012;21(1):75-82. PubMed PMID: 22457863.	Excluded from the final analysis	The article was excluded from the analysis because it was repetitive (duplicate article)

1036	Hughes DA, Pastores GM. Gaucher Disease. In: Adam MP, Feldman J, Mirzaa GM, Pagon RA, Wallace SE, Amemiya A, editors. GeneReviews(®). Seattle (WA): University of Washington, Seattle	Excluded from the final analysis	The article was excluded from the analysis because it was repetitive (duplicate article)
1037	Puppala S, Dodd GD, Fowler S, Arya R, Schneider J, Farook VS, et al. A genomewide search finds major susceptibility loci for gallbladder disease on chromosome 1 in Mexican Americans. Am J Hum Genet. 2006;78(3):377-92. Epub 20060106. doi: 10.1086/500274. PubMed PMID: 16400619; PubMed Central PMCID: PMCPMC1380282.	Excluded from the final analysis	The article was excluded from the analysis because it was repetitive (duplicate article)
1038	Key TJ, Davey GK, Appleby PN. Health benefits of a vegetarian diet. Proceedings of the Nutrition Society. 1999;58(2):271-5. doi: 10.1017/s0029665199000373. PubMed PMID: WOS:000081952800009.	Excluded from the final analysis	The article was excluded from the analysis because it was repetitive (duplicate article)
1039	Ros E. Health benefits of nut consumption. Nutrients. 2010;2(7):652-82. Epub 20100624. doi: 10.3390/nu2070652. PubMed PMID: 22254047; PubMed Central PMCID: PMCPMC3257681.	Excluded from the final analysis	The article was excluded from the analysis because it was repetitive (duplicate article)
1040	Swinburn B, Ashton T, Gillespie J, Cox B, Menon A, Simmons D, Birkbeck J. Health care costs of obesity in New Zealand. Int J Obes Relat Metab Disord. 1997;21(10):891-6. doi: 10.1038/sj.ijo.0800486. PubMed PMID: 9347407.	Excluded from the final analysis	The article was excluded from the analysis because it was repetitive (duplicate article)
1041	Welty TK. Health implications of obesity in American Indians and Alaska Natives. Am J Clin Nutr. 1991;53(6 Suppl):1616s-20s. doi: 10.1093/ajcn/53.6.1616S. PubMed PMID: 2031495.	Excluded from the final analysis	The article was excluded from the analysis because it was repetitive (duplicate article)
1042	Welty TK. HEALTH IMPLICATIONS OF OBESITY IN AMERICAN-INDIANS AND ALASKA NATIVES. American Journal of Clinical Nutrition. 1991;53(6):S1616-S20. doi: 10.1093/ajcn/53.6.1616S. PubMed PMID: WOS:A1991FP05900019.	Excluded from the final analysis	The article was excluded from the analysis because it was repetitive (duplicate article)
1043	Hainer V, Kunesová M, Parízková J, Stunkard A. [Health risks and economic costs associated with obesity requiring a comprehensive weight reduction program]. Cas Lek Cesk. 1997;136(12):367-72. PubMed PMID: 9333508.	Excluded from the final analysis	The article was excluded from the analysis because it was repetitive (duplicate article)

1044	Mikami T, Hirata K, Oikawa I, Kimura M, Kimura H. Hemobilia caused by a giant benign hemangioma of the liver: Report of a case. Surgery Today-the Japanese Journal of Surgery. 1998;28(9):948-52. doi: 10.1007/s005950050259. PubMed PMID: WOS:000075757200015.	Excluded from the final analysis	The article was excluded from the analysis because it was repetitive (duplicate article)
1045	Czerniak A, Thompson JN, Hemingway AP, Soreide O, Benjamin IS, Allison DJ, Blumgart LH. Hemobilia. A disease in evolution. Arch Surg. 1988;123(6):718-21. doi: 10.1001/archsurg.1988.01400300064010. PubMed PMID: 3369935.	Excluded from the final analysis	The article was excluded from the analysis because it was repetitive (duplicate article)
1046	Walton JM, Abraham RJ, Perey BJ, MacGregor JH, Campbell DR. Hepatic artery pseudoaneurysms in acute pancreatitis. Can J Surg. 1991;34(4):377-80. PubMed PMID: 1868396.	Excluded from the final analysis	The article was excluded from the analysis because it was repetitive (duplicate article)
1047	Mitchell E, Ranganathan S, McKiernan P, Squires RH, Strauss K, Soltys K, et al. Hepatic Parenchymal Injury in Crigler-Najjar Type I. J Pediatr Gastroenterol Nutr. 2018;66(4):588-94. doi: 10.1097/mpg.0000000000001843. PubMed PMID: 29176474.	Excluded from the final analysis	The article was excluded from the analysis because it was repetitive (duplicate article)
1048	Xiao JL, Ng CH, Chan KE, Fu C, Tay P, Yong JN, et al. Hepatic, Extra-hepatic Outcomes and Causes of Mortality in NAFLD-An Umbrella Overview of Systematic Review of Meta-Analysis. Journal of Clinical and Experimental Hepatology. 2023;13(4):656-65. doi: 10.1016/j.jceh.2022.11.006. PubMed PMID: WOS:001035247400001.	Excluded from the final analysis	The article was excluded from the analysis because it was repetitive (duplicate article)
1049	Pitt HA. Hepatobiliary Hands of Hopkins. Ann Surg. 2018;267(2S Suppl 2):S34-s9. doi: 10.1097/sla.0000000000002606. PubMed PMID: 29206676.	Excluded from the final analysis	The article was excluded from the analysis because it was repetitive (duplicate article)
1050	Ball NJ, Duggan MA. HEPATOLITHIASIS IN HEREDITARY HEMORRHAGIC TELANGIECTASIA. Archives of Pathology & Laboratory Medicine. 1990;114(4):423-5. PubMed PMID: WOS:A1990CX41300021.	Excluded from the final analysis	The article was excluded from the analysis because it was repetitive (duplicate article)
1051	Mendoza A, Oliff S, Elias E. Hereditary haemorrhagic telangiectasia and secondary biliary cirrhosis. Eur J Gastroenterol Hepatol. 1995;7(10):999-1002. doi: 10.1097/00042737-199510000-00017. PubMed PMID: 8590149.	Excluded from the final analysis	The article was excluded from the analysis because it was repetitive (duplicate article)

1052	Young SB, Arregui M, Singh K. HIDA scan ejection fraction does not predict sphincter of Oddi hypertension or clinical outcome in patients with suspected chronic acalculous cholecystitis. Surg Endosc. 2006;20(12):1872-8. doi: 10.1007/s00464-005-0245-z. PubMed PMID: 17031746.	Excluded from the final analysis	The article was excluded from the analysis because it was repetitive (duplicate article)
1053	Zeng D, Wu H, Huang Q, Zeng A, Yu Z, Zhong Z. High Levels of Serum Triglyceride, Low-density Lipoprotein Cholesterol, Total Bile Acid, and Total Bilirubin are Risk Factors for Gallstones. Clin Lab. 2021;67(8). doi: 10.7754/Clin.Lab.2021.201228. PubMed PMID: 34383399.	Excluded from the final analysis	The article was excluded from the analysis because it was repetitive (duplicate article)
1054	Shaka H, Asotibe JC, Achebe I, Pudasaini G. Higher Inpatient Morbidity and Mortality in Biliary Pancreatitis Compared to Hypertriglyceridemia-Induced Pancreatitis: A Nationwide Retrospective Study. Cureus Journal of Medical Science. 2020;12(9). doi: 10.7759/cureus.10351. PubMed PMID: WOS:000567771300005.	Excluded from the final analysis	The article was excluded from the analysis because it was repetitive (duplicate article)
1055	Pullingr CR, Eng C, Salen G, Shefer S, Batta AK, Erickson SK, et al. Human cholesterol 7α-hydroxylase (CYP7A1) deficiency has a hypercholesterolemic phenotype. Journal of Clinical Investigation. 2002;110(1):109-17. doi: 10.1172/jci200215387. PubMed PMID: WOS:000176665100015.	Excluded from the final analysis	The article was excluded from the analysis because it was repetitive (duplicate article)
1056	Manz F. Hydration and disease. Journal of the American College of Nutrition. 2007;26(5):535S-41S. doi: 10.1080/07315724.2007.10719655. PubMed PMID: WOS:000250506300005.	Excluded from the final analysis	The article was excluded from the analysis because it was repetitive (duplicate article)
1057	Liesemer K, Mullen N. Hypertensive emergency successfully treated with metoprolol: a case report. Pediatr Emerg Care. 2009;25(5):333-5. doi: 10.1097/PEC.0b013e3181a34816. PubMed PMID: 19444030.	Excluded from the final analysis	The article was excluded from the analysis because it was repetitive (duplicate article)
1058	Zobel MJ, Stewart L. Hyponatremia is associated with more severe biliary disease. World J Gastrointest Surg. 2020;12(2):45-54. doi: 10.4240/wjgs.v12.i2.45. PubMed PMID: 32128028; PubMed Central PMCID: PMCPMC7044107.	Excluded from the final analysis	The article was excluded from the analysis because it was repetitive (duplicate article)
1059	Verit A, Güner ND. <i>Helicobacter pylori</i> and urinary system stones: Endoluminal damage as sub-hypothesis to support the current stone theory. Medical Hypotheses. 2014;83(6):677-80. doi: 10.1016/j.mehy.2014.09.016. PubMed PMID: WOS:000347595600010.	Excluded from the final analysis	The article was excluded from the analysis because it was repetitive (duplicate article)

1060	Panwar U, Singh SK. Identification of Novel Pancreatic Lipase Inhibitors Using In Silico Studies. Endocr Metab Immune Disord Drug Targets. 2019;19(4):449-57. doi: 10.2174/1871530319666181128100903. PubMed PMID: 30484411.	Excluded from the final analysis	The article was excluded from the analysis because it was repetitive (duplicate article)
1061	Grimaldi CH, Nelson RG, Pettitt DJ, Sampliner RE, Bennett PH, Knowler WC. INCREASED MORTALITY WITH GALLSTONE DISEASE - RESULTS OF A 20-YEAR POPULATION-BASED SURVEY IN PIMA-INDIANS. Annals of Internal Medicine. 1993;118(3):185-90. doi: 10.7326/0003-4819-118-3-199302010-00005. PubMed PMID: WOS:A1993KJ43900005.	Excluded from the final analysis	The article was excluded from the analysis because it was repetitive (duplicate article)
1062	Kim SY, Lim H, Park B, Lim H, Kim M, Kong IG, Choi HG. Increased risk of gallstones after appendectomy: A longitudinal follow-up study using a national sample cohort. Medicine (Baltimore). 2020;99(20):e20269. doi: 10.1097/md.0000000000020269. PubMed PMID: 32443372; PubMed Central PMCID: PMCPMC7253851.	Excluded from the final analysis	The article was excluded from the analysis because it was repetitive (duplicate article)
1063	Al-Salem AH. Indications and complications of splenectomy for children with sickle cell disease. J Pediatr Surg. 2006;41(11):1909-15. doi: 10.1016/j.jpedsurg.2006.06.020. PubMed PMID: 17101369.	Excluded from the final analysis	The article was excluded from the analysis because it was repetitive (duplicate article)
1064	Tang K, Ford B, Grasso SL, Swisher J. Infectious aortitis and managing it at a community military hospital. BMJ Case Rep. 2024;17(3). Epub 20240327. doi: 10.1136/bcr-2023-257509. PubMed PMID: 38538095; PubMed Central PMCID: PMCPMC10982747.	Excluded from the final analysis	The article was excluded from the analysis because it was repetitive (duplicate article)
1065	Kasana V, Rajesh S, Chauhan U, Bihari C, Choudhury A, Sarin SK. Inflammatory Myofibroblastic Tumor of Liver Masquerading as Focal Nodular Hyperplasia in a Patient with Non-Cirrhotic Portal Hypertension and Biliary Pancreatitis. Indian J Surg Oncol. 2016;7(1):110-4. Epub 20150220. doi: 10.1007/s13193-015-0381-4. PubMed PMID: 27065695; PubMed Central PMCID: PMCPMC4811821.	Excluded from the final analysis	The article was excluded from the analysis because it was repetitive (duplicate article)
1066	Qi Q, Han Y, Xue C. [Influence of age on severe pancreatitis]. Zhonghua Wai Ke Za Zhi. 1995;33(9):542-4. PubMed PMID: 8731873.	Excluded from the final analysis	The article was excluded from the analysis because it was repetitive (duplicate article)

1067	Sharma M, Ponnusamy RP. Is balloon sweeping detrimental in portal biliopathy? A report of 3 cases. Gastrointestinal Endoscopy. 2009;70(1):171-3. doi: 10.1016/j.gie.2008.11.002. PubMed PMID: WOS:000267523200029.	Excluded from the final analysis	The article was excluded from the analysis because it was repetitive (duplicate article)
1068	Hussain A, Lafaurie G, Hafeez R, El-Hasani S. Is Specialisation Needed in Laparoscopic Cholecystectomy? A Retrospective Cohort Study of 5122 Patients. Chirurgia. 2020;115(6):756-66. doi: 10.21614/chirurgia.115.6.756. PubMed PMID: WOS:000604905000006.	Excluded from the final analysis	The article was excluded from the analysis because it was repetitive (duplicate article)
1069	Lonardo A, Grisendi A, Bonilauri S, Rambaldi M, Selmi I, Tondelli E. Ischaemic necrotizing pancreatitis after cardiac surgery. A case report and review of the literature. Italian Journal of Gastroenterology and Hepatology. 1999;31(9):872-5. PubMed PMID: WOS:000084809600009.	Excluded from the final analysis	The article was excluded from the analysis because it was repetitive (duplicate article)
1070	Prasaad PR, Shekhar S, Priyadharshini SA. Isolated non-necrotising granulomatous vasculitis of the gall bladder- a rare entity. J Clin Diagn Res. 2014;8(10):Fd01-2. Epub 20141020. doi: 10.7860/jcdr/2014/8599.4932. PubMed PMID: 25478351; PubMed Central PMCID: PMCPMC4253169.	Excluded from the final analysis	The article was excluded from the analysis because it was repetitive (duplicate article)
1071	Mostofsky E, Mukamal KJ, Giovannucci EL, Stampfer MJ, Rimm EB. Key Findings on Alcohol Consumption and a Variety of Health Outcomes From the Nurses' Health Study. American Journal of Public Health. 2016;106(9):1586-91. doi: 10.2105/ajph.2016.303336. PubMed PMID: WOS:000388072300022.	Excluded from the final analysis	The article was excluded from the analysis because it was repetitive (duplicate article)
1072	Hekmat M, Taghipoor HR, Nobahar MR, Monfared MB, Tehrani MMM, Arabnia MK, et al. Laparascopic cholecystectomy and open-heart surgery at the same time. Journal of Cardiac Surgery. 2005;20(6):557-9. doi: 10.1111/j.1540-8191.2005.00096.x. PubMed PMID: WOS:000233541500013.	Excluded from the final analysis	The article was excluded from the analysis because it was repetitive (duplicate article)
1073	Chen HY, Chang CJ, Yang YC, Lu FH, Sun ZJ, Wu JS. Renal Stones and Gallstones Correlated with the Ten-Year Risk Estimation of Atherosclerotic Cardiovascular Disease Based on the Pooled Cohort Risk Assessment of Males Aged 40-79. J Clin Med. 2023;12(6). Epub 20230316. doi: 10.3390/jcm12062309. PubMed PMID: 36983309; PubMed Central PMCID: PMCPMC10052154.	Excluded from the final analysis	The article was excluded from the analysis because it was repetitive (duplicate article)
1074	Chae W, Lee HS, Jo JH, Chung MJ, Bang S, Park SW, et al. Impact of cholecystectomy on acute coronary syndrome according to metabolic condition: a nationwide population-based cohort study. Sci Rep. 2023;13(1):7300. Epub 20230505. doi: 10.1038/s41598-023-33440-4. PubMed PMID: 37147417; PubMed Central PMCID: PMCPMC10163235.	Excluded from the final analysis	The article was excluded from the analysis because it was repetitive (duplicate article)

1075	Bai R, Wang J, Yang J, Cheng X, Zhang S, Zhang H, et al.!!!Gallbladder disease is associated with the risk of cardiovascular disease among Uyghurs in Xinjiang: a prospective cohort study. BMC Public Health. 2023;23(1):242. Epub 20230204. doi: 10.1186/s12889-023-15098-9. PubMed PMID: 36737734; PubMed Central PMCID: PMCPMC9898978.	Excluded from the final analysis	The article was excluded from the analysis because it was repetitive (duplicate article)
1076	Park SM, Kim HJ, Kang TU, Swan H, Ahn HS. Cholecystectomy reduces the risk of myocardial and cerebral infarction in patients with gallstone-related infection. Sci Rep. 2022;12(1):16749. Epub 20221006. doi: 10.1038/s41598-022-20700-y. PubMed PMID: 36202881; PubMed Central PMCID: PMCPMC9537563.	Excluded from the final analysis	The article was excluded from the analysis because it was repetitive (duplicate article)
1077	Ho TC, Chen YC, Lin CC, Tai HC, Wei CY, Yeh YH, Hsu CY. Reduced Risk of Atrial Fibrillation Following Cholecystectomy: A Nationwide Population-Based Study. Front Aging Neurosci. 2021;13:706815. Epub 20210902. doi: 10.3389/fnagi.2021.706815. PubMed PMID: 34539379; PubMed Central PMCID: PMCPMC8445074.	Excluded from the final analysis	The article was excluded from the analysis because it was repetitive (duplicate article)
1078	Gill ES, Jeong YJ, Lee J. Association between gallstone disease and ischemic stroke in Korea. Neurology Asia. 2021;26(4).	Excluded from the final analysis	The article was excluded from the analysis because it was repetitive (duplicate article)
1079	Chen CH, Lin CL, Kao CH. Risk of aortic dissection or aneurysm in patients with gallstone disease: a retrospective cohort study in Taiwan. BMJ Open. 2021;11(8):e049316. Epub 20210826. doi: 10.1136/bmjopen-2021-049316. PubMed PMID: 34446491; PubMed Central PMCID: PMCPMC8395267.	Excluded from the final analysis	The article was excluded from the analysis because it was repetitive (duplicate article)
1080	Shabanzadeh DM, Skaaby T, Sørensen LT, Jørgensen T. Screen-detected gallstone disease and cardiovascular disease. Eur J Epidemiol. 2017;32(6):501-10. Epub 20170527. doi: 10.1007/s10654-017-0263-x. PubMed PMID: 28551778.	Excluded from the final analysis	The article was excluded from the analysis because it was repetitive (duplicate article)
1081	Kwon CH, Kang JG, Lee HJ, Kim NH, Sung JW, Cheong E, Sung KC. ??Absence of association between gallstone and coronary artery calcification. Atherosclerosis. 2017;258:51-5. Epub 20170201. doi: 10.1016/j.atherosclerosis.2017.01.035. PubMed PMID: 28192729.	Excluded from the final analysis	The article was excluded from the analysis because it was repetitive (duplicate article)
1082	Zheng Y, Xu M, Li Y, Hruby A, Rimm EB, Hu FB, et al. Gallstones and Risk of Coronary Heart Disease: Prospective Analysis of 270 000 Men and Women From 3 US Cohorts and Meta-Analysis. Arterioscler Thromb	Excluded from the final analysis	The article was excluded from the analysis because it was repetitive (duplicate article)

	Vasc Biol. 2016;36(9):1997-2003. Epub 20160818. doi: 10.1161/atvbaha.116.307507. PubMed PMID: 27540264; PubMed Central PMCID: PMCPMC5001914.		
1083	Wirth J, di Giuseppe R, Wientzek A, Katzke VA, Kloss M, Kaaks R, et al. Presence of gallstones and the risk of cardiovascular diseases: The EPIC-Germany cohort study. Eur J Prev Cardiol. 2015;22(3):326-34. Epub 20131031. doi: 10.1177/2047487313512218. PubMed PMID: 24177267.	Excluded from the final analysis	The article was excluded from the analysis because it was repetitive (duplicate article)
1084	Lv J, Qi L, Yu C, Guo Y, Bian Z, Chen Y, et al. Gallstone Disease and the Risk of Ischemic Heart Disease. Arterioscler Thromb Vasc Biol. 2015;35(10):2232-7. Epub 20150813. doi: 10.1161/atvbaha.115.306043. PubMed PMID: 26272939; PubMed Central PMCID: PMCPMC4587542.	Excluded from the final analysis	The article was excluded from the analysis because it was repetitive (duplicate article)
1085	Wei CY, Chung TC, Chen CH, Lin CC, Sung FC, Chung WT, et al. Gallstone disease and the risk of stroke: a nationwide population-based study. J Stroke Cerebrovasc Dis. 2014;23(7):1813-20. Epub 20140621. doi: 10.1016/j.jstrokecerebrovasdis.2014.04.024. PubMed PMID: 24957305.	Excluded from the final analysis	The article was excluded from the analysis because it was repetitive (duplicate article)
1086	Olaiya MT, Chiou HY, Jeng JS, Lien LM, Hsieh FI. Significantly increased risk of cardiovascular disease among patients with gallstone disease: a population-based cohort study. PLoS One. 2013;8(10):e76448. Epub 20131003. doi: 10.1371/journal.pone.0076448. PubMed PMID: 24098504; PubMed Central PMCID: PMCPMC3789705.	Excluded from the final analysis	The article was excluded from the analysis because it was repetitive (duplicate article)
1087	Jiang ZY, Sheng X, Xu CY, Li WW, Chang XX, Sun LY, et al. Gallbladder gallstone disease is associated with newly diagnosed coronary artery atherosclerotic disease: a cross-sectional study. PLoS One. 2013;8(9):e75400. Epub 20130918. doi: 10.1371/journal.pone.0075400. PubMed PMID: 24058685; PubMed Central PMCID: PMCPMC3776774.	Excluded from the final analysis	The article was excluded from the analysis because it was repetitive (duplicate article)
1088	Khan HN, Harrison M, Bassett EE, Bates T. A 10-year follow-up of a longitudinal study of gallstone prevalence at necropsy in South East England. Dig Dis Sci. 2009;54(12):2736-41. doi: 10.1007/s10620-008-0682-3. PubMed PMID: 19160052.	Excluded from the final analysis	The article was excluded from the analysis because it was repetitive (duplicate article)
1089	Méndez-Sánchez N, Zamora-Valdés D, Flores-Rangel JA, Pérez-Sosa JA, Vásquez-Fernández F, Lezama-Mora JI, et al. Gallstones are associated with carotid atherosclerosis. Liver Int. 2008;28(3):402-6. Epub 20071206. doi: 10.1111/j.1478-3231.2007.01632.x. PubMed PMID: 18069975.	Excluded from the final analysis	The article was excluded from the analysis because it was repetitive (duplicate article)

1090	González-Pérez A, García Rodríguez LA. Gallbladder disease in the general population: association with cardiovascular morbidity and therapy. Pharmacoepidemiol Drug Saf. 2007;16(5):524-31. doi: 10.1002/pds.1346. PubMed PMID: 17103483.	Excluded from the final analysis	The article was excluded from the analysis because it was repetitive (duplicate article)
1091	Méndez-Sánchez N, Bahena-Aponte J, Chávez-Tapia NC, Motola-Kuba D, Sánchez-Lara K, Ponciano-Radríguez G, et al. Strong association between gallstones and cardiovascular disease. Am J Gastroenterol. 2005;100(4):827-30. doi: 10.1111/j.1572-0241.2005.41214.x. PubMed PMID: 15784027.	Excluded from the final analysis	The article was excluded from the analysis because it was repetitive (duplicate article)
1092	Bortnichak EA, Freeman DH, Jr., Ostfeld AM, Castelli WP, Kannel WB, Feinleib M, McNamara PM. The association between cholesterol cholelithiasis and coronary heart disease in Framingham, Massachusetts. Am J Epidemiol. 1985;121(1):19-30. doi: 10.1093/oxfordjournals.aje.a113978. PubMed PMID: 3155483.	Excluded from the final analysis	The article was excluded from the analysis because it was repetitive (duplicate article)
1093	Chen HY, Chang CJ, Yang YC, Lu FH, Sun ZJ, Wu JS. Renal Stones and Gallstones Correlated with the Ten-Year Risk Estimation of Atherosclerotic Cardiovascular Disease Based on the Pooled Cohort Risk Assessment of Males Aged 40-79. J Clin Med. 2023;12(6). Epub 20230316. doi: 10.3390/jcm12062309. PubMed PMID: 36983309; PubMed Central PMCID: PMCPMC10052154.	Excluded from the final analysis	The article was excluded from the analysis because it was repetitive (duplicate article)
1094	Chae W, Lee HS, Jo JH, Chung MJ, Bang S, Park SW, et al. Impact of cholecystectomy on acute coronary syndrome according to metabolic condition: a nationwide population-based cohort study. Sci Rep. 2023;13(1):7300. Epub 20230505. doi: 10.1038/s41598-023-33440-4. PubMed PMID: 37147417; PubMed Central PMCID: PMCPMC10163235.	Excluded from the final analysis	The article was excluded from the analysis because it was repetitive (duplicate article)
1095	Abraham P, Desai DC, Joshi AG. Gallstones in portal hypertension: is the liver or the portal hypertension responsible? J Assoc Physicians India. 2002;50:515-7. PubMed PMID: 12164400.	Excluded from the final analysis	The article was excluded from the analysis because it was repetitive (duplicate article)
1096	Abu-Abeid S, Gavert N, Klausner JM, Szold A. Bariatric surgery in adolescence. J Pediatr Surg. 2003;38(9):1379-82. doi: 10.1016/s0022-3468(03)00400-7. PubMed PMID: 14523824.	Excluded from the final analysis	The article was excluded from the analysis because it was repetitive (duplicate article)
1097	Acalovschi M, Buzas C, Radu C, Grigorescu M. Hepatitis C virus infection is a risk factor for gallstone disease: a prospective hospital-based study of patients with chronic viral C hepatitis. J Viral Hepat. 2009;16(12):860-6. Epub 20090526. doi: 10.1111/j.1365-2893.2009.01141.x. PubMed PMID: 19486279.	Excluded from the final analysis	The article was excluded from the analysis because it was repetitive (duplicate article)

1098	Adsay NV, Basturk O, Thirabanjasak D. Diagnostic features and differential diagnosis of autoimmune pancreatitis. Semin Diagn Pathol. 2005;22(4):309-17. doi: 10.1053/j.semdp.2006.04.008. PubMed PMID: 16939059.	Excluded from the final analysis	The article was excluded from the analysis because it was repetitive (duplicate article)
1099	Agrons GA, Corse WR, Markowitz RI, Suarez ES, Perry DR. Gastrointestinal manifestations of cystic fibrosis: radiologic-pathologic correlation. Radiographics. 1996;16(4):871-93. doi: 10.1148/radiographics.16.4.8835977. PubMed PMID: 8835977.	Excluded from the final analysis	The article was excluded from the analysis because it was repetitive (duplicate article)
1100	Aguiar RGP, Souza Júnior FEA, Rocha Júnior JLG, Pessoa F, Silva LPD, Carmo GCD. CLINICAL AND EPIDEMIOLOGICAL EVALUATION OF COMPLICATIONS ASSOCIATED WITH GALLSTONES IN A TERTIARY HOSPITAL. Arq Gastroenterol. 2022;59(3):352-7. doi: 10.1590/s0004-2803.202203000-64. PubMed PMID: 36102431.	Excluded from the final analysis	The article was excluded from the analysis because it was repetitive (duplicate article)
1101	Aguirre-Olmedo I, Cuendis-Velázquez A, Morales-Chávez CE, Torres-Ruiz MF, Rojano-Rodríguez ME, Cárdenas-Lailson LE. [Laparoscopic choledochoduodenostomy as an optional treatment choledocholithiasis]. Cir Cir. 2013;81(2):118-24. PubMed PMID: 23522312.	Excluded from the final analysis	The article was excluded from the analysis because it was repetitive (duplicate article)
1102	Ahmad A, Faridi S, Siddiqui F, Edhi MM, Khan M. Effect of bupivacaine soaked gauze in postoperative pain relief in laparoscopic cholecystectomy: a prospective observational controlled trial in 120 patients. Patient Saf Surg. 2015;9:31. Epub 20150915. doi: 10.1186/s13037-015-0077-2. PubMed PMID: 26379780; PubMed Central PMCID: PMCPMC4570680.	Excluded from the final analysis	The article was excluded from the analysis because it was repetitive (duplicate article)
1103	Ahmed MH, Barakat S, Almobarak AO. The association between renal stone disease and cholesterol gallstones: the easy to believe and not hard to retrieve theory of the metabolic syndrome. Ren Fail. 2014;36(6):957-62. Epub 20140331. doi: 10.3109/0886022x.2014.900424. PubMed PMID: 24678942.	Excluded from the final analysis	The article was excluded from the analysis because it was repetitive (duplicate article)
1104	Ahmed O, Asghar MS, Khurshaidi MN, Yasmin F, Kanwal N, Khokher AJ, et al. Provision of Surgical Services to COVID-19-Infected Patients at a Tertiary Care Center in Pakistan: A One-Year Clinical Review of the Year 2020 in General Surgery Department. Cureus. 2021;13(1):e12705. Epub 20210114. doi: 10.7759/cureus.12705. PubMed PMID: 33614312; PubMed Central PMCID: PMCPMC7883566.	Excluded from the final analysis	The article was excluded from the analysis because it was repetitive (duplicate article)

1105	Akbar N, Yaseen T, Muhammad A, Danish M, Adeel M, Khan SA, et al. A Tertiary Care Center's Experience with Clinicopathological Characteristics of Gallbladder Carcinoma in Our Population. Euroasian J Hepatogastroenterol. 2022;12(1):35-9. doi: 10.5005/jp-journals-10018-1375. PubMed PMID: 35990861; PubMed Central PMCID: PMCPMC9357526.	Excluded from the final analysis	The article was excluded from the analysis because it was repetitive (duplicate article)
1106	Akoudad S, Szklo M, McAdams MA, Fulop T, Anderson CA, Coresh J, Köttgen A. Correlates of kidney stone disease differ by race in a multi-ethnic middle-aged population: the ARIC study. Prev Med. 2010;51(5):416-20. Epub 20100827. doi: 10.1016/j.ypmed.2010.08.011. PubMed PMID: 20801154; PubMed Central PMCID: PMCPMC2964449.	Excluded from the final analysis	The article was excluded from the analysis because it was repetitive (duplicate article)
1107	Al-Abbasi G, Alhilfy AA, Al-Jasim A. Surgical Management of Gallstone Ileus in Low-Settings Hospital during COVID-19 Outbreak: A Case Report. Surg J (N Y). 2021;7(2):e54-e8. Epub 20210525. doi: 10.1055/s-0041-1725160. PubMed PMID: 34056103; PubMed Central PMCID: PMCPMC8149159.	Excluded from the final analysis	The article was excluded from the analysis because it was repetitive (duplicate article)
1108	Al-Salem AH. Should cholecystectomy be performed concomitantly with splenectomy in children with sickle-cell disease? Pediatr Surg Int. 2003;19(1-2):71-4. Epub 20030116. doi: 10.1007/s00383-002-0804-5. PubMed PMID: 12721729.	Excluded from the final analysis	The article was excluded from the analysis because it was repetitive (duplicate article)
1109	Al-Salem AH. Indications and complications of splenectomy for children with sickle cell disease. J Pediatr Surg. 2006;41(11):1909-15. doi: 10.1016/j.jpedsurg.2006.06.020. PubMed PMID: 17101369.	Excluded from the final analysis	The article was excluded from the analysis because it was repetitive (duplicate article)
1110	Al-Salem AH, Naserullah Z, Qaisaruddin S, Al-Abkari H, Al-Faraj A, Yassin YM. Splenic complications of the sickling syndromes and the role of splenectomy. J Pediatr Hematol Oncol. 1999;21(5):401-6. doi: 10.1097/00043426-199909000-00012. PubMed PMID: 10524454.	Excluded from the final analysis	The article was excluded from the analysis because it was repetitive (duplicate article)
1111	Alabdullah H, Aldarsouni FG, Dagestani H, Mashbari H. Balancing Urgency and Strategy in the Surgical Management of a Complex Case of Gallstone Ileus: A Surgical-Video-Based Case Report of a 60-Year-Old Female. Cureus. 2024;16(8):e67304. Epub 20240820. doi: 10.7759/cureus.67304. PubMed PMID: 39310589; PubMed Central PMCID: PMCPMC11415006.	Excluded from the final analysis	The article was excluded from the analysis because it was repetitive (duplicate article)

1112	Alammari RK, Alhessan AA, Alturki AA, Aburowais SA, Alsharif MH, Alshehri FH, et al. Xanthogranulomatous Cholecystitis: A Rare Variant of Chronic Cholecystitis. Cureus. 2022;14(1):e21400. Epub 20220119. doi: 10.7759/cureus.21400. PubMed PMID: 35198307; PubMed Central PMCID: PMCPMC8856638.	Excluded from the final analysis	The article was excluded from the analysis because it was repetitive (duplicate article)
1113	Alhamid MA, Ilie VC, Aioanei S, Stanciulea O, Minciuna CE, Lacatus M. Laparoscopic Cholecystectomy in Cirrhotic Patients: A Retrospective Study. Chirurgia (Bucur). 2021;116(1):34-41. doi: 10.21614/chirurgia.116.1.34. PubMed PMID: 33638324.	Excluded from the final analysis	The article was excluded from the analysis because it was repetitive (duplicate article)
1114	Alicioglu B. Right Liver Lobe Hypoplasia and Related Abnormalities. Pol J Radiol. 2015;80:503-5. Epub 20151113. doi: 10.12659/pjr.894658. PubMed PMID: 26634012; PubMed Central PMCID: PMCPMC4648108.	Excluded from the final analysis	The article was excluded from the analysis because it was repetitive (duplicate article)
1115	Almashhrawi AA, Ahmed KT, Rahman RN, Hammoud GM, Ibdah JA. Liver diseases in pregnancy: diseases not unique to pregnancy. World J Gastroenterol. 2013;19(43):7630-8. doi: 10.3748/wjg.v19.i43.7630. PubMed PMID: 24282352; PubMed Central PMCID: PMCPMC3837261.	Excluded from the final analysis	The article was excluded from the analysis because it was repetitive (duplicate article)
1116	Alshamali DM, Horan M, Alhmaidy O, Mozaffar WK, Mozaffar JA, Al Ahmad Y. Surgical removal of giant adrenal lipoma with gallstones in limited resources areas: A case report. Int J Surg Case Rep. 2024;121:109953. Epub 20240715. doi: 10.1016/j.ijscr.2024.109953. PubMed PMID: 39029216; PubMed Central PMCID: PMCPMC11295541.	Excluded from the final analysis	The article was excluded from the analysis because it was repetitive (duplicate article)
1117	Amato A, Mauro M, Trimarchi A, Secondo P, Battaglia C, Griffanti Bartoli F. [Postoperative course after laparoscopic surgery of the upper abdomen]. Minerva Chir. 1994;49(7-8):619-27. PubMed PMID: 7991166.	Excluded from the final analysis	The article was excluded from the analysis because it was repetitive (duplicate article)
1118	Ambartsumyan L, Flores A, Nurko S, Rodriguez L. Utility of Octreotide in Advancing Enteral Feeds in Children with Chronic Intestinal Pseudo-Obstruction. Paediatr Drugs. 2016;18(5):387-92. doi: 10.1007/s40272-016-0189-x. PubMed PMID: 27520652.	Excluded from the final analysis	The article was excluded from the analysis because it was repetitive (duplicate article)
1119	Amini A, Vaezi Z, Koury E, Zafar S, Chahla E. Portal Vein Thrombosis and Intra-Abdominal Hypertension Presenting as Complications of Hypertriglyceridemia-Induced Severe Acute Pancreatitis. Cureus.	Excluded from the final analysis	The article was excluded from the analysis because it was repetitive (duplicate article)

	2020;12(8):e9889. Epub 20200820. doi: 10.7759/cureus.9889. PubMed PMID: 32968555; PubMed Central PMCID: PMCPMC7502419.		
1120	Ando H, Ootake Y, Asaka S. Subacute pulmonary hypertension due to pulmonary tumor microembolism as a clinical manifestation of occult gallbladder adenocarcinoma. Jpn Circ J. 1997;61(1):82-6. doi: 10.1253/jcj.61.82. PubMed PMID: 9070964.	Excluded from the final analysis	The article was excluded from the analysis because it was repetitive (duplicate article)
1121	Antunes ML, Cabral G, Tavares R, Noronha C, Araújo J. Going Round in Circles with a Multisystemic Disease: A Unique Case of Parasitic Aortitis. Eur J Case Rep Intern Med. 2017;4(6):000601. Epub 20170517. doi: 10.12890/2017_000601. PubMed PMID: 30755949; PubMed Central PMCID: PMCPMC6346790.	Excluded from the final analysis	The article was excluded from the analysis because it was repetitive (duplicate article)
1122	Anwar S, Rasool Malik AA, Hamza A, Shahid MS, Subhan M, Bibi R. A Complex Case of Obstructive Jaundice in a Septuagenarian: Diagnostic Challenges and Therapeutic Strategies. Cureus. 2024;16(7):e64598. Epub 20240715. doi: 10.7759/cureus.64598. PubMed PMID: 39149640; PubMed Central PMCID: PMCPMC11325256.	Excluded from the final analysis	The article was excluded from the analysis because it was repetitive (duplicate article)
1123	Anwer M, Asghar MS, Rahman S, Kadir S, Yasmin F, Mohsin D, et al. Diagnostic Accuracy of Endoscopic Ultrasonography Versus the Gold Standard Endoscopic Retrograde Cholangiopancreatography in Detecting Common Bile Duct Stones. Cureus. 2020;12(12):e12162. Epub 20201219. doi: 10.7759/cureus.12162. PubMed PMID: 33489574; PubMed Central PMCID: PMCPMC7813932.	Excluded from the final analysis	The article was excluded from the analysis because it was repetitive (duplicate article)
1124	Arendt T, Nizze H, Mönig H, Kloehn S, Stüber E, Fölsch UR. Biliary pancreatic reflux-induced acute pancreatitis--myth or possibility? Eur J Gastroenterol Hepatol. 1999;11(3):329-35. doi: 10.1097/00042737-199903000-00019. PubMed PMID: 10333208.	Excluded from the final analysis	The article was excluded from the analysis because it was repetitive (duplicate article)
1125	Arian R, Farwati R, Toutounji Z, Farho MA, Assi BE. Intestinal obstruction induced by gallstone migration through unusual location of a cholecystogastric fistula: A rare case report. Int J Surg Case Rep. 2024;122:110149. Epub 20240810. doi: 10.1016/j.ijscr.2024.110149. PubMed PMID: 39137645; PubMed Central PMCID: PMCPMC11357798.	Excluded from the final analysis	The article was excluded from the analysis because it was repetitive (duplicate article)
1126	Arora S, Anubhuti. Role of neuropeptides in appetite regulation and obesity--a review. Neuropeptides. 2006;40(6):375-401. Epub 20060828. doi: 10.1016/j.npep.2006.07.001. PubMed PMID: 16935329.	Excluded from the final analysis	The article was excluded from the analysis because it was repetitive (duplicate article)

1127	Arya AK, Bhadada SK, Kumari P, Agrawal K, Mukhopadhyay S, Sarma D, Rao SD. Differences in Primary Hyperparathyroidism Between Pre- and Postmenopausal Women in India. Endocr Pract. 2021;27(7):710-5. Epub 20201229. doi: 10.1016/j.eprac.2020.12.012. PubMed PMID: 33685668.	Excluded from the final analysis	The article was excluded from the analysis because it was repetitive (duplicate article)
1128	Ashwell M. Obesity in men and women. Int J Obes Relat Metab Disord. 1994;18 Suppl 1:S1-7. PubMed PMID: 8087159.	Excluded from the final analysis	The article was excluded from the analysis because it was repetitive (duplicate article)
1129	Assakran BS, Khalid R, Albadrani H, Alsuhaibani A, Almutairi A, Alhomidan R, et al. Incidence of Asymptomatic Gallstones in Obese Patients Who Underwent Bariatric Surgery in Qassim Region at King Fahad Specialist Hospital. Cureus. 2023;15(8):e44154. Epub 20230826. doi: 10.7759/cureus.44154. PubMed PMID: 37753031; PubMed Central PMCID: PMCPMC10519147.	Excluded from the final analysis	The article was excluded from the analysis because it was repetitive (duplicate article)
1130	Auda A, Al Abdullah R, Khalid MO, Alrasheed WY, Alsulaiman SA, Almulhem FT, et al. Acute Cholecystitis Presenting With Septic Shock as the First Presentation in an Elderly Patient. Cureus. 2022;14(1):e20981. Epub 20220106. doi: 10.7759/cureus.20981. PubMed PMID: 35004094; PubMed Central PMCID: PMCPMC8733902.	Excluded from the final analysis	The article was excluded from the analysis because it was repetitive (duplicate article)
1131	Azadbakht S, Parvaee R, Darabian S. An epidemiological investigation of gallstone disease among patients admitted to Shahid Rahimi teaching hospital in Khorramabad in 2016-2020. Afr Health Sci. 2023;23(2):435-41. doi: 10.4314/ahs.v23i2.50. PubMed PMID: 38223601; PubMed Central PMCID: PMCPMC10782301.	Excluded from the final analysis	The article was excluded from the analysis because it was repetitive (duplicate article)
1132	Backman L, Hallberg D. Some somatic complications after small intestinal bypass operations for obesity. Possible factors of significance in the incidence. Acta Chir Scand. 1975;141(8):790-800. PubMed PMID: 1217445.	Excluded from the final analysis	The article was excluded from the analysis because it was repetitive (duplicate article)
1133	Baddam A, Akuma O, Raj R, Akuma CM, Augustine SW, Sheikh Hanafi I, et al. Analysis of Risk Factors for Cholelithiasis: A Single-Center Retrospective Study. Cureus. 2023;15(9):e46155. Epub 20230928. doi: 10.7759/cureus.46155. PubMed PMID: 37900464; PubMed Central PMCID: PMCPMC10613112.	Excluded from the final analysis	The article was excluded from the analysis because it was repetitive (duplicate article)
1134	Báez-García JJ, Martínez-Hernández-Magro P, Iriarte-Gállego G. [Bouveret's syndrome; a case report]. Rev Gastroenterol Mex. 2009;74(2):118-21. PubMed PMID: 19666294.	Excluded from the final analysis	The article was excluded from the analysis because it was repetitive (duplicate article)

1135	Bahloul M, Ayedi M, Dammak H, Trabelsi K, Bouaziz M. [Pregnancy-induced hypertension complicated by acute pancreatitis]. Ann Fr Anesth Reanim. 2004;23(2):157-9. doi: 10.1016/j.annfar.2003.11.017. PubMed PMID: 15030867.	Excluded from the final analysis	The article was excluded from the analysis because it was repetitive (duplicate article)
1136	Barcenas CG, Gonzalez-Molina M, Hull AR. Association between acute pancreatitis and malignant hypertension with renal failure. Arch Intern Med. 1978;138(8):1254-6. PubMed PMID: 677980.	Excluded from the final analysis	The article was excluded from the analysis because it was repetitive (duplicate article)
1137	Barie PS, Eachempati SR. Acute acalculous cholecystitis. Curr Gastroenterol Rep. 2003;5(4):302-9. doi: 10.1007/s11894-003-0067-x. PubMed PMID: 12864960.	Excluded from the final analysis	The article was excluded from the analysis because it was repetitive (duplicate article)
1138	Barie PS, Eachempati SR. Acute acalculous cholecystitis. Gastroenterol Clin North Am. 2010;39(2):343-57, x. doi: 10.1016/j.gtc.2010.02.012. PubMed PMID: 20478490.	Excluded from the final analysis	The article was excluded from the analysis because it was repetitive (duplicate article)
1139	Barnard ND, Nicholson A, Howard JL. The medical costs attributable to meat consumption. Prev Med. 1995;24(6):646-55. doi: 10.1006/pmed.1995.1100. PubMed PMID: 8610089.	Excluded from the final analysis	The article was excluded from the analysis because it was repetitive (duplicate article)
1140	Basnet S, Merikanto I, Lahti T, Männistö S, Laatikainen T, Vartiainen E, Partonen T. Associations of common chronic non-communicable diseases and medical conditions with sleep-related problems in a population-based health examination study. Sleep Sci. 2016;9(3):249-54. Epub 20161125. doi: 10.1016/j.slsci.2016.11.003. PubMed PMID: 28123670; PubMed Central PMCID: PMCPMC5241609.	Excluded from the final analysis	The article was excluded from the analysis because it was repetitive (duplicate article)
1141	Bessa SS, Katri KM, Abdel-Salam WN, El-Kayal el SA, Tawfik TA. Spinal versus general anesthesia for day-case laparoscopic cholecystectomy: a prospective randomized study. J Laparoendosc Adv Surg Tech A. 2012;22(6):550-5. Epub 20120611. doi: 10.1089/lap.2012.0110. PubMed PMID: 22686181.	Excluded from the final analysis	The article was excluded from the analysis because it was repetitive (duplicate article)
1142	Bhaskar BS, Rao G, Joshi SB, Arun S, Ajay S. Anaesthesia for laparoscopic cholecystectomy in Bartter's syndrome. Indian J Anaesth. 2010;54(4):327-30. doi: 10.4103/0019-5049.68377. PubMed PMID: 20882176; PubMed Central PMCID: PMCPMC2943703.	Excluded from the final analysis	The article was excluded from the analysis because it was repetitive (duplicate article)

1143	Bhatia P, John S, Kalhan S, Khetan M. Primary laparoscopic cholecystectomy in patients with portal cavernoma and non-obstructive portal biliopathy: Two case reports. J Minim Access Surg. 2014;10(3):161-2. doi: 10.4103/0972-9941.134885. PubMed PMID: 25013336; PubMed Central PMCID: PMCPMC4083552.	Excluded from the final analysis	The article was excluded from the analysis because it was repetitive (duplicate article)
1144	Bhattacharjee PK, Choudhury D, Rai H, Ram N, Chattopadhyay D, Roy RP. Spontaneous perforation of common bile duct: a rare complication of choledocholithiasis. Indian J Surg. 2009;71(2):92-4. Epub 20090502. doi: 10.1007/s12262-009-0024-5. PubMed PMID: 23133123; PubMed Central PMCID: PMCPMC3452614.	Excluded from the final analysis	The article was excluded from the analysis because it was repetitive (duplicate article)
1145	Bian SN, Yang HH, Wang Q, Xu D, Zhao Y. [The 452th case: rash, hypotension, abdominal pain and headache]. Zhonghua Nei Ke Za Zhi. 2016;55(9):741-4. doi: 10.3760/cma.j.issn.0578-1426.2016.09.020. PubMed PMID: 27586989.	Excluded from the final analysis	The article was excluded from the analysis because it was repetitive (duplicate article)
1146	Bianchi P, Fermo E. Molecular heterogeneity of pyruvate kinase deficiency. Haematologica. 2020;105(9):2218-28. Epub 20200901. doi: 10.3324/haematol.2019.241141. PubMed PMID: 33054047; PubMed Central PMCID: PMCPMC7556514.	Excluded from the final analysis	The article was excluded from the analysis because it was repetitive (duplicate article)
1147	Bickler SW, DeMaio A. Western diseases: current concepts and implications for pediatric surgery research and practice. Pediatr Surg Int. 2008;24(3):251-5. Epub 20071218. doi: 10.1007/s00383-007-2095-3. PubMed PMID: 18087704.	Excluded from the final analysis	The article was excluded from the analysis because it was repetitive (duplicate article)
1148	Boatright JH, Nickerson JM, Moring AG, Pardue MT. Bile acids in treatment of ocular disease. J Ocul Biol Dis Infor. 2009;2(3):149-59. Epub 20090827. doi: 10.1007/s12177-009-9030-x. PubMed PMID: 20046852; PubMed Central PMCID: PMCPMC2798994.	Excluded from the final analysis	The article was excluded from the analysis because it was repetitive (duplicate article)
1149	Boey JH, Way LW. Acute cholangitis. Ann Surg. 1980;191(3):264-70. doi: 10.1097/00000658-198003000-00002. PubMed PMID: 7362292; PubMed Central PMCID: PMCPMC1344694.	Excluded from the final analysis	The article was excluded from the analysis because it was repetitive (duplicate article)
1150	Boraschi P, Giugliano L, Mercogliano G, Donati F, Romano S, Neri E. Abdominal and gastrointestinal manifestations in COVID-19 patients: Is imaging useful? World J Gastroenterol. 2021;27(26):4143-59. doi: 10.3748/wjg.v27.i26.4143. PubMed PMID: 34326615; PubMed Central PMCID: PMCPMC8311532.	Excluded from the final analysis	The article was excluded from the analysis because it was repetitive (duplicate article)

1151	Borgna-Pignatti C, Marsella M, Zanforlin N. The natural history of thalassemia intermedia. Ann N Y Acad Sci. 2010;1202:214-20. doi: 10.1111/j.1749-6632.2010.05550.x. PubMed PMID: 20712795.	Excluded from the final analysis	The article was excluded from the analysis because it was repetitive (duplicate article)
1152	Bornman PC, Terblanche J. Subtotal cholecystectomy: for the difficult gallbladder in portal hypertension and cholecystitis. Surgery. 1985;98(1):1-6. PubMed PMID: 3892743.	Excluded from the final analysis	The article was excluded from the analysis because it was repetitive (duplicate article)
1153	Bray GA. Health hazards of obesity. Endocrinol Metab Clin North Am. 1996;25(4):907-19. doi: 10.1016/s0889-8529(05)70361-3. PubMed PMID: 8977052.	Excluded from the final analysis	The article was excluded from the analysis because it was repetitive (duplicate article)
1154	Brodribb AJ, Humphreys DM. Diverticular disease: three studies. Part I--Relation to other disorders and fibre intake. Br Med J. 1976;1(6007):424-5. doi: 10.1136/bmj.1.6007.424. PubMed PMID: 1252774; PubMed Central PMCID: PMCPMC1638933.	Excluded from the final analysis	The article was excluded from the analysis because it was repetitive (duplicate article)
1155	Busbaih Z, Busbaih J, Odeh A, Albeladi AM, Almohammed Saleh AA. Appendiceal Adhesion to the Gallbladder Detected During Laparoscopic Cholecystectomy: A Case Report. Cureus. 2021;13(12):e20625. Epub 20211222. doi: 10.7759/cureus.20625. PubMed PMID: 35106195; PubMed Central PMCID: PMCPMC8788890.	Excluded from the final analysis	The article was excluded from the analysis because it was repetitive (duplicate article)
1156	Butt Z, Hyder Q. Cholelithiasis in hepatic cirrhosis: evaluating the role of risk factors. J Pak Med Assoc. 2010;60(8):641-4. PubMed PMID: 20726194.	Excluded from the final analysis	The article was excluded from the analysis because it was repetitive (duplicate article)
1157	Canada R, Chaudry S, Gaber L, Waters B, Martinez A, Wall B. Polyarteritis nodosa and cryoglobulinemic glomerulonephritis related to chronic hepatitis C. Am J Med Sci. 2006;331(6):329-33. doi: 10.1097/00000441-200606000-00008. PubMed PMID: 16775442.	Excluded from the final analysis	The article was excluded from the analysis because it was repetitive (duplicate article)
1158	Cao Z, Wei J, Zhang N, Liu W, Hong T, He X, Qu Q. Risk factors of systematic biliary complications in patients with gallbladder stones. Ir J Med Sci. 2020;189(3):943-7. Epub 20191219. doi: 10.1007/s11845-019-02161-x. PubMed PMID: 31858450.	Excluded from the final analysis	The article was excluded from the analysis because it was repetitive (duplicate article)

1159	Cappellini MD, Musallam KM, Taher AT. Insight onto the pathophysiology and clinical complications of thalassemia intermedia. Hemoglobin. 2009;33 Suppl 1:S145-59. doi: 10.3109/03630260903351528. PubMed PMID: 20001620.	Excluded from the final analysis	The article was excluded from the analysis because it was repetitive (duplicate article)
1160	Cardoso R, Casela A, Lopes S, Agostinho C, Souto P, Camacho E, et al. Portal Hypertensive Biliopathy: An Infrequent Cause of Biliary Obstruction. GE Port J Gastroenterol. 2015;22(2):65-9. Epub 20150318. doi: 10.1016/j.jpge.2015.01.003. PubMed PMID: 28868376; PubMed Central PMCID: PMCPMC5579995.	Excluded from the final analysis	The article was excluded from the analysis because it was repetitive (duplicate article)
1161	Castellani C, Quinzii C, Altieri S, Mastella G, Assael BM. A pilot survey of cystic fibrosis clinical manifestations in CFTR mutation heterozygotes. Genet Test. 2001;5(3):249-54. doi: 10.1089/10906570152742317. PubMed PMID: 11788092.	Excluded from the final analysis	The article was excluded from the analysis because it was repetitive (duplicate article)
1162	Çelik A, Kut A, İlhan B. A report of a case: unusual portosystemic shunt in a hernia sac who has portal hypertension due to cirrhosis. Surg Radiol Anat. 2021;43(3):385-8. Epub 20200923. doi: 10.1007/s00276-020-02568-7. PubMed PMID: 32965518.	Excluded from the final analysis	The article was excluded from the analysis because it was repetitive (duplicate article)
1163	Cerhan JR, Bernstein L, Severson RK, Davis S, Colt JS, Blair A, Hartge P. Anthropometrics, physical activity, related medical conditions, and the risk of non-hodgkin lymphoma. Cancer Causes Control. 2005;16(10):1203-14. doi: 10.1007/s10552-005-0358-7. PubMed PMID: 16215871.	Excluded from the final analysis	The article was excluded from the analysis because it was repetitive (duplicate article)
1164	Cha BH. Epidemiological Characteristics of Gallbladder Cancer in Jeju Island: A Single-Center, Clinically Based, Age-Sex-Matched, Case-Control Study. Asian Pac J Cancer Prev. 2015;16(18):8451-4. doi: 10.7314/apjcp.2015.16.18.8451. PubMed PMID: 26745100.	Excluded from the final analysis	The article was excluded from the analysis because it was repetitive (duplicate article)
1165	Chacko S, Jadhav U, Ghewade B, Wagh P, Prasad R, Wanjari MB. Sickle Cell Disease (SCD) Leading to Pulmonary Arterial Hypertension (PAH) and Cholelithiasis (CL). Cureus. 2023;15(4):e37113. Epub 20230404. doi: 10.7759/cureus.37113. PubMed PMID: 37153282; PubMed Central PMCID: PMCPMC10159007.	Excluded from the final analysis	The article was excluded from the analysis because it was repetitive (duplicate article)
1166	Chae W, Lee HS, Jo JH, Chung MJ, Bang S, Park SW, et al. Impact of cholecystectomy on acute coronary syndrome according to metabolic condition: a nationwide population-based cohort study. Sci Rep. 2023;13(1):7300. Epub 20230505. doi: 10.1038/s41598-023-33440-4. PubMed PMID: 37147417; PubMed Central PMCID: PMCPMC10163235.	Excluded from the final analysis	The article was excluded from the analysis because it was repetitive (duplicate article)

1167	Chahal-Kummen M, Salte OBK, Hewitt S, Blom-Høgestøl IK, Risstad H, Kristinsson J, Mala T. Health benefits and risks during 10 years after Roux-en-Y gastric bypass. Surg Endosc. 2020;34(12):5368-76. Epub 20200128. doi: 10.1007/s00464-019-07328-2. PubMed PMID: 31993812; PubMed Central PMCID: PMCPMC7644522.	Excluded from the final analysis	The article was excluded from the analysis because it was repetitive (duplicate article)
1168	Challand C, Titcomb D, Armstrong CP. Pancreatic pseudocyst causing celiac artery trunk thrombosis. Jop. 2008;9(4):512-4. Epub 20080710. PubMed PMID: 18648144.	Excluded from the final analysis	The article was excluded from the analysis because it was repetitive (duplicate article)
1169	Chang CC, Hsieh YY, Tsai HD, Yang TC, Yeh LS, Hsu TY. Acute pancreatitis in pregnancy. Zhonghua Yi Xue Za Zhi (Taipei). 1998;61(2):85-92. PubMed PMID: 9532870.	Excluded from the final analysis	The article was excluded from the analysis because it was repetitive (duplicate article)
1170	Chang CH, Wang YY, Jiao Y. Hepatitis A virus-associated acute acalculous cholecystitis in an adult-onset Still's disease patient: A case report and review of the literature. World J Clin Cases. 2023;11(6):1410-8. doi: 10.12998/wjcc.v11.i6.1410. PubMed PMID: 36926135; PubMed Central PMCID: PMCPMC10013114.	Excluded from the final analysis	The article was excluded from the analysis because it was repetitive (duplicate article)
1171	Chaouch MA, Taieb AH, Kawach A, Zenati H, Gafsi B, Noomen F. Challenges in the management of acute lithiasic cholangitis due to a long-retained plastic biliary stent: A case report. Int J Surg Case Rep. 2024;118:109690. Epub 20240423. doi: 10.1016/j.ijscr.2024.109690. PubMed PMID: 38669806; PubMed Central PMCID: PMCPMC11064558.	Excluded from the final analysis	The article was excluded from the analysis because it was repetitive (duplicate article)
1172	Chaturvedi N, Ben-Shlomo Y. From the surgery to the surgeon: does deprivation influence consultation and operation rates? Br J Gen Pract. 1995;45(392):127-31. PubMed PMID: 7772389; PubMed Central PMCID: PMCPMC1239172.	Excluded from the final analysis	The article was excluded from the analysis because it was repetitive (duplicate article)
1173	Chaudry G, Navarro OM, Levine DS, Oudjhane K. Abdominal manifestations of cystic fibrosis in children. Pediatr Radiol. 2006;36(3):233-40. Epub 20060104. doi: 10.1007/s00247-005-0049-2. PubMed PMID: 16391928.	Excluded from the final analysis	The article was excluded from the analysis because it was repetitive (duplicate article)
1174	Chawla A, Dewan R, Sarin SK. The frequency and influence of gallbladder varices on gallbladder functions in patients with portal hypertension. Am J Gastroenterol. 1995;90(11):2010-4. PubMed PMID: 7485012.	Excluded from the final analysis	The article was excluded from the analysis because it was repetitive (duplicate article)

1175	Chen CH, Lin CL, Hsu CY, Kao CH. Risk of gallstones in patients with obstructive sleep apnea: a nationwide observational cohort study. Sleep Breath. 2019;23(1):355-62. Epub 20180707. doi: 10.1007/s11325-018-1696-5. PubMed PMID: 29980983.	Excluded from the final analysis	The article was excluded from the analysis because it was repetitive (duplicate article)
1176	Chen CH, Lin CL, Kao CH. Association of Gallbladder Polyp and Stroke: A Nationwide, Population-Based Study. Medicine (Baltimore). 2015;94(48):e2192. doi: 10.1097/md.0000000000002192. PubMed PMID: 26632906; PubMed Central PMCID: PMCPMC4674209.	Excluded from the final analysis	The article was excluded from the analysis because it was repetitive (duplicate article)
1177	Chen CH, Lin CL, Kao CH. The risk of coronary heart disease after diagnosis of gallbladder polyp: a retrospective nationwide population-based cohort study. Ann Transl Med. 2019;7(23):753. doi: 10.21037/atm.2019.11.114. PubMed PMID: 32042769; PubMed Central PMCID: PMCPMC6990032.	Excluded from the final analysis	The article was excluded from the analysis because it was repetitive (duplicate article)
1178	Chen CH, Lin CL, Kao CH. The Risk of Venous Thromboembolism in Patients with Gallstones. Int J Environ Res Public Health. 2020;17(8). Epub 20200423. doi: 10.3390/ijerph17082930. PubMed PMID: 32340378; PubMed Central PMCID: PMCPMC7215658.	Excluded from the final analysis	The article was excluded from the analysis because it was repetitive (duplicate article)
1179	Chen J, Liu ZT, Lyu JT, Jiang GP. Impact of metabolic disorders on gallstone disease and perioperative recovery after laparoscopic cholecystectomy. Hepatobiliary Pancreat Dis Int. 2024. Epub 20240805. doi: 10.1016/j.hbpd.2024.08.001. PubMed PMID: 39129076.	Excluded from the final analysis	The article was excluded from the analysis because it was repetitive (duplicate article)
1180	Chen LY, Qiao QH, Zhang SC, Chen YH, Chao GQ, Fang LZ. Metabolic syndrome and gallstone disease. World J Gastroenterol. 2012;18(31):4215-20. doi: 10.3748/wjg.v18.i31.4215. PubMed PMID: 22919256; PubMed Central PMCID: PMCPMC3422804.	Excluded from the final analysis	The article was excluded from the analysis because it was repetitive (duplicate article)
1181	Chetty R, Serra S. A pragmatic approach to vasculitis in the gastrointestinal tract. J Clin Pathol. 2017;70(6):470-5. Epub 20170124. doi: 10.1136/jclinpath-2016-204308. PubMed PMID: 28119348.	Excluded from the final analysis	The article was excluded from the analysis because it was repetitive (duplicate article)
1182	Chigot JP, Bitker M, Chalgadian R, Laroussinie G, Cabrol A, Gandjbakhch I, et al. [Abdominal complications of heart surgery]. Arch Mal Coeur Vaiss. 1981;74(6):665-73. PubMed PMID: 6794488.	Excluded from the final analysis	The article was excluded from the analysis because it was repetitive (duplicate article)

1183	Choi SY, Lee HK, Yi BH, Lee MH, Lee JE, Min JH, et al. Pope's hat sign: another valuable CT finding of early acute cholecystitis. Abdom Radiol (NY). 2018;43(7):1693-702. doi: 10.1007/s00261-017-1421-z. PubMed PMID: 29198010.	Excluded from the final analysis	The article was excluded from the analysis because it was repetitive (duplicate article)
1184	Chowdhary M, Kabbani AA, Chhabra A. Canagliflozin-induced pancreatitis: a rare side effect of a new drug. Ther Clin Risk Manag. 2015;11:991-4. Epub 20150626. doi: 10.2147/tcrm.S86641. PubMed PMID: 26170677; PubMed Central PMCID: PMCPMC4489815.	Excluded from the final analysis	The article was excluded from the analysis because it was repetitive (duplicate article)
1185	Coles M, Madray V, Uy P. Acute Esophageal Necrosis in a Septic Patient with a History of Cardiovascular Disease. Case Rep Gastrointest Med. 2020;2020:1416743. Epub 20200511. doi: 10.1155/2020/1416743. PubMed PMID: 32455033; PubMed Central PMCID: PMCPMC7238344.	Excluded from the final analysis	The article was excluded from the analysis because it was repetitive (duplicate article)
1186	Conn HO. A clinical hepatologist's predictions about non-absorbed carbohydrates for the early twenty-first century. Scand J Gastroenterol Suppl. 1997;222:88-92. doi: 10.1080/00365521.1997.11720727. PubMed PMID: 9145456.	Excluded from the final analysis	The article was excluded from the analysis because it was repetitive (duplicate article)
1187	Cormier RE, Chase BA, Peterson GS, Pauker SG. Abdominal pain, atherosclerosis, and atrial fibrillation. The case for mesenteric ischemia. Med Decis Making. 1982;2(3):323-39. doi: 10.1177/0272989x8200200309. PubMed PMID: 7169938.	Excluded from the final analysis	The article was excluded from the analysis because it was repetitive (duplicate article)
1188	Coulier B, Maldague P, Pierard F. Spontaneous transhepatic rupture of the gallbladder with massive hemoperitoneum. Jbr-btr. 2012;95(2):92-4. doi: 10.5334/jbr-btr.149. PubMed PMID: 22764665.	Excluded from the final analysis	The article was excluded from the analysis because it was repetitive (duplicate article)
1189	Covello B, Miller J, Fourzali R. Splenic vein stenting for recurrent chylous ascites in sinistral portal hypertension: a case report. CVIR Endovasc. 2021;4(1):26. Epub 20210303. doi: 10.1186/s42155-021-00213-x. PubMed PMID: 33656619; PubMed Central PMCID: PMCPMC7930171.	Excluded from the final analysis	The article was excluded from the analysis because it was repetitive (duplicate article)
1190	Cui Y, Li Z, Zhao E, Cui N. Risk factors in patients with hereditary gallstones in Chinese pedigrees. Med Princ Pract. 2012;21(5):467-71. Epub 20120331. doi: 10.1159/000337437. PubMed PMID: 22473058.	Excluded from the final analysis	The article was excluded from the analysis because it was repetitive (duplicate article)

1191	Culp WC, McCowan TC, DeValdenebro M, Wright LB, Workman JL, Culp WC, Jr. Paravertebral block: an improved method of pain control in percutaneous transhepatic biliary drainage. Cardiovasc Intervent Radiol. 2006;29(6):1015-21. doi: 10.1007/s00270-005-0273-z. PubMed PMID: 16988878.	Excluded from the final analysis	The article was excluded from the analysis because it was repetitive (duplicate article)
1192	Cunnigham D, Mills PR, Quigley EM, Patrick RS, Watkinson G, MacKenzie JF, Russell RI. Hepatic granulomas: experience over a 10-year period in the West of Scotland. Q J Med. 1982;51(202):162-70. PubMed PMID: 7111678.	Excluded from the final analysis	The article was excluded from the analysis because it was repetitive (duplicate article)
1193	Czerniak A, Thompson JN, Hemingway AP, Soreide O, Benjamin IS, Allison DJ, Blumgart LH. Hemobilia. A disease in evolution. Arch Surg. 1988;123(6):718-21. doi: 10.1001/archsurg.1988.01400300064010. PubMed PMID: 3369935.	Excluded from the final analysis	The article was excluded from the analysis because it was repetitive (duplicate article)
1194	Czupryniak L, Drzewoski J. [The role of pharmacotherapy for treatment of obesity in adults]. Pol Merkur Lekarski. 2000;9(53):741-5. PubMed PMID: 11204319.	Excluded from the final analysis	The article was excluded from the analysis because it was repetitive (duplicate article)
1195	D LTH, Papo T, Laraki R, Wechsler B, Blétry O, Chapelon C, et al. [Pancreatitis in systemic lupus erythematosus. Review of the literature apropos of 5 cases]. Rev Med Interne. 1994;15(2):89-94. doi: 10.1016/s0248-8663(05)81180-4. PubMed PMID: 8059127.	Excluded from the final analysis	The article was excluded from the analysis because it was repetitive (duplicate article)
1196	Dakour Aridi H, Alami R, Tamim H, Shamseddine G, Fouani T, Safadi B. Long-term outcomes of laparoscopic sleeve gastrectomy: a Lebanese center experience. Surg Obes Relat Dis. 2016;12(9):1689-96. Epub 20151127. doi: 10.1016/j.soard.2015.11.025. PubMed PMID: 26948944.	Excluded from the final analysis	The article was excluded from the analysis because it was repetitive (duplicate article)
1197	Dakour Aridi H, Sultanem S, Abtar H, Safadi BY, Fawal H, Alami RS. Management of gallbladder disease after sleeve gastrectomy in a selected Lebanese population. Surg Obes Relat Dis. 2016;12(7):1300-4. Epub 20160201. doi: 10.1016/j.soard.2016.01.029. PubMed PMID: 27178610.	Excluded from the final analysis	The article was excluded from the analysis because it was repetitive (duplicate article)
1198	Dalvi AN, Deshpande AA, Doctor NH, Maydeo A, Bapat RD. Laparoscopic cholecystectomy in patient with portal cavernoma and portal hypertension. Indian J Gastroenterol. 2001;20(1):32-3. PubMed PMID: 11206876.	Excluded from the final analysis	The article was excluded from the analysis because it was repetitive (duplicate article)

1199	de Alencastro MC, Cardoso KT, Mendes CA, Boteon YL, de Carvalho RB, Fraga GP. Acute intestinal obstruction due to gallstone ileus. Rev Col Bras Cir. 2013;40(4):275-80. doi: 10.1590/s0100-69912013000400004. PubMed PMID: 24173476.	Excluded from the final analysis	The article was excluded from the analysis because it was repetitive (duplicate article)
1200	de Lorimier AA. Alcohol, wine, and health. Am J Surg. 2000;180(5):357-61. doi: 10.1016/s0002-9610(00)00486-4. PubMed PMID: 11137687.	Excluded from the final analysis	The article was excluded from the analysis because it was repetitive (duplicate article)
1201	de Miranda Henriques MS, de Morais Villar EJ. The Liver and Polycystic Kidney Disease. In: Li X, editor. Polycystic Kidney Disease. Brisbane (AU): Codon Publications	Excluded from the final analysis	The article was excluded from the analysis because it was repetitive (duplicate article)
1202	Debry G. [Diet peculiarities. Vegetarianism, veganism, crudivorism, macrobiotism]. Rev Prat. 1991;41(11):967-72. PubMed PMID: 2063109.	Excluded from the final analysis	The article was excluded from the analysis because it was repetitive (duplicate article)
1203	Devkota HR, Bhandari B, Adhikary P. Perceived mental health, wellbeing and associated factors among Nepali male migrant and non-migrant workers: A qualitative study. J Migr Health. 2021;3:100013. Epub 20201204. doi: 10.1016/j.jmh.2020.100013. PubMed PMID: 34405181; PubMed Central PMCID: PMCPMC8352157.	Excluded from the final analysis	The article was excluded from the analysis because it was repetitive (duplicate article)
1204	Di Vita G, Sciumè C, Lauria Lauria G, Stella C, Raimondo D, Leo P. [Fibrinolytic system after laparoscopic cholecystectomy]. Minerva Chir. 2000;55(9):587-92. PubMed PMID: 11155471.	Excluded from the final analysis	The article was excluded from the analysis because it was repetitive (duplicate article)
1205	DiBianco JM, Lange J, Heidenberg D, Mufarrij P. Oxygen Venous Embolism After Hydrogen Peroxide Use During Percutaneous Nephrolithotomy. J Endourol Case Rep. 2019;5(1):25-7. Epub 20190318. doi: 10.1089/cren.2018.0111. PubMed PMID: 30989125; PubMed Central PMCID: PMCPMC6461058.	Excluded from the final analysis	The article was excluded from the analysis because it was repetitive (duplicate article)
1206	Diehl AK, Haffner SM, Hazuda HP, Stern MP. Coronary risk factors and clinical gallbladder disease: an approach to the prevention of gallstones? Am J Public Health. 1987;77(7):841-5. doi: 10.2105/ajph.77.7.841. PubMed PMID: 3496013; PubMed Central PMCID: PMCPMC1647214.	Excluded from the final analysis	The article was excluded from the analysis because it was repetitive (duplicate article)

1207	Diehl AK, Stern MP, Ostrower VS, Friedman PC. Prevalence of clinical gallbladder disease in Mexican-American, Anglo, and black women. South Med J. 1980;73(4):438-41, 43. doi: 10.1097/00007611-198004000-00012. PubMed PMID: 7367932.	Excluded from the final analysis	The article was excluded from the analysis because it was repetitive (duplicate article)
1208	Dietz WH. Obesity. J Am Coll Nutr. 1989;8 Suppl:13s-21s. doi: 10.1080/07315724.1989.10737966. PubMed PMID: 2681326.	Excluded from the final analysis	The article was excluded from the analysis because it was repetitive (duplicate article)
1209	Dong YH, Lu YF, Qiu Q, Huang H, Wei H. Clinical analysis of pericardial devascularization by preserving vagus trunks in 42 patients with portal hypertension. Asian J Surg. 2004;27(2):108-13. doi: 10.1016/s1015-9584(09)60322-5. PubMed PMID: 15140661.	Excluded from the final analysis	The article was excluded from the analysis because it was repetitive (duplicate article)
1210	Doran H, Mihalache O, Bobircă F, Bugă C, Pătraşcu T. [Acute acalculous cholecystitis--difficulties of diagnosis and treatment]. Chirurgia (Bucur). 2010;105(4):465-8. PubMed PMID: 20941966.	Excluded from the final analysis	The article was excluded from the analysis because it was repetitive (duplicate article)
1211	Dourakis SP, Tolis G. Sex hormonal preparations and the liver. Eur J Contracept Reprod Health Care. 1998;3(1):7-16. doi: 10.3109/13625189809167479. PubMed PMID: 9678067.	Excluded from the final analysis	The article was excluded from the analysis because it was repetitive (duplicate article)
1212	Drew BS, Dixon AF, Dixon JB. Obesity management: update on orlistat. Vasc Health Risk Manag. 2007;3(6):817-21. PubMed PMID: 18200802; PubMed Central PMCID: PMCPMC2350121.	Excluded from the final analysis	The article was excluded from the analysis because it was repetitive (duplicate article)
1213	Drinnon K, Puckett Y. Surgical Management of Gallstone Ileus: A Clinical Case Report. Cureus. 2021;13(12):e20141. Epub 20211203. doi: 10.7759/cureus.20141. PubMed PMID: 35003974; PubMed Central PMCID: PMCPMC8723775.	Excluded from the final analysis	The article was excluded from the analysis because it was repetitive (duplicate article)
1214	Dubecz A, Langer M, Stadlhuber RJ, Schweigert M, Solymosi N, Feith M, Stein HJ. Cholecystectomy in the very elderly--is 90 the new 70? J Gastrointest Surg. 2012;16(2):282-5. Epub 20111206. doi: 10.1007/s11605-011-1708-2. PubMed PMID: 22143419.	Excluded from the final analysis	The article was excluded from the analysis because it was repetitive (duplicate article)

1215	Dubnov G, Brzezinski A, Berry EM. Weight control and the management of obesity after menopause: the role of physical activity. Maturitas. 2003;44(2):89-101. doi: 10.1016/s0378-5122(02)00328-6. PubMed PMID: WOS:000181384600001.	Excluded from the final analysis	The article was excluded from the analysis because it was repetitive (duplicate article)
1216	Dumitraşcu DL, Pascu O, Drăghici A, Pop S, Dumitraşcu D, Iacob G. [Functional dyspepsia in liver cirrhosis]. Rom J Intern Med. 1996;34(1-2):91-6. PubMed PMID: 8963253.	Excluded from the final analysis	The article was excluded from the analysis because it was repetitive (duplicate article)
1217	Duseja A. Portal cavernoma cholangiopathy-clinical characteristics. J Clin Exp Hepatol. 2014;4(Suppl 1):S34-6. Epub 20131022. doi: 10.1016/j.jceh.2013.05.014. PubMed PMID: 25755593; PubMed Central PMCID: PMCPMC4244822.	Excluded from the final analysis	The article was excluded from the analysis because it was repetitive (duplicate article)
1218	Duvnjak M, Supanc V, Virović L, Tomasić V, Dojcinović B. [Caroli's disease]. Acta Med Croatica. 2003;57(3):249-52. PubMed PMID: 14582472.	Excluded from the final analysis	The article was excluded from the analysis because it was repetitive (duplicate article)
1219	Dwyer JT. Health aspects of vegetarian diets. Am J Clin Nutr. 1988;48(3 Suppl):712-38. doi: 10.1093/ajcn/48.3.712. PubMed PMID: 3046302.	Excluded from the final analysis	The article was excluded from the analysis because it was repetitive (duplicate article)
1220	Ebert E. Gastrointestinal involvement in spinal cord injury: a clinical perspective. J Gastrointestin Liver Dis. 2012;21(1):75-82. PubMed PMID: 22457863.	Excluded from the final analysis	The article was excluded from the analysis because it was repetitive (duplicate article)
1221	Efremova I, Maslennikov R, Poluektova E, Vasilieva E, Zharikov Y, Suslov A, et al. Epidemiology of small intestinal bacterial overgrowth. World J Gastroenterol. 2023;29(22):3400-21. doi: 10.3748/wjg.v29.i22.3400. PubMed PMID: 37389240; PubMed Central PMCID: PMCPMC10303511.	Excluded from the final analysis	The article was excluded from the analysis because it was repetitive (duplicate article)
1222	Ekwattanakit S, Siritanaratkul N, Viprakasit V. A prospective analysis for prevalence of complications in Thai nontransfusion-dependent Hb E/β-thalassemia and α-thalassemia (Hb H disease). Am J Hematol. 2018;93(5):623-9. Epub 20180214. doi: 10.1002/ajh.25046. PubMed PMID: 29359464.	Excluded from the final analysis	The article was excluded from the analysis because it was repetitive (duplicate article)

1223	El Kininy W, Kearney L, Hosam N, Broe P, Keeling A. Recurrent variceal haemorrhage managed with splenic vein stenting. Ir J Med Sci. 2017;186(2):323-7. Epub 20160225. doi: 10.1007/s11845-016-1420-z. PubMed PMID: 26911860.	Excluded from the final analysis	The article was excluded from the analysis because it was repetitive (duplicate article)
1224	Fan J, Sun Z, Yu C, Guo Y, Pei P, Yang L, et al. Multimorbidity patterns and association with mortality in 0.5 million Chinese adults. Chin Med J (Engl). 2022;135(6):648-57. Epub 20220320. doi: 10.1097/cm9.0000000000001985. PubMed PMID: 35191418; PubMed Central PMCID: PMCPMC9276333.	Excluded from the final analysis	The article was excluded from the analysis because it was repetitive (duplicate article)
1225	Fan Z, Pan JY, Zhang YW. Recovery from a biliary stricture of a common bile duct ligature injury: A case report. World J Clin Cases. 2020;8(16):3567-72. doi: 10.12998/wjcc.v8.i16.3567. PubMed PMID: 32913865; PubMed Central PMCID: PMCPMC7457115.	Excluded from the final analysis	The article was excluded from the analysis because it was repetitive (duplicate article)
1226	Fang JG, Zhu J, Li XJ, Li R, Dai F, Song XM, et al. [Epidemiological survey of prevalence of fatty liver and its risk factors in a general adult population of Shanghai]. Zhonghua Gan Zang Bing Za Zhi. 2005;13(2):83-8. PubMed PMID: 15727689.	Excluded from the final analysis	The article was excluded from the analysis because it was repetitive (duplicate article)
1227	Fei Y, Li WQ, Zong GQ, Chen J, Wang W. Cholecystolithotomy Combined Armillarisin A versus Cholecystectomy in Cirrhotic Portal Hypertension Patients with Symptomatic Cholelithiasis. Chirurgia (Bucur). 2017;112(2):143-51. doi: 10.21614/chirurgia.112.2.143. PubMed PMID: 28463673.	Excluded from the final analysis	The article was excluded from the analysis because it was repetitive (duplicate article)
1228	Fernandes V, Alfaro TM, Baptista JP, Regateiro FS, Fradinho F, Cordeiro CR. Severe ketorolac-induced asthma diagnosed by chest computed tomography. J Thorac Dis. 2017;9(Suppl 16):S1567-s9. doi: 10.21037/jtd.2017.11.36. PubMed PMID: 29255644; PubMed Central PMCID: PMCPMC5717355.	Excluded from the final analysis	The article was excluded from the analysis because it was repetitive (duplicate article)
1229	Field AE, Coakley EH, Must A, Spadano JL, Laird N, Dietz WH, et al. Impact of overweight on the risk of developing common chronic diseases during a 10-year period. Arch Intern Med. 2001;161(13):1581-6. doi: 10.1001/archinte.161.13.1581. PubMed PMID: 11434789.	Excluded from the final analysis	The article was excluded from the analysis because it was repetitive (duplicate article)
1230	Filippou DK, Triga A, Rizos S, Grigoriadis E, Shipkov CD, Nissiotis AS. Electrocardiographic changes after laparoscopic cholecystectomy. Folia Med (Plovdiv). 2004;46(4):37-41. PubMed PMID: 15962814.	Excluded from the final analysis	The article was excluded from the analysis because it was repetitive (duplicate article)

1231	Finucci G, Tirelli M, Bellon S, Zambon M, Toffolo L, Merkel C, Zuin R. Clinical significance of cholelithiasis in patients with decompensated cirrhosis. J Clin Gastroenterol. 1990;12(5):538-41. doi: 10.1097/00004836-199010000-00010. PubMed PMID: 2229996.	Excluded from the final analysis	The article was excluded from the analysis because it was repetitive (duplicate article)
1232	Foster RJ, Cowell GW. Acute paraumbilical vein recanalization: an unusual complication of acute pancreatitis. BJR Case Rep. 2015;1(1):20150021. Epub 20150420. doi: 10.1259/bjrcr.20150021. PubMed PMID: 30363191; PubMed Central PMCID: PMCPMC6159162.	Excluded from the final analysis	The article was excluded from the analysis because it was repetitive (duplicate article)
1233	Frankenburg FR, Zanarini MC. Obesity and obesity-related illnesses in borderline patients. J Pers Disord. 2006;20(1):71-80. doi: 10.1521/pedi.2006.20.1.71. PubMed PMID: 16563080.	Excluded from the final analysis	The article was excluded from the analysis because it was repetitive (duplicate article)
1234	Franks PW, Atabaki-Pasdar N. Causal inference in obesity research. J Intern Med. 2017;281(3):222-32. Epub 20161208. doi: 10.1111/joim.12577. PubMed PMID: 27933671.	Excluded from the final analysis	The article was excluded from the analysis because it was repetitive (duplicate article)
1235	Froutan Y, Alizadeh A, Mansour-Ghanaei F, Joukar F, Froutan H, Bagheri FB, et al. Gallstone disease founded by ultrasonography in functional dyspepsia: prevalence and associated factors. Int J Clin Exp Med. 2015;8(7):11283-8. Epub 20150715. PubMed PMID: 26379936; PubMed Central PMCID: PMCPMC4565319.	Excluded from the final analysis	The article was excluded from the analysis because it was repetitive (duplicate article)
1236	Fry RE, Fry WJ. Cholelithiasis and aortic reconstruction: the problem of simultaneous surgical therapy. Conclusions from a personal series. J Vasc Surg. 1986;4(4):345-50. PubMed PMID: 3761476.	Excluded from the final analysis	The article was excluded from the analysis because it was repetitive (duplicate article)
1237	Fujita T, Hayashi K, Katanoda K, Matsumura Y, Lee JS, Takagi H, et al. Prevalence of diseases and statistical power of the Japan Nurses' Health Study. Ind Health. 2007;45(5):687-94. doi: 10.2486/indhealth.45.687. PubMed PMID: 18057812.	Excluded from the final analysis	The article was excluded from the analysis because it was repetitive (duplicate article)
1238	Gadacz TR, Talamini MA, Lillemoe KD, Yeo CJ. Laparoscopic cholecystectomy. Surg Clin North Am. 1990;70(6):1249-62. doi: 10.1016/s0039-6109(16)45282-5. PubMed PMID: 2147301.	Excluded from the final analysis	The article was excluded from the analysis because it was repetitive (duplicate article)

1239	Gaitini D, Kreitenberg AJ, Fischer D, Maza I, Chowers Y. Color-coded duplex sonography compared to multidetector computed tomography for the diagnosis of crohn disease relapse and complications. J Ultrasound Med. 2011;30(12):1691-9. doi: 10.7863/jum.2011.30.12.1691. PubMed PMID: 22124005.	Excluded from the final analysis	The article was excluded from the analysis because it was repetitive (duplicate article)
1240	Garcea D, Martuzzi F, Santelmo N, Savoia M, Casertano MG, Furno A, Ruggeri V. Post-surgical deep vein thrombosis prevention: evaluation of the risk/benefit ratio of fractionated and unfractionated heparin. Curr Med Res Opin. 1992;12(9):572-83. doi: 10.1185/03007999209111524. PubMed PMID: 1316258.	Excluded from the final analysis	The article was excluded from the analysis because it was repetitive (duplicate article)
1241	Gasparrini M, Liverani A, Catracchia V, Conte S, Leonardo G, Marino G, et al. Gallstone ileus: a case report and review of the literature. Chir Ital. 2008;60(5):755-9. PubMed PMID: 19062503.	Excluded from the final analysis	The article was excluded from the analysis because it was repetitive (duplicate article)
1242	Gavriilidis P, Paily A. Colonic Perforation Secondary to Gallstone Impaction in the Sigmoid Colon. Case Rep Surg. 2023;2023:9986665. Epub 20230720. doi: 10.1155/2023/9986665. PubMed PMID: 37521369; PubMed Central PMCID: PMCPMC10374380.	Excluded from the final analysis	The article was excluded from the analysis because it was repetitive (duplicate article)
1243	Georgescu D, Ionita I, Lascu A, Hut EF, Dragan S, Ancusa OE, et al. Gallstone Disease and Bacterial Metabolic Performance of Gut Microbiota in Middle-Aged and Older Patients. Int J Gen Med. 2022;15:5513-31. Epub 20220608. doi: 10.2147/ijgm.S350104. PubMed PMID: 35702368; PubMed Central PMCID: PMCPMC9188808.	Excluded from the final analysis	The article was excluded from the analysis because it was repetitive (duplicate article)
1244	Ghassemian AJ, MacDonald KG, Cunningham PG, Swanson M, Brown BM, Morris PG, Pories WJ. The workup for bariatric surgery does not require a routine upper gastrointestinal series. Obes Surg. 1997;7(1):16-8. doi: 10.1381/096089297765556169. PubMed PMID: 9730531.	Excluded from the final analysis	The article was excluded from the analysis because it was repetitive (duplicate article)
1245	Gibadulina IO, Gibadulin NV. [Diagnostic aspects of chronic cholangitis after cholecystectomy]. Eksp Klin Gastroenterol. 2011;(6):68-72. PubMed PMID: 22168082.	Excluded from the final analysis	The article was excluded from the analysis because it was repetitive (duplicate article)
1246	Gonçalves M, Rebelo A, Costa R, Ferreira A. Endoscopic ampullectomy of a giant pedunculated ampullary adenoma. Rev Esp Enferm Dig. 2023;115(8):467-8. doi: 10.17235/reed.2022.9316/2022. PubMed PMID: 36426861.	Excluded from the final analysis	The article was excluded from the analysis because it was repetitive (duplicate article)

1247	González-Robles ME, Menéndez-Goti LL, de Jesús González-Luna A, Cuevas-Calla CV, Torres-Salazar QL. Gallstone ileus presenting in an elderly patient: A case report. Int J Surg Case Rep. 2024;124:110440. Epub 20241012. doi: 10.1016/j.ijscr.2024.110440. PubMed PMID: 39405751.	Excluded from the final analysis	The article was excluded from the analysis because it was repetitive (duplicate article)
1248	Gorsky RD, Pamuk E, Williamson DF, Shaffer PA, Koplan JP. The 25-year health care costs of women who remain overweight after 40 years of age. Am J Prev Med. 1996;12(5):388-94. PubMed PMID: 8909650.	Excluded from the final analysis	The article was excluded from the analysis because it was repetitive (duplicate article)
1249	Grace RF, Barcellini W. Management of pyruvate kinase deficiency in children and adults. Blood. 2020;136(11):1241-9. doi: 10.1182/blood.2019000945. PubMed PMID: 32702739.	Excluded from the final analysis	The article was excluded from the analysis because it was repetitive (duplicate article)
1250	Grace RF, Bianchi P, van Beers EJ, Eber SW, Glader B, Yaish HM, et al. Clinical spectrum of pyruvate kinase deficiency: data from the Pyruvate Kinase Deficiency Natural History Study. Blood. 2018;131(20):2183-92. Epub 20180316. doi: 10.1182/blood-2017-10-810796. PubMed PMID: 29549173.	Excluded from the final analysis	The article was excluded from the analysis because it was repetitive (duplicate article)
1251	Grigor'eva IN, Iamlikhanova A. [Frequency combination of a gallstone disease and diabetes]. Eksp Klin Gastroenterol. 2011;(4):99-102. PubMed PMID: 21916209.	Excluded from the final analysis	The article was excluded from the analysis because it was repetitive (duplicate article)
1252	Grigor'eva IN, Romanova TI. [Basic risk factors and quality of a life at gallstone disease patients]. Eksp Klin Gastroenterol. 2011;(4):21-5. PubMed PMID: 21919235.	Excluded from the final analysis	The article was excluded from the analysis because it was repetitive (duplicate article)
1253	Grønlykke L, Tarp B, Dutoit SH, Wilkens R. Peliosis hepatis: a complicating finding in a case of biliary colic. BMJ Case Rep. 2013;2013. Epub 20130926. doi: 10.1136/bcr-2013-200539. PubMed PMID: 24072833; PubMed Central PMCID: PMCPMC3794215.	Excluded from the final analysis	The article was excluded from the analysis because it was repetitive (duplicate article)
1254	Grossman SJ, Joyce JM. Hepatobiliary imaging. Emerg Med Clin North Am. 1991;9(4):853-74. PubMed PMID: 1915052.	Excluded from the final analysis	The article was excluded from the analysis because it was repetitive (duplicate article)

1255	Grundy SM. Recent nutrition research: implications for foods of the future. Ann Med. 1991;23(2):187-93. doi: 10.3109/07853899109148046. PubMed PMID: 2069795.	Excluded from the final analysis	The article was excluded from the analysis because it was repetitive (duplicate article)
1256	Guzmán HM, Sepúlveda M, Rosso N, San Martin A, Guzmán F, Guzmán HC. Incidence and Risk Factors for Cholelithiasis After Bariatric Surgery. Obes Surg. 2019;29(7):2110-4. doi: 10.1007/s11695-019-03760-4. PubMed PMID: 31001756.	Excluded from the final analysis	The article was excluded from the analysis because it was repetitive (duplicate article)
1257	Hackert T, Hartwig W, Fritz S, Schneider L, Strobel O, Werner J. Ischemic acute pancreatitis: clinical features of 11 patients and review of the literature. Am J Surg. 2009;197(4):450-4. Epub 20080907. doi: 10.1016/j.amjsurg.2008.04.011. PubMed PMID: 18778810.	Excluded from the final analysis	The article was excluded from the analysis because it was repetitive (duplicate article)
1258	Hadi A, Werge M, Kristiansen KT, Pedersen UG, Karstensen JG, Novovic S, Gluud LL. Coronavirus Disease-19 (COVID-19) associated with severe acute pancreatitis: Case report on three family members. Pancreatology. 2020;20(4):665-7. Epub 20200505. doi: 10.1016/j.pan.2020.04.021. PubMed PMID: 32387082; PubMed Central PMCID: PMCPMC7199002.	Excluded from the final analysis	The article was excluded from the analysis because it was repetitive (duplicate article)
1259	Haider G, Hussain D, Waheed S, Shah R, Khan AA, Ibrahim M, Shah SM. LAPAROSCOPIC CHOLECYSTECTOMY: OUTCOME OF FIRST 202 CASES IN A DISTRICT HOSPITAL IN GILGIT. J Ayub Med Coll Abbottabad. 2015;27(3):689-91. PubMed PMID: 26721041.	Excluded from the final analysis	The article was excluded from the analysis because it was repetitive (duplicate article)
1260	Hainer V, Kunesová M, Parízková J, Stunkard A. [Health risks and economic costs associated with obesity requiring a comprehensive weight reduction program]. Cas Lek Cesk. 1997;136(12):367-72. PubMed PMID: 9333508.	Excluded from the final analysis	The article was excluded from the analysis because it was repetitive (duplicate article)
1261	Hanefeld M, Hora C, Schulze J, Rothe G, Barthel U, Haller H. Reduced incidence of cardiovascular complications and mortality in hyperlipoproteinemia (HLP) with effective lipid correction. The Dresden HLP study. Atherosclerosis. 1984;53(1):47-58. doi: 10.1016/0021-9150(84)90104-7. PubMed PMID: 6497944.	Excluded from the final analysis	The article was excluded from the analysis because it was repetitive (duplicate article)
1262	Hanis CL, Hewett-Emmett D, Kubrusly LF, Maklad MN, Douglas TC, Mueller WH, et al. An ultrasound survey of gallbladder disease among Mexican Americans in Starr County, Texas: frequencies and risk factors. Ethn Dis. 1993;3(1):32-43. PubMed PMID: 8508103.	Excluded from the final analysis	The article was excluded from the analysis because it was repetitive (duplicate article)

1263	Harooni J, Joukar F, Goujani R, Sikaroudi MK, Hatami A, Zolghadrpour MA, et al. Cohort profile: the PERSIAN Dena Cohort Study (PDCS) of non-communicable diseases in Southwest Iran. BMJ Open. 2024;14(4):e079697. Epub 20240410. doi: 10.1136/bmjopen-2023-079697. PubMed PMID: 38604628; PubMed Central PMCID: PMCPMC11015286.	Excluded from the final analysis	The article was excluded from the analysis because it was repetitive (duplicate article)
1264	Harvey MH, Wedgwood KR, Austin JA, Reber HA. Pancreatic duct pressure, duct permeability and acute pancreatitis. Br J Surg. 1989;76(8):859-62. doi: 10.1002/bjs.1800760832. PubMed PMID: 2475200.	Excluded from the final analysis	The article was excluded from the analysis because it was repetitive (duplicate article)
1265	Hasan B, Asif T, Braun C, Bahaj W, Dosokey E, Pauly RR. Pancreatitis in the Setting of Vaso-occlusive Sickle Cell Crisis: A Rare Encounter. Cureus. 2017;9(4):e1193. Epub 20170425. doi: 10.7759/cureus.1193. PubMed PMID: 28553571; PubMed Central PMCID: PMCPMC5444912.	Excluded from the final analysis	The article was excluded from the analysis because it was repetitive (duplicate article)
1266	Hernández-Rodríguez J, Tan CD, Rodríguez ER, Hoffman GS. Single-organ gallbladder vasculitis: characterization and distinction from systemic vasculitis involving the gallbladder. An analysis of 61 patients. Medicine (Baltimore). 2014;93(24):405-13. doi: 10.1097/md.0000000000000205. PubMed PMID: 25500710; PubMed Central PMCID: PMCPMC4602437.	Excluded from the final analysis	The article was excluded from the analysis because it was repetitive (duplicate article)
1267	Hess W. [WHEN TO OPERATE IN DISEASES OF THE BILIARY TRACT AND PANCREAS]. Dia Med. 1963;35:1737-46. PubMed PMID: 14095886.	Excluded from the final analysis	The article was excluded from the analysis because it was repetitive (duplicate article)
1268	Higashizono K, Nakatani E, Hawke P, Fujimoto S, Oba N. Risk factors for gallstone disease onset in Japan: Findings from the Shizuoka Study, a population-based cohort study. PLoS One. 2022;17(12):e0274659. Epub 20221230. doi: 10.1371/journal.pone.0274659. PubMed PMID: 36584097; PubMed Central PMCID: PMCPMC9803237.	Excluded from the final analysis	The article was excluded from the analysis because it was repetitive (duplicate article)
1269	Hirano T, Manabe T. A possible mechanism for gallstone pancreatitis: repeated short-term pancreaticobiliary duct obstruction with exocrine stimulation in rats. Proc Soc Exp Biol Med. 1993;202(2):246-52. doi: 10.3181/00379727-202-43534. PubMed PMID: 7678705.	Excluded from the final analysis	The article was excluded from the analysis because it was repetitive (duplicate article)

1270	Hirano T, Manabe T. A new experimental model for gallstone pancreatitis: short-termed pancreatico-biliary duct obstruction and exocrine stimulation with systemic hypotension in rats. Nihon Geka Hokan. 1993;62(1):3-15. PubMed PMID: 8352638.	Excluded from the final analysis	The article was excluded from the analysis because it was repetitive (duplicate article)
1271	Hirano T, Manabe T. Effect of short-term pancreatico-biliary duct obstruction with intraductal hypertension on subcellular organelle fragility and pancreatic adenylate energy metabolism in rats: protective effect of a new protease inhibitor, E-3123. Acta Chir Belg. 1994;94(2):80-5. PubMed PMID: 7517090.	Excluded from the final analysis	The article was excluded from the analysis because it was repetitive (duplicate article)
1272	Hodge AM, English DR, Giles GG, Flicker L. Social connectedness and predictors of successful ageing. Maturitas. 2013;75(4):361-6. Epub 20130606. doi: 10.1016/j.maturitas.2013.05.002. PubMed PMID: 23746413.	Excluded from the final analysis	The article was excluded from the analysis because it was repetitive (duplicate article)
1273	Hofmann AF, Amelsberg A, Esch O, Schteingart CD, Lyche K, Jinich H, et al. Successful topical dissolution of cholesterol gallbladder stones using ethyl propionate. Dig Dis Sci. 1997;42(6):1274-82. doi: 10.1023/a:1018818527187. PubMed PMID: 9201095.	Excluded from the final analysis	The article was excluded from the analysis because it was repetitive (duplicate article)
1274	Hoshi Y, Takeshima K, Matsuoka S, Hoshikawa T, Senuma K, Nakamura T, et al. Survival in a Case of Emphysematous Cholecystitis With Sepsis Caused by Clostridium perfringens. Cureus. 2023;15(11):e49705. Epub 20231130. doi: 10.7759/cureus.49705. PubMed PMID: 38033444; PubMed Central PMCID: PMCPMC10688239.	Excluded from the final analysis	The article was excluded from the analysis because it was repetitive (duplicate article)
1275	Hoyme M, Surber R, Schulze PC, Prochnau D. [Acute chest pain and new ECG changes in pacemaker patients : A clinical challenge]. Herzschrittmacherther Elektrophysiol. 2017;28(1):60-3. Epub 20170215. doi: 10.1007/s00399-017-0491-5. PubMed PMID: 28204915.	Excluded from the final analysis	The article was excluded from the analysis because it was repetitive (duplicate article)
1276	Huang JL, Chen WK, Lin CL, Kao CH, Shih HM. Preeclampsia and the Risk of Pancreatitis: A Nationwide, Population-Based Cohort Study. Gastroenterol Res Pract. 2020;2020:3261542. Epub 20201230. doi: 10.1155/2020/3261542. PubMed PMID: 33456459; PubMed Central PMCID: PMCPMC7787823.	Excluded from the final analysis	The article was excluded from the analysis because it was repetitive (duplicate article)
1277	Huang X, Chen M, Tan H, Xiao S, Deng J. The morbidity rate of chronic disease among Chinese rural residents: results from Liuyang cohort. Med Princ Pract. 2013;22(4):362-7. Epub 20130125. doi: 10.1159/000346437. PubMed PMID: 23364269; PubMed Central PMCID: PMCPMC5586756.	Excluded from the final analysis	The article was excluded from the analysis because it was repetitive (duplicate article)

1278	Hughes DA, Pastores GM. Gaucher Disease. In: Adam MP, Feldman J, Mirzaa GM, Pagon RA, Wallace SE, Amemiya A, editors. GeneReviews(®). Seattle (WA): University of Washington, Seattle	Excluded from the final analysis	The article was excluded from the analysis because it was repetitive (duplicate article)
1279	Huh JH, Lee KJ, Cho YK, Moon S, Kim YJ, Han KD, et al. Cholecystectomy increases the risk of metabolic syndrome in the Korean population: a longitudinal cohort study. Hepatobiliary Surg Nutr. 2023;12(4):523-33. Epub 20221206. doi: 10.21037/hbsn-22-201. PubMed PMID: 37600984; PubMed Central PMCID: PMCPMC10432299.	Excluded from the final analysis	The article was excluded from the analysis because it was repetitive (duplicate article)
1280	Hung MC, Chen CF, Tsou MT, Lin HH, Hwang LC, Hsu CP. Relationship Between Gallstone Disease and Cardiometabolic Risk Factors in Elderly People with Non-Alcoholic Fatty Liver Disease. Diabetes Metab Syndr Obes. 2020;13:3579-85. Epub 20201009. doi: 10.2147/dmso.S266947. PubMed PMID: 33116709; PubMed Central PMCID: PMCPMC7553650.	Excluded from the final analysis	The article was excluded from the analysis because it was repetitive (duplicate article)
1281	Husain NE, Hassan AT, Elmadhoun WM, Ahmed MH. Evaluating the safety of Liptruzet (ezetimibe and atorvastatin): what are the potential benefits beyond low-density lipoprotein cholesterol-lowering effect? Expert Opin Drug Saf. 2015;14(9):1445-55. Epub 20150703. doi: 10.1517/14740338.2015.1063613. PubMed PMID: 26134926.	Excluded from the final analysis	The article was excluded from the analysis because it was repetitive (duplicate article)
1282	Huth K, Burkard M, Goebel T. [Dyslipoproteinemia and diabetes mellitus in a metabolic syndrome]. Fortschr Med. 1992;110(11):200-4. PubMed PMID: 1601361.	Excluded from the final analysis	The article was excluded from the analysis because it was repetitive (duplicate article)
1283	Imbelloni LE, Sant'anna R, Fornasari M, Fialho JC. Laparoscopic cholecystectomy under spinal anesthesia: comparative study between conventional-dose and low-dose hyperbaric bupivacaine. Local Reg Anesth. 2011;4:41-6. Epub 20111003. doi: 10.2147/lra.S19979. PubMed PMID: 22915892; PubMed Central PMCID: PMCPMC3417972.	Excluded from the final analysis	The article was excluded from the analysis because it was repetitive (duplicate article)
1284	Ionescu DL. [The gallstone and arterial hypertension]. Rev Med Chir Soc Med Nat Iasi. 2001;105(1):101-4. PubMed PMID: 12092132.	Excluded from the final analysis	The article was excluded from the analysis because it was repetitive (duplicate article)

1285	Isam SM, Ismail AA, Mohamed I, Suliman FS. Laparoscopic cholecystectomy in patients with bilharzial portal hypertension. Jsls. 2000;4(2):155-7. PubMed PMID: 10917123; PubMed Central PMCID: PMCPMC3015381.	Excluded from the final analysis	The article was excluded from the analysis because it was repetitive (duplicate article)
1286	Itagaki H, Hagino T. Vitamin K deficiency-induced hemorrhagic shock after thoracentesis: a case report. BMC Gastroenterol. 2019;19(1):58. Epub 20190418. doi: 10.1186/s12876-019-0978-0. PubMed PMID: 30999880; PubMed Central PMCID: PMCPMC6472080.	Excluded from the final analysis	The article was excluded from the analysis because it was repetitive (duplicate article)
1287	J F, C S, A RF, M LG, M G. Myotonic Dystrophy-2: Unusual Phenotype Due to a Small CCTG-expansion. Balkan J Med Genet. 2018;21(2):39-43. Epub 20181231. doi: 10.2478/bjmg-2018-0024. PubMed PMID: 30984523; PubMed Central PMCID: PMCPMC6454246.	Excluded from the final analysis	The article was excluded from the analysis because it was repetitive (duplicate article)
1288	Jang H, Park CH, Park Y, Jeong E, Lee N, Kim J, Jo Y. Spontaneous resolution of gallbladder hematoma in blunt traumatic injury: A case report. World J Clin Cases. 2021;9(28):8518-23. doi: 10.12998/wjcc.v9.i28.8518. PubMed PMID: 34754862; PubMed Central PMCID: PMCPMC8554416.	Excluded from the final analysis	The article was excluded from the analysis because it was repetitive (duplicate article)
1289	Janjua TK, Siddique S, Ibrahim MF, Khurshaidi MN. Aeromonas hydrophila induced necrotizing fasciitis following laparoscopic cholecystectomy. J Pak Med Assoc. 2024;74(3):576-9. doi: 10.47391/jpma.9344. PubMed PMID: 38591302.	Excluded from the final analysis	The article was excluded from the analysis because it was repetitive (duplicate article)
1290	Jarbou SM, al-Hammouri FA. Gallstone ileus in the Jordanian Royal Medical Services in a 10-year period. East Mediterr Health J. 2000;6(5-6):1117-21. PubMed PMID: 12197337.	Excluded from the final analysis	The article was excluded from the analysis because it was repetitive (duplicate article)
1291	Jeffrey GP, Brind AM, Ormonde DG, Frazer CK, Ferguson J, Bell R, et al. Management of biliary tract complications following liver transplantation. Aust N Z J Surg. 1999;69(10):717-22. doi: 10.1046/j.1440-1622.1999.01671.x. PubMed PMID: 10527348.	Excluded from the final analysis	The article was excluded from the analysis because it was repetitive (duplicate article)
1292	Jensen SL, Jensen PF, Wara P, Rokkjaer M. [Laparoscopic cholecystectomy. The first 45 operations]. Ugeskr Laeger. 1991;153(46):3225-8. PubMed PMID: 1835552.	Excluded from the final analysis	The article was excluded from the analysis because it was repetitive (duplicate article)

1293	Jeong YH, Kim KO, Lee HC, Sohn SH, Lee JW, Lee SH, et al. Gallstone prevalence and risk factors in patients with ulcerative colitis in Korean population. Medicine (Baltimore). 2017;96(31):e7653. doi: 10.1097/md.0000000000007653. PubMed PMID: 28767582; PubMed Central PMCID: PMCPMC5626136.	Excluded from the final analysis	The article was excluded from the analysis because it was repetitive (duplicate article)
1294	Jia G, Shu XO, Liu Y, Li HL, Cai H, Gao J, et al. Association of Adult Weight Gain With Major Health Outcomes Among Middle-aged Chinese Persons With Low Body Weight in Early Adulthood. JAMA Netw Open. 2019;2(12):e1917371. Epub 20191202. doi: 10.1001/jamanetworkopen.2019.17371. PubMed PMID: 31834393; PubMed Central PMCID: PMCPMC6991199.	Excluded from the final analysis	The article was excluded from the analysis because it was repetitive (duplicate article)
1295	Jiang BQ, Zhong PH, Cheng XB, Yang XL, Yang J, Cao YF. Investigation of health and nutrition status of middle-aged and old residents in the urban district of Chongqing. Asia Pac J Clin Nutr. 2007;16 Suppl 1:17-21. PubMed PMID: 17392070.	Excluded from the final analysis	The article was excluded from the analysis because it was repetitive (duplicate article)
1296	Jolly K, Chambers R. Improving outcomes for patients with obesity. Practitioner. 2014;258(1773):29-31, 3. PubMed PMID: 25211791.	Excluded from the final analysis	The article was excluded from the analysis because it was repetitive (duplicate article)
1297	Kakhktsyan T, Aleksanyan M, Acob T, Hakobyan K. A Case of Primary Biliary Cholangitis in a Patient With Multiple Sclerosis. Cureus. 2024;16(7):e63812. Epub 20240704. doi: 10.7759/cureus.63812. PubMed PMID: 39100035; PubMed Central PMCID: PMCPMC11297683.	Excluded from the final analysis	The article was excluded from the analysis because it was repetitive (duplicate article)
1298	Kano N, Yamakawa T, Ishikawa Y, Sakai S, Honda H, Kasugai H, Tachibana A. Laparoscopic cholecystectomy: a report of 409 consecutive cases and its future outlook. Surg Today. 1994;24(5):399-402. doi: 10.1007/bf01427031. PubMed PMID: 8054809.	Excluded from the final analysis	The article was excluded from the analysis because it was repetitive (duplicate article)
1299	Kara YB, Ozel Y. Comparison of the Grades of Fatty Liver Disease With Perioperative Risk Factors in Patients With Laparoscopic Sleeve Gastrectomy. Cureus. 2024;16(9):e69717. Epub 20240919. doi: 10.7759/cureus.69717. PubMed PMID: 39429298; PubMed Central PMCID: PMCPMC11490200.	Excluded from the final analysis	The article was excluded from the analysis because it was repetitive (duplicate article)
1300	Karayalcin G, Rosner F, Kim KY, Chandra P, Aballi AJ. Sickle cell anemia- clinical manifestations in 100 patients and review of the literature. Am J Med Sci. 1975;269(1):51-68. doi: 10.1097/00000441-197501000-00007. PubMed PMID: 1130436.	Excluded from the final analysis	The article was excluded from the analysis because it was repetitive (duplicate article)

1301	Kasana V, Rajesh S, Chauhan U, Bihari C, Choudhury A, Sarin SK. Inflammatory Myofibroblastic Tumor of Liver Masquerading as Focal Nodular Hyperplasia in a Patient with Non-Cirrhotic Portal Hypertension and Biliary Pancreatitis. Indian J Surg Oncol. 2016;7(1):110-4. Epub 20150220. doi: 10.1007/s13193-015-0381-4. PubMed PMID: 27065695; PubMed Central PMCID: PMCPMC4811821.	Excluded from the final analysis	The article was excluded from the analysis because it was repetitive (duplicate article)
1302	Kaura SH, Haghighi M, Matza BW, Hajdu CH, Rosenkrantz AB. Comparison of CT and MRI findings in the differentiation of acute from chronic cholecystitis. Clin Imaging. 2013;37(4):687-91. Epub 20130328. doi: 10.1016/j.clinimag.2013.02.009. PubMed PMID: 23541278.	Excluded from the final analysis	The article was excluded from the analysis because it was repetitive (duplicate article)
1303	Kaushik N, Dasari V, Jain D. Management of Pancreatic Calculi in Chronic Pancreatitis: A Review Article. Cureus. 2023;15(3):e35788. Epub 20230305. doi: 10.7759/cureus.35788. PubMed PMID: 37025704; PubMed Central PMCID: PMCPMC10072785.	Excluded from the final analysis	The article was excluded from the analysis because it was repetitive (duplicate article)
1304	Kelly DA. Intestinal failure-associated liver disease: what do we know today? Gastroenterology. 2006;130(2 Suppl 1):S70-7. doi: 10.1053/j.gastro.2005.10.066. PubMed PMID: 16473076.	Excluded from the final analysis	The article was excluded from the analysis because it was repetitive (duplicate article)
1305	Kelly DA. Preventing parenteral nutrition liver disease. Early Hum Dev. 2010;86(11):683-7. doi: 10.1016/j.earlhumdev.2010.08.012. PubMed PMID: 20923719.	Excluded from the final analysis	The article was excluded from the analysis because it was repetitive (duplicate article)
1306	Kelly SB, Gauhar T, Pollard R. Massive intraperitoneal hemorrhage from a pancreatic pseudocyst. Am J Gastroenterol. 1999;94(12):3638-41. doi: 10.1111/j.1572-0241.1999.01459.x. PubMed PMID: 10606335.	Excluded from the final analysis	The article was excluded from the analysis because it was repetitive (duplicate article)
1307	Keltikangas-Järvinen L. Concept of alexithymia. I. The prevalence of alexithymia in psychosomatic patients. Psychother Psychosom. 1985;44(3):132-8. doi: 10.1159/000287904. PubMed PMID: 3832145.	Excluded from the final analysis	The article was excluded from the analysis because it was repetitive (duplicate article)
1308	Keltikangas-Järvinen L. The prevalence and construct validity of type A behaviour in patients with duodenal ulcers. Br J Med Psychol. 1987;60 (Pt 2):163-7. doi: 10.1111/j.2044-8341.1987.tb02727.x. PubMed PMID: 3620393.	Excluded from the final analysis	The article was excluded from the analysis because it was repetitive (duplicate article)

1309	Keltikangas-Järvinen L. 'Psychosomatic personality'--a personality constellation or an illness-related reaction? Br J Med Psychol. 1989;62 (Pt 4):325-31. doi: 10.1111/j.2044-8341.1989.tb02842.x. PubMed PMID: 2597648.	Excluded from the final analysis	The article was excluded from the analysis because it was repetitive (duplicate article)
1310	Khan AR. Open laparoscopic access for primary trocar using modified Hasson's technique. Saudi Med J. 2003;24 Suppl:S21-4. PubMed PMID: 12778236.	Excluded from the final analysis	The article was excluded from the analysis because it was repetitive (duplicate article)
1311	Khurram M, Paracha SJ, Khar HT, Hasan Z. Obesity related complications in 100 obese subjects and their age matched controls. J Pak Med Assoc. 2006;56(2):50-3. PubMed PMID: 16555633.	Excluded from the final analysis	The article was excluded from the analysis because it was repetitive (duplicate article)
1312	Kim BH, Lee SH, Lee SS, Oh DJ, Chung ES, Lee SJ. Diverticular disease of the colon in Korea. Korean J Intern Med. 1987;2(1):79-83. doi: 10.3904/kjim.1987.2.1.79. PubMed PMID: 3154819; PubMed Central PMCID: PMCPMC4534912.	Excluded from the final analysis	The article was excluded from the analysis because it was repetitive (duplicate article)
1313	Kim KO, Kim TN, Lee SH. Endoscopic papillary large balloon dilation for the treatment of recurrent bile duct stones in patients with prior sphincterotomy. J Gastroenterol. 2010;45(12):1283-8. Epub 20100716. doi: 10.1007/s00535-010-0284-7. PubMed PMID: 20635102.	Excluded from the final analysis	The article was excluded from the analysis because it was repetitive (duplicate article)
1314	Kim SS, Lee JG, Kim DW, Kim BH, Jeon YK, Kim MR, et al. Insulin resistance as a risk factor for gallbladder stone formation in Korean postmenopausal women. Korean J Intern Med. 2011;26(3):285-93. Epub 20110913. doi: 10.3904/kjim.2011.26.3.285. PubMed PMID: 22016589; PubMed Central PMCID: PMCPMC3192201.	Excluded from the final analysis	The article was excluded from the analysis because it was repetitive (duplicate article)
1315	Kim SY, Bang WJ, Lim H, Lim MS, Kim M, Choi HG. Increased risk of gallstones after gastrectomy: A longitudinal follow-up study using a national sample cohort in korea. Medicine (Baltimore). 2019;98(22):e15932. doi: 10.1097/md.0000000000015932. PubMed PMID: 31145363; PubMed Central PMCID: PMCPMC6709130.	Excluded from the final analysis	The article was excluded from the analysis because it was repetitive (duplicate article)
1316	Kim SY, Lim H, Park B, Lim H, Kim M, Kong IG, Choi HG. Increased risk of gallstones after appendectomy: A longitudinal follow-up study using a national sample cohort. Medicine (Baltimore). 2020;99(20):e20269. doi: 10.1097/md.0000000000020269. PubMed PMID: 32443372; PubMed Central PMCID: PMCPMC7253851.	Excluded from the final analysis	The article was excluded from the analysis because it was repetitive (duplicate article)

1317	Kim SY, Song CM, Lim H, Lim MS, Bang W, Choi HG. Bidirectional association between gallstones and renal stones: Two longitudinal follow-up studies using a national sample cohort. Sci Rep. 2019;9(1):2620. Epub 20190222. doi: 10.1038/s41598-019-38964-2. PubMed PMID: 30796254; PubMed Central PMCID: PMCPMC6385337.	Excluded from the final analysis	The article was excluded from the analysis because it was repetitive (duplicate article)
1318	Klurfeld DM. The role of dietary fiber in gastrointestinal disease. J Am Diet Assoc. 1987;87(9):1172-7. PubMed PMID: 3040840.	Excluded from the final analysis	The article was excluded from the analysis because it was repetitive (duplicate article)
1319	Knobel B, Rosman P, Gewurtz G. Bilateral hydronephrosis due to fecaloma in an elderly woman. J Clin Gastroenterol. 2000;30(3):311-3. doi: 10.1097/00004836-200004000-00022. PubMed PMID: 10777195.	Excluded from the final analysis	The article was excluded from the analysis because it was repetitive (duplicate article)
1320	Ko JS, Yi NJ, Suh KS, Seo JK. Pediatric liver transplantation for fibropolycystic liver disease. Pediatr Transplant. 2012;16(2):195-200. doi: 10.1111/j.1399-3046.2012.01661.x. PubMed PMID: 22360404.	Excluded from the final analysis	The article was excluded from the analysis because it was repetitive (duplicate article)
1321	Koller T, Kollerova J, Hlavaty T, Huorka M, Payer J. Cholelithiasis and markers of nonalcoholic fatty liver disease in patients with metabolic risk factors. Scand J Gastroenterol. 2012;47(2):197-203. Epub 20111219. doi: 10.3109/00365521.2011.643481. PubMed PMID: 22182015.	Excluded from the final analysis	The article was excluded from the analysis because it was repetitive (duplicate article)
1322	Kreft I, Vollmannová A, Lidiková J, Musilová J, Germ M, Golob A, et al. Molecular Shield for Protection of Buckwheat Plants from UV-B Radiation. Molecules. 2022;27(17). Epub 20220830. doi: 10.3390/molecules27175577. PubMed PMID: 36080352; PubMed Central PMCID: PMCPMC9457819.	Excluded from the final analysis	The article was excluded from the analysis because it was repetitive (duplicate article)
1323	Kudva MV, Zawawi MM. Chronic liver disease in Kuala Lumpur, Malaysia: a clinical study. Singapore Med J. 1990;31(4):368-73. PubMed PMID: 2255936.	Excluded from the final analysis	The article was excluded from the analysis because it was repetitive (duplicate article)
1324	Kumar M, Saraswat VA. Natural history of portal cavernoma cholangiopathy. J Clin Exp Hepatol. 2014;4(Suppl 1):S62-6. Epub 20130827. doi: 10.1016/j.jceh.2013.08.003. PubMed PMID: 25755597; PubMed Central PMCID: PMCPMC4244826.	Excluded from the final analysis	The article was excluded from the analysis because it was repetitive (duplicate article)

1325	Kumar S, Chauhan S. Splenectomy in Thalassemia: The Role of Surgery as an Adjunct to Medical Management. Cureus. 2024;16(6):e62834. Epub 20240621. doi: 10.7759/cureus.62834. PubMed PMID: 39036111; PubMed Central PMCID: PMCPMC11260393.	Excluded from the final analysis	The article was excluded from the analysis because it was repetitive (duplicate article)
1326	Kwak MS, Kim D, Chung GE, Kim W, Kim YJ, Yoon JH. Cholecystectomy is independently associated with nonalcoholic fatty liver disease in an Asian population. World J Gastroenterol. 2015;21(20):6287-95. doi: 10.3748/wjg.v21.i20.6287. PubMed PMID: 26034364; PubMed Central PMCID: PMCPMC4445106.	Excluded from the final analysis	The article was excluded from the analysis because it was repetitive (duplicate article)
1327	La Vecchia C, Decarli A, Pagano R. Vegetable consumption and risk of chronic disease. Epidemiology. 1998;9(2):208-10. PubMed PMID: 9504293.	Excluded from the final analysis	The article was excluded from the analysis because it was repetitive (duplicate article)
1328	Lai HC, Chang SN, Lin CC, Chen CC, Chou JW, Peng CY, et al. Does diabetes mellitus with or without gallstones increase the risk of gallbladder cancer? Results from a population-based cohort study. J Gastroenterol. 2013;48(7):856-65. Epub 20121016. doi: 10.1007/s00535-012-0683-z. PubMed PMID: 23065035.	Excluded from the final analysis	The article was excluded from the analysis because it was repetitive (duplicate article)
1329	Lakshmi MV, Sridharan GV, Butterworth D. Gallstone cirrhosis: are we only seeing the tip of the iceberg? Br J Clin Pract. 1993;47(3):164-5. PubMed PMID: 8347447.	Excluded from the final analysis	The article was excluded from the analysis because it was repetitive (duplicate article)
1330	Lans CA. Ethnomedicines used in Trinidad and Tobago for urinary problems and diabetes mellitus. J Ethnobiol Ethnomed. 2006;2:45. Epub 20061013. doi: 10.1186/1746-4269-2-45. PubMed PMID: 17040567; PubMed Central PMCID: PMCPMC1624823.	Excluded from the final analysis	The article was excluded from the analysis because it was repetitive (duplicate article)
1331	Latenstein CSS, Alferink LJM, Darwish Murad S, Drenth JPH, van Laarhoven C, de Reuver PR. The Association Between Cholecystectomy, Metabolic Syndrome, and Nonalcoholic Fatty Liver Disease: A Population-Based Study. Clin Transl Gastroenterol. 2020;11(4):e00170. doi: 10.14309/ctg.0000000000000170. PubMed PMID: 32352682; PubMed Central PMCID: PMCPMC7263655.	Excluded from the final analysis	The article was excluded from the analysis because it was repetitive (duplicate article)
1332	Launay D, Hatron PY, Queyrel V, Leteurtre E, Beaulieu S, Lemann M, et al. [Unusual association: hepatic peliosis and Crohn's disease]. Rev Med Interne. 2002;23(2):198-202. doi: 10.1016/s0248-8663(01)00537-9. PubMed PMID: 11876065.	Excluded from the final analysis	The article was excluded from the analysis because it was repetitive (duplicate article)

1333	Leandros E, Albanopoulos K, Tsigris C, Archontovasilis F, Panoussopoulos SG, Skalistira M, et al. Laparoscopic cholecystectomy in cirrhotic patients with symptomatic gallstone disease. ANZ J Surg. 2008;78(5):363-5. doi: 10.1111/j.1445-2197.2008.04478.x. PubMed PMID: 18380734.	Excluded from the final analysis	The article was excluded from the analysis because it was repetitive (duplicate article)
1334	Leandros E, Gomatos IP, Mami P, Kastellanos E, Albanopoulos K, Konstadoulakis MM. Elective laparoscopic cholecystectomy for symptomatic gallstone disease in patients receiving anticoagulant therapy. J Laparoendosc Adv Surg Tech A. 2005;15(4):357-60. doi: 10.1089/lap.2005.15.357. PubMed PMID: 16108736.	Excluded from the final analysis	The article was excluded from the analysis because it was repetitive (duplicate article)
1335	Lee SI, Lim BH, Heo WG, Kim YJ, Kim TH. Successful Removal of a Large Common Bile Duct Stone by Using Direct Peroral Cholangioscopy and Laser Lithotripsy in a Patient with Severe Kyphosis. Clin Endosc. 2016;49(4):395-8. Epub 20160322. doi: 10.5946/ce.2015.109. PubMed PMID: 27000425; PubMed Central PMCID: PMCPMC4977743.	Excluded from the final analysis	The article was excluded from the analysis because it was repetitive (duplicate article)
1336	Lee YC, Wu JS, Yang YC, Chang CS, Lu FH, Chang CJ. Moderate to severe, but not mild, nonalcoholic fatty liver disease associated with increased risk of gallstone disease. Scand J Gastroenterol. 2014;49(8):1001-6. Epub 20140703. doi: 10.3109/00365521.2014.920912. PubMed PMID: 24989169.	Excluded from the final analysis	The article was excluded from the analysis because it was repetitive (duplicate article)
1337	Lee YM, Kaplan MM. Medical treatment of primary sclerosing cholangitis. J Hepatobiliary Pancreat Surg. 1999;6(4):361-5. doi: 10.1007/s005340050131. PubMed PMID: 10664282.	Excluded from the final analysis	The article was excluded from the analysis because it was repetitive (duplicate article)
1338	Leitzmann C. Vegetarian diets: what are the advantages? Forum Nutr. 2005;(57):147-56. doi: 10.1159/000083787. PubMed PMID: 15702597.	Excluded from the final analysis	The article was excluded from the analysis because it was repetitive (duplicate article)
1339	Lello L, Raben TG, Yong SY, Tellier L, Hsu SDH. Genomic Prediction of 16 Complex Disease Risks Including Heart Attack, Diabetes, Breast and Prostate Cancer. Sci Rep. 2019;9(1):15286. Epub 20191025. doi: 10.1038/s41598-019-51258-x. PubMed PMID: 31653892; PubMed Central PMCID: PMCPMC6814833.	Excluded from the final analysis	The article was excluded from the analysis because it was repetitive (duplicate article)
1340	Leppäniemi A, Kemppainen E. Recent advances in the surgical management of necrotizing pancreatitis. Curr Opin Crit Care. 2005;11(4):349-52. doi: 10.1097/01.ccx.0000166398.50517.fb. PubMed PMID: 16015114.	Excluded from the final analysis	The article was excluded from the analysis because it was repetitive (duplicate article)

1341	Leuci D, Quinto V. [Usefulness of the follow-up in outpatients affected by liver cirrhosis]. Recenti Prog Med. 2008;99(1):32. PubMed PMID: 18389869.	Excluded from the final analysis	The article was excluded from the analysis because it was repetitive (duplicate article)
1342	Li HL, Xu B, Zheng W, Xu WH, Gao J, Shu XO, Xiang YB. [Epidemiological characteristics of obesity and its relation to chronic diseases among middle aged and elderly men]. Zhonghua Liu Xing Bing Xue Za Zhi. 2010;31(4):370-4. PubMed PMID: 20513277.	Excluded from the final analysis	The article was excluded from the analysis because it was repetitive (duplicate article)
1343	Li P, He S, Wu Y, Pang Y, Yang L, Shi J, et al. Retroperitoneal infantile hemangioma: a case report and literature review. Discov Oncol. 2024;15(1):373. Epub 20240827. doi: 10.1007/s12672-024-01260-1. PubMed PMID: 39190162; PubMed Central PMCID: PMCPMC11349721.	Excluded from the final analysis	The article was excluded from the analysis because it was repetitive (duplicate article)
1344	Li Y, Han H, You K, Ma C, Fan X. Investigating the association between blood cobalt and gallstones: a cross-sectional study utilizing NHANES data. Front Public Health. 2024;12:1363815. Epub 20240207. doi: 10.3389/fpubh.2024.1363815. PubMed PMID: 38384872; PubMed Central PMCID: PMCPMC10879586.	Excluded from the final analysis	The article was excluded from the analysis because it was repetitive (duplicate article)
1345	Liang J, Jiang Y, Huang Y, Huang Y, Liu F, Zhang Y, et al. Comorbidities and factors influencing frequent gout attacks in patients with gout: a cross-sectional study. Clin Rheumatol. 2021;40(7):2873-80. Epub 20210204. doi: 10.1007/s10067-021-05595-w. PubMed PMID: 33538925.	Excluded from the final analysis	The article was excluded from the analysis because it was repetitive (duplicate article)
1346	Liao Q, Chen Y, Peng Q, Li C. Relationship between triglyceride-glucose index and gallstones risk: a population-based study. Front Endocrinol (Lausanne). 2024;15:1420999. Epub 20240711. doi: 10.3389/fendo.2024.1420999. PubMed PMID: 39055055; PubMed Central PMCID: PMCPMC11269096.	Excluded from the final analysis	The article was excluded from the analysis because it was repetitive (duplicate article)
1347	Liao XH, Cao X, Liu J, Xie XH, Sun YH, Zhong BH. Prevalence and features of fatty liver detected by physical examination in Guangzhou. World J Gastroenterol. 2013;19(32):5334-9. doi: 10.3748/wjg.v19.i32.5334. PubMed PMID: 23983438; PubMed Central PMCID: PMCPMC3752569.	Excluded from the final analysis	The article was excluded from the analysis because it was repetitive (duplicate article)
1348	Liesemer K, Mullen N. Hypertensive emergency successfully treated with metoprolol: a case report. Pediatr Emerg Care. 2009;25(5):333-5. doi: 10.1097/PEC.0b013e3181a34816. PubMed PMID: 19444030.	Excluded from the final analysis	The article was excluded from the analysis because it was repetitive (duplicate article)

1349	Lightner AM, Kirkwood KS. Pathophysiology of gallstone pancreatitis. Front Biosci. 2001;6:E66-76. Epub 20011001. doi: 10.2741/lightner. PubMed PMID: 11578966.	Excluded from the final analysis	The article was excluded from the analysis because it was repetitive (duplicate article)
1350	Lin BB, Huang RH, Lin BL, Hong YK, Lin ME, He XJ. Associations between nephrolithiasis and diabetes mellitus, hypertension and gallstones: A meta-analysis of cohort studies. Nephrology (Carlton). 2020;25(9):691-9. Epub 20200706. doi: 10.1111/nep.13740. PubMed PMID: 32525230.	Excluded from the final analysis	The article was excluded from the analysis because it was repetitive (duplicate article)
1351	Lin JN, Yen CM, Liu CS, Tsai MS, Kuo KK. Hepatic Schistosomiasis japonica in a patient with gallstones and bile duct stones--a case report. Kaohsiung J Med Sci. 2001;17(8):437-40. PubMed PMID: 11715844.	Excluded from the final analysis	The article was excluded from the analysis because it was repetitive (duplicate article)
1352	Lin K, Ofori E, Lin AN, Lin S, Lin T, Rasheed A, et al. Hypothermia-Related Acute Pancreatitis. Case Rep Gastroenterol. 2018;12(2):217-23. Epub 20180531. doi: 10.1159/000489296. PubMed PMID: 29928186; PubMed Central PMCID: PMCPMC6006605.	Excluded from the final analysis	The article was excluded from the analysis because it was repetitive (duplicate article)
1353	Lin YC, Chen IC, Chen YJ, Lin CT, Chang JC, Wang TJ, et al. Association between HNF4A rs1800961 polymorphisms and gallstones in a Taiwanese population. J Gastroenterol Hepatol. 2024;39(2):305-11. Epub 20231206. doi: 10.1111/jgh.16426. PubMed PMID: 38058101.	Excluded from the final analysis	The article was excluded from the analysis because it was repetitive (duplicate article)
1354	Lipsett PA, Pitt HA. Surgical treatment of choledochal cysts. J Hepatobiliary Pancreat Surg. 2003;10(5):352-9. doi: 10.1007/s00534-002-0797-4. PubMed PMID: 14598135.	Excluded from the final analysis	The article was excluded from the analysis because it was repetitive (duplicate article)
1355	Liu JK, Braschi C, de Virgilio C, Ozao-Choy J, Kim DY, Moazzez A. Predictors of poor outcomes after cholecystectomy in gallstone pancreatitis: NSQIP analysis of 30-day morbidity and mortality. Langenbecks Arch Surg. 2022;408(1):5. Epub 20221231. doi: 10.1007/s00423-022-02731-1. PubMed PMID: 36585495.	Excluded from the final analysis	The article was excluded from the analysis because it was repetitive (duplicate article)
1356	Liu W, Hada T, Fukui K, Imanishi H, Matsuoka N, Iwasaki A, Higashino K. Familial hypocholinesterasemia found in a family and a new confirmed mutation. Intern Med. 1997;36(1):9-13. doi: 10.2169/internalmedicine.36.9. PubMed PMID: 9058093.	Excluded from the final analysis	The article was excluded from the analysis because it was repetitive (duplicate article)

1357	Liu X, Yan G, Xu B, Sun M. Association between monocyte-to-high-density lipoprotein-cholesterol ratio and gallstones in U.S. adults: findings from the National Health and Nutrition Examination Survey 2017-2020. Lipids Health Dis. 2024;23(1):173. Epub 20240607. doi: 10.1186/s12944-024-02166-1. PubMed PMID: 38849878; PubMed Central PMCID: PMCPMC11157827.	Excluded from the final analysis	The article was excluded from the analysis because it was repetitive (duplicate article)
1358	Lodha M, Chauhan AS, Puranik A, Meena SP, Badkur M, Chaudhary R, et al. Clinical Profile and Evaluation of Outcomes of Symptomatic Gallstone Disease in the Senior Citizen Population. Cureus. 2022;14(8):e28492. Epub 20220828. doi: 10.7759/cureus.28492. PubMed PMID: 36185904; PubMed Central PMCID: PMCPMC9513743.	Excluded from the final analysis	The article was excluded from the analysis because it was repetitive (duplicate article)
1359	Loehfelm TW, Tse JR, Jeffrey RB, Kamaya A. The utility of hepatic artery velocity in diagnosing patients with acute cholecystitis. Abdom Radiol (NY). 2018;43(5):1159-67. doi: 10.1007/s00261-017-1288-z. PubMed PMID: 28840272.	Excluded from the final analysis	The article was excluded from the analysis because it was repetitive (duplicate article)
1360	Loreno M, Travali S, Bucceri AM, Scalisi G, Virgilio C, Brogna A. Ultrasonographic study of gallbladder wall thickness and emptying in cirrhotic patients without gallstones. Gastroenterol Res Pract. 2009;2009:683040. Epub 20090810. doi: 10.1155/2009/683040. PubMed PMID: 19680454; PubMed Central PMCID: PMCPMC2723919.	Excluded from the final analysis	The article was excluded from the analysis because it was repetitive (duplicate article)
1361	Lorenz FJ, Beauchamp-Perez F, Manni A, Chung T, Goldenberg D, Goyal N. Analysis of Time to Diagnosis and Outcomes Among Adults With Primary Hyperparathyroidism. JAMA Netw Open. 2022;5(12):e2248332. Epub 20221201. doi: 10.1001/jamanetworkopen.2022.48332. PubMed PMID: 36574247; PubMed Central PMCID: PMCPMC9857508.	Excluded from the final analysis	The article was excluded from the analysis because it was repetitive (duplicate article)
1362	Lu F, Wang L, Chen Y, Zhong X, Huang Z. In vitro cultured calculus bovis attenuates cerebral ischaemia-reperfusion injury by inhibiting neuronal apoptosis and protecting mitochondrial function in rats. J Ethnopharmacol. 2020;263:113168. Epub 20200727. doi: 10.1016/j.jep.2020.113168. PubMed PMID: 32730869.	Excluded from the final analysis	The article was excluded from the analysis because it was repetitive (duplicate article)
1363	Luitel P, Paudel S, Thapaliya I, Dhungana S, Thapa N, Devkota S. Bile duct injury following laparoscopic cholecystectomy in a patient with a trifurcated hepatic duct: A case report. Int J Surg Case Rep.	Excluded from the final analysis	The article was excluded from the analysis because it was repetitive (duplicate article)

	2024;123:110145. Epub 20240810. doi: 10.1016/j.ijscr.2024.110145. PubMed PMID: 39241475; PubMed Central PMCID: PMCPMC11408799.		
1364	Luthar Z, Golob A, Germ M, Vombergar B, Kreft I. Tartary Buckwheat in Human Nutrition. Plants (Basel). 2021;10(4). Epub 20210405. doi: 10.3390/plants10040700. PubMed PMID: 33916396; PubMed Central PMCID: PMCPMC8066602.	Excluded from the final analysis	The article was excluded from the analysis because it was repetitive (duplicate article)
1365	Lykavieris P, Chardot C, Sokhn M, Gauthier F, Valayer J, Bernard O. Outcome in adulthood of biliary atresia: a study of 63 patients who survived for over 20 years with their native liver. Hepatology. 2005;41(2):366-71. doi: 10.1002/hep.20547. PubMed PMID: 15660386.	Excluded from the final analysis	The article was excluded from the analysis because it was repetitive (duplicate article)
1366	Lyon C, Clark DC. Diagnosis of acute abdominal pain in older patients. Am Fam Physician. 2006;74(9):1537-44. PubMed PMID: 17111893.	Excluded from the final analysis	The article was excluded from the analysis because it was repetitive (duplicate article)
1367	Ma X, Gao J, Li Y, Xie J, Feng Z, Jia X, Chen W. Transplantation of splenic tissue after splenectomy: A case report. Exp Ther Med. 2022;24(4):612. Epub 20220803. doi: 10.3892/etm.2022.11549. PubMed PMID: 36160907; PubMed Central PMCID: PMCPMC9468849.	Excluded from the final analysis	The article was excluded from the analysis because it was repetitive (duplicate article)
1368	Magnano San Lio R, Barchitta M, Maugeri A, Quartarone S, Basile G, Agodi A. Preoperative Risk Factors for Conversion from Laparoscopic to Open Cholecystectomy: A Systematic Review and Meta-Analysis. Int J Environ Res Public Health. 2022;20(1). Epub 20221227. doi: 10.3390/ijerph20010408. PubMed PMID: 36612732; PubMed Central PMCID: PMCPMC9819914.	Excluded from the final analysis	The article was excluded from the analysis because it was repetitive (duplicate article)
1369	Mahfouz MEM, Altowairqi ADM, Alghamdi HY, Alzahrani MSZ, Alqurashi AK, Alhuraity TH, Alqurashi AS. Prevalence and Factors Associated With Post-Cholecystectomy Syndrome in Saudi Arabia. Cureus. 2022;14(12):e32827. Epub 20221222. doi: 10.7759/cureus.32827. PubMed PMID: 36694499; PubMed Central PMCID: PMCPMC9864481.	Excluded from the final analysis	The article was excluded from the analysis because it was repetitive (duplicate article)
1370	Makutonin M, Moghatederi A, Newton S, Ma Y, Meltzer AC. Biliary colic in the emergency department: A state-wide analysis of one-year costs and clinical outcomes. Surg Open Sci. 2023;12:9-13. Epub 20230210. doi: 10.1016/j.sopen.2023.02.002. PubMed PMID: 36866121; PubMed Central PMCID: PMCPMC9971543.	Excluded from the final analysis	The article was excluded from the analysis because it was repetitive (duplicate article)

1371	Malik AM. Difficult laparoscopic cholecystectomies. Is conversion a sensible option? J Pak Med Assoc. 2015;65(7):698-700. PubMed PMID: 26160075.	Excluded from the final analysis	The article was excluded from the analysis because it was repetitive (duplicate article)
1372	Malik AM, Laghari AA, Talpur KA, Memon A, Pathan R, Memon JM. Laparoscopic cholecystectomy in the elderly patients. An experience at Liaquat University Hospital Jamshoro. J Ayub Med Coll Abbottabad. 2007;19(4):45-8. PubMed PMID: 18693597.	Excluded from the final analysis	The article was excluded from the analysis because it was repetitive (duplicate article)
1373	Malkan GH, Bhatia SJ, Bashir K, Khemani R, Abraham P, Gandhi MS, Radhakrishnan R. Cholangiopathy associated with portal hypertension: diagnostic evaluation and clinical implications. Gastrointest Endosc. 1999;49(3 Pt 1):344-8. doi: 10.1016/s0016-5107(99)70011-8. PubMed PMID: 10049418.	Excluded from the final analysis	The article was excluded from the analysis because it was repetitive (duplicate article)
1374	Mall-Haefeli M. [Internal medicine problems regarding contraception. Part I]. Schweiz Med Wochenschr. 1980;110(36):1314-9. PubMed PMID: 7444416.	Excluded from the final analysis	The article was excluded from the analysis because it was repetitive (duplicate article)
1375	Mallick B, Anand AC. Gallstone Disease in Cirrhosis-Pathogenesis and Management. J Clin Exp Hepatol. 2022;12(2):551-9. Epub 20210916. doi: 10.1016/j.jceh.2021.09.011. PubMed PMID: 35535063; PubMed Central PMCID: PMCPMC9077239.	Excluded from the final analysis	The article was excluded from the analysis because it was repetitive (duplicate article)
1376	Manz F. Hydration and disease. J Am Coll Nutr. 2007;26(5 Suppl):535s-41s. doi: 10.1080/07315724.2007.10719655. PubMed PMID: 17921462.	Excluded from the final analysis	The article was excluded from the analysis because it was repetitive (duplicate article)
1377	Marcovici I, Marzano D. Pregnancy-induced hypertension complicated by postpartum renal failure and pancreatitis: a case report. Am J Perinatol. 2002;19(4):177-9. doi: 10.1055/s-2002-28500. PubMed PMID: 12012278.	Excluded from the final analysis	The article was excluded from the analysis because it was repetitive (duplicate article)
1378	Markaki I, Konsoula A, Markaki L, Spernovasilis N, Papadakis M. Acute acalculous cholecystitis due to infectious causes. World J Clin Cases. 2021;9(23):6674-85. doi: 10.12998/wjcc.v9.i23.6674. PubMed PMID: 34447814; PubMed Central PMCID: PMCPMC8362504.	Excluded from the final analysis	The article was excluded from the analysis because it was repetitive (duplicate article)

1379	Marques VL, Gormezano NW, Bonfá E, Aikawa NE, Terreri MT, Pereira RM, et al. Pancreatitis Subtypes Survey in 852 Childhood-Onset Systemic Lupus Erythematosus Patients. J Pediatr Gastroenterol Nutr. 2016;62(2):328-34. doi: 10.1097/mpg.0000000000000990. PubMed PMID: 26418213.	Excluded from the final analysis	The article was excluded from the analysis because it was repetitive (duplicate article)
1380	Martin S, Tyrrell J, Thomas EL, Bown MJ, Wood AR, Beaumont RN, et al. Disease consequences of higher adiposity uncoupled from its adverse metabolic effects using Mendelian randomisation. Elife. 2022;11. Epub 20220125. doi: 10.7554/eLife.72452. PubMed PMID: 35074047; PubMed Central PMCID: PMCPMC8789289.	Excluded from the final analysis	The article was excluded from the analysis because it was repetitive (duplicate article)
1381	Masabanda-Celorio VE, Alvares-Sores ED, Lara-Orosco U. [Acute cholangitis secondary to periampullary duodenal diverticulum. Case report]. Rev Med Inst Mex Seguro Soc. 2023;61(2):234-8. Epub 20230301. PubMed PMID: 37201190; PubMed Central PMCID: PMCPMC10395870.	Excluded from the final analysis	The article was excluded from the analysis because it was repetitive (duplicate article)
1382	Mason EE, Printen KJ, Blommers TJ, Scott DH. Gastric bypass for obesity after ten years experience. Int J Obes. 1978;2(2):197-206. PubMed PMID: 711364.	Excluded from the final analysis	The article was excluded from the analysis because it was repetitive (duplicate article)
1383	Massarrat S, Paidlik A, Pittner P, Schmitz-Moormann P, Wurbs M. The role of certain habits and various diseases in the occurrence of gastritis. Hepatogastroenterology. 1983;30(6):249-53. PubMed PMID: 6676145.	Excluded from the final analysis	The article was excluded from the analysis because it was repetitive (duplicate article)
1384	Mazza GR, Youssefzadeh AC, Aberle LS, Anderson ZS, Mandelbaum RS, Ouzounian JG, et al. Pregnant patients undergoing cholecystectomy: nationwide assessment of clinical characteristics and outcomes. AJOG Glob Rep. 2024;4(1):100310. Epub 20240111. doi: 10.1016/j.xagr.2024.100310. PubMed PMID: 38304305; PubMed Central PMCID: PMCPMC10830852.	Excluded from the final analysis	The article was excluded from the analysis because it was repetitive (duplicate article)
1385	Mead E, Atkinson G, Richter B, Metzendorf MI, Baur L, Finer N, et al. Drug interventions for the treatment of obesity in children and adolescents. Cochrane Database Syst Rev. 2016;11(11):Cd012436. Epub 20161129. doi: 10.1002/14651858.Cd012436. PubMed PMID: 27899001; PubMed Central PMCID: PMCPMC6472619.	Excluded from the final analysis	The article was excluded from the analysis because it was repetitive (duplicate article)
1386	Mehmood Y, Al-Swailmi FK, Al-Enazi SA. Frequency of obesity and comorbidities in medical students. Pak J Med Sci. 2016;32(6):1528-32. doi: 10.12669/pjms.326.10492. PubMed PMID: 28083058; PubMed Central PMCID: PMCPMC5216314.	Excluded from the final analysis	The article was excluded from the analysis because it was repetitive (duplicate article)

1387	Mellon M, Schiller A, Nelson AL, Stohl HE. Awareness of Pregnancy-Associated Health Risks Among Pregnant Women and Male Partners Surveyed in a Prenatal Clinic. J Womens Health (Larchmt). 2020;29(3):376-82. Epub 20191024. doi: 10.1089/jwh.2018.7585. PubMed PMID: 31647358.	Excluded from the final analysis	The article was excluded from the analysis because it was repetitive (duplicate article)
1388	Mendoza A, Oliff S, Elias E. Hereditary haemorrhagic telangiectasia and secondary biliary cirrhosis. Eur J Gastroenterol Hepatol. 1995;7(10):999-1002. doi: 10.1097/00042737-199510000-00017. PubMed PMID: 8590149.	Excluded from the final analysis	The article was excluded from the analysis because it was repetitive (duplicate article)
1389	Meyer FP. ber die laquo;Omnipotenz>> der Chelattherapie. Forsch Komplementarmed. 1998;5(6):266-71. doi: 10.1159/000021151. PubMed PMID: 9973659.	Excluded from the final analysis	The article was excluded from the analysis because it was repetitive (duplicate article)
1390	Michek J, Wendsche P. [Operations in acute blockade of the common bile duct and the papilla]. Zentralbl Chir. 1983;108(16):1023-5. PubMed PMID: 6637211.	Excluded from the final analysis	The article was excluded from the analysis because it was repetitive (duplicate article)
1391	Mikou MM, Mouaffak Y, Benyacob A, Mosaddek A, Faroudy M, Ababou A, et al. [Haemocholecyst: a rare complication of anticoagulant treatment]. Ann Fr Anesth Reanim. 2004;23(7):733-6. doi: 10.1016/j.annfar.2004.02.055. PubMed PMID: 15324963.	Excluded from the final analysis	The article was excluded from the analysis because it was repetitive (duplicate article)
1392	Millán T, Freitte A, Masalleras C, Porte L, Vargas C. [Prevalence of chronic diseases in young postpartum women]. Rev Med Chil. 1992;120(2):210-4. PubMed PMID: 1340563.	Excluded from the final analysis	The article was excluded from the analysis because it was repetitive (duplicate article)
1393	Min SK, Kim SY, Park YJ, Lee W, Jung IM, Lee T, et al. Role of three-dimensional computed tomography venography as a powerful navigator for varicose vein surgery. J Vasc Surg. 2010;51(4):893-9. doi: 10.1016/j.jvs.2009.10.117. PubMed PMID: 20347685.	Excluded from the final analysis	The article was excluded from the analysis because it was repetitive (duplicate article)
1394	Mishra K, Behari A, Shukla P, Tsuchiya Y, Endoh K, Asai T, et al. Risk factors for gallbladder cancer development in northern India: A gallstones-matched, case-control study. Indian J Med Res. 2021;154(5):699-706. doi: 10.4103/ijmr.IJMR_201_19. PubMed PMID: 35532588; PubMed Central PMCID: PMCPMC9210525.	Excluded from the final analysis	The article was excluded from the analysis because it was repetitive (duplicate article)

1395	Mishra N. Pregnancy With Diabetic Ketoacidosis and Hypertriglyceridemia-Induced Acute Pancreatitis: The Enigmatic Triad. Cureus. 2023;15(12):e50862. Epub 20231220. doi: 10.7759/cureus.50862. PubMed PMID: 38249199; PubMed Central PMCID: PMCPMC10798904.	Excluded from the final analysis	The article was excluded from the analysis because it was repetitive (duplicate article)
1396	Mitchell E, Ranganathan S, McKiernan P, Squires RH, Strauss K, Soltys K, et al. Hepatic Parenchymal Injury in Crigler-Najjar Type I. J Pediatr Gastroenterol Nutr. 2018;66(4):588-94. doi: 10.1097/mpg.0000000000001843. PubMed PMID: 29176474.	Excluded from the final analysis	The article was excluded from the analysis because it was repetitive (duplicate article)
1397	Mohr-Sasson A, Schiff E, Suday RR, Hayman Z, Kleinbaum Y, Kalter-Farber A, et al. The Yield of Abdominal Ultrasound in the Evaluation of Elevated Liver Enzymes during the Second and the Third Trimester of Pregnancy. Gynecol Obstet Invest. 2017;82(5):517-20. Epub 20170519. doi: 10.1159/000453612. PubMed PMID: 28521326.	Excluded from the final analysis	The article was excluded from the analysis because it was repetitive (duplicate article)
1398	Morán S, Duque-López MX, Salmerón-Castro J, Rodríguez-Leal G, Martínez-Salgado H, Uribe M. Association between serum concentration of apolipoproteins A-I and B with gallbladder disease. Arch Med Res. 2003;34(3):194-9. doi: 10.1016/s0188-4409(03)00025-0. PubMed PMID: 14567398.	Excluded from the final analysis	The article was excluded from the analysis because it was repetitive (duplicate article)
1399	Morley JE. The aging gut: physiology. Clin Geriatr Med. 2007;23(4):757-67, v-vi. doi: 10.1016/j.cger.2007.06.002. PubMed PMID: 17923336.	Excluded from the final analysis	The article was excluded from the analysis because it was repetitive (duplicate article)
1400	Morosetti D, Picchi E, Calcagni A, Lamacchia F, Cavallo AU, Bozzi A, et al. Anomalous development of the inferior vena cava: Case reports of agenesis and hypoplasia. Radiol Case Rep. 2018;13(4):895-903. Epub 20180705. doi: 10.1016/j.radcr.2018.04.018. PubMed PMID: 29997719; PubMed Central PMCID: PMCPMC6037009.	Excluded from the final analysis	The article was excluded from the analysis because it was repetitive (duplicate article)
1401	Moschopoulos C, Bailly JM, Bruninx G, Delcour C. [Agenesis of the right lobe of the liver. Apropos of a case]. Ann Radiol (Paris). 1993;36(4):323-7. PubMed PMID: 8239475.	Excluded from the final analysis	The article was excluded from the analysis because it was repetitive (duplicate article)
1402	Mostofsky E, Mukamal KJ, Giovannucci EL, Stampfer MJ, Rimm EB. Key Findings on Alcohol Consumption and a Variety of Health Outcomes From the Nurses' Health Study. Am J Public Health. 2016;106(9):1586-91.	Excluded from the final analysis	The article was excluded from the analysis because it was repetitive (duplicate article)

	Epub 20160726. doi: 10.2105/ajph.2016.303336. PubMed PMID: 27459455; PubMed Central PMCID: PMCPMC4981808.		
1403	Mun EC, Blackburn GL, Matthews JB. Current status of medical and surgical therapy for obesity. Gastroenterology. 2001;120(3):669-81. doi: 10.1053/gast.2001.22430. PubMed PMID: 11179243.	Excluded from the final analysis	The article was excluded from the analysis because it was repetitive (duplicate article)
1404	Murphy MA, Joyce WP. Information for surgical patients: implications of the World Wide Web. Eur J Surg. 2001;167(10):728-33. doi: 10.1080/11024150152707699. PubMed PMID: 11775723.	Excluded from the final analysis	The article was excluded from the analysis because it was repetitive (duplicate article)
1405	Mutiri B, Etonyeaku AC, Aloufi M, Alsaud JS. A Review of the Management of Cholelithiasis at Buraydah Central Hospital in the Qassim Region, Saudi Arabia: A Cross-Sectional Study. Cureus. 2023;15(12):e50846. Epub 20231220. doi: 10.7759/cureus.50846. PubMed PMID: 38249213; PubMed Central PMCID: PMCPMC10798649.	Excluded from the final analysis	The article was excluded from the analysis because it was repetitive (duplicate article)
1406	Naeem M, Rahimnajjad NA, Rahimnajjad MK, Khurshid M, Ahmed QJ, Shahid SM, et al. Assessment of characteristics of patients with cholelithiasis from economically deprived rural Karachi, Pakistan. BMC Res Notes. 2012;5:334. Epub 20120628. doi: 10.1186/1756-0500-5-334. PubMed PMID: 22741543; PubMed Central PMCID: PMCPMC3438066.	Excluded from the final analysis	The article was excluded from the analysis because it was repetitive (duplicate article)
1407	Nagamine Y, Godai K, Oki H, Kanmura Y. Management of a centenarian who underwent emergency laparoscopic cholecystectomy under general anesthesia with subcostal transversus abdominis plane block. JA Clin Rep. 2016;2(1):24. Epub 20160923. doi: 10.1186/s40981-016-0050-9. PubMed PMID: 29497679; PubMed Central PMCID: PMCPMC5818852.	Excluded from the final analysis	The article was excluded from the analysis because it was repetitive (duplicate article)
1408	Napolitano L, Waku M, Di Bartolomeo N, Aceto L, Innocenti P. [Clinical study on laparoscopic approach to cholelithiasis in the elderly]. G Chir. 2004;25(8-9):301-3. PubMed PMID: 15560307.	Excluded from the final analysis	The article was excluded from the analysis because it was repetitive (duplicate article)
1409	Navarro SM, Chen S, Situ X, Corwin MT, Loehfelm T, Fananapazir G. Sonographic Assessment of Acute Versus Chronic Cholecystitis: An Ultrasound Probability Stratification Model. J Ultrasound Med.	Excluded from the final analysis	The article was excluded from the analysis because it was repetitive (duplicate article)

	2023;42(6):1257-65. Epub 20221201. doi: 10.1002/jum.16138. PubMed PMID: 36457230; PubMed Central PMCID: PMCPMC10191874.		
1410	Neilson A, Schneider H. Obesity and its comorbidities: present and future importance on health status in Switzerland. Soz Praventivmed. 2005;50(2):78-86. doi: 10.1007/s00038-004-4066-y. PubMed PMID: 15900960.	Excluded from the final analysis	The article was excluded from the analysis because it was repetitive (duplicate article)
1411	Nelly Manrique M, Frisancho O, Zumaeta E, Palomino A, Rodriguez C. [Obscure digestive bleeding by ileal carcinoid tumor]. Rev Gastroenterol Peru. 2011;31(1):81-6. PubMed PMID: 21544161.	Excluded from the final analysis	The article was excluded from the analysis because it was repetitive (duplicate article)
1412	Ni HY, Zhang ZH, Fu HZ. [Research and development of Fructus Gardeniae]. Zhongguo Zhong Yao Za Zhi. 2006;31(7):538-41. PubMed PMID: 16780151.	Excluded from the final analysis	The article was excluded from the analysis because it was repetitive (duplicate article)
1413	Nikfarjam M, Shen L, Fink MA, Muralidharan V, Starkey G, Jones RM, Christophi C. Percutaneous cholecystostomy for treatment of acute cholecystitis in the era of early laparoscopic cholecystectomy. Surg Laparosc Endosc Percutan Tech. 2013;23(5):474-80. doi: 10.1097/SLE.0b013e318290142d. PubMed PMID: 24105289.	Excluded from the final analysis	The article was excluded from the analysis because it was repetitive (duplicate article)
1414	Nio M, Ohi R, Shimaoka S, Iwami D, Sano N. [Long-term outcome of surgery for biliary atresia]. Nihon Geka Gakkai Zasshi. 1996;97(8):637-41. PubMed PMID: 8905814.	Excluded from the final analysis	The article was excluded from the analysis because it was repetitive (duplicate article)
1415	Nio M, Ohi R, Shimaoka S, Iwami D, Sano N. The outcome of surgery for biliary atresia and the current status of long-term survivors. Tohoku J Exp Med. 1997;181(1):235-44. doi: 10.1620/tjem.181.235. PubMed PMID: 9149360.	Excluded from the final analysis	The article was excluded from the analysis because it was repetitive (duplicate article)
1416	Nogoy DM, Padmanaban V, Balazero LL, Rosado J, Sifri ZC. Predictors of Difficult Laparoscopic Cholecystectomy on Humanitarian Missions to Peru Difficult Laparoscopic Cholecystectomy in Surgical Missions. J Surg Res. 2021;267:102-8. Epub 20210620. doi: 10.1016/j.jss.2021.04.020. PubMed PMID: 34157489.	Excluded from the final analysis	The article was excluded from the analysis because it was repetitive (duplicate article)

1417	Nojkov B, Cappell MS. Safety and efficacy of ERCP after recent myocardial infarction or unstable angina. Gastrointest Endosc. 2010;72(4):870-80. doi: 10.1016/j.gie.2010.06.024. PubMed PMID: 20883868.	Excluded from the final analysis	The article was excluded from the analysis because it was repetitive (duplicate article)
1418	Nomura Y, Sakai H, Akiba J, Hisaka T, Sato T, Goto Y, et al. Laparoscopic left hepatectomy for a patient with intrahepatic cholangiocarcinoma metastasis in the falciform ligament: a case report. BMC Surg. 2021;21(1):122. Epub 20210308. doi: 10.1186/s12893-021-01115-4. PubMed PMID: 33685435; PubMed Central PMCID: PMCPMC7941886.	Excluded from the final analysis	The article was excluded from the analysis because it was repetitive (duplicate article)
1419	Okumura K, Shoji F, Yoshida M, Mizuta A, Makino I, Higashi H. Severe sepsis caused by Aeromonas hydrophila in a patient using tocilizumab: a case report. J Med Case Rep. 2011;5:499. Epub 20111005. doi: 10.1186/1752-1947-5-499. PubMed PMID: 21970314; PubMed Central PMCID: PMCPMC3214171.	Excluded from the final analysis	The article was excluded from the analysis because it was repetitive (duplicate article)
1420	Oldenkamp CL, Kitamura K. Nonobstetric Surgical Emergencies in Pregnancy. Emerg Med Clin North Am. 2023;41(2):259-67. Epub 20230209. doi: 10.1016/j.emc.2023.01.001. PubMed PMID: 37024162.	Excluded from the final analysis	The article was excluded from the analysis because it was repetitive (duplicate article)
1421	Orzeł D, Żmijewski M, Bronkowska M. Impact of products from ground buckwheat added to balanced diets on biochemical blood markers in Wistar rats. Rocz Panstw Zakl Hig. 2015;66(3):239-44. PubMed PMID: 26400120.	Excluded from the final analysis	The article was excluded from the analysis because it was repetitive (duplicate article)
1422	Osborne DR. Porto-biliary fistula and portal hypertension due to gallstones. A case report. Acta Chir Scand. 1980;146(5):363-5. PubMed PMID: 7468067.	Excluded from the final analysis	The article was excluded from the analysis because it was repetitive (duplicate article)
1423	Ostrowska L, Stefańska E, Jastrzebska M, Adamska E, Wujek A, Waszczeniuk M. [Effects of dietary habits modifications on selected metabolic parameters during weight loss in obese persons]. Rocz Panstw Zakl Hig. 2012;63(1):83-90. PubMed PMID: 22642074.	Excluded from the final analysis	The article was excluded from the analysis because it was repetitive (duplicate article)
1424	Oudhoff JP, Timmermans DR, Bijnen AB, van der Wal G. Waiting for elective general surgery: physical, psychological and social consequences. ANZ J Surg. 2004;74(5):361-7. doi: 10.1111/j.1445-1433.2004.02998.x. PubMed PMID: 15144258.	Excluded from the final analysis	The article was excluded from the analysis because it was repetitive (duplicate article)

1425	Oudhoff JP, Timmermans DR, Knol DL, Bijnen AB, Van der Wal G. Waiting for elective surgery: effect on physical problems and postoperative recovery. ANZ J Surg. 2007;77(10):892-8. doi: 10.1111/j.1445-2197.2007.04268.x. PubMed PMID: 17803558.	Excluded from the final analysis	The article was excluded from the analysis because it was repetitive (duplicate article)
1426	Oudhoff JP, Timmermans DR, Knol DL, Bijnen AB, van der Wal G. Waiting for elective general surgery: impact on health related quality of life and psychosocial consequences. BMC Public Health. 2007;7:164. Epub 20070719. doi: 10.1186/1471-2458-7-164. PubMed PMID: 17640382; PubMed Central PMCID: PMCPMC1959190.	Excluded from the final analysis	The article was excluded from the analysis because it was repetitive (duplicate article)
1427	Oudhoff JP, Timmermans DR, Knol DL, Bijnen AB, Van der Wal G. Prioritising patients on surgical waiting lists: a conjoint analysis study on the priority judgements of patients, surgeons, occupational physicians, and general practitioners. Soc Sci Med. 2007;64(9):1863-75. Epub 20070226. doi: 10.1016/j.socscimed.2007.01.002. PubMed PMID: 17324491.	Excluded from the final analysis	The article was excluded from the analysis because it was repetitive (duplicate article)
1428	Oudhoff JP, Timmermans DR, Rietberg M, Knol DL, van der Wal G. The acceptability of waiting times for elective general surgery and the appropriateness of prioritising patients. BMC Health Serv Res. 2007;7:32. Epub 20070228. doi: 10.1186/1472-6963-7-32. PubMed PMID: 17328816; PubMed Central PMCID: PMCPMC1847814.	Excluded from the final analysis	The article was excluded from the analysis because it was repetitive (duplicate article)
1429	Özdaş S, Bozkurt H. Factors Affecting the Development of Gallstones Following Laparoscopic Sleeve Gastrectomy. Obes Surg. 2019;29(10):3174-8. doi: 10.1007/s11695-019-03946-w. PubMed PMID: 31129884.	Excluded from the final analysis	The article was excluded from the analysis because it was repetitive (duplicate article)
1430	Paajanen H, Käkelä P, Suuronen S, Paajanen J, Juvonen P, Pihlajamäki J. Impact of obesity and associated diseases on outcome after laparoscopic cholecystectomy. Surg Laparosc Endosc Percutan Tech. 2012;22(6):509-13. doi: 10.1097/SLE.0b013e318270473b. PubMed PMID: 23238377.	Excluded from the final analysis	The article was excluded from the analysis because it was repetitive (duplicate article)
1431	Pan T, Zhang C, Liang J, Wang X, Di X, Zhou Y, et al. Association between life-ever gallstones and depressive symptoms in U.S. adults: a cross-sectional study. Sci Rep. 2024;14(1):18845. Epub 20240814. doi: 10.1038/s41598-024-69777-7. PubMed PMID: 39143232; PubMed Central PMCID: PMCPMC11325026.	Excluded from the final analysis	The article was excluded from the analysis because it was repetitive (duplicate article)

1432	Pantsyrev Iu M, Budzinskiĭ AA, Nozdrachev VI, Lagunchik BP, Konovalov A. [Therapeutic tactics in acute obstruction of the terminal part of the common bile duct]. Khirurgiia (Mosk). 1990;(10):3-8. PubMed PMID: 2283748.	Excluded from the final analysis	The article was excluded from the analysis because it was repetitive (duplicate article)
1433	Panwar U, Singh SK. Identification of Novel Pancreatic Lipase Inhibitors Using In Silico Studies. Endocr Metab Immune Disord Drug Targets. 2019;19(4):449-57. doi: 10.2174/1871530319666181128100903. PubMed PMID: 30484411.	Excluded from the final analysis	The article was excluded from the analysis because it was repetitive (duplicate article)
1434	Papavramidis TS, Michalopoulos A, Papadopoulos VN, Paramythiotis D, Karadimou V, Kokkinakis H, Fahantidis E. Emphysematous cholecystitis: a case report. Cases J. 2008;1(1):73. Epub 20080807. doi: 10.1186/1757-1626-1-73. PubMed PMID: 18687128; PubMed Central PMCID: PMCPMC2518129.	Excluded from the final analysis	The article was excluded from the analysis because it was repetitive (duplicate article)
1435	Paraf F, Fabiani JN, Laurian C, Bruneval P. [Ischemic cholecystitis from cholesterol crystal embolism]. Gastroenterol Clin Biol. 1999;23(5):577-80. PubMed PMID: 10429867.	Excluded from the final analysis	The article was excluded from the analysis because it was repetitive (duplicate article)
1436	Park JM, Park N, Lee SH, Han KD, Kang CD, Lee JM, et al. A population-based cohort study on risk factors for acute pancreatitis: A comparison by age group. Pancreatology. 2023;23(3):321-9. Epub 20230317. doi: 10.1016/j.pan.2023.03.004. PubMed PMID: 36964006.	Excluded from the final analysis	The article was excluded from the analysis because it was repetitive (duplicate article)
1437	Park S, Balasooriya J, Ncube T. A Rare Case of Gallstone Ileus: Bouveret Syndrome Presenting with Concurrent Gallstone Coleus. Case Rep Surg. 2020;2020:8844199. Epub 20201107. doi: 10.1155/2020/8844199. PubMed PMID: 33204566; PubMed Central PMCID: PMCPMC7666622.	Excluded from the final analysis	The article was excluded from the analysis because it was repetitive (duplicate article)
1438	Parker MA, Kragh N, Sandoval J, Erabti S, Soliman B. Surgical Management of Gallstone Ileus: Approach, Outcome, Case Report, and Literature Review. Cureus. 2024;16(9):e69930. Epub 20240922. doi: 10.7759/cureus.69930. PubMed PMID: 39439607; PubMed Central PMCID: PMCPMC11495830.	Excluded from the final analysis	The article was excluded from the analysis because it was repetitive (duplicate article)
1439	Parodi HC, Gutiérrez S, Lattanzi M, Martínez R, Colombato LO. [Value of laboratory tests and echography in the diagnosis of biliary disease in the initial phase of acute pancreatitis]. Acta Gastroenterol Latinoam. 1990;20(3):137-44. PubMed PMID: 2095097.	Excluded from the final analysis	The article was excluded from the analysis because it was repetitive (duplicate article)

1440	Pasławski M, Gwizdak J, Złomaniec J. The diagnostic value of different imaging modalities in evaluation of bowel obstruction. Ann Univ Mariae Curie Sklodowska Med. 2004;59(2):268-74. PubMed PMID: 16146091.	Excluded from the final analysis	The article was excluded from the analysis because it was repetitive (duplicate article)
1441	Pata F, Stamati G, Nardo B. Abdominal Pain and Hypotension in a 70-Year-Old Woman. Jama. 2023;329(18):1603-4. doi: 10.1001/jama.2023.4441. PubMed PMID: 37083972.	Excluded from the final analysis	The article was excluded from the analysis because it was repetitive (duplicate article)
1442	Patel AM, Yeola M, Mahakalkar C. Demographic and Risk Factor Profile in Patients of Gallstone Disease in Central India. Cureus. 2022;14(5):e24993. Epub 20220514. doi: 10.7759/cureus.24993. PubMed PMID: 35719762; PubMed Central PMCID: PMCPMC9190443.	Excluded from the final analysis	The article was excluded from the analysis because it was repetitive (duplicate article)
1443	Patel K, Devireddy N, Long C, Daya A, Cherneskie J, Krill K. When Blood Is Thicker Than Water: A Case of Acute Pancreatitis Secondary to Familial Hypertriglyceridemia. Cureus. 2024;16(1):e51511. Epub 20240102. doi: 10.7759/cureus.51511. PubMed PMID: 38304641; PubMed Central PMCID: PMCPMC10832547.	Excluded from the final analysis	The article was excluded from the analysis because it was repetitive (duplicate article)
1444	Patil SS, Pawar SC, Divekar V, Bakhshi RG. Transversus abdominis plane block for an emergency laparotomy in a high-risk, elderly patient. Indian J Anaesth. 2010;54(3):249-54. doi: 10.4103/0019-5049.65377. PubMed PMID: 20885876; PubMed Central PMCID: PMCPMC2933488.	Excluded from the final analysis	The article was excluded from the analysis because it was repetitive (duplicate article)
1445	Patriquin HB, DiPietro M, Barber FE, Teele RL. Sonography of thickened gallbladder wall: causes in children. AJR Am J Roentgenol. 1983;141(1):57-60. doi: 10.2214/ajr.141.1.57. PubMed PMID: 6602530.	Excluded from the final analysis	The article was excluded from the analysis because it was repetitive (duplicate article)
1446	Patrono D, Mazza E, Paraluppi G, Strignano P, David E, Romagnoli R, Salizzoni M. Liver transplantation for "mass-forming" sclerosing cholangitis after laparoscopic cholecystectomy. Int J Surg Case Rep. 2013;4(10):907-10. Epub 20130803. doi: 10.1016/j.ijscr.2013.07.021. PubMed PMID: 23995476; PubMed Central PMCID: PMCPMC3785926.	Excluded from the final analysis	The article was excluded from the analysis because it was repetitive (duplicate article)
1447	Pawlikowska L, Strautnieks S, Jankowska I, Czubkowski P, Emerick K, Antoniou A, et al. Differences in presentation and progression between severe FIC1 and BSEP deficiencies. J Hepatol. 2010;53(1):170-8. Epub 20100413. doi: 10.1016/j.jhep.2010.01.034. PubMed PMID: 20447715; PubMed Central PMCID: PMCPMC3042805.	Excluded from the final analysis	The article was excluded from the analysis because it was repetitive (duplicate article)

1448	Pedersen SB, Langsted A, Nordestgaard BG. Nonfasting Mild-to-Moderate Hypertriglyceridemia and Risk of Acute Pancreatitis. JAMA Intern Med. 2016;176(12):1834-42. doi: 10.1001/jamainternmed.2016.6875. PubMed PMID: 27820614.	Excluded from the final analysis	The article was excluded from the analysis because it was repetitive (duplicate article)
1449	Peng J, Zhang Y, Ling Q, Zhu L, Yao H. Case Report of Overlapping Pyloric Obstruction Due to Dichlorvos Poisoning and Cholelithiasis with Choledocholithiasis. Am J Case Rep. 2024;25:e943101. Epub 20240327. doi: 10.12659/ajcr.943101. PubMed PMID: 38532541; PubMed Central PMCID: PMCPMC10985417.	Excluded from the final analysis	The article was excluded from the analysis because it was repetitive (duplicate article)
1450	Pi-Sunyer FX. Short-term medical benefits and adverse effects of weight loss. Ann Intern Med. 1993;119(7 Pt 2):722-6. doi: 10.7326/0003-4819-119-7_part_2-199310011-00019. PubMed PMID: 8363205.	Excluded from the final analysis	The article was excluded from the analysis because it was repetitive (duplicate article)
1451	Pi-Sunyer FX. The medical risks of obesity. Obes Surg. 2002;12 Suppl 1:6s-11s. doi: 10.1007/bf03342140. PubMed PMID: 11969107.	Excluded from the final analysis	The article was excluded from the analysis because it was repetitive (duplicate article)
1452	Pineño-Flores C, Segura-Sampedro JJ, Morales-Soriano R, González Argente FX. Dorsal inflammatory mass secondary to lost stones after laparoscopic cholecystectomy. Rev Esp Enferm Dig. 2017;109(6):455-6. PubMed PMID: 28597676.	Excluded from the final analysis	The article was excluded from the analysis because it was repetitive (duplicate article)
1453	Ping WW, Puvan IS. Current status of oral contraceptive. Med J Malaysia. 1976;30(3):173-7. PubMed PMID: 822261.	Excluded from the final analysis	The article was excluded from the analysis because it was repetitive (duplicate article)
1454	Pitt HA. Hepatobiliary Hands of Hopkins. Ann Surg. 2018;267(2S Suppl 2):S34-s9. doi: 10.1097/sla.0000000000002606. PubMed PMID: 29206676.	Excluded from the final analysis	The article was excluded from the analysis because it was repetitive (duplicate article)
1455	Poo S, Pencavel TD, Jackson J, Jiao LR. Portal hypertension and chylous ascites complicating acute pancreatitis: the therapeutic value of portal vein stenting. Ann R Coll Surg Engl. 2018;100(1):e1-e3. Epub 20171019. doi: 10.1308/rcsann.2017.0078. PubMed PMID: 29046075; PubMed Central PMCID: PMCPMC5838657.	Excluded from the final analysis	The article was excluded from the analysis because it was repetitive (duplicate article)

1456	Popović M. [Anemia in chronic renal insufficiency--case report]. Med Pregl. 1999;52(6-8):279-81. PubMed PMID: 10518388.	Excluded from the final analysis	The article was excluded from the analysis because it was repetitive (duplicate article)
1457	Porterfield LM. Estrogen replacement therapy. Adv Clin Care. 1991;6(4):5. PubMed PMID: 1854417.	Excluded from the final analysis	The article was excluded from the analysis because it was repetitive (duplicate article)
1458	Prasaad PR, Shekhar S, Priyadharshini SA. Isolated non-necrotising granulomatous vasculitis of the gall bladder- a rare entity. J Clin Diagn Res. 2014;8(10):Fd01-2. Epub 20141020. doi: 10.7860/jcdr/2014/8599.4932. PubMed PMID: 25478351; PubMed Central PMCID: PMCPMC4253169.	Excluded from the final analysis	The article was excluded from the analysis because it was repetitive (duplicate article)
1459	Pribis P, Shukitt-Hale B. Cognition: the new frontier for nuts and berries. Am J Clin Nutr. 2014;100 Suppl 1:347s-52s. Epub 20140528. doi: 10.3945/ajcn.113.071506. PubMed PMID: 24871475.	Excluded from the final analysis	The article was excluded from the analysis because it was repetitive (duplicate article)
1460	Prinz RA, Aranha GV. The association of primary hyperparathyroidism and pancreatitis. Am Surg. 1985;51(6):325-9. PubMed PMID: 3994175.	Excluded from the final analysis	The article was excluded from the analysis because it was repetitive (duplicate article)
1461	Pu Y, Luo Y. Multi-modal imaging for the diagnosis of spontaneous visceral artery dissection: A case report. World J Gastrointest Surg. 2024;16(5):1430-5. doi: 10.4240/wjgs.v16.i5.1430. PubMed PMID: 38817294; PubMed Central PMCID: PMCPMC11135292.	Excluded from the final analysis	The article was excluded from the analysis because it was repetitive (duplicate article)
1462	Puppala S, Dodd GD, Fowler S, Arya R, Schneider J, Farook VS, et al. A genomewide search finds major susceptibility loci for gallbladder disease on chromosome 1 in Mexican Americans. Am J Hum Genet. 2006;78(3):377-92. Epub 20060106. doi: 10.1086/500274. PubMed PMID: 16400619; PubMed Central PMCID: PMCPMC1380282.	Excluded from the final analysis	The article was excluded from the analysis because it was repetitive (duplicate article)
1463	Qi Q, Han Y, Xue C. [Influence of age on severe pancreatitis]. Zhonghua Wai Ke Za Zhi. 1995;33(9):542-4. PubMed PMID: 8731873.	Excluded from the final analysis	The article was excluded from the analysis because it was repetitive (duplicate article)

1464	Qin J, Han TQ, Cai XX, Jiang ZH, Yang XM, Zhang Y, et al. [Study on the characteristics of inheritance and epidemiology in one pedigree with gallstone disease]. Zhonghua Liu Xing Bing Xue Za Zhi. 2005;26(6):448-50. PubMed PMID: 16185464.	Excluded from the final analysis	The article was excluded from the analysis because it was repetitive (duplicate article)
1465	Qin J, Han TQ, Fei J, Jiang ZY, Zhang Y, Yang SY, et al. [Risk factors of familial gallstone disease: study of 135 pedigrees]. Zhonghua Yi Xue Za Zhi. 2005;85(28):1966-9. PubMed PMID: 16313772.	Excluded from the final analysis	The article was excluded from the analysis because it was repetitive (duplicate article)
1466	Qureshi I, Awad ZT. Predictors of failure of the laparoscopic approach for the management of small bowel obstruction. Am Surg. 2010;76(9):947-50. PubMed PMID: 20836340.	Excluded from the final analysis	The article was excluded from the analysis because it was repetitive (duplicate article)
1467	Rai R, Nagral S, Nagral A. Surgery in a patient with liver disease. J Clin Exp Hepatol. 2012;2(3):238-46. Epub 20120921. doi: 10.1016/j.jceh.2012.05.003. PubMed PMID: 25755440; PubMed Central PMCID: PMCPMC3940091.	Excluded from the final analysis	The article was excluded from the analysis because it was repetitive (duplicate article)
1468	Rajvanshi P, Atac BS, Seno R, Gupta S. Gallbladder vasculitis associated with type-1 cryoglobulinemia. Dig Dis Sci. 2001;46(2):296-300. doi: 10.1023/a:1005648715013. PubMed PMID: 11281178.	Excluded from the final analysis	The article was excluded from the analysis because it was repetitive (duplicate article)
1469	Rana SS, Kumar A, Lal A, Sharma R, Kang M, Gorsi U, Gupta R. Safety and efficacy of angioembolisation followed by endoscopic ultrasound guided transmural drainage for pancreatic fluid collections associated with arterial pseudoaneurysm. Pancreatology. 2017;17(5):658-62. Epub 20170819. doi: 10.1016/j.pan.2017.08.002. PubMed PMID: 28843715.	Excluded from the final analysis	The article was excluded from the analysis because it was repetitive (duplicate article)
1470	Rana SS, Sharma R, Ahmed SU, Gupta R. Endoscopic ultrasound-guided transmural drainage of walled-off pancreatic necrosis in patients with portal hypertension and intra-abdominal collaterals. Indian J Gastroenterol. 2017;36(5):400-4. Epub 20171002. doi: 10.1007/s12664-017-0792-y. PubMed PMID: 28971378.	Excluded from the final analysis	The article was excluded from the analysis because it was repetitive (duplicate article)
1471	Rappaport WD, Gordon P, Warneke JA, Neal D, Hunter GC. Contraindications and complications of laparoscopic cholecystectomy. Am Fam Physician. 1994;50(8):1707-11, 14. PubMed PMID: 7977000.	Excluded from the final analysis	The article was excluded from the analysis because it was repetitive (duplicate article)

1472	Rathi P, Nair S. An Audit of Extrahepatic Portal Vein Obstruction: Experience from Tertiary Referral Center. J Assoc Physicians India. 2023;71(7):11-2. doi: 10.59556/japi.71.0281. PubMed PMID: 37449688.	Excluded from the final analysis	The article was excluded from the analysis because it was repetitive (duplicate article)
1473	Reddy SK, Zhan M, Alexander HR, El-Kamary SS. Nonalcoholic fatty liver disease is associated with benign gastrointestinal disorders. World J Gastroenterol. 2013;19(45):8301-11. doi: 10.3748/wjg.v19.i45.8301. PubMed PMID: 24363521; PubMed Central PMCID: PMCPMC3857453.	Excluded from the final analysis	The article was excluded from the analysis because it was repetitive (duplicate article)
1474	Régent D, Laurent V, Meyer-Bisch L, Barbary-Lefèvre C, Corby-Ciprian S, Mathias J. [Biliary colic: imaging diagnosis]. J Radiol. 2006;87(4 Pt 2):413-29. doi: 10.1016/s0221-0363(06)74024-0. PubMed PMID: 16691173.	Excluded from the final analysis	The article was excluded from the analysis because it was repetitive (duplicate article)
1475	Ronni-Sivula H. The state of health of patients previously operated on for primary hyperparathyroidism compared with randomized controls. Ann Chir Gynaecol. 1985;74(2):60-5. PubMed PMID: 4026176.	Excluded from the final analysis	The article was excluded from the analysis because it was repetitive (duplicate article)
1476	Ros E. Health benefits of nut consumption. Nutrients. 2010;2(7):652-82. Epub 20100624. doi: 10.3390/nu2070652. PubMed PMID: 22254047; PubMed Central PMCID: PMCPMC3257681.	Excluded from the final analysis	The article was excluded from the analysis because it was repetitive (duplicate article)
1477	Ros E, Armengol X, Grande L, Toledo-Pimentel V, Lacima G, Sanz G. Chest pain at rest in patients with coronary artery disease. Myocardial ischemia, esophageal dysfunction, or panic disorder? Dig Dis Sci. 1997;42(7):1344-53. doi: 10.1023/a:1018821417134. PubMed PMID: 9246027.	Excluded from the final analysis	The article was excluded from the analysis because it was repetitive (duplicate article)
1478	Rosato V, Bosetti C, Dal Maso L, Montella M, Serraino D, Negri E, La Vecchia C. Medical conditions, family history of cancer, and the risk of biliary tract cancers. Tumori. 2016;2016(3):252-7. Epub 20150804. doi: 10.5301/tj.5000397. PubMed PMID: 26350180.	Excluded from the final analysis	The article was excluded from the analysis because it was repetitive (duplicate article)
1479	Rosenberg JB, Hutcheson KA. Pediatric sickle cell retinopathy: correlation with clinical factors. J aapos. 2011;15(1):49-53. doi: 10.1016/j.jaapos.2010.11.014. PubMed PMID: 21397806.	Excluded from the final analysis	The article was excluded from the analysis because it was repetitive (duplicate article)

1480	Zobel MJ, Stewart L. Hyponatremia is associated with more severe biliary disease. World J Gastrointest Surg. 2020;12(2):45-54. doi: 10.4240/wjgs.v12.i2.45. PubMed PMID: 32128028; PubMed Central PMCID: PMCPMC7044107.	Excluded from the final analysis	The article was excluded from the analysis because it was repetitive (duplicate article)
1481	Rumba R, Vanags A, Strumfa I, Pupkevics A, Pavars M. Laparoscopic Cholecystectomy for a Patient with a Lumboperitoneal Shunt: A Rare Case. Chirurgia (Bucur). 2016;111(3):263-5. PubMed PMID: 27452939.	Excluded from the final analysis	The article was excluded from the analysis because it was repetitive (duplicate article)
1482	Sachdeva S, Khan Z, Ansari MA, Khalique N, Anees A. Lifestyle and gallstone disease: scope for primary prevention. Indian J Community Med. 2011;36(4):263-7. doi: 10.4103/0970-0218.91327. PubMed PMID: 22279255; PubMed Central PMCID: PMCPMC3263145.	Excluded from the final analysis	The article was excluded from the analysis because it was repetitive (duplicate article)
1483	Sağlam F, Sivrikoz E, Alemdar A, Kamalı S, Arslan U, Güven H. Bouveret syndrome: A fatal diagnostic dilemma of gastric outlet obstruction. Ulus Travma Acil Cerrahi Derg. 2015;21(2):157-9. doi: 10.5505/tjtes.2015.62558. PubMed PMID: 25904280.	Excluded from the final analysis	The article was excluded from the analysis because it was repetitive (duplicate article)
1484	Samsonova NG, Zvenigorodskaia LA, Cherkashova EA, Lazebnik LB. [Intestinal dysbiosis and atherogenic dyslipidemia]. Eksp Klin Gastroenterol. 2010;(3):88-94. PubMed PMID: 20499450.	Excluded from the final analysis	The article was excluded from the analysis because it was repetitive (duplicate article)
1485	Sandhu SK, Choy G. Vasculitis of the gallbladder in early rheumatoid arthritis. BMJ Case Rep. 2013;2013. Epub 20130823. doi: 10.1136/bcr-2012-008228. PubMed PMID: 23975907; PubMed Central PMCID: PMCPMC3762444.	Excluded from the final analysis	The article was excluded from the analysis because it was repetitive (duplicate article)
1486	Saraswat VA, Rai P, Kumar T, Mohindra S, Dhiman RK. Endoscopic management of portal cavernoma cholangiopathy: practice, principles and strategy. J Clin Exp Hepatol. 2014;4(Suppl 1):S67-76. Epub 20131022. doi: 10.1016/j.jceh.2013.08.011. PubMed PMID: 25755598; PubMed Central PMCID: PMCPMC4244828.	Excluded from the final analysis	The article was excluded from the analysis because it was repetitive (duplicate article)
1487	Sarin SK, Guptan RC, Malhotra S. Increased frequency of gallstones in cirrhotic and non-cirrhotic portal hypertension. J Assoc Physicians India. 2002;50:518-22. PubMed PMID: 12164401.	Excluded from the final analysis	The article was excluded from the analysis because it was repetitive (duplicate article)

1488	Sasaki H, Tanaka H, Nio M. Current management of long-term survivors of biliary atresia: over 40 years of experience in a single center and review of the literature. Pediatr Surg Int. 2017;33(12):1327-33. Epub 20170927. doi: 10.1007/s00383-017-4163-7. PubMed PMID: 28956136.	Excluded from the final analysis	The article was excluded from the analysis because it was repetitive (duplicate article)
1489	Sasturkar SV, Agrawal N, Arora A, Kumar MPS, Kilambi R, Thapar S, Chattopadhyay TK. Laparoscopic cholecystectomy in patients with portal cavernoma without portal vein decompression. J Minim Access Surg. 2021;17(3):351-5. doi: 10.4103/jmas.JMAS_106_20. PubMed PMID: 32964890; PubMed Central PMCID: PMCPMC8270052.	Excluded from the final analysis	The article was excluded from the analysis because it was repetitive (duplicate article)
1490	Saw SM, Rajan U. The epidemiology of obesity: a review. Ann Acad Med Singap. 1997;26(4):489-93. PubMed PMID: 9395816.	Excluded from the final analysis	The article was excluded from the analysis because it was repetitive (duplicate article)
1491	Sayenko VF, Lavryk AS, Stetsenko OP. Report on bariatric surgery in the Ukraine. Obes Surg. 2000;10(1):54-7. doi: 10.1381/09608920060674120. PubMed PMID: 10715646.	Excluded from the final analysis	The article was excluded from the analysis because it was repetitive (duplicate article)
1492	Sbeit W, Abukaes H, Said Ahmad H, Sbeit M, Kalisky I, Katz L, et al. The possible association of proton pump inhibitor use with acute cholangitis in patients with choledocholithiasis: a multi-center study. Scand J Gastroenterol. 2023;58(1):83-7. Epub 20220805. doi: 10.1080/00365521.2022.2106150. PubMed PMID: 35930433.	Excluded from the final analysis	The article was excluded from the analysis because it was repetitive (duplicate article)
1493	Sbeit W, Greener T, Kadah A, Mari A, Goldin E, Mahamid M, Khoury T. Pancreatic and hepatobiliary manifestations of nonalcoholic fatty pancreatic disease: a referral multi-center experience. Eur J Gastroenterol Hepatol. 2021;33(1S Suppl 1):e297-e301. doi: 10.1097/meg.0000000000002041. PubMed PMID: 33600093.	Excluded from the final analysis	The article was excluded from the analysis because it was repetitive (duplicate article)
1494	Sbeit W, Sbeit M, Kalisky I, Katz L, Mari A, Khoury T. The Possible Association of Non-Alcoholic Fatty Liver Disease with Acute Cholangitis: A Retrospective Multicenter Cohort Study. Life (Basel). 2021;12(1). Epub 20211227. doi: 10.3390/life12010035. PubMed PMID: 35054428; PubMed Central PMCID: PMCPMC8779558.	Excluded from the final analysis	The article was excluded from the analysis because it was repetitive (duplicate article)

1495	Scarpioni R, Ricardi M, Albertazzi V, Melfa L. Treatment of dyslipidemia in chronic kidney disease: Effectiveness and safety of statins. World J Nephrol. 2012;1(6):184-94. doi: 10.5527/wjn.v1.i6.184. PubMed PMID: 24175258; PubMed Central PMCID: PMCPMC3782216.	Excluded from the final analysis	The article was excluded from the analysis because it was repetitive (duplicate article)
1496	Schreinemakers JM, van Dam PS, Seldenrijk CA, Biesma DH, Borel Rinkes IH. [The adrenocortical carcinoma, a tumour of wide clinical diversity]. Ned Tijdschr Geneeskd. 2004;148(43):2109-13. PubMed PMID: 15553352.	Excluded from the final analysis	The article was excluded from the analysis because it was repetitive (duplicate article)
1497	Seal ML. Cholecystitis occurring without stones. Postgrad Med. 1986;79(4):151-4, 8. doi: 10.1080/00325481.1986.11699320. PubMed PMID: 3513145.	Excluded from the final analysis	The article was excluded from the analysis because it was repetitive (duplicate article)
1498	Seftel HC. Diseases in urban and rural Black populations. S Afr Med J. 1977;51(5):121-3. PubMed PMID: 850843.	Excluded from the final analysis	The article was excluded from the analysis because it was repetitive (duplicate article)
1499	Segal L, Carter R, Zimmet P. The cost of obesity: the Australian perspective. Pharmacoeconomics. 1994;5(Suppl 1):45-52. doi: 10.2165/00019053-199400051-00009. PubMed PMID: 10147249.	Excluded from the final analysis	The article was excluded from the analysis because it was repetitive (duplicate article)
1500	Sejeeni NF, Alfhmi S, Aljahdali S, Alzahrani S, Jaha R. Kawasaki Disease With Acute Acalculous Cholecystitis: A Case Report. Cureus. 2023;15(12):e49789. Epub 20231201. doi: 10.7759/cureus.49789. PubMed PMID: 38161523; PubMed Central PMCID: PMCPMC10757816.	Excluded from the final analysis	The article was excluded from the analysis because it was repetitive (duplicate article)
1501	Sekine K, Nagata N, Sakamoto K, Arai T, Shimbo T, Shinozaki M, et al. Abdominal visceral fat accumulation measured by computed tomography associated with an increased risk of gallstone disease. J Gastroenterol Hepatol. 2015;30(8):1325-31. doi: 10.1111/jgh.12965. PubMed PMID: 25869941.	Excluded from the final analysis	The article was excluded from the analysis because it was repetitive (duplicate article)
1502	Sequeira C, Santos I, Lopes S, Carvalheiro V, Mangualde J, Oliveira AP. Gastroduodenal artery pseudoaneurysm presenting with hyperamylasemia. Rev Esp Enferm Dig. 2023;115(9):535-6. doi: 10.17235/reed.2022.9407/2022. PubMed PMID: 36537340.	Excluded from the final analysis	The article was excluded from the analysis because it was repetitive (duplicate article)

1503	Serin H, Yilmaz YK, Turan Y, Arslan E, Erkoç MF, Doğan A, Celikbilek M. The association between gallstone disease and plaque in the abdominopelvic arteries. J Res Med Sci. 2017;22:11. Epub 20170127. doi: 10.4103/1735-1995.199087. PubMed PMID: 28458703; PubMed Central PMCID: PMCPMC5367247.	Excluded from the final analysis	The article was excluded from the analysis because it was repetitive (duplicate article)
1504	Sethi H, Peddu P, Prachalias A, Kane P, Karani J, Rela M, Heaton N. Selective embolization for bleeding visceral artery pseudoaneurysms in patients with pancreatitis. Hepatobiliary Pancreat Dis Int. 2010;9(6):634-8. PubMed PMID: 21134834.	Excluded from the final analysis	The article was excluded from the analysis because it was repetitive (duplicate article)
1505	Shanti I, Samardali M, Bambhroliya Z, Alhusari L. Post-endoscopic Retrograde Cholangiopancreatography Hemorrhagic Pancreatitis in a Young Female: A Case Report. Cureus. 2024;16(5):e60929. Epub 20240523. doi: 10.7759/cureus.60929. PubMed PMID: 38910698; PubMed Central PMCID: PMCPMC11193661.	Excluded from the final analysis	The article was excluded from the analysis because it was repetitive (duplicate article)
1506	Sharma AK. External biliary fistula. Trop Gastroenterol. 2001;22(3):163-8. PubMed PMID: 11681114.	Excluded from the final analysis	The article was excluded from the analysis because it was repetitive (duplicate article)
1507	Sharma M, Ponnusamy RP. Is balloon sweeping detrimental in portal biliopathy? A report of 3 cases. Gastrointest Endosc. 2009;70(1):171-3. Epub 20090505. doi: 10.1016/j.gie.2008.11.002. PubMed PMID: 19409559.	Excluded from the final analysis	The article was excluded from the analysis because it was repetitive (duplicate article)
1508	Sharma V, Sharma A, Aggarwal A, Bhardwaj G, Aggarwal S. Acute pancreatitis in a patient with vivax malaria. Jop. 2012;13(2):215-6. Epub 20120310. PubMed PMID: 22406604.	Excluded from the final analysis	The article was excluded from the analysis because it was repetitive (duplicate article)
1509	Sharpe JC, Marxer WL. Physical examination of well persons. what is "adequate" for detection of unsuspected disease? Calif Med. 1962;96(1):35-40. PubMed PMID: 13911411; PubMed Central PMCID: PMCPMC1574759.	Excluded from the final analysis	The article was excluded from the analysis because it was repetitive (duplicate article)
1510	Shearman DJ. The treatment of dyspepsia. Aust Fam Physician. 1977;6(8):1028-32. PubMed PMID: 921613.	Excluded from the final analysis	The article was excluded from the analysis because it was repetitive (duplicate article)

1511	Sher PP. Drug interference with laboratory tests: oral contraceptives. Drug Ther (NY). 1977;2(10):61-3. PubMed PMID: 12260155.	Excluded from the final analysis	The article was excluded from the analysis because it was repetitive (duplicate article)
1512	Shibata A, Ogimoto I, Kurozawa Y, Nose T, Yoshimura T, Suzuki H, et al. Past medical history and risk of death due to hepatocellular carcinoma, univariate analysis of JACC study data. Kurume Med J. 2003;50(3-4):109-19. doi: 10.2739/kurumemedj.50.109. PubMed PMID: 14768473.	Excluded from the final analysis	The article was excluded from the analysis because it was repetitive (duplicate article)
1513	Simon-Vermot I, Keller U. [Consequences and complications of obesity]. Ther Umsch. 2000;57(8):493-7. doi: 10.1024/0040-5930.57.8.493. PubMed PMID: 11026085.	Excluded from the final analysis	The article was excluded from the analysis because it was repetitive (duplicate article)
1514	Sinha AC, Singh PM, Bhat S. Are we operating too late? Mortality Analysis and Stochastic Simulation of Costs Associated with Bariatric Surgery: Reconsidering the BMI Threshold. Obes Surg. 2016;26(1):219-28. doi: 10.1007/s11695-015-1934-x. PubMed PMID: 26487650.	Excluded from the final analysis	The article was excluded from the analysis because it was repetitive (duplicate article)
1515	Sinha SR, Prakash P. Prevalence of Thyroid Disorder in Gallstone Disease Patients: A Cross-Sectional Study. Cureus. 2024;16(1):e52422. Epub 20240117. doi: 10.7759/cureus.52422. PubMed PMID: 38371154; PubMed Central PMCID: PMCPMC10870244.	Excluded from the final analysis	The article was excluded from the analysis because it was repetitive (duplicate article)
1516	Smedema JP, Louw VJ. Pulmonary arterial hypertension after splenectomy for hereditary spherocytosis. Cardiovasc J Afr. 2007;18(2):84-9. PubMed PMID: 17497044.	Excluded from the final analysis	The article was excluded from the analysis because it was repetitive (duplicate article)
1517	Smith FC, Gwynn BR. Direct access surgery. Ann R Coll Surg Engl. 1995;77(2):94-6. PubMed PMID: 7793823; PubMed Central PMCID: PMCPMC2502149.	Excluded from the final analysis	The article was excluded from the analysis because it was repetitive (duplicate article)
1518	Sneineh MA, Harel L, Elnasasra A, Razin H, Rotmensh A, Moscovici S, et al. Increased Incidence of Symptomatic Cholelithiasis After Bariatric Roux-En-Y Gastric Bypass and Previous Bariatric Surgery: a Single Center Experience. Obes Surg. 2020;30(3):846-50. doi: 10.1007/s11695-019-04366-6. PubMed PMID: 31901127.	Excluded from the final analysis	The article was excluded from the analysis because it was repetitive (duplicate article)

1519	Sninsky BC, Sehgal PD, Hinshaw JL, McDermott JC, Nakada SY. Expanding endourology for biliary stone disease: the efficacy of intracorporeal lithotripsy on refractory biliary calculi. J Endourol. 2014;28(7):877-80. Epub 20140411. doi: 10.1089/end.2014.0083. PubMed PMID: 24617771.	Excluded from the final analysis	The article was excluded from the analysis because it was repetitive (duplicate article)
1520	Snow RE, Vandewater SL. GALL-STONES AND ANAESTHETICS. Can Anaesth Soc J. 1963;10:555-66. doi: 10.1007/bf03002089. PubMed PMID: 14076155.	Excluded from the final analysis	The article was excluded from the analysis because it was repetitive (duplicate article)
1521	Soliman S. Hypertriglyceridemia-Induced Pancreatitis With Rapid Response to Insulin Therapy. J Med Cases. 2021;12(1):23-6. Epub 20201118. doi: 10.14740/jmc3595. PubMed PMID: 34434423; PubMed Central PMCID: PMCPMC8383640.	Excluded from the final analysis	The article was excluded from the analysis because it was repetitive (duplicate article)
1522	Sommerfleck F, Schneeberger E, Citera G. Comorbidities in Argentine patients with axial spondyloarthritis: Is nephrolithiasis associated with this disease? Eur J Rheumatol. 2018;5(3):169-72. Epub 20180622. doi: 10.5152/eurjrheum.2018.18002. PubMed PMID: 30071942; PubMed Central PMCID: PMCPMC6116842.	Excluded from the final analysis	The article was excluded from the analysis because it was repetitive (duplicate article)
1523	Somogyi L, Martin SP, Ulrich CD. Recurrent Acute Pancreatitis. Curr Treat Options Gastroenterol. 2001;4(5):361-8. doi: 10.1007/s11938-001-0001-y. PubMed PMID: 11560783.	Excluded from the final analysis	The article was excluded from the analysis because it was repetitive (duplicate article)
1524	Song ST, Cai LY, Zeng X, Xie WF. Gut Microbial Profile in Asymptomatic Gallstones. Front Microbiol. 2022;13:882265. Epub 20220613. doi: 10.3389/fmicb.2022.882265. PubMed PMID: 35770155; PubMed Central PMCID: PMCPMC9234526.	Excluded from the final analysis	The article was excluded from the analysis because it was repetitive (duplicate article)
1525	Song ST, Shi J, Wang XH, Guo YB, Hu PF, Zhu F, et al. Prevalence and risk factors for gallstone disease: A population-based cross-sectional study. J Dig Dis. 2020;21(4):237-45. Epub 20200415. doi: 10.1111/1751-2980.12857. PubMed PMID: 32166900.	Excluded from the final analysis	The article was excluded from the analysis because it was repetitive (duplicate article)
1526	Starkov Iu G, Strekalovskiĭ VP, Vishnevskiĭ VA, Grigor'ian RS. [Diverticuli of duodenal papillar region and their role in development of choledocholithiasis and strictures of bile and pancreatic ducts]. Khirurgiia (Mosk). 2000;(3):10-3. PubMed PMID: 10761374.	Excluded from the final analysis	The article was excluded from the analysis because it was repetitive (duplicate article)

1527	Stefano GB, Miller J. Communication between animal cells and the plant foods they ingest: phyto-zooidal dependencies and signaling (Review). Int J Mol Med. 2002;10(4):413-21. PubMed PMID: 12239587.	Excluded from the final analysis	The article was excluded from the analysis because it was repetitive (duplicate article)
1528	Stewart L, Griffiss JM, Jarvis GA, Way LW. The association between body mass index and severe biliary infections: a multivariate analysis. Am J Surg. 2012;204(5):574-9. Epub 20120811. doi: 10.1016/j.amjsurg.2012.07.002. PubMed PMID: 22892201.	Excluded from the final analysis	The article was excluded from the analysis because it was repetitive (duplicate article)
1529	Stewart L, Griffiss JM, Way LW. Spectrum of gallstone disease in the veterans population. Am J Surg. 2005;190(5):746-51. doi: 10.1016/j.amjsurg.2005.07.014. PubMed PMID: 16226952.	Excluded from the final analysis	The article was excluded from the analysis because it was repetitive (duplicate article)
1530	Stewart L, Grifiss JM, Jarvis GA, Way LW. Elderly patients have more severe biliary infections: influence of complement-killing and induction of TNFalpha production. Surgery. 2008;143(1):103-12. Epub 20071203. doi: 10.1016/j.surg.2007.06.035. PubMed PMID: 18154938.	Excluded from the final analysis	The article was excluded from the analysis because it was repetitive (duplicate article)
1531	Stewart L, Oesterle AL, Griffiss JM, Jarvis GA, Aagaard B, Way LW. Gram-negative bacteria killed by complement are associated with more severe biliary infections and produce more tumor necrosis factor-alpha in sera. Surgery. 2002;132(2):408-14. doi: 10.1067/msy.2002.127423. PubMed PMID: 12219042.	Excluded from the final analysis	The article was excluded from the analysis because it was repetitive (duplicate article)
1532	Strömberg J, Sadr-Azodi O, Videhult P, Hammarqvist F, Sandblom G. Incidence and risk factors for symptomatic venous thromboembolism following cholecystectomy. Langenbecks Arch Surg. 2015;400(4):463-9. Epub 20150215. doi: 10.1007/s00423-015-1284-0. PubMed PMID: 25682056.	Excluded from the final analysis	The article was excluded from the analysis because it was repetitive (duplicate article)
1533	Suárez V, Puerta A, Santos LF, Pérez JM, Varón A, Botero RC. Portal hypertensive biliopathy: A single center experience and literature review. World J Hepatol. 2013;5(3):137-44. doi: 10.4254/wjh.v5.i3.137. PubMed PMID: 23556047; PubMed Central PMCID: PMCPMC3612573.	Excluded from the final analysis	The article was excluded from the analysis because it was repetitive (duplicate article)
1534	Sundaram SS, Sokol RJ. The Multiple Facets of ABCB4 (MDR3) Deficiency. Curr Treat Options Gastroenterol. 2007;10(6):495-503. doi: 10.1007/s11938-007-0049-4. PubMed PMID: 18221610; PubMed Central PMCID: PMCPMC3888315.	Excluded from the final analysis	The article was excluded from the analysis because it was repetitive (duplicate article)

1535	Suzuki N, Furuya R, Otsuka T, Miyazaki H, Okano H, Komatsu T, et al. Acute pancreatitis caused by duodenal bezoar and treated with endoscopic procedures. Acute Med Surg. 2022;9(1):e797. Epub 20220930. doi: 10.1002/ams2.797. PubMed PMID: 36203854; PubMed Central PMCID: PMCPMC9525622.	Excluded from the final analysis	The article was excluded from the analysis because it was repetitive (duplicate article)
1536	Swinburn B, Ashton T, Gillespie J, Cox B, Menon A, Simmons D, Birkbeck J. Health care costs of obesity in New Zealand. Int J Obes Relat Metab Disord. 1997;21(10):891-6. doi: 10.1038/sj.ijo.0800486. PubMed PMID: 9347407.	Excluded from the final analysis	The article was excluded from the analysis because it was repetitive (duplicate article)
1537	Syme RG, Thomas EJ. Massive hemoperitoneum from transhepatic perforation of the gallbladder: a rare complication of cholelithiasis. Surgery. 1989;105(4):556-9. PubMed PMID: 2928958.	Excluded from the final analysis	The article was excluded from the analysis because it was repetitive (duplicate article)
1538	Taher AT, Musallam KM, Cappellini MD. Thalassaemia intermedia: an update. Mediterr J Hematol Infect Dis. 2009;1(1):e2009004. Epub 20090829. doi: 10.4084/mjhid.2009.004. PubMed PMID: 21415986; PubMed Central PMCID: PMCPMC3033165.	Excluded from the final analysis	The article was excluded from the analysis because it was repetitive (duplicate article)
1539	Tang K, Ford B, Grasso SL, Swisher J. Infectious aortitis and managing it at a community military hospital. BMJ Case Rep. 2024;17(3). Epub 20240327. doi: 10.1136/bcr-2023-257509. PubMed PMID: 38538095; PubMed Central PMCID: PMCPMC10982747.	Excluded from the final analysis	The article was excluded from the analysis because it was repetitive (duplicate article)
1540	Teply BA, Wang H, Luber B, Sullivan R, Rifkind I, Bruns A, et al. Bipolar androgen therapy in men with metastatic castration-resistant prostate cancer after progression on enzalutamide: an open-label, phase 2, multicohort study. Lancet Oncol. 2018;19(1):76-86. Epub 20171214. doi: 10.1016/s1470-2045(17)30906-3. PubMed PMID: 29248236; PubMed Central PMCID: PMCPMC5875180.	Excluded from the final analysis	The article was excluded from the analysis because it was repetitive (duplicate article)
1541	Thorbjarnarson B, Glenn F. COMPLICATIONS OF BILIARY TRACT SURGERY. Surg Clin North Am. 1964;44:431-7. doi: 10.1016/s0039-6109(16)37240-1. PubMed PMID: 14180331.	Excluded from the final analysis	The article was excluded from the analysis because it was repetitive (duplicate article)
1542	Tiseo D, Borrelli F, Gentile I, Benassai G, Quarto G, Borgia G. [Cystic echinococcosis in humans: our clinic experience]. Parassitologia. 2004;46(1-2):45-51. PubMed PMID: 15305685.	Excluded from the final analysis	The article was excluded from the analysis because it was repetitive (duplicate article)

1543	Tokur O, Aydın S, Kantarci M. Commentary on "Gallstone associated celiac trunk thromboembolisms complicated with splenic infarction: A case report". World J Clin Cases. 2022;10(32):12059-61. doi: 10.12998/wjcc.v10.i32.12059. PubMed PMID: 36405298; PubMed Central PMCID: PMCPMC9669843.	Excluded from the final analysis	The article was excluded from the analysis because it was repetitive (duplicate article)
1544	Tracey JY, Moossa AR. [Surgical treatment of benign lesions and strictures of the bile ducts]. Chirurg. 2006;77(4):315-24. doi: 10.1007/s00104-006-1168-5. PubMed PMID: 16557406.	Excluded from the final analysis	The article was excluded from the analysis because it was repetitive (duplicate article)
1545	Trovato GM, Catalano D, Sciacchitano G, Zuccalà G, Iannetti E. Resistive index of renal artery and blood pressure in postmenopausal women. Maturitas. 2002;41(3):223-30. doi: 10.1016/s0378-5122(01)00290-0. PubMed PMID: 11886768.	Excluded from the final analysis	The article was excluded from the analysis because it was repetitive (duplicate article)
1546	Trucco G, Chiusa L, Tandoi F, Bertero L. First report of a gallbladder hemangioma coexisting with gallstones: a case report and literature review of a rare finding. BMC Surg. 2022;22(1):128. Epub 20220406. doi: 10.1186/s12893-022-01554-7. PubMed PMID: 35382806; PubMed Central PMCID: PMCPMC8985283.	Excluded from the final analysis	The article was excluded from the analysis because it was repetitive (duplicate article)
1547	Tsomidis I, Leonidou K, Papachristodoulou A, Rafailidis V, Prassopoulos P. A rare cause of acute pancreatitis: ischemia caused by free-floating intraluminal aortic thrombus. Hippokratia. 2021;25(3):138-40. PubMed PMID: 36683902; PubMed Central PMCID: PMCPMC9851138.	Excluded from the final analysis	The article was excluded from the analysis because it was repetitive (duplicate article)
1548	Udekwu PO, Sullivan WG. Contemporary experience with cholecystectomy: establishing 'benchmarks' two decades after the introduction of laparoscopic cholecystectomy. Am Surg. 2013;79(12):1253-7. PubMed PMID: 24351351.	Excluded from the final analysis	The article was excluded from the analysis because it was repetitive (duplicate article)
1549	Utian WH. Estrogen replacement in the menopause. Obstet Gynecol Annu. 1979;8:369-91. PubMed PMID: 390456.	Excluded from the final analysis	The article was excluded from the analysis because it was repetitive (duplicate article)
1550	Utter A, Goss F. Exercise and gall bladder function. Sports Med. 1997;23(4):218-27. doi: 10.2165/00007256-199723040-00002. PubMed PMID: 9160479.	Excluded from the final analysis	The article was excluded from the analysis because it was repetitive (duplicate article)

1551	van Boxel EJ, Rahman S, Lai K, Boulos N, Davis N. Semaglutide treatment for children with obesity: an observational study. Arch Dis Child. 2024;109(10):822-5. Epub 20240925. doi: 10.1136/archdischild-2023-326687. PubMed PMID: 38471743.	Excluded from the final analysis	The article was excluded from the analysis because it was repetitive (duplicate article)
1552	Van De Walle P, Van Outryve L. Hand-assisted laparoscopic vertical banded gastroplasty: technique and analysis of the first 140 cases. Obes Surg. 2002;12(5):628-33. doi: 10.1381/096089202321019585. PubMed PMID: 12448382.	Excluded from the final analysis	The article was excluded from the analysis because it was repetitive (duplicate article)
1553	vanSonnenberg E, Casola G, Varney RR, Zakko S, Wittich GR, Cox J, Hofmann AF. Interventional radiology in the gallbladder. Radiographics. 1989;9(1):39-49. doi: 10.1148/radiographics.9.1.2643821. PubMed PMID: 2643821.	Excluded from the final analysis	The article was excluded from the analysis because it was repetitive (duplicate article)
1554	vanSonnenberg E, Casola G, Zakko SF, Varney RR, Cox J, Wittich GR, Hofmann AF. Gallbladder and bile duct stones: percutaneous therapy with primary MTBE dissolution and mechanical methods. Radiology. 1988;169(2):505-9. doi: 10.1148/radiology.169.2.3174999. PubMed PMID: 3174999.	Excluded from the final analysis	The article was excluded from the analysis because it was repetitive (duplicate article)
1555	vanSonnenberg E, D'Agostino HB, Goodacre BW, Sanchez RB, Casola G. Percutaneous gallbladder puncture and cholecystostomy: results, complications, and caveats for safety. Radiology. 1992;183(1):167-70. doi: 10.1148/radiology.183.1.1549666. PubMed PMID: 1549666.	Excluded from the final analysis	The article was excluded from the analysis because it was repetitive (duplicate article)
1556	vanSonnenberg E, Wittich GR, Casola G, Princenthal RA, Hofmann AF, Keightley A, Wing VW. Diagnostic and therapeutic percutaneous gallbladder procedures. Radiology. 1986;160(1):23-6. doi: 10.1148/radiology.160.1.3520647. PubMed PMID: 3520647.	Excluded from the final analysis	The article was excluded from the analysis because it was repetitive (duplicate article)
1557	Varma V, Behera A, Kaman L, Chattopadhyay S, Nundy S. Surgical management of portal cavernoma cholangiopathy. J Clin Exp Hepatol. 2014;4(Suppl 1):S77-84. Epub 20130917. doi: 10.1016/j.jceh.2013.07.005. PubMed PMID: 25755599; PubMed Central PMCID: PMCPMC4244827.	Excluded from the final analysis	The article was excluded from the analysis because it was repetitive (duplicate article)
1558	Vasiliadis K, Engelmann G, Sauer P, Weitz J, Schmidt J. Right porto-ovarian H-shunt for the surgical treatment of symptomatic portal biliopathy: a case report and literature review. HPB Surg. 2009;2009:152195. Epub 20090625. doi: 10.1155/2009/152195. PubMed PMID: 19584934; PubMed Central PMCID: PMCPMC2703746.	Excluded from the final analysis	The article was excluded from the analysis because it was repetitive (duplicate article)

1559	Vasiljević N, Pecelj-Gec M, Marinković J. [Health implications of obesity]. Srp Arh Celok Lek. 1997;125(9-10):299-302. PubMed PMID: 9340803.	Excluded from the final analysis	The article was excluded from the analysis because it was repetitive (duplicate article)
1560	Vitale GC, Zavaleta CM. Endoscopic retrograde cholangiopancreatography for surgeons. Semin Laparosc Surg. 2003;10(1):19-27. doi: 10.1177/107155170301000105. PubMed PMID: 12695806.	Excluded from the final analysis	The article was excluded from the analysis because it was repetitive (duplicate article)
1561	Walton JM, Abraham RJ, Perey BJ, MacGregor JH, Campbell DR. Hepatic artery pseudoaneurysms in acute pancreatitis. Can J Surg. 1991;34(4):377-80. PubMed PMID: 1868396.	Excluded from the final analysis	The article was excluded from the analysis because it was repetitive (duplicate article)
1562	Wang H, So H, Ko SW, Jung SW, Bang SJ, Park EJ. Gallstone Is Associated with Metabolic Factors and Exercise in Korea. Healthcare (Basel). 2022;10(8). Epub 20220724. doi: 10.3390/healthcare10081372. PubMed PMID: 35893194; PubMed Central PMCID: PMCPMC9329956.	Excluded from the final analysis	The article was excluded from the analysis because it was repetitive (duplicate article)
1563	Wang JY, Lu FH, Sun ZJ, Wu JS, Yang YC, Lee CT, Chang CJ. Gallstone disease associated with increased risk of arterial stiffness in a Taiwanese population. J Hum Hypertens. 2017;31(10):616-9. Epub 20170629. doi: 10.1038/jhh.2017.43. PubMed PMID: 28660886.	Excluded from the final analysis	The article was excluded from the analysis because it was repetitive (duplicate article)
1564	Wang L, Jiang T, Zhao YH. [One-year outcomes of laparoscopic single-anastomosis duodenal-ileal bypass with sleeve gastrectomy versus laparoscopic sleeve gastrectomy for the treatment of obesity and obesity-related metabolic diseases]. Zhonghua Wei Chang Wai Ke Za Zhi. 2021;24(12):1058-64. doi: 10.3760/cma.j.cn441530-20210126-00044. PubMed PMID: 34923788.	Excluded from the final analysis	The article was excluded from the analysis because it was repetitive (duplicate article)
1565	Wang L, Zhang Z, Wang Z, Jiang T. First study on the outcomes of biliopancreatic diversion with duodenal switch in Chinese patients with obesity. Front Surg. 2022;9:934434. Epub 20230106. doi: 10.3389/fsurg.2022.934434. PubMed PMID: 36684353; PubMed Central PMCID: PMCPMC9852535.	Excluded from the final analysis	The article was excluded from the analysis because it was repetitive (duplicate article)
1566	Wang S, Kou C, Liu Y, Li B, Tao Y, D'Arcy C, et al. Rural-urban differences in the prevalence of chronic disease in northeast China. Asia Pac J Public Health. 2015;27(4):394-406. Epub 20140922. doi: 10.1177/1010539514551200. PubMed PMID: 25246500.	Excluded from the final analysis	The article was excluded from the analysis because it was repetitive (duplicate article)

1567	Wang TF, Hwang SJ, Lee EY, Tsai YT, Lin HC, Li CP, et al. Gall-bladder wall thickening in patients with liver cirrhosis. J Gastroenterol Hepatol. 1997;12(6):445-9. doi: 10.1111/j.1440-1746.1997.tb00464.x. PubMed PMID: 9195402.	Excluded from the final analysis	The article was excluded from the analysis because it was repetitive (duplicate article)
1568	Waninger J, Salm R, van Hüllen C, Farthmann EH. [Laparoscopic cholecystectomy]. Fortschr Med. 1991;109(19):392-6. PubMed PMID: 1833296.	Excluded from the final analysis	The article was excluded from the analysis because it was repetitive (duplicate article)
1569	Wei CY, Chung TC, Chen CH, Lin CC, Sung FC, Chung WT, et al. Gallstone disease and the risk of stroke: a nationwide population-based study. J Stroke Cerebrovasc Dis. 2014;23(7):1813-20. Epub 20140621. doi: 10.1016/j.jstrokecerebrovasdis.2014.04.024. PubMed PMID: 24957305.	Excluded from the final analysis	The article was excluded from the analysis because it was repetitive (duplicate article)
1570	Welty TK. Health implications of obesity in American Indians and Alaska Natives. Am J Clin Nutr. 1991;53(6 Suppl):1616s-20s. doi: 10.1093/ajcn/53.6.1616S. PubMed PMID: 2031495.	Excluded from the final analysis	The article was excluded from the analysis because it was repetitive (duplicate article)
1571	Wirth J, Song M, Fung TT, Joshi AD, Tabung FK, Chan AT, et al. Diet-quality scores and the risk of symptomatic gallstone disease: a prospective cohort study of male US health professionals. Int J Epidemiol. 2018;47(6):1938-46. doi: 10.1093/ije/dyy210. PubMed PMID: 30312404; PubMed Central PMCID: PMCPMC6280928.	Excluded from the final analysis	The article was excluded from the analysis because it was repetitive (duplicate article)
1572	Wolff G, Liebscher C, Orban U. [Chronic gastritis and serum lipids, hypertension and cholelithiasis]. Dtsch Z Verdau Stoffwechselkr. 1988;48(3-4):149-52. PubMed PMID: 3234298.	Excluded from the final analysis	The article was excluded from the analysis because it was repetitive (duplicate article)
1573	Wood OB, Popovich NG. Nonpharmacologic treatment of obesity. J Am Pharm Assoc (Wash). 1996;Ns36(11):636-50. doi: 10.1016/s1086-5802(16)30148-6. PubMed PMID: 8952251.	Excluded from the final analysis	The article was excluded from the analysis because it was repetitive (duplicate article)
1574	Wu CY, Su CC, Huang HH, Wang YT, Wang CC. Gallstone associated celiac trunk thromboembolisms complicated with splenic infarction: A case report. World J Clin Cases. 2022;10(25):8968-73. doi: 10.12998/wjcc.v10.i25.8968. PubMed PMID: 36157657; PubMed Central PMCID: PMCPMC9477054.	Excluded from the final analysis	The article was excluded from the analysis because it was repetitive (duplicate article)

1575	Yang QY, Ouyang J, Yang JD. Sepsis as an important risk factor for gastrointestinal bleeding in acute coronary syndrome patients: Two case reports. Medicine (Baltimore). 2018;97(36):e12273. doi: 10.1097/md.0000000000012273. PubMed PMID: 30200168; PubMed Central PMCID: PMCPMC6133616.	Excluded from the final analysis	The article was excluded from the analysis because it was repetitive (duplicate article)
1576	Yang T, Zhong J, Zhang R, Xiao F, Wang Y, Tao H, Hong F. Different types and numbers metabolic abnormalities and risk of gallbladder stone disease in adults. Front Nutr. 2024;11:1443575. Epub 20240909. doi: 10.3389/fnut.2024.1443575. PubMed PMID: 39315007; PubMed Central PMCID: PMCPMC11416965.	Excluded from the final analysis	The article was excluded from the analysis because it was repetitive (duplicate article)
1577	Yassin MA, Soliman AT, De Sanctis V, Yassin KS, Abdulla MA. Final Height and Endocrine Complications in Patients with β-Thalassemia Intermedia: Our Experience in Non-Transfused Versus Infrequently Transfused Patients and Correlations with Liver Iron Content. Mediterr J Hematol Infect Dis. 2019;11(1):e2019026. Epub 20190501. doi: 10.4084/mjhid.2019.026. PubMed PMID: 31205630; PubMed Central PMCID: PMCPMC6548215.	Excluded from the final analysis	The article was excluded from the analysis because it was repetitive (duplicate article)
1578	Young SB, Arregui M, Singh K. HIDA scan ejection fraction does not predict sphincter of Oddi hypertension or clinical outcome in patients with suspected chronic acalculous cholecystitis. Surg Endosc. 2006;20(12):1872-8. doi: 10.1007/s00464-005-0245-z. PubMed PMID: 17031746.	Excluded from the final analysis	The article was excluded from the analysis because it was repetitive (duplicate article)
1579	Yuan Z, Meyerholz DK, Twait EC, Kempuraj D, Williard DE, Samuel I. Systemic inflammation with multiorgan dysfunction is the cause of death in murine ligation-induced acute pancreatitis. J Gastrointest Surg. 2011;15(10):1670-8. Epub 20110729. doi: 10.1007/s11605-011-1643-2. PubMed PMID: 21800226.	Excluded from the final analysis	The article was excluded from the analysis because it was repetitive (duplicate article)
1580	Yuksek YN, Akat AZ, Gozalan U, Daglar G, Pala Y, Canturk M, et al. Laparoscopic cholecystectomy under spinal anesthesia. Am J Surg. 2008;195(4):533-6. doi: 10.1016/j.amjsurg.2007.05.043. PubMed PMID: 18304510.	Excluded from the final analysis	The article was excluded from the analysis because it was repetitive (duplicate article)
1581	Zamani F, Sohrabi M, Alipour A, Motamed N, Saeedian FS, Pirzad R, et al. Prevalence and risk factors of cholelithiasis in Amol city, northern Iran: a population based study. Arch Iran Med. 2014;17(11):750-4. PubMed PMID: 25365614.	Excluded from the final analysis	The article was excluded from the analysis because it was repetitive (duplicate article)
1582	Zappia F, Petracca G, Talarico CA. Gallstone ileus. A case treated with minilaparotomy and a review of the literature. Ann Ital Chir. 2017;6. Epub 20170329. PubMed PMID: 28401880.	Excluded from the final analysis	The article was excluded from the analysis because it was repetitive (duplicate article)

1583	Zeng D, Wu H, Huang Q, Zeng A, Yu Z, Zhong Z. High Levels of Serum Triglyceride, Low-density Lipoprotein Cholesterol, Total Bile Acid, and Total Bilirubin are Risk Factors for Gallstones. Clin Lab. 2021;67(8). doi: 10.7754/Clin.Lab.2021.201228. PubMed PMID: 34383399.	Excluded from the final analysis	The article was excluded from the analysis because it was repetitive (duplicate article)
1584	Zhang Y, Sun L, Wang X, Chen Z. The association between hypertension and the risk of gallstone disease: a cross-sectional study. BMC Gastroenterol. 2022;22(1):138. Epub 20220326. doi: 10.1186/s12876-022-02149-5. PubMed PMID: 35346065; PubMed Central PMCID: PMCPMC8961935.	Excluded from the final analysis	The article was excluded from the analysis because it was repetitive (duplicate article)
1585	Zhao J, Tian L, Xia B, Mi N, He Q, Yang M, et al. Cholecystectomy is associated with a higher risk of irritable bowel syndrome in the UK Biobank: a prospective cohort study. Front Pharmacol. 2023;14:1244563. Epub 20231208. doi: 10.3389/fphar.2023.1244563. PubMed PMID: 38143491; PubMed Central PMCID: PMCPMC10749201.	Excluded from the final analysis	The article was excluded from the analysis because it was repetitive (duplicate article)
1586	Zheng C, Zhong X, Ma M, Zheng X, Jiang B, Zheng YP. Hyperlipidaemic acute pancreatitis complicated with multiple deep vein thromboses and pulmonary embolism: a case successfully salvaged by radiologic intervention. Curr Med Res Opin. 2021;37(1):53-7. Epub 20201203. doi: 10.1080/03007995.2020.1854702. PubMed PMID: 33222536.	Excluded from the final analysis	The article was excluded from the analysis because it was repetitive (duplicate article)
1587	Zheng Y, Xu M, Heianza Y, Ma W, Wang T, Sun D, et al. Gallstone disease and increased risk of mortality: Two large prospective studies in US men and women. J Gastroenterol Hepatol. 2018;33(11):1925-31. Epub 20180527. doi: 10.1111/jgh.14264. PubMed PMID: 29671893; PubMed Central PMCID: PMCPMC9015210.	Excluded from the final analysis	The article was excluded from the analysis because it was repetitive (duplicate article)
1588	Zheng Z, Zhang C, Yan J, Ruan Y, Zhao X, San X, et al. Diabetes mellitus is associated with hepatocellular carcinoma: a retrospective case-control study in hepatitis endemic area. PLoS One. 2013;8(12):e84776. Epub 20131226. doi: 10.1371/journal.pone.0084776. PubMed PMID: 24386416; PubMed Central PMCID: PMCPMC3873428.	Excluded from the final analysis	The article was excluded from the analysis because it was repetitive (duplicate article)
1589	Zhu JF. [Gallbladder contractile function in patients with portal hypertension]. Zhonghua Wai Ke Za Zhi. 1993;31(1):34-6. PubMed PMID: 8404340.	Excluded from the final analysis	The article was excluded from the analysis because it was repetitive (duplicate article)

1590	Zhu L, Aili A, Zhang C, Saiding A, Abudureyimu K. Prevalence of and risk factors for gallstones in Uighur and Han Chinese. World J Gastroenterol. 2014;20(40):14942-9. doi: 10.3748/wjg.v20.i40.14942. PubMed PMID: 25356055; PubMed Central PMCID: PMCPMC4209558.	Excluded from the final analysis	The article was excluded from the analysis because it was repetitive (duplicate article)
1591	Zhuang X, Li L. [A case - control study of gallstone disease in female population in Taicang]. Zhonghua Liu Xing Bing Xue Za Zhi. 2000;21(1):44-7. PubMed PMID: 11860758.	Excluded from the final analysis	The article was excluded from the analysis because it was repetitive (duplicate article)
1592	Bates T, Harrison M, Lowe D, Lawson C, Padley N. LONGITUDINAL-STUDY OF GALL STONE PREVALENCE AT NECROPSY. Gut. 1992;33(1):103-7. doi: 10.1136/gut.33.1.103. PubMed PMID: WOS:A1992HA21200020.	Excluded from the final analysis	The article was excluded from the analysis because it was repetitive (duplicate article)
1593	Bhattacharjee PK, Choudhury D, Rai H, Ram N, Chattopadhyay D, Roy RP. Spontaneous perforation of common bile duct: a rare complication of choledocholithiasis. Indian Journal of Surgery. 2009;71(2):92-4. doi: 10.1007/s12262-009-0024-5. PubMed PMID: WOS:000266440700008.	Excluded from the final analysis	The article was excluded from the analysis because it was repetitive (duplicate article)
1594	Birgani NB, Motamedi P, Kanaani L. A survey on the today's world problem of increasing obesity. Progress in Nutrition. 2019;21:22-32. doi: 10.23751/pn.v21i2-S.6415. PubMed PMID: WOS:000509707300003.	Excluded from the final analysis	The article was excluded from the analysis because it was repetitive (duplicate article)
1595	Bismuth H, Majno PE. Hepatobiliary surgery. Journal of Hepatology. 2000;32:208-24. doi: 10.1016/s0168-8278(00)80427-4. PubMed PMID: WOS:000085968400018.	Excluded from the final analysis	The article was excluded from the analysis because it was repetitive (duplicate article)
1596	Bohr URM, Annibale B, Franceschi F, Roccarina D, Gasbarrini A. Extragastric manifestations of <i>Helicobacter pylori</i> infection -: Other <i>Helicobacters</i>. Helicobacter. 2007;12:45-53. doi: 10.1111/j.1523-5378.2007.00533.x. PubMed PMID: WOS:000249021000009.	Excluded from the final analysis	The article was excluded from the analysis because it was repetitive (duplicate article)
1597	Bougard M, Barbier L, Godart B, Le Bayon-Bréard AG, Marques F, Salamé E. Management of biliary acute pancreatitis. Journal of Visceral Surgery. 2019;156(2):113-25. doi: 10.1016/j.jviscsurg.2018.08.002. PubMed PMID: WOS:000464972200006.	Excluded from the final analysis	The article was excluded from the analysis because it was repetitive (duplicate article)

1598	Bramucci A, Miceli F, Fontana A, Tusini N, Sereni G, Sassatelli R. Successful Endovascular and Endoscopic Treatment of a Symptomatic Celiac Artery Aneurysm for Obstructive Jaundice: A Clinical Case Report. Annals of Vascular Surgery. 2022;80. doi: 10.1016/j.avsg.2021.10.057. PubMed PMID: WOS:000819841600056.	Excluded from the final analysis	The article was excluded from the analysis because it was repetitive (duplicate article)
1599	Brown RC, Gray AR, Tey SL, Chisholm A, Burley V, Greenwood DC, Cade J. Associations between Nut Consumption and Health Vary between Omnivores, Vegetarians, and Vegans. Nutrients. 2017;9(11). doi: 10.3390/nu9111219. PubMed PMID: WOS:000416547200057.	Excluded from the final analysis	The article was excluded from the analysis because it was repetitive (duplicate article)
1600	Chan AW, Sabaratnam RM, Pillay Y. Massive gallstone in an asymptomatic Indigenous Canadian male: Case report and literature review. International Journal of Surgery Case Reports. 2020;72:429-32. doi: 10.1016/j.ijscr.2020.06.028. PubMed PMID: WOS:000548926300020.	Excluded from the final analysis	The article was excluded from the analysis because it was repetitive (duplicate article)
1601	Chen CH, Nien CK, Yang CC, Yeh YH. Association Between Nonalcoholic Fatty Liver Disease and Coronary Artery Calcification. Digestive Diseases and Sciences. 2010;55(6):1752-60. doi: 10.1007/s10620-009-0935-9. PubMed PMID: WOS:000278578800037.	Excluded from the final analysis	The article was excluded from the analysis because it was repetitive (duplicate article)
1602	Chen J, Sun YH, Fu T, Lu SY, Shi WM, Zhao JH, et al. Risk of incident cardiovascular disease among patients with gastrointestinal disorder: a prospective cohort study of 330751 individuals. European Heart Journal-Quality of Care and Clinical Outcomes. 2023. doi: 10.1093/ehjqcco/qcad059. PubMed PMID: WOS:001186209200001.	Excluded from the final analysis	The article was excluded from the analysis because it was repetitive (duplicate article)
1603	Chiu CC, Lee KJ, Weng SF, Yang YM, Lin YS. Gallstone is correlated with an increased risk of idiopathic sudden sensorineural hearing loss: a retrospective cohort study. Bmj Open. 2015;5(9). doi: 10.1136/bmjopen-2015-009018. PubMed PMID: WOS:000363484000084.	Excluded from the final analysis	The article was excluded from the analysis because it was repetitive (duplicate article)
1604	Colletti PM, Barakos JA, Ralls PW, Siegel ME, Halls JM. HEPATOBILIARY SCINTIGRAPHY AND SCINTIANGIOGRAPHY IN ABDOMINAL-TRAUMA. Clinical Nuclear Medicine. 1987;12(11):901-9. doi: 10.1097/00003072-198711000-00024. PubMed PMID: WOS:A1987L064600024.	Excluded from the final analysis	The article was excluded from the analysis because it was repetitive (duplicate article)
1605	Covello B, Miller J, Fourzali R. Splenic vein stenting for recurrent chylous ascites in sinistral portal hypertension: a case report. Cvir Endovascular. 2021;4(1). doi: 10.1186/s42155-021-00213-x. PubMed PMID: WOS:000703917000001.	Excluded from the final analysis	The article was excluded from the analysis because it was repetitive (duplicate article)

1606	Cucchiaro G, Rossitch JC, Bowie J, Branum GD, Niotis MT, Watters CR, Meyers WC. CLINICAL-SIGNIFICANCE OF ULTRASONOGRAPHICALLY DETECTED COINCIDENTAL GALLSTONES. Digestive Diseases and Sciences. 1990;35(4):417-21. doi: 10.1007/bf01536912. PubMed PMID: WOS:A1990CY65400001.	Excluded from the final analysis	The article was excluded from the analysis because it was repetitive (duplicate article)
1607	Cucchiaro G, Watters CR, Rossitch JC, Meyers WC. DEATHS FROM GALLSTONES - INCIDENCE AND ASSOCIATED CLINICAL FACTORS. Annals of Surgery. 1989;209(2):149-51. doi: 10.1097/00000658-198902000-00002. PubMed PMID: WOS:A1989T253400002.	Excluded from the final analysis	The article was excluded from the analysis because it was repetitive (duplicate article)
1608	Cunningham D, Mills PR, Quigley EMM, Patrick RS, Watkinson G, Mackenzie JF, Russell RI. HEPATIC GRANULOMAS - EXPERIENCE OVER A 10-YEAR PERIOD IN THE WEST OF SCOTLAND. Quarterly Journal of Medicine. 1982;51(202):162-70. PubMed PMID: WOS:A1982NR41000005.	Excluded from the final analysis	The article was excluded from the analysis because it was repetitive (duplicate article)
1609	Curry SJ, Krist AH, Owens DK, Barry MJ, Caughey AB, Davidson K, et al. Behavioral Weight Loss Interventions to Prevent Obesity-Related Morbidity and Mortality in Adults US Preventive Services Task Force Recommendation Statement. Jama-Journal of the American Medical Association. 2018;320(11):1163-71. doi: 10.1001/jama.2018.13022. PubMed PMID: WOS:000444878600016.	Excluded from the final analysis	The article was excluded from the analysis because it was repetitive (duplicate article)
1610	Cuschieri A, Lezoche E, Morino M, Croce E, Lacy A, Toouli J, et al. EAES multicenter prospective randomized trial comparing two-stage vs single-stage management of patients with gallstone disease and ductal calculi. Surgical Endoscopy-Ultrasound and Interventional Techniques. 1999;13(10):952-7. doi: 10.1007/s004649901145. PubMed PMID: WOS:000082887100001.	Excluded from the final analysis	The article was excluded from the analysis because it was repetitive (duplicate article)
1611	Czepiel J, Merc A, Jedrychowski M, Cholewczuk A, Biesiada G, Garlicki A. Septic shock caused by <i>Raoultella ornithinolytica</i> in a patient with Caroli syndrome. Reviews and Research in Medical Microbiology. 2024;35(4):227-9. doi: 10.1097/mrm.0000000000000388. PubMed PMID: WOS:001321268900002.	Excluded from the final analysis	The article was excluded from the analysis because it was repetitive (duplicate article)
1612	Das AK, Chiura A, Conlin MJ, Eschelman D, Bagley DH. Treatment of biliary calculi using holmium:yttrium aluminum garnet laser. Gastrointestinal Endoscopy. 1998;48(2):207-9. doi: 10.1016/s0016-5107(98)70167-1. PubMed PMID: WOS:000075342400016.	Excluded from the final analysis	The article was excluded from the analysis because it was repetitive (duplicate article)

1613	de Lorimier AA. Alcohol, wine, and health. American Journal of Surgery. 2000;180(5):357-61. doi: 10.1016/s0002-9610(00)00486-4. PubMed PMID: WOS:000166008200009.	Excluded from the final analysis	The article was excluded from the analysis because it was repetitive (duplicate article)
1614	Demircioglu MK, Demircioglu ZG, Önal C, Özler S. Difficult Management of Giant Gallstone Ileus of a Post-cardiopulmonary Resuscitation Patient: A Case Report. Cureus Journal of Medical Science. 2022;14(4). doi: 10.7759/cureus.23911. PubMed PMID: WOS:000785819800023.	Excluded from the final analysis	The article was excluded from the analysis because it was repetitive (duplicate article)
1615	Diehl AK. EPIDEMIOLOGY AND NATURAL-HISTORY OF GALLSTONE DISEASE. Gastroenterology Clinics of North America. 1991;20(1):1-19. PubMed PMID: WOS:A1991FB57200001.	Excluded from the final analysis	The article was excluded from the analysis because it was repetitive (duplicate article)
1616	Dixit M, Choudhuri G, Mittal B. Association of lipoprotein receptor, receptor-associated protein, and metabolizing enzyme gene polymorphisms with gallstone disease: A case-control study. Hepatology Research. 2006;36(1):61-9. doi: 10.1016/j.hepres.2006.05.005. PubMed PMID: WOS:000241198900010.	Excluded from the final analysis	The article was excluded from the analysis because it was repetitive (duplicate article)
1617	Doran H, Mihalache O, Bobirca F, Buga C, Patrascu T. Acute acalculous cholecystitis - difficulties of the diagnosis and treatment. Chirurgia. 2010;105(4):465-8. PubMed PMID: WOS:000281760700003.	Excluded from the final analysis	The article was excluded from the analysis because it was repetitive (duplicate article)
1618	Dotsenko YV, Naumov VG, Lyakishev AA, Bolotina MG, Bratchikova TV. Treatment of ischemic heart disease in postmenopausal women. Kardiologiya. 2001;41(3):64-70. PubMed PMID: WOS:000170939600017.	Excluded from the final analysis	The article was excluded from the analysis because it was repetitive (duplicate article)
1619	Drouard F, PassoneSzerzyna N, Berthou JC. Laparoscopic treatment of common bile duct stones. Hepato-Gastroenterology. 1997;44(13):16-21. PubMed PMID: WOS:A1997WK98800005.	Excluded from the final analysis	The article was excluded from the analysis because it was repetitive (duplicate article)
1620	Dumortier J, Vaillant E, Boillot O, Poncet G, Henry L, Scoazec JY, et al. Diagnosis and treatment of biliary obstruction caused by portal cavernoma. Endoscopy. 2003;35(5):446-50. doi: 10.1055/s-2003-38779. PubMed PMID: WOS:000182484600016.	Excluded from the final analysis	The article was excluded from the analysis because it was repetitive (duplicate article)

1621	Dvoretsky LI. Dr. Sergey P. Botkin's case sheet. Terapevticheskii Arkhiv. 2023;95(7):602-6. doi: 10.26442/00403660.2023.07.202310. PubMed PMID: WOS:001104956400003.	Excluded from the final analysis	The article was excluded from the analysis because it was repetitive (duplicate article)
1622	Ebert EC, Nagar M, Hagspiel KD. Gastrointestinal and Hepatic Complications of Sickle Cell Disease. Clinical Gastroenterology and Hepatology. 2010;8(6):483-9. doi: 10.1016/j.cgh.2010.02.016. PubMed PMID: WOS:000278747200007.	Excluded from the final analysis	The article was excluded from the analysis because it was repetitive (duplicate article)
1623	Eliakim R, Abulafia O, Sherer DM, Rayburn WF. Estrogen, progesterone and the gastrointestinal tract. Journal of Reproductive Medicine. 2000;45(10):781-8. PubMed PMID: WOS:000090105300001.	Excluded from the final analysis	The article was excluded from the analysis because it was repetitive (duplicate article)
1624	Elkerkary MA, Gbr H, Shaban H. Diagnostic accuracy of intraoperative cholangiography for detection of anatomical variations of the biliary system. Egyptian Journal of Surgery. 2021;40(1):131-9. doi: 10.4103/ejs.ejs_266_20. PubMed PMID: WOS:000656200800017.	Excluded from the final analysis	The article was excluded from the analysis because it was repetitive (duplicate article)
1625	Chen LY, Qiao QH, Zhang SC, Chen YH, Chao GQ, Fang LZ. Metabolic syndrome and gallstone disease. World Journal of Gastroenterology. 2012;18(31):4215-20. doi: 10.3748/wjg.v18.i31.4215. PubMed PMID: WOS:000307986800020.	Excluded from the final analysis	The article was excluded from the analysis because it was repetitive (duplicate article)
1626	Colletti PM, Barakos JA, Ralls PW, Siegel ME, Halls JM. HEPATOBILIARY SCINTIGRAPHY AND SCINTIANGIOGRAPHY IN ABDOMINAL-TRAUMA. Clinical Nuclear Medicine. 1987;12(11):901-9. doi: 10.1097/00003072-198711000-00024. PubMed PMID: WOS:A1987L064600024.	Excluded from the final analysis	The article was excluded from the analysis because it was repetitive (duplicate article)
